# *Brachyspira hyodysenteriae* and *B. pilosicoli* Proteins Recognized by Sera of Challenged Pigs

**DOI:** 10.3389/fmicb.2017.00723

**Published:** 2017-05-04

**Authors:** Vanessa Casas, Arantza Rodríguez-Asiain, Roberto Pinto-Llorente, Santiago Vadillo, Montserrat Carrascal, Joaquin Abian

**Affiliations:** ^1^CSIC/UAB Proteomics Laboratory, IIBB-CSIC, IDIBAPSBarcelona, Spain; ^2^Faculty of Medicine, Autonomous University of BarcelonaBarcelona, Spain; ^3^Departamento Sanidad Animal, Facultad de Veterinaria, Universidad de ExtremaduraCáceres, Spain

**Keywords:** *Brachyspira*, vaccine, immunoblot, mass spectrometry, antigen, flagellar protein, swine dysentery, spirochaetosis

## Abstract

The spirochetes *Brachyspira hyodysenteriae* and *B. pilosicoli* are pig intestinal pathogens that are the causative agents of swine dysentery (SD) and porcine intestinal spirochaetosis (PIS), respectively. Although some inactivated bacterin and recombinant vaccines have been explored as prophylactic treatments against these species, no effective vaccine is yet available. Immunoproteomics approaches hold the potential for the identification of new, suitable candidates for subunit vaccines against SD and PIS. These strategies take into account the gene products actually expressed and present in the cells, and thus susceptible of being targets of immune recognition. In this context, we have analyzed the immunogenic pattern of two *B. pilosicoli* porcine isolates (the Spanish farm isolate OLA9 and the commercial P43/6/78 strain) and one *B. hyodysenteriae* isolate (the Spanish farm V1). The proteins from the *Brachyspira* lysates were fractionated by preparative isoelectric focusing, and the fractions were analyzed by Western blot with hyperimmune sera from challenged pigs. Of the 28 challenge-specific immunoreactive bands detected, 21 were identified as single proteins by MS, while the other 7 were shown to contain several major proteins. None of these proteins were detected in the control immunoreactive bands. The proteins identified included 11 from *B. hyodysenteriae* and 28 from the two *B. pilosicoli* strains. *Eight* proteins were common to the *B. pilosicoli* strains (i.e., elongation factor G, aspartyl-tRNA synthase, biotin lipoyl, TmpB outer membrane protein, flagellar protein FlaA, enolase, PEPCK, and VspD), and enolase and PEPCK were common to both *species*. Many of the identified proteins were flagellar proteins or predicted to be located on the cell surface and some of them had been previously described as antigenic or as bacterial virulence factors. Here we report on the identification and semiquantitative data of these immunoreactive proteins which constitute a unique antigen collection from these bacteria.

## Introduction

Bacteria in the *Brachyspiraceae* family are gram-negative and spiral-shaped *Spirochaetes*. Members of this phylum are characterized by their unique motility and a loosely coiled morphology caused by the existence of periplasmic flagella (Charon and Goldstein, 2002). Species of the *Brachyspiraceae* family are anaerobic, host-associated intestinal bacteria in pigs, humans and other species, and can cause gastrointestinal pathologies and mortality (Stanton, 2006). Currently, the *Brachyspiraceae* comprises 16 species. *B. hyodysenteriae* and *B. pilosicoli* are well-known *Brachyspira* intestinal pathogens in pigs, responsible for swine dysentery (SD, a severe mucohaemorrhagic colitis) (Alvarez-Ordóñez et al., 2013) and porcine intestinal spirochaetosis (PIS, porcine spirochaetal diarrhea, a mild, non-haemorrhagic colitis), respectively (Trott et al., 1996; Stanton, 2006).

SD is produced by *B. hyodysenteriae*, a restricted pig-associated species, and is a disease present worldwide that has an important economic impact on the farming business. SD mainly affects pigs in the growing-finishing periods. This contributes to the high cost of the disease, which is associated not only with mortality, which is relatively low when animals are treated (Hamdy, 1974), but also with high morbidity, growth retardation and the need for continual in-feed medication.

Unlike most *Brachyspira* species, *B. pilosicoli* has a wide host range. It is zoonotic in pigs, poultry, dogs and humans (Hampson et al., 2006), in which it can lead to intestinal spirochaetosis (IS). In humans, the prevalence of IS is very uneven, much higher in developing regions than in industrialized regions (Tsinganou and Gebbers, 2010).

The treatment for the control of a *Brachyspira* infection involves the use of multiple antimicrobial agents (Alvarez-Ordóñez et al., 2013). Nevertheless, several studies have highlighted the increasing occurrence of *B. hyodysenteriae* and *B. pilosicoli* isolates resistant to these antibiotics in many countries (Molnar, 1996; Karlsson et al., 2001; Rohde et al., 2004; Hidalgo et al., 2009; Ohya and Sueyoshi, 2010; Pringle et al., 2012), greatly compromising the efficacy of this global treatment.

Since Joens et al. described that pigs that recovered from SD acquired immunological protection, and therefore rarely relapse when re-exposed to the infective agent (Joens et al., 1979), several endeavors have been launched to design a vaccine. Unfortunately, these attempts have not been successful to date, and no effective vaccine against *B. hyodysenteriae* or *B. pilosicoli* is available. Early approaches in this area included immunization with an inactivated bacterin (Hampson, 2000) or attenuated strains (Hyatt et al., 1994). In addition to conferring only partial protection in the best cases, these strategies entailed a cumbersome anaerobic-culture of *Brachyspira* which was not appropriate for large-scale production.

In recent years, the development of subunit vaccines based on *Brachyspira* recombinant proteins has also been explored. Molecules studied included flagellar proteins such as FlaA and FlaB (Boyden et al., 1989; Kent et al., 1989; Gabe et al., 1995; Ge and Charon, 1997) and some structural and metabolic proteins, such as outer-membrane proteins BmpB (La et al., 2004), SmpB (Holden et al., 2008), or variable surface proteins (Vsp) (Witchell et al., 2006, 2011). Similar to an attempt to formulate a vaccine based on the ferritin protein FtnA (Davis et al., 2005), none of these antigens provided enough protection for SD. On the other hand, several surface proteins of *B. pilosicoli* (ClpX and two putative oligopeptide-binding proteins) have been evaluated as candidates for vaccination against IS (Movahedi and Hampson, 2007, 2010).

The recent publication of the genome sequences of *B. hyodysenteriae* and *B. pilosicoli* provided a useful tool for the exploration of new candidates for inclusion in vaccination processes. To date, the genome sequence of 20 strains of *B. hyodysenteriae* (including the reference strain WA1, ATCC 49526) (Black et al., 2015) and three strains of *B. pilosicoli* (porcine isolates P43/6/78 and 95/1,000, and an avian isolate B2904) (Wanchanthuek et al., 2010; Mappley et al., 2012; Lin et al., 2013) have been published. The availability of these data enables extensive *in silico* analysis to identify vaccine candidates, which can then be expressed and tested together in a subunit vaccine. The potential of this reverse vaccinology approach was demonstrated by Song et al. who explored the development of a vaccine against SD using a partial genome sequence of the *B. hyodysenteriae* WA1 strain (Song et al., 2009). More recently, a list of 33 ORF candidates to vaccine targets has been patented (Bellgard et al., 2015). These genes were selected on the basis of their homology with known amino acid sequences of surface proteins, secreted proteins and virulence factors from other species.

Despite these important advances on the genomic level, its translation to proteomic knowledge of *B. hyodysenteriae* and *B. pilosicoli* is still a challenge. Undoubtedly, future studies focused on the description of *Brachyspira* proteomes will be necessary to design an effective vaccination strategy. In this regard, we recently characterized a subset of proteins exposed on the cell surface (surfaceome) of *B. hyodysenteriae* and *B. pilosicoli* (Casas et al., 2016). This will not only contribute to select good candidates for a vaccine, but will also impart biological knowledge about invasive and pathogenic mechanisms of *Brachyspira*.

In this study, we extended our proteomic approach to identify potential immunogenic proteins from *B. hyodysenteriae* and *B. pilosicoli*. For this purpose we studied the immunoproteome of two *B. pilosicoli* strains (the isolate OLA9 and the commercial ATCC strain P43/6/78) and one *B*. *hyodysenteriae* isolate (isolate V1). Cell lysates were fractionated using preparative off-gel isoelectrofocusing and the fractions were separated by SDS-PAGE. The gels were immunoblotted using pig immune-sera, and the reactive bands were identified by mass spectrometry. *Brachyspira* isolates came from Spanish farms. It was reported that during 2001-2003, more than 30% of commercial pig farms in Spain had at least one positive for *B. pilosicoli* or *B. hyodysenteriae* (Carvajal et al., 2006). There is thus a major concern in relation to intestinal diseases caused by *Brachyspira* species in the country (Osorio et al., 2012, 2013), which is the world's fourth largest producer of pig meat and where the porcine industry has a huge socioeconomic impact. We propose the reported proteins as suitable candidates to be included in vaccines for the treatment of SD and porcine IS.

## Materials and methods

### *Brachyspira* cultures

Two isolates of *Brachyspira pilosicoli* and *B. hyodysenteriae* (strains OLA9 and V1, respectively) and a commercial *B. pilosicoli* (ATCC strain P43/6/78, ATCC 51139) were used for the immunoproteomics study. Three additional *B. hyodysenteriae* strains (the commercial ATCC strains WA1 and B-78, and the isolate INF1) were included in the study of Vsp profiles.

The isolates came from Iberian pigs that showed disease symptoms on two different farms in the Badajoz province (Spain). The medium for the isolation of *Brachyspira* was based on the blood agar modified medium described by Calderaro et al. (2001, 2005), supplemented with antibiotics to remove most of the fecal micropopulation (Feberwee et al., 2008). The medium was composed of blood agar base n° 2 (40 g/l) supplemented with 5% defibrinated horse blood (50 ml) (Oxoid, Thermo Scientific, Waltham, MA, USA), beef extract (3 g/l), Bacto-peptone (5 g/l), (Difco, BD, Franklin Lakes, NJ, USA) and spectinomycin (0.2 g/l), spiramycin (0.025 g/l), rifampicin (0.012 g/l) vancomycin (6.2 g/l), and colistin (6.25 mg/l) (all from Sigma-Aldrich, St. Louis, MO, USA) and 810 ml distilled water. The plates were incubated for 4–7 days at 42°C in an anaerobic jar with CO_2_ produced by an AnaeroGen TM 3.5 L (Oxoid, Thermo Fisher Scientific, MA, USA). The colonies were examined by phase contrast microscopy (40x). The isolates were characterized by PCR using species-specific primers for *nox* (*B. hyodysenteriae*) and 16S rRNA (*B. pilosicoli*) as previously described (Casas et al., 2016) and stored at −80°C.

Blood agar solid subcultures of the isolates were seeded in Brain-heart infusion (BHI) broth (Laboratorios CONDA Pronadisa, Torrejón de Ardoz, Spain) enriched with horse serum (15%) and incubated with shaking in anaerobiosis jars at 4°C for 4–7 days. The grown cultures were centrifuged at 12,900 × g for 10 min, and the pellet washed twice with TE buffer (10 mM Tris pH 8.0, 1 mM EDTA, both from Sigma-Aldrich, St. Louis, MO, USA). All bacterial growth and handling procedures were carried out under biosafety level 2 conditions.

### *Brachyspira* cell lysate and protein quantification

Bacterial pellets (~150 mg) were resuspended in 500 μl denaturing lysis buffer containing 4% SDS, 0.1 M DTT and 100 mM Tris-HCl pH 7.5. After incubation in a Thermomixer (Eppendorf model Comfort, 600 rpm, 1 h, 95°C), the samples were homogenized in a Bullet Blender (Next Advance Storm, NY, USA) for 3 min at level 8, using 250 μl zirconium silicate beads (0.1 mm diameter, BioSpec, 11079101z). The beads were then pelleted by centrifugation at 14,000 × g for 3 min and the *Brachyspira* cell lysate was recovered from supernatant.

Prior to protein quantification, the excess SDS was removed from the sample using an SDS-Out ™ Precipitation Kit (Thermo Fisher Scientific). The sample was diluted with 100 mM Tris-HCl pH 7.5 (1/2 v/v) for this procedure and the SDS was precipitated with the precipitation reagent (1/20 v/v). After incubation in an ice bath for 20 min, the anionic detergent was pelleted by centrifugation (10,000 × g, 10 min) and the *Brachyspira* SDS-free lysate was recovered from supernatant. After clarification by an additional centrifugation (10,000 × g, 1 min) in a spin cup column, the cell lysate was aliquoted and kept at −40°C until use. A small aliquot was used to measure the protein concentration using the Bradford method (Bio-Rad Laboratories, CA, USA).

### Off-gel protein fractionation

Proteins in the *Brachyspira* lysates were fractionated by off-gel isoelectric focusing (Ros et al., 2002; Michel et al., 2003) using an OFFGEL Fractionator 3100 (Agilent Technologies CA, USA). For this procedure, 1 mg of protein (ca. 120–140 μl of cell lysate) was diluted to a final volume of 3.6 ml with a 1.25X protein off-gel stock solution (PROSS 1.25X: 7 M urea, 2 M thiourea, 1.5 M glycerol and 0.1 M DTT) supplemented with ampholytes (GE Healthcare Life Sciences, Barcelona, Spain). Large 24-cm dry strips with a wide immobilized pH gradient of 3–10 units (GE Healthcare Life Sciences) were used for the separation. After placing the 24 well frame, the strips were rehydrated with 50 μl/well of IPG strip rehydration solution (PROSS-ampholytes 1X) for 15 min, and the sample was loaded by pipetting 150 μl/well. To have enough material for an immuno-analysis, the experiments were carried out in triplicate so a total of 3 mg of each *Brachyspira* isolate was processed. The samples were focused for 36 h at 64 kVh with a maximum current of 50 μA and power of 200 mW (4,500 V final voltage). Twenty-four fractions were collected per sample. Three replicates were fractionated per sample. Triplicates of each fraction were pooled in a single tube, aliquoted and stored at −40°C.

### *Brachyspira*-challenge and control pig sera

Pig sera were provided by the company Laboratorios Larrasa S.L. (Badajoz, Spain) in the frame of a project funded by the Spanish Ministry of Economy and Competitivity (MINECO, IPT-2011-0735-010000). Laboratorios Larrasa S.L. was in compliance with Spanish legislation (R.D. 1201/2005 and Law 32/2007) and EU Council Guidelines (2003/65/CE) for the use of experimental animals. These sera were obtained from pigs from a healthy herd in Extremadura (South-Western Spain) which were inoculated at the age of 14 weeks with active viable cells of *B. hyodysenteriae* strain WA1 (ATCC 49526) or the *B. pilosicoli* porcine strain P43/6/78 (ATCC 51139). The serum was extracted from challenged animals at 12 weeks post-infection. Five different sera from each *Brachyspira* species challenge (*B. hyodysenteriae*-challenge serum 1–5 and *B. pilosicoli*-challenge serum 6–10) were used in this study.

### SDS-PAGE separation of off-gel IEF fractions

Five (for the WB analyses) or ten (for silver staining) microliters of the off-gel fractions were separated by SDS-PAGE. A sample of 20 μl of total lysate (input) was also prepared as a control. The samples were prepared in sample loading buffer (2% w/v SDS, 10% glycerol, 0.002% w/v bromophenol blue and 25 mM Tris-HCl pH 6.8), heated at 56°C for 30 min and resolved on 12 or 7.5%-polyacrylamide gels.

Six replicates were done for each separation. The protein bands in one replicate were visualized by mass spectrometry-compatible silver staining (Shevchenko et al., 1996; Casanovas et al., 2009). The other five replicates were used for immunoblotting with the appropriate antisera.

### Immunoblotting

After SDS-PAGE, the proteins in the gel were transferred to a nitrocellulose membrane using an iBlot ™ system (Life Technologies, CA, USA). Following Ponceau staining of the proteins (Sigma-Aldrich, St. Louis, MO, USA), the membrane was blocked with TBS-T (20 mM Tris-HCl pH 7.5, 150 mM NaCl and 0.2% Tween-20) containing 5% (w/v) skimmed milk (1 h, room temperature). The membrane was incubated with the appropriate pig serum diluted in blocking solution (1/3,000 for control serum and 1/5,000 for *Brachyspira* challenge serum) with gentle agitation (1 h, room temperature). The membranes were incubated (1 h) with a Rabbit Anti-pig IgG H&L horseradish peroxidase-conjugated secondary antibody (Abcam ab6777, 1/5,000 in blocking solution) and visualized with luminol.

### Characterization of immunoreactive bands

Optical density (OD) profiles of the immunoblots and SDS-PAGE gels for all fractions were acquired using ImageJ Version 1.47 (NIH). The profiles were measured between 15 and 100 kDa, and the OD values were normalized relative to the total lane intensity. The Rf value was determined with the Quantity One 1D Analysis Software (Bio-Rad) and position of each band expressed as its molecular mass calculated from the Rf value using molecular mass markers. Calculations using bands for the same protein in a gel showed an average coefficient of variation of 0.85% (*n* = 14) for the calculated mass. As a consequence, bands in different lanes of the same gel with a mass difference less than 1.7% (2 × standard deviation) were considered the same band and numbered accordingly.

Bands observed in at least two OD profiles of challenge sera but absent in control sera were designated as challenge-specific, differential immunoreactive bands. Some bands were also considered differential when also potentially present in only one of the control sera but observed at a very low intensity. Other bands observed both in the control and challenge sera were considered as challenge-non-specific bands (Supplementary Tables S1, S2).

### Mass spectrometry analysis and protein identification

Silver stained bands corresponding to the reactive bands detected in the immunoblots were excised and digested using an automatic device (DigestPro MS, Intavis, Cologne, Germany). The process involved reduction with dithiothreitol, derivatization with iodoacetamide, and enzymatic digestion with trypsin (37°C, 8 h) (Casanovas et al., 2009). The tryptic digests were evaporated and redissolved in 5 μl of methanol/water/trifluoroacetic acid (30/70/0.1 v/v).

Proteins in the tryptic digests (0.5 μl) were identified by MALDI-TOF peptide mass fingerprinting combined with MS/MS ion search in a 4800 TOF/TOF mass spectrometer (ABSciex, Barcelona, Spain) in the reflectron mode. The spectra were externally mass calibrated using a standard peptide mixture. Alpha-cyano-4-hydroxycinnamic acid (3 mg/ml) was used as the matrix. The five signals with the greatest intensity in each MALDI-TOF spectrum were automatically analyzed by TOF/TOF. The combined TOF and TOF/TOF spectra were interpreted by database search (Mascot, Matrix Science, MA, USA) using the following parameters: peptide mass tolerance, 50 ppm; fragment mass tolerance, 0.5 Da; fixed modification, carbamidomethyl cysteine; variable modification, oxidation of methionine; significance threshold of the MOWSE score, *p* < 0.05. All identifications were manually validated.

Samples which did not produce a positive identification by MALDI were reanalysed by LC-MS/MS in a Velos-LTQ or an Orbitrap-XL mass spectrometer (Thermo Fisher Scientific) equipped with a microESI ion source. Four microliters of each sample digest were diluted to 20 μl with 5% methanol and 1% formic acid, and loaded into a chromatographic system consisting of a C18 preconcentration cartridge (Agilent Technologies) connected to a 15-cm long, 150 μm i.d. (Velos-LTQ) or 100 μm i.d. (Orbitrap-XL) C18 column (Nikkyo Technos Co.). The separation was performed at 1 μl/min (Velos-LTQ) or 0.4 μl/min (Orbitrap XL) in a 30-min gradient from 3 to 40% acetonitrile (solvent A: 0.1% formic acid, solvent B: acetonitrile 0.1% formic acid). The instruments were operated in the positive ion mode with a spray voltage of 1.5 kV. The spectrometric analysis was performed in a data dependent mode. The scan range for full scans was m/z 400–1,800. The LC-MS/MS spectra were searched using SEQUEST (Proteome Discoverer v1.4, Thermo–Fisher Scientific) with the following parameters: peptide mass tolerance, 1 Da (Velos-LTQ) or 20 ppm (Orbitrap-XL); fragment tolerance, 0.6 Da; enzyme, trypsin; two missed cleavages allowed; dynamic modification, methionine oxidation (+16 Da); fixed modification, cysteine carbamidomethylation (+57 Da). The peptide identifications were filtered at 0.1% FDR and only proteins identified with two or more peptides and peptide rank 1 were considered. Relative abundance of the identified proteins in each sample was roughly estimated from the product of the total peptide sequence matches pointing to that protein and its sequence coverage. The group of more abundant proteins bearing more than 80% of the total abundance in the sample were considered for discussion (Full data is available in Supplementary Tables S3–S9).

Searches for the MALDI and LC-MS/MS methods described above were carried out against the Uniprot database (2015_11 version) restricted to *Brachyspira*. When results pointed to indistinguishable different accessions to the same protein in different strains, the accession for the reference ATCC strains was reported in Table 1, Supplementary Tables S1, S2.

**Table 1:** **Proteins identified in the immunoreactive bands from the two ***Brachyspira*** species**.

**Band id^a^**	**Sample**	**Specific^b^**	**Observed Mass (kDa)**		**Protein identified**		
			**12%**	**7.5%**	**Accession^c^**	**Name**	**Theor. Mass (kDa)**
2	OLA9	X	86	76	D8ICZ7	Elongation factor G	75.8
3	OLA9		83		D8IDC2	Uncharacterized protein	62
4	ATCCBP	X	81	78	D8ICZ7	Elongation factor G	75.8
5^*^	OLA9	X	78	72	J9UT37	Putative polymerase	72.3
					D8IEM8/A0A0G4K4U5	Chaperone protein HtpG	73.9
					D8IBS0	Chaperone protein DnaK	67.6
6	ATCCBP		77		D8IDC2	Uncharacterized protein	62
7	OLA9		75		D8IDC2	Uncharacterized protein	62.1
9	V1		73		G0EJY7	Uncharacterized protein	65.4
10^*^	ATCCBP	X	72	70	D8IEM8	Chaperone protein HtpG	73.9
					J9UT37	Putative polymerase	72.3
					D8ICG5	Uncharacterized protein	85
12	V1		72		C0R0R7	Putative treponemal membrane protein	63.9
13	OLA9	X	72	68	D8IE58	Aspartyl-tRNA synthase	67.5
15	ATCCBP		69		D8IDC2	Uncharacterized protein	62.1
16	ATCCBP	X	69		D8IFS5	Phosphoenol pyruvate carboxykinase	67.5
17	ATCCBP	X	69	70	D8IE58	Aspartyl-tRNA synthase	67.5
18	V1	X	68		C0R0E5	Phosphoenol pyruvate carboxykinase	67.5
19	OLA9	X	65		D8I9T6	Biotin lipoyl	65
20	OLA9		64	61	D8IB78	60 kDa chaperonin	58.1
22	ATCCBP	X	63		D8I9T6	Biotin lipoyl	65.0
24	ATCCBP		62	61	D8IB78	60 kDa chaperonin	58.1
25	V1		61		C0QWH4	60 kDa chaperonin	58.2
26^*^	OLA9	X	58	53	J9USS2	60 kDa chaperonin	58.1
					D8IAM3	Amidohydrolase 3	60.6
					J9UU81	2-isopropylmalate synthase	54.4
					D8IF78	Outer membrane efflux protein	54.7
					D8IDP7	Trigger factor, C-terminal domain protein	50.6
27	OLA9	X	57		D8IBK7	TmpB outer membrane protein	42.5
28	ATCCBP	X	56	55	K0JIZ9	Carboxyl terminal protease	54.9
29^*^	ATCCBP	X	54		D8IBK7	TmpB outer membrane protein	42.4
31	ATCCBP	X	51	50	D8IBH9	ATP synthase subunit beta	45.6
32	ATCCBP	X^*^	50		D8IET2	Enolase	47
36	V1	X	45		C0QW84	Uncharacterized protein	38.6
37	OLA9		44		Q9FA06	Putative elongation factor Tu	16.3
					D8ICZ6	Elongation factor Tu	44.8
38	ATCCBP		44		Q9FA06	Putative elongation factor Tu	16.3
					D8ICZ6	Elongation factor Tu	44.8
39	ATCCBP	X	44		D8ICG3	Uncharacterized protein	38.9
40	OLA9	X	43	42	D8IBY6	FlaA	35.7
41	V1		43		C0QVZ4	Elongation factor Tu	44.7
					Q9FA06	Putative elongation factor Tu	16.3
42	V1		42		C0QXS8	NADH oxidase	50.5
					C0QVZ4	Elongation factor Tu	44.7
43	V1		42		C0R0T5	FlaA	24.5
					P32520	FlaA1	36
44	ATCCBP	X	41		D8IBY6	FlaA	35.7
45	OLA9	X	40	38	D8ICU0	VspD	42.7
46^*^	ATCCBP	X	38	38	D8ICU0	VspD	42.7
					J9TU32	Ribonucleotide-diphosphate reductase subunit beta	41.1
					D8IEW7	Mannose-1-phosphate guanylyltransferase	40.5
					J9URY6	2-oxoacid:ferredoxin oxidoreductase subunit alpha-like protein	82.8
					J9UBH8	ATP-dependent 6-phosphofructokinase	34.8
					D8ICA2	Toxic anion resistance family protein	40.9
					D8I9T4	Uncharacterized protein	39.9
					D8ICR1	Pyruvate oxireductase	35.5
47	OLA9		36		D8IAP2	Flagellin FlaB2	32
48	V1	X	35		C0R1L9	UDP-glucose 4 epimerase	36
					C0QYC2	Galactose-glucose binding protein	38.2
49	ATCCBP		35		D8IAP2	Flagellin FlaB2	32
50	V1		35		Q26501	FlaB1	32
51^*^	V1	X	35		A0A0H0W3D6	Enolase	47
					Q26501	Flagellar protein FlaB1	32
					A0A0HOUMF4/G0EJK5	Fructose-bisphosphate aldolase	35.4/35.4
					C0QYC2	Methyl-galactoside ABC transporter substrate-binding protein	38.2
					C0QZV6	Pseudouridine-5'-phosphate glycosidase	33.9
52	OLA9	X^*^	33		D8ICA8	Putative FlaA	26.7
53	OLA9		32		K0JHQ4	Flagellin FlaB2	31.3
54	V1		31		P80160	Flagellin FlaB2	31.1
55	ATCCBP		31		K0JHQ4	Flagellin FlaB2	31.3
56	OLA9		29		K0JLS4	Flagellin FlaB3	30
57	V1	X^*^	29		C0QV52	Enolase	47
					Q9F0F6	Flagellin FlaB3	30.4
58	ATCCBP		29		D8IDG1	Flagellin FlaB3	26.6
59	V1		29		Q9F0F6	Flagellin FlaB3	30.4
61	V1	X	26		C0QWY9	Putative FlaAL	24.7
62	ATCCBP	X	24		D8ICA7	FlaA-2	24

*Complete identification data is available in Supplementary Table S1 (MALDI TOF/TOF) and Supplementary Tables S3–S9 (LC-MS/MS). Specific, indicates immunoreactivity is only detected in challenged animals. Observed mass, correspond to the experimental mass calculated from the gel bands. ATCCBP, ATCC 51139 (P43/6/78) B. pilosicoli strain*.

a *All identifications by MALDI TOF/TOF except those indicated with an asterisk which were identified by LC-MS/MS*.

b Specific bands with X^*^ have been detected in a control serum with an intensity of one.

c *Group head accession, full data in Supplementary Tables S1, S2*.

### Mass spectrometry target analysis of Vsp proteins

LC-MS/MS analyses of Vsps were carried out in a Velos-LTQ using the configuration described above for protein identification. The equivalent to 1 μg of protein in 20 μl of 5% methanol, 1% formic acid was loaded into a system and a 120-min acetonitrile gradient from 0 to 40% was used. The spectrometric analysis was performed in the target mode, acquiring the MS/MS scans of the signals included in the corresponding mass list (Supplementary Table S10). The mass list for each species included at least two unique peptides for each Vsp protein. Potential peptide targets detected in previous shotgun analyses were preferentially selected to build the mass lists. Other targets were selected among the best proteotypic peptides predicted by the PeptideRank software (Qeli et al., 2014).

## Results

### Protein fractionation of *B. pilosicoli* and *B. hyodysenteriae* strains and selection of immunogenic fractions

Proteins from the cell lysates were fractionated by preparative isoelectric focusing (IEF) prior to SDS-PAGE. Via this technique, we could fractionate up to 1 mg of each *Brachyspira* lysate using 24-cm pH 3-10 Immobiline Drystrips. The Off-gel system provided adequate resolution and reproducibility for protein fractionation as verified in a parallel study in which replicate SDS-PAGE analyses were carried out on an arbitrary selection of 7 out of the 24 fractions collected (Supplementary Figure S1). This reproducibility allowed the pooling of fractions from the three independent IEF fractionations, thereby providing of the equivalent of a total of 3 mg of fractionated proteins for each *Brachyspira* strain.

**Figure 1:**
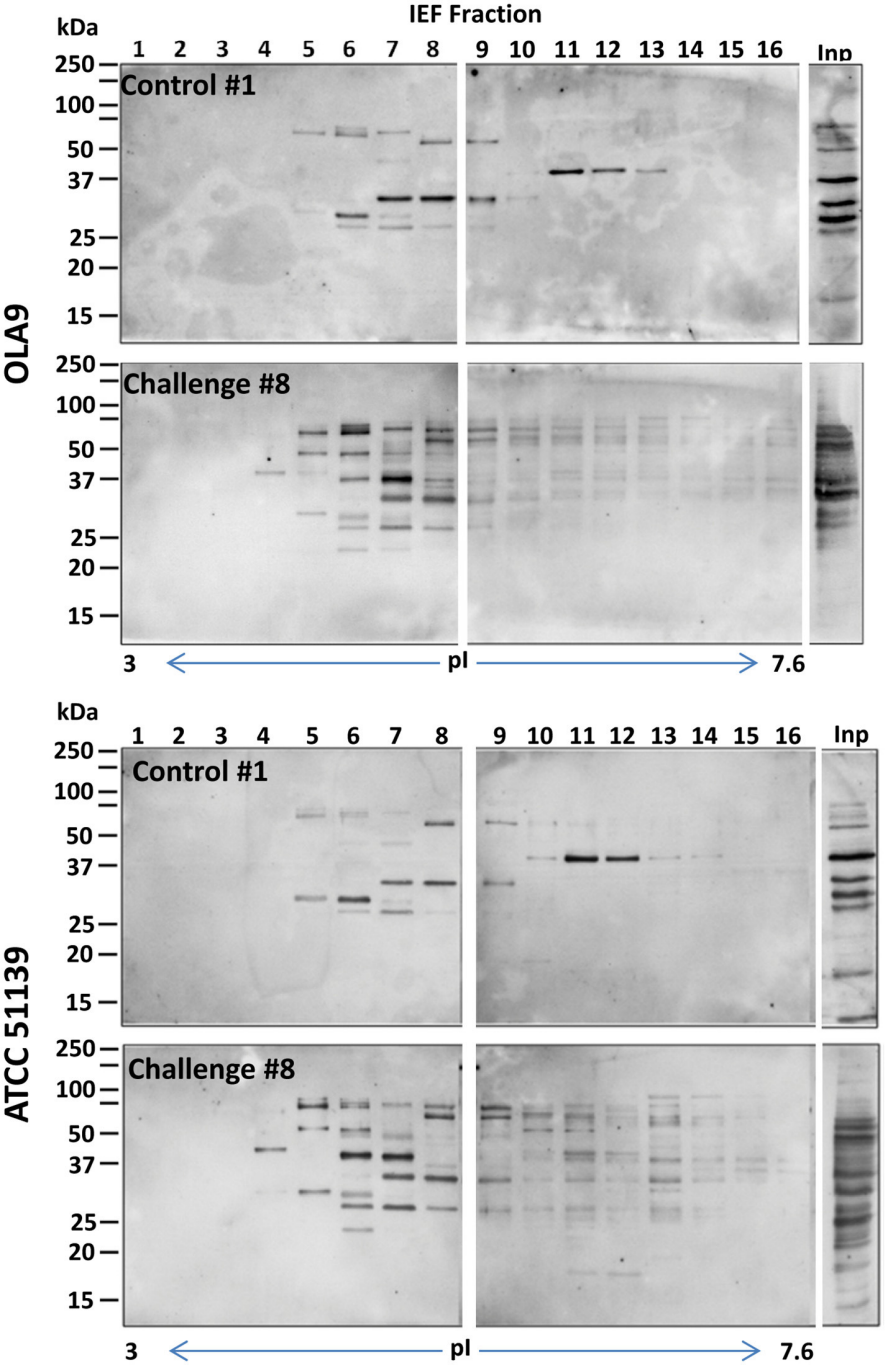
**General view of the immunoreactive proteins in each of the IEF fractions of the protein extracts of OLA9 (top) and ATCC 51139 (bottom) ***B. pilosicoli*** strains**. The images are examples of the Western blots prepared with sera #1 and #8 (from a control and a challenged animal, respectively) (upper and lower gels for each strain). Twenty four consecutive IEF protein fractions, covering a pI range from 3 to 10, were analyzed in the corresponding lanes of three SDS-PAGE gels. Fractions presenting intense immunoreactive bands in these preliminary experiments were submitted to a detailed immunoproteomics analysis using all the individual sera available. The Figure shows only the images for the two first gels (lanes for IEF fractions 1–16), containing the more acidic fractions, and the lane corresponding to the crude proteome extract (lane Inp). IEF fractions 17–24 did not show relevant immunoreactive bands (complete images for all the fractions and individual sera tested can be found in Supplementary Figures S2A–C).

**Figure 2:**
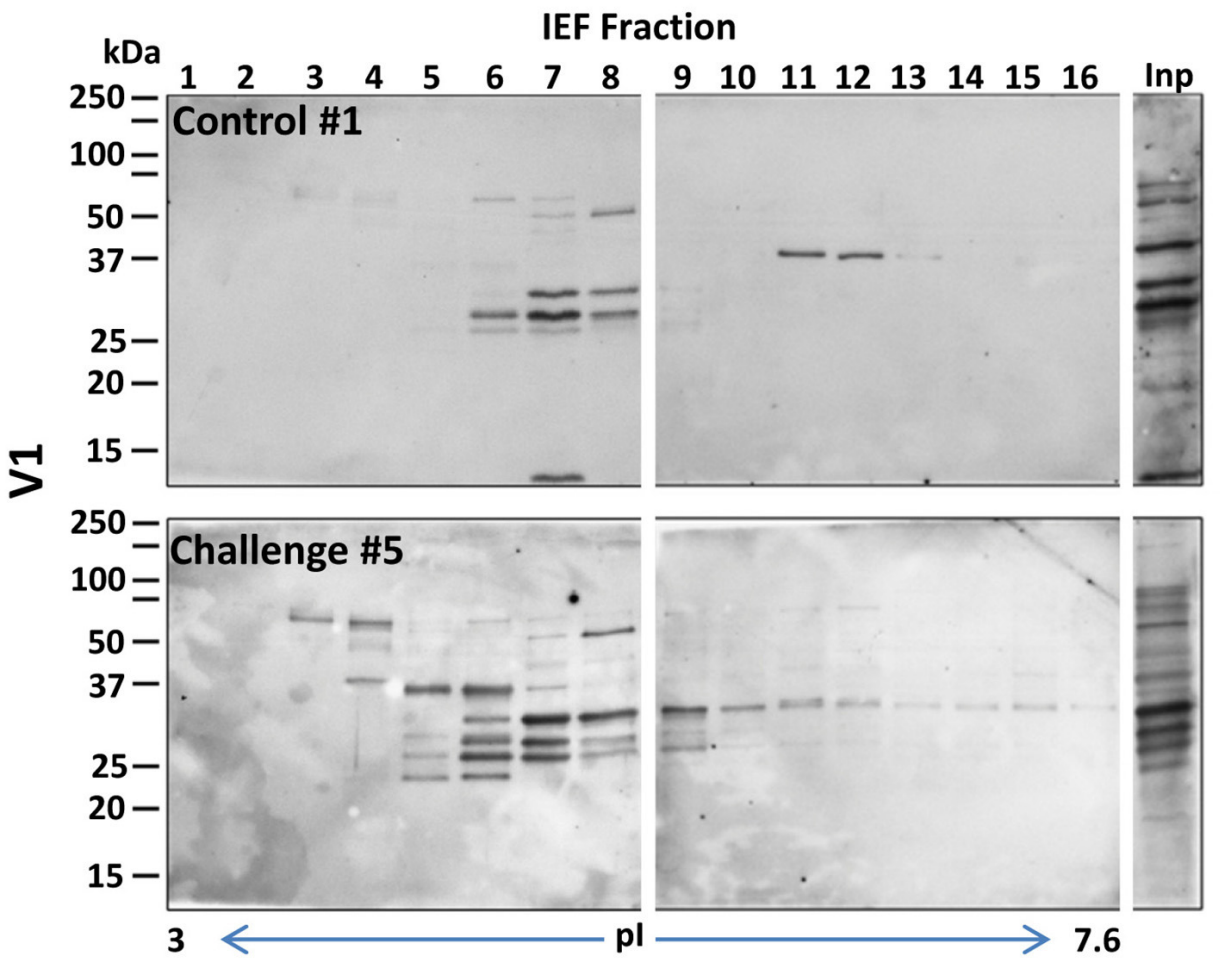
**General view of the immunoreactive proteins in each of the IEF fractions of the protein extracts of the ***B. hyodysenteriae*** strain V1**. The images correspond to examples of the Western blots prepared with sera #1 and #5 (a control and a challenged animal, respectively) (upper and lower gels for each strain). Fractions presenting intense immunoreactive bands in these preliminary experiments were submitted to a detailed immunoproteomics analysis, see Figure 1 for details. Inp, lane corresponding to the crude extract before IEF separation.

To select the fractions that contained the immunogenic proteins, the 24 recovered fractions were immunoblotted with sera from pigs challenged with *B. pilosicoli* (serum 6–10, for OLA9 and ATCC 51139 fractionations) or *B. hyodysenteriae* (serum 1–5 for V1 fractionation). Most of the immunogenic bands from the three *Brachyspira* strains appeared in the early-middle fractions (numbers 3–13) that had isoelectric points between 3.5 and 6.5 (Figures 1, 2 and Supplementary Figure S2). Note that for all strains, an immunoblot analysis with control serum from non-challenged pigs also revealed some reactive bands (Supplementary Figure S3). This cross-reactivity was consistent with previous reports that showed that healthy pig serum detects *B. hyodysenteriae* surface antigens (Wannemuehler et al., 1988) or some of the recombinant proteins tested for a vaccine against SD (Song et al., 2009).

### Protein identification

**Figure 3:**
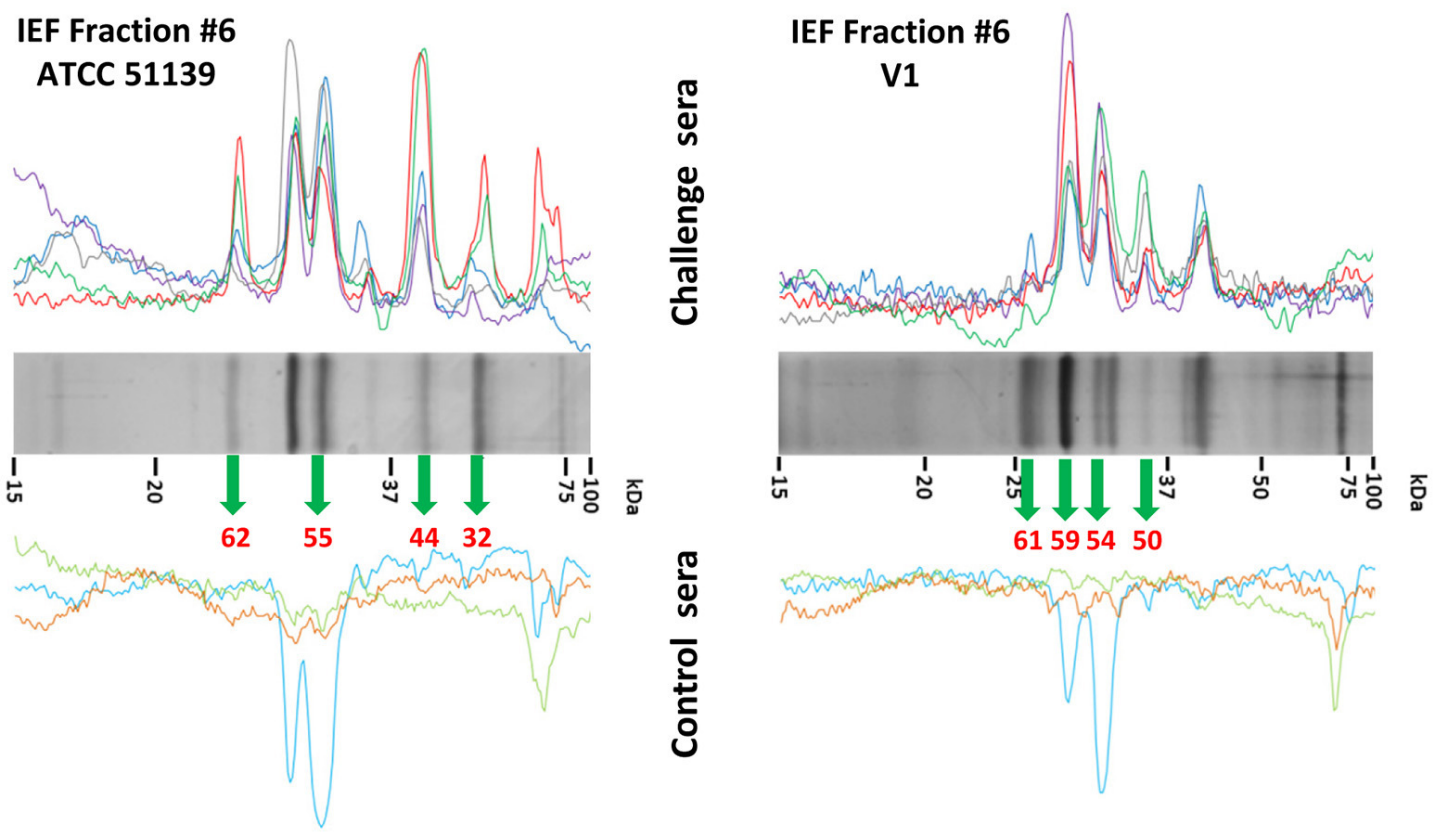
**Identification of immunoreactive proteins in the IEF fractions of the ***Brachyspira*** proteomes**. The image shows the Western blot densitometry profiles **(top and bottom)** and the protein band profile of the corresponding silver-stained gel lane (center). Immunoreactivity traces for the 5 sera from challenged pigs **(top)** and the 3 sera for control pigs **(bottom)** are shown with different colors (see SI for color codes). Bands identified as immunoreactive were sliced from the SDS gel lane and submitted to MS analyses for identification. Code numbers for the bands analyzed from these specific lanes are indicated in red. The example given corresponds to the IEF fractions #6 from ATCC 51139 (B. *pilosicoli*) (left) and V1 (*B. hyodysenteriae*) (right) strains. The full set of images for all the fractions is given in Supplementary Figures S5A–H.

**Figure 4:**
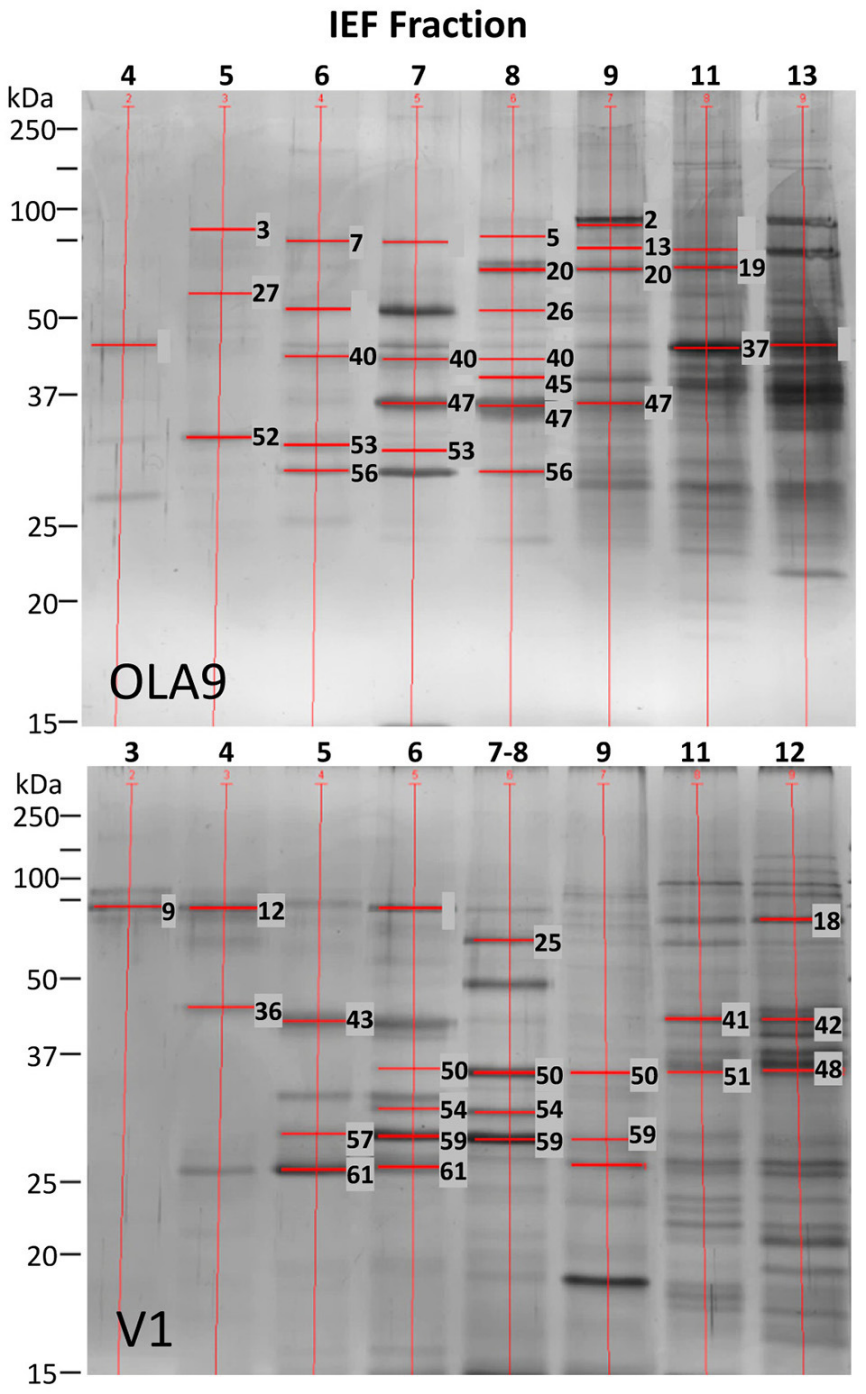
**Summary of the analyzed bands from SDS-PAGE silver stained gels for the selected IEF fractions of OLA9 strain (***B. pilosicoli***) and V1 strain (***B. hyodysenteriae***)**.

IEF fractions from the different *Brachyspira* strains showing the highest immunogenic response (fractions 4–9, 11, 13 and 3–9, 11–12, for *B pilosicoli* and *hyodysenteriae* respectively) (Figures 1, 2) were selected for a more detailed image analysis and band characterization. For this purpose, these fractions were reanalysed by SDS-PAGE and immunoblotted. Immunoreactive bands were identified by densitometry and the immunoblot images were matched with those obtained by silver staining on replicate SDS-PAGE separations (Figures 3, 4). The bands were then excised, trypsin digested and analyzed by mass spectrometry. Overall the components in 51 gel bands (36 from *B. pilosicoli* and 15 from *B. hyodysenteriae* strains) were identified (Table 1, Supplementary Tables S1–S9).

Most of the bands (45) could be identified by MALDI, except for six of them that required an LC-MS/MS analysis. The failure of MALDI in the analysis of these bands was probably due to the presence of several major proteins in the band as confirmed from the LC-MS/MS identification data. Thus, when LC-MS/MS identifications were filtered to select the most abundant components, only one of these bands produced a single protein while the others showed the presence of 3–8 major components.

### *B. pilosicoli* immunoreactive proteins

**Figure 5:**
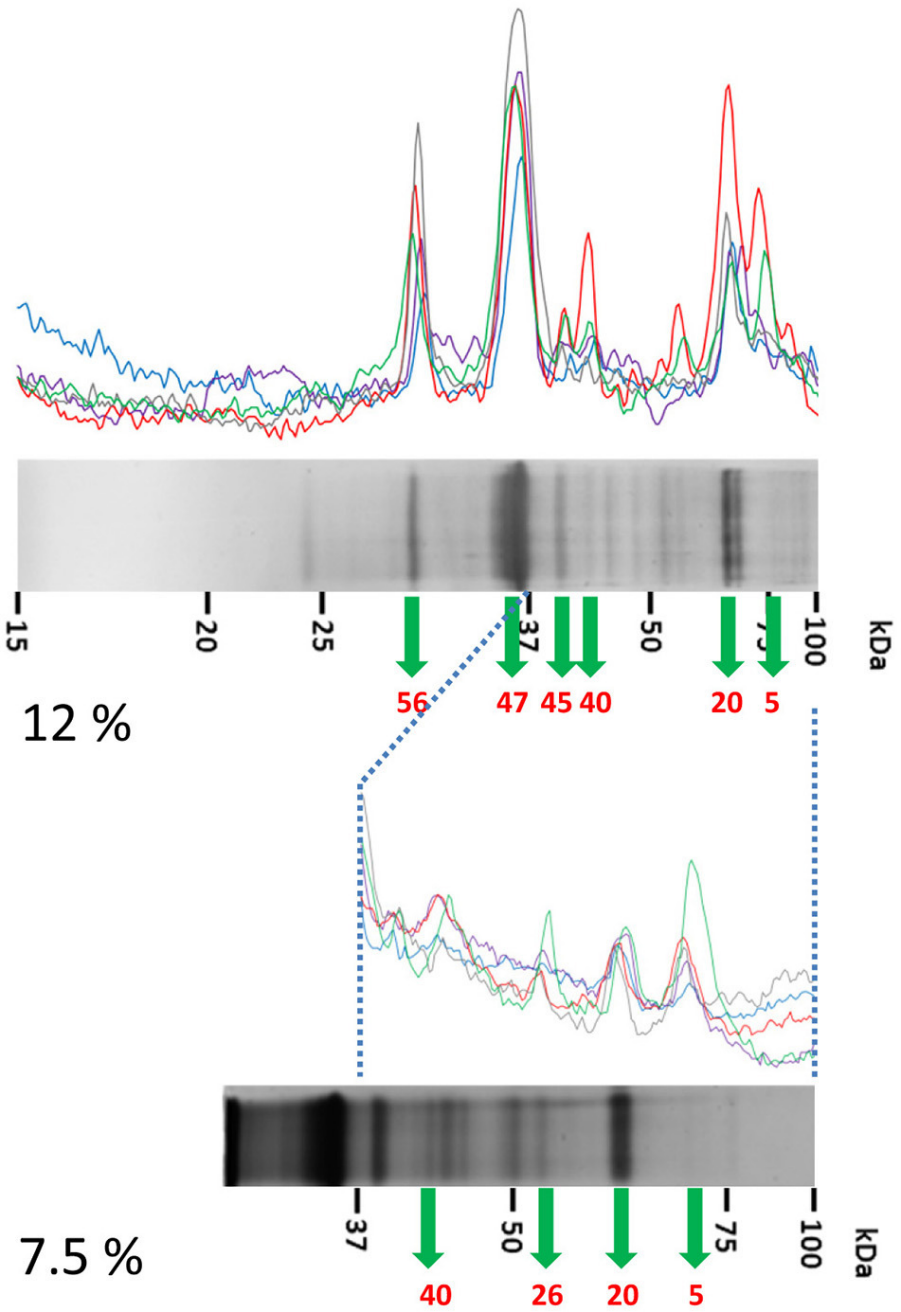
**Detailed analysis of the high-mass proteins in IEF fractions #8 and #9 from ATCC 51139**. IEF fractions #8 and #9 from B. *pilosicoli* showed unresolved, complex profiles of bands in 12% acrylamide gels (up). These profiles were resolved by a parallel analysis of these fractions in 7.5% acrylamide gels (down). Data from the 12% and 7.5% separations were later combined. The example shown corresponds to IEF fraction #8 (see Figure 3 for details).

Sixteen and 20 immunoreactive bands were detected for the *B. pilosicoli* OLA9 and ATCC 51139 strains, respectively (Supplementary Figures S4, S5A–F and Table 1, Supplementary Tables S1, S2). Several of these bands were common to both strains. Some of the fractions (#8 and #9) showed a complex profile at the high mass range of the silver-stained gels. To increase the resolution of the more complex fractions, parallel SDS-PAGE and Western blot analyses were carried out using 7.5% acrylamide gels (Figure 5, Supplementary Figures S5B,E). The mass spectrometric analysis of the corresponding silver-stained SDS-PAGE bands (Figures 3, 4) produced the identification of 28 different proteins in challenge-specific immunoreactive bands (Table 1). Eighteen of the immunoreactive specific bands yield a single protein (7 for OLA9 and 11 for ATCC 51139), while 4 other bands were shown to contain more than one protein.

The two *B. pilosicoli* strains shared eight proteins in common in the bands with specific reactivity toward the challenge sera (the outer membrane protein of the TmpB family, a flagellar filament outer layer protein FlaA, the variable surface protein VspD, the chaperone protein htpG, a putative polymerase, the aspartyl-tRNA synthase, the biotin lipoyl and the elongation factor G) (Table 1).

Seven other proteins common to both strains were found in challenge-non-specific bands including a 60 kDa chaperonin, flagellins B2 and B3 and the elongation factor Tu.

### *B. hyodysenteriae* immunoreactive proteins

Fifteen immunoreactive bands were detected in the selected IEF fractions from the *B. hyodysenteriae* farm isolate V1 using sera from *B. hyodysenteriae*-challenged animals (Figures 3, 4, Supplementary Figures S4, S5G,H). Six of these bands showed challenge specific immunoreactivity (#18, #36, #48, #51, #57, #61) while nine others also cross-reacted with control sera from healthy pigs (#9, #12, #25, #41, #42, #43, #50, #54, and #59). Three of the challenge-specific bands, produced a single protein identification, while the other bands were shown to contain several proteins up to a total of 11 (Table 1, Supplementary Tables S1–S9). Among the challenge-specific proteins PEPCK (phosphoenolpyruvate carboxykinase) and enolase had also been identified as antigenic in the *B. pilosicoli* isolates.

### Cross-reactivity with control sera

For all strains, the immunoblots with sera from control, non-challenged pigs also revealed several immunoreactive bands (Supplementary Figure S3).

Cross-reactivity was observed for all flagellar proteins except *B. pilosicoli* FlaA. The FlaB proteins were the primary targets of the control sera for *B. pilosicoli* and *B. hyodysenteriae* (Table 1, Supplementary Tables S1, S2). The three isoforms of FlaB that constitute the inner core of the spirochaetal flagella (FlaB1, FlaB2, and FlaB3) were identified in the immunoreactive bands (Table 1, Figure 4).

Other proteins immunoreactive to control sera were Elongation factor Tu, which was detected in both species and the uncharacterized protein D8IDC2, which was identified in the two B. *pilosicoli* strains.

### Distribution of Vsp among *Brachyspira* species and strains

Although the variable surface protein VspD has been suggested as a potential vaccine candidate against *B. hyodysenteriae* we could only identify VspD in the immunoreactive bands from *B. pilosicoli* samples (Table 2, Supplementary Tables S11–S17). To confirm these findings, a more detailed LC-MS/MS analysis was performed targeting the family of Vsp proteins. For this, each Vsp protein was monitored by targeting two or more tryptic peptides with sequences unique to that specific protein (Supplementary Table S10). Three strains of *B. hyodysenteriae* (two commercial ATCC strains and one isolate) and two strains of *B. pilosicoli* (one commercial ATCC strain and one isolate) were analyzed.

**Table 2:** **Presence of Vsp proteins in *Brachyspira* strains**.

	***B. hyodysenteriae***			***B. pilosicoli***	
	**WA1**	**B-78**	**INF1**	**ATCCBP**	**OLA9**
**Protein**	**Collection**	**Collection**	**Isolate**	**Collection**	**Isolate**
VspA	nd	nd	nd	na	na
VspB	^*^^a^	^*^^a^	^*^^a^	na	na
VspC	^**^	^**^	nd	na	na
VspD	^*^	nd	nd	^***^	^****^
VspE	^*^	^**^	^**^	^**^	^*^
VspF	^****^	nd	nd	na	na
VspG	nd	nd	nd	na	na
VspH	nd	^***^^a^	nd	^**^	nd
VspI	?	?	^**^	na	na
VspJ	nd	nd	nd	na	na

Asterisks indicate total number of peptide sequence matches (PSM) for the protein (

* , 0–25 PSM;

** , 25–100 PSM;

*** , 100–300 PSM;

**** *, > 300 PSM)*.

*PSM is correlated with protein abundance. ATCCBP, ATCC 51139 (P43/6/78) B. pilosicoli strain*.

a *Identified from only one protein-exclusive peptide*.

*nd, not detected; na, not monitored (sequence not described for the species)*.

*?, Identified from only one, non-protein-exclusive peptide (common with VspA)*.

The analysis showed that the profile of Vsp proteins was very variable between species and strains (Table 2). In agreement with our previous results, VspD, was highly abundant in both B. *pilosicoli* strains (more than 200 PSMs per strain). Contrarily, it was detected with a very low abundance (<25 PSMs) (Table 2, Supplementary Tables S13, S17) in only one of the three *B. hyodysenteriae* strains (WA1). Another proposed vaccine candidate in this family, VspH, was found in both species but not in all strains.

The vspC and vspF genes have not been described in B. *pilosicoli*. Both proteins were detected in *B. hyodysenteriae* although the expression was strain-dependent. VspC was detected in the commercial strains (range 25–100 PSMs), but not in the isolate. To the best of our knowledge, this result constitutes the first experimental evidence at the protein level of the expression of this protein. On the other hand, VspF was found to be greatly abundant in the *B. hyodysenteriae* WA1 strain but was not detected in the others. This is similar to reports by Black et al. (2015), who described the absence of the vspF gen in some strains from *B. hyodysenteriae*. According to Witchell et al. (2011), VspF and VspE are found in the cell in a protein complex which can include also other Vsps. Differently to the other members discussed above, VspE was detected in both species and in all strains.

We could not identify VspA, VspG, or VspJ in any of the strains analyzed. Another member, VspB, described from the genome of *B. hyodysenteriae* was detected in all strains but with a very low abundance.

## Discussion

### Challenge-specific immunoreactive proteins common to both *Brachyspira* species

Two proteins (PEPCK and enolase) were revealed by challenged sera from both *Brachyspira* species. These proteins showed a high degree of identity between species (96% identity for *B. hyodysenteriae* and *B. pilosicoli* enolases and 95% of identity for the corresponding PEPCK).

In a vaccine search, candidates are often selected from membrane-exposed proteins, because surface exposure facilitates recognition by an antibody (Boyle et al., 1997). Still, many cytosolic proteins have been described as major antigens and some of them have also been explored as vaccine components (Davis et al., 2005). In our study, many of the proteins detected were proteins annotated as cytoplasmic. This is the case for PEPCK, whose antigenic capacity had been previously reported in other species. PEPCK was identified as the antigen triggering the cellular response responsible for the hepatic granulomatous inflammation in schistosomiasis (Asahi et al., 2000). Additionally, PEPCK from *Mycobacterium tuberculosis* has been demonstrated to induce a strong immune response in mice and, for this reason, was proposed as a component of a subunit vaccine for tuberculosis (Liu et al., 2006).

Enolase has been reported to be immunoreactive in several pathogenic species, such as *Mycobacterium tuberculosis* (Rahi et al., 2017), and *Borrelia burgdorferi* (Barbour et al., 2008). This protein has also been described as a differential, immunogenic protein in strains of *Bifidobacterium longum* ssp. longum (Górska et al., 2016) and showed protective activity against colitis in mice (Srutkova et al., 2015). Enolase is a moonlighting enzyme found on the surface of some pathogens and involved in the activation of plasminogen (Rahi et al., 2017). In a previous work (Casas et al., 2016), we reported enolase to be among the ten most abundant proteins detected in the surfaceome and exoproteome compartments for *B. pilosicoli* and *B. hyodysenteriae*.

These two proteins are thus tentative candidates for vaccines against *Brachyspira* infections that have not been included in earlier reported vaccines.

Two other putative uncharacterized proteins (C0QW84 and D8ICG3) were identified in challenge-specific bands (bands #36 and #39, respectively) (Figure 4) from *B. hyodysenteriae* and *B. pilosicoli*, respectively. Both are predicted (PSORTb v3.0 (Yu et al., 2010), SignalP v4.1 (Petersen et al., 2011) to be extracellular or located on the outer membrane, although C0QW84 lacks the signal peptide on the N-terminal side. These proteins have the same molecular weight and isoelectric point and 62% of identity and 72% similarity between them, suggesting they are different forms of the same functional molecule in these species. In this case, a potential vaccine candidate common to both species would require identifying possible common epitopes capable of inducing an immune response.

### *B. pilosicoli* challenge-specific immunoreactive proteins

Twenty-eight *B. pilosicoli* proteins were identified in the challenge-specific bands, with 8 of them common to the ATCC 51139 and environmental OLA9 strains. More than half of these proteins could be identified as proteins potentially exposed on the surface of the bacteria or secreted into the media. Thus, 9 corresponded to known or predicted (Gene Ontology Annotation (GOA), PSORTb) membrane or membrane-exposed proteins (TmpB outer membrane protein, outer membrane efflux protein, VspD, enolase, D8ICG5, D8ICG3) or flagellar proteins (FlaA, FlaA2, D8ICA8) (Supplementary Table S2). Six of these proteins (ATP-dependent 6-phosphofructokinase, 2-isopropyl malate synthase, chaperone protein DnaK, pyruvate oxidoreductase, enolase and VspD) were identified in a previous work among the most abundant proteins in the exposed proteome of these bacteria (Casas et al., 2016). Especially VspD, similar to enolase, was among the 10 most abundant proteins exclusively found on the exoproteome (i.e., proteins present in the bacterial culture media). Variable surface proteins constitute a well-known family of antigenic bacterial components. However, no evidence at the protein level had been previously reported for the expression of any Vsp protein in *B. pilosicoli*.

The treponemal outer membrane protein B (TmpB, D8IBK7) was identified as challenge-specific for both *B. pilosicoli* strains. Two putative treponemal membrane proteins (C0R0R7 and C4MGG7) with 47 and 55% homology with *B. pilosicoli* TmpB were included in a reverse vaccinology study against *B. hyodysenteriae*, but they were not immunoreactive when immunoblotted with porcine sera from challenged animals (Song et al., 2009). Contrarily, we detected C0R0R7 in immunoreactive bands of *B. hyodysenteriae*. However, this reactivity was observed toward both the challenge and control sera, so this protein was discarded as a specific vaccine candidate.

The flagellar protein FlaA was also found in the immunoreactive bands of both *B. pilosicoli* strains. FlaA constitutes the outer sheath of periplasmic flagella in spirochetes, impacting the unusual morphology and motility of this bacterial phylum (Li et al., 2000, 2008; Rosa et al., 2005; Jiang et al., 2014; Zhao et al., 2014). Flagellar proteins are major immunoreactive proteins in *B. hyodysenteriae* (Kent et al., 1989). It has been reported that FlaA from *S. hyodysenteriae, S. innocens*, and *S. pilosicoli* was recognized by rabbit polyclonal and murine monoclonal antibodies produced against *S. hyodysenteriae* lysates (Fisher et al., 1997). However, no data were previously available on the antigenicity of *B. pilosicoli* FlaA. In our study, both *B. pilosicoli* and *B. hyodysenteriae* FlaA were found in immunoreactive bands although only *B. pilosicoli* evidenced a challenge-specific response.

Two other cytoplasmic proteins, the chaperone protein HtpG and aspartyl tRNA synthase, were found to be immunogenic in both *B. pilosicoli* strains. HtpG has been reported to be responsible for a strong humoural response in human periodontitis caused by *Porphyromonas gingivalis* (Shelburne et al., 2008). On the other hand, aminoacyl tRNA synthases are the targets of many antibacterial compounds (Chopra and Reader, 2014). They play an important role in bacterial resistance as described for *Mycobacterium tuberculosis* strains where mutations in this protein are involved in their resistance to whole-cell inhibitors (Ioerger et al., 2013). No data were available on the antigenicity of these molecules.

### *B. hyodysenteriae* challenge-specific immunoreactive proteins

Flagellar proteins were the most frequent class of immunoreactive proteins identified in *B. hyodysenteriae* (Table 1, Supplementary Tables S1, S2). However, except for a putative flagellar filament outer layer-like protein (C0QWY9), other flagellar proteins identified such as FlaA1, FlaB1, and FlaB3 were also found in bands immunoreactive toward control sera. FlaA1 had been previously described as one of the molecules that produced a highly specific immune reaction in *B. hyodysenteriae* (Li et al., 1993).

Other proteins detected in the challenge-specific immunoreactive bands included the galactose-glucose binding protein, a periplasmic protein, and two cytoplasmic proteins, pyridine nucleotide-disulfide oxidoreductase and fructose-bisphosphate aldolase. These proteins have been detected in the extracellular space in other species involved in processes related to the interaction/ adhesion to the host cell (Tunio et al., 2010; Roier et al., 2015; Zhe et al., 2016). The galactose-glucose binding protein was identified as the main component of the outer membrane vesicles released from 5 different strains of *Haemophilus influenza* (Roier et al., 2015). Extracellular nanovesicles are released by all pathogenic and non-pathogenic gram-negative bacteria (Lusta, 2015). They are composed of outer membrane components such as LPS, glycerophospholipids as well as proteins from the outer membrane and the periplasm (Kuehn and Kesty, 2005; Bai et al., 2014; Lusta, 2015). Outer membrane vesicles are considered as potent virulence factors because they provide a means for the extracellular secretion of proteins and lipids that can interact with the host tissues. The pyridine nucleotide-disulfide oxidoreductase (Zhe et al., 2016) has been identified as one of the proteins that interacts with brain microvascular endothelial cells, which may contribute to invasion by *Streptococcus equi* ssp. zooepidemicus through the blood-brain barrier. Finally, fructose-bisphosphate aldolase has been reported to be immunogenic in *Candida albicans* (Calcedo et al., 2012) and *Madurella mycetomatis*, in which it has been proposed as a vaccine candidate (de Klerk et al., 2012).

Vsp proteins are the most abundant outer membrane proteins of *B. hyodysenteriae* (Gabe et al., 1998), in which they have been postulated to have an antigenic role either as protein complexes or as individual molecules (McCaman et al., 1999; Witchell et al., 2011). Two members of the Vsp family, VspH, and VspD, have been included as components of a potential vaccine against *B. hyodysenteriae* (Bellgard et al., 2015). However, little is known about the expression of these proteins in *Brachyspira* species. The expression of VspH was reported in a B204 strain of *B. hyodysenteriae* (Witchell et al., 2006), although in further studies they described the absence of the gene in other strains (ATCC WA1 and X576) (Witchell et al., 2011). In the latter study, the expression of the VspD protein (together with vspF, vspE, and vspI) in a virulent Australian isolate of *B. hyodysenteriae* (Witchell et al., 2011) was reported. These authors suggested that Vsp proteins form complexes and that they are immunoreactive only in that form.

Interestingly, the analysis of our immunoreactive bands identified VspD only in the *B. pilosicoli* samples. This was in agreement with our previous work (Casas et al., 2016) on the exposed proteomes of *B. pilosicoli* and *B. hyodysenteriae*. In that study, the VspD protein was classified as exclusive from the exoproteome of *B. pilosicoli* because it was not found in any compartment of *B. hyodysenteriae*. To confirm our findings about the differential presence of VspD and to depict the distribution of the Vsp proteins in different *Brachyspira* strains, a more detailed targeted LC-MS/MS analysis was performed. The study confirmed the high expression of VspD in both *B. pilosicoli* strains and the low or no expression in *B. hyodysenteriae* strains.

Thus, Vsp proteins have a broad expression profile in different strains and species, a trait that could determine the efficiency of proteins such as VspH or VspD as vaccine components. Vsp proteins are components of a mechanism used by pathogenic bacteria to adapt to host conditions and optimize colonization. These proteins can show reversible on/off switching of their expression (phase variation) or antigenic changes by the expression of alternative protein phenotypes (Lysnyansky et al., 2001). Variability in the expression of variable surface proteins has been reported for several species of *Mycoplasma*. In *in vitro* experiments with *Mycoplasma bovis*, it was observed that exposure of the bacteria to anti-Vsp antibodies induced a change in the Vsp expression pattern (Caswell et al., 2010). Vsp proteins are the major antigenic targets in *Mycoplasma bovis*, however the immune response observed was not protective. In *Mycoplasma mycoides*, the different Vsp expression pattern observed in several outbreaks was suggested to be related to different Vsp proteins triggering the immune response in each case (Hamsten et al., 2008). These facts stress the value of determining the actual gene product levels present under different conditions as a complement of genomic-based approaches for vaccine design.

### Cross-reactivity with control sera

We observed immunoreactive bands using control sera from non-challenged pigs with all strains tested (Supplementary Figure S3). This was consistent with previous reports showing that healthy pig serum can detect *B. hyodysenteriae* surface antigens (Wannemuehler et al., 1988). Song et al. also observed that non-vaccinated pigs from a herd with no reported history of SD developed increasing systemic lgG and lgM levels to all antigens of a vaccine being tested as the body weight of the animals increased (Song et al., 2009).

The FlaB family of proteins was the primary target of control sera in both species. This finding could be related with reports indicating that endoflagellar FlaBs, but not FlaA, from *B. hyodysenteriae* cross-react with the corresponding proteins from the non-pathogenic *B. innocens* (Li et al., 1993). In fact, it has been shown that the immunoreactivity patterns of purified flagellar proteins from different strains of *B. hyodysenteriae* and several non-pathogenic spirochetes have similar distributions, suggesting the existence of shared epitopes in these species (Kent et al., 1989). Thus, the observed cross-reactivity of flagellar proteins could be due to exposure to other non-pathogenic bacteria. Vaccination of pigs with the endoflagellar protein FlaB1 (recombinant or purified from *B. hyodysenteriae*) generated antibodies against the protein (Gabe et al., 1995). However, that response was not sufficient to protect the animals against the disease. It was suggested that the efficiency of the anti-FlaB antibodies would be reduced by the reduced accessibility to the inner part of the *B. hyodysenteriae* flagella (Gabe et al., 1995).

Other identified targets of the control sera were the Elongation factor Tu and the uncharacterized protein D8IDC2. Elongation factor Tu, is a protein which is conserved in different bacterial species so its detection with sera from pigs that had not been infected with SD or IS is not surprising. This protein has been detected on the surface of *Leptospira interrogans* (the spirochete which is the aetiological agent of leptospirosis) and it is related to the binding with the host plasminogen (Wolff et al., 2013). It is interesting to note that D8IDC2, which was identified in the two *B. pilosicoli* strains, has a 97% of homology with Bpmp-72, a membrane protein whose sequence was patented by Hampson and La (2009) for its use as a vaccine and a therapeutic treatment against intestinal spirochaetosis.

## Conclusions

The immunoproteomics approach applied in this study has been demonstrated to be very effective for the characterization of new *Brachyspira* antigens. Data reported here was restricted to IgG's immunoreactivity toward these molecules. Further studies focussing on serum and secretory IgA's could potentially increase this collection of potential candidates. Most previously reported vaccine candidates were selected on the basis of previous knowledge from other species and through *in silico* reverse vaccinology approaches. The advantage of an immunoproteomics approach is that it intrinsically takes into account the actual expression levels, molecular characteristics and exposure to the host of the specific antigens that elicit an immune response. Consequently, the immunoreactive proteins described are unrivaled candidates to be components of vaccines for the treatment of SD and porcine IS. As discussed, we identified two abundant antigenic proteins shared by the two *Brachyspira* species (enolase and PEPCK) that could be considered as candidates for common vaccines for these species. In addition, 8 and 11 challenge-specific immunoreactive proteins were described for *Brachyspira pilosicoli* and *B. hyodysenteriae*, respectively. Although some of the immunoreactive bands were shown to contain more than one component and the actual antigen should be confirmed, the collection of proteins described constitutes a unique antigen collection from these bacteria.

## Author contributions

JA, VC, AR, and MC conceived the project and designed the work. SV prepared the bacterial cultures and characterized the isolates by PCR. AR performed the immunoblot assays and RP the image analysis. VC collected proteomics data. VC and JA analyzed and interpreted the data. AR and VC redacted the manuscript draft and JA made the critical revision and produced the final manuscript. All the authors approved the final version of the article. VC carried out this work in the framework of the Immunology Ph.D. program of the Autonomous University of Barcelona.

## Funding

This work was funded by the Spanish MINECO (IPT-2011-0735-010000). The CSIC/UAB Proteomics Laboratory of IIBB-CSIC is a member of Proteored, PRB2-ISCIII and is supported by grant PT13/0001, of the PE I+D+i 2013-2016, funded by ISCIII and FEDER.

### Conflict of interest statement

The authors declare that the research was conducted in the absence of any commercial or financial relationships that could be construed as a potential conflict of interest.
