# Supplementary material for: Brachyspira hyodysenteriae and B. pilosicoli Proteins Recognized by Sera of Challenged Pigs
Source: Front Microbiol. 2017 May 4;8:723. doi: 10.3389/fmicb.2017.00723 (PMC5415613; doi:10.3389/fmicb.2017.00723)
Supplement: Supplementary file 1 [file Table1.PDF]

lane	strain	band number	Reference for MS data file
9	o	2	ppw_H4_145467558616.txt
5	o	3	ppw_G7_139601421306.txt
9	a	4	ppw_H12_145397049216.txt
8	*o*	5	no result, LC-MS/MS
5	a	6	ppw_G4_145631468011.txt
6	o	7	ppw_H1_145467558313.txt
3	v	9	ppw_H8_145467558920.txt
4	v	12	Peak List Id: 56492
9	o	13	ppw_F7_145631467706.txt
5	a	15	ppw_G3_145631468010.txt
13	a	16	ppw_G17_145397048304.txt
9	a	17	ppw_H7_145467558819.txt
12	v	18	ppw_H13_145467559325.txt
11	o	19	ppw_H10_139601422133.txt
9	o	20	ppw_F6_modified.txt
8	o	20	ppw_F4_145631467503.txt
11	a	22	ppw_G16_145397048203.txt
9	a	24	ppw_H10_145397049014.txt
7+8	v	25	ppw_I17_145397050633.txt
8	*o*	26	no result, LC-MS/MS
5	o	27	ppw_G6_139601421305.txt
9	a	28	ppw_H9_145397048913.txt
5	a	29	no result, LC-MS/MS
9	a	31	ppw_H7_145397048811.txt
7	a	32	ppw_G14_145397048001.txt
6	a	32	ppw_G8_145631468315.txt
4	v	36	ppw_H9_145467559021.txt
11	o	37	ppw_H9_139601422132.txt
11	a	38	ppw_G15_145397048102.txt
4	a	39	ppw_G1_145631467808.txt
6	o	40	ppw_G10_139601421409.txt
7	o	40	ppw_G18_139601421617.txt
8	o	40	ppw_F3_145631467402.txt
11	v	41	ppw_I22_145397051038.txt
12	v	42	Peak List Id: 56513
5	v	43	ppw_I6_145397049722.txt
6	a	44	ppw_G7_145631468214.txt
7	a	44	ppw_G13_145397048000.txt
8	o	45	ppw_F2_145631467401.txt
8	a	46	ppw_H6_145467558718.txt
9	*a*	46	no result, LC-MS/MS
7	o	47	ppw_G17_139601421616.txt
9	o	47	ppw_F5_145631467604.txt
8	o	47	ppw_F1_145631467300.txt
12	v	48	ppw_H12_145467559224.txt
7	a	49	ppw_G12_145631468519.txt
8	a	49	ppw_H5_145467558717.txt
6	v	50	ppw_I10_145397050026.txt
7+8	v	50	ppw_I15_145397050431.txt
9	v	50	ppw_I21_145397050937.txt
11	*v*	51	no result, LC-MS/MS
5	o	52	ppw_G4_139601421203.txt
6	o	53	ppw_G9_139601421308.txt
7	o	53	ppw_G16_139601421515.txt
6	v	54	ppw_I9_145397049925.txt
7+8	v	54	ppw_I14_145397050330.txt
6	a	55	ppw_G6_145631468213.txt
6	o	56	ppw_G8_139601421307.txt
8	o	56	ppw_G21_139601421720.txt
5	v	57	ppw_H11_145467559123.txt
7	a	58	ppw_G11_145631468418.txt
6	v	59	ppw_I8_145397049824.txt
7+8	v	59	ppw_I13_145397050229.txt
9	v	59	ppw_I20_145397050836.txt
6	v	61	ppw_I7_145397049723.txt
4	v	61	ppw_H10_145467559122.txt
6	a	62	ppw_G5_145631468112.txt

Mascot Search Results

User	:
Email	:
Search title	: F:\ROD_inmunoproteoma\MALDI\ppw_2016_561_562_563_565\ppw_H4_145467558616.txt
MS data file	: F:\ROD_inmunoproteoma\MALDI\ppw_2016_561_562_563_565\ppw_H4_145467558616.txt
Database	: Brachyspiraceae Brachyspiraceae_20160127-151122 (40573 sequences; 13470793 residues)
Timestamp	: 23 Feb 2016 at 16:36:52 GMT
Warning	: A Peptide summary report will usually give a much clearer picture of MS/MS search results.
Top Score	: 429 for G0ENS5, G0ENS5_BRAIP Elongation factor G OS=Brachyspira intermedia (strain ATCC 51140 / PWS/A) GN=fusA PE=3 SV=1

Probability Based Mowse Score

Protein score is -10*Log(P), where P is the probability that the observed match is a random event.
Protein scores greater than 59 are significant (p<0.05).
Protein scores are derived from ions scores as a non-probabilistic basis for ranking protein hits.

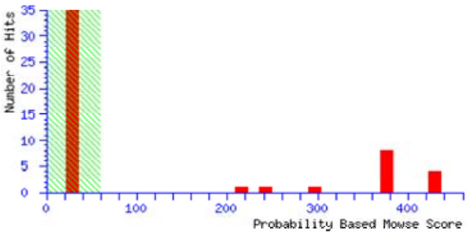

Protein Summary Report

Format As | Protein Summary (deprecated) | Help

Significance threshold p< 0.05 | Max. number of hits AUTO

Standard scoring ◉ MudPIT scoring ○ | Ions score or expect cut-off 0 | Show sub-sets 0

Show pop-ups ◉ Suppress pop-ups ○ | Sort unassigned Decreasing Score | Require bold red ☐

Re-Search All | Search Unmatched

Index

	Accession	Mass	Score	Description
1.	G0ENS5	75722	429	G0ENS5_BRAIP Elongation factor G OS=Brachyspira intermedia (strain ATCC 51140 / PWS/A) GN=fusA PE=3 SV=1
2.	D8ICZ7	75831	429	D8ICZ7_BRAP9 Elongation factor G OS=Brachyspira pilosicoli (strain ATCC BAA-1826 / 95/1000) GN=fusA PE=3 SV=1
3.	J9UH41	77570	428	J9UH41_BRAPL Elongation factor G OS=Brachyspira pilosicoli B2904 GN=el-G PE=3 SV=1
4.	K0JMR3	77568	428	K0JMR3_BRAPL Elongation factor G OS=Brachyspira pilosicoli WesB GN=elG PE=3 SV=1
5.	C0QVZ3	75708	364	C0QVZ3_BRAHW Elongation factor G OS=Brachyspira hyodysenteriae (strain ATCC 49526 / WA1) GN=fusA PE=3 SV=1
6.	A0A0H0UHC1	77391	364	A0A0H0UHC1_BRAHO Elongation factor G OS=Brachyspira hyodysenteriae GN=fusA PE=3 SV=1
7.	A0A0H0VJ13	77405	364	A0A0H0VJ13_BRAHO Elongation factor G OS=Brachyspira hyodysenteriae GN=fusA PE=3 SV=1
8.	A0A0G4K3H3	77391	364	A0A0G4K3H3_9SPIR Elongation factor G OS=Brachyspira suanatina GN=fusA2 PE=3 SV=1
9.	A0A0H0UDC6	77405	364	A0A0H0UDC6_BRAHO Elongation factor G OS=Brachyspira hyodysenteriae GN=fusA PE=3 SV=1
10.	A0A0H0UJ78	77347	364	A0A0H0UJ78_BRAHO Elongation factor G OS=Brachyspira hyodysenteriae GN=fusA PE=3 SV=1
11.	L8XVJ7	77428	364	L8XVJ7_9SPIR Elongation factor G OS=Brachyspira hampsonii 30599 GN=fusA PE=3 SV=1
12.	L0X297	77383	364	L0X297_9SPIR Elongation factor G OS=Brachyspira hampsonii 30446 GN=fusA PE=3 SV=1
13.	R5LFK5	77531	297	R5LFK5_9SPIR Elongation factor G OS=Brachyspira sp. CAG:700 GN=fusA PE=3 SV=1
14.	A0A0H0V822	77418	237	A0A0H0V822_BRAHO Elongation factor G OS=Brachyspira hyodysenteriae GN=fusA PE=3 SV=1
15.	D5UB42	77473	215	D5UB42_BRAM5 Elongation factor G OS=Brachyspira murdochii (strain ATCC 51284 / DSM 12563 / 56-150) GN=fusA PE=
16.	R5HJM8	9899	40	R5HJM8_9SPIR Uncharacterized protein OS=Brachyspira sp. CAG:484 GN=BN676_01511 PE=4 SV=1

Results List

1. G0ENS5 **Mass:** 75722 **Score:** 429 **Expect:** 5.1e-39 **Queries matched:** 5
G0ENS5_BRAIP Elongation factor G OS=Brachyspira intermedia (strain ATCC 51140 / PWS/A) GN=fusA PE=3 SV=1

Observed	Mr(expt)	Mr(calc)	ppm	Start	End	Miss	Ions	Peptide
1093.6030	1092.5957	1092.5750	19.0	116 -	124	1	24	R.AIFVNKMDR.I
1738.9197	1737.9124	1737.8798	18.8	125 -	139	1	42	R.IGANFYAVLDQTRDR.L
1895.0137	1894.0064	1893.9697	19.4	451 -	466	1	47	R.EYKVEANVGRPQVSYR.E
2026.0645	2025.0572	2025.0167	20.0	62 -	79	0	130	R.INLIDTPGHVDFTAEVER.S
2071.0918	2070.0845	2070.0456	18.8	624 -	642	0	107	K.SINATVPLAEMFGYTTSIR.N

2. D8ICZ7 **Mass:** 75831 **Score:** 429 **Expect:** 5.1e-39 **Queries matched:** 5
D8ICZ7_BRAP9 Elongation factor G OS=Brachyspira pilosicoli (strain ATCC BAA-1826 / 95/1000) GN=fusA PE=3 SV=1

Observed	Mr(expt)	Mr(calc)	ppm	Start	End	Miss	Ions	Peptide
1093.6030	1092.5957	1092.5750	19.0	116 -	124	1	24	R.AIFVNKMDR.I
1738.9197	1737.9124	1737.8798	18.8	125 -	139	1	42	R.IGANFYAVLDQTRDR.L
1895.0137	1894.0064	1893.9697	19.4	451 -	466	1	47	R.EYKVEANVGRPQVSYR.E
2026.0645	2025.0572	2025.0167	20.0	62 -	79	0	130	R.INLIDTPGHVDFTAEVER.S
2071.0918	2070.0845	2070.0456	18.8	624 -	642	0	107	K.SINATVPLAEMFGYTTSIR.N

3. J9UH41 **Mass:** 77570 **Score:** 428 **Expect:** 6.4e-39 **Queries matched:** 5
J9UH41_BRAPL Elongation factor G OS=Brachyspira pilosicoli B2904 GN=el-G PE=3 SV=1

Observed	Mr(expt)	Mr(calc)	ppm	Start	End	Miss	Ions	Peptide
1093.6030	1092.5957	1092.5750	19.0	131 -	139	1	24	R.AIFVNKMDR.I
1738.9197	1737.9124	1737.8798	18.8	140 -	154	1	42	R.IGANFYAVLDQTRDR.L
1895.0137	1894.0064	1893.9697	19.4	466 -	481	1	47	R.EYKVEANVGRPQVSYR.E

2026.0645	2025.0572	2025.0167	20.0	77 - 94	0	130	R.INLIDTPGHVDFTAEVER.S
2071.0918	2070.0845	2070.0456	18.8	639 - 657	0	107	K.SINATVPLAEMFGYTTSIR.N

4. K0JMR3 **Mass:** 77568 **Score:** 428 **Expect:** 6.4e-39 **Queries matched:** 5
K0JMR3_BRAPL Elongation factor G OS=Brachyspira pilosicoli WesB GN=elG PE=3 SV=1

Observed	Mr(expt)	Mr(calc)	ppm	Start End	Miss	Ions	Peptide
1093.6030	1092.5957	1092.5750	19.0	131 - 139	1	24	R.AIFVNKMDR.I
1738.9197	1737.9124	1737.8798	18.8	140 - 154	1	42	R.IGANFYAVLDQTRDR.L
1895.0137	1894.0064	1893.9697	19.4	466 - 481	1	47	R.EYKVEANVGRPQVSYR.E
2026.0645	2025.0572	2025.0167	20.0	77 - 94	0	130	R.INLIDTPGHVDFTAEVER.S
2071.0918	2070.0845	2070.0456	18.8	639 - 657	0	107	K.SINATVPLAEMFGYTTSIR.N

5. C0QVZ3 **Mass:** 75708 **Score:** 364 **Expect:** 1.6e-32 **Queries matched:** 4
C0QVZ3_BRAHW Elongation factor G OS=Brachyspira hyodysenteriae (strain ATCC 49526 / WA1) GN=fusA PE=3 SV=1

Observed	Mr(expt)	Mr(calc)	ppm	Start End	Miss	Ions	Peptide
1093.6030	1092.5957	1092.5750	19.0	116 - 124	1	24	R.AIFVNKMDR.I
1895.0137	1894.0064	1893.9697	19.4	451 - 466	1	47	R.EYKVEANVGRPQVSYR.E
2026.0645	2025.0572	2025.0167	20.0	62 - 79	0	130	R.INLIDTPGHVDFTAEVER.S
2071.0918	2070.0845	2070.0456	18.8	624 - 642	0	107	K.SINATVPLAEMFGYTTSIR.N

No match to: 1738.9197

6. A0A0H0UHC1 **Mass:** 77391 **Score:** 364 **Expect:** 1.6e-32 **Queries matched:** 4
A0A0H0UHC1_BRAHO Elongation factor G OS=Brachyspira hyodysenteriae GN=fusA PE=3 SV=1

Observed	Mr(expt)	Mr(calc)	ppm	Start End	Miss	Ions	Peptide
1093.6030	1092.5957	1092.5750	19.0	131 - 139	1	24	R.AIFVNKMDR.I
1895.0137	1894.0064	1893.9697	19.4	466 - 481	1	47	R.EYKVEANVGRPQVSYR.E
2026.0645	2025.0572	2025.0167	20.0	77 - 94	0	130	R.INLIDTPGHVDFTAEVER.S
2071.0918	2070.0845	2070.0456	18.8	639 - 657	0	107	K.SINATVPLAEMFGYTTSIR.N

No match to: 1738.9197

7. A0A0H0VJ13 **Mass:** 77405 **Score:** 364 **Expect:** 1.6e-32 **Queries matched:** 4
A0A0H0VJ13_BRAHO Elongation factor G OS=Brachyspira hyodysenteriae GN=fusA PE=3 SV=1

Observed	Mr(expt)	Mr(calc)	ppm	Start End	Miss	Ions	Peptide
1093.6030	1092.5957	1092.5750	19.0	131 - 139	1	24	R.AIFVNKMDR.I
1895.0137	1894.0064	1893.9697	19.4	466 - 481	1	47	R.EYKVEANVGRPQVSYR.E
2026.0645	2025.0572	2025.0167	20.0	77 - 94	0	130	R.INLIDTPGHVDFTAEVER.S
2071.0918	2070.0845	2070.0456	18.8	639 - 657	0	107	K.SINATVPLAEMFGYTTSIR.N

No match to: 1738.9197

8. A0A0G4K3H3 **Mass:** 77391 **Score:** 364 **Expect:** 1.6e-32 **Queries matched:** 4
A0A0G4K3H3_9SPIR Elongation factor G OS=Brachyspira suanatina GN=fusA2 PE=3 SV=1

Observed	Mr(expt)	Mr(calc)	ppm	Start End	Miss	Ions	Peptide
1093.6030	1092.5957	1092.5750	19.0	131 - 139	1	24	R.AIFVNKMDR.I
1895.0137	1894.0064	1893.9697	19.4	466 - 481	1	47	R.EYKVEANVGRPQVSYR.E
2026.0645	2025.0572	2025.0167	20.0	77 - 94	0	130	R.INLIDTPGHVDFTAEVER.S
2071.0918	2070.0845	2070.0456	18.8	639 - 657	0	107	K.SINATVPLAEMFGYTTSIR.N

No match to: 1738.9197

9. A0A0H0UDC6 **Mass:** 77405 **Score:** 364 **Expect:** 1.6e-32 **Queries matched:** 4
A0A0H0UDC6_BRAHO Elongation factor G OS=Brachyspira hyodysenteriae GN=fusA PE=3 SV=1

Observed	Mr(expt)	Mr(calc)	ppm	Start End	Miss	Ions	Peptide
1093.6030	1092.5957	1092.5750	19.0	131 - 139	1	24	R.AIFVNKMDR.I
1895.0137	1894.0064	1893.9697	19.4	466 - 481	1	47	R.EYKVEANVGRPQVSYR.E
2026.0645	2025.0572	2025.0167	20.0	77 - 94	0	130	R.INLIDTPGHVDFTAEVER.S
2071.0918	2070.0845	2070.0456	18.8	639 - 657	0	107	K.SINATVPLAEMFGYTTSIR.N

No match to: 1738.9197

10. A0A0H0UJ78 **Mass:** 77347 **Score:** 364 **Expect:** 1.6e-32 **Queries matched:** 4
A0A0H0UJ78_BRAHO Elongation factor G OS=Brachyspira hyodysenteriae GN=fusA PE=3 SV=1

Observed	Mr(expt)	Mr(calc)	ppm	Start End	Miss	Ions	Peptide
1093.6030	1092.5957	1092.5750	19.0	131 - 139	1	24	R.AIFVNKMDR.I
1895.0137	1894.0064	1893.9697	19.4	466 - 481	1	47	R.EYKVEANVGRPQVSYR.E
2026.0645	2025.0572	2025.0167	20.0	77 - 94	0	130	R.INLIDTPGHVDFTAEVER.S
2071.0918	2070.0845	2070.0456	18.8	639 - 657	0	107	K.SINATVPLAEMFGYTTSIR.N

No match to: 1738.9197

11. L8XVJ7 **Mass:** 77428 **Score:** 364 **Expect:** 1.6e-32 **Queries matched:** 4
L8XVJ7_9SPIR Elongation factor G OS=Brachyspira hampsonii 30599 GN=fusA PE=3 SV=1

Observed	Mr(expt)	Mr(calc)	ppm	Start End	Miss	Ions	Peptide
1093.6030	1092.5957	1092.5750	19.0	131 - 139	1	24	R.AIFVNKMDR.I
1895.0137	1894.0064	1893.9697	19.4	466 - 481	1	47	R.EYKVEANVGRPQVSYR.E
2026.0645	2025.0572	2025.0167	20.0	77 - 94	0	130	R.INLIDTPGHVDFTAEVER.S
2071.0918	2070.0845	2070.0456	18.8	639 - 657	0	107	K.SINATVPLAEMFGYTTSIR.N

No match to: 1738.9197

12. L0X297 **Mass:** 77383 **Score:** 364 **Expect:** 1.6e-32 **Queries matched:** 4
L0X297_9SPIR Elongation factor G OS=Brachyspira hampsonii 30446 GN=fusA PE=3 SV=1

Observed	Mr(expt)	Mr(calc)	ppm	Start End	Miss	Ions	Peptide
1093.6030	1092.5957	1092.5750	19.0	131 - 139	1	24	R.AIFVNKMDR.I
1895.0137	1894.0064	1893.9697	19.4	466 - 481	1	47	R.EYKVEANVGRPQVSYR.E
2026.0645	2025.0572	2025.0167	20.0	77 - 94	0	130	R.INLIDTPGHVDFTAEVER.S
2071.0918	2070.0845	2070.0456	18.8	639 - 657	0	107	K.SINATVPLAEMFGYTTSIR.N

No match to: 1738.9197

13. R5LFK5 **Mass:** 77531 **Score:** 297 **Expect:** 8.1e-26 **Queries matched:** 3
R5LFK5_9SPIR Elongation factor G OS=Brachyspira sp. CAG:700 GN=fusA PE=3 SV=1

Observed	Mr(expt)	Mr(calc)	ppm	Start	End	Miss	Ions	Peptide
1093.6030	1092.5957	1092.5750	19.0	131 -	139	1	24	R.AIFVNKMDR.I
2026.0645	2025.0572	2025.0167	20.0	77 -	94	0	130	R.INLIDTPGHVDFTAEVER.S
2071.0918	2070.0845	2070.0456	18.8	639 -	657	0	107	K.SINATVPLAEMFGYTTSIR.N

No match to: 1738.9197, 1895.0137

14. A0A0H0V822 **Mass:** 77418 **Score:** 237 **Expect:** 8.1e-20 **Queries matched:** 3
A0A0H0V822_BRAHO Elongation factor G OS=Brachyspira hyodysenteriae GN=fusA PE=3 SV=1

Observed	Mr(expt)	Mr(calc)	ppm	Start	End	Miss	Ions	Peptide
1093.6030	1092.5957	1092.5750	19.0	131 -	139	1	24	R.AIFVNKMDR.I
1895.0137	1894.0064	1893.9697	19.4	466 -	481	1	47	R.EYKVEANVGRPQVSYR.E
2026.0645	2025.0572	2025.0167	20.0	77 -	94	0	130	R.INLIDTPGHVDFTAEVER.S

No match to: 1738.9197, 2071.0918

15. D5UB42 **Mass:** 77473 **Score:** 215 **Expect:** 1.3e-17 **Queries matched:** 3
D5UB42_BRAM5 Elongation factor G OS=Brachyspira murdochii (strain ATCC 51284 / DSM 12563 / 56-150) GN=fusA PE=3 SV=1

Observed	Mr(expt)	Mr(calc)	ppm	Start	End	Miss	Ions	Peptide
1093.6030	1092.5957	1092.5750	19.0	131 -	139	1	24	R.AIFVNKMDR.I
1895.0137	1894.0064	1893.9697	19.4	466 -	481	1	47	R.EYKVEANVGRPQVSYR.E
2071.0918	2070.0845	2070.0456	18.8	639 -	657	0	107	K.SINATVPLAEMFGYTTSIR.N

No match to: 1738.9197, 2026.0645

16. R5HJM8 **Mass:** 9899 **Score:** 40 **Expect:** 4 **Queries matched:** 2
R5HJM8_9SPIR Uncharacterized protein OS=Brachyspira sp. CAG:484 GN=BN676_01511 PE=4 SV=1

Observed	Mr(expt)	Mr(calc)	ppm	Start	End	Miss	Ions	Peptide
1895.0137	1894.0064	1893.9982	4.33	2 -	18	0	---	M.MNTIQPVQTIQPPINGK.S + Oxidation (M)
2026.0645	2025.0572	2025.0387	9.14	1 -	18	0	---	-.MMNTIQPVQTIQPPINGK.S + Oxidation (M)

No match to: 1093.6030, 1738.9197, 2071.0918

Search Parameters

Type of search : Sequence Query
Enzyme : Trypsin
Fixed modifications : Carbamidomethyl (C)
Variable modifications : Oxidation (M)
Mass values : Monoisotopic
Protein Mass : Unrestricted
Peptide Mass Tolerance : ± 50 ppm
Fragment Mass Tolerance: ± 0.5 Da
Max Missed Cleavages : 1
Instrument type : MALDI-TOF-TOF
Query1 (1093.6030,1+) : Locus:4.1.1.212738.57018
Query2 (1738.9197,1+) : Locus:4.1.1.212738.57017
Query3 (1895.0137,1+) : Locus:4.1.1.212738.57016
Query4 (2026.0645,1+) : Locus:4.1.1.212738.57014
Query5 (2071.0918,1+) : Locus:4.1.1.212738.57015

Mascot: http://www.matrixscience.com/

(MATRIX SCIENCE) Mascot Search Results

```
User          :
Email         :
Search title  : F:\ROD_inmunoproteoma\MALDI\ppw_2014_442_ROD\ppw_G7_139601421306.txt
MS data file  : F:\ROD_inmunoproteoma\MALDI\ppw_2014_442_ROD\ppw_G7_139601421306.txt
Database      : Brachyspiraceae Brachyspiraceae_20160127-151122 (40573 sequences; 13470793 residues)
Timestamp     : 22 Feb 2016 at 15:14:03 GMT
Warning       : A Peptide summary report will usually give a much clearer picture of MS/MS search results.
Top Score     : 170 for D8IDC2, D8IDC2_BRAP9 Uncharacterized protein OS=Brachyspira pilosicoli (strain ATCC BAA-1826 / 95/1000) GN=BP95100
```

Probability Based Mowse Score

Protein score is -10*Log(P), where P is the probability that the observed match is a random event.
Protein scores greater than 59 are significant (p<0.05).
Protein scores are derived from ions scores as a non-probabilistic basis for ranking protein hits.

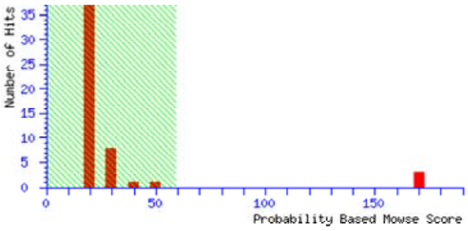

Protein Summary Report

Format As | Protein Summary (deprecated) | Help

Significance threshold p< 0.05 | Max. number of hits AUTO

Standard scoring ◉ MudPIT scoring ○ | Ions score or expect cut-off 0 | Show sub-sets 0

Show pop-ups ◉ Suppress pop-ups ○ | Sort unassigned Decreasing Score | Require bold red ☐

Re-Search All | Search Unmatched

Index

	Accession	Mass	Score	Description
1.	D8IDC2	62081	170	D8IDC2_BRAP9 Uncharacterized protein OS=Brachyspira pilosicoli (strain ATCC BAA-1826 / 95/1000) GN=BP951000_1156
2.	K0JN47	62251	170	K0JN47_BRAPL Putative treponemal membrane protein OS=Brachyspira pilosicoli WesB GN=tmpB PE=4 SV=1
3.	J9UXF7	62380	170	J9UXF7_BRAPL Putative treponemal membrane protein OS=Brachyspira pilosicoli B2904 GN=tmpB PE=4 SV=1
4.	R5L7Q6	47801	51	R5L7Q6_9SPIR Galactokinase OS=Brachyspira sp. CAG:700 GN=BN758_01141 PE=3 SV=1

Results List

1. D8IDC2 **Mass:** 62081 **Score:** 170 **Expect:** 4.1e-13 **Queries matched:** 2
D8IDC2_BRAP9 Uncharacterized protein OS=Brachyspira pilosicoli (strain ATCC BAA-1826 / 95/1000) GN=BP951000_1156 PE=4 SV=1

Observed	Mr(expt)	Mr(calc)	ppm	Start	End	Miss	Ions	Peptide
1435.8481	1434.8408	1434.8235	12.1	462 -	473	0	47	K.VTVLPQYYVVVR.R
1795.8529	1794.8456	1794.8272	10.3	33 -	48	0	95	R.ITGEAETLQNDGEYQK.S

No match to: 713.4030

2. K0JN47 **Mass:** 62251 **Score:** 170 **Expect:** 4.1e-13 **Queries matched:** 2
K0JN47_BRAPL Putative treponemal membrane protein OS=Brachyspira pilosicoli WesB GN=tmpB PE=4 SV=1

Observed	Mr(expt)	Mr(calc)	ppm	Start	End	Miss	Ions	Peptide
1435.8481	1434.8408	1434.8235	12.1	462 -	473	0	47	K.VTVLPQYYVVVR.R
1795.8529	1794.8456	1794.8272	10.3	33 -	48	0	95	R.ITGEAETLQNDGEYQK.S

No match to: 713.4030

3. J9UXF7 **Mass:** 62380 **Score:** 170 **Expect:** 4.1e-13 **Queries matched:** 2
J9UXF7_BRAPL Putative treponemal membrane protein OS=Brachyspira pilosicoli B2904 GN=tmpB PE=4 SV=1

Observed	Mr(expt)	Mr(calc)	ppm	Start	End	Miss	Ions	Peptide
1435.8481	1434.8408	1434.8235	12.1	464 -	475	0	47	K.VTVLPQYYVVVR.R
1795.8529	1794.8456	1794.8272	10.3	33 -	48	0	95	R.ITGEAETLQNDGEYQK.S

No match to: 713.4030

4. R5L7Q6 **Mass:** 47801 **Score:** 51 **Expect:** 0.29 **Queries matched:** 3
R5L7Q6_9SPIR Galactokinase OS=Brachyspira sp. CAG:700 GN=BN758_01141 PE=3 SV=1

Observed	Mr(expt)	Mr(calc)	ppm	Start	End	Miss	Ions	Peptide
713.4030	712.3957	712.4119	-22.79	228 -	233	0	---	K.NPIIEK.V
1435.8481	1434.8408	1434.7718	48.1	38 -	50	1	---	R.LSSALGEFQKIDK.S
1795.8529	1794.8456	1794.7771	38.2	398 -	412	0	---	K.VEEYTNYMNSVFGDK.S

Search Parameters

```
Type of search         : Sequence Query
Enzyme                 : Trypsin
Fixed modifications    : Carbamidomethyl (C)
Variable modifications : Oxidation (M)
Mass values            : Monoisotopic
```

```
Protein Mass           : Unrestricted
Peptide Mass Tolerance : ± 50 ppm
Fragment Mass Tolerance: ± 0.5 Da
Max Missed Cleavages   : 1
Instrument type        : MALDI-TOF-TOF
Query1 (713.4030,1+)   : Locus:7.1.1.170605.52753
Query2 (1435.8481,1+)  : Locus:7.1.1.170605.52754
Query3 (1795.8529,1+)  : Locus:7.1.1.170605.52755
```

**Mascot:** http://www.matrixscience.com/

Mascot Search Results

```
User         :
Email        :
Search title : F:\ROD_inmunoproteoma\MALDI\ppw_2016_562_563_ROD\ppw_H12_145397049216.txt
MS data file : F:\ROD_inmunoproteoma\MALDI\ppw_2016_562_563_ROD\ppw_H12_145397049216.txt
Database     : Brachyspiraceae Brachyspiraceae_20160127-151122 (40573 sequences; 13470793 residues)
Timestamp    : 23 Feb 2016 at 16:05:43 GMT
Warning      : A Peptide summary report will usually give a much clearer picture of MS/MS search results.
Top Score    : 264 for C0QVZ3, C0QVZ3_BRAHW Elongation factor G OS=Brachyspira hyodysenteriae (strain ATCC 49526 / WA1) GN=fusA PE=3 SV=1
```

Probability Based Mowse Score

Protein score is -10*Log(P), where P is the probability that the observed match is a random event.
Protein scores greater than 59 are significant (p<0.05).
Protein scores are derived from ions scores as a non-probabilistic basis for ranking protein hits.

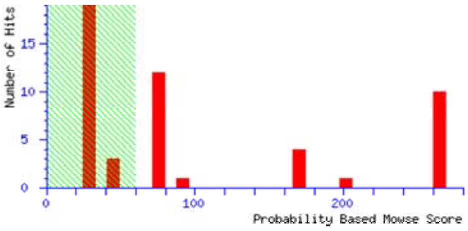

Protein Summary Report

Format As | Protein Summary (deprecated) | Help

Significance threshold p< 0.05 | Max. number of hits AUTO

Standard scoring ◉ MudPIT scoring ○ | Ions score or expect cut-off 0 | Show sub-sets 0

Show pop-ups ◉ Suppress pop-ups ○ | Sort unassigned Decreasing Score | Require bold red ☐

Re-Search All | Search Unmatched

Index

	Accession	Mass	Score	Description
1.	C0QVZ3	75708	264	C0QVZ3_BRAHW Elongation factor G OS=Brachyspira hyodysenteriae (strain ATCC 49526 / WA1) GN=fusA PE=3 SV=1
2.	G0ENS5	75722	264	G0ENS5_BRAIP Elongation factor G OS=Brachyspira intermedia (strain ATCC 51140 / PWS/A) GN=fusA PE=3 SV=1
3.	D8ICZ7	75831	264	D8ICZ7_BRAP9 Elongation factor G OS=Brachyspira pilosicoli (strain ATCC BAA-1826 / 95/1000) GN=fusA PE=3 SV=1
4.	A0A0H0UHC1	77391	264	A0A0H0UHC1_BRAHO Elongation factor G OS=Brachyspira hyodysenteriae GN=fusA PE=3 SV=1
5.	A0A0H0VJ13	77405	264	A0A0H0VJ13_BRAHO Elongation factor G OS=Brachyspira hyodysenteriae GN=fusA PE=3 SV=1
6.	A0A0G4K3H3	77391	264	A0A0G4K3H3_9SPIR Elongation factor G OS=Brachyspira suanatina GN=fusA2 PE=3 SV=1
7.	A0A0H0UDC6	77405	264	A0A0H0UDC6_BRAHO Elongation factor G OS=Brachyspira hyodysenteriae GN=fusA PE=3 SV=1
8.	A0A0H0UJ78	77347	264	A0A0H0UJ78_BRAHO Elongation factor G OS=Brachyspira hyodysenteriae GN=fusA PE=3 SV=1
9.	J9UH41	77570	264	J9UH41_BRAPL Elongation factor G OS=Brachyspira pilosicoli B2904 GN=el-G PE=3 SV=1
10.	K0JMR3	77568	264	K0JMR3_BRAPL Elongation factor G OS=Brachyspira pilosicoli WesB GN=elG PE=3 SV=1
11.	D5UB42	77473	195	D5UB42_BRAM5 Elongation factor G OS=Brachyspira murdochii (strain ATCC 51284 / DSM 12563 / 56-150) GN=fusA PE=
12.	A0A0H0V822	77418	176	A0A0H0V822_BRAHO Elongation factor G OS=Brachyspira hyodysenteriae GN=fusA PE=3 SV=1
13.	R5LFK5	77531	165	R5LFK5_9SPIR Elongation factor G OS=Brachyspira sp. CAG:700 GN=fusA PE=3 SV=1
14.	L8XVJ7	77428	163	L8XVJ7_9SPIR Elongation factor G OS=Brachyspira hampsonii 30599 GN=fusA PE=3 SV=1
15.	L0X297	77383	163	L0X297_9SPIR Elongation factor G OS=Brachyspira hampsonii 30446 GN=fusA PE=3 SV=1
16.	A0A0G4K8D6	78989	88	A0A0G4K8D6_9SPIR Polyribonucleotide nucleotidyltransferase OS=Brachyspira suanatina GN=pnp PE=3 SV=1
17.	G0ELF8	78247	75	G0ELF8_BRAIP Polyribonucleotide nucleotidyltransferase OS=Brachyspira intermedia (strain ATCC 51140 / PWS/A) G
18.	C0R0E0	78954	75	PNP_BRAHW Polyribonucleotide nucleotidyltransferase OS=Brachyspira hyodysenteriae (strain ATCC 49526 / WA1) GN
19.	A0A0H0WD31	78954	75	A0A0H0WD31_BRAHO Polyribonucleotide nucleotidyltransferase OS=Brachyspira hyodysenteriae GN=pnp PE=3 SV=1
20.	A0A0H0U557	78971	75	A0A0H0U557_BRAHO Polyribonucleotide nucleotidyltransferase OS=Brachyspira hyodysenteriae GN=pnp PE=3 SV=1
21.	A0A0H0V372	78998	75	A0A0H0V372_BRAHO Polyribonucleotide nucleotidyltransferase OS=Brachyspira hyodysenteriae GN=pnp PE=3 SV=1
22.	A0A0H0TY55	79015	75	A0A0H0TY55_BRAHO Polyribonucleotide nucleotidyltransferase OS=Brachyspira hyodysenteriae GN=pnp PE=3 SV=1
23.	J9UKS0	79013	75	J9UKS0_BRAPL Polyribonucleotide nucleotidyltransferase OS=Brachyspira pilosicoli B2904 GN=pnp PE=3 SV=1
24.	D8IEN6	78999	75	D8IEN6_BRAP9 Polyribonucleotide nucleotidyltransferase OS=Brachyspira pilosicoli (strain ATCC BAA-1826 / 95/10
25.	K0JF68	79013	75	K0JF68_BRAPL Polyribonucleotide nucleotidyltransferase OS=Brachyspira pilosicoli WesB GN=pnp PE=3 SV=1
26.	L0WY06	78755	75	L0WY06_9SPIR Polyribonucleotide nucleotidyltransferase OS=Brachyspira hampsonii 30446 GN=pnp PE=3 SV=1
27.	L8XSI1	79053	75	L8XSI1_9SPIR Polyribonucleotide nucleotidyltransferase OS=Brachyspira hampsonii 30599 GN=pnp PE=3 SV=1
28.	D5U7E8	79436	75	D5U7E8_BRAM5 Polyribonucleotide nucleotidyltransferase OS=Brachyspira murdochii (strain ATCC 51284 / DSM 12563
29.	R5HB49	7827	42	R5HB49_9SPIR Uncharacterized protein OS=Brachyspira sp. CAG:484 GN=BN676_01916 PE=4 SV=1

Results List

1. C0QVZ3 **Mass:** 75708 **Score:** 264 **Expect:** 1.6e-22 **Queries matched:** 3
C0QVZ3_BRAHW Elongation factor G OS=Brachyspira hyodysenteriae (strain ATCC 49526 / WA1) GN=fusA PE=3 SV=1

Observed	Mr(expt)	Mr(calc)	ppm	Start	End	Miss	Ions	Peptide
1467.7052	1466.6979	1466.7518	-36.71	125 -	137	0	85	R.IGANFYAVLDQTR.E
2025.9441	2024.9368	2025.0167	-39.46	62 -	79	0	70	R.INLIDTPGHVDFTAEVER.S
2070.9729	2069.9656	2070.0456	-38.62	624 -	642	0	72	K.SINATVPLAEMFGYTTSIR.N

No match to: 987.5048, 1993.0137

2. G0ENS5 **Mass:** 75722 **Score:** 264 **Expect:** 1.6e-22 **Queries matched:** 3
G0ENS5_BRAIP Elongation factor G OS=Brachyspira intermedia (strain ATCC 51140 / PWS/A) GN=fusA PE=3 SV=1

Observed	Mr(expt)	Mr(calc)	ppm	Start	End	Miss	Ions	Peptide
1467.7052	1466.6979	1466.7518	-36.71	125 -	137	0	85	R.IGANFYAVLDQTR.D
2025.9441	2024.9368	2025.0167	-39.46	62 -	79	0	70	R.INLIDTPGHVDFTAEVER.S

2070.9729	2069.9656	2070.0456	-38.62	624	642	0	72	K.SINATVPLAEMFGYTTSIR.N

No match to: 987.5048, 1993.0137

3. D8ICZ7 **Mass:** 75831 **Score:** 264 **Expect:** 1.6e-22 **Queries matched:** 3
D8ICZ7_BRAP9 Elongation factor G OS=Brachyspira pilosicoli (strain ATCC BAA-1826 / 95/1000) GN=fusA PE=3 SV=1

Observed	Mr(expt)	Mr(calc)	ppm	Start	End	Miss	Ions	Peptide
1467.7052	1466.6979	1466.7518	-36.71	125	137	0	85	R.IGANFYAVLDQTR.D
2025.9441	2024.9368	2025.0167	-39.46	62	79	0	70	R.INLIDTPGHVDFTAEVER.S
2070.9729	2069.9656	2070.0456	-38.62	624	642	0	72	K.SINATVPLAEMFGYTTSIR.N

No match to: 987.5048, 1993.0137

4. A0A0H0UHC1 **Mass:** 77391 **Score:** 264 **Expect:** 1.6e-22 **Queries matched:** 3
A0A0H0UHC1_BRAHO Elongation factor G OS=Brachyspira hyodysenteriae GN=fusA PE=3 SV=1

Observed	Mr(expt)	Mr(calc)	ppm	Start	End	Miss	Ions	Peptide
1467.7052	1466.6979	1466.7518	-36.71	140	152	0	85	R.IGANFYAVLDQTR.E
2025.9441	2024.9368	2025.0167	-39.46	77	94	0	70	R.INLIDTPGHVDFTAEVER.S
2070.9729	2069.9656	2070.0456	-38.62	639	657	0	72	K.SINATVPLAEMFGYTTSIR.N

No match to: 987.5048, 1993.0137

5. A0A0H0VJ13 **Mass:** 77405 **Score:** 264 **Expect:** 1.6e-22 **Queries matched:** 3
A0A0H0VJ13_BRAHO Elongation factor G OS=Brachyspira hyodysenteriae GN=fusA PE=3 SV=1

Observed	Mr(expt)	Mr(calc)	ppm	Start	End	Miss	Ions	Peptide
1467.7052	1466.6979	1466.7518	-36.71	140	152	0	85	R.IGANFYAVLDQTR.E
2025.9441	2024.9368	2025.0167	-39.46	77	94	0	70	R.INLIDTPGHVDFTAEVER.S
2070.9729	2069.9656	2070.0456	-38.62	639	657	0	72	K.SINATVPLAEMFGYTTSIR.N

No match to: 987.5048, 1993.0137

6. A0A0G4K3H3 **Mass:** 77391 **Score:** 264 **Expect:** 1.6e-22 **Queries matched:** 3
A0A0G4K3H3_9SPIR Elongation factor G OS=Brachyspira suanatina GN=fusA2 PE=3 SV=1

Observed	Mr(expt)	Mr(calc)	ppm	Start	End	Miss	Ions	Peptide
1467.7052	1466.6979	1466.7518	-36.71	140	152	0	85	R.IGANFYAVLDQTR.E
2025.9441	2024.9368	2025.0167	-39.46	77	94	0	70	R.INLIDTPGHVDFTAEVER.S
2070.9729	2069.9656	2070.0456	-38.62	639	657	0	72	K.SINATVPLAEMFGYTTSIR.N

No match to: 987.5048, 1993.0137

7. A0A0H0UDC6 **Mass:** 77405 **Score:** 264 **Expect:** 1.6e-22 **Queries matched:** 3
A0A0H0UDC6_BRAHO Elongation factor G OS=Brachyspira hyodysenteriae GN=fusA PE=3 SV=1

Observed	Mr(expt)	Mr(calc)	ppm	Start	End	Miss	Ions	Peptide
1467.7052	1466.6979	1466.7518	-36.71	140	152	0	85	R.IGANFYAVLDQTR.E
2025.9441	2024.9368	2025.0167	-39.46	77	94	0	70	R.INLIDTPGHVDFTAEVER.S
2070.9729	2069.9656	2070.0456	-38.62	639	657	0	72	K.SINATVPLAEMFGYTTSIR.N

No match to: 987.5048, 1993.0137

8. A0A0H0UJ78 **Mass:** 77347 **Score:** 264 **Expect:** 1.6e-22 **Queries matched:** 3
A0A0H0UJ78_BRAHO Elongation factor G OS=Brachyspira hyodysenteriae GN=fusA PE=3 SV=1

Observed	Mr(expt)	Mr(calc)	ppm	Start	End	Miss	Ions	Peptide
1467.7052	1466.6979	1466.7518	-36.71	140	152	0	85	R.IGANFYAVLDQTR.E
2025.9441	2024.9368	2025.0167	-39.46	77	94	0	70	R.INLIDTPGHVDFTAEVER.S
2070.9729	2069.9656	2070.0456	-38.62	639	657	0	72	K.SINATVPLAEMFGYTTSIR.N

No match to: 987.5048, 1993.0137

9. J9UH41 **Mass:** 77570 **Score:** 264 **Expect:** 1.6e-22 **Queries matched:** 3
J9UH41_BRAPL Elongation factor G OS=Brachyspira pilosicoli B2904 GN=el-G PE=3 SV=1

Observed	Mr(expt)	Mr(calc)	ppm	Start	End	Miss	Ions	Peptide
1467.7052	1466.6979	1466.7518	-36.71	140	152	0	85	R.IGANFYAVLDQTR.D
2025.9441	2024.9368	2025.0167	-39.46	77	94	0	70	R.INLIDTPGHVDFTAEVER.S
2070.9729	2069.9656	2070.0456	-38.62	639	657	0	72	K.SINATVPLAEMFGYTTSIR.N

No match to: 987.5048, 1993.0137

10. K0JMR3 **Mass:** 77568 **Score:** 264 **Expect:** 1.6e-22 **Queries matched:** 3
K0JMR3_BRAPL Elongation factor G OS=Brachyspira pilosicoli WesB GN=elG PE=3 SV=1

Observed	Mr(expt)	Mr(calc)	ppm	Start	End	Miss	Ions	Peptide
1467.7052	1466.6979	1466.7518	-36.71	140	152	0	85	R.IGANFYAVLDQTR.D
2025.9441	2024.9368	2025.0167	-39.46	77	94	0	70	R.INLIDTPGHVDFTAEVER.S
2070.9729	2069.9656	2070.0456	-38.62	639	657	0	72	K.SINATVPLAEMFGYTTSIR.N

No match to: 987.5048, 1993.0137

11. D5UB42 **Mass:** 77473 **Score:** 195 **Expect:** 1.3e-15 **Queries matched:** 3
D5UB42_BRAM5 Elongation factor G OS=Brachyspira murdochii (strain ATCC 51284 / DSM 12563 / 56-150) GN=fusA PE=3 SV=1

Observed	Mr(expt)	Mr(calc)	ppm	Start	End	Miss	Ions	Peptide
1467.7052	1466.6979	1466.7518	-36.71	140	152	0	85	R.IGANFYAVLDQTR.E
2025.9441	2024.9368	2024.9376	-0.40	246	263	0	---	R.TATLTADFFPMFCGTAFK.N
2070.9729	2069.9656	2070.0456	-38.62	639	657	0	72	K.SINATVPLAEMFGYTTSIR.N

No match to: 987.5048, 1993.0137

12. A0A0H0V822 **Mass:** 77418 **Score:** 176 **Expect:** 1e-13 **Queries matched:** 2
A0A0H0V822_BRAHO Elongation factor G OS=Brachyspira hyodysenteriae GN=fusA PE=3 SV=1

Observed	Mr(expt)	Mr(calc)	ppm	Start	End	Miss	Ions	Peptide
1467.7052	1466.6979	1466.7518	-36.71	140	152	0	85	R.IGANFYAVLDQTR.E
2025.9441	2024.9368	2025.0167	-39.46	77	94	0	70	R.INLIDTPGHVDFTAEVER.S

No match to: 987.5048, 1993.0137, 2070.9729

13. R5LFK5 **Mass:** 77531 **Score:** 165 **Expect:** 1.3e-12 **Queries matched:** 2
R5LFK5_9SPIR Elongation factor G OS=Brachyspira sp. CAG:700 GN=fusA PE=3 SV=1

Observed	Mr(expt)	Mr(calc)	ppm	Start	End	Miss	Ions	Peptide
2025.9441	2024.9368	2025.0167	-39.46	77	94	0	70	R.INLIDTPGHVDFTAEVER.S
2070.9729	2069.9656	2070.0456	-38.62	639	657	0	72	K.SINATVPLAEMFGYTTSIR.N

No match to: 987.5048, 1467.7052, 1993.0137

14. L8XVJ7 **Mass:** 77428 **Score:** 163 **Expect:** 2e-12 **Queries matched:** 2
L8XVJ7_9SPIR Elongation factor G OS=Brachyspira hampsonii 30599 GN=fusA PE=3 SV=1

Observed	Mr(expt)	Mr(calc)	ppm	Start	End	Miss	Ions	Peptide
2025.9441	2024.9368	2025.0167	-39.46	77 -	94	0	70	R.INLIDTPGHVDFTAEVER.S
2070.9729	2069.9656	2070.0456	-38.62	639 -	657	0	72	K.SINATVPLAEMFGYTTSIR.N

No match to: 987.5048, 1467.7052, 1993.0137

15. L0X297 **Mass:** 77383 **Score:** 163 **Expect:** 2e-12 **Queries matched:** 2
L0X297_9SPIR Elongation factor G OS=Brachyspira hampsonii 30446 GN=fusA PE=3 SV=1

Observed	Mr(expt)	Mr(calc)	ppm	Start	End	Miss	Ions	Peptide
2025.9441	2024.9368	2025.0167	-39.46	77 -	94	0	70	R.INLIDTPGHVDFTAEVER.S
2070.9729	2069.9656	2070.0456	-38.62	639 -	657	0	72	K.SINATVPLAEMFGYTTSIR.N

No match to: 987.5048, 1467.7052, 1993.0137

16. A0A0G4K8D6 **Mass:** 78989 **Score:** 88 **Expect:** 7.2e-05 **Queries matched:** 2
A0A0G4K8D6_9SPIR Polyribonucleotide nucleotidyltransferase OS=Brachyspira suanatina GN=pnp PE=3 SV=1

Observed	Mr(expt)	Mr(calc)	ppm	Start	End	Miss	Ions	Peptide
987.5048	986.4975	986.5298	-32.67	335 -	343	0	67	R.VHGSALFTR.G
2025.9441	2024.9368	2025.0306	-46.30	686 -	702	1	---	R.KALLEKPADYVEEEYTK.K

No match to: 1467.7052, 1993.0137, 2070.9729

17. G0ELF8 **Mass:** 78247 **Score:** 75 **Expect:** 0.0014 **Queries matched:** 1
G0ELF8_BRAIP Polyribonucleotide nucleotidyltransferase OS=Brachyspira intermedia (strain ATCC 51140 / PWS/A) GN=pnp PE=3 SV=1

Observed	Mr(expt)	Mr(calc)	ppm	Start	End	Miss	Ions	Peptide
987.5048	986.4975	986.5298	-32.67	329 -	337	0	67	R.VHGSALFTR.G

No match to: 1467.7052, 1993.0137, 2025.9441, 2070.9729

18. C0R0E0 **Mass:** 78954 **Score:** 75 **Expect:** 0.0014 **Queries matched:** 1
PNP_BRAHW Polyribonucleotide nucleotidyltransferase OS=Brachyspira hyodysenteriae (strain ATCC 49526 / WA1) GN=pnp PE=3 SV=1

Observed	Mr(expt)	Mr(calc)	ppm	Start	End	Miss	Ions	Peptide
987.5048	986.4975	986.5298	-32.67	335 -	343	0	67	R.VHGSALFTR.G

No match to: 1467.7052, 1993.0137, 2025.9441, 2070.9729

19. A0A0H0WD31 **Mass:** 78954 **Score:** 75 **Expect:** 0.0014 **Queries matched:** 1
A0A0H0WD31_BRAHO Polyribonucleotide nucleotidyltransferase OS=Brachyspira hyodysenteriae GN=pnp PE=3 SV=1

Observed	Mr(expt)	Mr(calc)	ppm	Start	End	Miss	Ions	Peptide
987.5048	986.4975	986.5298	-32.67	335 -	343	0	67	R.VHGSALFTR.G

No match to: 1467.7052, 1993.0137, 2025.9441, 2070.9729

20. A0A0H0U557 **Mass:** 78971 **Score:** 75 **Expect:** 0.0014 **Queries matched:** 1
A0A0H0U557_BRAHO Polyribonucleotide nucleotidyltransferase OS=Brachyspira hyodysenteriae GN=pnp PE=3 SV=1

Observed	Mr(expt)	Mr(calc)	ppm	Start	End	Miss	Ions	Peptide
987.5048	986.4975	986.5298	-32.67	335 -	343	0	67	R.VHGSALFTR.G

No match to: 1467.7052, 1993.0137, 2025.9441, 2070.9729

21. A0A0H0V372 **Mass:** 78998 **Score:** 75 **Expect:** 0.0014 **Queries matched:** 1
A0A0H0V372_BRAHO Polyribonucleotide nucleotidyltransferase OS=Brachyspira hyodysenteriae GN=pnp PE=3 SV=1

Observed	Mr(expt)	Mr(calc)	ppm	Start	End	Miss	Ions	Peptide
987.5048	986.4975	986.5298	-32.67	335 -	343	0	67	R.VHGSALFTR.G

No match to: 1467.7052, 1993.0137, 2025.9441, 2070.9729

22. A0A0H0TY55 **Mass:** 79015 **Score:** 75 **Expect:** 0.0014 **Queries matched:** 1
A0A0H0TY55_BRAHO Polyribonucleotide nucleotidyltransferase OS=Brachyspira hyodysenteriae GN=pnp PE=3 SV=1

Observed	Mr(expt)	Mr(calc)	ppm	Start	End	Miss	Ions	Peptide
987.5048	986.4975	986.5298	-32.67	335 -	343	0	67	R.VHGSALFTR.G

No match to: 1467.7052, 1993.0137, 2025.9441, 2070.9729

23. J9UKS0 **Mass:** 79013 **Score:** 75 **Expect:** 0.0014 **Queries matched:** 1
J9UKS0_BRAPL Polyribonucleotide nucleotidyltransferase OS=Brachyspira pilosicoli B2904 GN=pnp PE=3 SV=1

Observed	Mr(expt)	Mr(calc)	ppm	Start	End	Miss	Ions	Peptide
987.5048	986.4975	986.5298	-32.67	335 -	343	0	67	R.VHGSALFTR.G

No match to: 1467.7052, 1993.0137, 2025.9441, 2070.9729

24. D8IEN6 **Mass:** 78999 **Score:** 75 **Expect:** 0.0014 **Queries matched:** 1
D8IEN6_BRAP9 Polyribonucleotide nucleotidyltransferase OS=Brachyspira pilosicoli (strain ATCC BAA-1826 / 95/1000) GN=pnp PE=3 SV=1

Observed	Mr(expt)	Mr(calc)	ppm	Start	End	Miss	Ions	Peptide
987.5048	986.4975	986.5298	-32.67	335 -	343	0	67	R.VHGSALFTR.G

No match to: 1467.7052, 1993.0137, 2025.9441, 2070.9729

25. K0JF68 **Mass:** 79013 **Score:** 75 **Expect:** 0.0014 **Queries matched:** 1
K0JF68_BRAPL Polyribonucleotide nucleotidyltransferase OS=Brachyspira pilosicoli WesB GN=pnp PE=3 SV=1

Observed	Mr(expt)	Mr(calc)	ppm	Start	End	Miss	Ions	Peptide
987.5048	986.4975	986.5298	-32.67	335 -	343	0	67	R.VHGSALFTR.G

No match to: 1467.7052, 1993.0137, 2025.9441, 2070.9729

26. L0WY06 **Mass:** 78755 **Score:** 75 **Expect:** 0.0014 **Queries matched:** 1
L0WY06_9SPIR Polyribonucleotide nucleotidyltransferase OS=Brachyspira hampsonii 30446 GN=pnp PE=3 SV=1

Observed	Mr(expt)	Mr(calc)	ppm	Start	End	Miss	Ions	Peptide
987.5048	986.4975	986.5298	-32.67	335 -	343	0	67	R.VHGSALFTR.G

No match to: 1467.7052, 1993.0137, 2025.9441, 2070.9729

27. L8XSI1 **Mass:** 79053 **Score:** 75 **Expect:** 0.0014 **Queries matched:** 1
L8XSI1_9SPIR Polyribonucleotide nucleotidyltransferase OS=Brachyspira hampsonii 30599 GN=pnp PE=3 SV=1

Observed	Mr(expt)	Mr(calc)	ppm	Start	End	Miss	Ions	Peptide

```
987.5048  986.4975  986.5298  -32.67   335 - 343   0   67   R.VHGSALFTR.G
```
No match to: 1467.7052, 1993.0137, 2025.9441, 2070.9729

28. D5U7E8 **Mass:** 79436 **Score:** 75 **Expect:** 0.0014 **Queries matched:** 1

D5U7E8_BRAM5 Polyribonucleotide nucleotidyltransferase OS=Brachyspira murdochii (strain ATCC 51284 / DSM 12563 / 56-150) GN=pnp PE=3 S

Observed	Mr(expt)	Mr(calc)	ppm	Start	End	Miss	Ions	Peptide
987.5048	986.4975	986.5298	-32.67	335 -	343	0	67	R.VHGSALFTR.G

No match to: 1467.7052, 1993.0137, 2025.9441, 2070.9729

29. R5HB49 **Mass:** 7827 **Score:** 42 **Expect:** 2.9 **Queries matched:** 2

R5HB49_9SPIR Uncharacterized protein OS=Brachyspira sp. CAG:484 GN=BN676_01916 PE=4 SV=1

Observed	Mr(expt)	Mr(calc)	ppm	Start	End	Miss	Ions	Peptide
1467.7052	1466.6979	1466.6711	18.3	16 -	28	0	---	R.ALMGEFTDLDEAR.A
2025.9441	2024.9368	2024.9295	3.60	16 -	33	1	---	R.ALMGEFTDLDEARACAQK.S

No match to: 987.5048, 1993.0137, 2070.9729

Search Parameters

```
Type of search         : Sequence Query
Enzyme                 : Trypsin
Fixed modifications    : Carbamidomethyl (C)
Variable modifications : Oxidation (M)
Mass values            : Monoisotopic
Protein Mass           : Unrestricted
Peptide Mass Tolerance : ± 50 ppm
Fragment Mass Tolerance: ± 0.5 Da
Max Missed Cleavages   : 1
Instrument type        : MALDI-TOF-TOF
Query1 (987.5048,1+)   : Locus:17.1.1.211152.56597
Query2 (1467.7052,1+)  : Locus:17.1.1.211152.56596
Query3 (1993.0137,1+)  : Locus:17.1.1.211152.56595
Query4 (2025.9441,1+)  : Locus:17.1.1.211152.56599
Query5 (2070.9729,1+)  : Locus:17.1.1.211152.56598
```

Mascot: http://www.matrixscience.com/

Mascot Search Results

```
User          :
Email         :
Search title  : F:\ROD_inmunoproteoma\MALDI\160223_2016_561_562_ROD\ppw_G4_145631468011.txt
MS data file  : F:\ROD_inmunoproteoma\MALDI\160223_2016_561_562_ROD\ppw_G4_145631468011.txt
Database      : Brachyspiraceae Brachyspiraceae_20160127-151122 (40573 sequences; 13470793 residues)
Timestamp     : 24 Feb 2016 at 12:02:50 GMT
Warning       : A Peptide summary report will usually give a much clearer picture of MS/MS search results.
Top Score     : 479 for D8IDC2, D8IDC2_BRAP9 Uncharacterized protein OS=Brachyspira pilosicoli (strain ATCC BAA-1826 / 95/1000) GN=BP95100
```

Probability Based Mowse Score

Protein score is -10*Log(P), where P is the probability that the observed match is a random event.
Protein scores greater than 59 are significant (p<0.05).
Protein scores are derived from ions scores as a non-probabilistic basis for ranking protein hits.

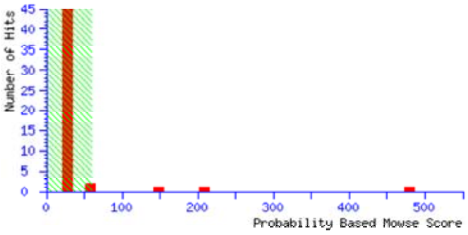

Protein Summary Report

Format As | Protein Summary (deprecated) | Help

Significance threshold p< 0.05 | Max. number of hits AUTO

Standard scoring ◉ MudPIT scoring ○ | Ions score or expect cut-off 0 | Show sub-sets 0

Show pop-ups ◉ Suppress pop-ups ○ | Sort unassigned Decreasing Score | Require bold red ☐

Re-Search All | Search Unmatched

Index

	Accession	Mass	Score	Description
1.	D8IDC2	62081	479	D8IDC2_BRAP9 Uncharacterized protein OS=Brachyspira pilosicoli (strain ATCC BAA-1826 / 95/1000) GN=BP951000_1156
2.	K0JN47	62251	209	K0JN47_BRAPL Putative treponemal membrane protein OS=Brachyspira pilosicoli WesB GN=tmpB PE=4 SV=1
3.	J9UXF7	62380	148	J9UXF7_BRAPL Putative treponemal membrane protein OS=Brachyspira pilosicoli B2904 GN=tmpB PE=4 SV=1
4.	D5UB84	41760	47	D5UB84_BRAM5 Acetyl-CoA acetyltransferase OS=Brachyspira murdochii (strain ATCC 51284 / DSM 12563 / 56-150) GN=B

Results List

1. D8IDC2 **Mass:** 62081 **Score:** 479 **Expect:** 5.1e-44 **Queries matched:** 5
D8IDC2_BRAP9 Uncharacterized protein OS=Brachyspira pilosicoli (strain ATCC BAA-1826 / 95/1000) GN=BP951000_1156 PE=4 SV=1

Observed	Mr(expt)	Mr(calc)	ppm	Start		End	Miss	Ions	Peptide
1240.7073	1239.7000	1239.6823	14.3	523	-	533	0	45	R.LIIPSLNGEER.S
1435.8492	1434.8419	1434.8235	12.8	462	-	473	0	60	K.VTVLPQYYVVVR.R
1931.0648	1930.0575	1930.0384	9.89	506	-	522	1	66	R.NVLRDPNNPDLILPGQR.L
2021.9998	2020.9925	2020.9683	12.0	484	-	499	0	65	R.IAGYSYIYNNPIEWYR.I
2421.1140	2420.1067	2420.0842	9.31	534	-	553	0	160	R.SGDYNPDLEYLTYDEVMQLR.Q

2. K0JN47 **Mass:** 62251 **Score:** 209 **Expect:** 5.1e-17 **Queries matched:** 3
K0JN47_BRAPL Putative treponemal membrane protein OS=Brachyspira pilosicoli WesB GN=tmpB PE=4 SV=1

Observed	Mr(expt)	Mr(calc)	ppm	Start		End	Miss	Ions	Peptide
1240.7073	1239.7000	1239.6823	14.3	523	-	533	0	45	R.LIIPSLNGEER.S
1435.8492	1434.8419	1434.8235	12.8	462	-	473	0	60	K.VTVLPQYYVVVR.R
2021.9998	2020.9925	2020.9683	12.0	484	-	499	0	65	R.IAGYSYIYNNPIEWYR.I

No match to: 1931.0648, 2421.1140

3. J9UXF7 **Mass:** 62380 **Score:** 148 **Expect:** 6.4e-11 **Queries matched:** 2
J9UXF7_BRAPL Putative treponemal membrane protein OS=Brachyspira pilosicoli B2904 GN=tmpB PE=4 SV=1

Observed	Mr(expt)	Mr(calc)	ppm	Start		End	Miss	Ions	Peptide
1435.8492	1434.8419	1434.8235	12.8	464	-	475	0	60	K.VTVLPQYYVVVR.R
2021.9998	2020.9925	2020.9683	12.0	486	-	501	0	65	R.IAGYSYIYNNPIEWYR.I

No match to: 1240.7073, 1931.0648, 2421.1140

4. D5UB84 **Mass:** 41760 **Score:** 47 **Expect:** 0.85 **Queries matched:** 3
D5UB84_BRAM5 Acetyl-CoA acetyltransferase OS=Brachyspira murdochii (strain ATCC 51284 / DSM 12563 / 56-150) GN=Bmur_1875 PE=3 SV=1

Observed	Mr(expt)	Mr(calc)	ppm	Start		End	Miss	Ions	Peptide
1240.7073	1239.7000	1239.6784	17.4	357	-	366	0	---	R.ILTTLLYEMK.K + Oxidation (M)
2021.9998	2020.9925	2021.0108	-9.04	372	-	391	1	---	K.RGLATLCIGGGMGTALVVEM.- + Oxidation (M)
2421.1140	2420.1067	2420.2217	-47.50	237	-	261	1	---	K.KDGTVTAGNASGINDAASALVLMTK.E + Oxidation (M)

No match to: 1435.8492, 1931.0648

Search Parameters

```
Type of search         : Sequence Query
Enzyme                 : Trypsin
Fixed modifications    : Carbamidomethyl (C)
Variable modifications : Oxidation (M)
Mass values            : Monoisotopic
Protein Mass           : Unrestricted
Peptide Mass Tolerance : ± 50 ppm
Fragment Mass Tolerance: ± 0.5 Da
Max Missed Cleavages   : 1
Instrument type        : MALDI-TOF-TOF
Query1 (1240.7073,1+)  : Locus:12.1.1.213511.57295
Query2 (1435.8492,1+)  : Locus:12.1.1.213511.57291
Query3 (1931.0648,1+)  : Locus:12.1.1.213511.57293
Query4 (2021.9998,1+)  : Locus:12.1.1.213511.57294
Query5 (2421.1140,1+)  : Locus:12.1.1.213511.57292
```

**Mascot:** http://www.matrixscience.com/

(MATRIX SCIENCE) Mascot Search Results

```
User          :
Email         :
Search title  : F:\ROD_inmunoproteoma\MALDI\ppw_2016_561_562_563_565\ppw_H1_145467558313.txt
MS data file  : F:\ROD_inmunoproteoma\MALDI\ppw_2016_561_562_563_565\ppw_H1_145467558313.txt
Database      : Brachyspiraceae Brachyspiraceae_20160127-151122 (40573 sequences; 13470793 residues)
Timestamp     : 23 Feb 2016 at 16:36:44 GMT
Warning       : A Peptide summary report will usually give a much clearer picture of MS/MS search results.
Top Score     : 92 for D8IDC2, D8IDC2_BRAP9 Uncharacterized protein OS=Brachyspira pilosicoli (strain ATCC BAA-1826 / 95/1000) GN=BP951000
```

Probability Based Mowse Score

Protein score is -10*Log(P), where P is the probability that the observed match is a random event.
Protein scores greater than 59 are significant (p<0.05).
Protein scores are derived from ions scores as a non-probabilistic basis for ranking protein hits.

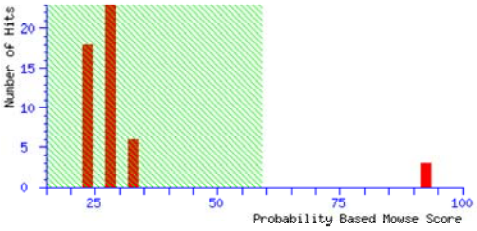

Protein Summary Report

Format As [Protein Summary (deprecated)] Help

Significance threshold p< [0.05] Max. number of hits [AUTO]

Standard scoring ◉ MudPIT scoring ○ Ions score or expect cut-off [0] Show sub-sets [0]

Show pop-ups ◉ Suppress pop-ups ○ Sort unassigned [Decreasing Score] Require bold red ☐

[Re-Search All] [Search Unmatched]

Index

	Accession	Mass	Score	Description
1.	D8IDC2	62081	92	D8IDC2_BRAP9 Uncharacterized protein OS=Brachyspira pilosicoli (strain ATCC BAA-1826 / 95/1000) GN=BP951000_115
2.	K0JN47	62251	92	K0JN47_BRAPL Putative treponemal membrane protein OS=Brachyspira pilosicoli WesB GN=tmpB PE=4 SV=1
3.	J9UXF7	62380	91	J9UXF7_BRAPL Putative treponemal membrane protein OS=Brachyspira pilosicoli B2904 GN=tmpB PE=4 SV=1
4.	A0A0H0WMD3	19626	35	A0A0H0WMD3_BRAHO Uncharacterized protein (Fragment) OS=Brachyspira hyodysenteriae GN=SZ41_00610 PE=4 SV=1

Results List

1. D8IDC2 **Mass:** 62081 **Score:** 92 **Expect:** 2.3e-05 **Queries matched:** 3
D8IDC2_BRAP9 Uncharacterized protein OS=Brachyspira pilosicoli (strain ATCC BAA-1826 / 95/1000) GN=BP951000_1156 PE=4 SV=1

Observed	Mr(expt)	Mr(calc)	ppm	Start - End	Miss	Ions	Peptide
1226.6833	1225.6760	1225.6931	-13.96	474 - 483	1	5	R.RVPLTDALWR.I
1435.8159	1434.8086	1434.8235	-10.37	462 - 473	0	41	K.VTVLPQYYVVVR.R
2021.9493	2020.9420	2020.9683	-12.98	484 - 499	0	8	R.IAGYSYIYNNPIEWYR.I

No match to: 2691.2561, 2748.2759

2. K0JN47 **Mass:** 62251 **Score:** 92 **Expect:** 2.3e-05 **Queries matched:** 3
K0JN47_BRAPL Putative treponemal membrane protein OS=Brachyspira pilosicoli WesB GN=tmpB PE=4 SV=1

Observed	Mr(expt)	Mr(calc)	ppm	Start - End	Miss	Ions	Peptide
1226.6833	1225.6760	1225.6931	-13.96	474 - 483	1	5	R.RVPLTDALWR.I
1435.8159	1434.8086	1434.8235	-10.37	462 - 473	0	41	K.VTVLPQYYVVVR.R
2021.9493	2020.9420	2020.9683	-12.98	484 - 499	0	8	R.IAGYSYIYNNPIEWYR.I

No match to: 2691.2561, 2748.2759

3. J9UXF7 **Mass:** 62380 **Score:** 91 **Expect:** 3.4e-05 **Queries matched:** 3
J9UXF7_BRAPL Putative treponemal membrane protein OS=Brachyspira pilosicoli B2904 GN=tmpB PE=4 SV=1

Observed	Mr(expt)	Mr(calc)	ppm	Start - End	Miss	Ions	Peptide
1226.6833	1225.6760	1225.6931	-13.96	476 - 485	1	5	R.RVPLTDALWR.I
1435.8159	1434.8086	1434.8235	-10.37	464 - 475	0	41	K.VTVLPQYYVVVR.R
2021.9493	2020.9420	2020.9683	-12.98	486 - 501	0	8	R.IAGYSYIYNNPIEWYR.I

No match to: 2691.2561, 2748.2759

4. A0A0H0WMD3 **Mass:** 19626 **Score:** 35 **Expect:** 14 **Queries matched:** 2
A0A0H0WMD3_BRAHO Uncharacterized protein (Fragment) OS=Brachyspira hyodysenteriae GN=SZ41_00610 PE=4 SV=1

Observed	Mr(expt)	Mr(calc)	ppm	Start - End	Miss	Ions	Peptide
1435.8159	1434.8086	1434.7830	17.8	128 - 139	1	---	K.EIITKHPNINEK.Y
2748.2759	2747.2686	2747.3112	-15.48	106 - 127	1	---	K.NYLENEMENIYKQLDNYNSLLK.E

No match to: 1226.6833, 2021.9493, 2691.2561

Search Parameters

Type of search : Sequence Query

```
Enzyme                 : Trypsin
Fixed modifications    : Carbamidomethyl (C)
Variable modifications : Oxidation (M)
Mass values            : Monoisotopic
Protein Mass           : Unrestricted
Peptide Mass Tolerance : ± 50 ppm
Fragment Mass Tolerance: ± 0.5 Da
Max Missed Cleavages   : 1
Instrument type        : MALDI-TOF-TOF
Query1 (1226.6833,1+)  : Locus:1.1.1.212735.57002
Query2 (1435.8159,1+)  : Locus:1.1.1.212735.57001
Query3 (2021.9493,1+)  : Locus:1.1.1.212735.57003
Query4 (2691.2561,1+)  : Locus:1.1.1.212735.56999
Query5 (2748.2759,1+)  : Locus:1.1.1.212735.57000
```

Mascot: http://www.matrixscience.com/

Mascot Search Results

```
User          :
Email         :
Search title  : F:\ROD_inmunoproteoma\MALDI\ppw_2016_561_562_563_565\ppw_H8_145467558920.txt
MS data file  : F:\ROD_inmunoproteoma\MALDI\ppw_2016_561_562_563_565\ppw_H8_145467558920.txt
Database      : Brachyspiraceae Brachyspiraceae_20160127-151122 (40573 sequences; 13470793 residues)
Timestamp     : 23 Feb 2016 at 16:37:02 GMT
Warning       : A Peptide summary report will usually give a much clearer picture of MS/MS search results.
Top Score     : 257 for G0EJY7, G0EJY7_BRAIP Uncharacterized protein OS=Brachyspira intermedia (strain ATCC 51140 / PWS/A) GN=Bint_0627 PE
```

Probability Based Mowse Score

Protein score is -10*Log(P), where P is the probability that the observed match is a random event.
Protein scores greater than 59 are significant (p<0.05).
Protein scores are derived from ions scores as a non-probabilistic basis for ranking protein hits.

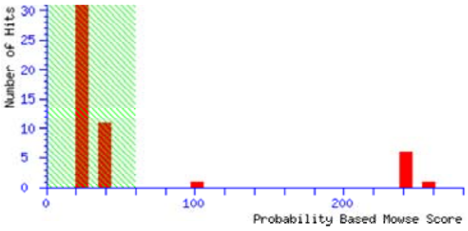

Protein Summary Report

Format As | Protein Summary (deprecated) | Help

Significance threshold p< 0.05 | Max. number of hits AUTO

Standard scoring ◉ MudPIT scoring ○ | Ions score or expect cut-off 0 | Show sub-sets 0

Show pop-ups ◉ Suppress pop-ups ○ | Sort unassigned Decreasing Score | Require bold red ☐

Re-Search All | Search Unmatched

Index

	Accession	Mass	Score	Description
1.	G0EJY7	65366	257	G0EJY7_BRAIP Uncharacterized protein OS=Brachyspira intermedia (strain ATCC 51140 / PWS/A) GN=Bint_0627 PE=4 SV
2.	A0A0H0VSR5	37102	246	A0A0H0VSR5_BRAHO Treponemal membrane protein (Fragment) OS=Brachyspira hyodysenteriae GN=SZ52_08900 PE=4 SV=1
3.	A0A0G4K981	64928	241	A0A0G4K981_9SPIR Treponemal membrane protein OS=Brachyspira suanatina GN=BRSU_1971 PE=4 SV=1
4.	C0R0R7	65924	241	C0R0R7_BRAHW Putative treponemal membrane protein OS=Brachyspira hyodysenteriae (strain ATCC 49526 / WA1) GN=BH
5.	A0A0H0UU11	65881	241	A0A0H0UU11_BRAHO Treponemal membrane protein OS=Brachyspira hyodysenteriae GN=SR30_03260 PE=4 SV=1
6.	L0X712	68181	241	L0X712_9SPIR Uncharacterized protein OS=Brachyspira hampsonii 30446 GN=A966_04185 PE=4 SV=1
7.	L8XQM6	67026	239	L8XQM6_9SPIR Uncharacterized protein OS=Brachyspira hampsonii 30599 GN=H263_04833 PE=4 SV=1
8.	D5U894	67654	96	D5U894_BRAM5 Uncharacterized protein OS=Brachyspira murdochii (strain ATCC 51284 / DSM 12563 / 56-150) GN=Bmur_
9.	L8XTF9	15756	35	L8XTF9_9SPIR Preprotein translocase subunit YajC OS=Brachyspira hampsonii 30599 GN=H263_09136 PE=4 SV=1

Results List

1. G0EJY7 **Mass:** 65366 **Score:** 257 **Expect:** 8.1e-22 **Queries matched:** 3
G0EJY7_BRAIP Uncharacterized protein OS=Brachyspira intermedia (strain ATCC 51140 / PWS/A) GN=Bint_0627 PE=4 SV=1

Observed	Mr(expt)	Mr(calc)	ppm	Start - End	Miss	Ions	Peptide
1116.6403	1115.6330	1115.5822	45.6	299 - 308	1	---	K.AEQEALAKEK.A
1568.9052	1567.8979	1567.8432	34.9	494 - 506	0	86	K.VTVLPMYYTVVQR.T
2366.1431	2365.1358	2365.0606	31.8	566 - 585	0	131	R.AGTYDPNMEYITYDEAMILR.N

No match to: 1520.9022, 2318.1533

2. A0A0H0VSR5 **Mass:** 37102 **Score:** 246 **Expect:** 1e-20 **Queries matched:** 2
A0A0H0VSR5_BRAHO Treponemal membrane protein (Fragment) OS=Brachyspira hyodysenteriae GN=SZ52_08900 PE=4 SV=1

Observed	Mr(expt)	Mr(calc)	ppm	Start - End	Miss	Ions	Peptide
1568.9052	1567.8979	1567.8432	34.9	222 - 234	0	86	K.VTVLPMYYTVVQR.T
2366.1431	2365.1358	2365.0606	31.8	294 - 313	0	131	R.AGTYDPNMEYITYDEAMILR.N

No match to: 1116.6403, 1520.9022, 2318.1533

3. A0A0G4K981 **Mass:** 64928 **Score:** 241 **Expect:** 3.2e-20 **Queries matched:** 2
A0A0G4K981_9SPIR Treponemal membrane protein OS=Brachyspira suanatina GN=BRSU_1971 PE=4 SV=1

Observed	Mr(expt)	Mr(calc)	ppm	Start - End	Miss	Ions	Peptide
1568.9052	1567.8979	1567.8432	34.9	492 - 504	0	86	K.VTVLPMYYTVVQR.T
2366.1431	2365.1358	2365.0606	31.8	564 - 583	0	131	R.AGTYDPNMEYITYDEAMILR.N

No match to: 1116.6403, 1520.9022, 2318.1533

4. C0R0R7 **Mass:** 65924 **Score:** 241 **Expect:** 3.2e-20 **Queries matched:** 2
C0R0R7_BRAHW Putative treponemal membrane protein OS=Brachyspira hyodysenteriae (strain ATCC 49526 / WA1) GN=BHWA1_01226 PE=4 SV=1

Observed	Mr(expt)	Mr(calc)	ppm	Start - End	Miss	Ions	Peptide
1568.9052	1567.8979	1567.8432	34.9	493 - 505	0	86	K.VTVLPMYYTVVQR.T
2366.1431	2365.1358	2365.0606	31.8	565 - 584	0	131	R.AGTYDPNMEYITYDEAMILR.N

No match to: 1116.6403, 1520.9022, 2318.1533

5. A0A0H0UU11 **Mass:** 65881 **Score:** 241 **Expect:** 3.2e-20 **Queries matched:** 2

A0A0H0UU11_BRAHO Treponemal membrane protein OS=Brachyspira hyodysenteriae GN=SR30_03260 PE=4 SV=1

Observed	Mr(expt)	Mr(calc)	ppm	Start	End	Miss	Ions	Peptide
1568.9052	1567.8979	1567.8432	34.9	493 -	505	0	86	K.VTVLPMYYTVVQR.T
2366.1431	2365.1358	2365.0606	31.8	565 -	584	0	131	R.AGTYDPNMEYITYDEAMILR.N

No match to: 1116.6403, 1520.9022, 2318.1533

6. L0X712 **Mass:** 68181 **Score:** 241 **Expect:** 3.2e-20 **Queries matched:** 2

L0X712_9SPIR Uncharacterized protein OS=Brachyspira hampsonii 30446 GN=A966_04185 PE=4 SV=1

Observed	Mr(expt)	Mr(calc)	ppm	Start	End	Miss	Ions	Peptide
1568.9052	1567.8979	1567.8432	34.9	511 -	523	0	86	K.VTVLPMYYTVVQR.T
2366.1431	2365.1358	2365.0606	31.8	583 -	602	0	131	R.AGTYDPNMEYITYDEAMILR.N

No match to: 1116.6403, 1520.9022, 2318.1533

7. L8XQM6 **Mass:** 67026 **Score:** 239 **Expect:** 5.1e-20 **Queries matched:** 2

L8XQM6_9SPIR Uncharacterized protein OS=Brachyspira hampsonii 30599 GN=H263_04833 PE=4 SV=1

Observed	Mr(expt)	Mr(calc)	ppm	Start	End	Miss	Ions	Peptide
1568.9052	1567.8979	1567.8432	34.9	501 -	513	0	86	K.VTVLPMYYTVVQR.T
2366.1431	2365.1358	2365.0606	31.8	573 -	592	0	131	R.AGTYDPNMEYITYDEAMILR.N

No match to: 1116.6403, 1520.9022, 2318.1533

8. D5U894 **Mass:** 67654 **Score:** 96 **Expect:** 1.1e-05 **Queries matched:** 1

D5U894_BRAM5 Uncharacterized protein OS=Brachyspira murdochii (strain ATCC 51284 / DSM 12563 / 56-150) GN=Bmur_0818 PE=4 SV=1

Observed	Mr(expt)	Mr(calc)	ppm	Start	End	Miss	Ions	Peptide
1568.9052	1567.8979	1567.8432	34.9	507 -	519	0	86	K.VTVLPMYYTVVQR.T

No match to: 1116.6403, 1520.9022, 2318.1533, 2366.1431

9. L8XTF9 **Mass:** 15756 **Score:** 35 **Expect:** 13 **Queries matched:** 2

L8XTF9_9SPIR Preprotein translocase subunit YajC OS=Brachyspira hampsonii 30599 GN=H263_09136 PE=4 SV=1

Observed	Mr(expt)	Mr(calc)	ppm	Start	End	Miss	Ions	Peptide
1116.6403	1115.6330	1115.5822	45.5	127 -	135	1	---	K.NQIKEDLEK.A
2318.1533	2317.1460	2317.1909	-19.36	91 -	111	1	---	K.MEIVKSSISAVVTEEMLNPPK.K + Oxidation (M)

No match to: 1520.9022, 1568.9052, 2366.1431

Search Parameters

Type of search : Sequence Query
Enzyme : Trypsin
Fixed modifications : Carbamidomethyl (C)
Variable modifications : Oxidation (M)
Mass values : Monoisotopic
Protein Mass : Unrestricted
Peptide Mass Tolerance : ± 50 ppm
Fragment Mass Tolerance: ± 0.5 Da
Max Missed Cleavages : 1
Instrument type : MALDI-TOF-TOF
Query1 (1116.6403,1+) : Locus:8.1.1.212742.57036
Query2 (1520.9022,1+) : Locus:8.1.1.212742.57037
Query3 (1568.9052,1+) : Locus:8.1.1.212742.57034
Query4 (2318.1533,1+) : Locus:8.1.1.212742.57038
Query5 (2366.1431,1+) : Locus:8.1.1.212742.57035

Mascot: http://www.matrixscience.com/

Mascot Search Results

User	: VANESSA CASAS
Email	: vanessa.casas.csic@uab.cat
Search title	: Project: _SERVEI, Spot Set: _SERVEI\160127_2016_562_563_ROD, Label: I3, Spot Id: 211167, Peak List Id: 56492, MS Job Run Id: 13133
Database	: NCBIprot 20160920 (95963614 sequences; 35367425320 residues)
Taxonomy	: Other Bacteria (8703853 sequences)
Timestamp	: 3 Nov 2016 at 14:01:32 GMT
Warning	: A Peptide summary report will usually give a much clearer picture of MS/MS search results.
Top Score	: 180 for WP_047102140.1, hypothetical protein [Brachyspira hyodysenteriae]

Mascot Score Histogram

Protein score is -10*Log(P), where P is the probability that the observed match is a random event.
Protein scores greater than 82 are significant (p<0.05).
Protein scores are derived from ions scores as a non-probabilistic basis for ranking protein hits.

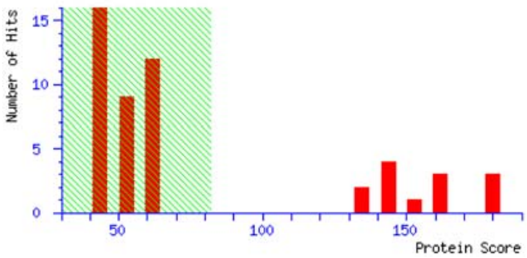

Protein Summary Report

Format As | Protein Summary (deprecated) | Help

Significance threshold p< 0.05 | Max. number of hits AUTO

Preferred taxonomy | All entries

Re-Search All | Search Unmatched

Index

	Accession	Mass	Score	Description
1.	WP_047102140.1	63915	180	hypothetical protein [Brachyspira hyodysenteriae]
2.	WP_020064491.1	65881	179	hypothetical protein [Brachyspira hyodysenteriae]
3.	WP_012670751.1	65924	179	hypothetical protein [Brachyspira hyodysenteriae]
4.	CRF34363.1	64928	160	treponemal membrane protein [Brachyspira suanatina]
5.	WP_048595182.1	65059	160	hypothetical protein [Brachyspira suanatina]
6.	WP_014487102.1	65366	159	hypothetical protein [Brachyspira intermedia]
7.	KLI41108.1	37102	156	treponemal membrane protein, partial [Brachyspira hyodysenteriae]
8.	WP_051087725.1	67971	147	hypothetical protein [Brachyspira innocens]
9.	ELV06323.1	67026	141	hypothetical protein H263_04833 [Brachyspira hampsonii 30599]
10.	EKV57758.1	68181	140	hypothetical protein A966_04185 [Brachyspira hampsonii 30446]
11.	WP_039955452.1	68312	140	hypothetical protein [Brachyspira hampsonii]
12.	ADG70917.1	67654	136	conserved hypothetical protein [Brachyspira murdochii DSM 12563]
13.	WP_041749978.1	67785	136	hypothetical protein [Brachyspira murdochii]
14.	ACN83535.1	52760	63	putative outer membrane protein (tmpB) [Brachyspira hyodysenteriae WA1]

Results List

1. WP_047102140.1 **Mass:** 63915 **Score:** 180 **Expect:** 8.7e-12 **Matches:** 10
hypothetical protein [Brachyspira hyodysenteriae]

Observed	Mr(expt)	Mr(calc)	ppm	Start - End	Miss	Ions	Peptide
1072.5177	1071.5104	1071.5713	-56.80	485 - 493	0	---	R.TPLTDALWR.I
1072.5177	1071.5104	1071.5713	-56.80	485 - 493	0	48	R.TPLTDALWR.I
1460.7374	1459.7301	1459.7267	2.37	191 - 205	1	---	K.AGDNNDKAVSGALTK.A
1568.7583	1567.7510	1567.8432	-58.82	472 - 484	0	---	K.VTVLPMYYTVVQR.T
1568.7583	1567.7510	1567.8432	-58.82	472 - 484	0	83	K.VTVLPMYYTVVQR.T
1584.7546	1583.7473	1583.8064	-37.32	62 - 74	1	---	K.AQMALFYRLMNAR.I
1646.7163	1645.7090	1645.7729	-38.84	346 - 360	0	---	K.ALEASQAMDDLVNNR.D
2371.9314	2370.9241	2371.0564	-55.79	32 - 51	0	---	R.ITQEADDLQNQGQYQQSYDK.S
2379.9409	2378.9336	2379.0761	-59.87	80 - 101	0	---	K.NDANNAITEINQMGAATDNQFK.A
3116.4670	3115.4597	3115.5972	-44.11	516 - 543	1	---	R.DILRDPNNPDLILPGQVLTIPSMNGEER.A

No match to: 1037.4690, 1084.5382, 1101.5303, 1104.4912, 1116.5339, 1118.5109, 1130.5094, 1136.5675, 1212.6610, 1282.6490, 1320.5104, 1360.5771, 1490.6385, 1520.7601, 1580.8514, 1618.7909, 1638.7607, 1788.7609, 1788.7609, 1800.7844, 1817.7853, 1838.8033, 1916.9016, 1944.8574, 1987.8623, 2005.8568, 2005.8568, 2017.8933, 2021.8773, 2033.9769, 2057.9063, 2072.9871, 2135.9636, 2210.9722, 2223.9563, 2284.9172, 2338.9873, 2365.9209, 2365.9209, 2383.9265, 2604.0366, 2691.1211, 2720.1304, 2748.1340

2. WP_020064491.1 **Mass:** 65881 **Score:** 179 **Expect:** 1.1e-11 **Matches:** 10
hypothetical protein [Brachyspira hyodysenteriae]

Observed	Mr(expt)	Mr(calc)	ppm	Start - End	Miss	Ions	Peptide
1072.5177	1071.5104	1071.5713	-56.80	506 - 514	0	---	R.TPLTDALWR.I
1072.5177	1071.5104	1071.5713	-56.80	506 - 514	0	48	R.TPLTDALWR.I
1460.7374	1459.7301	1459.7267	2.37	191 - 205	1	---	K.AGDNNDKAVSGALTK.A
1568.7583	1567.7510	1567.8432	-58.82	493 - 505	0	---	K.VTVLPMYYTVVQR.T
1568.7583	1567.7510	1567.8432	-58.82	493 - 505	0	83	K.VTVLPMYYTVVQR.T
1584.7546	1583.7473	1583.8064	-37.32	62 - 74	1	---	K.AQMALFYRLMNAR.I
1646.7163	1645.7090	1645.7729	-38.84	367 - 381	0	---	K.ALEASQAMDDLVNNR.D

2371.9314	2370.9241	2371.0564	-55.79	32 -	51	0	---	R.ITQEADDLQNQGQYQQSYDK.S
2379.9409	2378.9336	2379.0761	-59.87	80 -	101	0	---	K.NDANNAITEINQMGAATDNQFK.A
3116.4670	3115.4597	3115.5972	-44.11	537 -	564	1	---	R.DILRDPNNPDLILPGQVLTIPSMNGEER.A

No match to: 1037.4690, 1084.5382, 1101.5303, 1104.4912, 1116.5339, 1118.5109, 1130.5094, 1136.5675, 1212.6610, 1282.6490, 1320.5104, 1360.5771, 1490.6385, 1520.7601, 1580.8514, 1618.7909, 1638.7607, 1788.7609, 1788.7609, 1800.7844, 1817.7853, 1838.8033, 1916.9016, 1944.8574, 1987.8623, 2005.8568, 2005.8568, 2017.8933, 2021.8773, 2033.9769, 2057.9063, 2072.9871, 2135.9636, 2210.9722, 2223.9563, 2284.9172, 2338.9873, 2365.9209, 2365.9209, 2383.9265, 2604.0366, 2691.1211, 2720.1304, 2748.1340

3. WP_012670751.1 **Mass:** 65924 **Score:** 179 **Expect:** 1.1e-11 **Matches:** 10

hypothetical protein [Brachyspira hyodysenteriae]

Observed	Mr(expt)	Mr(calc)	ppm	Start	End	Miss	Ions	Peptide
1072.5177	1071.5104	1071.5713	-56.80	506 -	514	0	---	R.TPLTDALWR.I
1072.5177	1071.5104	1071.5713	-56.80	506 -	514	0	48	R.TPLTDALWR.I
1460.7374	1459.7301	1459.7267	2.37	191 -	205	1	---	K.AGDNNDKAVSGALTK.A
1568.7583	1567.7510	1567.8432	-58.82	493 -	505	0	---	K.VTVLPMYYTVVQR.T
1568.7583	1567.7510	1567.8432	-58.82	493 -	505	0	83	K.VTVLPMYYTVVQR.T
1584.7546	1583.7473	1583.8064	-37.32	62 -	74	1	---	K.AQMALFYRLMNAR.I
1646.7163	1645.7090	1645.7729	-38.84	367 -	381	0	---	K.ALEASQAMDDLVNNR.D
2371.9314	2370.9241	2371.0564	-55.79	32 -	51	0	---	R.ITQEADDLQNQGQYQQSYDK.S
2379.9409	2378.9336	2379.0761	-59.87	80 -	101	0	---	K.NDANNAITEINQMGAATDNQFK.A
3116.4670	3115.4597	3115.5972	-44.11	537 -	564	1	---	R.DILRDPNNPDLILPGQVLTIPSMNGEER.A

No match to: 1037.4690, 1084.5382, 1101.5303, 1104.4912, 1116.5339, 1118.5109, 1130.5094, 1136.5675, 1212.6610, 1282.6490, 1320.5104, 1360.5771, 1490.6385, 1520.7601, 1580.8514, 1618.7909, 1638.7607, 1788.7609, 1788.7609, 1800.7844, 1817.7853, 1838.8033, 1916.9016, 1944.8574, 1987.8623, 2005.8568, 2005.8568, 2017.8933, 2021.8773, 2033.9769, 2057.9063, 2072.9871, 2135.9636, 2210.9722, 2223.9563, 2284.9172, 2338.9873, 2365.9209, 2365.9209, 2383.9265, 2604.0366, 2691.1211, 2720.1304, 2748.1340

4. CRF34363.1 **Mass:** 64928 **Score:** 160 **Expect:** 8.7e-10 **Matches:** 8

treponemal membrane protein [Brachyspira suanatina]

Observed	Mr(expt)	Mr(calc)	ppm	Start	End	Miss	Ions	Peptide
1072.5177	1071.5104	1071.5713	-56.80	505 -	513	0	---	R.TPLTDALWR.I
1072.5177	1071.5104	1071.5713	-56.80	505 -	513	0	48	R.TPLTDALWR.I
1568.7583	1567.7510	1567.8432	-58.82	492 -	504	0	---	K.VTVLPMYYTVVQR.T
1568.7583	1567.7510	1567.8432	-58.82	492 -	504	0	83	K.VTVLPMYYTVVQR.T
1584.7546	1583.7473	1583.8064	-37.32	61 -	73	1	---	K.AQMALFYRLMNAR.I
1646.7163	1645.7090	1645.7729	-38.84	366 -	380	0	---	K.ALEASQAMDDLVNNR.D
2371.9314	2370.9241	2371.0564	-55.79	31 -	50	0	---	R.ITQEADDLQNQGQYQQSYDK.S
2379.9409	2378.9336	2379.0761	-59.87	79 -	100	0	---	K.NDANNAITEINQMGAATDNQFK.T

No match to: 1037.4690, 1084.5382, 1101.5303, 1104.4912, 1116.5339, 1118.5109, 1130.5094, 1136.5675, 1212.6610, 1282.6490, 1320.5104, 1360.5771, 1460.7374, 1490.6385, 1520.7601, 1580.8514, 1618.7909, 1638.7607, 1788.7609, 1788.7609, 1800.7844, 1817.7853, 1838.8033, 1916.9016, 1944.8574, 1987.8623, 2005.8568, 2005.8568, 2017.8933, 2021.8773, 2033.9769, 2057.9063, 2072.9871, 2135.9636, 2210.9722, 2223.9563, 2284.9172, 2338.9873, 2365.9209, 2365.9209, 2383.9265, 2604.0366, 2691.1211, 2720.1304, 2748.1340, 3116.4670

5. WP_048595182.1 **Mass:** 65059 **Score:** 160 **Expect:** 8.7e-10 **Matches:** 8

hypothetical protein [Brachyspira suanatina]

Observed	Mr(expt)	Mr(calc)	ppm	Start	End	Miss	Ions	Peptide
1072.5177	1071.5104	1071.5713	-56.80	506 -	514	0	---	R.TPLTDALWR.I
1072.5177	1071.5104	1071.5713	-56.80	506 -	514	0	48	R.TPLTDALWR.I
1568.7583	1567.7510	1567.8432	-58.82	493 -	505	0	---	K.VTVLPMYYTVVQR.T
1568.7583	1567.7510	1567.8432	-58.82	493 -	505	0	83	K.VTVLPMYYTVVQR.T
1584.7546	1583.7473	1583.8064	-37.32	62 -	74	1	---	K.AQMALFYRLMNAR.I
1646.7163	1645.7090	1645.7729	-38.84	367 -	381	0	---	K.ALEASQAMDDLVNNR.D
2371.9314	2370.9241	2371.0564	-55.79	32 -	51	0	---	R.ITQEADDLQNQGQYQQSYDK.S
2379.9409	2378.9336	2379.0761	-59.87	80 -	101	0	---	K.NDANNAITEINQMGAATDNQFK.T

No match to: 1037.4690, 1084.5382, 1101.5303, 1104.4912, 1116.5339, 1118.5109, 1130.5094, 1136.5675, 1212.6610, 1282.6490, 1320.5104, 1360.5771, 1460.7374, 1490.6385, 1520.7601, 1580.8514, 1618.7909, 1638.7607, 1788.7609, 1788.7609, 1800.7844, 1817.7853, 1838.8033, 1916.9016, 1944.8574, 1987.8623, 2005.8568, 2005.8568, 2017.8933, 2021.8773, 2033.9769, 2057.9063, 2072.9871, 2135.9636, 2210.9722, 2223.9563, 2284.9172, 2338.9873, 2365.9209, 2365.9209, 2383.9265, 2604.0366, 2691.1211, 2720.1304, 2748.1340, 3116.4670

6. WP_014487102.1 **Mass:** 65366 **Score:** 159 **Expect:** 1.1e-09 **Matches:** 8

hypothetical protein [Brachyspira intermedia]

Observed	Mr(expt)	Mr(calc)	ppm	Start	End	Miss	Ions	Peptide
1072.5177	1071.5104	1071.5713	-56.80	507 -	515	0	---	R.TPLTDALWR.I
1072.5177	1071.5104	1071.5713	-56.80	507 -	515	0	48	R.TPLTDALWR.I
1116.5339	1115.5266	1115.5822	-49.82	299 -	308	1	---	K.AEQEALAKEK.A
1568.7583	1567.7510	1567.8432	-58.82	494 -	506	0	---	K.VTVLPMYYTVVQR.T
1568.7583	1567.7510	1567.8432	-58.82	494 -	506	0	83	K.VTVLPMYYTVVQR.T
1584.7546	1583.7473	1583.8064	-37.32	62 -	74	1	---	K.AQMALFYRLMNAR.I
2371.9314	2370.9241	2371.0564	-55.79	32 -	51	0	---	R.ITQEADDLQNQGQYQQSYDK.S
2379.9409	2378.9336	2379.0761	-59.87	80 -	101	0	---	K.NDANNAITEINQMGAATDNQFK.T

No match to: 1037.4690, 1084.5382, 1101.5303, 1104.4912, 1118.5109, 1130.5094, 1136.5675, 1212.6610, 1282.6490, 1320.5104, 1360.5771, 1460.7374, 1490.6385, 1520.7601, 1580.8514, 1618.7909, 1638.7607, 1646.7163, 1788.7609, 1788.7609, 1800.7844, 1817.7853, 1838.8033, 1916.9016, 1944.8574, 1987.8623, 2005.8568, 2005.8568, 2017.8933, 2021.8773, 2033.9769, 2057.9063, 2072.9871, 2135.9636, 2210.9722, 2223.9563, 2284.9172, 2338.9873, 2365.9209, 2365.9209, 2383.9265, 2604.0366, 2691.1211, 2720.1304, 2748.1340, 3116.4670

7. KLI41108.1 **Mass:** 37102 **Score:** 156 **Expect:** 2.2e-09 **Matches:** 7

treponemal membrane protein, partial [Brachyspira hyodysenteriae]

Observed	Mr(expt)	Mr(calc)	ppm	Start	End	Miss	Ions	Peptide
1072.5177	1071.5104	1071.5713	-56.80	235 -	243	0	---	R.TPLTDALWR.I
1072.5177	1071.5104	1071.5713	-56.80	235 -	243	0	48	R.TPLTDALWR.I
1568.7583	1567.7510	1567.8432	-58.82	222 -	234	0	---	K.VTVLPMYYTVVQR.T
1568.7583	1567.7510	1567.8432	-58.82	222 -	234	0	83	K.VTVLPMYYTVVQR.T
1584.7546	1583.7473	1583.8382	-57.35	222 -	234	0	---	K.VTVLPMYYTVVQR.T + Oxidation (M)
1646.7163	1645.7090	1645.7729	-38.84	96 -	110	0	---	K.ALEASQAMDDLVNNR.D
3116.4670	3115.4597	3115.5972	-44.11	266 -	293	1	---	R.DILRDPNNPDLILPGQVLTIPSMNGEER.A

No match to: 1037.4690, 1084.5382, 1101.5303, 1104.4912, 1116.5339, 1118.5109, 1130.5094, 1136.5675, 1212.6610, 1282.6490, 1320.5104, 1360.5771, 1460.7374, 1490.6385, 1520.7601, 1580.8514, 1618.7909, 1638.7607, 1788.7609, 1788.7609, 1800.7844, 1817.7853, 1838.8033, 1916.9016, 1944.8574, 1987.8623, 2005.8568, 2005.8568, 2017.8933, 2021.8773, 2033.9769, 2057.9063, 2072.9871, 2135.9636, 2210.9722, 2223.9563, 2284.9172, 2338.9873, 2365.9209, 2365.9209, 2371.9314, 2379.9409, 2383.9265, 2604.0366, 2691.1211, 2720.1304, 2748.1340

8. WP_051087725.1 **Mass:** 67971 **Score:** 147 **Expect:** 1.7e-08 **Matches:** 7

hypothetical protein [Brachyspira innocens]

Observed	Mr(expt)	Mr(calc)	ppm	Start	End	Miss	Ions	Peptide
1072.5177	1071.5104	1071.5713	-56.80	521	529	0	---	R.TPLTDALWR.I
1072.5177	1071.5104	1071.5713	-56.80	521	529	0	48	R.TPLTDALWR.I
1460.7374	1459.7301	1459.7267	2.37	191	205	1	---	K.AGDNNDKTVSGALAK.A
1568.7583	1567.7510	1567.8432	-58.82	508	520	0	---	K.VTVLPMYYTVVQR.T
1568.7583	1567.7510	1567.8432	-58.82	508	520	0	83	K.VTVLPMYYTVVQR.T
1584.7546	1583.7473	1583.8382	-57.35	508	520	0	---	K.VTVLPMYYTVVQR.T + Oxidation (M)
3116.4670	3115.4597	3115.6084	-47.72	552	579	1	---	R.NVLRDPNNPDLILPGQVLTIPSMNGEQR.A + Oxidation (M)

No match to: 1037.4690, 1084.5382, 1101.5303, 1104.4912, 1116.5339, 1118.5109, 1130.5094, 1136.5675, 1212.6610, 1282.6490, 1320.5104, 1360.5771, 1490.6385, 1520.7601, 1580.8514, 1618.7909, 1638.7607, 1646.7163, 1788.7609, 1788.7609, 1800.7844, 1817.7853, 1838.8033, 1916.9016, 1944.8574, 1987.8623, 2005.8568, 2005.8568, 2017.8933, 2021.8773, 2033.9769, 2057.9063, 2072.9871, 2135.9636, 2210.9722, 2223.9563, 2284.9172, 2338.9873, 2365.9209, 2365.9209, 2371.9314, 2379.9409, 2383.9265, 2604.0366, 2691.1211, 2720.1304, 2748.1340

9. ELV06323.1 **Mass:** 67026 **Score:** 141 **Expect:** 6.9e-08 **Matches:** 6

hypothetical protein H263_04833 [Brachyspira hampsonii 30599]

Observed	Mr(expt)	Mr(calc)	ppm	Start	End	Miss	Ions	Peptide
1072.5177	1071.5104	1071.5713	-56.80	514	522	0	---	R.TPLTDALWR.I
1072.5177	1071.5104	1071.5713	-56.80	514	522	0	48	R.TPLTDALWR.I
1568.7583	1567.7510	1567.8432	-58.82	501	513	0	---	K.VTVLPMYYTVVQR.T
1568.7583	1567.7510	1567.8432	-58.82	501	513	0	83	K.VTVLPMYYTVVQR.T
1584.7546	1583.7473	1583.8382	-57.35	501	513	0	---	K.VTVLPMYYTVVQR.T + Oxidation (M)
2371.9314	2370.9241	2371.0564	-55.79	31	50	0	---	R.ITQEADDLQNQGQYQQSYDK.S

No match to: 1037.4690, 1084.5382, 1101.5303, 1104.4912, 1116.5339, 1118.5109, 1130.5094, 1136.5675, 1212.6610, 1282.6490, 1320.5104, 1360.5771, 1460.7374, 1490.6385, 1520.7601, 1580.8514, 1618.7909, 1638.7607, 1646.7163, 1788.7609, 1788.7609, 1800.7844, 1817.7853, 1838.8033, 1916.9016, 1944.8574, 1987.8623, 2005.8568, 2005.8568, 2017.8933, 2021.8773, 2033.9769, 2057.9063, 2072.9871, 2135.9636, 2210.9722, 2223.9563, 2284.9172, 2338.9873, 2365.9209, 2365.9209, 2379.9409, 2383.9265, 2604.0366, 2691.1211, 2720.1304, 2748.1340, 3116.4670

10. EKV57758.1 **Mass:** 68181 **Score:** 140 **Expect:** 8.7e-08 **Matches:** 6

hypothetical protein A966_04185 [Brachyspira hampsonii 30446]

Observed	Mr(expt)	Mr(calc)	ppm	Start	End	Miss	Ions	Peptide
1072.5177	1071.5104	1071.5713	-56.80	524	532	0	---	R.TPLTDALWR.I
1072.5177	1071.5104	1071.5713	-56.80	524	532	0	48	R.TPLTDALWR.I
1568.7583	1567.7510	1567.8432	-58.82	511	523	0	---	K.VTVLPMYYTVVQR.T
1568.7583	1567.7510	1567.8432	-58.82	511	523	0	83	K.VTVLPMYYTVVQR.T
1584.7546	1583.7473	1583.8382	-57.35	511	523	0	---	K.VTVLPMYYTVVQR.T + Oxidation (M)
2371.9314	2370.9241	2371.0564	-55.79	31	50	0	---	R.ITQEADDLQNQGQYQQSYDK.S

No match to: 1037.4690, 1084.5382, 1101.5303, 1104.4912, 1116.5339, 1118.5109, 1130.5094, 1136.5675, 1212.6610, 1282.6490, 1320.5104, 1360.5771, 1460.7374, 1490.6385, 1520.7601, 1580.8514, 1618.7909, 1638.7607, 1646.7163, 1788.7609, 1788.7609, 1800.7844, 1817.7853, 1838.8033, 1916.9016, 1944.8574, 1987.8623, 2005.8568, 2005.8568, 2017.8933, 2021.8773, 2033.9769, 2057.9063, 2072.9871, 2135.9636, 2210.9722, 2223.9563, 2284.9172, 2338.9873, 2365.9209, 2365.9209, 2379.9409, 2383.9265, 2604.0366, 2691.1211, 2720.1304, 2748.1340, 3116.4670

11. WP_039955452.1 **Mass:** 68312 **Score:** 140 **Expect:** 8.7e-08 **Matches:** 6

hypothetical protein [Brachyspira hampsonii]

Observed	Mr(expt)	Mr(calc)	ppm	Start	End	Miss	Ions	Peptide
1072.5177	1071.5104	1071.5713	-56.80	525	533	0	---	R.TPLTDALWR.I
1072.5177	1071.5104	1071.5713	-56.80	525	533	0	48	R.TPLTDALWR.I
1568.7583	1567.7510	1567.8432	-58.82	512	524	0	---	K.VTVLPMYYTVVQR.T
1568.7583	1567.7510	1567.8432	-58.82	512	524	0	83	K.VTVLPMYYTVVQR.T
1584.7546	1583.7473	1583.8382	-57.35	512	524	0	---	K.VTVLPMYYTVVQR.T + Oxidation (M)
2371.9314	2370.9241	2371.0564	-55.79	32	51	0	---	R.ITQEADDLQNQGQYQQSYDK.S

No match to: 1037.4690, 1084.5382, 1101.5303, 1104.4912, 1116.5339, 1118.5109, 1130.5094, 1136.5675, 1212.6610, 1282.6490, 1320.5104, 1360.5771, 1460.7374, 1490.6385, 1520.7601, 1580.8514, 1618.7909, 1638.7607, 1646.7163, 1788.7609, 1788.7609, 1800.7844, 1817.7853, 1838.8033, 1916.9016, 1944.8574, 1987.8623, 2005.8568, 2005.8568, 2017.8933, 2021.8773, 2033.9769, 2057.9063, 2072.9871, 2135.9636, 2210.9722, 2223.9563, 2284.9172, 2338.9873, 2365.9209, 2365.9209, 2379.9409, 2383.9265, 2604.0366, 2691.1211, 2720.1304, 2748.1340, 3116.4670

12. ADG70917.1 **Mass:** 67654 **Score:** 136 **Expect:** 2.2e-07 **Matches:** 5

conserved hypothetical protein [Brachyspira murdochii DSM 12563]

Observed	Mr(expt)	Mr(calc)	ppm	Start	End	Miss	Ions	Peptide
1072.5177	1071.5104	1071.5713	-56.80	520	528	0	---	R.TPLTDALWR.I
1072.5177	1071.5104	1071.5713	-56.80	520	528	0	48	R.TPLTDALWR.I
1568.7583	1567.7510	1567.8432	-58.82	507	519	0	---	K.VTVLPMYYTVVQR.T
1568.7583	1567.7510	1567.8432	-58.82	507	519	0	83	K.VTVLPMYYTVVQR.T
1584.7546	1583.7473	1583.8382	-57.35	507	519	0	---	K.VTVLPMYYTVVQR.T + Oxidation (M)

No match to: 1037.4690, 1084.5382, 1101.5303, 1104.4912, 1116.5339, 1118.5109, 1130.5094, 1136.5675, 1212.6610, 1282.6490, 1320.5104, 1360.5771, 1460.7374, 1490.6385, 1520.7601, 1580.8514, 1618.7909, 1638.7607, 1646.7163, 1788.7609, 1788.7609, 1800.7844, 1817.7853, 1838.8033, 1916.9016, 1944.8574, 1987.8623, 2005.8568, 2005.8568, 2017.8933, 2021.8773, 2033.9769, 2057.9063, 2072.9871, 2135.9636, 2210.9722, 2223.9563, 2284.9172, 2338.9873, 2365.9209, 2365.9209, 2371.9314, 2379.9409, 2383.9265, 2604.0366, 2691.1211, 2720.1304, 2748.1340, 3116.4670

13. WP_041749978.1 **Mass:** 67785 **Score:** 136 **Expect:** 2.2e-07 **Matches:** 5

hypothetical protein [Brachyspira murdochii]

Observed	Mr(expt)	Mr(calc)	ppm	Start	End	Miss	Ions	Peptide
1072.5177	1071.5104	1071.5713	-56.80	521	529	0	---	R.TPLTDALWR.I
1072.5177	1071.5104	1071.5713	-56.80	521	529	0	48	R.TPLTDALWR.I
1568.7583	1567.7510	1567.8432	-58.82	508	520	0	---	K.VTVLPMYYTVVQR.T
1568.7583	1567.7510	1567.8432	-58.82	508	520	0	83	K.VTVLPMYYTVVQR.T
1584.7546	1583.7473	1583.8382	-57.35	508	520	0	---	K.VTVLPMYYTVVQR.T + Oxidation (M)

No match to: 1037.4690, 1084.5382, 1101.5303, 1104.4912, 1116.5339, 1118.5109, 1130.5094, 1136.5675, 1212.6610, 1282.6490, 1320.5104, 1360.5771, 1460.7374, 1490.6385, 1520.7601, 1580.8514, 1618.7909, 1638.7607, 1646.7163, 1788.7609, 1788.7609, 1800.7844, 1817.7853, 1838.8033, 1916.9016, 1944.8574, 1987.8623, 2005.8568, 2005.8568, 2017.8933, 2021.8773, 2033.9769, 2057.9063, 2072.9871, 2135.9636, 2210.9722, 2223.9563, 2284.9172, 2338.9873, 2365.9209, 2365.9209, 2371.9314, 2379.9409, 2383.9265, 2604.0366, 2691.1211, 2720.1304, 2748.1340, 3116.4670

14. ACN83535.1 **Mass:** 52760 **Score:** 63 **Expect:** 4.7 **Matches:** 9

putative outer membrane protein (tmpB) [Brachyspira hyodysenteriae WA1]

Observed	Mr(expt)	Mr(calc)	ppm	Start	End	Miss	Ions	Peptide
1118.5109	1117.5036	1117.5628	-52.98	302	310	1	---	R.RANTDSLWR.I

1136.5675	1135.5602	1135.6237	-55.88	60 - 68	1	---	K.EYSDKIILR.Y
1212.6610	1211.6537	1211.7238	-57.79	347 - 357	1	---	R.LVIPSLKGETR.Q
1490.6385	1489.6312	1489.7201	-59.67	69 - 80	0	---	R.YGVYEYVLNSQR.D
1944.8574	1943.8501	1943.9642	-58.68	311 - 326	1	---	R.IASYDFIYGNGNLWRR.I
2135.9636	2134.9563	2135.0754	-55.78	196 - 214	1	---	K.SREAMNILDMLEAPLAYAK.A
2284.9172	2283.9099	2284.0066	-42.32	37 - 56	0	---	R.EMAIDAHSVGDYNQSIEYSR.Q
2338.9873	2337.9800	2338.1111	-56.04	241 - 262	0	---	K.SLSSAGETLIANNYTDSLMHSK.N
2604.0366	2603.0293	2603.1710	-54.43	35 - 56	1	---	R.YREMAIDAHSVGDYNQSIEYSR.Q

No match to: 1037.4690, 1072.5177, 1072.5177, 1084.5382, 1101.5303, 1104.4912, 1116.5339, 1130.5094, 1282.6490, 1320.5104, 1360.5771, 1460.7374, 1520.7601, 1568.7583, 1568.7583, 1580.8514, 1584.7546, 1618.7909, 1638.7607, 1646.7163, 1788.7609, 1788.7609, 1800.7844, 1817.7853, 1838.8033, 1916.9016, 1987.8623, 2005.8568, 2005.8568, 2017.8933, 2021.8773, 2033.9769, 2057.9063, 2072.9871, 2210.9722, 2223.9563, 2365.9209, 2365.9209, 2371.9314, 2379.9409, 2383.9265, 2691.1211, 2720.1304, 2748.1340, 3116.4670

Search Parameters

Type of search : Sequence Query
Enzyme : Trypsin
Fixed modifications : Carbamidomethyl (C)
Variable modifications : Oxidation (M)
Mass values : Monoisotopic
Protein Mass : Unrestricted
Peptide Mass Tolerance : ± 60 ppm
Fragment Mass Tolerance: ± 0.6 Da
Max Missed Cleavages : 1
Instrument type : MALDI-TOF-TOF
Query1 (1037.4690,1+): <no title>
Query2 (1072.5177,1+): <no title>
Query3 (1072.5177,1+): Label: I3, Spot_Id: 211167, Peak_List_Id: 56614, MSMS Job_Run_Id: 13135, Comment:
Query4 (1084.5382,1+): <no title>
Query5 (1101.5303,1+): <no title>
Query6 (1104.4912,1+): <no title>
Query7 (1116.5339,1+): <no title>
Query8 (1118.5109,1+): <no title>
Query9 (1130.5094,1+): <no title>
Query10 (1136.5675,1+): <no title>
Query11 (1212.6610,1+): <no title>
Query12 (1282.6490,1+): <no title>
Query13 (1320.5104,1+): <no title>
Query14 (1360.5771,1+): <no title>
Query15 (1460.7374,1+): <no title>
Query16 (1490.6385,1+): <no title>
Query17 (1520.7601,1+): <no title>
Query18 (1568.7583,1+): <no title>
Query19 (1568.7583,1+): Label: I3, Spot_Id: 211167, Peak_List_Id: 56610, MSMS Job_Run_Id: 13135, Comment:
Query20 (1580.8514,1+): <no title>
Query21 (1584.7546,1+): <no title>
Query22 (1618.7909,1+): <no title>
Query23 (1638.7607,1+): <no title>
Query24 (1646.7163,1+): <no title>
Query25 (1788.7609,1+): <no title>
Query26 (1788.7609,1+): Label: I3, Spot_Id: 211167, Peak_List_Id: 56613, MSMS Job_Run_Id: 13135, Comment:
Query27 (1800.7844,1+): <no title>
Query28 (1817.7853,1+): <no title>
Query29 (1838.8033,1+): <no title>
Query30 (1916.9016,1+): <no title>
Query31 (1944.8574,1+): <no title>
Query32 (1987.8623,1+): <no title>
Query33 (2005.8568,1+): <no title>
Query34 (2005.8568,1+): Label: I3, Spot_Id: 211167, Peak_List_Id: 56611, MSMS Job_Run_Id: 13135, Comment:
Query35 (2017.8933,1+): <no title>
Query36 (2021.8773,1+): <no title>
Query37 (2033.9769,1+): <no title>
Query38 (2057.9063,1+): <no title>
Query39 (2072.9871,1+): <no title>
Query40 (2135.9636,1+): <no title>
Query41 (2210.9722,1+): <no title>
Query42 (2223.9563,1+): <no title>
Query43 (2284.9172,1+): <no title>
Query44 (2338.9873,1+): <no title>
Query45 (2365.9209,1+): <no title>
Query46 (2365.9209,1+): Label: I3, Spot_Id: 211167, Peak_List_Id: 56612, MSMS Job_Run_Id: 13135, Comment:
Query47 (2371.9314,1+): <no title>
Query48 (2379.9409,1+): <no title>
Query49 (2383.9265,1+): <no title>
Query50 (2604.0366,1+): <no title>
Query51 (2691.1211,1+): <no title>
Query52 (2720.1304,1+): <no title>
Query53 (2748.1340,1+): <no title>
Query54 (3116.4670,1+): <no title>

Mascot Search Results

User :
Email :
Search title : F:\ROD_inmunoproteoma\MALDI\160223_2016_561_562_ROD\ppw_F7_145631467706.txt
MS data file : F:\ROD_inmunoproteoma\MALDI\160223_2016_561_562_ROD\ppw_F7_145631467706.txt
Database : Brachyspiraceae Brachyspiraceae_20160127-151122 (40573 sequences; 13470793 residues)
Timestamp : 24 Feb 2016 at 12:02:37 GMT
Warning : A Peptide summary report will usually give a much clearer picture of MS/MS search results.
Top Score : 245 for J9ULK0, J9ULK0_BRAPL Aspartyl-tRNA synthetase OS=Brachyspira pilosicoli B2904 GN=syd PE=3 SV=1

Probability Based Mowse Score

Protein score is -10*Log(P), where P is the probability that the observed match is a random event.
Protein scores greater than 59 are significant (p<0.05).
Protein scores are derived from ions scores as a non-probabilistic basis for ranking protein hits.

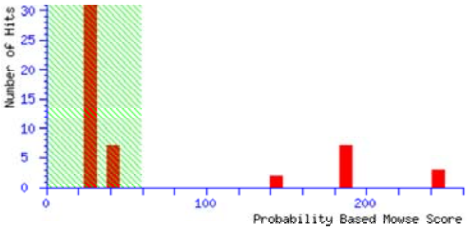

Protein Summary Report

Format As | Protein Summary (deprecated) | Help

Significance threshold p< 0.05 | Max. number of hits AUTO

Standard scoring ◉ MudPIT scoring ○ | Ions score or expect cut-off 0 | Show sub-sets 0

Show pop-ups ◉ Suppress pop-ups ○ | Sort unassigned Decreasing Score | Require bold red ☐

Re-Search All | Search Unmatched

Index

	Accession	Mass	Score	Description
1.	J9ULK0	67502	245	J9ULK0_BRAPL Aspartyl-tRNA synthetase OS=Brachyspira pilosicoli B2904 GN=syd PE=3 SV=1
2.	D8IE58	67502	245	D8IE58_BRAP9 Aspartyl-tRNA synthetase OS=Brachyspira pilosicoli (strain ATCC BAA-1826 / 95/1000) GN=aspS PE=3
3.	K0JHD0	67605	245	K0JHD0_BRAPL Aspartyl-tRNA synthetase OS=Brachyspira pilosicoli WesB GN=syd PE=3 SV=1
4.	C0R2C9	67239	191	SYDND_BRAHW Aspartate--tRNA(Asp/Asn) ligase OS=Brachyspira hyodysenteriae (strain ATCC 49526 / WA1) GN=aspS PE
5.	A0A0H0W5F8	67239	191	A0A0H0W5F8_BRAHO Aspartyl-tRNA synthetase OS=Brachyspira hyodysenteriae GN=SU43_06195 PE=3 SV=1
6.	A0A0H0UF37	67225	191	A0A0H0UF37_BRAHO Aspartyl-tRNA synthetase OS=Brachyspira hyodysenteriae GN=SR30_08840 PE=3 SV=1
7.	A0A0G4KB43	67239	191	A0A0G4KB43_9SPIR Aspartate--tRNA(Asp/Asn) ligase OS=Brachyspira suanatina GN=aspS PE=3 SV=1
8.	G0EIE2	67211	191	G0EIE2_BRAIP Aspartyl-tRNA synthetase OS=Brachyspira intermedia (strain ATCC 51140 / PWS/A) GN=aspS PE=3 SV=1
9.	L8XTL3	67173	191	L8XTL3_9SPIR Aspartyl-tRNA synthetase OS=Brachyspira hampsonii 30599 GN=H263_08879 PE=3 SV=1
10.	L0X5G2	67255	191	L0X5G2_9SPIR Aspartyl-tRNA synthetase OS=Brachyspira hampsonii 30446 GN=A966_01371 PE=3 SV=1
11.	D5UA48	67314	145	D5UA48_BRAM5 Aspartyl-tRNA synthetase OS=Brachyspira murdochii (strain ATCC 51284 / DSM 12563 / 56-150) GN=Bmu
12.	R5LAG2	67585	145	R5LAG2_9SPIR Aspartate--tRNA ligase OS=Brachyspira sp. CAG:700 GN=BN758_01548 PE=3 SV=1
13.	D8IB69	64822	37	D8IB69_BRAP9 Proline--tRNA ligase OS=Brachyspira pilosicoli (strain ATCC BAA-1826 / 95/1000) GN=proS PE=3 SV=1

Results List

1. J9ULK0 **Mass:** 67502 **Score:** 245 **Expect:** 1.3e-20 **Queries matched:** 4
J9ULK0_BRAPL Aspartyl-tRNA synthetase OS=Brachyspira pilosicoli B2904 GN=syd PE=3 SV=1

Observed	Mr(expt)	Mr(calc)	ppm	Start	End	Miss	Ions	Peptide
1105.6233	1104.6160	1104.5928	21.1	402 -	411	0	34	K.VTFDALGNLR.L
1125.6263	1124.6190	1124.5978	18.9	147 -	155	1	12	R.FEITNAFRK.H
1163.6514	1162.6441	1162.6169	23.5	204 -	213	0	8	K.QILMIGGFER.Y
1964.9600	1963.9527	1963.9236	14.9	473 -	491	0	134	R.SDTYDLVLNGNEIGGGGQR.I

No match to: 2691.3352

2. D8IE58 **Mass:** 67502 **Score:** 245 **Expect:** 1.3e-20 **Queries matched:** 4
D8IE58_BRAP9 Aspartyl-tRNA synthetase OS=Brachyspira pilosicoli (strain ATCC BAA-1826 / 95/1000) GN=aspS PE=3 SV=1

Observed	Mr(expt)	Mr(calc)	ppm	Start	End	Miss	Ions	Peptide
1105.6233	1104.6160	1104.5928	21.1	402 -	411	0	34	K.VTFDALGNLR.L
1125.6263	1124.6190	1124.5978	18.9	147 -	155	1	12	R.FEITNAFRK.H
1163.6514	1162.6441	1162.6169	23.5	204 -	213	0	8	K.QILMIGGFER.Y
1964.9600	1963.9527	1963.9236	14.9	473 -	491	0	134	R.SDTYDLVLNGNEIGGGGQR.I

No match to: 2691.3352

3. K0JHD0 **Mass:** 67605 **Score:** 245 **Expect:** 1.3e-20 **Queries matched:** 4
K0JHD0_BRAPL Aspartyl-tRNA synthetase OS=Brachyspira pilosicoli WesB GN=syd PE=3 SV=1

Observed	Mr(expt)	Mr(calc)	ppm	Start	End	Miss	Ions	Peptide
1105.6233	1104.6160	1104.5928	21.1	402 -	411	0	34	K.VTFDALGNLR.L
1125.6263	1124.6190	1124.5978	18.9	147 -	155	1	12	R.FEITNAFRK.H
1163.6514	1162.6441	1162.6169	23.5	204 -	213	0	8	K.QILMIGGFER.Y
1964.9600	1963.9527	1963.9236	14.9	473 -	491	0	134	R.SDTYDLVLNGNEIGGGGQR.I

No match to: 2691.3352

4. C0R2C9 **Mass:** 67239 **Score:** 191 **Expect:** 3.2e-15 **Queries matched:** 2
SYDND_BRAHW Aspartate--tRNA(Asp/Asn) ligase OS=Brachyspira hyodysenteriae (strain ATCC 49526 / WA1) GN=aspS PE=3 SV=1

Observed	Mr(expt)	Mr(calc)	ppm	Start - End	Miss	Ions	Peptide
1105.6233	1104.6160	1104.5928	21.1	401 - 410	0	34	K.VTFDALGNLR.L
1964.9600	1963.9527	1963.9236	14.9	472 - 490	0	134	R.SDTYDLVLNGNEIGGGGQR.I

No match to: 1125.6263, 1163.6514, 2691.3352

5. A0A0H0W5F8 **Mass:** 67239 **Score:** 191 **Expect:** 3.2e-15 **Queries matched:** 2
A0A0H0W5F8_BRAHO Aspartyl-tRNA synthetase OS=Brachyspira hyodysenteriae GN=SU43_06195 PE=3 SV=1

Observed	Mr(expt)	Mr(calc)	ppm	Start - End	Miss	Ions	Peptide
1105.6233	1104.6160	1104.5928	21.1	401 - 410	0	34	K.VTFDALGNLR.L
1964.9600	1963.9527	1963.9236	14.9	472 - 490	0	134	R.SDTYDLVLNGNEIGGGGQR.I

No match to: 1125.6263, 1163.6514, 2691.3352

6. A0A0H0UF37 **Mass:** 67225 **Score:** 191 **Expect:** 3.2e-15 **Queries matched:** 2
A0A0H0UF37_BRAHO Aspartyl-tRNA synthetase OS=Brachyspira hyodysenteriae GN=SR30_08840 PE=3 SV=1

Observed	Mr(expt)	Mr(calc)	ppm	Start - End	Miss	Ions	Peptide
1105.6233	1104.6160	1104.5928	21.1	401 - 410	0	34	K.VTFDALGNLR.L
1964.9600	1963.9527	1963.9236	14.9	472 - 490	0	134	R.SDTYDLVLNGNEIGGGGQR.I

No match to: 1125.6263, 1163.6514, 2691.3352

7. A0A0G4KB43 **Mass:** 67239 **Score:** 191 **Expect:** 3.2e-15 **Queries matched:** 2
A0A0G4KB43_9SPIR Aspartate--tRNA(Asp/Asn) ligase OS=Brachyspira suanatina GN=aspS PE=3 SV=1

Observed	Mr(expt)	Mr(calc)	ppm	Start - End	Miss	Ions	Peptide
1105.6233	1104.6160	1104.5928	21.1	401 - 410	0	34	K.VTFDALGNLR.L
1964.9600	1963.9527	1963.9236	14.9	472 - 490	0	134	R.SDTYDLVLNGNEIGGGGQR.I

No match to: 1125.6263, 1163.6514, 2691.3352

8. G0EIE2 **Mass:** 67211 **Score:** 191 **Expect:** 3.2e-15 **Queries matched:** 2
G0EIE2_BRAIP Aspartyl-tRNA synthetase OS=Brachyspira intermedia (strain ATCC 51140 / PWS/A) GN=aspS PE=3 SV=1

Observed	Mr(expt)	Mr(calc)	ppm	Start - End	Miss	Ions	Peptide
1105.6233	1104.6160	1104.5928	21.1	401 - 410	0	34	K.VTFDALGNLR.L
1964.9600	1963.9527	1963.9236	14.9	472 - 490	0	134	R.SDTYDLVLNGNEIGGGGQR.I

No match to: 1125.6263, 1163.6514, 2691.3352

9. L8XTL3 **Mass:** 67173 **Score:** 191 **Expect:** 3.2e-15 **Queries matched:** 2
L8XTL3_9SPIR Aspartyl-tRNA synthetase OS=Brachyspira hampsonii 30599 GN=H263_08879 PE=3 SV=1

Observed	Mr(expt)	Mr(calc)	ppm	Start - End	Miss	Ions	Peptide
1105.6233	1104.6160	1104.5928	21.1	401 - 410	0	34	K.VTFDALGNLR.L
1964.9600	1963.9527	1963.9236	14.9	472 - 490	0	134	R.SDTYDLVLNGNEIGGGGQR.I

No match to: 1125.6263, 1163.6514, 2691.3352

10. L0X5G2 **Mass:** 67255 **Score:** 191 **Expect:** 3.2e-15 **Queries matched:** 2
L0X5G2_9SPIR Aspartyl-tRNA synthetase OS=Brachyspira hampsonii 30446 GN=A966_01371 PE=3 SV=1

Observed	Mr(expt)	Mr(calc)	ppm	Start - End	Miss	Ions	Peptide
1105.6233	1104.6160	1104.5928	21.1	401 - 410	0	34	K.VTFDALGNLR.L
1964.9600	1963.9527	1963.9236	14.9	472 - 490	0	134	R.SDTYDLVLNGNEIGGGGQR.I

No match to: 1125.6263, 1163.6514, 2691.3352

11. D5UA48 **Mass:** 67314 **Score:** 145 **Expect:** 1.3e-10 **Queries matched:** 1
D5UA48_BRAM5 Aspartyl-tRNA synthetase OS=Brachyspira murdochii (strain ATCC 51284 / DSM 12563 / 56-150) GN=Bmur_1484 PE=3 SV=1

Observed	Mr(expt)	Mr(calc)	ppm	Start - End	Miss	Ions	Peptide
1964.9600	1963.9527	1963.9236	14.9	473 - 491	0	134	R.SDTYDLVLNGNEIGGGGQR.I

No match to: 1105.6233, 1125.6263, 1163.6514, 2691.3352

12. R5LAG2 **Mass:** 67585 **Score:** 145 **Expect:** 1.3e-10 **Queries matched:** 1
R5LAG2_9SPIR Aspartate--tRNA ligase OS=Brachyspira sp. CAG:700 GN=BN758_01548 PE=3 SV=1

Observed	Mr(expt)	Mr(calc)	ppm	Start - End	Miss	Ions	Peptide
1964.9600	1963.9527	1963.9236	14.9	472 - 490	0	134	R.SDTYDLVLNGNEIGGGGQR.I

No match to: 1105.6233, 1125.6263, 1163.6514, 2691.3352

13. D8IB69 **Mass:** 64822 **Score:** 37 **Expect:** 8.3 **Queries matched:** 3
D8IB69_BRAP9 Proline--tRNA ligase OS=Brachyspira pilosicoli (strain ATCC BAA-1826 / 95/1000) GN=proS PE=3 SV=1

Observed	Mr(expt)	Mr(calc)	ppm	Start - End	Miss	Ions	Peptide
1105.6233	1104.6160	1104.5961	18.0	180 - 189	1	---	K.RMGLDTVSVK.A
1125.6263	1124.6190	1124.6593	-35.85	269 - 277	1	---	K.NFIKSIIYK.T
1163.6514	1162.6441	1162.6346	8.18	528 - 537	1	---	K.SLERGVVEFK.L

No match to: 1964.9600, 2691.3352

Search Parameters

Type of search : Sequence Query
Enzyme : Trypsin
Fixed modifications : Carbamidomethyl (C)
Variable modifications : Oxidation (M)
Mass values : Monoisotopic
Protein Mass : Unrestricted
Peptide Mass Tolerance : ± 50 ppm
Fragment Mass Tolerance: ± 0.5 Da
Max Missed Cleavages : 1
Instrument type : MALDI-TOF-TOF
Query1 (1105.6233,1+) : Locus:7.1.1.213490.57269
Query2 (1125.6263,1+) : Locus:7.1.1.213490.57267
Query3 (1163.6514,1+) : Locus:7.1.1.213490.57268
Query4 (1964.9600,1+) : Locus:7.1.1.213490.57266
Query5 (2691.3352,1+) : Locus:7.1.1.213490.57270

Mascot: http://www.matrixscience.com/

Mascot Search Results

User	:	
Email	:	
Search title	:	F:\ROD_inmunoproteoma\MALDI\160223_2016_561_562_ROD\ppw_G3_145631468010.txt
MS data file	:	F:\ROD_inmunoproteoma\MALDI\160223_2016_561_562_ROD\ppw_G3_145631468010.txt
Database	:	Brachyspiraceae Brachyspiraceae_20160127-151122 (40573 sequences; 13470793 residues)
Timestamp	:	24 Feb 2016 at 12:02:47 GMT
Warning	:	A Peptide summary report will usually give a much clearer picture of MS/MS search results.
Top Score	:	331 for D8IDC2, D8IDC2_BRAP9 Uncharacterized protein OS=Brachyspira pilosicoli (strain ATCC BAA-1826 / 95/1000) GN=BP95100

Probability Based Mowse Score

Protein score is -10*Log(P), where P is the probability that the observed match is a random event.
Protein scores greater than 59 are significant (p<0.05).
Protein scores are derived from ions scores as a non-probabilistic basis for ranking protein hits.

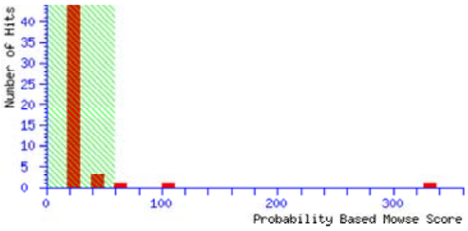

Protein Summary Report

Format As | Protein Summary (deprecated) | Help

Significance threshold p< 0.05 | Max. number of hits AUTO

Standard scoring ◉ MudPIT scoring ○ | Ions score or expect cut-off 0 | Show sub-sets 0

Show pop-ups ◉ Suppress pop-ups ○ | Sort unassigned Decreasing Score | Require bold red ☐

Re-Search All | Search Unmatched

Index

	Accession	Mass	Score	Description
1.	D8IDC2	62081	331	D8IDC2_BRAP9 Uncharacterized protein OS=Brachyspira pilosicoli (strain ATCC BAA-1826 / 95/1000) GN=BP951000_115
2.	K0JN47	62251	113	K0JN47_BRAPL Putative treponemal membrane protein OS=Brachyspira pilosicoli WesB GN=tmpB PE=4 SV=1
3.	J9UXF7	62380	63	J9UXF7_BRAPL Putative treponemal membrane protein OS=Brachyspira pilosicoli B2904 GN=tmpB PE=4 SV=1
4.	A0A0H0UXU8	50776	44	A0A0H0UXU8_BRAHO Peptidase C69 OS=Brachyspira hyodysenteriae GN=SZ49_08125 PE=4 SV=1

Results List

1. D8IDC2 **Mass:** 62081 **Score:** 331 **Expect:** 3.2e-29 **Queries matched:** 4
D8IDC2_BRAP9 Uncharacterized protein OS=Brachyspira pilosicoli (strain ATCC BAA-1826 / 95/1000) GN=BP951000_1156 PE=4 SV=1

Observed	Mr(expt)	Mr(calc)	ppm	Start - End	Miss	Ions	Peptide
1240.6873	1239.6800	1239.6823	-1.81	523 - 533	0	37	R.LIIPSLNGEER.S
1435.8287	1434.8214	1434.8235	-1.45	462 - 473	0	54	K.VTVLPQYYVVVR.R
1931.0433	1930.0360	1930.0384	-1.25	506 - 522	1	46	R.NVLRDPNNPDLILPGQR.L
2421.0911	2420.0838	2420.0842	-0.15	534 - 553	0	135	R.SGDYNPDLEYLTYDEVMQLR.Q

No match to: 1097.4120

2. K0JN47 **Mass:** 62251 **Score:** 113 **Expect:** 2e-07 **Queries matched:** 2
K0JN47_BRAPL Putative treponemal membrane protein OS=Brachyspira pilosicoli WesB GN=tmpB PE=4 SV=1

Observed	Mr(expt)	Mr(calc)	ppm	Start - End	Miss	Ions	Peptide
1240.6873	1239.6800	1239.6823	-1.81	523 - 533	0	37	R.LIIPSLNGEER.S
1435.8287	1434.8214	1434.8235	-1.45	462 - 473	0	54	K.VTVLPQYYVVVR.R

No match to: 1097.4120, 1931.0433, 2421.0911

3. J9UXF7 **Mass:** 62380 **Score:** 63 **Expect:** 0.019 **Queries matched:** 1
J9UXF7_BRAPL Putative treponemal membrane protein OS=Brachyspira pilosicoli B2904 GN=tmpB PE=4 SV=1

Observed	Mr(expt)	Mr(calc)	ppm	Start - End	Miss	Ions	Peptide
1435.8287	1434.8214	1434.8235	-1.45	464 - 475	0	54	K.VTVLPQYYVVVR.R

No match to: 1097.4120, 1240.6873, 1931.0433, 2421.0911

4. A0A0H0UXU8 **Mass:** 50776 **Score:** 44 **Expect:** 1.7 **Queries matched:** 3
A0A0H0UXU8_BRAHO Peptidase C69 OS=Brachyspira hyodysenteriae GN=SZ49_08125 PE=4 SV=1

Observed	Mr(expt)	Mr(calc)	ppm	Start - End	Miss	Ions	Peptide
1240.6873	1239.6800	1239.6863	-5.07	95 - 104	1	---	K.YIVKDFIQSK.E
1931.0433	1930.0360	1930.0259	5.25	269 - 287	1	---	K.LGEKIASDVVTAVDDGTIK.N
2421.0911	2420.0838	2420.1926	-44.96	204 - 225	1	---	K.DINLEDMAIETANSAIKMLNSK.Y

No match to: 1097.4120, 1435.8287

Search Parameters

Type of search	:	Sequence Query
Enzyme	:	Trypsin

```
Fixed modifications    : Carbamidomethyl (C)
Variable modifications : Oxidation (M)
Mass values            : Monoisotopic
Protein Mass           : Unrestricted
Peptide Mass Tolerance : ± 50 ppm
Fragment Mass Tolerance: ± 0.5 Da
Max Missed Cleavages   : 1
Instrument type        : MALDI-TOF-TOF
Query1 (1097.4120,1+)  : Locus:11.1.1.213510.57290
Query2 (1240.6873,1+)  : Locus:11.1.1.213510.57288
Query3 (1435.8287,1+)  : Locus:11.1.1.213510.57286
Query4 (1931.0433,1+)  : Locus:11.1.1.213510.57289
Query5 (2421.0911,1+)  : Locus:11.1.1.213510.57287
```

Mascot: http://www.matrixscience.com/

MATRIX SCIENCE Mascot Search Results

User	:
Email	:
Search title	: F:\ROD_inmunoproteoma\MALDI\ppw_2016_562_563_ROD\ppw_G17_145397048304.txt
MS data file	: F:\ROD_inmunoproteoma\MALDI\ppw_2016_562_563_ROD\ppw_G17_145397048304.txt
Database	: Brachyspiraceae Brachyspiraceae_20160127-151122 (40573 sequences; 13470793 residues)
Timestamp	: 23 Feb 2016 at 16:05:13 GMT
Warning	: A Peptide summary report will usually give a much clearer picture of MS/MS search results.
Top Score	: 170 for A0A0H0UN97, A0A0H0UN97_BRAHO Phosphoenolpyruvate carboxykinase [GTP] OS=Brachyspira hyodysenteriae GN=pckG PE=3 SV

Probability Based Mowse Score

Protein score is -10*Log(P), where P is the probability that the observed match is a random event.
Protein scores greater than 59 are significant (p<0.05).
Protein scores are derived from ions scores as a non-probabilistic basis for ranking protein hits.

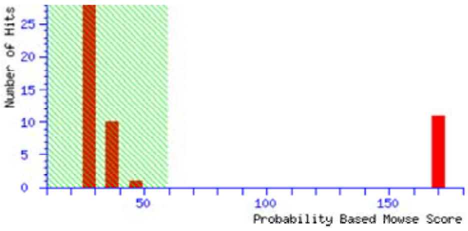

Protein Summary Report

Format As | Protein Summary (deprecated) | Help

Significance threshold p< 0.05 | Max. number of hits AUTO

Standard scoring ◉ MudPIT scoring ○ | Ions score or expect cut-off 0 | Show sub-sets 0

Show pop-ups ◉ Suppress pop-ups ○ | Sort unassigned Decreasing Score | Require bold red ☐

Re-Search All | Search Unmatched

Index

	Accession	Mass	Score	Description
1.	A0A0H0UN97	66923	170	A0A0H0UN97_BRAHO Phosphoenolpyruvate carboxykinase [GTP] OS=Brachyspira hyodysenteriae GN=pckG PE=3 SV=1
2.	A0A0H0W3V6	67483	170	A0A0H0W3V6_BRAHO Phosphoenolpyruvate carboxykinase [GTP] OS=Brachyspira hyodysenteriae GN=pckG PE=3 SV=1
3.	A0A0G4K843	67513	170	A0A0G4K843_9SPIR Phosphoenolpyruvate carboxykinase [GTP] OS=Brachyspira suanatina GN=pckG PE=3 SV=1
4.	C0R0E5	67483	170	C0R0E5_BRAHW Phosphoenolpyruvate carboxykinase [GTP] OS=Brachyspira hyodysenteriae (strain ATCC 49526 / WA1) G
5.	G0ELG2	67542	170	G0ELG2_BRAIP Phosphoenolpyruvate carboxykinase [GTP] OS=Brachyspira intermedia (strain ATCC 51140 / PWS/A) GN=
6.	J9UPD6	67388	170	J9UPD6_BRAPL Phosphoenolpyruvate carboxykinase [GTP] OS=Brachyspira pilosicoli B2904 GN=pckG PE=3 SV=1
7.	D8IFS5	67465	170	D8IFS5_BRAP9 Phosphoenolpyruvate carboxykinase [GTP] OS=Brachyspira pilosicoli (strain ATCC BAA-1826 / 95/1000
8.	K0JJV6	67371	170	K0JJV6_BRAPL Phosphoenolpyruvate carboxykinase [GTP] OS=Brachyspira pilosicoli WesB GN=pckG PE=3 SV=1
9.	D5U5S2	67457	170	D5U5S2_BRAM5 Phosphoenolpyruvate carboxykinase [GTP] OS=Brachyspira murdochii (strain ATCC 51284 / DSM 12563 /
10.	L8XUY8	67488	170	L8XUY8_9SPIR Phosphoenolpyruvate carboxykinase [GTP] OS=Brachyspira hampsonii 30599 GN=pckG PE=3 SV=1
11.	L0X1S2	67509	170	L0X1S2_9SPIR Phosphoenolpyruvate carboxykinase [GTP] OS=Brachyspira hampsonii 30446 GN=pckG PE=3 SV=1
12.	R5LL43	67626	48	R5LL43_9SPIR Phosphoenolpyruvate carboxykinase [GTP] OS=Brachyspira sp. CAG:700 GN=pckG PE=3 SV=1

Results List

1. A0A0H0UN97 **Mass:** 66923 **Score:** 170 **Expect:** 4.1e-13 **Queries matched:** 3
A0A0H0UN97_BRAHO Phosphoenolpyruvate carboxykinase [GTP] OS=Brachyspira hyodysenteriae GN=pckG PE=3 SV=1

Observed	Mr(expt)	Mr(calc)	ppm	Start	End	Miss	Ions	Peptide
1023.5002	1022.4929	1022.5158	-22.41	56 -	63	0	53	K.RPHSHSFR.S
1272.6560	1271.6487	1271.6815	-25.78	480 -	488	1	39	R.IFYVNWFRK.D
1850.8539	1849.8466	1849.8932	-25.17	56 -	71	1	41	K.RPHSHSFRSDPSDVAR.V

No match to: 1316.6786, 1832.7952

2. A0A0H0W3V6 **Mass:** 67483 **Score:** 170 **Expect:** 4.1e-13 **Queries matched:** 3
A0A0H0W3V6_BRAHO Phosphoenolpyruvate carboxykinase [GTP] OS=Brachyspira hyodysenteriae GN=pckG PE=3 SV=1

Observed	Mr(expt)	Mr(calc)	ppm	Start	End	Miss	Ions	Peptide
1023.5002	1022.4929	1022.5158	-22.41	56 -	63	0	53	K.RPHSHSFR.S
1272.6560	1271.6487	1271.6815	-25.78	486 -	494	1	39	R.IFYVNWFRK.D
1850.8539	1849.8466	1849.8932	-25.17	56 -	71	1	41	K.RPHSHSFRSDPSDVAR.V

No match to: 1316.6786, 1832.7952

3. A0A0G4K843 **Mass:** 67513 **Score:** 170 **Expect:** 4.1e-13 **Queries matched:** 3
A0A0G4K843_9SPIR Phosphoenolpyruvate carboxykinase [GTP] OS=Brachyspira suanatina GN=pckG PE=3 SV=1

Observed	Mr(expt)	Mr(calc)	ppm	Start	End	Miss	Ions	Peptide
1023.5002	1022.4929	1022.5158	-22.41	56 -	63	0	53	K.RPHSHSFR.S
1272.6560	1271.6487	1271.6815	-25.78	486 -	494	1	39	R.IFYVNWFRK.D
1850.8539	1849.8466	1849.8932	-25.17	56 -	71	1	41	K.RPHSHSFRSDPSDVAR.V

No match to: 1316.6786, 1832.7952

4. C0R0E5 **Mass:** 67483 **Score:** 170 **Expect:** 4.1e-13 **Queries matched:** 3
C0R0E5_BRAHW Phosphoenolpyruvate carboxykinase [GTP] OS=Brachyspira hyodysenteriae (strain ATCC 49526 / WA1) GN=pckG PE=3 SV=1

Observed	Mr(expt)	Mr(calc)	ppm	Start	End	Miss	Ions	Peptide

1023.5002	1022.4929	1022.5158	-22.41	56	63	0	53	K.RPHSHSFR.S
1272.6560	1271.6487	1271.6815	-25.78	486	494	1	39	R.IFYVNWFRK.D
1850.8539	1849.8466	1849.8932	-25.17	56	71	1	41	K.RPHSHSFRSDPSDVAR.V

No match to: 1316.6786, 1832.7952

5. G0ELG2 **Mass:** 67542 **Score:** 170 **Expect:** 4.1e-13 **Queries matched:** 3

G0ELG2_BRAIP Phosphoenolpyruvate carboxykinase [GTP] OS=Brachyspira intermedia (strain ATCC 51140 / PWS/A) GN=pckG PE=3 SV=1

Observed	Mr(expt)	Mr(calc)	ppm	Start	End	Miss	Ions	Peptide
1023.5002	1022.4929	1022.5158	-22.41	56	63	0	53	K.RPHSHSFR.S
1272.6560	1271.6487	1271.6815	-25.78	486	494	1	39	R.IFYVNWFRK.D
1850.8539	1849.8466	1849.8932	-25.17	56	71	1	41	K.RPHSHSFRSDPSDVAR.V

No match to: 1316.6786, 1832.7952

6. J9UPD6 **Mass:** 67388 **Score:** 170 **Expect:** 4.1e-13 **Queries matched:** 3

J9UPD6_BRAPL Phosphoenolpyruvate carboxykinase [GTP] OS=Brachyspira pilosicoli B2904 GN=pckG PE=3 SV=1

Observed	Mr(expt)	Mr(calc)	ppm	Start	End	Miss	Ions	Peptide
1023.5002	1022.4929	1022.5158	-22.41	56	63	0	53	K.RPHSHSFR.S
1272.6560	1271.6487	1271.6815	-25.78	486	494	1	39	R.IFYVNWFRK.D
1850.8539	1849.8466	1849.8932	-25.17	56	71	1	41	K.RPHSHSFRSDPSDVAR.V

No match to: 1316.6786, 1832.7952

7. D8IFS5 **Mass:** 67465 **Score:** 170 **Expect:** 4.1e-13 **Queries matched:** 3

D8IFS5_BRAP9 Phosphoenolpyruvate carboxykinase [GTP] OS=Brachyspira pilosicoli (strain ATCC BAA-1826 / 95/1000) GN=pckG PE=3 SV=1

Observed	Mr(expt)	Mr(calc)	ppm	Start	End	Miss	Ions	Peptide
1023.5002	1022.4929	1022.5158	-22.41	56	63	0	53	K.RPHSHSFR.S
1272.6560	1271.6487	1271.6815	-25.78	486	494	1	39	R.IFYVNWFRK.D
1850.8539	1849.8466	1849.8932	-25.17	56	71	1	41	K.RPHSHSFRSDPSDVAR.V

No match to: 1316.6786, 1832.7952

8. K0JJV6 **Mass:** 67371 **Score:** 170 **Expect:** 4.1e-13 **Queries matched:** 3

K0JJV6_BRAPL Phosphoenolpyruvate carboxykinase [GTP] OS=Brachyspira pilosicoli WesB GN=pckG PE=3 SV=1

Observed	Mr(expt)	Mr(calc)	ppm	Start	End	Miss	Ions	Peptide
1023.5002	1022.4929	1022.5158	-22.41	56	63	0	53	K.RPHSHSFR.S
1272.6560	1271.6487	1271.6815	-25.78	486	494	1	39	R.IFYVNWFRK.D
1850.8539	1849.8466	1849.8932	-25.17	56	71	1	41	K.RPHSHSFRSDPSDVAR.V

No match to: 1316.6786, 1832.7952

9. D5U5S2 **Mass:** 67457 **Score:** 170 **Expect:** 4.1e-13 **Queries matched:** 3

D5U5S2_BRAM5 Phosphoenolpyruvate carboxykinase [GTP] OS=Brachyspira murdochii (strain ATCC 51284 / DSM 12563 / 56-150) GN=pckG PE=3 SV

Observed	Mr(expt)	Mr(calc)	ppm	Start	End	Miss	Ions	Peptide
1023.5002	1022.4929	1022.5158	-22.41	56	63	0	53	K.RPHSHSFR.S
1272.6560	1271.6487	1271.6815	-25.78	486	494	1	39	R.IFYVNWFRK.N
1850.8539	1849.8466	1849.8932	-25.17	56	71	1	41	K.RPHSHSFRSDPSDVAR.V

No match to: 1316.6786, 1832.7952

10. L8XUY8 **Mass:** 67488 **Score:** 170 **Expect:** 4.1e-13 **Queries matched:** 3

L8XUY8_9SPIR Phosphoenolpyruvate carboxykinase [GTP] OS=Brachyspira hampsonii 30599 GN=pckG PE=3 SV=1

Observed	Mr(expt)	Mr(calc)	ppm	Start	End	Miss	Ions	Peptide
1023.5002	1022.4929	1022.5158	-22.41	56	63	0	53	K.RPHSHSFR.S
1272.6560	1271.6487	1271.6815	-25.78	486	494	1	39	R.IFYVNWFRK.N
1850.8539	1849.8466	1849.8932	-25.17	56	71	1	41	K.RPHSHSFRSDPSDVAR.V

No match to: 1316.6786, 1832.7952

11. L0X1S2 **Mass:** 67509 **Score:** 170 **Expect:** 4.1e-13 **Queries matched:** 3

L0X1S2_9SPIR Phosphoenolpyruvate carboxykinase [GTP] OS=Brachyspira hampsonii 30446 GN=pckG PE=3 SV=1

Observed	Mr(expt)	Mr(calc)	ppm	Start	End	Miss	Ions	Peptide
1023.5002	1022.4929	1022.5158	-22.41	56	63	0	53	K.RPHSHSFR.S
1272.6560	1271.6487	1271.6815	-25.78	486	494	1	39	K.IFYVNWFRK.D
1850.8539	1849.8466	1849.8932	-25.17	56	71	1	41	K.RPHSHSFRSDPSDVAR.V

No match to: 1316.6786, 1832.7952

12. R5LL43 **Mass:** 67626 **Score:** 48 **Expect:** 0.67 **Queries matched:** 1

R5LL43_9SPIR Phosphoenolpyruvate carboxykinase [GTP] OS=Brachyspira sp. CAG:700 GN=pckG PE=3 SV=1

Observed	Mr(expt)	Mr(calc)	ppm	Start	End	Miss	Ions	Peptide
1272.6560	1271.6487	1271.6815	-25.78	485	493	1	39	K.IFYVNWFRK.D

No match to: 1023.5002, 1316.6786, 1832.7952, 1850.8539

Search Parameters

**Type of search**	**: Sequence Query**
**Enzyme**	**: Trypsin**
**Fixed modifications**	**: Carbamidomethyl (C)**
**Variable modifications**	**: Oxidation (M)**
**Mass values**	**: Monoisotopic**
**Protein Mass**	**: Unrestricted**
**Peptide Mass Tolerance**	**: ± 50 ppm**
**Fragment Mass Tolerance**	**: ± 0.5 Da**
**Max Missed Cleavages**	**: 1**
**Instrument type**	**: MALDI-TOF-TOF**
**Query1 (1023.5002,1+)**	**: Locus:5.1.1.211133.56535**
**Query2 (1272.6560,1+)**	**: Locus:5.1.1.211133.56537**
**Query3 (1316.6786,1+)**	**: Locus:5.1.1.211133.56536**
**Query4 (1832.7952,1+)**	**: Locus:5.1.1.211133.56539**
**Query5 (1850.8539,1+)**	**: Locus:5.1.1.211133.56538**

Mascot: http://www.matrixscience.com/

Mascot Search Results

User	:	
Email	:	
Search title	:	F:\ROD_inmunoproteoma\MALDI\ppw_2016_561_562_563_565\ppw_H7_145467558819.txt
MS data file	:	F:\ROD_inmunoproteoma\MALDI\ppw_2016_561_562_563_565\ppw_H7_145467558819.txt
Database	:	Brachyspiraceae Brachyspiraceae_20160127-151122 (40573 sequences; 13470793 residues)
Timestamp	:	23 Feb 2016 at 16:37:00 GMT
Warning	:	A Peptide summary report will usually give a much clearer picture of MS/MS search results.
Top Score	:	108 for J9ULK0, J9ULK0_BRAPL Aspartyl-tRNA synthetase OS=Brachyspira pilosicoli B2904 GN=syd PE=3 SV=1

Probability Based Mowse Score

Protein score is -10*Log(P), where P is the probability that the observed match is a random event.
Protein scores greater than 59 are significant (p<0.05).
Protein scores are derived from ions scores as a non-probabilistic basis for ranking protein hits.

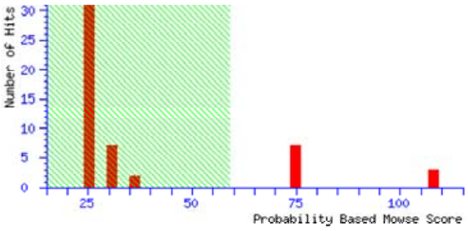

Protein Summary Report

Format As | Protein Summary (deprecated) | Help

Significance threshold p< 0.05 | Max. number of hits AUTO

Standard scoring ◉ MudPIT scoring ○ | Ions score or expect cut-off 0 | Show sub-sets 0

Show pop-ups ◉ Suppress pop-ups ○ | Sort unassigned Decreasing Score | Require bold red ☐

Re-Search All | Search Unmatched

Index

	Accession	Mass	Score	Description
1.	J9ULK0	67502	108	J9ULK0_BRAPL Aspartyl-tRNA synthetase OS=Brachyspira pilosicoli B2904 GN=syd PE=3 SV=1
2.	D8IE58	67502	108	D8IE58_BRAP9 Aspartyl-tRNA synthetase OS=Brachyspira pilosicoli (strain ATCC BAA-1826 / 95/1000) GN=aspS PE=3
3.	K0JHD0	67605	108	K0JHD0_BRAPL Aspartyl-tRNA synthetase OS=Brachyspira pilosicoli WesB GN=syd PE=3 SV=1
4.	C0R2C9	67239	72	SYDND_BRAHW Aspartate--tRNA(Asp/Asn) ligase OS=Brachyspira hyodysenteriae (strain ATCC 49526 / WA1) GN=aspS PE
5.	A0A0H0W5F8	67239	72	A0A0H0W5F8_BRAHO Aspartyl-tRNA synthetase OS=Brachyspira hyodysenteriae GN=SU43_06195 PE=3 SV=1
6.	A0A0H0UF37	67225	72	A0A0H0UF37_BRAHO Aspartyl-tRNA synthetase OS=Brachyspira hyodysenteriae GN=SR30_08840 PE=3 SV=1
7.	A0A0G4KB43	67239	72	A0A0G4KB43_9SPIR Aspartate--tRNA(Asp/Asn) ligase OS=Brachyspira suanatina GN=aspS PE=3 SV=1
8.	G0EIE2	67211	72	G0EIE2_BRAIP Aspartyl-tRNA synthetase OS=Brachyspira intermedia (strain ATCC 51140 / PWS/A) GN=aspS PE=3 SV=1
9.	L8XTL3	67173	72	L8XTL3_9SPIR Aspartyl-tRNA synthetase OS=Brachyspira hampsonii 30599 GN=H263_08879 PE=3 SV=1
10.	L0X5G2	67255	72	L0X5G2_9SPIR Aspartyl-tRNA synthetase OS=Brachyspira hampsonii 30446 GN=A966_01371 PE=3 SV=1
11.	L8XPV1	12037	38	L8XPV1_9SPIR Cupin domain-containing protein OS=Brachyspira hampsonii 30599 GN=H263_06662 PE=4 SV=1

Results List

1. J9ULK0 **Mass:** 67502 **Score:** 108 **Expect:** 6.4e-07 **Queries matched:** 3
J9ULK0_BRAPL Aspartyl-tRNA synthetase OS=Brachyspira pilosicoli B2904 GN=syd PE=3 SV=1

Observed	Mr(expt)	Mr(calc)	ppm	Start	End	Miss	Ions	Peptide
1105.6346	1104.6273	1104.5928	31.3	402 -	411	0	32	K.VTFDALGNLR.L
1163.6599	1162.6526	1162.6169	30.8	204 -	213	0	20	K.QILMIGGFER.Y
1203.7272	1202.7199	1202.6771	35.6	315 -	324	1	19	K.NALDNKFIIR.C

No match to: 1320.6287, 2225.1716

2. D8IE58 **Mass:** 67502 **Score:** 108 **Expect:** 6.4e-07 **Queries matched:** 3
D8IE58_BRAP9 Aspartyl-tRNA synthetase OS=Brachyspira pilosicoli (strain ATCC BAA-1826 / 95/1000) GN=aspS PE=3 SV=1

Observed	Mr(expt)	Mr(calc)	ppm	Start	End	Miss	Ions	Peptide
1105.6346	1104.6273	1104.5928	31.3	402 -	411	0	32	K.VTFDALGNLR.L
1163.6599	1162.6526	1162.6169	30.8	204 -	213	0	20	K.QILMIGGFER.Y
1203.7272	1202.7199	1202.6771	35.6	315 -	324	1	19	K.NALDNKFIIR.C

No match to: 1320.6287, 2225.1716

3. K0JHD0 **Mass:** 67605 **Score:** 108 **Expect:** 6.4e-07 **Queries matched:** 3
K0JHD0_BRAPL Aspartyl-tRNA synthetase OS=Brachyspira pilosicoli WesB GN=syd PE=3 SV=1

Observed	Mr(expt)	Mr(calc)	ppm	Start	End	Miss	Ions	Peptide
1105.6346	1104.6273	1104.5928	31.3	402 -	411	0	32	K.VTFDALGNLR.L
1163.6599	1162.6526	1162.6169	30.8	204 -	213	0	20	K.QILMIGGFER.Y
1203.7272	1202.7199	1202.6771	35.6	315 -	324	1	19	K.NALDNKFIIR.C

No match to: 1320.6287, 2225.1716

4. C0R2C9 **Mass:** 67239 **Score:** 72 **Expect:** 0.0023 **Queries matched:** 2
SYDND_BRAHW Aspartate--tRNA(Asp/Asn) ligase OS=Brachyspira hyodysenteriae (strain ATCC 49526 / WA1) GN=aspS PE=3 SV=1

Observed	Mr(expt)	Mr(calc)	ppm	Start	End	Miss	Ions	Peptide
1105.6346	1104.6273	1104.5928	31.3	401 -	410	0	32	K.VTFDALGNLR.L

```
     1203.7272  1202.7199  1202.6771   35.6   315 - 324   1   19   K.NALDNKFIIR.C
     No match to: 1163.6599, 1320.6287, 2225.1716

5.   A0A0H0W5F8    Mass: 67239    Score: 72     Expect: 0.0023  Queries matched: 2
     A0A0H0W5F8_BRAHO Aspartyl-tRNA synthetase OS=Brachyspira hyodysenteriae GN=SU43_06195 PE=3 SV=1
      Observed   Mr(expt)   Mr(calc)   ppm  Start   End Miss Ions  Peptide
     1105.6346  1104.6273  1104.5928   31.3   401 - 410   0   32   K.VTFDALGNLR.L
     1203.7272  1202.7199  1202.6771   35.6   315 - 324   1   19   K.NALDNKFIIR.C
     No match to: 1163.6599, 1320.6287, 2225.1716

6.   A0A0H0UF37    Mass: 67225    Score: 72     Expect: 0.0023  Queries matched: 2
     A0A0H0UF37_BRAHO Aspartyl-tRNA synthetase OS=Brachyspira hyodysenteriae GN=SR30_08840 PE=3 SV=1
      Observed   Mr(expt)   Mr(calc)   ppm  Start   End Miss Ions  Peptide
     1105.6346  1104.6273  1104.5928   31.3   401 - 410   0   32   K.VTFDALGNLR.L
     1203.7272  1202.7199  1202.6771   35.6   315 - 324   1   19   K.NALDNKFIIR.C
     No match to: 1163.6599, 1320.6287, 2225.1716

7.   A0A0G4KB43    Mass: 67239    Score: 72     Expect: 0.0023  Queries matched: 2
     A0A0G4KB43_9SPIR Aspartate--tRNA(Asp/Asn) ligase OS=Brachyspira suanatina GN=aspS PE=3 SV=1
      Observed   Mr(expt)   Mr(calc)   ppm  Start   End Miss Ions  Peptide
     1105.6346  1104.6273  1104.5928   31.3   401 - 410   0   32   K.VTFDALGNLR.L
     1203.7272  1202.7199  1202.6771   35.6   315 - 324   1   19   K.NALDNKFIIR.C
     No match to: 1163.6599, 1320.6287, 2225.1716

8.   G0EIE2    Mass: 67211    Score: 72     Expect: 0.0023  Queries matched: 2
     G0EIE2_BRAIP Aspartyl-tRNA synthetase OS=Brachyspira intermedia (strain ATCC 51140 / PWS/A) GN=aspS PE=3 SV=1
      Observed   Mr(expt)   Mr(calc)   ppm  Start   End Miss Ions  Peptide
     1105.6346  1104.6273  1104.5928   31.3   401 - 410   0   32   K.VTFDALGNLR.L
     1203.7272  1202.7199  1202.6771   35.6   315 - 324   1   19   K.NALDNKFIIR.C
     No match to: 1163.6599, 1320.6287, 2225.1716

9.   L8XTL3    Mass: 67173    Score: 72     Expect: 0.0023  Queries matched: 2
     L8XTL3_9SPIR Aspartyl-tRNA synthetase OS=Brachyspira hampsonii 30599 GN=H263_08879 PE=3 SV=1
      Observed   Mr(expt)   Mr(calc)   ppm  Start   End Miss Ions  Peptide
     1105.6346  1104.6273  1104.5928   31.3   401 - 410   0   32   K.VTFDALGNLR.L
     1203.7272  1202.7199  1202.6771   35.6   315 - 324   1   19   K.NALDNKFIIR.C
     No match to: 1163.6599, 1320.6287, 2225.1716

10.  L0X5G2    Mass: 67255    Score: 72     Expect: 0.0023  Queries matched: 2
     L0X5G2_9SPIR Aspartyl-tRNA synthetase OS=Brachyspira hampsonii 30446 GN=A966_01371 PE=3 SV=1
      Observed   Mr(expt)   Mr(calc)   ppm  Start   End Miss Ions  Peptide
     1105.6346  1104.6273  1104.5928   31.3   401 - 410   0   32   K.VTFDALGNLR.L
     1203.7272  1202.7199  1202.6771   35.6   315 - 324   1   19   K.NALDNKFIIR.C
     No match to: 1163.6599, 1320.6287, 2225.1716

11.  L8XPV1    Mass: 12037    Score: 38     Expect: 6.9  Queries matched: 2
     L8XPV1_9SPIR Cupin domain-containing protein OS=Brachyspira hampsonii 30599 GN=H263_06662 PE=4 SV=1
      Observed   Mr(expt)   Mr(calc)   ppm  Start   End Miss Ions  Peptide
     1163.6599  1162.6526  1162.6267   22.3    35 -  45   0   ---   K.SLVMSIISADK.D
     2225.1716  2224.1643  2224.1587   2.52    46 -  66   1   ---   K.DKEIPTHTSTGDVLVTVIDGK.A
     No match to: 1105.6346, 1203.7272, 1320.6287
```

Search Parameters

```
Type of search         : Sequence Query
Enzyme                 : Trypsin
Fixed modifications    : Carbamidomethyl (C)
Variable modifications : Oxidation (M)
Mass values            : Monoisotopic
Protein Mass           : Unrestricted
Peptide Mass Tolerance : ± 50 ppm
Fragment Mass Tolerance: ± 0.5 Da
Max Missed Cleavages   : 1
Instrument type        : MALDI-TOF-TOF
Query1 (1105.6346,1+)  : Locus:7.1.1.212741.57029
Query2 (1163.6599,1+)  : Locus:7.1.1.212741.57030
Query3 (1203.7272,1+)  : Locus:7.1.1.212741.57032
Query4 (1320.6287,1+)  : Locus:7.1.1.212741.57033
Query5 (2225.1716,1+)  : Locus:7.1.1.212741.57031
```

Mascot: http://www.matrixscience.com/

Mascot Search Results

User :
Email :
Search title : F:\ROD_inmunoproteoma\MALDI\ppw_2016_561_562_563_565\ppw_H13_145467559325.txt
MS data file : F:\ROD_inmunoproteoma\MALDI\ppw_2016_561_562_563_565\ppw_H13_145467559325.txt
Database : Brachyspiraceae Brachyspiraceae_20160127-151122 (40573 sequences; 13470793 residues)
Timestamp : 23 Feb 2016 at 16:37:15 GMT
Warning : A Peptide summary report will usually give a much clearer picture of MS/MS search results.
Top Score : 272 for A0A0H0UN97, A0A0H0UN97_BRAHO Phosphoenolpyruvate carboxykinase [GTP] OS=Brachyspira hyodysenteriae GN=pckG PE=3 SV

Probability Based Mowse Score

Protein score is -10*Log(P), where P is the probability that the observed match is a random event.
Protein scores greater than 59 are significant (p<0.05).
Protein scores are derived from ions scores as a non-probabilistic basis for ranking protein hits.

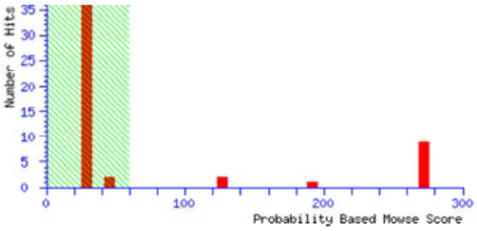

Protein Summary Report

Format As | Protein Summary (deprecated) | Help

Significance threshold p< 0.05 Max. number of hits AUTO

Standard scoring ◉ MudPIT scoring ○ Ions score or expect cut-off 0 Show sub-sets 0

Show pop-ups ◉ Suppress pop-ups ○ Sort unassigned Decreasing Score Require bold red ☐

Re-Search All | Search Unmatched

Index

	Accession	Mass	Score	Description
1.	A0A0H0UN97	66923	272	A0A0H0UN97_BRAHO Phosphoenolpyruvate carboxykinase [GTP] OS=Brachyspira hyodysenteriae GN=pckG PE=3 SV=1
2.	A0A0H0W3V6	67483	272	A0A0H0W3V6_BRAHO Phosphoenolpyruvate carboxykinase [GTP] OS=Brachyspira hyodysenteriae GN=pckG PE=3 SV=1
3.	A0A0G4K843	67513	272	A0A0G4K843_9SPIR Phosphoenolpyruvate carboxykinase [GTP] OS=Brachyspira suanatina GN=pckG PE=3 SV=1
4.	C0R0E5	67483	272	C0R0E5_BRAHW Phosphoenolpyruvate carboxykinase [GTP] OS=Brachyspira hyodysenteriae (strain ATCC 49526 / WA1) G
5.	G0ELG2	67542	272	G0ELG2_BRAIP Phosphoenolpyruvate carboxykinase [GTP] OS=Brachyspira intermedia (strain ATCC 51140 / PWS/A) GN=
6.	J9UPD6	67388	272	J9UPD6_BRAPL Phosphoenolpyruvate carboxykinase [GTP] OS=Brachyspira pilosicoli B2904 GN=pckG PE=3 SV=1
7.	D8IFS5	67465	272	D8IFS5_BRAP9 Phosphoenolpyruvate carboxykinase [GTP] OS=Brachyspira pilosicoli (strain ATCC BAA-1826 / 95/1000
8.	K0JJV6	67371	272	K0JJV6_BRAPL Phosphoenolpyruvate carboxykinase [GTP] OS=Brachyspira pilosicoli WesB GN=pckG PE=3 SV=1
9.	L0X1S2	67509	272	L0X1S2_9SPIR Phosphoenolpyruvate carboxykinase [GTP] OS=Brachyspira hampsonii 30446 GN=pckG PE=3 SV=1
10.	R5LL43	67626	197	R5LL43_9SPIR Phosphoenolpyruvate carboxykinase [GTP] OS=Brachyspira sp. CAG:700 GN=pckG PE=3 SV=1
11.	D5U5S2	67457	129	D5U5S2_BRAM5 Phosphoenolpyruvate carboxykinase [GTP] OS=Brachyspira murdochii (strain ATCC 51284 / DSM 12563 /
12.	L8XUY8	67488	129	L8XUY8_9SPIR Phosphoenolpyruvate carboxykinase [GTP] OS=Brachyspira hampsonii 30599 GN=pckG PE=3 SV=1
13.	C0QYR6	6386	42	C0QYR6_BRAHW Uncharacterized protein OS=Brachyspira hyodysenteriae (strain ATCC 49526 / WA1) GN=BHWA1_00508 PE

Results List

1. A0A0H0UN97 **Mass:** 66923 **Score:** 272 **Expect:** 2.6e-23 **Queries matched:** 3
A0A0H0UN97_BRAHO Phosphoenolpyruvate carboxykinase [GTP] OS=Brachyspira hyodysenteriae GN=pckG PE=3 SV=1

Observed	Mr(expt)	Mr(calc)	ppm	Start	End	Miss	Ions	Peptide
1023.5614	1022.5541	1022.5158	37.4	56 -	63	0	60	K.RPHSHSFR.S
1144.6373	1143.6300	1143.5865	38.0	480 -	487	0	48	R.IFYVNWFR.K
1820.9037	1819.8964	1819.8278	37.7	489 -	504	1	127	K.DADGKWLWPGFGDNSR.V

No match to: 1320.6344, 1765.7992

2. A0A0H0W3V6 **Mass:** 67483 **Score:** 272 **Expect:** 2.6e-23 **Queries matched:** 3
A0A0H0W3V6_BRAHO Phosphoenolpyruvate carboxykinase [GTP] OS=Brachyspira hyodysenteriae GN=pckG PE=3 SV=1

Observed	Mr(expt)	Mr(calc)	ppm	Start	End	Miss	Ions	Peptide
1023.5614	1022.5541	1022.5158	37.4	56 -	63	0	60	K.RPHSHSFR.S
1144.6373	1143.6300	1143.5865	38.0	486 -	493	0	48	R.IFYVNWFR.K
1820.9037	1819.8964	1819.8278	37.7	495 -	510	1	127	K.DADGKWLWPGFGDNSR.V

No match to: 1320.6344, 1765.7992

3. A0A0G4K843 **Mass:** 67513 **Score:** 272 **Expect:** 2.6e-23 **Queries matched:** 3
A0A0G4K843_9SPIR Phosphoenolpyruvate carboxykinase [GTP] OS=Brachyspira suanatina GN=pckG PE=3 SV=1

Observed	Mr(expt)	Mr(calc)	ppm	Start	End	Miss	Ions	Peptide
1023.5614	1022.5541	1022.5158	37.4	56 -	63	0	60	K.RPHSHSFR.S
1144.6373	1143.6300	1143.5865	38.0	486 -	493	0	48	R.IFYVNWFR.K
1820.9037	1819.8964	1819.8278	37.7	495 -	510	1	127	K.DADGKWLWPGFGDNSR.V

No match to: 1320.6344, 1765.7992

4. C0R0E5 **Mass:** 67483 **Score:** 272 **Expect:** 2.6e-23 **Queries matched:** 3
C0R0E5_BRAHW Phosphoenolpyruvate carboxykinase [GTP] OS=Brachyspira hyodysenteriae (strain ATCC 49526 / WA1) GN=pckG PE=3 SV=1

Observed	Mr(expt)	Mr(calc)	ppm	Start	End	Miss	Ions	Peptide
1023.5614	1022.5541	1022.5158	37.4	56 -	63	0	60	K.RPHSHSFR.S
1144.6373	1143.6300	1143.5865	38.0	486 -	493	0	48	R.IFYVNWFR.K
1820.9037	1819.8964	1819.8278	37.7	495 -	510	1	127	K.DADGKWLWPGFGDNSR.V

No match to: 1320.6344, 1765.7992

5. G0ELG2 **Mass:** 67542 **Score:** 272 **Expect:** 2.6e-23 **Queries matched:** 3
G0ELG2_BRAIP Phosphoenolpyruvate carboxykinase [GTP] OS=Brachyspira intermedia (strain ATCC 51140 / PWS/A) GN=pckG PE=3 SV=1

Observed	Mr(expt)	Mr(calc)	ppm	Start	End	Miss	Ions	Peptide
1023.5614	1022.5541	1022.5158	37.4	56 -	63	0	60	K.RPHSHSFR.S
1144.6373	1143.6300	1143.5865	38.0	486 -	493	0	48	R.IFYVNWFR.K
1820.9037	1819.8964	1819.8278	37.7	495 -	510	1	127	K.DADGKWLWPGFGDNSR.V

No match to: 1320.6344, 1765.7992

6. J9UPD6 **Mass:** 67388 **Score:** 272 **Expect:** 2.6e-23 **Queries matched:** 3
J9UPD6_BRAPL Phosphoenolpyruvate carboxykinase [GTP] OS=Brachyspira pilosicoli B2904 GN=pckG PE=3 SV=1

Observed	Mr(expt)	Mr(calc)	ppm	Start	End	Miss	Ions	Peptide
1023.5614	1022.5541	1022.5158	37.4	56 -	63	0	60	K.RPHSHSFR.S
1144.6373	1143.6300	1143.5865	38.0	486 -	493	0	48	R.IFYVNWFR.K
1820.9037	1819.8964	1819.8278	37.7	495 -	510	1	127	K.DADGKWLWPGFGDNSR.V

No match to: 1320.6344, 1765.7992

7. D8IFS5 **Mass:** 67465 **Score:** 272 **Expect:** 2.6e-23 **Queries matched:** 3
D8IFS5_BRAP9 Phosphoenolpyruvate carboxykinase [GTP] OS=Brachyspira pilosicoli (strain ATCC BAA-1826 / 95/1000) GN=pckG PE=3 SV=1

Observed	Mr(expt)	Mr(calc)	ppm	Start	End	Miss	Ions	Peptide
1023.5614	1022.5541	1022.5158	37.4	56 -	63	0	60	K.RPHSHSFR.S
1144.6373	1143.6300	1143.5865	38.0	486 -	493	0	48	R.IFYVNWFR.K
1820.9037	1819.8964	1819.8278	37.7	495 -	510	1	127	K.DADGKWLWPGFGDNSR.V

No match to: 1320.6344, 1765.7992

8. K0JJV6 **Mass:** 67371 **Score:** 272 **Expect:** 2.6e-23 **Queries matched:** 3
K0JJV6_BRAPL Phosphoenolpyruvate carboxykinase [GTP] OS=Brachyspira pilosicoli WesB GN=pckG PE=3 SV=1

Observed	Mr(expt)	Mr(calc)	ppm	Start	End	Miss	Ions	Peptide
1023.5614	1022.5541	1022.5158	37.4	56 -	63	0	60	K.RPHSHSFR.S
1144.6373	1143.6300	1143.5865	38.0	486 -	493	0	48	R.IFYVNWFR.K
1820.9037	1819.8964	1819.8278	37.7	495 -	510	1	127	K.DADGKWLWPGFGDNSR.V

No match to: 1320.6344, 1765.7992

9. L0X1S2 **Mass:** 67509 **Score:** 272 **Expect:** 2.6e-23 **Queries matched:** 3
L0X1S2_9SPIR Phosphoenolpyruvate carboxykinase [GTP] OS=Brachyspira hampsonii 30446 GN=pckG PE=3 SV=1

Observed	Mr(expt)	Mr(calc)	ppm	Start	End	Miss	Ions	Peptide
1023.5614	1022.5541	1022.5158	37.4	56 -	63	0	60	K.RPHSHSFR.S
1144.6373	1143.6300	1143.5865	38.0	486 -	493	0	48	K.IFYVNWFR.K
1820.9037	1819.8964	1819.8278	37.7	495 -	510	1	127	K.DADGKWLWPGFGDNSR.V

No match to: 1320.6344, 1765.7992

10. R5LL43 **Mass:** 67626 **Score:** 197 **Expect:** 8.1e-16 **Queries matched:** 2
R5LL43_9SPIR Phosphoenolpyruvate carboxykinase [GTP] OS=Brachyspira sp. CAG:700 GN=pckG PE=3 SV=1

Observed	Mr(expt)	Mr(calc)	ppm	Start	End	Miss	Ions	Peptide
1144.6373	1143.6300	1143.5865	38.0	485 -	492	0	48	K.IFYVNWFR.K
1820.9037	1819.8964	1819.8278	37.7	494 -	509	1	127	K.DADGKWLWPGFGDNSR.V

No match to: 1023.5614, 1320.6344, 1765.7992

11. D5U5S2 **Mass:** 67457 **Score:** 129 **Expect:** 5.1e-09 **Queries matched:** 2
D5U5S2_BRAM5 Phosphoenolpyruvate carboxykinase [GTP] OS=Brachyspira murdochii (strain ATCC 51284 / DSM 12563 / 56-150) GN=pckG PE=3 SV

Observed	Mr(expt)	Mr(calc)	ppm	Start	End	Miss	Ions	Peptide
1023.5614	1022.5541	1022.5158	37.4	56 -	63	0	60	K.RPHSHSFR.S
1144.6373	1143.6300	1143.5865	38.0	486 -	493	0	48	R.IFYVNWFR.K

No match to: 1320.6344, 1765.7992, 1820.9037

12. L8XUY8 **Mass:** 67488 **Score:** 129 **Expect:** 5.1e-09 **Queries matched:** 2
L8XUY8_9SPIR Phosphoenolpyruvate carboxykinase [GTP] OS=Brachyspira hampsonii 30599 GN=pckG PE=3 SV=1

Observed	Mr(expt)	Mr(calc)	ppm	Start	End	Miss	Ions	Peptide
1023.5614	1022.5541	1022.5158	37.4	56 -	63	0	60	K.RPHSHSFR.S
1144.6373	1143.6300	1143.5865	38.0	486 -	493	0	48	R.IFYVNWFR.K

No match to: 1320.6344, 1765.7992, 1820.9037

13. C0QYR6 **Mass:** 6386 **Score:** 42 **Expect:** 2.6 **Queries matched:** 2
C0QYR6_BRAHW Uncharacterized protein OS=Brachyspira hyodysenteriae (strain ATCC 49526 / WA1) GN=BHWA1_00508 PE=4 SV=1

Observed	Mr(expt)	Mr(calc)	ppm	Start	End	Miss	Ions	Peptide
1144.6373	1143.6300	1143.6328	-2.44	44 -	52	1	---	K.IYFLIGYKQ.-
1820.9037	1819.8964	1819.8967	-0.16	37 -	51	1	---	K.HGDMYGKIYFLIGYK.Q + Oxidation (M)

No match to: 1023.5614, 1320.6344, 1765.7992

Search Parameters

Type of search : Sequence Query
Enzyme : Trypsin
Fixed modifications : Carbamidomethyl (C)
Variable modifications : Oxidation (M)
Mass values : Monoisotopic
Protein Mass : Unrestricted
Peptide Mass Tolerance : ± 50 ppm
Fragment Mass Tolerance: ± 0.5 Da
Max Missed Cleavages : 1
Instrument type : MALDI-TOF-TOF
Query1 (1023.5614,1+) : Locus:13.1.1.212747.57059
Query2 (1144.6373,1+) : Locus:13.1.1.212747.57060

```
Query3 (1320.6344,1+)  : Locus:13.1.1.212747.57062
Query4 (1765.7992,1+)  : Locus:13.1.1.212747.57063
Query5 (1820.9037,1+)  : Locus:13.1.1.212747.57061
```

Mascot: http://www.matrixscience.com/

MATRIX SCIENCE Mascot Search Results

User	:	
Email	:	
Search title	:	F:\ROD_inmunoproteoma\MALDI\ppw_2014_442_ROD\ppw_H10_139601422133.txt
MS data file	:	F:\ROD_inmunoproteoma\MALDI\ppw_2014_442_ROD\ppw_H10_139601422133.txt
Database	:	Brachyspiraceae Brachyspiraceae_20160127-151122 (40573 sequences; 13470793 residues)
Timestamp	:	22 Feb 2016 at 15:15:12 GMT
Warning	:	A Peptide summary report will usually give a much clearer picture of MS/MS search results.
Top Score	:	153 for A0A0H0V274, A0A0H0V274_BRAHO Biotin attachment protein OS=Brachyspira hyodysenteriae GN=SZ42_00235 PE=4 SV=1

Probability Based Mowse Score

Protein score is -10*Log(P), where P is the probability that the observed match is a random event.
Protein scores greater than 59 are significant (p<0.05).
Protein scores are derived from ions scores as a non-probabilistic basis for ranking protein hits.

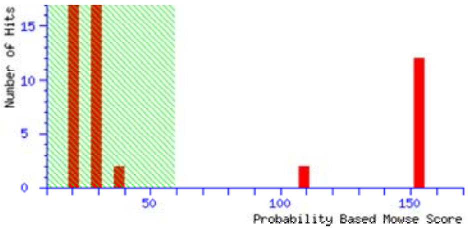

Protein Summary Report

Format As | Protein Summary (deprecated) | Help

Significance threshold p< 0.05 | Max. number of hits AUTO

Standard scoring ◉ MudPIT scoring ○ | Ions score or expect cut-off 0 | Show sub-sets 0

Show pop-ups ◉ Suppress pop-ups ○ | Sort unassigned Decreasing Score | Require bold red ☐

Re-Search All | Search Unmatched

Index

	Accession	Mass	Score	Description
1.	A0A0H0V274	64689	153	A0A0H0V274_BRAHO Biotin attachment protein OS=Brachyspira hyodysenteriae GN=SZ42_00235 PE=4 SV=1
2.	A0A0H0XA98	64689	153	A0A0H0XA98_BRAHO Biotin attachment protein OS=Brachyspira hyodysenteriae GN=SU46_04095 PE=4 SV=1
3.	A0A0H0TY53	64689	153	A0A0H0TY53_BRAHO Biotin attachment protein OS=Brachyspira hyodysenteriae GN=SR30_05280 PE=4 SV=1
4.	C0QY07	64689	153	C0QY07_BRAHW Biotin/lipoyl attachment domain-containing protein OS=Brachyspira hyodysenteriae (strain ATCC 495
5.	A0A0H0V9F5	64703	153	A0A0H0V9F5_BRAHO Biotin attachment protein OS=Brachyspira hyodysenteriae GN=SZ48_06550 PE=4 SV=1
6.	G0EPB7	64659	153	G0EPB7_BRAIP Biotin/lipoyl attachment domain-containing protein OS=Brachyspira intermedia (strain ATCC 51140 /
7.	J9UGL5	64816	153	J9UGL5_BRAPL Biotin/lipoyl attachment domain-containing protein OS=Brachyspira pilosicoli B2904 GN=B2904_orf13
8.	D8I9T6	64972	153	D8I9T6_BRAP9 Biotin/lipoyl attachment domain-containing protein OS=Brachyspira pilosicoli (strain ATCC BAA-182
9.	K0JKW5	64928	153	K0JKW5_BRAPL Biotin/lipoyl attachment domain-containing protein OS=Brachyspira pilosicoli WesB GN=WESB_1472 PE
10.	L0X024	64721	153	L0X024_9SPIR Pyruvate carboxylase OS=Brachyspira hampsonii 30446 GN=A966_11217 PE=4 SV=1
11.	L8XSV2	64877	153	L8XSV2_9SPIR Pyruvate carboxylase OS=Brachyspira hampsonii 30599 GN=H263_10407 PE=4 SV=1
12.	D5U4C9	64969	153	D5U4C9_BRAM5 Pyruvate carboxylase OS=Brachyspira murdochii (strain ATCC 51284 / DSM 12563 / 56-150) GN=Bmur_00
13.	R5LE51	65062	113	R5LE51_9SPIR Biotin/lipoyl attachment domain-containing protein OS=Brachyspira sp. CAG:700 GN=BN758_02112 PE=4
14.	A0A0G4K6M6	64677	113	A0A0G4K6M6_9SPIR Biotin attachment protein OS=Brachyspira suanatina GN=BRSU_1275 PE=4 SV=1
15.	L0X2N0	27890	35	L0X2N0_9SPIR Transcription-repair coupling factor OS=Brachyspira hampsonii 30446 GN=A966_08499 PE=4 SV=1

Results List

1. A0A0H0V274 **Mass:** 64689 **Score:** 153 **Expect:** 2e-11 **Queries matched:** 3
A0A0H0V274_BRAHO Biotin attachment protein OS=Brachyspira hyodysenteriae GN=SZ42_00235 PE=4 SV=1

Observed	Mr(expt)	Mr(calc)	ppm	Start	End	Miss	Ions	Peptide
1254.7012	1253.6939	1253.6728	16.8	87 -	98	0	46	R.GVNVVGLESQPR.E
1383.7887	1382.7814	1382.7518	21.4	74 -	86	0	20	K.TVGPDVNLQTLAR.G
1574.8707	1573.8634	1573.8286	22.1	320 -	332	1	39	R.DINVMNRFPEVIK.A

2. A0A0H0XA98 **Mass:** 64689 **Score:** 153 **Expect:** 2e-11 **Queries matched:** 3
A0A0H0XA98_BRAHO Biotin attachment protein OS=Brachyspira hyodysenteriae GN=SU46_04095 PE=4 SV=1

Observed	Mr(expt)	Mr(calc)	ppm	Start	End	Miss	Ions	Peptide
1254.7012	1253.6939	1253.6728	16.8	87 -	98	0	46	R.GVNVVGLESQPR.E
1383.7887	1382.7814	1382.7518	21.4	74 -	86	0	20	K.TVGPDVNLQTLAR.G
1574.8707	1573.8634	1573.8286	22.1	320 -	332	1	39	R.DINVMNRFPEVIK.A

3. A0A0H0TY53 **Mass:** 64689 **Score:** 153 **Expect:** 2e-11 **Queries matched:** 3
A0A0H0TY53_BRAHO Biotin attachment protein OS=Brachyspira hyodysenteriae GN=SR30_05280 PE=4 SV=1

Observed	Mr(expt)	Mr(calc)	ppm	Start	End	Miss	Ions	Peptide
1254.7012	1253.6939	1253.6728	16.8	87 -	98	0	46	R.GVNVVGLESQPR.E
1383.7887	1382.7814	1382.7518	21.4	74 -	86	0	20	K.TVGPDVNLQTLAR.G
1574.8707	1573.8634	1573.8286	22.1	320 -	332	1	39	R.DINVMNRFPEVIK.A

4. C0QY07 **Mass:** 64689 **Score:** 153 **Expect:** 2e-11 **Queries matched:** 3
C0QY07_BRAHW Biotin/lipoyl attachment domain-containing protein OS=Brachyspira hyodysenteriae (strain ATCC 49526 / WA1) GN=BHWA1_0038(

Observed	Mr(expt)	Mr(calc)	ppm	Start	End	Miss	Ions	Peptide
1254.7012	1253.6939	1253.6728	16.8	87 -	98	0	46	R.GVNVVGLESQPR.E

1383.7887	1382.7814	1382.7518	21.4	74 - 86	0	20	K.TVGPDVNLQTLAR.G
1574.8707	1573.8634	1573.8286	22.1	320 - 332	1	39	R.DINVMNRFPEVIK.A

5. A0A0H0V9F5 **Mass:** 64703 **Score:** 153 **Expect:** 2e-11 **Queries matched:** 3
A0A0H0V9F5_BRAHO Biotin attachment protein OS=Brachyspira hyodysenteriae GN=SZ48_06550 PE=4 SV=1

Observed	Mr(expt)	Mr(calc)	ppm	Start - End	Miss	Ions	Peptide
1254.7012	1253.6939	1253.6728	16.8	87 - 98	0	46	R.GVNVVGLESQPR.E
1383.7887	1382.7814	1382.7518	21.4	74 - 86	0	20	K.TVGPDVNLQTLAR.G
1574.8707	1573.8634	1573.8286	22.1	320 - 332	1	39	R.DINVMNRFPEVIK.A

6. G0EPB7 **Mass:** 64659 **Score:** 153 **Expect:** 2e-11 **Queries matched:** 3
G0EPB7_BRAIP Biotin/lipoyl attachment domain-containing protein OS=Brachyspira intermedia (strain ATCC 51140 / PWS/A) GN=Bint_1368 PE=

Observed	Mr(expt)	Mr(calc)	ppm	Start - End	Miss	Ions	Peptide
1254.7012	1253.6939	1253.6728	16.8	87 - 98	0	46	R.GVNVVGLESQPR.E
1383.7887	1382.7814	1382.7518	21.4	74 - 86	0	20	K.TVGPDVNLQTLAR.G
1574.8707	1573.8634	1573.8286	22.1	320 - 332	1	39	R.DINVMNRFPEVIK.A

7. J9UGL5 **Mass:** 64816 **Score:** 153 **Expect:** 2e-11 **Queries matched:** 3
J9UGL5_BRAPL Biotin/lipoyl attachment domain-containing protein OS=Brachyspira pilosicoli B2904 GN=B2904_orf1329 PE=4 SV=1

Observed	Mr(expt)	Mr(calc)	ppm	Start - End	Miss	Ions	Peptide
1254.7012	1253.6939	1253.6728	16.8	87 - 98	0	46	R.GVNVVGLESQPR.D
1383.7887	1382.7814	1382.7518	21.4	74 - 86	0	20	K.TVGPDVNLQTLAR.G
1574.8707	1573.8634	1573.8286	22.1	320 - 332	1	39	R.DINVMNRFPEVIK.A

8. D8I9T6 **Mass:** 64972 **Score:** 153 **Expect:** 2e-11 **Queries matched:** 3
D8I9T6_BRAP9 Biotin/lipoyl attachment domain-containing protein OS=Brachyspira pilosicoli (strain ATCC BAA-1826 / 95/1000) GN=BP95100C

Observed	Mr(expt)	Mr(calc)	ppm	Start - End	Miss	Ions	Peptide
1254.7012	1253.6939	1253.6728	16.8	87 - 98	0	46	R.GVNVVGLESQPR.D
1383.7887	1382.7814	1382.7518	21.4	74 - 86	0	20	K.TVGPDVNLQTLAR.G
1574.8707	1573.8634	1573.8286	22.1	320 - 332	1	39	R.DINVMNRFPEVIK.A

9. K0JKW5 **Mass:** 64928 **Score:** 153 **Expect:** 2e-11 **Queries matched:** 3
K0JKW5_BRAPL Biotin/lipoyl attachment domain-containing protein OS=Brachyspira pilosicoli WesB GN=WESB_1472 PE=4 SV=1

Observed	Mr(expt)	Mr(calc)	ppm	Start - End	Miss	Ions	Peptide
1254.7012	1253.6939	1253.6728	16.8	87 - 98	0	46	R.GVNVVGLESQPR.D
1383.7887	1382.7814	1382.7518	21.4	74 - 86	0	20	K.TVGPDVNLQTLAR.G
1574.8707	1573.8634	1573.8286	22.1	320 - 332	1	39	R.DINVMNRFPEVIK.A

10. L0X024 **Mass:** 64721 **Score:** 153 **Expect:** 2e-11 **Queries matched:** 3
L0X024_9SPIR Pyruvate carboxylase OS=Brachyspira hampsonii 30446 GN=A966_11217 PE=4 SV=1

Observed	Mr(expt)	Mr(calc)	ppm	Start - End	Miss	Ions	Peptide
1254.7012	1253.6939	1253.6728	16.8	87 - 98	0	46	R.GVNVVGLESQPR.E
1383.7887	1382.7814	1382.7518	21.4	74 - 86	0	20	K.TVGPDVNLQTLAR.G
1574.8707	1573.8634	1573.8286	22.1	320 - 332	1	39	R.DINVMNRFPEVIK.A

11. L8XSV2 **Mass:** 64877 **Score:** 153 **Expect:** 2e-11 **Queries matched:** 3
L8XSV2_9SPIR Pyruvate carboxylase OS=Brachyspira hampsonii 30599 GN=H263_10407 PE=4 SV=1

Observed	Mr(expt)	Mr(calc)	ppm	Start - End	Miss	Ions	Peptide
1254.7012	1253.6939	1253.6728	16.8	87 - 98	0	46	R.GVNVVGLESQPR.E
1383.7887	1382.7814	1382.7518	21.4	74 - 86	0	20	K.TVGPDVNLQTLAR.G
1574.8707	1573.8634	1573.8286	22.1	320 - 332	1	39	R.DINVMNRFPEVIK.A

12. D5U4C9 **Mass:** 64969 **Score:** 153 **Expect:** 2e-11 **Queries matched:** 3
D5U4C9_BRAM5 Pyruvate carboxylase OS=Brachyspira murdochii (strain ATCC 51284 / DSM 12563 / 56-150) GN=Bmur_0063 PE=4 SV=1

Observed	Mr(expt)	Mr(calc)	ppm	Start - End	Miss	Ions	Peptide
1254.7012	1253.6939	1253.6728	16.8	87 - 98	0	46	R.GVNVVGLESQPR.E
1383.7887	1382.7814	1382.7518	21.4	74 - 86	0	20	K.TVGPDVNLQTLAR.G
1574.8707	1573.8634	1573.8286	22.1	320 - 332	1	39	R.DINVMNRFPEVIK.A

13. R5LE51 **Mass:** 65062 **Score:** 113 **Expect:** 2e-07 **Queries matched:** 2
R5LE51_9SPIR Biotin/lipoyl attachment domain-containing protein OS=Brachyspira sp. CAG:700 GN=BN758_02112 PE=4 SV=1

Observed	Mr(expt)	Mr(calc)	ppm	Start - End	Miss	Ions	Peptide
1254.7012	1253.6939	1253.6728	16.8	87 - 98	0	46	R.GVNVVGLESQPR.E
1574.8707	1573.8634	1573.8286	22.1	320 - 332	1	39	R.DINVMNRFPEVIK.A

No match to: 1383.7887

14. A0A0G4K6M6 **Mass:** 64677 **Score:** 113 **Expect:** 2e-07 **Queries matched:** 2
A0A0G4K6M6_9SPIR Biotin attachment protein OS=Brachyspira suanatina GN=BRSU_1275 PE=4 SV=1

Observed	Mr(expt)	Mr(calc)	ppm	Start - End	Miss	Ions	Peptide
1254.7012	1253.6939	1253.6728	16.8	87 - 98	0	46	R.GVNVVGLESQPR.E
1574.8707	1573.8634	1573.8286	22.1	320 - 332	1	39	R.DINVMNRFPEVIK.A

No match to: 1383.7887

15. L0X2N0 **Mass:** 27890 **Score:** 35 **Expect:** 13 **Queries matched:** 2
L0X2N0_9SPIR Transcription-repair coupling factor OS=Brachyspira hampsonii 30446 GN=A966_08499 PE=4 SV=1

Observed	Mr(expt)	Mr(calc)	ppm	Start - End	Miss	Ions	Peptide
1254.7012	1253.6939	1253.6689	19.9	133 - 143	1	---	K.IESYLIGGKMK.M + Oxidation (M)
1574.8707	1573.8634	1573.8862	-14.44	144 - 159	0	---	K.MLTGALVGASAVLSIR.N + Oxidation (M)

No match to: 1383.7887

Search Parameters

Type of search : Sequence Query
Enzyme : Trypsin
Fixed modifications : Carbamidomethyl (C)
Variable modifications : Oxidation (M)
Mass values : Monoisotopic

```
Protein Mass           : Unrestricted
Peptide Mass Tolerance : ± 50 ppm
Fragment Mass Tolerance: ± 0.5 Da
Max Missed Cleavages   : 1
Instrument type        : MALDI-TOF-TOF
Query1 (1254.7012,1+)  : Locus:34.1.1.170632.52836
Query2 (1383.7887,1+)  : Locus:34.1.1.170632.52835
Query3 (1574.8707,1+)  : Locus:34.1.1.170632.52834
```

**Mascot:** http://www.matrixscience.com/

Mascot Search Results

```
User          :
Email         :
Search title  : F:\ROD_inmunoproteoma\MALDI\160223_2016_561_562_ROD\ppw_F4_145631467503.txt
MS data file  : F:\ROD_inmunoproteoma\MALDI\160223_2016_561_562_ROD\ppw_F4_145631467503.txt
Database      : Brachyspiraceae Brachyspiraceae_20160127-151122 (40573 sequences; 13470793 residues)
Timestamp     : 24 Feb 2016 at 12:02:30 GMT
Warning       : A Peptide summary report will usually give a much clearer picture of MS/MS search results.
Top Score     : 156 for A0A0H0V6M8, A0A0H0V6M8_BRAHO 60 kDa chaperonin OS=Brachyspira hyodysenteriae GN=groEL PE=3 SV=1
```

Probability Based Mowse Score

Protein score is -10*Log(P), where P is the probability that the observed match is a random event.
Protein scores greater than 59 are significant (p<0.05).
Protein scores are derived from ions scores as a non-probabilistic basis for ranking protein hits.

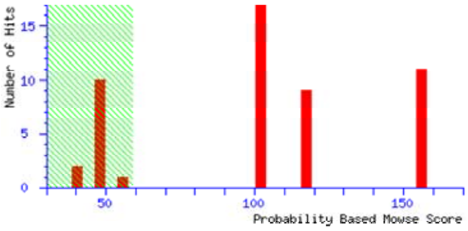

Protein Summary Report

Format As | Protein Summary (deprecated) | Help

Significance threshold p< 0.05 | Max. number of hits AUTO

Standard scoring ◉ MudPIT scoring ○ | Ions score or expect cut-off 0 | Show sub-sets 0

Show pop-ups ◉ Suppress pop-ups ○ | Sort unassigned Decreasing Score | Require bold red ☐

Re-Search All | Search Unmatched

Index

	Accession	Mass	Score	Description
1.	A0A0H0V6M8	58212	156	A0A0H0V6M8_BRAHO 60 kDa chaperonin OS=Brachyspira hyodysenteriae GN=groEL PE=3 SV=1
2.	A0A0H0TIS1	58228	156	A0A0H0TIS1_BRAHO 60 kDa chaperonin OS=Brachyspira hyodysenteriae GN=groEL PE=3 SV=1
3.	A0A0G4K5P2	58212	156	A0A0G4K5P2_9SPIR 60 kDa chaperonin OS=Brachyspira suanatina GN=groL PE=3 SV=1
4.	C0QWM4	58212	156	C0QWM4_BRAHW 60 kDa chaperonin OS=Brachyspira hyodysenteriae (strain ATCC 49526 / WA1) GN=groL PE=3 SV=1
5.	G0EJ80	58197	156	G0EJ80_BRAIP 60 kDa chaperonin OS=Brachyspira intermedia (strain ATCC 51140 / PWS/A) GN=groL PE=3 SV=1
6.	D5U544	58148	156	D5U544_BRAM5 60 kDa chaperonin OS=Brachyspira murdochii (strain ATCC 51284 / DSM 12563 / 56-150) GN=groL PE=3
7.	L8XV88	58195	156	L8XV88_9SPIR 60 kDa chaperonin OS=Brachyspira hampsonii 30599 GN=groL PE=3 SV=1
8.	J9USS2	58110	154	J9USS2_BRAPL 60 kDa chaperonin OS=Brachyspira pilosicoli B2904 GN=groL PE=3 SV=1
9.	D8IB78	58110	154	D8IB78_BRAP9 60 kDa chaperonin OS=Brachyspira pilosicoli (strain ATCC BAA-1826 / 95/1000) GN=groL PE=3 SV=1
10.	K0JLL7	58110	154	K0JLL7_BRAPL 60 kDa chaperonin OS=Brachyspira pilosicoli WesB GN=groL PE=3 SV=1
11.	L0X051	58181	154	L0X051_9SPIR 60 kDa chaperonin OS=Brachyspira hampsonii 30446 GN=groL PE=3 SV=1
12.	T1WGB0	20074	121	T1WGB0_9SPIR 60 kDa chaperonin (Fragment) OS=Brachyspira sp. CH-003 GN=cpn60 PE=4 SV=1
13.	T1WFK2	20144	121	T1WFK2_9SPIR 60 kDa chaperonin (Fragment) OS=Brachyspira sp. SHI-129 GN=cpn60 PE=4 SV=1
14.	T1WGB4	20074	121	T1WGB4_9SPIR 60 kDa chaperonin (Fragment) OS=Brachyspira hampsonii bv. II GN=cpn60 PE=4 SV=1
15.	T1WFJ6	20144	121	T1WFJ6_9SPIR 60 kDa chaperonin (Fragment) OS=Brachyspira sp. SHI-031 GN=cpn60 PE=4 SV=1
16.	Q3YLA3	19965	121	Q3YLA3_BRAPL 60 kDa chaperonin (Fragment) OS=Brachyspira pilosicoli GN=cpn60 PE=4 SV=1
17.	R5LET5	65397	116	R5LET5_9SPIR V-type ATP synthase subunit A OS=Brachyspira sp. CAG:700 GN=BN758_00162 PE=4 SV=1
18.	J9TR55	65599	116	J9TR55_BRAPL V-type ATP synthase subunit A OS=Brachyspira pilosicoli B2904 GN=B2904_orf311 PE=4 SV=1
19.	D8ID04	65599	116	D8ID04_BRAP9 V-type ATP synthase subunit A OS=Brachyspira pilosicoli (strain ATCC BAA-1826 / 95/1000) GN=BP951
20.	K0JMS2	65585	116	K0JMS2_BRAPL V-type ATP synthase subunit A OS=Brachyspira pilosicoli WesB GN=atpA2 PE=4 SV=1
21.	T1WGB9	20103	103	T1WGB9_9SPIR 60 kDa chaperonin (Fragment) OS=Brachyspira sp. SHI-067 GN=cpn60 PE=4 SV=1
22.	Q3YL98	20103	103	Q3YL98_9SPIR 60 kDa chaperonin (Fragment) OS=Brachyspira murdochii GN=cpn60 PE=4 SV=1
23.	T1WFJ0	20174	103	T1WFJ0_9SPIR 60 kDa chaperonin (Fragment) OS=Brachyspira sp. KL-169 GN=cpn60 PE=4 SV=1
24.	Q3YLA1	20036	103	Q3YLA1_BRAHO 60 kDa chaperonin (Fragment) OS=Brachyspira hyodysenteriae GN=cpn60 PE=4 SV=1
25.	T1WF97	20097	103	T1WF97_9SPIR 60 kDa chaperonin (Fragment) OS=Brachyspira sp. KL-181 GN=cpn60 PE=4 SV=1
26.	T1WF92	20075	103	T1WF92_9SPIR 60 kDa chaperonin (Fragment) OS=Brachyspira sp. CH-013 GN=cpn60 PE=4 SV=1
27.	Q3YLA0	20083	103	Q3YLA0_9SPIR 60 kDa chaperonin (Fragment) OS=Brachyspira innocens GN=cpn60 PE=4 SV=1
28.	T1WEF0	20103	103	T1WEF0_9SPIR 60 kDa chaperonin (Fragment) OS=Brachyspira sp. CH-014 GN=cpn60 PE=4 SV=1
29.	Q3YL99	20091	103	Q3YL99_9SPIR 60 kDa chaperonin (Fragment) OS=Brachyspira alvinipulli GN=cpn60 PE=4 SV=1
30.	T1WEW3	20103	103	T1WEW3_9SPIR 60 kDa chaperonin (Fragment) OS=Brachyspira sp. KL-207 GN=cpn60 PE=4 SV=1
31.	T1WFA1	20103	103	T1WFA1_9SPIR 60 kDa chaperonin (Fragment) OS=Brachyspira sp. SHI-068 GN=cpn60 PE=4 SV=1
32.	G1EI49	20092	103	G1EI49_9SPIR 60 kDa chaperonin (Fragment) OS=Brachyspira intermedia GN=cpn60 PE=4 SV=1
33.	F8UU24	20092	103	F8UU24_9SPIR CPN60 (Fragment) OS=Brachyspira sp. j1330 GN=cpn60 PE=4 SV=1
34.	T1WEG1	20103	103	T1WEG1_9SPIR 60 kDa chaperonin (Fragment) OS=Brachyspira sp. CH-108 GN=cpn60 PE=4 SV=1
35.	T1WEV9	20100	103	T1WEV9_9SPIR 60 kDa chaperonin (Fragment) OS=Brachyspira sp. KL-049 GN=cpn60 PE=4 SV=1
36.	T1WEW8	20103	103	T1WEW8_9SPIR 60 kDa chaperonin (Fragment) OS=Brachyspira sp. CH-110 GN=cpn60 PE=4 SV=1
37.	T1WEF7	20075	103	T1WEF7_9SPIR 60 kDa chaperonin (Fragment) OS=Brachyspira sp. KL-194 GN=cpn60 PE=4 SV=1
38.	R5LQ94	58190	53	R5LQ94_9SPIR 60 kDa chaperonin OS=Brachyspira sp. CAG:700 GN=groL PE=3 SV=1

Results List

1. A0A0H0V6M8 **Mass:** 58212 **Score:** 156 **Expect:** 1e-11 **Queries matched:** 2
A0A0H0V6M8_BRAHO 60 kDa chaperonin OS=Brachyspira hyodysenteriae GN=groEL PE=3 SV=1

Observed	Mr(expt)	Mr(calc)	ppm	Start	End	Miss	Ions	Peptide
1276.6761	1275.6688	1275.6571	9.17	5 -	14	1	44	K.QLLFDEEARR.A
1811.9034	1810.8961	1810.8771	10.5	182 -	197	0	88	K.SLETSLSLVEGMQFDR.G

No match to: 1259.6888, 1290.6913, 1737.8358

2. A0A0H0TIS1 **Mass:** 58228 **Score:** 156 **Expect:** 1e-11 **Queries matched:** 2
A0A0H0TIS1_BRAHO 60 kDa chaperonin OS=Brachyspira hyodysenteriae GN=groEL PE=3 SV=1

Observed	Mr(expt)	Mr(calc)	ppm	Start	End	Miss	Ions	Peptide
1276.6761	1275.6688	1275.6571	9.17	5 -	14	1	44	K.QLLFDEEARR.A
1811.9034	1810.8961	1810.8771	10.5	182 -	197	0	88	K.SLETSLSLVEGMQFDR.G

No match to: 1259.6888, 1290.6913, 1737.8358

3. A0A0G4K5P2 **Mass:** 58212 **Score:** 156 **Expect:** 1e-11 **Queries matched:** 2
A0A0G4K5P2_9SPIR 60 kDa chaperonin OS=Brachyspira suanatina GN=groL PE=3 SV=1

Observed	Mr(expt)	Mr(calc)	ppm	Start	End	Miss	Ions	Peptide
1276.6761	1275.6688	1275.6571	9.17	5 -	14	1	44	K.QLLFDEEARR.A
1811.9034	1810.8961	1810.8771	10.5	182 -	197	0	88	K.SLETSLSLVEGMQFDR.G

No match to: 1259.6888, 1290.6913, 1737.8358

4. C0QWM4 **Mass:** 58212 **Score:** 156 **Expect:** 1e-11 **Queries matched:** 2
C0QWM4_BRAHW 60 kDa chaperonin OS=Brachyspira hyodysenteriae (strain ATCC 49526 / WA1) GN=groL PE=3 SV=1

Observed	Mr(expt)	Mr(calc)	ppm	Start	End	Miss	Ions	Peptide
1276.6761	1275.6688	1275.6571	9.17	5 -	14	1	44	K.QLLFDEEARR.A
1811.9034	1810.8961	1810.8771	10.5	182 -	197	0	88	K.SLETSLSLVEGMQFDR.G

No match to: 1259.6888, 1290.6913, 1737.8358

5. G0EJ80 **Mass:** 58197 **Score:** 156 **Expect:** 1e-11 **Queries matched:** 2
G0EJ80_BRAIP 60 kDa chaperonin OS=Brachyspira intermedia (strain ATCC 51140 / PWS/A) GN=groL PE=3 SV=1

Observed	Mr(expt)	Mr(calc)	ppm	Start	End	Miss	Ions	Peptide
1276.6761	1275.6688	1275.6571	9.17	5 -	14	1	44	K.QLLFDEEARR.A
1811.9034	1810.8961	1810.8771	10.5	182 -	197	0	88	K.SLETSLSLVEGMQFDR.G

No match to: 1259.6888, 1290.6913, 1737.8358

6. D5U544 **Mass:** 58148 **Score:** 156 **Expect:** 1e-11 **Queries matched:** 2
D5U544_BRAM5 60 kDa chaperonin OS=Brachyspira murdochii (strain ATCC 51284 / DSM 12563 / 56-150) GN=groL PE=3 SV=1

Observed	Mr(expt)	Mr(calc)	ppm	Start	End	Miss	Ions	Peptide
1276.6761	1275.6688	1275.6571	9.17	5 -	14	1	44	K.QLLFDEEARR.A
1811.9034	1810.8961	1810.8771	10.5	182 -	197	0	88	K.SLETSLSLVEGMQFDR.G

No match to: 1259.6888, 1290.6913, 1737.8358

7. L8XV88 **Mass:** 58195 **Score:** 156 **Expect:** 1e-11 **Queries matched:** 2
L8XV88_9SPIR 60 kDa chaperonin OS=Brachyspira hampsonii 30599 GN=groL PE=3 SV=1

Observed	Mr(expt)	Mr(calc)	ppm	Start	End	Miss	Ions	Peptide
1276.6761	1275.6688	1275.6571	9.17	5 -	14	1	44	K.QLLFDEEARR.A
1811.9034	1810.8961	1810.8771	10.5	182 -	197	0	88	K.SLETSLSLVEGMQFDR.G

No match to: 1259.6888, 1290.6913, 1737.8358

8. J9USS2 **Mass:** 58110 **Score:** 154 **Expect:** 1.6e-11 **Queries matched:** 2
J9USS2_BRAPL 60 kDa chaperonin OS=Brachyspira pilosicoli B2904 GN=groL PE=3 SV=1

Observed	Mr(expt)	Mr(calc)	ppm	Start	End	Miss	Ions	Peptide
1276.6761	1275.6688	1275.6571	9.17	5 -	14	1	44	K.QLLFDEEARR.S
1811.9034	1810.8961	1810.8771	10.5	182 -	197	0	88	K.SLETSLSLVEGMQFDR.G

No match to: 1259.6888, 1290.6913, 1737.8358

9. D8IB78 **Mass:** 58110 **Score:** 154 **Expect:** 1.6e-11 **Queries matched:** 2
D8IB78_BRAP9 60 kDa chaperonin OS=Brachyspira pilosicoli (strain ATCC BAA-1826 / 95/1000) GN=groL PE=3 SV=1

Observed	Mr(expt)	Mr(calc)	ppm	Start	End	Miss	Ions	Peptide
1276.6761	1275.6688	1275.6571	9.17	5 -	14	1	44	K.QLLFDEEARR.S
1811.9034	1810.8961	1810.8771	10.5	182 -	197	0	88	K.SLETSLSLVEGMQFDR.G

No match to: 1259.6888, 1290.6913, 1737.8358

10. K0JLL7 **Mass:** 58110 **Score:** 154 **Expect:** 1.6e-11 **Queries matched:** 2
K0JLL7_BRAPL 60 kDa chaperonin OS=Brachyspira pilosicoli WesB GN=groL PE=3 SV=1

Observed	Mr(expt)	Mr(calc)	ppm	Start	End	Miss	Ions	Peptide
1276.6761	1275.6688	1275.6571	9.17	5 -	14	1	44	K.QLLFDEEARR.S
1811.9034	1810.8961	1810.8771	10.5	182 -	197	0	88	K.SLETSLSLVEGMQFDR.G

No match to: 1259.6888, 1290.6913, 1737.8358

11. L0X051 **Mass:** 58181 **Score:** 154 **Expect:** 1.6e-11 **Queries matched:** 2
L0X051_9SPIR 60 kDa chaperonin OS=Brachyspira hampsonii 30446 GN=groL PE=3 SV=1

Observed	Mr(expt)	Mr(calc)	ppm	Start	End	Miss	Ions	Peptide
1276.6761	1275.6688	1275.6571	9.17	5 -	14	1	44	K.QLLFDEEARR.A
1811.9034	1810.8961	1810.8771	10.5	182 -	197	0	88	K.SLETSLSLVEGMQFDR.G

No match to: 1259.6888, 1290.6913, 1737.8358

12. T1WGB0 **Mass:** 20074 **Score:** 121 **Expect:** 3.2e-08 **Queries matched:** 2
T1WGB0_9SPIR 60 kDa chaperonin (Fragment) OS=Brachyspira sp. CH-003 GN=cpn60 PE=4 SV=1

Observed	Mr(expt)	Mr(calc)	ppm	Start	End	Miss	Ions	Peptide
1276.6761	1275.6688	1275.6380	24.2	66 -	77	0	---	K.EIGALISDAMEK.V
1811.9034	1810.8961	1810.8771	10.5	91 -	106	0	88	K.SLETSLSLVEGMQFDR.G

No match to: 1259.6888, 1290.6913, 1737.8358

13. T1WFK2 **Mass:** 20144 **Score:** 121 **Expect:** 3.2e-08 **Queries matched:** 2
T1WFK2_9SPIR 60 kDa chaperonin (Fragment) OS=Brachyspira sp. SHI-129 GN=cpn60 PE=4 SV=1

Observed	Mr(expt)	Mr(calc)	ppm	Start	End	Miss	Ions	Peptide
1276.6761	1275.6688	1275.6380	24.2	66 -	77	0	---	K.EIGALISDAMEK.V
1811.9034	1810.8961	1810.8771	10.5	91 -	106	0	88	K.SLETSLSLVEGMQFDR.G

No match to: 1259.6888, 1290.6913, 1737.8358

14. T1WGB4 **Mass:** 20074 **Score:** 121 **Expect:** 3.2e-08 **Queries matched:** 2

T1WGB4_9SPIR 60 kDa chaperonin (Fragment) OS=Brachyspira hampsonii bv. II GN=cpn60 PE=4 SV=1

Observed	Mr(expt)	Mr(calc)	ppm	Start	End	Miss	Ions	Peptide
1276.6761	1275.6688	1275.6380	24.2	66 -	77	0	---	K.EIGALISDAMEK.V
1811.9034	1810.8961	1810.8771	10.5	91 -	106	0	88	K.SLETSLSLVEGMQFDR.G

No match to: 1259.6888, 1290.6913, 1737.8358

15. T1WFJ6 **Mass:** 20144 **Score:** 121 **Expect:** 3.2e-08 **Queries matched:** 2
T1WFJ6_9SPIR 60 kDa chaperonin (Fragment) OS=Brachyspira sp. SHI-031 GN=cpn60 PE=4 SV=1

Observed	Mr(expt)	Mr(calc)	ppm	Start	End	Miss	Ions	Peptide
1276.6761	1275.6688	1275.6380	24.2	66 -	77	0	---	K.EIGALISDAMEK.V
1811.9034	1810.8961	1810.8771	10.5	91 -	106	0	88	K.SLETSLSLVEGMQFDR.G

No match to: 1259.6888, 1290.6913, 1737.8358

16. Q3YLA3 **Mass:** 19965 **Score:** 121 **Expect:** 3.2e-08 **Queries matched:** 2
Q3YLA3_BRAPL 60 kDa chaperonin (Fragment) OS=Brachyspira pilosicoli GN=cpn60 PE=4 SV=1

Observed	Mr(expt)	Mr(calc)	ppm	Start	End	Miss	Ions	Peptide
1276.6761	1275.6688	1275.6380	24.2	66 -	77	0	---	K.EIGALISDAMEK.V
1811.9034	1810.8961	1810.8771	10.5	91 -	106	0	88	K.SLETSLSLVEGMQFDR.G

No match to: 1259.6888, 1290.6913, 1737.8358

17. R5LET5 **Mass:** 65397 **Score:** 116 **Expect:** 1e-07 **Queries matched:** 1
R5LET5_9SPIR V-type ATP synthase subunit A OS=Brachyspira sp. CAG:700 GN=BN758_00162 PE=4 SV=1

Observed	Mr(expt)	Mr(calc)	ppm	Start	End	Miss	Ions	Peptide
1737.8358	1736.8285	1736.8080	11.8	307 -	321	0	106	R.EASVYVGMTVAEYYR.S

No match to: 1259.6888, 1276.6761, 1290.6913, 1811.9034

18. J9TR55 **Mass:** 65599 **Score:** 116 **Expect:** 1e-07 **Queries matched:** 1
J9TR55_BRAPL V-type ATP synthase subunit A OS=Brachyspira pilosicoli B2904 GN=B2904_orf311 PE=4 SV=1

Observed	Mr(expt)	Mr(calc)	ppm	Start	End	Miss	Ions	Peptide
1737.8358	1736.8285	1736.8080	11.8	307 -	321	0	106	R.EASVYVGMTVAEYYR.S

No match to: 1259.6888, 1276.6761, 1290.6913, 1811.9034

19. D8ID04 **Mass:** 65599 **Score:** 116 **Expect:** 1e-07 **Queries matched:** 1
D8ID04_BRAP9 V-type ATP synthase subunit A OS=Brachyspira pilosicoli (strain ATCC BAA-1826 / 95/1000) GN=BP951000_1036 PE=4 SV=1

Observed	Mr(expt)	Mr(calc)	ppm	Start	End	Miss	Ions	Peptide
1737.8358	1736.8285	1736.8080	11.8	307 -	321	0	106	R.EASVYVGMTVAEYYR.S

No match to: 1259.6888, 1276.6761, 1290.6913, 1811.9034

20. K0JMS2 **Mass:** 65585 **Score:** 116 **Expect:** 1e-07 **Queries matched:** 1
K0JMS2_BRAPL V-type ATP synthase subunit A OS=Brachyspira pilosicoli WesB GN=atpA2 PE=4 SV=1

Observed	Mr(expt)	Mr(calc)	ppm	Start	End	Miss	Ions	Peptide
1737.8358	1736.8285	1736.8080	11.8	307 -	321	0	106	R.EASVYVGMTVAEYYR.S

No match to: 1259.6888, 1276.6761, 1290.6913, 1811.9034

21. T1WGB9 **Mass:** 20103 **Score:** 103 **Expect:** 2e-06 **Queries matched:** 1
T1WGB9_9SPIR 60 kDa chaperonin (Fragment) OS=Brachyspira sp. SHI-067 GN=cpn60 PE=4 SV=1

Observed	Mr(expt)	Mr(calc)	ppm	Start	End	Miss	Ions	Peptide
1811.9034	1810.8961	1810.8771	10.5	91 -	106	0	88	K.SLETSLSLVEGMQFDR.G

No match to: 1259.6888, 1276.6761, 1290.6913, 1737.8358

22. Q3YL98 **Mass:** 20103 **Score:** 103 **Expect:** 2e-06 **Queries matched:** 1
Q3YL98_9SPIR 60 kDa chaperonin (Fragment) OS=Brachyspira murdochii GN=cpn60 PE=4 SV=1

Observed	Mr(expt)	Mr(calc)	ppm	Start	End	Miss	Ions	Peptide
1811.9034	1810.8961	1810.8771	10.5	91 -	106	0	88	K.SLETSLSLVEGMQFDR.G

No match to: 1259.6888, 1276.6761, 1290.6913, 1737.8358

23. T1WFJ0 **Mass:** 20174 **Score:** 103 **Expect:** 2e-06 **Queries matched:** 1
T1WFJ0_9SPIR 60 kDa chaperonin (Fragment) OS=Brachyspira sp. KL-169 GN=cpn60 PE=4 SV=1

Observed	Mr(expt)	Mr(calc)	ppm	Start	End	Miss	Ions	Peptide
1811.9034	1810.8961	1810.8771	10.5	91 -	106	0	88	K.SLETSLSLVEGMQFDR.G

No match to: 1259.6888, 1276.6761, 1290.6913, 1737.8358

24. Q3YLA1 **Mass:** 20036 **Score:** 103 **Expect:** 2e-06 **Queries matched:** 1
Q3YLA1_BRAHO 60 kDa chaperonin (Fragment) OS=Brachyspira hyodysenteriae GN=cpn60 PE=4 SV=1

Observed	Mr(expt)	Mr(calc)	ppm	Start	End	Miss	Ions	Peptide
1811.9034	1810.8961	1810.8771	10.5	91 -	106	0	88	K.SLETSLSLVEGMQFDR.G

No match to: 1259.6888, 1276.6761, 1290.6913, 1737.8358

25. T1WF97 **Mass:** 20097 **Score:** 103 **Expect:** 2e-06 **Queries matched:** 1
T1WF97_9SPIR 60 kDa chaperonin (Fragment) OS=Brachyspira sp. KL-181 GN=cpn60 PE=4 SV=1

Observed	Mr(expt)	Mr(calc)	ppm	Start	End	Miss	Ions	Peptide
1811.9034	1810.8961	1810.8771	10.5	91 -	106	0	88	K.SLETSLSLVEGMQFDR.G

No match to: 1259.6888, 1276.6761, 1290.6913, 1737.8358

26. T1WF92 **Mass:** 20075 **Score:** 103 **Expect:** 2e-06 **Queries matched:** 1
T1WF92_9SPIR 60 kDa chaperonin (Fragment) OS=Brachyspira sp. CH-013 GN=cpn60 PE=4 SV=1

Observed	Mr(expt)	Mr(calc)	ppm	Start	End	Miss	Ions	Peptide
1811.9034	1810.8961	1810.8771	10.5	91 -	106	0	88	K.SLETSLSLVEGMQFDR.G

No match to: 1259.6888, 1276.6761, 1290.6913, 1737.8358

27. Q3YLA0 **Mass:** 20083 **Score:** 103 **Expect:** 2e-06 **Queries matched:** 1
Q3YLA0_9SPIR 60 kDa chaperonin (Fragment) OS=Brachyspira innocens GN=cpn60 PE=4 SV=1

Observed	Mr(expt)	Mr(calc)	ppm	Start	End	Miss	Ions	Peptide
1811.9034	1810.8961	1810.8771	10.5	91 -	106	0	88	K.SLETSLSLVEGMQFDR.G

No match to: 1259.6888, 1276.6761, 1290.6913, 1737.8358

28. T1WEF0 **Mass:** 20103 **Score:** 103 **Expect:** 2e-06 **Queries matched:** 1

T1WEF0_9SPIR 60 kDa chaperonin (Fragment) OS=Brachyspira sp. CH-014 GN=cpn60 PE=4 SV=1

Observed	Mr(expt)	Mr(calc)	ppm	Start		End	Miss	Ions	Peptide
1811.9034	1810.8961	1810.8771	10.5	91	-	106	0	88	K.SLETSLSLVEGMQFDR.G

No match to: 1259.6888, 1276.6761, 1290.6913, 1737.8358

29. Q3YL99 **Mass:** 20091 **Score:** 103 **Expect:** 2e-06 **Queries matched:** 1

Q3YL99_9SPIR 60 kDa chaperonin (Fragment) OS=Brachyspira alvinipulli GN=cpn60 PE=4 SV=1

Observed	Mr(expt)	Mr(calc)	ppm	Start		End	Miss	Ions	Peptide
1811.9034	1810.8961	1810.8771	10.5	91	-	106	0	88	K.SLETSLSLVEGMQFDR.G

No match to: 1259.6888, 1276.6761, 1290.6913, 1737.8358

30. T1WEW3 **Mass:** 20103 **Score:** 103 **Expect:** 2e-06 **Queries matched:** 1

T1WEW3_9SPIR 60 kDa chaperonin (Fragment) OS=Brachyspira sp. KL-207 GN=cpn60 PE=4 SV=1

Observed	Mr(expt)	Mr(calc)	ppm	Start		End	Miss	Ions	Peptide
1811.9034	1810.8961	1810.8771	10.5	91	-	106	0	88	K.SLETSLSLVEGMQFDR.G

No match to: 1259.6888, 1276.6761, 1290.6913, 1737.8358

31. T1WFA1 **Mass:** 20103 **Score:** 103 **Expect:** 2e-06 **Queries matched:** 1

T1WFA1_9SPIR 60 kDa chaperonin (Fragment) OS=Brachyspira sp. SHI-068 GN=cpn60 PE=4 SV=1

Observed	Mr(expt)	Mr(calc)	ppm	Start		End	Miss	Ions	Peptide
1811.9034	1810.8961	1810.8771	10.5	91	-	106	0	88	K.SLETSLSLVEGMQFDR.G

No match to: 1259.6888, 1276.6761, 1290.6913, 1737.8358

32. G1EI49 **Mass:** 20092 **Score:** 103 **Expect:** 2e-06 **Queries matched:** 1

G1EI49_9SPIR 60 kDa chaperonin (Fragment) OS=Brachyspira intermedia GN=cpn60 PE=4 SV=1

Observed	Mr(expt)	Mr(calc)	ppm	Start		End	Miss	Ions	Peptide
1811.9034	1810.8961	1810.8771	10.5	91	-	106	0	88	K.SLETSLSLVEGMQFDR.G

No match to: 1259.6888, 1276.6761, 1290.6913, 1737.8358

33. F8UU24 **Mass:** 20092 **Score:** 103 **Expect:** 2e-06 **Queries matched:** 1

F8UU24_9SPIR CPN60 (Fragment) OS=Brachyspira sp. j1330 GN=cpn60 PE=4 SV=1

Observed	Mr(expt)	Mr(calc)	ppm	Start		End	Miss	Ions	Peptide
1811.9034	1810.8961	1810.8771	10.5	91	-	106	0	88	K.SLETSLSLVEGMQFDR.G

No match to: 1259.6888, 1276.6761, 1290.6913, 1737.8358

34. T1WEG1 **Mass:** 20103 **Score:** 103 **Expect:** 2e-06 **Queries matched:** 1

T1WEG1_9SPIR 60 kDa chaperonin (Fragment) OS=Brachyspira sp. CH-108 GN=cpn60 PE=4 SV=1

Observed	Mr(expt)	Mr(calc)	ppm	Start		End	Miss	Ions	Peptide
1811.9034	1810.8961	1810.8771	10.5	91	-	106	0	88	K.SLETSLSLVEGMQFDR.G

No match to: 1259.6888, 1276.6761, 1290.6913, 1737.8358

35. T1WEV9 **Mass:** 20100 **Score:** 103 **Expect:** 2e-06 **Queries matched:** 1

T1WEV9_9SPIR 60 kDa chaperonin (Fragment) OS=Brachyspira sp. KL-049 GN=cpn60 PE=4 SV=1

Observed	Mr(expt)	Mr(calc)	ppm	Start		End	Miss	Ions	Peptide
1811.9034	1810.8961	1810.8771	10.5	91	-	106	0	88	K.SLETSLSLVEGMQFDR.G

No match to: 1259.6888, 1276.6761, 1290.6913, 1737.8358

36. T1WEW8 **Mass:** 20103 **Score:** 103 **Expect:** 2e-06 **Queries matched:** 1

T1WEW8_9SPIR 60 kDa chaperonin (Fragment) OS=Brachyspira sp. CH-110 GN=cpn60 PE=4 SV=1

Observed	Mr(expt)	Mr(calc)	ppm	Start		End	Miss	Ions	Peptide
1811.9034	1810.8961	1810.8771	10.5	91	-	106	0	88	K.SLETSLSLVEGMQFDR.G

No match to: 1259.6888, 1276.6761, 1290.6913, 1737.8358

37. T1WEF7 **Mass:** 20075 **Score:** 103 **Expect:** 2e-06 **Queries matched:** 1

T1WEF7_9SPIR 60 kDa chaperonin (Fragment) OS=Brachyspira sp. KL-194 GN=cpn60 PE=4 SV=1

Observed	Mr(expt)	Mr(calc)	ppm	Start		End	Miss	Ions	Peptide
1811.9034	1810.8961	1810.8771	10.5	91	-	106	0	88	K.SLETSLSLVEGMQFDR.G

No match to: 1259.6888, 1276.6761, 1290.6913, 1737.8358

38. R5LQ94 **Mass:** 58190 **Score:** 53 **Expect:** 0.19 **Queries matched:** 1

R5LQ94_9SPIR 60 kDa chaperonin OS=Brachyspira sp. CAG:700 GN=groL PE=3 SV=1

Observed	Mr(expt)	Mr(calc)	ppm	Start		End	Miss	Ions	Peptide
1276.6761	1275.6688	1275.6571	9.17	5	-	14	1	44	K.QLLFDEEARR.S

No match to: 1259.6888, 1290.6913, 1737.8358, 1811.9034

Search Parameters

**Type of search**	:	**Sequence Query**
**Enzyme**	:	**Trypsin**
**Fixed modifications**	:	**Carbamidomethyl (C)**
**Variable modifications**	:	**Oxidation (M)**
**Mass values**	:	**Monoisotopic**
**Protein Mass**	:	**Unrestricted**
**Peptide Mass Tolerance**	:	**± 50 ppm**
**Fragment Mass Tolerance**	:	**± 0.5 Da**
**Max Missed Cleavages**	:	**1**
**Instrument type**	:	**MALDI-TOF-TOF**
**Query1 (1259.6888,1+)**	:	**Locus:4.1.1.213487.57253**
**Query2 (1276.6761,1+)**	:	**Locus:4.1.1.213487.57251**
**Query3 (1290.6913,1+)**	:	**Locus:4.1.1.213487.57252**
**Query4 (1737.8358,1+)**	:	**Locus:4.1.1.213487.57254**
**Query5 (1811.9034,1+)**	:	**Locus:4.1.1.213487.57255**

Mascot: http://www.matrixscience.com/

Mascot Search Results

**User**	:
**Email**	:
**Search title**	: **F:\ROD_inmunoproteoma\MALDI\ppw_F6_modified.txt**
**MS data file**	: **F:\ROD_inmunoproteoma\MALDI\ppw_F6_modified.txt**
**Database**	: **Brachyspiraceae Brachyspiraceae_20160127-151122 (40573 sequences; 13470793 residues)**
**Timestamp**	: **29 Feb 2016 at 15:22:43 GMT**
**Warning**	: **A Peptide summary report will usually give a much clearer picture of MS/MS search results.**
**Top Score**	: **80 for A0A0H0V6M8, A0A0H0V6M8_BRAHO 60 kDa chaperonin OS=Brachyspira hyodysenteriae GN=groEL PE=3 SV=1**

Probability Based Mowse Score

Protein score is -10*Log(P), where P is the probability that the observed match is a random event.
Protein scores greater than 59 are significant (p<0.05).
Protein scores are derived from ions scores as a non-probabilistic basis for ranking protein hits.

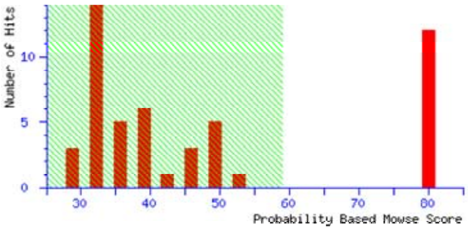

Protein Summary Report

Format As | Protein Summary (deprecated) | Help

Significance threshold p< 0.05 | Max. number of hits AUTO

Standard scoring ◉ MudPIT scoring ○ | Ions score or expect cut-off 0 | Show sub-sets 0

Show pop-ups ◉ Suppress pop-ups ○ | Sort unassigned Decreasing Score | Require bold red ☐

Re-Search All | Search Unmatched

Index

	Accession	Mass	Score	Description
1.	A0A0H0V6M8	58212	80	A0A0H0V6M8_BRAHO 60 kDa chaperonin OS=Brachyspira hyodysenteriae GN=groEL PE=3 SV=1
2.	A0A0H0TIS1	58228	80	A0A0H0TIS1_BRAHO 60 kDa chaperonin OS=Brachyspira hyodysenteriae GN=groEL PE=3 SV=1
3.	A0A0G4K5P2	58212	80	A0A0G4K5P2_9SPIR 60 kDa chaperonin OS=Brachyspira suanatina GN=groL PE=3 SV=1
4.	R5LQ94	58190	80	R5LQ94_9SPIR 60 kDa chaperonin OS=Brachyspira sp. CAG:700 GN=groL PE=3 SV=1
5.	C0QWM4	58212	80	C0QWM4_BRAHW 60 kDa chaperonin OS=Brachyspira hyodysenteriae (strain ATCC 49526 / WA1) GN=groL PE=3 SV=1
6.	G0EJ80	58197	80	G0EJ80_BRAIP 60 kDa chaperonin OS=Brachyspira intermedia (strain ATCC 51140 / PWS/A) GN=groL PE=3 SV=1
7.	D5U544	58148	80	D5U544_BRAM5 60 kDa chaperonin OS=Brachyspira murdochii (strain ATCC 51284 / DSM 12563 / 56-150) GN=groL PE=3
8.	L8XV88	58195	80	L8XV88_9SPIR 60 kDa chaperonin OS=Brachyspira hampsonii 30599 GN=groL PE=3 SV=1
9.	J9USS2	58110	78	J9USS2_BRAPL 60 kDa chaperonin OS=Brachyspira pilosicoli B2904 GN=groL PE=3 SV=1
10.	D8IB78	58110	78	D8IB78_BRAP9 60 kDa chaperonin OS=Brachyspira pilosicoli (strain ATCC BAA-1826 / 95/1000) GN=groL PE=3 SV=1
11.	K0JLL7	58110	78	K0JLL7_BRAPL 60 kDa chaperonin OS=Brachyspira pilosicoli WesB GN=groL PE=3 SV=1
12.	L0X051	58181	78	L0X051_9SPIR 60 kDa chaperonin OS=Brachyspira hampsonii 30446 GN=groL PE=3 SV=1
13.	A0A0H0U3M7	22322	52	A0A0H0U3M7_BRAHO Uncharacterized protein OS=Brachyspira hyodysenteriae GN=SU43_12350 PE=4 SV=1

Results List

1. A0A0H0V6M8 **Mass:** 58212 **Score:** 80 **Expect:** 0.00041 **Queries matched:** 2
A0A0H0V6M8_BRAHO 60 kDa chaperonin OS=Brachyspira hyodysenteriae GN=groEL PE=3 SV=1

Observed	Mr(expt)	Mr(calc)	ppm	Start	End	Miss	Ions	Peptide
1276.6908	1275.6835	1275.6571	20.7	5 -	14	1	27	K.QLLFDEEARR.A
1542.8787	1541.8714	1541.8454	16.9	44 -	58	0	30	K.FGPPTIINDGVTIAK.E

No match to: 1259.6700, 1290.7073, 1304.7234

2. A0A0H0TIS1 **Mass:** 58228 **Score:** 80 **Expect:** 0.00041 **Queries matched:** 2
A0A0H0TIS1_BRAHO 60 kDa chaperonin OS=Brachyspira hyodysenteriae GN=groEL PE=3 SV=1

Observed	Mr(expt)	Mr(calc)	ppm	Start	End	Miss	Ions	Peptide
1276.6908	1275.6835	1275.6571	20.7	5 -	14	1	27	K.QLLFDEEARR.A
1542.8787	1541.8714	1541.8454	16.9	44 -	58	0	30	K.FGPPTIINDGVTIAK.E

No match to: 1259.6700, 1290.7073, 1304.7234

3. A0A0G4K5P2 **Mass:** 58212 **Score:** 80 **Expect:** 0.00041 **Queries matched:** 2
A0A0G4K5P2_9SPIR 60 kDa chaperonin OS=Brachyspira suanatina GN=groL PE=3 SV=1

Observed	Mr(expt)	Mr(calc)	ppm	Start	End	Miss	Ions	Peptide
1276.6908	1275.6835	1275.6571	20.7	5 -	14	1	27	K.QLLFDEEARR.A
1542.8787	1541.8714	1541.8454	16.9	44 -	58	0	30	K.FGPPTIINDGVTIAK.E

No match to: 1259.6700, 1290.7073, 1304.7234

4. R5LQ94 **Mass:** 58190 **Score:** 80 **Expect:** 0.00041 **Queries matched:** 2
R5LQ94_9SPIR 60 kDa chaperonin OS=Brachyspira sp. CAG:700 GN=groL PE=3 SV=1

Observed	Mr(expt)	Mr(calc)	ppm	Start	End	Miss	Ions	Peptide
1276.6908	1275.6835	1275.6571	20.7	5 -	14	1	27	K.QLLFDEEARR.S
1542.8787	1541.8714	1541.8454	16.9	44 -	58	0	30	K.FGPPTIINDGVTIAK.E

No match to: 1259.6700, 1290.7073, 1304.7234

5. C0QWM4 **Mass:** 58212 **Score:** 80 **Expect:** 0.00041 **Queries matched:** 2
C0QWM4_BRAHW 60 kDa chaperonin OS=Brachyspira hyodysenteriae (strain ATCC 49526 / WA1) GN=groL PE=3 SV=1

Observed	Mr(expt)	Mr(calc)	ppm	Start	End	Miss	Ions	Peptide
1276.6908	1275.6835	1275.6571	20.7	5 -	14	1	27	K.QLLFDEEARR.A
1542.8787	1541.8714	1541.8454	16.9	44 -	58	0	30	K.FGPPTIINDGVTIAK.E

No match to: 1259.6700, 1290.7073, 1304.7234

6. G0EJ80 **Mass:** 58197 **Score:** 80 **Expect:** 0.00041 **Queries matched:** 2
G0EJ80_BRAIP 60 kDa chaperonin OS=Brachyspira intermedia (strain ATCC 51140 / PWS/A) GN=groL PE=3 SV=1

Observed	Mr(expt)	Mr(calc)	ppm	Start	End	Miss	Ions	Peptide
1276.6908	1275.6835	1275.6571	20.7	5 -	14	1	27	K.QLLFDEEARR.A
1542.8787	1541.8714	1541.8454	16.9	44 -	58	0	30	K.FGPPTIINDGVTIAK.E

No match to: 1259.6700, 1290.7073, 1304.7234

7. D5U544 **Mass:** 58148 **Score:** 80 **Expect:** 0.00041 **Queries matched:** 2
D5U544_BRAM5 60 kDa chaperonin OS=Brachyspira murdochii (strain ATCC 51284 / DSM 12563 / 56-150) GN=groL PE=3 SV=1

Observed	Mr(expt)	Mr(calc)	ppm	Start	End	Miss	Ions	Peptide
1276.6908	1275.6835	1275.6571	20.7	5 -	14	1	27	K.QLLFDEEARR.A
1542.8787	1541.8714	1541.8454	16.9	44 -	58	0	30	K.FGPPTIINDGVTIAK.E

No match to: 1259.6700, 1290.7073, 1304.7234

8. L8XV88 **Mass:** 58195 **Score:** 80 **Expect:** 0.00041 **Queries matched:** 2
L8XV88_9SPIR 60 kDa chaperonin OS=Brachyspira hampsonii 30599 GN=groL PE=3 SV=1

Observed	Mr(expt)	Mr(calc)	ppm	Start	End	Miss	Ions	Peptide
1276.6908	1275.6835	1275.6571	20.7	5 -	14	1	27	K.QLLFDEEARR.A
1542.8787	1541.8714	1541.8454	16.9	44 -	58	0	30	K.FGPPTIINDGVTIAK.E

No match to: 1259.6700, 1290.7073, 1304.7234

9. J9USS2 **Mass:** 58110 **Score:** 78 **Expect:** 0.0006 **Queries matched:** 2
J9USS2_BRAPL 60 kDa chaperonin OS=Brachyspira pilosicoli B2904 GN=groL PE=3 SV=1

Observed	Mr(expt)	Mr(calc)	ppm	Start	End	Miss	Ions	Peptide
1276.6908	1275.6835	1275.6571	20.7	5 -	14	1	27	K.QLLFDEEARR.S
1542.8787	1541.8714	1541.8454	16.9	44 -	58	0	30	K.FGPPTIINDGVTIAK.E

No match to: 1259.6700, 1290.7073, 1304.7234

10. D8IB78 **Mass:** 58110 **Score:** 78 **Expect:** 0.0006 **Queries matched:** 2
D8IB78_BRAP9 60 kDa chaperonin OS=Brachyspira pilosicoli (strain ATCC BAA-1826 / 95/1000) GN=groL PE=3 SV=1

Observed	Mr(expt)	Mr(calc)	ppm	Start	End	Miss	Ions	Peptide
1276.6908	1275.6835	1275.6571	20.7	5 -	14	1	27	K.QLLFDEEARR.S
1542.8787	1541.8714	1541.8454	16.9	44 -	58	0	30	K.FGPPTIINDGVTIAK.E

No match to: 1259.6700, 1290.7073, 1304.7234

11. K0JLL7 **Mass:** 58110 **Score:** 78 **Expect:** 0.0006 **Queries matched:** 2
K0JLL7_BRAPL 60 kDa chaperonin OS=Brachyspira pilosicoli WesB GN=groL PE=3 SV=1

Observed	Mr(expt)	Mr(calc)	ppm	Start	End	Miss	Ions	Peptide
1276.6908	1275.6835	1275.6571	20.7	5 -	14	1	27	K.QLLFDEEARR.S
1542.8787	1541.8714	1541.8454	16.9	44 -	58	0	30	K.FGPPTIINDGVTIAK.E

No match to: 1259.6700, 1290.7073, 1304.7234

12. L0X051 **Mass:** 58181 **Score:** 78 **Expect:** 0.0006 **Queries matched:** 2
L0X051_9SPIR 60 kDa chaperonin OS=Brachyspira hampsonii 30446 GN=groL PE=3 SV=1

Observed	Mr(expt)	Mr(calc)	ppm	Start	End	Miss	Ions	Peptide
1276.6908	1275.6835	1275.6571	20.7	5 -	14	1	27	K.QLLFDEEARR.A
1542.8787	1541.8714	1541.8454	16.9	44 -	58	0	30	K.FGPPTIINDGVTIAK.E

No match to: 1259.6700, 1290.7073, 1304.7234

13. A0A0H0U3M7 **Mass:** 22322 **Score:** 52 **Expect:** 0.26 **Queries matched:** 2
A0A0H0U3M7_BRAHO Uncharacterized protein OS=Brachyspira hyodysenteriae GN=SU43_12350 PE=4 SV=1

Observed	Mr(expt)	Mr(calc)	ppm	Start	End	Miss	Ions	Peptide
1259.6700	1258.6627	1258.6961	-26.54	7 -	16	1	---	K.YKFGLLDYIK.D
1290.7073	1289.7000	1289.6901	7.72	141 -	151	0	21	K.LQAMLETLDIK.E + Oxidation (M)

No match to: 1276.6908, 1304.7234, 1542.8787

Search Parameters

Type of search : Sequence Query
Enzyme : Trypsin
Fixed modifications : Carbamidomethyl (C)
Variable modifications : Oxidation (M)
Mass values : Monoisotopic
Protein Mass : Unrestricted
Peptide Mass Tolerance : ± 50 ppm
Fragment Mass Tolerance: ± 0.5 Da
Max Missed Cleavages : 1
Instrument type : MALDI-TOF-TOF
Query1 (1259.6700,1+) : Locus:1.1.1.210701.56307
Query2 (1276.6908,1+) : Locus:1.1.1.210701.56385
Query3 (1290.7073,1+) : Locus:1.1.1.210701.56306
Query4 (1304.7234,1+) : Locus:1.1.1.210701.56308
Query5 (1542.8787,1+) : Locus:1.1.1.210701.56309

Mascot: http://www.matrixscience.com/

Mascot Search Results

```
User          :
Email         :
Search title  : F:\ROD_inmunoproteoma\MALDI\ppw_2016_562_563_ROD\ppw_G16_145397048203.txt
MS data file  : F:\ROD_inmunoproteoma\MALDI\ppw_2016_562_563_ROD\ppw_G16_145397048203.txt
Database      : Brachyspiraceae Brachyspiraceae_20160127-151122 (40573 sequences; 13470793 residues)
Timestamp     : 23 Feb 2016 at 16:05:10 GMT
Warning       : A Peptide summary report will usually give a much clearer picture of MS/MS search results.
Top Score     : 390 for J9UGL5, J9UGL5_BRAPL Biotin/lipoyl attachment domain-containing protein OS=Brachyspira pilosicoli B2904 GN=B2904_o
```

Probability Based Mowse Score

Protein score is -10*Log(P), where P is the probability that the observed match is a random event.
Protein scores greater than 59 are significant (p<0.05).
Protein scores are derived from ions scores as a non-probabilistic basis for ranking protein hits.

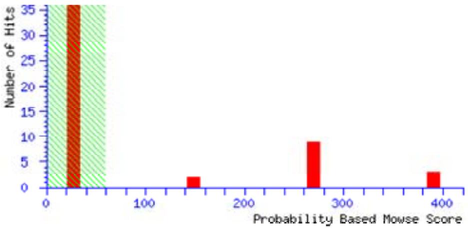

Protein Summary Report

Format As | Protein Summary (deprecated) | Help

Significance threshold p< 0.05 | Max. number of hits AUTO

Standard scoring ◉ MudPIT scoring ○ | Ions score or expect cut-off 0 | Show sub-sets 0

Show pop-ups ◉ Suppress pop-ups ○ | Sort unassigned Decreasing Score | Require bold red ☐

Re-Search All | Search Unmatched

Index

	Accession	Mass	Score	Description
1.	J9UGL5	64816	390	J9UGL5_BRAPL Biotin/lipoyl attachment domain-containing protein OS=Brachyspira pilosicoli B2904 GN=B2904_orf13
2.	D8I9T6	64972	390	D8I9T6_BRAP9 Biotin/lipoyl attachment domain-containing protein OS=Brachyspira pilosicoli (strain ATCC BAA-182
3.	K0JKW5	64928	390	K0JKW5_BRAPL Biotin/lipoyl attachment domain-containing protein OS=Brachyspira pilosicoli WesB GN=WESB_1472 PE
4.	A0A0H0V274	64689	272	A0A0H0V274_BRAHO Biotin attachment protein OS=Brachyspira hyodysenteriae GN=SZ42_00235 PE=4 SV=1
5.	A0A0H0XA98	64689	272	A0A0H0XA98_BRAHO Biotin attachment protein OS=Brachyspira hyodysenteriae GN=SU46_04095 PE=4 SV=1
6.	A0A0H0TY53	64689	272	A0A0H0TY53_BRAHO Biotin attachment protein OS=Brachyspira hyodysenteriae GN=SR30_05280 PE=4 SV=1
7.	C0QY07	64689	272	C0QY07_BRAHW Biotin/lipoyl attachment domain-containing protein OS=Brachyspira hyodysenteriae (strain ATCC 495
8.	A0A0H0V9F5	64703	272	A0A0H0V9F5_BRAHO Biotin attachment protein OS=Brachyspira hyodysenteriae GN=SZ48_06550 PE=4 SV=1
9.	G0EPB7	64659	272	G0EPB7_BRAIP Biotin/lipoyl attachment domain-containing protein OS=Brachyspira intermedia (strain ATCC 51140 /
10.	L0X024	64721	272	L0X024_9SPIR Pyruvate carboxylase OS=Brachyspira hampsonii 30446 GN=A966_11217 PE=4 SV=1
11.	L8XSV2	64877	272	L8XSV2_9SPIR Pyruvate carboxylase OS=Brachyspira hampsonii 30599 GN=H263_10407 PE=4 SV=1
12.	D5U4C9	64969	272	D5U4C9_BRAM5 Pyruvate carboxylase OS=Brachyspira murdochii (strain ATCC 51284 / DSM 12563 / 56-150) GN=Bmur_00
13.	R5LE51	65062	154	R5LE51_9SPIR Biotin/lipoyl attachment domain-containing protein OS=Brachyspira sp. CAG:700 GN=BN758_02112 PE=4
14.	A0A0G4K6M6	64677	154	A0A0G4K6M6_9SPIR Biotin attachment protein OS=Brachyspira suanatina GN=BRSU_1275 PE=4 SV=1
15.	J9UUY5	18470	36	J9UUY5_BRAPL 3-deoxy-D-manno-octulosonate 8-phosphate phosphatase OS=Brachyspira pilosicoli B2904 GN=B2904_orf

Results List

1. J9UGL5 **Mass:** 64816 **Score:** 390 **Expect:** 4.1e-35 **Queries matched:** 4
J9UGL5_BRAPL Biotin/lipoyl attachment domain-containing protein OS=Brachyspira pilosicoli B2904 GN=B2904_orf1329 PE=4 SV=1

Observed	Mr(expt)	Mr(calc)	ppm	Start	End	Miss	Ions	Peptide
1254.6470	1253.6397	1253.6728	-26.39	87 -	98	0	60	R.GVNVVGLESQPR.D
1383.7205	1382.7132	1382.7518	-27.89	74 -	86	0	102	K.TVGPDVNLQTLAR.G
1574.7877	1573.7804	1573.8286	-30.63	320 -	332	1	73	R.DINVMNRFPEVIK.A
2655.2666	2654.2593	2654.3221	-23.66	396 -	419	1	97	K.IASEQLGLQPTTELAMDIDDRNPK.K

No match to: 2691.2236

2. D8I9T6 **Mass:** 64972 **Score:** 390 **Expect:** 4.1e-35 **Queries matched:** 4
D8I9T6_BRAP9 Biotin/lipoyl attachment domain-containing protein OS=Brachyspira pilosicoli (strain ATCC BAA-1826 / 95/1000) GN=BP951000

Observed	Mr(expt)	Mr(calc)	ppm	Start	End	Miss	Ions	Peptide
1254.6470	1253.6397	1253.6728	-26.39	87 -	98	0	60	R.GVNVVGLESQPR.D
1383.7205	1382.7132	1382.7518	-27.89	74 -	86	0	102	K.TVGPDVNLQTLAR.G
1574.7877	1573.7804	1573.8286	-30.63	320 -	332	1	73	R.DINVMNRFPEVIK.A
2655.2666	2654.2593	2654.3221	-23.66	396 -	419	1	97	K.IASEQLGLQPTTELAMDIDDRNPK.K

No match to: 2691.2236

3. K0JKW5 **Mass:** 64928 **Score:** 390 **Expect:** 4.1e-35 **Queries matched:** 4
K0JKW5_BRAPL Biotin/lipoyl attachment domain-containing protein OS=Brachyspira pilosicoli WesB GN=WESB_1472 PE=4 SV=1

Observed	Mr(expt)	Mr(calc)	ppm	Start	End	Miss	Ions	Peptide
1254.6470	1253.6397	1253.6728	-26.39	87 -	98	0	60	R.GVNVVGLESQPR.D
1383.7205	1382.7132	1382.7518	-27.89	74 -	86	0	102	K.TVGPDVNLQTLAR.G
1574.7877	1573.7804	1573.8286	-30.63	320 -	332	1	73	R.DINVMNRFPEVIK.A
2655.2666	2654.2593	2654.3221	-23.66	396 -	419	1	97	K.IASEQLGLQPTTELAMDIDDRNPK.K

No match to: 2691.2236

4. A0A0H0V274 **Mass:** 64689 **Score:** 272 **Expect:** 2.6e-23 **Queries matched:** 3
A0A0H0V274_BRAHO Biotin attachment protein OS=Brachyspira hyodysenteriae GN=SZ42_00235 PE=4 SV=1

Observed	Mr(expt)	Mr(calc)	ppm	Start		End	Miss	Ions	Peptide
1254.6470	1253.6397	1253.6728	-26.39	87	-	98	0	60	R.GVNVVGLESQPR.E
1383.7205	1382.7132	1382.7518	-27.89	74	-	86	0	102	K.TVGPDVNLQTLAR.G
1574.7877	1573.7804	1573.8286	-30.63	320	-	332	1	73	R.DINVMNRFPEVIK.A

No match to: 2655.2666, 2691.2236

5. A0A0H0XA98 **Mass:** 64689 **Score:** 272 **Expect:** 2.6e-23 **Queries matched:** 3
A0A0H0XA98_BRAHO Biotin attachment protein OS=Brachyspira hyodysenteriae GN=SU46_04095 PE=4 SV=1

Observed	Mr(expt)	Mr(calc)	ppm	Start		End	Miss	Ions	Peptide
1254.6470	1253.6397	1253.6728	-26.39	87	-	98	0	60	R.GVNVVGLESQPR.E
1383.7205	1382.7132	1382.7518	-27.89	74	-	86	0	102	K.TVGPDVNLQTLAR.G
1574.7877	1573.7804	1573.8286	-30.63	320	-	332	1	73	R.DINVMNRFPEVIK.A

No match to: 2655.2666, 2691.2236

6. A0A0H0TY53 **Mass:** 64689 **Score:** 272 **Expect:** 2.6e-23 **Queries matched:** 3
A0A0H0TY53_BRAHO Biotin attachment protein OS=Brachyspira hyodysenteriae GN=SR30_05280 PE=4 SV=1

Observed	Mr(expt)	Mr(calc)	ppm	Start		End	Miss	Ions	Peptide
1254.6470	1253.6397	1253.6728	-26.39	87	-	98	0	60	R.GVNVVGLESQPR.E
1383.7205	1382.7132	1382.7518	-27.89	74	-	86	0	102	K.TVGPDVNLQTLAR.G
1574.7877	1573.7804	1573.8286	-30.63	320	-	332	1	73	R.DINVMNRFPEVIK.A

No match to: 2655.2666, 2691.2236

7. C0QY07 **Mass:** 64689 **Score:** 272 **Expect:** 2.6e-23 **Queries matched:** 3
C0QY07_BRAHW Biotin/lipoyl attachment domain-containing protein OS=Brachyspira hyodysenteriae (strain ATCC 49526 / WA1) GN=BHWA1_0038(

Observed	Mr(expt)	Mr(calc)	ppm	Start		End	Miss	Ions	Peptide
1254.6470	1253.6397	1253.6728	-26.39	87	-	98	0	60	R.GVNVVGLESQPR.E
1383.7205	1382.7132	1382.7518	-27.89	74	-	86	0	102	K.TVGPDVNLQTLAR.G
1574.7877	1573.7804	1573.8286	-30.63	320	-	332	1	73	R.DINVMNRFPEVIK.A

No match to: 2655.2666, 2691.2236

8. A0A0H0V9F5 **Mass:** 64703 **Score:** 272 **Expect:** 2.6e-23 **Queries matched:** 3
A0A0H0V9F5_BRAHO Biotin attachment protein OS=Brachyspira hyodysenteriae GN=SZ48_06550 PE=4 SV=1

Observed	Mr(expt)	Mr(calc)	ppm	Start		End	Miss	Ions	Peptide
1254.6470	1253.6397	1253.6728	-26.39	87	-	98	0	60	R.GVNVVGLESQPR.E
1383.7205	1382.7132	1382.7518	-27.89	74	-	86	0	102	K.TVGPDVNLQTLAR.G
1574.7877	1573.7804	1573.8286	-30.63	320	-	332	1	73	R.DINVMNRFPEVIK.A

No match to: 2655.2666, 2691.2236

9. G0EPB7 **Mass:** 64659 **Score:** 272 **Expect:** 2.6e-23 **Queries matched:** 3
G0EPB7_BRAIP Biotin/lipoyl attachment domain-containing protein OS=Brachyspira intermedia (strain ATCC 51140 / PWS/A) GN=Bint_1368 PE=

Observed	Mr(expt)	Mr(calc)	ppm	Start		End	Miss	Ions	Peptide
1254.6470	1253.6397	1253.6728	-26.39	87	-	98	0	60	R.GVNVVGLESQPR.E
1383.7205	1382.7132	1382.7518	-27.89	74	-	86	0	102	K.TVGPDVNLQTLAR.G
1574.7877	1573.7804	1573.8286	-30.63	320	-	332	1	73	R.DINVMNRFPEVIK.A

No match to: 2655.2666, 2691.2236

10. L0X024 **Mass:** 64721 **Score:** 272 **Expect:** 2.6e-23 **Queries matched:** 3
L0X024_9SPIR Pyruvate carboxylase OS=Brachyspira hampsonii 30446 GN=A966_11217 PE=4 SV=1

Observed	Mr(expt)	Mr(calc)	ppm	Start		End	Miss	Ions	Peptide
1254.6470	1253.6397	1253.6728	-26.39	87	-	98	0	60	R.GVNVVGLESQPR.E
1383.7205	1382.7132	1382.7518	-27.89	74	-	86	0	102	K.TVGPDVNLQTLAR.G
1574.7877	1573.7804	1573.8286	-30.63	320	-	332	1	73	R.DINVMNRFPEVIK.A

No match to: 2655.2666, 2691.2236

11. L8XSV2 **Mass:** 64877 **Score:** 272 **Expect:** 2.6e-23 **Queries matched:** 3
L8XSV2_9SPIR Pyruvate carboxylase OS=Brachyspira hampsonii 30599 GN=H263_10407 PE=4 SV=1

Observed	Mr(expt)	Mr(calc)	ppm	Start		End	Miss	Ions	Peptide
1254.6470	1253.6397	1253.6728	-26.39	87	-	98	0	60	R.GVNVVGLESQPR.E
1383.7205	1382.7132	1382.7518	-27.89	74	-	86	0	102	K.TVGPDVNLQTLAR.G
1574.7877	1573.7804	1573.8286	-30.63	320	-	332	1	73	R.DINVMNRFPEVIK.A

No match to: 2655.2666, 2691.2236

12. D5U4C9 **Mass:** 64969 **Score:** 272 **Expect:** 2.6e-23 **Queries matched:** 3
D5U4C9_BRAM5 Pyruvate carboxylase OS=Brachyspira murdochii (strain ATCC 51284 / DSM 12563 / 56-150) GN=Bmur_0063 PE=4 SV=1

Observed	Mr(expt)	Mr(calc)	ppm	Start		End	Miss	Ions	Peptide
1254.6470	1253.6397	1253.6728	-26.39	87	-	98	0	60	R.GVNVVGLESQPR.E
1383.7205	1382.7132	1382.7518	-27.89	74	-	86	0	102	K.TVGPDVNLQTLAR.G
1574.7877	1573.7804	1573.8286	-30.63	320	-	332	1	73	R.DINVMNRFPEVIK.A

No match to: 2655.2666, 2691.2236

13. R5LE51 **Mass:** 65062 **Score:** 154 **Expect:** 1.6e-11 **Queries matched:** 2
R5LE51_9SPIR Biotin/lipoyl attachment domain-containing protein OS=Brachyspira sp. CAG:700 GN=BN758_02112 PE=4 SV=1

Observed	Mr(expt)	Mr(calc)	ppm	Start		End	Miss	Ions	Peptide
1254.6470	1253.6397	1253.6728	-26.39	87	-	98	0	60	R.GVNVVGLESQPR.E
1574.7877	1573.7804	1573.8286	-30.63	320	-	332	1	73	R.DINVMNRFPEVIK.A

No match to: 1383.7205, 2655.2666, 2691.2236

14. A0A0G4K6M6 **Mass:** 64677 **Score:** 154 **Expect:** 1.6e-11 **Queries matched:** 2
A0A0G4K6M6_9SPIR Biotin attachment protein OS=Brachyspira suanatina GN=BRSU_1275 PE=4 SV=1

Observed	Mr(expt)	Mr(calc)	ppm	Start		End	Miss	Ions	Peptide
1254.6470	1253.6397	1253.6728	-26.39	87	-	98	0	60	R.GVNVVGLESQPR.E
1574.7877	1573.7804	1573.8286	-30.63	320	-	332	1	73	R.DINVMNRFPEVIK.A

No match to: 1383.7205, 2655.2666, 2691.2236

15. J9UUY5 **Mass:** 18470 **Score:** 36 **Expect:** 11 **Queries matched:** 2
J9UUY5_BRAPL 3-deoxy-D-manno-octulosonate 8-phosphate phosphatase OS=Brachyspira pilosicoli B2904 GN=B2904_orf1436 PE=4 SV=1

Observed	Mr(expt)	Mr(calc)	ppm	Start	End	Miss	Ions	Peptide
1574.7877	1573.7804	1573.7157	41.1	1 -	14	0	---	-.MFVFDIDGVMTDGK.L
2691.2236	2690.2163	2690.2786	-23.15	2 -	25	1	---	M.FVFDIDGVMTDGKLIFDDNGVETK.F + Oxidation (M)

No match to: 1254.6470, 1383.7205, 2655.2666

Search Parameters

```
Type of search         : Sequence Query
Enzyme                 : Trypsin
Fixed modifications    : Carbamidomethyl (C)
Variable modifications : Oxidation (M)
Mass values            : Monoisotopic
Protein Mass           : Unrestricted
Peptide Mass Tolerance : ± 50 ppm
Fragment Mass Tolerance: ± 0.5 Da
Max Missed Cleavages   : 1
Instrument type        : MALDI-TOF-TOF
Query1 (1254.6470,1+)  : Locus:4.1.1.211132.56534
Query2 (1383.7205,1+)  : Locus:4.1.1.211132.56530
Query3 (1574.7877,1+)  : Locus:4.1.1.211132.56531
Query4 (2655.2666,1+)  : Locus:4.1.1.211132.56533
Query5 (2691.2236,1+)  : Locus:4.1.1.211132.56532
```

Mascot: http://www.matrixscience.com/

Mascot Search Results

```
User          :
Email         :
Search title  : F:\ROD_inmunoproteoma\MALDI\ppw_2016_562_563_ROD\ppw_H10_145397049014.txt
MS data file  : F:\ROD_inmunoproteoma\MALDI\ppw_2016_562_563_ROD\ppw_H10_145397049014.txt
Database      : Brachyspiraceae Brachyspiraceae_20160127-151122 (40573 sequences; 13470793 residues)
Timestamp     : 23 Feb 2016 at 16:05:38 GMT
Warning       : A Peptide summary report will usually give a much clearer picture of MS/MS search results.
Top Score     : 293 for J9USS2, J9USS2_BRAPL 60 kDa chaperonin OS=Brachyspira pilosicoli B2904 GN=groL PE=3 SV=1
```

Probability Based Mowse Score

Protein score is -10*Log(P), where P is the probability that the observed match is a random event.
Protein scores greater than 59 are significant (p<0.05).
Protein scores are derived from ions scores as a non-probabilistic basis for ranking protein hits.

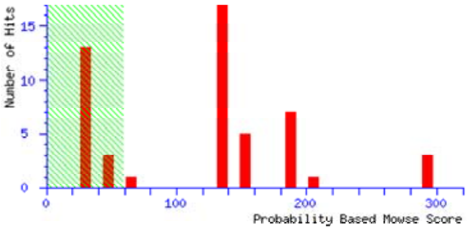

Protein Summary Report

Format As | Protein Summary (deprecated) | Help

Significance threshold p< 0.05 | Max. number of hits AUTO

Standard scoring ◉ MudPIT scoring ○ | Ions score or expect cut-off 0 | Show sub-sets 0

Show pop-ups ◉ Suppress pop-ups ○ | Sort unassigned Decreasing Score | Require bold red ☐

Re-Search All | Search Unmatched

Index

	Accession	Mass	Score	Description
1.	J9USS2	58110	293	J9USS2_BRAPL 60 kDa chaperonin OS=Brachyspira pilosicoli B2904 GN=groL PE=3 SV=1
2.	D8IB78	58110	293	D8IB78_BRAP9 60 kDa chaperonin OS=Brachyspira pilosicoli (strain ATCC BAA-1826 / 95/1000) GN=groL PE=3 SV=1
3.	K0JLL7	58110	293	K0JLL7_BRAPL 60 kDa chaperonin OS=Brachyspira pilosicoli WesB GN=groL PE=3 SV=1
4.	L0X051	58181	205	L0X051_9SPIR 60 kDa chaperonin OS=Brachyspira hampsonii 30446 GN=groL PE=3 SV=1
5.	A0A0H0V6M8	58212	189	A0A0H0V6M8_BRAHO 60 kDa chaperonin OS=Brachyspira hyodysenteriae GN=groEL PE=3 SV=1
6.	A0A0H0TIS1	58228	189	A0A0H0TIS1_BRAHO 60 kDa chaperonin OS=Brachyspira hyodysenteriae GN=groEL PE=3 SV=1
7.	A0A0G4K5P2	58212	189	A0A0G4K5P2_9SPIR 60 kDa chaperonin OS=Brachyspira suanatina GN=groL PE=3 SV=1
8.	C0QWM4	58212	189	C0QWM4_BRAHW 60 kDa chaperonin OS=Brachyspira hyodysenteriae (strain ATCC 49526 / WA1) GN=groL PE=3 SV=1
9.	G0EJ80	58197	189	G0EJ80_BRAIP 60 kDa chaperonin OS=Brachyspira intermedia (strain ATCC 51140 / PWS/A) GN=groL PE=3 SV=1
10.	D5U544	58148	189	D5U544_BRAM5 60 kDa chaperonin OS=Brachyspira murdochii (strain ATCC 51284 / DSM 12563 / 56-150) GN=groL PE=3
11.	L8XV88	58195	189	L8XV88_9SPIR 60 kDa chaperonin OS=Brachyspira hampsonii 30599 GN=groL PE=3 SV=1
12.	T1WGB0	20074	146	T1WGB0_9SPIR 60 kDa chaperonin (Fragment) OS=Brachyspira sp. CH-003 GN=cpn60 PE=4 SV=1
13.	T1WFK2	20144	146	T1WFK2_9SPIR 60 kDa chaperonin (Fragment) OS=Brachyspira sp. SHI-129 GN=cpn60 PE=4 SV=1
14.	T1WGB4	20074	146	T1WGB4_9SPIR 60 kDa chaperonin (Fragment) OS=Brachyspira hampsonii bv. II GN=cpn60 PE=4 SV=1
15.	T1WFJ6	20144	146	T1WFJ6_9SPIR 60 kDa chaperonin (Fragment) OS=Brachyspira sp. SHI-031 GN=cpn60 PE=4 SV=1
16.	Q3YLA3	19965	146	Q3YLA3_BRAPL 60 kDa chaperonin (Fragment) OS=Brachyspira pilosicoli GN=cpn60 PE=4 SV=1
17.	T1WGB9	20103	128	T1WGB9_9SPIR 60 kDa chaperonin (Fragment) OS=Brachyspira sp. SHI-067 GN=cpn60 PE=4 SV=1
18.	Q3YL98	20103	128	Q3YL98_9SPIR 60 kDa chaperonin (Fragment) OS=Brachyspira murdochii GN=cpn60 PE=4 SV=1
19.	T1WFJ0	20174	128	T1WFJ0_9SPIR 60 kDa chaperonin (Fragment) OS=Brachyspira sp. KL-169 GN=cpn60 PE=4 SV=1
20.	Q3YLA1	20036	128	Q3YLA1_BRAHO 60 kDa chaperonin (Fragment) OS=Brachyspira hyodysenteriae GN=cpn60 PE=4 SV=1
21.	T1WF97	20097	128	T1WF97_9SPIR 60 kDa chaperonin (Fragment) OS=Brachyspira sp. KL-181 GN=cpn60 PE=4 SV=1
22.	T1WF92	20075	128	T1WF92_9SPIR 60 kDa chaperonin (Fragment) OS=Brachyspira sp. CH-013 GN=cpn60 PE=4 SV=1
23.	Q3YLA0	20083	128	Q3YLA0_9SPIR 60 kDa chaperonin (Fragment) OS=Brachyspira innocens GN=cpn60 PE=4 SV=1
24.	T1WEF0	20103	128	T1WEF0_9SPIR 60 kDa chaperonin (Fragment) OS=Brachyspira sp. CH-014 GN=cpn60 PE=4 SV=1
25.	Q3YL99	20091	128	Q3YL99_9SPIR 60 kDa chaperonin (Fragment) OS=Brachyspira alvinipulli GN=cpn60 PE=4 SV=1
26.	T1WEW3	20103	128	T1WEW3_9SPIR 60 kDa chaperonin (Fragment) OS=Brachyspira sp. KL-207 GN=cpn60 PE=4 SV=1
27.	T1WFA1	20103	128	T1WFA1_9SPIR 60 kDa chaperonin (Fragment) OS=Brachyspira sp. SHI-068 GN=cpn60 PE=4 SV=1
28.	G1EI49	20092	128	G1EI49_9SPIR 60 kDa chaperonin (Fragment) OS=Brachyspira intermedia GN=cpn60 PE=4 SV=1
29.	F8UU24	20092	128	F8UU24_9SPIR CPN60 (Fragment) OS=Brachyspira sp. j1330 GN=cpn60 PE=4 SV=1
30.	T1WEG1	20103	128	T1WEG1_9SPIR 60 kDa chaperonin (Fragment) OS=Brachyspira sp. CH-108 GN=cpn60 PE=4 SV=1
31.	T1WEV9	20100	128	T1WEV9_9SPIR 60 kDa chaperonin (Fragment) OS=Brachyspira sp. KL-049 GN=cpn60 PE=4 SV=1
32.	T1WEW8	20103	128	T1WEW8_9SPIR 60 kDa chaperonin (Fragment) OS=Brachyspira sp. CH-110 GN=cpn60 PE=4 SV=1
33.	T1WEF7	20075	128	T1WEF7_9SPIR 60 kDa chaperonin (Fragment) OS=Brachyspira sp. KL-194 GN=cpn60 PE=4 SV=1
34.	R5LQ94	58190	62	R5LQ94_9SPIR 60 kDa chaperonin OS=Brachyspira sp. CAG:700 GN=groL PE=3 SV=1
35.	A0A0H0UCV1	38722	41	A0A0H0UCV1_BRAHO Molecular chaperone GroES OS=Brachyspira hyodysenteriae GN=SZ47_11215 PE=3 SV=1

Results List

1. J9USS2 **Mass:** 58110 **Score:** 293 **Expect:** 2e-25 **Queries matched:** 3
J9USS2_BRAPL 60 kDa chaperonin OS=Brachyspira pilosicoli B2904 GN=groL PE=3 SV=1

Observed	Mr(expt)	Mr(calc)	ppm	Start	End	Miss	Ions	Peptide
1276.6064	1275.5991	1275.6571	-45.46	5 -	14	1	53	K.QLLFDEEARR.S
1811.8038	1810.7965	1810.8771	-44.50	182 -	197	0	114	K.SLETSLSLVEGMQFDR.G
2097.0054	2095.9981	2096.0902	-43.93	405 -	425	0	87	R.AAVEEGVIPGGGITYLHAQSK.L

No match to: 1259.5890, 2250.1423

2. D8IB78 **Mass:** 58110 **Score:** 293 **Expect:** 2e-25 **Queries matched:** 3
D8IB78_BRAP9 60 kDa chaperonin OS=Brachyspira pilosicoli (strain ATCC BAA-1826 / 95/1000) GN=groL PE=3 SV=1

Observed	Mr(expt)	Mr(calc)	ppm	Start	End	Miss	Ions	Peptide
1276.6064	1275.5991	1275.6571	-45.46	5 -	14	1	53	K.QLLFDEEARR.S
1811.8038	1810.7965	1810.8771	-44.50	182 -	197	0	114	K.SLETSLSLVEGMQFDR.G
2097.0054	2095.9981	2096.0902	-43.93	405 -	425	0	87	R.AAVEEGVIPGGGITYLHAQSK.L

No match to: 1259.5890, 2250.1423

3. K0JLL7 **Mass:** 58110 **Score:** 293 **Expect:** 2e-25 **Queries matched:** 3
K0JLL7_BRAPL 60 kDa chaperonin OS=Brachyspira pilosicoli WesB GN=groL PE=3 SV=1

Observed	Mr(expt)	Mr(calc)	ppm	Start	End	Miss	Ions	Peptide
1276.6064	1275.5991	1275.6571	-45.46	5 -	14	1	53	K.QLLFDEEARR.S
1811.8038	1810.7965	1810.8771	-44.50	182 -	197	0	114	K.SLETSLSLVEGMQFDR.G
2097.0054	2095.9981	2096.0902	-43.93	405 -	425	0	87	R.AAVEEGVIPGGGITYLHAQSK.L

No match to: 1259.5890, 2250.1423

4. L0X051 **Mass:** 58181 **Score:** 205 **Expect:** 1.3e-16 **Queries matched:** 3
L0X051_9SPIR 60 kDa chaperonin OS=Brachyspira hampsonii 30446 GN=groL PE=3 SV=1

Observed	Mr(expt)	Mr(calc)	ppm	Start	End	Miss	Ions	Peptide
1276.6064	1275.5991	1275.6571	-45.46	5 -	14	1	53	K.QLLFDEEARR.A
1811.8038	1810.7965	1810.8771	-44.50	182 -	197	0	114	K.SLETSLSLVEGMQFDR.G
2097.0054	2095.9981	2096.1001	-48.65	426 -	444	0	---	K.LESLTLENPDEQVGVNIVK.R

No match to: 1259.5890, 2250.1423

5. A0A0H0V6M8 **Mass:** 58212 **Score:** 189 **Expect:** 5.1e-15 **Queries matched:** 2
A0A0H0V6M8_BRAHO 60 kDa chaperonin OS=Brachyspira hyodysenteriae GN=groEL PE=3 SV=1

Observed	Mr(expt)	Mr(calc)	ppm	Start	End	Miss	Ions	Peptide
1276.6064	1275.5991	1275.6571	-45.46	5 -	14	1	53	K.QLLFDEEARR.A
1811.8038	1810.7965	1810.8771	-44.50	182 -	197	0	114	K.SLETSLSLVEGMQFDR.G

No match to: 1259.5890, 2097.0054, 2250.1423

6. A0A0H0TIS1 **Mass:** 58228 **Score:** 189 **Expect:** 5.1e-15 **Queries matched:** 2
A0A0H0TIS1_BRAHO 60 kDa chaperonin OS=Brachyspira hyodysenteriae GN=groEL PE=3 SV=1

Observed	Mr(expt)	Mr(calc)	ppm	Start	End	Miss	Ions	Peptide
1276.6064	1275.5991	1275.6571	-45.46	5 -	14	1	53	K.QLLFDEEARR.A
1811.8038	1810.7965	1810.8771	-44.50	182 -	197	0	114	K.SLETSLSLVEGMQFDR.G

No match to: 1259.5890, 2097.0054, 2250.1423

7. A0A0G4K5P2 **Mass:** 58212 **Score:** 189 **Expect:** 5.1e-15 **Queries matched:** 2
A0A0G4K5P2_9SPIR 60 kDa chaperonin OS=Brachyspira suanatina GN=groL PE=3 SV=1

Observed	Mr(expt)	Mr(calc)	ppm	Start	End	Miss	Ions	Peptide
1276.6064	1275.5991	1275.6571	-45.46	5 -	14	1	53	K.QLLFDEEARR.A
1811.8038	1810.7965	1810.8771	-44.50	182 -	197	0	114	K.SLETSLSLVEGMQFDR.G

No match to: 1259.5890, 2097.0054, 2250.1423

8. C0QWM4 **Mass:** 58212 **Score:** 189 **Expect:** 5.1e-15 **Queries matched:** 2
C0QWM4_BRAHW 60 kDa chaperonin OS=Brachyspira hyodysenteriae (strain ATCC 49526 / WA1) GN=groL PE=3 SV=1

Observed	Mr(expt)	Mr(calc)	ppm	Start	End	Miss	Ions	Peptide
1276.6064	1275.5991	1275.6571	-45.46	5 -	14	1	53	K.QLLFDEEARR.A
1811.8038	1810.7965	1810.8771	-44.50	182 -	197	0	114	K.SLETSLSLVEGMQFDR.G

No match to: 1259.5890, 2097.0054, 2250.1423

9. G0EJ80 **Mass:** 58197 **Score:** 189 **Expect:** 5.1e-15 **Queries matched:** 2
G0EJ80_BRAIP 60 kDa chaperonin OS=Brachyspira intermedia (strain ATCC 51140 / PWS/A) GN=groL PE=3 SV=1

Observed	Mr(expt)	Mr(calc)	ppm	Start	End	Miss	Ions	Peptide
1276.6064	1275.5991	1275.6571	-45.46	5 -	14	1	53	K.QLLFDEEARR.A
1811.8038	1810.7965	1810.8771	-44.50	182 -	197	0	114	K.SLETSLSLVEGMQFDR.G

No match to: 1259.5890, 2097.0054, 2250.1423

10. D5U544 **Mass:** 58148 **Score:** 189 **Expect:** 5.1e-15 **Queries matched:** 2
D5U544_BRAM5 60 kDa chaperonin OS=Brachyspira murdochii (strain ATCC 51284 / DSM 12563 / 56-150) GN=groL PE=3 SV=1

Observed	Mr(expt)	Mr(calc)	ppm	Start	End	Miss	Ions	Peptide
1276.6064	1275.5991	1275.6571	-45.46	5 -	14	1	53	K.QLLFDEEARR.A
1811.8038	1810.7965	1810.8771	-44.50	182 -	197	0	114	K.SLETSLSLVEGMQFDR.G

No match to: 1259.5890, 2097.0054, 2250.1423

11. L8XV88 **Mass:** 58195 **Score:** 189 **Expect:** 5.1e-15 **Queries matched:** 2
L8XV88_9SPIR 60 kDa chaperonin OS=Brachyspira hampsonii 30599 GN=groL PE=3 SV=1

Observed	Mr(expt)	Mr(calc)	ppm	Start	End	Miss	Ions	Peptide
1276.6064	1275.5991	1275.6571	-45.46	5 -	14	1	53	K.QLLFDEEARR.A
1811.8038	1810.7965	1810.8771	-44.50	182 -	197	0	114	K.SLETSLSLVEGMQFDR.G

No match to: 1259.5890, 2097.0054, 2250.1423

12. T1WGB0 **Mass:** 20074 **Score:** 146 **Expect:** 1e-10 **Queries matched:** 2
T1WGB0_9SPIR 60 kDa chaperonin (Fragment) OS=Brachyspira sp. CH-003 GN=cpn60 PE=4 SV=1

Observed	Mr(expt)	Mr(calc)	ppm	Start	End	Miss	Ions	Peptide
1276.6064	1275.5991	1275.6380	-30.49	66 -	77	0	---	K.EIGALISDAMEK.V
1811.8038	1810.7965	1810.8771	-44.50	91 -	106	0	114	K.SLETSLSLVEGMQFDR.G

No match to: 1259.5890, 2097.0054, 2250.1423

13. T1WFK2 **Mass:** 20144 **Score:** 146 **Expect:** 1e-10 **Queries matched:** 2
T1WFK2_9SPIR 60 kDa chaperonin (Fragment) OS=Brachyspira sp. SHI-129 GN=cpn60 PE=4 SV=1

Observed	Mr(expt)	Mr(calc)	ppm	Start	End	Miss	Ions	Peptide
1276.6064	1275.5991	1275.6380	-30.49	66 -	77	0	---	K.EIGALISDAMEK.V
1811.8038	1810.7965	1810.8771	-44.50	91 -	106	0	114	K.SLETSLSLVEGMQFDR.G

No match to: 1259.5890, 2097.0054, 2250.1423

14. T1WGB4 **Mass:** 20074 **Score:** 146 **Expect:** 1e-10 **Queries matched:** 2
T1WGB4_9SPIR 60 kDa chaperonin (Fragment) OS=Brachyspira hampsonii bv. II GN=cpn60 PE=4 SV=1

Observed	Mr(expt)	Mr(calc)	ppm	Start	End	Miss	Ions	Peptide
1276.6064	1275.5991	1275.6380	-30.49	66 -	77	0	---	K.EIGALISDAMEK.V
1811.8038	1810.7965	1810.8771	-44.50	91 -	106	0	114	K.SLETSLSLVEGMQFDR.G

No match to: 1259.5890, 2097.0054, 2250.1423

15. T1WFJ6 **Mass:** 20144 **Score:** 146 **Expect:** 1e-10 **Queries matched:** 2
T1WFJ6_9SPIR 60 kDa chaperonin (Fragment) OS=Brachyspira sp. SHI-031 GN=cpn60 PE=4 SV=1

Observed	Mr(expt)	Mr(calc)	ppm	Start	End	Miss	Ions	Peptide
1276.6064	1275.5991	1275.6380	-30.49	66 -	77	0	---	K.EIGALISDAMEK.V
1811.8038	1810.7965	1810.8771	-44.50	91 -	106	0	114	K.SLETSLSLVEGMQFDR.G

No match to: 1259.5890, 2097.0054, 2250.1423

16. Q3YLA3 **Mass:** 19965 **Score:** 146 **Expect:** 1e-10 **Queries matched:** 2
Q3YLA3_BRAPL 60 kDa chaperonin (Fragment) OS=Brachyspira pilosicoli GN=cpn60 PE=4 SV=1

Observed	Mr(expt)	Mr(calc)	ppm	Start	End	Miss	Ions	Peptide
1276.6064	1275.5991	1275.6380	-30.49	66 -	77	0	---	K.EIGALISDAMEK.V
1811.8038	1810.7965	1810.8771	-44.50	91 -	106	0	114	K.SLETSLSLVEGMQFDR.G

No match to: 1259.5890, 2097.0054, 2250.1423

17. T1WGB9 **Mass:** 20103 **Score:** 128 **Expect:** 6.4e-09 **Queries matched:** 1
T1WGB9_9SPIR 60 kDa chaperonin (Fragment) OS=Brachyspira sp. SHI-067 GN=cpn60 PE=4 SV=1

Observed	Mr(expt)	Mr(calc)	ppm	Start	End	Miss	Ions	Peptide
1811.8038	1810.7965	1810.8771	-44.50	91 -	106	0	114	K.SLETSLSLVEGMQFDR.G

No match to: 1259.5890, 1276.6064, 2097.0054, 2250.1423

18. Q3YL98 **Mass:** 20103 **Score:** 128 **Expect:** 6.4e-09 **Queries matched:** 1
Q3YL98_9SPIR 60 kDa chaperonin (Fragment) OS=Brachyspira murdochii GN=cpn60 PE=4 SV=1

Observed	Mr(expt)	Mr(calc)	ppm	Start	End	Miss	Ions	Peptide
1811.8038	1810.7965	1810.8771	-44.50	91 -	106	0	114	K.SLETSLSLVEGMQFDR.G

No match to: 1259.5890, 1276.6064, 2097.0054, 2250.1423

19. T1WFJ0 **Mass:** 20174 **Score:** 128 **Expect:** 6.4e-09 **Queries matched:** 1
T1WFJ0_9SPIR 60 kDa chaperonin (Fragment) OS=Brachyspira sp. KL-169 GN=cpn60 PE=4 SV=1

Observed	Mr(expt)	Mr(calc)	ppm	Start	End	Miss	Ions	Peptide
1811.8038	1810.7965	1810.8771	-44.50	91 -	106	0	114	K.SLETSLSLVEGMQFDR.G

No match to: 1259.5890, 1276.6064, 2097.0054, 2250.1423

20. Q3YLA1 **Mass:** 20036 **Score:** 128 **Expect:** 6.4e-09 **Queries matched:** 1
Q3YLA1_BRAHO 60 kDa chaperonin (Fragment) OS=Brachyspira hyodysenteriae GN=cpn60 PE=4 SV=1

Observed	Mr(expt)	Mr(calc)	ppm	Start	End	Miss	Ions	Peptide
1811.8038	1810.7965	1810.8771	-44.50	91 -	106	0	114	K.SLETSLSLVEGMQFDR.G

No match to: 1259.5890, 1276.6064, 2097.0054, 2250.1423

21. T1WF97 **Mass:** 20097 **Score:** 128 **Expect:** 6.4e-09 **Queries matched:** 1
T1WF97_9SPIR 60 kDa chaperonin (Fragment) OS=Brachyspira sp. KL-181 GN=cpn60 PE=4 SV=1

Observed	Mr(expt)	Mr(calc)	ppm	Start	End	Miss	Ions	Peptide
1811.8038	1810.7965	1810.8771	-44.50	91 -	106	0	114	K.SLETSLSLVEGMQFDR.G

No match to: 1259.5890, 1276.6064, 2097.0054, 2250.1423

22. T1WF92 **Mass:** 20075 **Score:** 128 **Expect:** 6.4e-09 **Queries matched:** 1
T1WF92_9SPIR 60 kDa chaperonin (Fragment) OS=Brachyspira sp. CH-013 GN=cpn60 PE=4 SV=1

Observed	Mr(expt)	Mr(calc)	ppm	Start	End	Miss	Ions	Peptide
1811.8038	1810.7965	1810.8771	-44.50	91 -	106	0	114	K.SLETSLSLVEGMQFDR.G

No match to: 1259.5890, 1276.6064, 2097.0054, 2250.1423

23. Q3YLA0 **Mass:** 20083 **Score:** 128 **Expect:** 6.4e-09 **Queries matched:** 1
Q3YLA0_9SPIR 60 kDa chaperonin (Fragment) OS=Brachyspira innocens GN=cpn60 PE=4 SV=1

Observed	Mr(expt)	Mr(calc)	ppm	Start	End	Miss	Ions	Peptide
1811.8038	1810.7965	1810.8771	-44.50	91 -	106	0	114	K.SLETSLSLVEGMQFDR.G

No match to: 1259.5890, 1276.6064, 2097.0054, 2250.1423

24. T1WEF0 **Mass:** 20103 **Score:** 128 **Expect:** 6.4e-09 **Queries matched:** 1
T1WEF0_9SPIR 60 kDa chaperonin (Fragment) OS=Brachyspira sp. CH-014 GN=cpn60 PE=4 SV=1

Observed	Mr(expt)	Mr(calc)	ppm	Start	End	Miss	Ions	Peptide
1811.8038	1810.7965	1810.8771	-44.50	91 -	106	0	114	K.SLETSLSLVEGMQFDR.G

No match to: 1259.5890, 1276.6064, 2097.0054, 2250.1423

25. Q3YL99 **Mass:** 20091 **Score:** 128 **Expect:** 6.4e-09 **Queries matched:** 1
Q3YL99_9SPIR 60 kDa chaperonin (Fragment) OS=Brachyspira alvinipulli GN=cpn60 PE=4 SV=1

Observed	Mr(expt)	Mr(calc)	ppm	Start	End	Miss	Ions	Peptide
1811.8038	1810.7965	1810.8771	-44.50	91 -	106	0	114	K.SLETSLSLVEGMQFDR.G

No match to: 1259.5890, 1276.6064, 2097.0054, 2250.1423

26. T1WEW3 **Mass:** 20103 **Score:** 128 **Expect:** 6.4e-09 **Queries matched:** 1
T1WEW3_9SPIR 60 kDa chaperonin (Fragment) OS=Brachyspira sp. KL-207 GN=cpn60 PE=4 SV=1

Observed	Mr(expt)	Mr(calc)	ppm	Start	End	Miss	Ions	Peptide
1811.8038	1810.7965	1810.8771	-44.50	91 -	106	0	114	K.SLETSLSLVEGMQFDR.G

No match to: 1259.5890, 1276.6064, 2097.0054, 2250.1423

27. T1WFA1 **Mass:** 20103 **Score:** 128 **Expect:** 6.4e-09 **Queries matched:** 1
T1WFA1_9SPIR 60 kDa chaperonin (Fragment) OS=Brachyspira sp. SHI-068 GN=cpn60 PE=4 SV=1

Observed	Mr(expt)	Mr(calc)	ppm	Start	End	Miss	Ions	Peptide
1811.8038	1810.7965	1810.8771	-44.50	91 -	106	0	114	K.SLETSLSLVEGMQFDR.G

No match to: 1259.5890, 1276.6064, 2097.0054, 2250.1423

28. G1EI49 **Mass:** 20092 **Score:** 128 **Expect:** 6.4e-09 **Queries matched:** 1
G1EI49_9SPIR 60 kDa chaperonin (Fragment) OS=Brachyspira intermedia GN=cpn60 PE=4 SV=1

Observed	Mr(expt)	Mr(calc)	ppm	Start	End	Miss	Ions	Peptide
1811.8038	1810.7965	1810.8771	-44.50	91 -	106	0	114	K.SLETSLSLVEGMQFDR.G

No match to: 1259.5890, 1276.6064, 2097.0054, 2250.1423

29. F8UU24 **Mass:** 20092 **Score:** 128 **Expect:** 6.4e-09 **Queries matched:** 1
F8UU24_9SPIR CPN60 (Fragment) OS=Brachyspira sp. j1330 GN=cpn60 PE=4 SV=1

Observed	Mr(expt)	Mr(calc)	ppm	Start	End	Miss	Ions	Peptide
1811.8038	1810.7965	1810.8771	-44.50	91 -	106	0	114	K.SLETSLSLVEGMQFDR.G

No match to: 1259.5890, 1276.6064, 2097.0054, 2250.1423

30. T1WEG1 **Mass:** 20103 **Score:** 128 **Expect:** 6.4e-09 **Queries matched:** 1
T1WEG1_9SPIR 60 kDa chaperonin (Fragment) OS=Brachyspira sp. CH-108 GN=cpn60 PE=4 SV=1

Observed	Mr(expt)	Mr(calc)	ppm	Start	End	Miss	Ions	Peptide
1811.8038	1810.7965	1810.8771	-44.50	91 -	106	0	114	K.SLETSLSLVEGMQFDR.G

No match to: 1259.5890, 1276.6064, 2097.0054, 2250.1423

31. T1WEV9 **Mass:** 20100 **Score:** 128 **Expect:** 6.4e-09 **Queries matched:** 1
T1WEV9_9SPIR 60 kDa chaperonin (Fragment) OS=Brachyspira sp. KL-049 GN=cpn60 PE=4 SV=1

Observed	Mr(expt)	Mr(calc)	ppm	Start	End	Miss	Ions	Peptide
1811.8038	1810.7965	1810.8771	-44.50	91 -	106	0	114	K.SLETSLSLVEGMQFDR.G

No match to: 1259.5890, 1276.6064, 2097.0054, 2250.1423

32. T1WEW8 **Mass:** 20103 **Score:** 128 **Expect:** 6.4e-09 **Queries matched:** 1
T1WEW8_9SPIR 60 kDa chaperonin (Fragment) OS=Brachyspira sp. CH-110 GN=cpn60 PE=4 SV=1

Observed	Mr(expt)	Mr(calc)	ppm	Start	End	Miss	Ions	Peptide
1811.8038	1810.7965	1810.8771	-44.50	91 -	106	0	114	K.SLETSLSLVEGMQFDR.G

No match to: 1259.5890, 1276.6064, 2097.0054, 2250.1423

33. T1WEF7 **Mass:** 20075 **Score:** 128 **Expect:** 6.4e-09 **Queries matched:** 1
T1WEF7_9SPIR 60 kDa chaperonin (Fragment) OS=Brachyspira sp. KL-194 GN=cpn60 PE=4 SV=1

Observed	Mr(expt)	Mr(calc)	ppm	Start	End	Miss	Ions	Peptide
1811.8038	1810.7965	1810.8771	-44.50	91 -	106	0	114	K.SLETSLSLVEGMQFDR.G

No match to: 1259.5890, 1276.6064, 2097.0054, 2250.1423

34. R5LQ94 **Mass:** 58190 **Score:** 62 **Expect:** 0.026 **Queries matched:** 1
R5LQ94_9SPIR 60 kDa chaperonin OS=Brachyspira sp. CAG:700 GN=groL PE=3 SV=1

Observed	Mr(expt)	Mr(calc)	ppm	Start	End	Miss	Ions	Peptide
1276.6064	1275.5991	1275.6571	-45.46	5 -	14	1	53	K.QLLFDEEARR.S

No match to: 1259.5890, 1811.8038, 2097.0054, 2250.1423

35. A0A0H0UCV1 **Mass:** 38722 **Score:** 41 **Expect:** 2.9 **Queries matched:** 2
A0A0H0UCV1_BRAHO Molecular chaperone GroES OS=Brachyspira hyodysenteriae GN=SZ47_11215 PE=3 SV=1

Observed	Mr(expt)	Mr(calc)	ppm	Start	End	Miss	Ions	Peptide
2097.0054	2095.9981	2095.9568	19.7	107 -	124	1	---	K.GGWEIGCRINGCQAEYVK.V
2250.1423	2249.1350	2249.0603	33.2	165 -	186	0	12	K.NNDTIAVIGSGPVGLCAMMSAR.V + Oxidation (M)

No match to: 1259.5890, 1276.6064, 1811.8038

Search Parameters

Type of search : Sequence Query
Enzyme : Trypsin
Fixed modifications : Carbamidomethyl (C)
Variable modifications : Oxidation (M)
Mass values : Monoisotopic
Protein Mass : Unrestricted
Peptide Mass Tolerance : ± 50 ppm
Fragment Mass Tolerance: ± 0.5 Da
Max Missed Cleavages : 1
Instrument type : MALDI-TOF-TOF
Query1 (1259.5890,1+) : Locus:15.1.1.211150.56587
Query2 (1276.6064,1+) : Locus:15.1.1.211150.56585
Query3 (1811.8038,1+) : Locus:15.1.1.211150.56586
Query4 (2097.0054,1+) : Locus:15.1.1.211150.56589
Query5 (2250.1423,1+) : Locus:15.1.1.211150.56588

Mascot: http://www.matrixscience.com/

Mascot Search Results

```
User          :
Email         :
Search title  : F:\ROD_inmunoproteoma\MALDI\ppw_2016_562_563_ROD\ppw_I17_145397050633.txt
MS data file  : F:\ROD_inmunoproteoma\MALDI\ppw_2016_562_563_ROD\ppw_I17_145397050633.txt
Database      : Brachyspiraceae Brachyspiraceae_20160127-151122 (40573 sequences; 13470793 residues)
Timestamp     : 23 Feb 2016 at 16:06:27 GMT
Warning       : A Peptide summary report will usually give a much clearer picture of MS/MS search results.
Top Score     : 172 for A0A0H0V6M8, A0A0H0V6M8_BRAHO 60 kDa chaperonin OS=Brachyspira hyodysenteriae GN=groEL PE=3 SV=1
```

Probability Based Mowse Score

Protein score is -10*Log(P), where P is the probability that the observed match is a random event.
Protein scores greater than 59 are significant (p<0.05).
Protein scores are derived from ions scores as a non-probabilistic basis for ranking protein hits.

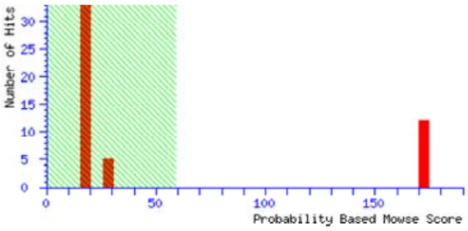

Protein Summary Report

Format As | Protein Summary (deprecated) | Help

Significance threshold p< 0.05 Max. number of hits AUTO

Standard scoring ◉ MudPIT scoring ○ Ions score or expect cut-off 0 Show sub-sets 0

Show pop-ups ◉ Suppress pop-ups ○ Sort unassigned Decreasing Score Require bold red ☐

Re-Search All | Search Unmatched

Index

	Accession	Mass	Score	Description
1.	A0A0H0V6M8	58212	172	A0A0H0V6M8_BRAHO 60 kDa chaperonin OS=Brachyspira hyodysenteriae GN=groEL PE=3 SV=1
2.	A0A0H0TIS1	58228	172	A0A0H0TIS1_BRAHO 60 kDa chaperonin OS=Brachyspira hyodysenteriae GN=groEL PE=3 SV=1
3.	A0A0G4K5P2	58212	172	A0A0G4K5P2_9SPIR 60 kDa chaperonin OS=Brachyspira suanatina GN=groL PE=3 SV=1
4.	R5LQ94	58190	172	R5LQ94_9SPIR 60 kDa chaperonin OS=Brachyspira sp. CAG:700 GN=groL PE=3 SV=1
5.	C0QWM4	58212	172	C0QWM4_BRAHW 60 kDa chaperonin OS=Brachyspira hyodysenteriae (strain ATCC 49526 / WA1) GN=groL PE=3 SV=1
6.	G0EJ80	58197	172	G0EJ80_BRAIP 60 kDa chaperonin OS=Brachyspira intermedia (strain ATCC 51140 / PWS/A) GN=groL PE=3 SV=1
7.	D5U544	58148	172	D5U544_BRAM5 60 kDa chaperonin OS=Brachyspira murdochii (strain ATCC 51284 / DSM 12563 / 56-150) GN=groL PE=3
8.	L8XV88	58195	172	L8XV88_9SPIR 60 kDa chaperonin OS=Brachyspira hampsonii 30599 GN=groL PE=3 SV=1
9.	J9USS2	58110	171	J9USS2_BRAPL 60 kDa chaperonin OS=Brachyspira pilosicoli B2904 GN=groL PE=3 SV=1
10.	D8IB78	58110	171	D8IB78_BRAP9 60 kDa chaperonin OS=Brachyspira pilosicoli (strain ATCC BAA-1826 / 95/1000) GN=groL PE=3 SV=1
11.	K0JLL7	58110	171	K0JLL7_BRAPL 60 kDa chaperonin OS=Brachyspira pilosicoli WesB GN=groL PE=3 SV=1
12.	L0X051	58181	171	L0X051_9SPIR 60 kDa chaperonin OS=Brachyspira hampsonii 30446 GN=groL PE=3 SV=1
13.	L8XL53	19713	26	L8XL53_9SPIR Site-specific DNA-methyltransferase OS=Brachyspira hampsonii 30599 GN=H263_14015 PE=4 SV=1

Results List

1. A0A0H0V6M8 **Mass:** 58212 **Score:** 172 **Expect:** 2.6e-13 **Queries matched:** 2
A0A0H0V6M8_BRAHO 60 kDa chaperonin OS=Brachyspira hyodysenteriae GN=groEL PE=3 SV=1

Observed	Mr(expt)	Mr(calc)	ppm	Start		End	Miss	Ions	Peptide
1276.6273	1275.6200	1275.6571	-29.08	5	-	14	1	39	K.QLLFDEEARR.A
1580.8666	1579.8593	1579.9046	-28.65	19	-	34	1	110	R.GVDALANAVKVTLGPR.G

No match to: 1320.5509, 1791.6768, 2383.8765

2. A0A0H0TIS1 **Mass:** 58228 **Score:** 172 **Expect:** 2.6e-13 **Queries matched:** 2
A0A0H0TIS1_BRAHO 60 kDa chaperonin OS=Brachyspira hyodysenteriae GN=groEL PE=3 SV=1

Observed	Mr(expt)	Mr(calc)	ppm	Start		End	Miss	Ions	Peptide
1276.6273	1275.6200	1275.6571	-29.08	5	-	14	1	39	K.QLLFDEEARR.A
1580.8666	1579.8593	1579.9046	-28.65	19	-	34	1	110	R.GVDALANAVKVTLGPR.G

No match to: 1320.5509, 1791.6768, 2383.8765

3. A0A0G4K5P2 **Mass:** 58212 **Score:** 172 **Expect:** 2.6e-13 **Queries matched:** 2
A0A0G4K5P2_9SPIR 60 kDa chaperonin OS=Brachyspira suanatina GN=groL PE=3 SV=1

Observed	Mr(expt)	Mr(calc)	ppm	Start		End	Miss	Ions	Peptide
1276.6273	1275.6200	1275.6571	-29.08	5	-	14	1	39	K.QLLFDEEARR.A
1580.8666	1579.8593	1579.9046	-28.65	19	-	34	1	110	R.GVDALANAVKVTLGPR.G

No match to: 1320.5509, 1791.6768, 2383.8765

4. R5LQ94 **Mass:** 58190 **Score:** 172 **Expect:** 2.6e-13 **Queries matched:** 2
R5LQ94_9SPIR 60 kDa chaperonin OS=Brachyspira sp. CAG:700 GN=groL PE=3 SV=1

Observed	Mr(expt)	Mr(calc)	ppm	Start		End	Miss	Ions	Peptide
1276.6273	1275.6200	1275.6571	-29.08	5	-	14	1	39	K.QLLFDEEARR.S
1580.8666	1579.8593	1579.9046	-28.65	19	-	34	1	110	R.GVDALANAVKVTLGPR.G

No match to: 1320.5509, 1791.6768, 2383.8765

5. C0QWM4 **Mass:** 58212 **Score:** 172 **Expect:** 2.6e-13 **Queries matched:** 2
C0QWM4_BRAHW 60 kDa chaperonin OS=Brachyspira hyodysenteriae (strain ATCC 49526 / WA1) GN=groL PE=3 SV=1

Observed	Mr(expt)	Mr(calc)	ppm	Start		End	Miss	Ions	Peptide
1276.6273	1275.6200	1275.6571	-29.08	5	-	14	1	39	K.QLLFDEEARR.A
1580.8666	1579.8593	1579.9046	-28.65	19	-	34	1	110	R.GVDALANAVKVTLGPR.G

No match to: 1320.5509, 1791.6768, 2383.8765

6. G0EJ80 **Mass:** 58197 **Score:** 172 **Expect:** 2.6e-13 **Queries matched:** 2
G0EJ80_BRAIP 60 kDa chaperonin OS=Brachyspira intermedia (strain ATCC 51140 / PWS/A) GN=groL PE=3 SV=1

Observed	Mr(expt)	Mr(calc)	ppm	Start		End	Miss	Ions	Peptide
1276.6273	1275.6200	1275.6571	-29.08	5	-	14	1	39	K.QLLFDEEARR.A
1580.8666	1579.8593	1579.9046	-28.65	19	-	34	1	110	R.GVDALANAVKVTLGPR.G

No match to: 1320.5509, 1791.6768, 2383.8765

7. D5U544 **Mass:** 58148 **Score:** 172 **Expect:** 2.6e-13 **Queries matched:** 2
D5U544_BRAM5 60 kDa chaperonin OS=Brachyspira murdochii (strain ATCC 51284 / DSM 12563 / 56-150) GN=groL PE=3 SV=1

Observed	Mr(expt)	Mr(calc)	ppm	Start		End	Miss	Ions	Peptide
1276.6273	1275.6200	1275.6571	-29.08	5	-	14	1	39	K.QLLFDEEARR.A
1580.8666	1579.8593	1579.9046	-28.65	19	-	34	1	110	R.GVDALANAVKVTLGPR.G

No match to: 1320.5509, 1791.6768, 2383.8765

8. L8XV88 **Mass:** 58195 **Score:** 172 **Expect:** 2.6e-13 **Queries matched:** 2
L8XV88_9SPIR 60 kDa chaperonin OS=Brachyspira hampsonii 30599 GN=groL PE=3 SV=1

Observed	Mr(expt)	Mr(calc)	ppm	Start		End	Miss	Ions	Peptide
1276.6273	1275.6200	1275.6571	-29.08	5	-	14	1	39	K.QLLFDEEARR.A
1580.8666	1579.8593	1579.9046	-28.65	19	-	34	1	110	R.GVDALANAVKVTLGPR.G

No match to: 1320.5509, 1791.6768, 2383.8765

9. J9USS2 **Mass:** 58110 **Score:** 171 **Expect:** 3.2e-13 **Queries matched:** 2
J9USS2_BRAPL 60 kDa chaperonin OS=Brachyspira pilosicoli B2904 GN=groL PE=3 SV=1

Observed	Mr(expt)	Mr(calc)	ppm	Start		End	Miss	Ions	Peptide
1276.6273	1275.6200	1275.6571	-29.08	5	-	14	1	39	K.QLLFDEEARR.S
1580.8666	1579.8593	1579.9046	-28.65	19	-	34	1	110	R.GVDALANAVKVTLGPR.G

No match to: 1320.5509, 1791.6768, 2383.8765

10. D8IB78 **Mass:** 58110 **Score:** 171 **Expect:** 3.2e-13 **Queries matched:** 2
D8IB78_BRAP9 60 kDa chaperonin OS=Brachyspira pilosicoli (strain ATCC BAA-1826 / 95/1000) GN=groL PE=3 SV=1

Observed	Mr(expt)	Mr(calc)	ppm	Start		End	Miss	Ions	Peptide
1276.6273	1275.6200	1275.6571	-29.08	5	-	14	1	39	K.QLLFDEEARR.S
1580.8666	1579.8593	1579.9046	-28.65	19	-	34	1	110	R.GVDALANAVKVTLGPR.G

No match to: 1320.5509, 1791.6768, 2383.8765

11. K0JLL7 **Mass:** 58110 **Score:** 171 **Expect:** 3.2e-13 **Queries matched:** 2
K0JLL7_BRAPL 60 kDa chaperonin OS=Brachyspira pilosicoli WesB GN=groL PE=3 SV=1

Observed	Mr(expt)	Mr(calc)	ppm	Start		End	Miss	Ions	Peptide
1276.6273	1275.6200	1275.6571	-29.08	5	-	14	1	39	K.QLLFDEEARR.S
1580.8666	1579.8593	1579.9046	-28.65	19	-	34	1	110	R.GVDALANAVKVTLGPR.G

No match to: 1320.5509, 1791.6768, 2383.8765

12. L0X051 **Mass:** 58181 **Score:** 171 **Expect:** 3.2e-13 **Queries matched:** 2
L0X051_9SPIR 60 kDa chaperonin OS=Brachyspira hampsonii 30446 GN=groL PE=3 SV=1

Observed	Mr(expt)	Mr(calc)	ppm	Start		End	Miss	Ions	Peptide
1276.6273	1275.6200	1275.6571	-29.08	5	-	14	1	39	K.QLLFDEEARR.A
1580.8666	1579.8593	1579.9046	-28.65	19	-	34	1	110	R.GVDALANAVKVTLGPR.G

No match to: 1320.5509, 1791.6768, 2383.8765

13. L8XL53 **Mass:** 19713 **Score:** 26 **Expect:** 1e+02 **Queries matched:** 1
L8XL53_9SPIR Site-specific DNA-methyltransferase OS=Brachyspira hampsonii 30599 GN=H263_14015 PE=4 SV=1

Observed	Mr(expt)	Mr(calc)	ppm	Start		End	Miss	Ions	Peptide
1276.6273	1275.6200	1275.6306	-8.33	2	-	12	1	12	M.ITNTEDNVKDK.Y

No match to: 1320.5509, 1580.8666, 1791.6768, 2383.8765

Search Parameters

Type of search : Sequence Query
Enzyme : Trypsin
Fixed modifications : Carbamidomethyl (C)
Variable modifications : Oxidation (M)
Mass values : Monoisotopic
Protein Mass : Unrestricted
Peptide Mass Tolerance : ± 50 ppm
Fragment Mass Tolerance: ± 0.5 Da
Max Missed Cleavages : 1
Instrument type : MALDI-TOF-TOF
Query1 (1276.6273,1+) : Locus:34.1.1.211181.56681
Query2 (1320.5509,1+) : Locus:34.1.1.211181.56683
Query3 (1580.8666,1+) : Locus:34.1.1.211181.56680
Query4 (1791.6768,1+) : Locus:34.1.1.211181.56682
Query5 (2383.8765,1+) : Locus:34.1.1.211181.56684

Mascot: http://www.matrixscience.com/

Mascot Search Results

User :
Email :
Search title : F:\ROD_inmunoproteoma\MALDI\ppw_2014_442_ROD\ppw_G6_139601421305.txt
MS data file : F:\ROD_inmunoproteoma\MALDI\ppw_2014_442_ROD\ppw_G6_139601421305.txt
Database : Brachyspiraceae Brachyspiraceae_20160127-151122 (40573 sequences; 13470793 residues)
Timestamp : 22 Feb 2016 at 15:14:00 GMT
Warning : A Peptide summary report will usually give a much clearer picture of MS/MS search results.
Top Score : 133 for J9UT77, J9UT77_BRAPL TmpB family outer membrane protein OS=Brachyspira pilosicoli B2904 GN=B2904_orf843 PE=4 SV=1

Probability Based Mowse Score

Protein score is -10*Log(P), where P is the probability that the observed match is a random event.
Protein scores greater than 59 are significant (p<0.05).
Protein scores are derived from ions scores as a non-probabilistic basis for ranking protein hits.

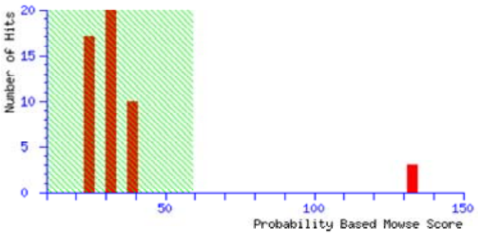

Protein Summary Report

Format As: Protein Summary (deprecated) — Help

Significance threshold p< 0.05 — Max. number of hits AUTO

Standard scoring ◉ MudPIT scoring ○ — Ions score or expect cut-off 0 — Show sub-sets 0

Show pop-ups ◉ Suppress pop-ups ○ — Sort unassigned Decreasing Score — Require bold red ☐

Re-Search All — Search Unmatched

Index

	Accession	Mass	Score	Description
1.	J9UT77	42496	133	J9UT77_BRAPL TmpB family outer membrane protein OS=Brachyspira pilosicoli B2904 GN=B2904_orf843 PE=4 SV=1
2.	D8IBK7	42481	133	D8IBK7_BRAP9 Outer membrane protein, TmpB family OS=Brachyspira pilosicoli (strain ATCC BAA-1826 / 95/1000) GN=
3.	K0JLI6	42497	133	K0JLI6_BRAPL TmpB family outer membrane protein OS=Brachyspira pilosicoli WesB GN=WESB_1830 PE=4 SV=1
4.	A0A0H0UY13	11812	42	A0A0H0UY13_BRAHO Uncharacterized protein OS=Brachyspira hyodysenteriae GN=SZ49_06190 PE=4 SV=1

Results List

1. J9UT77 **Mass:** 42496 **Score:** 133 **Expect:** 2e-09 **Queries matched:** 1
J9UT77_BRAPL TmpB family outer membrane protein OS=Brachyspira pilosicoli B2904 GN=B2904_orf843 PE=4 SV=1

Observed	Mr(expt)	Mr(calc)	ppm	Start	End	Miss	Ions	Peptide
1945.8859	1944.8786	1944.8701	4.39	361 -	377	1	119	R.DGTYDSNNEYGNIKDIK.-

No match to: 713.4000, 816.4357

2. D8IBK7 **Mass:** 42481 **Score:** 133 **Expect:** 2e-09 **Queries matched:** 1
D8IBK7_BRAP9 Outer membrane protein, TmpB family OS=Brachyspira pilosicoli (strain ATCC BAA-1826 / 95/1000) GN=tmpB PE=4 SV=1

Observed	Mr(expt)	Mr(calc)	ppm	Start	End	Miss	Ions	Peptide
1945.8859	1944.8786	1944.8701	4.39	361 -	377	1	119	R.DGTYDSNNEYGNIKDIK.-

No match to: 713.4000, 816.4357

3. K0JLI6 **Mass:** 42497 **Score:** 133 **Expect:** 2e-09 **Queries matched:** 1
K0JLI6_BRAPL TmpB family outer membrane protein OS=Brachyspira pilosicoli WesB GN=WESB_1830 PE=4 SV=1

Observed	Mr(expt)	Mr(calc)	ppm	Start	End	Miss	Ions	Peptide
1945.8859	1944.8786	1944.8701	4.39	361 -	377	1	119	R.DGTYDSNNEYGNIKDIK.-

No match to: 713.4000, 816.4357

4. A0A0H0UY13 **Mass:** 11812 **Score:** 42 **Expect:** 2.9 **Queries matched:** 2
A0A0H0UY13_BRAHO Uncharacterized protein OS=Brachyspira hyodysenteriae GN=SZ49_06190 PE=4 SV=1

Observed	Mr(expt)	Mr(calc)	ppm	Start	End	Miss	Ions	Peptide
816.4357	815.4284	815.4501	-26.59	31 -	37	1	---	K.NLENAKK.F
1945.8859	1944.8786	1944.9177	-20.10	81 -	96	1	---	K.DDETVVDQNKQNWLNK.F

No match to: 713.4000

Search Parameters

Type of search : Sequence Query
Enzyme : Trypsin
Fixed modifications : Carbamidomethyl (C)
Variable modifications : Oxidation (M)
Mass values : Monoisotopic
Protein Mass : Unrestricted
Peptide Mass Tolerance : ± 50 ppm
Fragment Mass Tolerance: ± 0.5 Da

```
Max Missed Cleavages   : 1
Instrument type        : MALDI-TOF-TOF
Query1 (713.4000,1+)   : Locus:6.1.1.170604.52750
Query2 (816.4357,1+)   : Locus:6.1.1.170604.52752
Query3 (1945.8859,1+)  : Locus:6.1.1.170604.52751
```

Mascot: http://www.matrixscience.com/

Mascot Search Results

**User**	:	
**Email**	:	
**Search title**	:	F:\ROD_inmunoproteoma\MALDI\ppw_2016_562_563_ROD\ppw_H9_145397048913.txt
**MS data file**	:	F:\ROD_inmunoproteoma\MALDI\ppw_2016_562_563_ROD\ppw_H9_145397048913.txt
**Database**	:	Brachyspiraceae Brachyspiraceae_20160127-151122 (40573 sequences; 13470793 residues)
**Timestamp**	:	23 Feb 2016 at 16:05:36 GMT
**Warning**	:	A Peptide summary report will usually give a much clearer picture of MS/MS search results.
**Top Score**	:	176 for J9UL67, J9UL67_BRAPL Carboxyl terminal protease OS=Brachyspira pilosicoli B2904 GN=B2904_orf2273 PE=3 SV=1

Probability Based Mowse Score

Protein score is -10*Log(P), where P is the probability that the observed match is a random event.
Protein scores greater than 59 are significant (p<0.05).
Protein scores are derived from ions scores as a non-probabilistic basis for ranking protein hits.

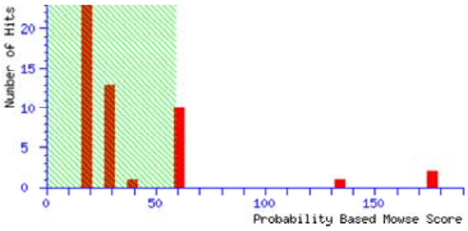

Protein Summary Report

Format As | Protein Summary (deprecated) | Help

Significance threshold p< 0.05 | Max. number of hits AUTO

Standard scoring ◉ MudPIT scoring ○ | Ions score or expect cut-off 0 | Show sub-sets 0

Show pop-ups ◉ Suppress pop-ups ○ | Sort unassigned Decreasing Score | Require bold red ☐

Re-Search All | Search Unmatched

Index

	Accession	Mass	Score	Description
1.	J9UL67	54900	176	J9UL67_BRAPL Carboxyl terminal protease OS=Brachyspira pilosicoli B2904 GN=B2904_orf2273 PE=3 SV=1
2.	K0JIZ9	54884	176	K0JIZ9_BRAPL Carboxyl terminal protease OS=Brachyspira pilosicoli WesB GN=WESB_0581 PE=3 SV=1
3.	D8IF04	50717	138	D8IF04_BRAP9 Carboxyl terminal protease OS=Brachyspira pilosicoli (strain ATCC BAA-1826 / 95/1000) GN=BP951000
4.	R5LDF9	54957	62	R5LDF9_9SPIR Carboxyl-terminal protease OS=Brachyspira sp. CAG:700 GN=BN758_01320 PE=3 SV=1
5.	A0A0H0U272	54982	62	A0A0H0U272_BRAHO Carboxyl-terminal protease OS=Brachyspira hyodysenteriae GN=SZ44_03720 PE=3 SV=1
6.	A0A0H0V600	54998	62	A0A0H0V600_BRAHO Carboxyl-terminal protease OS=Brachyspira hyodysenteriae GN=SR30_12295 PE=3 SV=1
7.	A0A0H0X0I2	55025	62	A0A0H0X0I2_BRAHO Carboxyl-terminal protease OS=Brachyspira hyodysenteriae GN=SU45_00880 PE=3 SV=1
8.	A0A0G4KAT6	54978	62	A0A0G4KAT6_9SPIR Carboxyl-terminal protease OS=Brachyspira suanatina GN=BRSU_2723 PE=3 SV=1
9.	C0QV38	55025	62	C0QV38_BRAHW Carboxyl-terminal protease OS=Brachyspira hyodysenteriae (strain ATCC 49526 / WA1) GN=BHWA1_01876
10.	G0EQY1	55006	62	G0EQY1_BRAIP Carboxyl-terminal protease OS=Brachyspira intermedia (strain ATCC 51140 / PWS/A) GN=Bint_2833 PE=
11.	D5UA53	54925	62	D5UA53_BRAM5 Carboxyl-terminal protease OS=Brachyspira murdochii (strain ATCC 51284 / DSM 12563 / 56-150) GN=B
12.	L8XSF3	55005	62	L8XSF3_9SPIR Carboxyl-terminal protease OS=Brachyspira hampsonii 30599 GN=H263_08294 PE=3 SV=1
13.	L0X5I9	54934	62	L0X5I9_9SPIR Carboxyl-terminal protease OS=Brachyspira hampsonii 30446 GN=A966_01506 PE=3 SV=1
14.	L8XRS6	11096	36	L8XRS6_9SPIR GntR family transcriptional regulator (Fragment) OS=Brachyspira hampsonii 30599 GN=H263_12574 PE=

Results List

1. J9UL67 **Mass:** 54900 **Score:** 176 **Expect:** 1e-13 **Queries matched:** 3
J9UL67_BRAPL Carboxyl terminal protease OS=Brachyspira pilosicoli B2904 GN=B2904_orf2273 PE=3 SV=1

Observed	Mr(expt)	Mr(calc)	ppm	Start		End	Miss	Ions	Peptide
1297.5983	1296.5910	1296.6384	-36.51	205	-	215	0	22	K.MLEDSTIGYIR.I
1425.6359	1424.6286	1424.6837	-38.66	352	-	363	0	52	K.FTVAHYYTPNGR.R
1504.7391	1503.7318	1503.7933	-40.88	181	-	193	0	62	R.QGVVEPIEYTLTR.A

No match to: 1320.5353, 1955.7052

2. K0JIZ9 **Mass:** 54884 **Score:** 176 **Expect:** 1e-13 **Queries matched:** 3
K0JIZ9_BRAPL Carboxyl terminal protease OS=Brachyspira pilosicoli WesB GN=WESB_0581 PE=3 SV=1

Observed	Mr(expt)	Mr(calc)	ppm	Start		End	Miss	Ions	Peptide
1297.5983	1296.5910	1296.6384	-36.51	205	-	215	0	22	K.MLEDSTIGYIR.I
1425.6359	1424.6286	1424.6837	-38.66	352	-	363	0	52	K.FTVAHYYTPNGR.R
1504.7391	1503.7318	1503.7933	-40.88	181	-	193	0	62	R.QGVVEPIEYTLTR.A

No match to: 1320.5353, 1955.7052

3. D8IF04 **Mass:** 50717 **Score:** 138 **Expect:** 6.4e-10 **Queries matched:** 2
D8IF04_BRAP9 Carboxyl terminal protease OS=Brachyspira pilosicoli (strain ATCC BAA-1826 / 95/1000) GN=BP951000_1746 PE=3 SV=1

Observed	Mr(expt)	Mr(calc)	ppm	Start		End	Miss	Ions	Peptide
1425.6359	1424.6286	1424.6837	-38.66	316	-	327	0	52	K.FTVAHYYTPNGR.R
1504.7391	1503.7318	1503.7933	-40.88	145	-	157	0	62	R.QGVVEPIEYTLTR.A

No match to: 1297.5983, 1320.5353, 1955.7052

4. R5LDF9 **Mass:** 54957 **Score:** 62 **Expect:** 0.024 **Queries matched:** 1
R5LDF9_9SPIR Carboxyl-terminal protease OS=Brachyspira sp. CAG:700 GN=BN758_01320 PE=3 SV=1

Observed	Mr(expt)	Mr(calc)	ppm	Start	End	Miss	Ions	Peptide

```
     1425.6359  1424.6286  1424.6837  -38.66   351 - 362   0    52   K.FTVAHYYTPNGR.R
     No match to: 1297.5983, 1320.5353, 1504.7391, 1955.7052

5.   A0A0H0U272    Mass: 54982    Score: 62     Expect: 0.024  Queries matched: 1
     A0A0H0U272_BRAHO Carboxyl-terminal protease OS=Brachyspira hyodysenteriae GN=SZ44_03720 PE=3 SV=1
      Observed    Mr(expt)   Mr(calc)     ppm  Start    End Miss Ions  Peptide
     1425.6359  1424.6286  1424.6837  -38.66   351 - 362   0    52   K.FTVAHYYTPNGR.R
     No match to: 1297.5983, 1320.5353, 1504.7391, 1955.7052

6.   A0A0H0V600    Mass: 54998    Score: 62     Expect: 0.024  Queries matched: 1
     A0A0H0V600_BRAHO Carboxyl-terminal protease OS=Brachyspira hyodysenteriae GN=SR30_12295 PE=3 SV=1
      Observed    Mr(expt)   Mr(calc)     ppm  Start    End Miss Ions  Peptide
     1425.6359  1424.6286  1424.6837  -38.66   351 - 362   0    52   K.FTVAHYYTPNGR.R
     No match to: 1297.5983, 1320.5353, 1504.7391, 1955.7052

7.   A0A0H0X0I2    Mass: 55025    Score: 62     Expect: 0.024  Queries matched: 1
     A0A0H0X0I2_BRAHO Carboxyl-terminal protease OS=Brachyspira hyodysenteriae GN=SU45_00880 PE=3 SV=1
      Observed    Mr(expt)   Mr(calc)     ppm  Start    End Miss Ions  Peptide
     1425.6359  1424.6286  1424.6837  -38.66   351 - 362   0    52   K.FTVAHYYTPNGR.R
     No match to: 1297.5983, 1320.5353, 1504.7391, 1955.7052

8.   A0A0G4KAT6    Mass: 54978    Score: 62     Expect: 0.024  Queries matched: 1
     A0A0G4KAT6_9SPIR Carboxyl-terminal protease OS=Brachyspira suanatina GN=BRSU_2723 PE=3 SV=1
      Observed    Mr(expt)   Mr(calc)     ppm  Start    End Miss Ions  Peptide
     1425.6359  1424.6286  1424.6837  -38.66   351 - 362   0    52   K.FTVAHYYTPNGR.R
     No match to: 1297.5983, 1320.5353, 1504.7391, 1955.7052

9.   C0QV38    Mass: 55025    Score: 62     Expect: 0.024  Queries matched: 1
     C0QV38_BRAHW Carboxyl-terminal protease OS=Brachyspira hyodysenteriae (strain ATCC 49526 / WA1) GN=BHWA1_01876 PE=3 SV=1
      Observed    Mr(expt)   Mr(calc)     ppm  Start    End Miss Ions  Peptide
     1425.6359  1424.6286  1424.6837  -38.66   351 - 362   0    52   K.FTVAHYYTPNGR.R
     No match to: 1297.5983, 1320.5353, 1504.7391, 1955.7052

10.  G0EQY1    Mass: 55006    Score: 62     Expect: 0.024  Queries matched: 1
     G0EQY1_BRAIP Carboxyl-terminal protease OS=Brachyspira intermedia (strain ATCC 51140 / PWS/A) GN=Bint_2833 PE=3 SV=1
      Observed    Mr(expt)   Mr(calc)     ppm  Start    End Miss Ions  Peptide
     1425.6359  1424.6286  1424.6837  -38.66   351 - 362   0    52   K.FTVAHYYTPNGR.R
     No match to: 1297.5983, 1320.5353, 1504.7391, 1955.7052

11.  D5UA53    Mass: 54925    Score: 62     Expect: 0.024  Queries matched: 1
     D5UA53_BRAM5 Carboxyl-terminal protease OS=Brachyspira murdochii (strain ATCC 51284 / DSM 12563 / 56-150) GN=Bmur_1489 PE=3 SV=1
      Observed    Mr(expt)   Mr(calc)     ppm  Start    End Miss Ions  Peptide
     1425.6359  1424.6286  1424.6837  -38.66   351 - 362   0    52   K.FTVAHYYTPNGR.R
     No match to: 1297.5983, 1320.5353, 1504.7391, 1955.7052

12.  L8XSF3    Mass: 55005    Score: 62     Expect: 0.024  Queries matched: 1
     L8XSF3_9SPIR Carboxyl-terminal protease OS=Brachyspira hampsonii 30599 GN=H263_08294 PE=3 SV=1
      Observed    Mr(expt)   Mr(calc)     ppm  Start    End Miss Ions  Peptide
     1425.6359  1424.6286  1424.6837  -38.66   351 - 362   0    52   K.FTVAHYYTPNGR.R
     No match to: 1297.5983, 1320.5353, 1504.7391, 1955.7052

13.  L0X5I9    Mass: 54934    Score: 62     Expect: 0.024  Queries matched: 1
     L0X5I9_9SPIR Carboxyl-terminal protease OS=Brachyspira hampsonii 30446 GN=A966_01506 PE=3 SV=1
      Observed    Mr(expt)   Mr(calc)     ppm  Start    End Miss Ions  Peptide
     1425.6359  1424.6286  1424.6837  -38.66   351 - 362   0    52   K.FTVAHYYTPNGR.R
     No match to: 1297.5983, 1320.5353, 1504.7391, 1955.7052

14.  L8XRS6    Mass: 11096    Score: 36     Expect: 9.1  Queries matched: 2
     L8XRS6_9SPIR GntR family transcriptional regulator (Fragment) OS=Brachyspira hampsonii 30599 GN=H263_12574 PE=4 SV=1
      Observed    Mr(expt)   Mr(calc)     ppm  Start   End Miss Ions  Peptide
     1297.5983  1296.5910  1296.5326    45.1     7 -  16   0   ---   K.EIDMNMQEQK.I + 2 Oxidation (M)
     1425.6359  1424.6286  1424.6275    0.77     6 -  16   1   ---   K.KEIDMNMQEQK.I + 2 Oxidation (M)
     No match to: 1320.5353, 1504.7391, 1955.7052
```

Search Parameters

```
Type of search         : Sequence Query
Enzyme                 : Trypsin
Fixed modifications    : Carbamidomethyl (C)
Variable modifications : Oxidation (M)
Mass values            : Monoisotopic
Protein Mass           : Unrestricted
Peptide Mass Tolerance : ± 50 ppm
Fragment Mass Tolerance: ± 0.5 Da
Max Missed Cleavages   : 1
Instrument type        : MALDI-TOF-TOF
Query1 (1297.5983,1+)  : Locus:14.1.1.211149.56584
Query2 (1320.5353,1+)  : Locus:14.1.1.211149.56583
Query3 (1425.6359,1+)  : Locus:14.1.1.211149.56582
Query4 (1504.7391,1+)  : Locus:14.1.1.211149.56581
Query5 (1955.7052,1+)  : Locus:14.1.1.211149.56580
```

Mascot: http://www.matrixscience.com/

Mascot Search Results

```
User          :
Email         :
Search title  : F:\ROD_inmunoproteoma\MALDI\ppw_2016_562_563_ROD\ppw_H7_145397048811.txt
MS data file  : F:\ROD_inmunoproteoma\MALDI\ppw_2016_562_563_ROD\ppw_H7_145397048811.txt
Database      : Brachyspiraceae Brachyspiraceae_20160127-151122 (40573 sequences; 13470793 residues)
Timestamp     : 23 Feb 2016 at 16:05:31 GMT
Warning       : A Peptide summary report will usually give a much clearer picture of MS/MS search results.
Top Score     : 199 for D8IBH9, D8IBH9_BRAP9 ATP synthase subunit beta OS=Brachyspira pilosicoli (strain ATCC BAA-1826 / 95/1000) GN=atpB
```

Probability Based Mowse Score

Protein score is -10*Log(P), where P is the probability that the observed match is a random event.
Protein scores greater than 59 are significant (p<0.05).
Protein scores are derived from ions scores as a non-probabilistic basis for ranking protein hits.

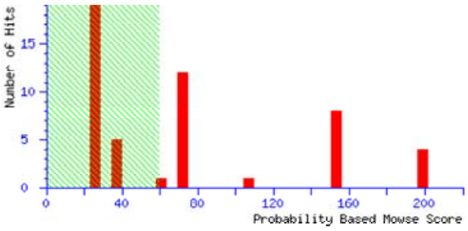

Protein Summary Report

Format As | Protein Summary (deprecated) | Help

Significance threshold p< 0.05 | Max. number of hits AUTO

Standard scoring ◉ MudPIT scoring ○ | Ions score or expect cut-off 0 | Show sub-sets 0

Show pop-ups ◉ Suppress pop-ups ○ | Sort unassigned Decreasing Score | Require bold red ☐

Re-Search All | Search Unmatched

Index

	Accession	Mass	Score	Description
1.	D8IBH9	45558	199	D8IBH9_BRAP9 ATP synthase subunit beta OS=Brachyspira pilosicoli (strain ATCC BAA-1826 / 95/1000) GN=atpB PE=3
2.	J9TU52	51489	197	J9TU52_BRAPL ATP synthase subunit beta OS=Brachyspira pilosicoli B2904 GN=atpB PE=3 SV=1
3.	K0JGH7	51489	197	K0JGH7_BRAPL ATP synthase subunit beta OS=Brachyspira pilosicoli WesB GN=atpB PE=3 SV=1
4.	D5U6G9	51572	197	D5U6G9_BRAM5 ATP synthase subunit beta OS=Brachyspira murdochii (strain ATCC 51284 / DSM 12563 / 56-150) GN=at
5.	A0A0H0W5X7	51422	152	A0A0H0W5X7_BRAHO ATP synthase subunit beta OS=Brachyspira hyodysenteriae GN=atpD PE=3 SV=1
6.	A0A0G4K5M1	51396	152	A0A0G4K5M1_9SPIR ATP synthase subunit beta OS=Brachyspira suanatina GN=atpD PE=3 SV=1
7.	C0QX45	51422	152	C0QX45_BRAHW ATP synthase subunit beta OS=Brachyspira hyodysenteriae (strain ATCC 49526 / WA1) GN=atpB PE=3 SV
8.	A0A0H0UVK0	51408	152	A0A0H0UVK0_BRAHO ATP synthase subunit beta OS=Brachyspira hyodysenteriae GN=atpD PE=3 SV=1
9.	A0A0H0VYZ1	51448	152	A0A0H0VYZ1_BRAHO ATP synthase subunit beta OS=Brachyspira hyodysenteriae GN=atpD PE=3 SV=1
10.	G0EIV5	51425	152	G0EIV5_BRAIP ATP synthase subunit beta OS=Brachyspira intermedia (strain ATCC 51140 / PWS/A) GN=atpD PE=3 SV=1
11.	L0X001	51460	152	L0X001_9SPIR ATP synthase subunit beta OS=Brachyspira hampsonii 30446 GN=atpD PE=3 SV=1
12.	L8XRE7	53590	152	L8XRE7_9SPIR ATP synthase subunit beta OS=Brachyspira hampsonii 30599 GN=atpD PE=3 SV=1
13.	R5LYW4	51867	107	R5LYW4_9SPIR ATP synthase subunit beta OS=Brachyspira sp. CAG:700 GN=atpD PE=3 SV=1
14.	C0QW65	54629	69	ATPA_BRAHW ATP synthase subunit alpha OS=Brachyspira hyodysenteriae (strain ATCC 49526 / WA1) GN=atpA PE=3 SV=
15.	A0A0H0TMK8	54643	69	A0A0H0TMK8_BRAHO ATP synthase subunit alpha OS=Brachyspira hyodysenteriae GN=atpA PE=3 SV=1
16.	A0A0G4K3V0	54626	69	A0A0G4K3V0_9SPIR ATP synthase subunit alpha OS=Brachyspira suanatina GN=atpA PE=3 SV=1
17.	R5L6E9	55169	69	R5L6E9_9SPIR ATP synthase subunit alpha OS=Brachyspira sp. CAG:700 GN=atpA PE=3 SV=1
18.	A0A0H0VHV0	54659	69	A0A0H0VHV0_BRAHO ATP synthase subunit alpha OS=Brachyspira hyodysenteriae GN=atpA PE=3 SV=1
19.	G0ENC9	54685	69	G0ENC9_BRAIP ATP synthase subunit alpha OS=Brachyspira intermedia (strain ATCC 51140 / PWS/A) GN=atpA PE=3 SV=
20.	J9UJ83	54730	69	J9UJ83_BRAPL ATP synthase subunit alpha OS=Brachyspira pilosicoli B2904 GN=atpA PE=3 SV=1
21.	D8IBP7	54730	69	D8IBP7_BRAP9 ATP synthase subunit alpha OS=Brachyspira pilosicoli (strain ATCC BAA-1826 / 95/1000) GN=atpA PE=
22.	K0JLM2	54730	69	K0JLM2_BRAPL ATP synthase subunit alpha OS=Brachyspira pilosicoli WesB GN=atpA1 PE=3 SV=1
23.	D5U3H4	54409	69	D5U3H4_BRAM5 ATP synthase subunit alpha OS=Brachyspira murdochii (strain ATCC 51284 / DSM 12563 / 56-150) GN=a
24.	L8XPW2	54698	69	L8XPW2_9SPIR ATP synthase subunit alpha OS=Brachyspira hampsonii 30599 GN=atpA PE=3 SV=1
25.	L0X262	54629	69	L0X262_9SPIR ATP synthase subunit alpha OS=Brachyspira hampsonii 30446 GN=atpA PE=3 SV=1
26.	R5H8C6	51497	65	R5H8C6_9SPIR ATP synthase subunit beta OS=Brachyspira sp. CAG:484 GN=atpD PE=3 SV=1
27.	J9U9U5	10944	37	J9U9U5_BRAPL HTH_3 domain containing protein OS=Brachyspira pilosicoli B2904 GN=B2904_orf159 PE=4 SV=1

Results List

1. D8IBH9 **Mass:** 45558 **Score:** 199 **Expect:** 5.1e-16 **Queries matched:** 3
D8IBH9_BRAP9 ATP synthase subunit beta OS=Brachyspira pilosicoli (strain ATCC BAA-1826 / 95/1000) GN=atpB PE=3 SV=1

Observed	Mr(expt)	Mr(calc)	ppm	Start	End	Miss	Ions	Peptide
1435.6978	1434.6905	1434.7467	-39.15	203 -	216	0	54	R.FTQAGSEVSALLGR.M
1485.7166	1484.7093	1484.7697	-40.67	174 -	186	0	29	R.VALTALTMAEYFR.D
1855.7207	1854.7134	1854.7843	-38.20	385 -	400	0	73	K.GICNGDYDDLPDQAFR.L

No match to: 1401.6362, 1537.6815

2. J9TU52 **Mass:** 51489 **Score:** 197 **Expect:** 8.1e-16 **Queries matched:** 3
J9TU52_BRAPL ATP synthase subunit beta OS=Brachyspira pilosicoli B2904 GN=atpB PE=3 SV=1

Observed	Mr(expt)	Mr(calc)	ppm	Start	End	Miss	Ions	Peptide
1435.6978	1434.6905	1434.7467	-39.15	256 -	269	0	54	R.FTQAGSEVSALLGR.M
1485.7166	1484.7093	1484.7697	-40.67	227 -	239	0	29	R.VALTALTMAEYFR.D
1855.7207	1854.7134	1854.7843	-38.20	438 -	453	0	73	K.GICNGDYDDLPDQAFR.L

No match to: 1401.6362, 1537.6815

3. K0JGH7 **Mass:** 51489 **Score:** 197 **Expect:** 8.1e-16 **Queries matched:** 3
K0JGH7_BRAPL ATP synthase subunit beta OS=Brachyspira pilosicoli WesB GN=atpB PE=3 SV=1

Observed	Mr(expt)	Mr(calc)	ppm	Start		End	Miss	Ions	Peptide
1435.6978	1434.6905	1434.7467	-39.15	256	-	269	0	54	R.FTQAGSEVSALLGR.M
1485.7166	1484.7093	1484.7697	-40.67	227	-	239	0	29	R.VALTALTMAEYFR.D
1855.7207	1854.7134	1854.7843	-38.20	438	-	453	0	73	K.GICNGDYDDLPDQAFR.L

No match to: 1401.6362, 1537.6815

4. D5U6G9 **Mass:** 51572 **Score:** 197 **Expect:** 8.1e-16 **Queries matched:** 3
D5U6G9_BRAM5 ATP synthase subunit beta OS=Brachyspira murdochii (strain ATCC 51284 / DSM 12563 / 56-150) GN=atpD PE=3 SV=1

Observed	Mr(expt)	Mr(calc)	ppm	Start		End	Miss	Ions	Peptide
1435.6978	1434.6905	1434.7467	-39.15	256	-	269	0	54	R.FTQAGSEVSALLGR.M
1485.7166	1484.7093	1484.7697	-40.67	227	-	239	0	29	R.VALTALTMAEYFR.D
1855.7207	1854.7134	1854.7843	-38.20	438	-	453	0	73	K.GICNGDYDDLPDQAFR.F

No match to: 1401.6362, 1537.6815

5. A0A0H0W5X7 **Mass:** 51422 **Score:** 152 **Expect:** 2.6e-11 **Queries matched:** 2
A0A0H0W5X7_BRAHO ATP synthase subunit beta OS=Brachyspira hyodysenteriae GN=atpD PE=3 SV=1

Observed	Mr(expt)	Mr(calc)	ppm	Start		End	Miss	Ions	Peptide
1435.6978	1434.6905	1434.7467	-39.15	256	-	269	0	54	R.FTQAGSEVSALLGR.M
1855.7207	1854.7134	1854.7843	-38.20	438	-	453	0	73	K.GICNGDYDDLPDQAFR.F

No match to: 1401.6362, 1485.7166, 1537.6815

6. A0A0G4K5M1 **Mass:** 51396 **Score:** 152 **Expect:** 2.6e-11 **Queries matched:** 2
A0A0G4K5M1_9SPIR ATP synthase subunit beta OS=Brachyspira suanatina GN=atpD PE=3 SV=1

Observed	Mr(expt)	Mr(calc)	ppm	Start		End	Miss	Ions	Peptide
1435.6978	1434.6905	1434.7467	-39.15	256	-	269	0	54	R.FTQAGSEVSALLGR.M
1855.7207	1854.7134	1854.7843	-38.20	438	-	453	0	73	K.GICNGDYDDLPDQAFR.F

No match to: 1401.6362, 1485.7166, 1537.6815

7. C0QX45 **Mass:** 51422 **Score:** 152 **Expect:** 2.6e-11 **Queries matched:** 2
C0QX45_BRAHW ATP synthase subunit beta OS=Brachyspira hyodysenteriae (strain ATCC 49526 / WA1) GN=atpB PE=3 SV=1

Observed	Mr(expt)	Mr(calc)	ppm	Start		End	Miss	Ions	Peptide
1435.6978	1434.6905	1434.7467	-39.15	256	-	269	0	54	R.FTQAGSEVSALLGR.M
1855.7207	1854.7134	1854.7843	-38.20	438	-	453	0	73	K.GICNGDYDDLPDQAFR.F

No match to: 1401.6362, 1485.7166, 1537.6815

8. A0A0H0UVK0 **Mass:** 51408 **Score:** 152 **Expect:** 2.6e-11 **Queries matched:** 2
A0A0H0UVK0_BRAHO ATP synthase subunit beta OS=Brachyspira hyodysenteriae GN=atpD PE=3 SV=1

Observed	Mr(expt)	Mr(calc)	ppm	Start		End	Miss	Ions	Peptide
1435.6978	1434.6905	1434.7467	-39.15	256	-	269	0	54	R.FTQAGSEVSALLGR.M
1855.7207	1854.7134	1854.7843	-38.20	438	-	453	0	73	K.GICNGDYDDLPDQAFR.F

No match to: 1401.6362, 1485.7166, 1537.6815

9. A0A0H0VYZ1 **Mass:** 51448 **Score:** 152 **Expect:** 2.6e-11 **Queries matched:** 2
A0A0H0VYZ1_BRAHO ATP synthase subunit beta OS=Brachyspira hyodysenteriae GN=atpD PE=3 SV=1

Observed	Mr(expt)	Mr(calc)	ppm	Start		End	Miss	Ions	Peptide
1435.6978	1434.6905	1434.7467	-39.15	256	-	269	0	54	R.FTQAGSEVSALLGR.M
1855.7207	1854.7134	1854.7843	-38.20	438	-	453	0	73	K.GICNGDYDDLPDQAFR.F

No match to: 1401.6362, 1485.7166, 1537.6815

10. G0EIV5 **Mass:** 51425 **Score:** 152 **Expect:** 2.6e-11 **Queries matched:** 2
G0EIV5_BRAIP ATP synthase subunit beta OS=Brachyspira intermedia (strain ATCC 51140 / PWS/A) GN=atpD PE=3 SV=1

Observed	Mr(expt)	Mr(calc)	ppm	Start		End	Miss	Ions	Peptide
1435.6978	1434.6905	1434.7467	-39.15	256	-	269	0	54	R.FTQAGSEVSALLGR.M
1855.7207	1854.7134	1854.7843	-38.20	438	-	453	0	73	K.GICNGDYDDLPDQAFR.F

No match to: 1401.6362, 1485.7166, 1537.6815

11. L0X001 **Mass:** 51460 **Score:** 152 **Expect:** 2.6e-11 **Queries matched:** 2
L0X001_9SPIR ATP synthase subunit beta OS=Brachyspira hampsonii 30446 GN=atpD PE=3 SV=1

Observed	Mr(expt)	Mr(calc)	ppm	Start		End	Miss	Ions	Peptide
1435.6978	1434.6905	1434.7467	-39.15	256	-	269	0	54	R.FTQAGSEVSALLGR.M
1855.7207	1854.7134	1854.7843	-38.20	438	-	453	0	73	K.GICNGDYDDLPDQAFR.F

No match to: 1401.6362, 1485.7166, 1537.6815

12. L8XRE7 **Mass:** 53590 **Score:** 152 **Expect:** 2.6e-11 **Queries matched:** 2
L8XRE7_9SPIR ATP synthase subunit beta OS=Brachyspira hampsonii 30599 GN=atpD PE=3 SV=1

Observed	Mr(expt)	Mr(calc)	ppm	Start		End	Miss	Ions	Peptide
1435.6978	1434.6905	1434.7467	-39.15	273	-	286	0	54	R.FTQAGSEVSALLGR.M
1855.7207	1854.7134	1854.7843	-38.20	455	-	470	0	73	K.GICNGDYDDLPDQAFR.F

No match to: 1401.6362, 1485.7166, 1537.6815

13. R5LYW4 **Mass:** 51867 **Score:** 107 **Expect:** 8.1e-07 **Queries matched:** 2
R5LYW4_9SPIR ATP synthase subunit beta OS=Brachyspira sp. CAG:700 GN=atpD PE=3 SV=1

Observed	Mr(expt)	Mr(calc)	ppm	Start		End	Miss	Ions	Peptide
1435.6978	1434.6905	1434.7467	-39.15	259	-	272	0	54	R.FTQAGSEVSALLGR.M
1485.7166	1484.7093	1484.7697	-40.67	230	-	242	0	29	R.VALTALTMAEYFR.D

No match to: 1401.6362, 1537.6815, 1855.7207

14. C0QW65 **Mass:** 54629 **Score:** 69 **Expect:** 0.0053 **Queries matched:** 1
ATPA_BRAHW ATP synthase subunit alpha OS=Brachyspira hyodysenteriae (strain ATCC 49526 / WA1) GN=atpA PE=3 SV=1

Observed	Mr(expt)	Mr(calc)	ppm	Start		End	Miss	Ions	Peptide
1537.6815	1536.6742	1536.7361	-40.28	285	-	297	0	59	R.EAFPGDVFYLHSR.L

No match to: 1401.6362, 1435.6978, 1485.7166, 1855.7207

```
15.   A0A0H0TMK8    Mass: 54643    Score: 69     Expect: 0.0053  Queries matched: 1
      A0A0H0TMK8_BRAHO ATP synthase subunit alpha OS=Brachyspira hyodysenteriae GN=atpA PE=3 SV=1
       Observed    Mr(expt)    Mr(calc)     ppm  Start   End Miss Ions  Peptide
      1537.6815  1536.6742  1536.7361  -40.28   285 - 297   0   59   R.EAFPGDVFYLHSR.L
       No match to: 1401.6362, 1435.6978, 1485.7166, 1855.7207

16.   A0A0G4K3V0    Mass: 54626    Score: 69     Expect: 0.0053  Queries matched: 1
      A0A0G4K3V0_9SPIR ATP synthase subunit alpha OS=Brachyspira suanatina GN=atpA PE=3 SV=1
       Observed    Mr(expt)    Mr(calc)     ppm  Start   End Miss Ions  Peptide
      1537.6815  1536.6742  1536.7361  -40.28   285 - 297   0   59   R.EAFPGDVFYLHSR.L
       No match to: 1401.6362, 1435.6978, 1485.7166, 1855.7207

17.   R5L6E9    Mass: 55169    Score: 69     Expect: 0.0053  Queries matched: 1
      R5L6E9_9SPIR ATP synthase subunit alpha OS=Brachyspira sp. CAG:700 GN=atpA PE=3 SV=1
       Observed    Mr(expt)    Mr(calc)     ppm  Start   End Miss Ions  Peptide
      1537.6815  1536.6742  1536.7361  -40.28   285 - 297   0   59   R.EAFPGDVFYLHSR.L
       No match to: 1401.6362, 1435.6978, 1485.7166, 1855.7207

18.   A0A0H0VHV0    Mass: 54659    Score: 69     Expect: 0.0053  Queries matched: 1
      A0A0H0VHV0_BRAHO ATP synthase subunit alpha OS=Brachyspira hyodysenteriae GN=atpA PE=3 SV=1
       Observed    Mr(expt)    Mr(calc)     ppm  Start   End Miss Ions  Peptide
      1537.6815  1536.6742  1536.7361  -40.28   285 - 297   0   59   R.EAFPGDVFYLHSR.L
       No match to: 1401.6362, 1435.6978, 1485.7166, 1855.7207

19.   G0ENC9    Mass: 54685    Score: 69     Expect: 0.0053  Queries matched: 1
      G0ENC9_BRAIP ATP synthase subunit alpha OS=Brachyspira intermedia (strain ATCC 51140 / PWS/A) GN=atpA PE=3 SV=1
       Observed    Mr(expt)    Mr(calc)     ppm  Start   End Miss Ions  Peptide
      1537.6815  1536.6742  1536.7361  -40.28   285 - 297   0   59   R.EAFPGDVFYLHSR.L
       No match to: 1401.6362, 1435.6978, 1485.7166, 1855.7207

20.   J9UJ83    Mass: 54730    Score: 69     Expect: 0.0053  Queries matched: 1
      J9UJ83_BRAPL ATP synthase subunit alpha OS=Brachyspira pilosicoli B2904 GN=atpA PE=3 SV=1
       Observed    Mr(expt)    Mr(calc)     ppm  Start   End Miss Ions  Peptide
      1537.6815  1536.6742  1536.7361  -40.28   285 - 297   0   59   R.EAFPGDVFYLHSR.L
       No match to: 1401.6362, 1435.6978, 1485.7166, 1855.7207

21.   D8IBP7    Mass: 54730    Score: 69     Expect: 0.0053  Queries matched: 1
      D8IBP7_BRAP9 ATP synthase subunit alpha OS=Brachyspira pilosicoli (strain ATCC BAA-1826 / 95/1000) GN=atpA PE=3 SV=1
       Observed    Mr(expt)    Mr(calc)     ppm  Start   End Miss Ions  Peptide
      1537.6815  1536.6742  1536.7361  -40.28   285 - 297   0   59   R.EAFPGDVFYLHSR.L
       No match to: 1401.6362, 1435.6978, 1485.7166, 1855.7207

22.   K0JLM2    Mass: 54730    Score: 69     Expect: 0.0053  Queries matched: 1
      K0JLM2_BRAPL ATP synthase subunit alpha OS=Brachyspira pilosicoli WesB GN=atpA1 PE=3 SV=1
       Observed    Mr(expt)    Mr(calc)     ppm  Start   End Miss Ions  Peptide
      1537.6815  1536.6742  1536.7361  -40.28   285 - 297   0   59   R.EAFPGDVFYLHSR.L
       No match to: 1401.6362, 1435.6978, 1485.7166, 1855.7207

23.   D5U3H4    Mass: 54409    Score: 69     Expect: 0.0053  Queries matched: 1
      D5U3H4_BRAM5 ATP synthase subunit alpha OS=Brachyspira murdochii (strain ATCC 51284 / DSM 12563 / 56-150) GN=atpA PE=3 SV=1
       Observed    Mr(expt)    Mr(calc)     ppm  Start   End Miss Ions  Peptide
      1537.6815  1536.6742  1536.7361  -40.28   285 - 297   0   59   R.EAFPGDVFYLHSR.L
       No match to: 1401.6362, 1435.6978, 1485.7166, 1855.7207

24.   L8XPW2    Mass: 54698    Score: 69     Expect: 0.0053  Queries matched: 1
      L8XPW2_9SPIR ATP synthase subunit alpha OS=Brachyspira hampsonii 30599 GN=atpA PE=3 SV=1
       Observed    Mr(expt)    Mr(calc)     ppm  Start   End Miss Ions  Peptide
      1537.6815  1536.6742  1536.7361  -40.28   285 - 297   0   59   R.EAFPGDVFYLHSR.L
       No match to: 1401.6362, 1435.6978, 1485.7166, 1855.7207

25.   L0X262    Mass: 54629    Score: 69     Expect: 0.0053  Queries matched: 1
      L0X262_9SPIR ATP synthase subunit alpha OS=Brachyspira hampsonii 30446 GN=atpA PE=3 SV=1
       Observed    Mr(expt)    Mr(calc)     ppm  Start   End Miss Ions  Peptide
      1537.6815  1536.6742  1536.7361  -40.28   285 - 297   0   59   R.EAFPGDVFYLHSR.L
       No match to: 1401.6362, 1435.6978, 1485.7166, 1855.7207

26.   R5H8C6    Mass: 51497    Score: 65     Expect: 0.013  Queries matched: 1
      R5H8C6_9SPIR ATP synthase subunit beta OS=Brachyspira sp. CAG:484 GN=atpD PE=3 SV=1
       Observed    Mr(expt)    Mr(calc)     ppm  Start   End Miss Ions  Peptide
      1435.6978  1434.6905  1434.7467  -39.15   255 - 268   0   54   R.FTQAGSEVSALLGR.V
       No match to: 1401.6362, 1485.7166, 1537.6815, 1855.7207

27.   J9U9U5    Mass: 10944    Score: 37     Expect: 7.7  Queries matched: 2
      J9U9U5_BRAPL HTH_3 domain containing protein OS=Brachyspira pilosicoli B2904 GN=B2904_orf159 PE=4 SV=1
       Observed    Mr(expt)    Mr(calc)     ppm  Start   End Miss Ions  Peptide
      1401.6362  1400.6289  1400.6540  -17.93     4 -  14   1  ---   K.MLGMTKQQYNR.Y + 2 Oxidation (M)
      1537.6815  1536.6742  1536.7501  -49.36    52 -  64   1  ---   K.SIQTRCMSVLGNR.N + Oxidation (M)
       No match to: 1435.6978, 1485.7166, 1855.7207
```

Search Parameters

```
Type of search         : Sequence Query
Enzyme                 : Trypsin
Fixed modifications    : Carbamidomethyl (C)
Variable modifications : Oxidation (M)
Mass values            : Monoisotopic
Protein Mass           : Unrestricted
```

```
Peptide Mass Tolerance : ± 50 ppm
Fragment Mass Tolerance: ± 0.5 Da
Max Missed Cleavages   : 1
Instrument type        : MALDI-TOF-TOF
Query1 (1401.6362,1+)  : Locus:12.1.1.211147.56570
Query2 (1435.6978,1+)  : Locus:12.1.1.211147.56571
Query3 (1485.7166,1+)  : Locus:12.1.1.211147.56574
Query4 (1537.6815,1+)  : Locus:12.1.1.211147.56573
Query5 (1855.7207,1+)  : Locus:12.1.1.211147.56572
```

**Mascot:** http://www.matrixscience.com/

Mascot Search Results

User :
Email :
Search title : F:\ROD_inmunoproteoma\MALDI\160223_2016_561_562_ROD\ppw_G8_145631468315.txt
MS data file : F:\ROD_inmunoproteoma\MALDI\160223_2016_561_562_ROD\ppw_G8_145631468315.txt
Database : Brachyspiraceae Brachyspiraceae_20160127-151122 (40573 sequences; 13470793 residues)
Timestamp : 24 Feb 2016 at 12:03:00 GMT
Warning : A Peptide summary report will usually give a much clearer picture of MS/MS search results.
Top Score : 328 for A0A0H0W3D6, A0A0H0W3D6_BRAHO Enolase OS=Brachyspira hyodysenteriae GN=eno PE=3 SV=1

Probability Based Mowse Score

Protein score is -10*Log(P), where P is the probability that the observed match is a random event.
Protein scores greater than 59 are significant (p<0.05).
Protein scores are derived from ions scores as a non-probabilistic basis for ranking protein hits.

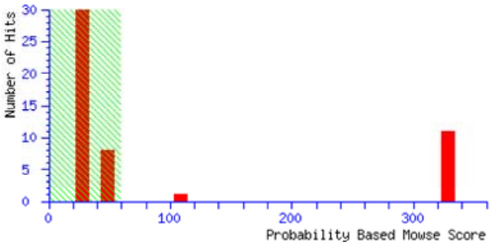

Protein Summary Report

Format As | Protein Summary (deprecated) | Help

Significance threshold p< 0.05 Max. number of hits AUTO

Standard scoring ◉ MudPIT scoring ○ Ions score or expect cut-off 0 Show sub-sets 0

Show pop-ups ◉ Suppress pop-ups ○ Sort unassigned Decreasing Score Require bold red ☐

Re-Search All | Search Unmatched

Index

	Accession	Mass	Score	Description
1.	A0A0H0W3D6	47082	328	A0A0H0W3D6_BRAHO Enolase OS=Brachyspira hyodysenteriae GN=eno PE=3 SV=1
2.	A0A0G4KAH6	47151	328	A0A0G4KAH6_9SPIR Enolase OS=Brachyspira suanatina GN=eno PE=3 SV=1
3.	C0QV52	47082	328	C0QV52_BRAHW Enolase OS=Brachyspira hyodysenteriae (strain ATCC 49526 / WA1) GN=eno PE=3 SV=1
4.	A0A0H0XEZ9	47112	328	A0A0H0XEZ9_BRAHO Enolase OS=Brachyspira hyodysenteriae GN=eno PE=3 SV=1
5.	G0EQW4	47138	328	G0EQW4_BRAIP Enolase OS=Brachyspira intermedia (strain ATCC 51140 / PWS/A) GN=eno PE=3 SV=1
6.	J9URW4	47046	328	J9URW4_BRAPL Enolase OS=Brachyspira pilosicoli B2904 GN=eno PE=3 SV=1
7.	D8IET2	47046	328	D8IET2_BRAP9 Enolase OS=Brachyspira pilosicoli (strain ATCC BAA-1826 / 95/1000) GN=eno PE=3 SV=1
8.	K0JI69	47046	328	K0JI69_BRAPL Enolase OS=Brachyspira pilosicoli WesB GN=eno PE=3 SV=1
9.	D5U9W9	47170	328	D5U9W9_BRAM5 Enolase OS=Brachyspira murdochii (strain ATCC 51284 / DSM 12563 / 56-150) GN=eno PE=3 SV=1
10.	L8XSM5	47036	328	L8XSM5_9SPIR Enolase OS=Brachyspira hampsonii 30599 GN=eno PE=3 SV=1
11.	L0X4B5	46993	328	L0X4B5_9SPIR Enolase OS=Brachyspira hampsonii 30446 GN=eno PE=3 SV=1
12.	R5LML6	47006	98	R5LML6_9SPIR Enolase OS=Brachyspira sp. CAG:700 GN=eno PE=3 SV=1
13.	L8XSP5	37789	44	L8XSP5_9SPIR Uncharacterized protein OS=Brachyspira hampsonii 30599 GN=H263_07780 PE=4 SV=1

Results List

1. A0A0H0W3D6 **Mass:** 47082 **Score:** 328 **Expect:** 6.4e-29 **Queries matched:** 4
A0A0H0W3D6_BRAHO Enolase OS=Brachyspira hyodysenteriae GN=eno PE=3 SV=1

Observed	Mr(expt)	Mr(calc)	ppm	Start	End	Miss	Ions	Peptide
1219.6710	1218.6637	1218.6397	19.7	181 -	190	0	64	R.YVAEVFHNLK.A
1284.6805	1283.6732	1283.6371	28.2	359 -	369	0	52	K.THNYTAVVSHR.S
1320.7004	1319.6931	1319.6543	29.4	121 -	132	0	63	K.AAAEELGMPLYR.Y
1702.9937	1701.9864	1701.9414	26.5	310 -	324	1	87	K.VQLVGDDLFVTNVKR.L

No match to: 1334.7179

2. A0A0G4KAH6 **Mass:** 47151 **Score:** 328 **Expect:** 6.4e-29 **Queries matched:** 4
A0A0G4KAH6_9SPIR Enolase OS=Brachyspira suanatina GN=eno PE=3 SV=1

Observed	Mr(expt)	Mr(calc)	ppm	Start	End	Miss	Ions	Peptide
1219.6710	1218.6637	1218.6397	19.7	181 -	190	0	64	R.YVAEVFHNLK.A
1284.6805	1283.6732	1283.6371	28.2	359 -	369	0	52	K.THNYTAVVSHR.S
1320.7004	1319.6931	1319.6543	29.4	121 -	132	0	63	K.AAAEELGMPLYR.Y
1702.9937	1701.9864	1701.9414	26.5	310 -	324	1	87	K.VQLVGDDLFVTNVKR.L

No match to: 1334.7179

3. C0QV52 **Mass:** 47082 **Score:** 328 **Expect:** 6.4e-29 **Queries matched:** 4
C0QV52_BRAHW Enolase OS=Brachyspira hyodysenteriae (strain ATCC 49526 / WA1) GN=eno PE=3 SV=1

Observed	Mr(expt)	Mr(calc)	ppm	Start	End	Miss	Ions	Peptide
1219.6710	1218.6637	1218.6397	19.7	181 -	190	0	64	R.YVAEVFHNLK.A

1284.6805	1283.6732	1283.6371	28.2	359 - 369	0	52	K.THNYTAVVSHR.S
1320.7004	1319.6931	1319.6543	29.4	121 - 132	0	63	K.AAAEELGMPLYR.Y
1702.9937	1701.9864	1701.9414	26.5	310 - 324	1	87	K.VQLVGDDLFVTNVKR.L

No match to: 1334.7179

4. A0A0H0XEZ9 **Mass:** 47112 **Score:** 328 **Expect:** 6.4e-29 **Queries matched:** 4
A0A0H0XEZ9_BRAHO Enolase OS=Brachyspira hyodysenteriae GN=eno PE=3 SV=1

Observed	Mr(expt)	Mr(calc)	ppm	Start - End	Miss	Ions	Peptide
1219.6710	1218.6637	1218.6397	19.7	181 - 190	0	64	R.YVAEVFHNLK.A
1284.6805	1283.6732	1283.6371	28.2	359 - 369	0	52	K.THNYTAVVSHR.S
1320.7004	1319.6931	1319.6543	29.4	121 - 132	0	63	K.AAAEELGMPLYR.Y
1702.9937	1701.9864	1701.9414	26.5	310 - 324	1	87	K.VQLVGDDLFVTNVKR.L

No match to: 1334.7179

5. G0EQW4 **Mass:** 47138 **Score:** 328 **Expect:** 6.4e-29 **Queries matched:** 4
G0EQW4_BRAIP Enolase OS=Brachyspira intermedia (strain ATCC 51140 / PWS/A) GN=eno PE=3 SV=1

Observed	Mr(expt)	Mr(calc)	ppm	Start - End	Miss	Ions	Peptide
1219.6710	1218.6637	1218.6397	19.7	181 - 190	0	64	R.YVAEVFHNLK.A
1284.6805	1283.6732	1283.6371	28.2	359 - 369	0	52	K.THNYTAVVSHR.S
1320.7004	1319.6931	1319.6543	29.4	121 - 132	0	63	K.AAAEELGMPLYR.Y
1702.9937	1701.9864	1701.9414	26.5	310 - 324	1	87	K.VQLVGDDLFVTNVKR.L

No match to: 1334.7179

6. J9URW4 **Mass:** 47046 **Score:** 328 **Expect:** 6.4e-29 **Queries matched:** 4
J9URW4_BRAPL Enolase OS=Brachyspira pilosicoli B2904 GN=eno PE=3 SV=1

Observed	Mr(expt)	Mr(calc)	ppm	Start - End	Miss	Ions	Peptide
1219.6710	1218.6637	1218.6397	19.7	181 - 190	0	64	R.YVAEVFHNLK.A
1284.6805	1283.6732	1283.6371	28.2	359 - 369	0	52	K.THNYTAVVSHR.S
1320.7004	1319.6931	1319.6543	29.4	121 - 132	0	63	K.AAAEELGMPLYR.Y
1702.9937	1701.9864	1701.9414	26.5	310 - 324	1	87	K.VQLVGDDLFVTNVKR.L

No match to: 1334.7179

7. D8IET2 **Mass:** 47046 **Score:** 328 **Expect:** 6.4e-29 **Queries matched:** 4
D8IET2_BRAP9 Enolase OS=Brachyspira pilosicoli (strain ATCC BAA-1826 / 95/1000) GN=eno PE=3 SV=1

Observed	Mr(expt)	Mr(calc)	ppm	Start - End	Miss	Ions	Peptide
1219.6710	1218.6637	1218.6397	19.7	181 - 190	0	64	R.YVAEVFHNLK.A
1284.6805	1283.6732	1283.6371	28.2	359 - 369	0	52	K.THNYTAVVSHR.S
1320.7004	1319.6931	1319.6543	29.4	121 - 132	0	63	K.AAAEELGMPLYR.Y
1702.9937	1701.9864	1701.9414	26.5	310 - 324	1	87	K.VQLVGDDLFVTNVKR.L

No match to: 1334.7179

8. K0JI69 **Mass:** 47046 **Score:** 328 **Expect:** 6.4e-29 **Queries matched:** 4
K0JI69_BRAPL Enolase OS=Brachyspira pilosicoli WesB GN=eno PE=3 SV=1

Observed	Mr(expt)	Mr(calc)	ppm	Start - End	Miss	Ions	Peptide
1219.6710	1218.6637	1218.6397	19.7	181 - 190	0	64	R.YVAEVFHNLK.A
1284.6805	1283.6732	1283.6371	28.2	359 - 369	0	52	K.THNYTAVVSHR.S
1320.7004	1319.6931	1319.6543	29.4	121 - 132	0	63	K.AAAEELGMPLYR.Y
1702.9937	1701.9864	1701.9414	26.5	310 - 324	1	87	K.VQLVGDDLFVTNVKR.L

No match to: 1334.7179

9. D5U9W9 **Mass:** 47170 **Score:** 328 **Expect:** 6.4e-29 **Queries matched:** 4
D5U9W9_BRAM5 Enolase OS=Brachyspira murdochii (strain ATCC 51284 / DSM 12563 / 56-150) GN=eno PE=3 SV=1

Observed	Mr(expt)	Mr(calc)	ppm	Start - End	Miss	Ions	Peptide
1219.6710	1218.6637	1218.6397	19.7	181 - 190	0	64	R.YVAEVFHNLK.N
1284.6805	1283.6732	1283.6371	28.2	359 - 369	0	52	K.THNYTAVVSHR.S
1320.7004	1319.6931	1319.6543	29.4	121 - 132	0	63	K.AAAEELGMPLYR.Y
1702.9937	1701.9864	1701.9414	26.5	310 - 324	1	87	K.VQLVGDDLFVTNVKR.L

No match to: 1334.7179

10. L8XSM5 **Mass:** 47036 **Score:** 328 **Expect:** 6.4e-29 **Queries matched:** 4
L8XSM5_9SPIR Enolase OS=Brachyspira hampsonii 30599 GN=eno PE=3 SV=1

Observed	Mr(expt)	Mr(calc)	ppm	Start - End	Miss	Ions	Peptide
1219.6710	1218.6637	1218.6397	19.7	181 - 190	0	64	R.YVAEVFHNLK.N
1284.6805	1283.6732	1283.6371	28.2	359 - 369	0	52	K.THNYTAVVSHR.S
1320.7004	1319.6931	1319.6543	29.4	121 - 132	0	63	K.AAAEELGMPLYR.Y
1702.9937	1701.9864	1701.9414	26.5	310 - 324	1	87	K.VQLVGDDLFVTNVKR.L

No match to: 1334.7179

11. L0X4B5 **Mass:** 46993 **Score:** 328 **Expect:** 6.4e-29 **Queries matched:** 4
L0X4B5_9SPIR Enolase OS=Brachyspira hampsonii 30446 GN=eno PE=3 SV=1

Observed	Mr(expt)	Mr(calc)	ppm	Start - End	Miss	Ions	Peptide
1219.6710	1218.6637	1218.6397	19.7	181 - 190	0	64	R.YVAEVFHNLK.A
1284.6805	1283.6732	1283.6371	28.2	359 - 369	0	52	K.THNYTAVVSHR.S
1320.7004	1319.6931	1319.6543	29.4	121 - 132	0	63	K.AAAEELGMPLYR.Y
1702.9937	1701.9864	1701.9414	26.5	310 - 324	1	87	K.VQLVGDDLFVTNVKR.L

No match to: 1334.7179

12. R5LML6 **Mass:** 47006 **Score:** 98 **Expect:** 6e-06 **Queries matched:** 1
R5LML6_9SPIR Enolase OS=Brachyspira sp. CAG:700 GN=eno PE=3 SV=1

Observed	Mr(expt)	Mr(calc)	ppm	Start - End	Miss	Ions	Peptide
1702.9937	1701.9864	1701.9414	26.5	310 - 324	1	87	K.VQLVGDDLFVTNVKR.L

No match to: 1219.6710, 1284.6805, 1320.7004, 1334.7179

13. L8XSP5 **Mass:** 37789 **Score:** 44 **Expect:** 1.5 **Queries matched:** 2

L8XSP5_9SPIR Uncharacterized protein OS=Brachyspira hampsonii 30599 GN=H263_07780 PE=4 SV=1

Observed	Mr(expt)	Mr(calc)	ppm	Start		End	Miss	Ions	Peptide
1320.7004	1319.6931	1319.7449	-39.21	103	-	114	1	---	R.AYILGVKENVSK.S
1702.9937	1701.9864	1701.9261	35.5	110	-	125	1	17	K.ENVSKSALIASVVTER.L

No match to: 1219.6710, 1284.6805, 1334.7179

Search Parameters

```
Type of search         : Sequence Query
Enzyme                 : Trypsin
Fixed modifications    : Carbamidomethyl (C)
Variable modifications : Oxidation (M)
Mass values            : Monoisotopic
Protein Mass           : Unrestricted
Peptide Mass Tolerance : ± 50 ppm
Fragment Mass Tolerance: ± 0.5 Da
Max Missed Cleavages   : 1
Instrument type        : MALDI-TOF-TOF
Query1 (1219.6710,1+)  : Locus:16.1.1.213515.57314
Query2 (1284.6805,1+)  : Locus:16.1.1.213515.57315
Query3 (1320.7004,1+)  : Locus:16.1.1.213515.57312
Query4 (1334.7179,1+)  : Locus:16.1.1.213515.57311
Query5 (1702.9937,1+)  : Locus:16.1.1.213515.57313
```

Mascot: http://www.matrixscience.com/

Mascot Search Results

```
User          :
Email         :
Search title  : F:\ROD_inmunoproteoma\MALDI\ppw_2016_562_563_ROD\ppw_G14_145397048001.txt
MS data file  : F:\ROD_inmunoproteoma\MALDI\ppw_2016_562_563_ROD\ppw_G14_145397048001.txt
Database      : Brachyspiraceae Brachyspiraceae_20160127-151122 (40573 sequences; 13470793 residues)
Timestamp     : 23 Feb 2016 at 16:05:05 GMT
Warning       : A Peptide summary report will usually give a much clearer picture of MS/MS search results.
Top Score     : 351 for A0A0H0W3D6, A0A0H0W3D6_BRAHO Enolase OS=Brachyspira hyodysenteriae GN=eno PE=3 SV=1
```

Probability Based Mowse Score

Protein score is -10*Log(P), where P is the probability that the observed match is a random event.
Protein scores greater than 59 are significant (p<0.05).
Protein scores are derived from ions scores as a non-probabilistic basis for ranking protein hits.

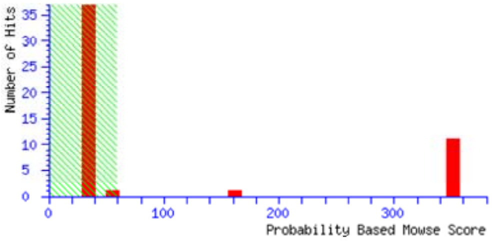

Protein Summary Report

Format As | Protein Summary (deprecated) | Help

Significance threshold p< 0.05 | Max. number of hits AUTO

Standard scoring ◉ MudPIT scoring ○ | Ions score or expect cut-off 0 | Show sub-sets 0

Show pop-ups ◉ Suppress pop-ups ○ | Sort unassigned Decreasing Score | Require bold red ☐

Re-Search All | Search Unmatched

Index

	Accession	Mass	Score	Description
1.	A0A0H0W3D6	47082	351	A0A0H0W3D6_BRAHO Enolase OS=Brachyspira hyodysenteriae GN=eno PE=3 SV=1
2.	A0A0G4KAH6	47151	351	A0A0G4KAH6_9SPIR Enolase OS=Brachyspira suanatina GN=eno PE=3 SV=1
3.	C0QV52	47082	351	C0QV52_BRAHW Enolase OS=Brachyspira hyodysenteriae (strain ATCC 49526 / WA1) GN=eno PE=3 SV=1
4.	A0A0H0XEZ9	47112	351	A0A0H0XEZ9_BRAHO Enolase OS=Brachyspira hyodysenteriae GN=eno PE=3 SV=1
5.	G0EQW4	47138	351	G0EQW4_BRAIP Enolase OS=Brachyspira intermedia (strain ATCC 51140 / PWS/A) GN=eno PE=3 SV=1
6.	J9URW4	47046	351	J9URW4_BRAPL Enolase OS=Brachyspira pilosicoli B2904 GN=eno PE=3 SV=1
7.	D8IET2	47046	351	D8IET2_BRAP9 Enolase OS=Brachyspira pilosicoli (strain ATCC BAA-1826 / 95/1000) GN=eno PE=3 SV=1
8.	K0JI69	47046	351	K0JI69_BRAPL Enolase OS=Brachyspira pilosicoli WesB GN=eno PE=3 SV=1
9.	D5U9W9	47170	351	D5U9W9_BRAM5 Enolase OS=Brachyspira murdochii (strain ATCC 51284 / DSM 12563 / 56-150) GN=eno PE=3 SV=1
10.	L8XSM5	47036	351	L8XSM5_9SPIR Enolase OS=Brachyspira hampsonii 30599 GN=eno PE=3 SV=1
11.	L0X4B5	46993	351	L0X4B5_9SPIR Enolase OS=Brachyspira hampsonii 30446 GN=eno PE=3 SV=1
12.	R5LML6	47006	154	R5LML6_9SPIR Enolase OS=Brachyspira sp. CAG:700 GN=eno PE=3 SV=1
13.	A0A0H0XDT2	71798	46	A0A0H0XDT2_BRAHO Chemotaxis protein OS=Brachyspira hyodysenteriae GN=SZ44_05160 PE=4 SV=1

Results List

1. A0A0H0W3D6 **Mass:** 47082 **Score:** 351 **Expect:** 3.2e-31 **Queries matched:** 3
A0A0H0W3D6_BRAHO Enolase OS=Brachyspira hyodysenteriae GN=eno PE=3 SV=1

Observed	Mr(expt)	Mr(calc)	ppm	Start	End	Miss	Ions	Peptide
1219.6086	1218.6013	1218.6397	-31.49	181 -	190	0	86	R.YVAEVFHNLK.A
1320.6356	1319.6283	1319.6543	-19.70	121 -	132	0	80	K.AAAEELGMPLYR.Y
1702.9001	1701.8928	1701.9414	-28.54	310 -	324	1	143	K.VQLVGDDLFVTNVKR.L

No match to: 1334.6422, 2439.1404

2. A0A0G4KAH6 **Mass:** 47151 **Score:** 351 **Expect:** 3.2e-31 **Queries matched:** 3
A0A0G4KAH6_9SPIR Enolase OS=Brachyspira suanatina GN=eno PE=3 SV=1

Observed	Mr(expt)	Mr(calc)	ppm	Start	End	Miss	Ions	Peptide
1219.6086	1218.6013	1218.6397	-31.49	181 -	190	0	86	R.YVAEVFHNLK.A
1320.6356	1319.6283	1319.6543	-19.70	121 -	132	0	80	K.AAAEELGMPLYR.Y
1702.9001	1701.8928	1701.9414	-28.54	310 -	324	1	143	K.VQLVGDDLFVTNVKR.L

No match to: 1334.6422, 2439.1404

3. C0QV52 **Mass:** 47082 **Score:** 351 **Expect:** 3.2e-31 **Queries matched:** 3
C0QV52_BRAHW Enolase OS=Brachyspira hyodysenteriae (strain ATCC 49526 / WA1) GN=eno PE=3 SV=1

Observed	Mr(expt)	Mr(calc)	ppm	Start	End	Miss	Ions	Peptide
1219.6086	1218.6013	1218.6397	-31.49	181 -	190	0	86	R.YVAEVFHNLK.A
1320.6356	1319.6283	1319.6543	-19.70	121 -	132	0	80	K.AAAEELGMPLYR.Y
1702.9001	1701.8928	1701.9414	-28.54	310 -	324	1	143	K.VQLVGDDLFVTNVKR.L

No match to: 1334.6422, 2439.1404

4. A0A0H0XEZ9 **Mass:** 47112 **Score:** 351 **Expect:** 3.2e-31 **Queries matched:** 3
A0A0H0XEZ9_BRAHO Enolase OS=Brachyspira hyodysenteriae GN=eno PE=3 SV=1

Observed	Mr(expt)	Mr(calc)	ppm	Start - End	Miss	Ions	Peptide
1219.6086	1218.6013	1218.6397	-31.49	181 - 190	0	86	R.YVAEVFHNLK.A
1320.6356	1319.6283	1319.6543	-19.70	121 - 132	0	80	K.AAAEELGMPLYR.Y
1702.9001	1701.8928	1701.9414	-28.54	310 - 324	1	143	K.VQLVGDDLFVTNVKR.L

No match to: 1334.6422, 2439.1404

5. G0EQW4 **Mass:** 47138 **Score:** 351 **Expect:** 3.2e-31 **Queries matched:** 3
G0EQW4_BRAIP Enolase OS=Brachyspira intermedia (strain ATCC 51140 / PWS/A) GN=eno PE=3 SV=1

Observed	Mr(expt)	Mr(calc)	ppm	Start - End	Miss	Ions	Peptide
1219.6086	1218.6013	1218.6397	-31.49	181 - 190	0	86	R.YVAEVFHNLK.A
1320.6356	1319.6283	1319.6543	-19.70	121 - 132	0	80	K.AAAEELGMPLYR.Y
1702.9001	1701.8928	1701.9414	-28.54	310 - 324	1	143	K.VQLVGDDLFVTNVKR.L

No match to: 1334.6422, 2439.1404

6. J9URW4 **Mass:** 47046 **Score:** 351 **Expect:** 3.2e-31 **Queries matched:** 3
J9URW4_BRAPL Enolase OS=Brachyspira pilosicoli B2904 GN=eno PE=3 SV=1

Observed	Mr(expt)	Mr(calc)	ppm	Start - End	Miss	Ions	Peptide
1219.6086	1218.6013	1218.6397	-31.49	181 - 190	0	86	R.YVAEVFHNLK.A
1320.6356	1319.6283	1319.6543	-19.70	121 - 132	0	80	K.AAAEELGMPLYR.Y
1702.9001	1701.8928	1701.9414	-28.54	310 - 324	1	143	K.VQLVGDDLFVTNVKR.L

No match to: 1334.6422, 2439.1404

7. D8IET2 **Mass:** 47046 **Score:** 351 **Expect:** 3.2e-31 **Queries matched:** 3
D8IET2_BRAP9 Enolase OS=Brachyspira pilosicoli (strain ATCC BAA-1826 / 95/1000) GN=eno PE=3 SV=1

Observed	Mr(expt)	Mr(calc)	ppm	Start - End	Miss	Ions	Peptide
1219.6086	1218.6013	1218.6397	-31.49	181 - 190	0	86	R.YVAEVFHNLK.A
1320.6356	1319.6283	1319.6543	-19.70	121 - 132	0	80	K.AAAEELGMPLYR.Y
1702.9001	1701.8928	1701.9414	-28.54	310 - 324	1	143	K.VQLVGDDLFVTNVKR.L

No match to: 1334.6422, 2439.1404

8. K0JI69 **Mass:** 47046 **Score:** 351 **Expect:** 3.2e-31 **Queries matched:** 3
K0JI69_BRAPL Enolase OS=Brachyspira pilosicoli WesB GN=eno PE=3 SV=1

Observed	Mr(expt)	Mr(calc)	ppm	Start - End	Miss	Ions	Peptide
1219.6086	1218.6013	1218.6397	-31.49	181 - 190	0	86	R.YVAEVFHNLK.A
1320.6356	1319.6283	1319.6543	-19.70	121 - 132	0	80	K.AAAEELGMPLYR.Y
1702.9001	1701.8928	1701.9414	-28.54	310 - 324	1	143	K.VQLVGDDLFVTNVKR.L

No match to: 1334.6422, 2439.1404

9. D5U9W9 **Mass:** 47170 **Score:** 351 **Expect:** 3.2e-31 **Queries matched:** 3
D5U9W9_BRAM5 Enolase OS=Brachyspira murdochii (strain ATCC 51284 / DSM 12563 / 56-150) GN=eno PE=3 SV=1

Observed	Mr(expt)	Mr(calc)	ppm	Start - End	Miss	Ions	Peptide
1219.6086	1218.6013	1218.6397	-31.49	181 - 190	0	86	R.YVAEVFHNLK.N
1320.6356	1319.6283	1319.6543	-19.70	121 - 132	0	80	K.AAAEELGMPLYR.Y
1702.9001	1701.8928	1701.9414	-28.54	310 - 324	1	143	K.VQLVGDDLFVTNVKR.L

No match to: 1334.6422, 2439.1404

10. L8XSM5 **Mass:** 47036 **Score:** 351 **Expect:** 3.2e-31 **Queries matched:** 3
L8XSM5_9SPIR Enolase OS=Brachyspira hampsonii 30599 GN=eno PE=3 SV=1

Observed	Mr(expt)	Mr(calc)	ppm	Start - End	Miss	Ions	Peptide
1219.6086	1218.6013	1218.6397	-31.49	181 - 190	0	86	R.YVAEVFHNLK.N
1320.6356	1319.6283	1319.6543	-19.70	121 - 132	0	80	K.AAAEELGMPLYR.Y
1702.9001	1701.8928	1701.9414	-28.54	310 - 324	1	143	K.VQLVGDDLFVTNVKR.L

No match to: 1334.6422, 2439.1404

11. L0X4B5 **Mass:** 46993 **Score:** 351 **Expect:** 3.2e-31 **Queries matched:** 3
L0X4B5_9SPIR Enolase OS=Brachyspira hampsonii 30446 GN=eno PE=3 SV=1

Observed	Mr(expt)	Mr(calc)	ppm	Start - End	Miss	Ions	Peptide
1219.6086	1218.6013	1218.6397	-31.49	181 - 190	0	86	R.YVAEVFHNLK.A
1320.6356	1319.6283	1319.6543	-19.70	121 - 132	0	80	K.AAAEELGMPLYR.Y
1702.9001	1701.8928	1701.9414	-28.54	310 - 324	1	143	K.VQLVGDDLFVTNVKR.L

No match to: 1334.6422, 2439.1404

12. R5LML6 **Mass:** 47006 **Score:** 154 **Expect:** 1.6e-11 **Queries matched:** 1
R5LML6_9SPIR Enolase OS=Brachyspira sp. CAG:700 GN=eno PE=3 SV=1

Observed	Mr(expt)	Mr(calc)	ppm	Start - End	Miss	Ions	Peptide
1702.9001	1701.8928	1701.9414	-28.54	310 - 324	1	143	K.VQLVGDDLFVTNVKR.L

No match to: 1219.6086, 1320.6356, 1334.6422, 2439.1404

13. A0A0H0XDT2 **Mass:** 71798 **Score:** 46 **Expect:** 0.95 **Queries matched:** 3
A0A0H0XDT2_BRAHO Chemotaxis protein OS=Brachyspira hyodysenteriae GN=SZ44_05160 PE=4 SV=1

Observed	Mr(expt)	Mr(calc)	ppm	Start - End	Miss	Ions	Peptide
1219.6086	1218.6013	1218.6219	-16.91	64 - 72	0	10	K.HLYNVMWLK.V + Oxidation (M)
1320.6356	1319.6283	1319.6391	-8.17	350 - 361	0	---	K.DILAMAEGDLTR.E + Oxidation (M)
1334.6422	1333.6349	1333.6725	-28.18	541 - 552	0	---	K.NITSLITDSNEK.I

No match to: 1702.9001, 2439.1404

Search Parameters

```
Type of search         : Sequence Query
Enzyme                 : Trypsin
Fixed modifications    : Carbamidomethyl (C)
Variable modifications : Oxidation (M)
Mass values            : Monoisotopic
Protein Mass           : Unrestricted
Peptide Mass Tolerance : ± 50 ppm
Fragment Mass Tolerance: ± 0.5 Da
Max Missed Cleavages   : 1
Instrument type        : MALDI-TOF-TOF
Query1 (1219.6086,1+)  : Locus:2.1.1.211130.56523
Query2 (1320.6356,1+)  : Locus:2.1.1.211130.56520
Query3 (1334.6422,1+)  : Locus:2.1.1.211130.56521
Query4 (1702.9001,1+)  : Locus:2.1.1.211130.56522
Query5 (2439.1404,1+)  : Locus:2.1.1.211130.56524
```

Mascot: http://www.matrixscience.com/

Mascot Search Results

```
User            :
Email           :
Search title    : F:\ROD_inmunoproteoma\MALDI\ppw_2016_561_562_563_565\ppw_H9_145467559021.txt
MS data file    : F:\ROD_inmunoproteoma\MALDI\ppw_2016_561_562_563_565\ppw_H9_145467559021.txt
Database        : Brachyspiraceae Brachyspiraceae_20160127-151122 (40573 sequences; 13470793 residues)
Timestamp       : 23 Feb 2016 at 16:37:05 GMT
Warning         : A Peptide summary report will usually give a much clearer picture of MS/MS search results.
Top Score       : 253 for C0QW84, C0QW84_BRAHW Uncharacterized protein OS=Brachyspira hyodysenteriae (strain ATCC 49526 / WA1) GN=BHWA1_0221
```

Probability Based Mowse Score

Protein score is -10*Log(P), where P is the probability that the observed match is a random event.
Protein scores greater than 59 are significant (p<0.05).
Protein scores are derived from ions scores as a non-probabilistic basis for ranking protein hits.

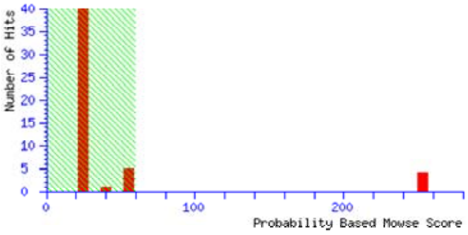

Protein Summary Report

Format As | Protein Summary (deprecated) | Help

Significance threshold p< 0.05 | Max. number of hits AUTO

Standard scoring ◉ MudPIT scoring ○ | Ions score or expect cut-off 0 | Show sub-sets 0

Show pop-ups ◉ Suppress pop-ups ○ | Sort unassigned Decreasing Score | Require bold red ☐

Re-Search All | Search Unmatched

Index

	Accession	Mass	Score	Description
1.	C0QW84	38618	253	C0QW84_BRAHW Uncharacterized protein OS=Brachyspira hyodysenteriae (strain ATCC 49526 / WA1) GN=BHWA1_02212 PE=
2.	A0A0H0XF11	38956	253	A0A0H0XF11_BRAHO Uncharacterized protein OS=Brachyspira hyodysenteriae GN=SZ44_10735 PE=4 SV=1
3.	A0A0H0U2P4	39777	252	A0A0H0U2P4_BRAHO Uncharacterized protein OS=Brachyspira hyodysenteriae GN=SR30_05440 PE=4 SV=1
4.	A0A0H0V4Q6	39805	252	A0A0H0V4Q6_BRAHO Uncharacterized protein OS=Brachyspira hyodysenteriae GN=SZ48_10380 PE=4 SV=1
5.	A0A0G4K3Q4	39617	50	A0A0G4K3Q4_9SPIR Uncharacterized protein OS=Brachyspira suanatina GN=BRSU_0279 PE=4 SV=1

Results List

1. C0QW84 **Mass:** 38618 **Score:** 253 **Expect:** 2e-21 **Queries matched:** 2
C0QW84_BRAHW Uncharacterized protein OS=Brachyspira hyodysenteriae (strain ATCC 49526 / WA1) GN=BHWA1_02212 PE=4 SV=1

Observed	Mr(expt)	Mr(calc)	ppm	Start	End	Miss	Ions	Peptide
1129.5786	1128.5713	1128.5386	29.0	78 -	85	1	39	K.MREDYLFR.V
2255.1108	2254.1035	2254.0430	26.9	286 -	304	0	187	K.YYDELGFVYGQTESEVLSR.E

No match to: 1112.6509, 1143.5909, 2267.1951

2. A0A0H0XF11 **Mass:** 38956 **Score:** 253 **Expect:** 2e-21 **Queries matched:** 2
A0A0H0XF11_BRAHO Uncharacterized protein OS=Brachyspira hyodysenteriae GN=SZ44_10735 PE=4 SV=1

Observed	Mr(expt)	Mr(calc)	ppm	Start	End	Miss	Ions	Peptide
1129.5786	1128.5713	1128.5386	29.0	80 -	87	1	39	K.MREDYLFR.V
2255.1108	2254.1035	2254.0430	26.9	288 -	306	0	187	K.YYDELGFVYGQTESEVLSR.E

No match to: 1112.6509, 1143.5909, 2267.1951

3. A0A0H0U2P4 **Mass:** 39777 **Score:** 252 **Expect:** 2.6e-21 **Queries matched:** 2
A0A0H0U2P4_BRAHO Uncharacterized protein OS=Brachyspira hyodysenteriae GN=SR30_05440 PE=4 SV=1

Observed	Mr(expt)	Mr(calc)	ppm	Start	End	Miss	Ions	Peptide
1129.5786	1128.5713	1128.5386	29.0	88 -	95	1	39	K.MREDYLFR.V
2255.1108	2254.1035	2254.0430	26.9	296 -	314	0	187	K.YYDELGFVYGQTESEVLSR.E

No match to: 1112.6509, 1143.5909, 2267.1951

4. A0A0H0V4Q6 **Mass:** 39805 **Score:** 252 **Expect:** 2.6e-21 **Queries matched:** 2
A0A0H0V4Q6_BRAHO Uncharacterized protein OS=Brachyspira hyodysenteriae GN=SZ48_10380 PE=4 SV=1

Observed	Mr(expt)	Mr(calc)	ppm	Start	End	Miss	Ions	Peptide
1129.5786	1128.5713	1128.5386	29.0	88 -	95	1	39	K.MREDYLFR.V
2255.1108	2254.1035	2254.0430	26.9	296 -	314	0	187	K.YYDELGFVYGQTESEVLSR.E

No match to: 1112.6509, 1143.5909, 2267.1951

5. A0A0G4K3Q4 **Mass:** 39617 **Score:** 50 **Expect:** 0.46 **Queries matched:** 1
A0A0G4K3Q4_9SPIR Uncharacterized protein OS=Brachyspira suanatina GN=BRSU_0279 PE=4 SV=1

Observed	Mr(expt)	Mr(calc)	ppm	Start	End	Miss	Ions	Peptide
1129.5786	1128.5713	1128.5386	29.0	88 -	95	1	39	K.MREDYLFR.V

No match to: 1112.6509, 1143.5909, 2255.1108, 2267.1951

Search Parameters

```
Type of search         : Sequence Query
Enzyme                 : Trypsin
Fixed modifications    : Carbamidomethyl (C)
Variable modifications : Oxidation (M)
Mass values            : Monoisotopic
Protein Mass           : Unrestricted
Peptide Mass Tolerance : ± 50 ppm
Fragment Mass Tolerance: ± 0.5 Da
Max Missed Cleavages   : 1
Instrument type        : MALDI-TOF-TOF
Query1 (1112.6509,1+)  : Locus:9.1.1.212743.57042
Query2 (1129.5786,1+)  : Locus:9.1.1.212743.57039
Query3 (1143.5909,1+)  : Locus:9.1.1.212743.57041
Query4 (2255.1108,1+)  : Locus:9.1.1.212743.57040
Query5 (2267.1951,1+)  : Locus:9.1.1.212743.57043
```

**Mascot:** http://www.matrixscience.com/

Mascot Search Results

**User**	:	
**Email**	:	
**Search title**	:	F:\ROD_inmunoproteoma\MALDI\ppw_2014_442_ROD\ppw_H9_139601422132.txt
**MS data file**	:	F:\ROD_inmunoproteoma\MALDI\ppw_2014_442_ROD\ppw_H9_139601422132.txt
**Database**	:	Brachyspiraceae Brachyspiraceae_20160127-151122 (40573 sequences; 13470793 residues)
**Timestamp**	:	22 Feb 2016 at 15:15:10 GMT
**Warning**	:	A Peptide summary report will usually give a much clearer picture of MS/MS search results.
**Top Score**	:	111 for Q9FA06, Q9FA06_BRAPL Putative elongation factor OS=Brachyspira pilosicoli GN=tuf PE=4 SV=1

Probability Based Mowse Score

Protein score is -10*Log(P), where P is the probability that the observed match is a random event.
Protein scores greater than 59 are significant (p<0.05).
Protein scores are derived from ions scores as a non-probabilistic basis for ranking protein hits.

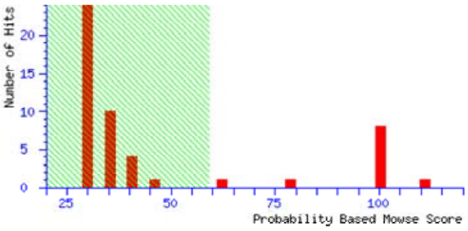

Protein Summary Report

Format As | Protein Summary (deprecated) | Help

Significance threshold p< 0.05 | Max. number of hits AUTO

Standard scoring ◉ MudPIT scoring ○ | Ions score or expect cut-off 0 | Show sub-sets 0

Show pop-ups ◉ Suppress pop-ups ○ | Sort unassigned Decreasing Score | Require bold red ☐

Re-Search All | Search Unmatched

Index

	Accession	Mass	Score	Description
1.	Q9FA06	16290	111	Q9FA06_BRAPL Putative elongation factor OS=Brachyspira pilosicoli GN=tuf PE=4 SV=1
2.	C0QVZ4	44776	101	EFTU_BRAHW Elongation factor Tu OS=Brachyspira hyodysenteriae (strain ATCC 49526 / WA1) GN=tuf PE=3 SV=1
3.	A0A0H0TPC4	44776	101	A0A0H0TPC4_BRAHO Elongation factor Tu OS=Brachyspira hyodysenteriae GN=tuf PE=3 SV=1
4.	A0A0G4K3M8	44733	101	A0A0G4K3M8_9SPIR Elongation factor Tu OS=Brachyspira suanatina GN=tuf PE=3 SV=1
5.	G0ENS4	44733	101	G0ENS4_BRAIP Elongation factor Tu OS=Brachyspira intermedia (strain ATCC 51140 / PWS/A) GN=tufA PE=3 SV=1
6.	J9UB96	44777	101	J9UB96_BRAPL Elongation factor Tu OS=Brachyspira pilosicoli B2904 GN=el-tu PE=3 SV=1
7.	D8ICZ6	44826	101	D8ICZ6_BRAP9 Elongation factor Tu OS=Brachyspira pilosicoli (strain ATCC BAA-1826 / 95/1000) GN=tuf PE=3 SV=1
8.	K0JL99	44826	101	K0JL99_BRAPL Elongation factor Tu OS=Brachyspira pilosicoli WesB GN=tufA PE=3 SV=1
9.	P52854	45030	101	EFTU_BRAHO Elongation factor Tu OS=Brachyspira hyodysenteriae GN=tuf PE=3 SV=1
10.	R5LAV7	44698	78	R5LAV7_9SPIR Elongation factor Tu OS=Brachyspira sp. CAG:700 GN=tuf PE=3 SV=1
11.	L8XS56	44815	60	L8XS56_9SPIR Elongation factor Tu OS=Brachyspira hampsonii 30599 GN=tuf PE=3 SV=1
12.	R5HH89	11666	48	R5HH89_9SPIR Histidine triad (HIT) family protein OS=Brachyspira sp. CAG:484 GN=BN676_00702 PE=4 SV=1

Results List

1. Q9FA06 **Mass:** 16290 **Score:** 111 **Expect:** 3.2e-07 **Queries matched:** 3
Q9FA06_BRAPL Putative elongation factor OS=Brachyspira pilosicoli GN=tuf PE=4 SV=1

Observed	Mr(expt)	Mr(calc)	ppm	Start	End	Miss	Ions	Peptide
1619.8923	1618.8850	1618.8501	21.6	53 -	67	0	47	K.EVVGIAGYNVGCLLR.G
1831.9127	1830.9054	1830.8624	23.5	107 -	121	0	(3)	R.HSGFVSGYRPQMYFR.T
1847.9136	1846.9063	1846.8573	26.5	107 -	121	0	20	R.HSGFVSGYRPQMYFR.T + Oxidation (M)

2. C0QVZ4 **Mass:** 44776 **Score:** 101 **Expect:** 3.2e-06 **Queries matched:** 3
EFTU_BRAHW Elongation factor Tu OS=Brachyspira hyodysenteriae (strain ATCC 49526 / WA1) GN=tuf PE=3 SV=1

Observed	Mr(expt)	Mr(calc)	ppm	Start	End	Miss	Ions	Peptide
1619.8923	1618.8850	1618.8501	21.6	280 -	294	0	47	K.EVVGIAGYNVGCLLR.G
1831.9127	1830.9054	1830.8624	23.5	334 -	348	0	(3)	R.HSGFVSGYRPQMYFR.T
1847.9136	1846.9063	1846.8573	26.5	334 -	348	0	20	R.HSGFVSGYRPQMYFR.T + Oxidation (M)

3. A0A0H0TPC4 **Mass:** 44776 **Score:** 101 **Expect:** 3.2e-06 **Queries matched:** 3
A0A0H0TPC4_BRAHO Elongation factor Tu OS=Brachyspira hyodysenteriae GN=tuf PE=3 SV=1

Observed	Mr(expt)	Mr(calc)	ppm	Start	End	Miss	Ions	Peptide
1619.8923	1618.8850	1618.8501	21.6	280 -	294	0	47	K.EVVGIAGYNVGCLLR.G
1831.9127	1830.9054	1830.8624	23.5	334 -	348	0	(3)	R.HSGFVSGYRPQMYFR.T
1847.9136	1846.9063	1846.8573	26.5	334 -	348	0	20	R.HSGFVSGYRPQMYFR.T + Oxidation (M)

4. A0A0G4K3M8 **Mass:** 44733 **Score:** 101 **Expect:** 3.2e-06 **Queries matched:** 3
A0A0G4K3M8_9SPIR Elongation factor Tu OS=Brachyspira suanatina GN=tuf PE=3 SV=1

Observed	Mr(expt)	Mr(calc)	ppm	Start	End	Miss	Ions	Peptide
1619.8923	1618.8850	1618.8501	21.6	280 -	294	0	47	K.EVVGIAGYNVGCLLR.G
1831.9127	1830.9054	1830.8624	23.5	334 -	348	0	(3)	R.HSGFVSGYRPQMYFR.T
1847.9136	1846.9063	1846.8573	26.5	334 -	348	0	20	R.HSGFVSGYRPQMYFR.T + Oxidation (M)

5. G0ENS4 **Mass:** 44733 **Score:** 101 **Expect:** 3.2e-06 **Queries matched:** 3
G0ENS4_BRAIP Elongation factor Tu OS=Brachyspira intermedia (strain ATCC 51140 / PWS/A) GN=tufA PE=3 SV=1

Observed	Mr(expt)	Mr(calc)	ppm	Start	End	Miss	Ions	Peptide
1619.8923	1618.8850	1618.8501	21.6	280 -	294	0	47	K.EVVGIAGYNVGCLLR.G
1831.9127	1830.9054	1830.8624	23.5	334 -	348	0	(3)	R.HSGFVSGYRPQMYFR.T
1847.9136	1846.9063	1846.8573	26.5	334 -	348	0	20	R.HSGFVSGYRPQMYFR.T + Oxidation (M)

6. J9UB96 **Mass:** 44777 **Score:** 101 **Expect:** 3.2e-06 **Queries matched:** 3
J9UB96_BRAPL Elongation factor Tu OS=Brachyspira pilosicoli B2904 GN=el-tu PE=3 SV=1

Observed	Mr(expt)	Mr(calc)	ppm	Start	End	Miss	Ions	Peptide
1619.8923	1618.8850	1618.8501	21.6	280 -	294	0	47	K.EVVGIAGYNVGCLLR.G
1831.9127	1830.9054	1830.8624	23.5	334 -	348	0	(3)	R.HSGFVSGYRPQMYFR.T
1847.9136	1846.9063	1846.8573	26.5	334 -	348	0	20	R.HSGFVSGYRPQMYFR.T + Oxidation (M)

7. D8ICZ6 **Mass:** 44826 **Score:** 101 **Expect:** 3.2e-06 **Queries matched:** 3
D8ICZ6_BRAP9 Elongation factor Tu OS=Brachyspira pilosicoli (strain ATCC BAA-1826 / 95/1000) GN=tuf PE=3 SV=1

Observed	Mr(expt)	Mr(calc)	ppm	Start	End	Miss	Ions	Peptide
1619.8923	1618.8850	1618.8501	21.6	280 -	294	0	47	K.EVVGIAGYNVGCLLR.G
1831.9127	1830.9054	1830.8624	23.5	334 -	348	0	(3)	R.HSGFVSGYRPQMYFR.T
1847.9136	1846.9063	1846.8573	26.5	334 -	348	0	20	R.HSGFVSGYRPQMYFR.T + Oxidation (M)

8. K0JL99 **Mass:** 44826 **Score:** 101 **Expect:** 3.2e-06 **Queries matched:** 3
K0JL99_BRAPL Elongation factor Tu OS=Brachyspira pilosicoli WesB GN=tufA PE=3 SV=1

Observed	Mr(expt)	Mr(calc)	ppm	Start	End	Miss	Ions	Peptide
1619.8923	1618.8850	1618.8501	21.6	280 -	294	0	47	K.EVVGIAGYNVGCLLR.G
1831.9127	1830.9054	1830.8624	23.5	334 -	348	0	(3)	R.HSGFVSGYRPQMYFR.T
1847.9136	1846.9063	1846.8573	26.5	334 -	348	0	20	R.HSGFVSGYRPQMYFR.T + Oxidation (M)

9. P52854 **Mass:** 45030 **Score:** 101 **Expect:** 3.2e-06 **Queries matched:** 3
EFTU_BRAHO Elongation factor Tu OS=Brachyspira hyodysenteriae GN=tuf PE=3 SV=1

Observed	Mr(expt)	Mr(calc)	ppm	Start	End	Miss	Ions	Peptide
1619.8923	1618.8850	1618.8501	21.6	280 -	294	0	47	K.EVVGIAGYNVGCLLR.G
1831.9127	1830.9054	1830.8624	23.5	334 -	348	0	(3)	R.HSGFVSGYRPQMYFR.T
1847.9136	1846.9063	1846.8573	26.5	334 -	348	0	20	R.HSGFVSGYRPQMYFR.T + Oxidation (M)

10. R5LAV7 **Mass:** 44698 **Score:** 78 **Expect:** 0.00066 **Queries matched:** 2
R5LAV7_9SPIR Elongation factor Tu OS=Brachyspira sp. CAG:700 GN=tuf PE=3 SV=1

Observed	Mr(expt)	Mr(calc)	ppm	Start	End	Miss	Ions	Peptide
1619.8923	1618.8850	1618.8501	21.6	279 -	293	0	47	K.EVVGIAGYNVGCLLR.G
1847.9136	1846.9063	1846.9061	0.13	66 -	81	0	---	K.ILTIATSHVEYESDNR.H

No match to: 1831.9127

11. L8XS56 **Mass:** 44815 **Score:** 60 **Expect:** 0.042 **Queries matched:** 1
L8XS56_9SPIR Elongation factor Tu OS=Brachyspira hampsonii 30599 GN=tuf PE=3 SV=1

Observed	Mr(expt)	Mr(calc)	ppm	Start	End	Miss	Ions	Peptide
1619.8923	1618.8850	1618.8501	21.6	280 -	294	0	47	K.EVVGIAGYNVGCLLR.G

No match to: 1831.9127, 1847.9136

12. R5HH89 **Mass:** 11666 **Score:** 48 **Expect:** 0.69 **Queries matched:** 3
R5HH89_9SPIR Histidine triad (HIT) family protein OS=Brachyspira sp. CAG:484 GN=BN676_00702 PE=4 SV=1

Observed	Mr(expt)	Mr(calc)	ppm	Start	End	Miss	Ions	Peptide
1619.8923	1618.8850	1618.8422	26.4	58 -	72	1	---	K.NLMGELLMGVKEAAK.K + Oxidation (M)
1831.9127	1830.9054	1830.9199	-7.92	88 -	103	0	---	K.EAGQEVFHLHIHVMGK.-
1847.9136	1846.9063	1846.9148	-4.61	88 -	103	0	---	K.EAGQEVFHLHIHVMGK.- + Oxidation (M)

Search Parameters

Type of search : Sequence Query
Enzyme : Trypsin
Fixed modifications : Carbamidomethyl (C)
Variable modifications : Oxidation (M)
Mass values : Monoisotopic
Protein Mass : Unrestricted
Peptide Mass Tolerance : ± 50 ppm
Fragment Mass Tolerance: ± 0.5 Da
Max Missed Cleavages : 1
Instrument type : MALDI-TOF-TOF
Query1 (1619.8923,1+) : Locus:33.1.1.170631.52832
Query2 (1831.9127,1+) : Locus:33.1.1.170631.52831
Query3 (1847.9136,1+) : Locus:33.1.1.170631.52833

Mascot: http://www.matrixscience.com/

MATRIX SCIENCE Mascot Search Results

**User**	:	
**Email**	:	
**Search title**	:	**F:\ROD_inmunoproteoma\MALDI\ppw_2016_562_563_ROD\ppw_G15_145397048102.txt**
**MS data file**	:	**F:\ROD_inmunoproteoma\MALDI\ppw_2016_562_563_ROD\ppw_G15_145397048102.txt**
**Database**	:	**Brachyspiraceae Brachyspiraceae_20160127-151122 (40573 sequences; 13470793 residues)**
**Timestamp**	:	**23 Feb 2016 at 16:05:07 GMT**
**Warning**	:	**A Peptide summary report will usually give a much clearer picture of MS/MS search results.**
**Top Score**	:	**230 for Q9FA06, Q9FA06_BRAPL Putative elongation factor OS=Brachyspira pilosicoli GN=tuf PE=4 SV=1**

Probability Based Mowse Score

Protein score is -10*Log(P), where P is the probability that the observed match is a random event.
Protein scores greater than 59 are significant (p<0.05).
Protein scores are derived from ions scores as a non-probabilistic basis for ranking protein hits.

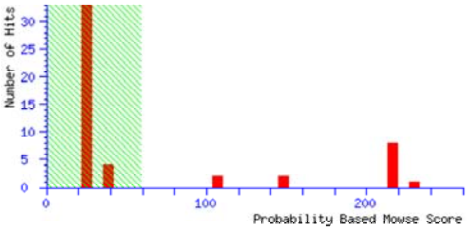

Protein Summary Report

Format As | Protein Summary (deprecated) | Help

Significance threshold p< 0.05 | Max. number of hits AUTO

Standard scoring ◉ MudPIT scoring ○ | Ions score or expect cut-off 0 | Show sub-sets 0

Show pop-ups ◉ Suppress pop-ups ○ | Sort unassigned Decreasing Score | Require bold red ☐

Re-Search All | Search Unmatched

Index

	Accession	Mass	Score	Description
1.	Q9FA06	16290	230	Q9FA06_BRAPL Putative elongation factor OS=Brachyspira pilosicoli GN=tuf PE=4 SV=1
2.	C0QVZ4	44776	221	EFTU_BRAHW Elongation factor Tu OS=Brachyspira hyodysenteriae (strain ATCC 49526 / WA1) GN=tuf PE=3 SV=1
3.	A0A0H0TPC4	44776	221	A0A0H0TPC4_BRAHO Elongation factor Tu OS=Brachyspira hyodysenteriae GN=tuf PE=3 SV=1
4.	A0A0G4K3M8	44733	221	A0A0G4K3M8_9SPIR Elongation factor Tu OS=Brachyspira suanatina GN=tuf PE=3 SV=1
5.	G0ENS4	44733	221	G0ENS4_BRAIP Elongation factor Tu OS=Brachyspira intermedia (strain ATCC 51140 / PWS/A) GN=tufA PE=3 SV=1
6.	J9UB96	44777	221	J9UB96_BRAPL Elongation factor Tu OS=Brachyspira pilosicoli B2904 GN=el-tu PE=3 SV=1
7.	D8ICZ6	44826	221	D8ICZ6_BRAP9 Elongation factor Tu OS=Brachyspira pilosicoli (strain ATCC BAA-1826 / 95/1000) GN=tuf PE=3 SV=1
8.	K0JL99	44826	221	K0JL99_BRAPL Elongation factor Tu OS=Brachyspira pilosicoli WesB GN=tufA PE=3 SV=1
9.	P52854	45030	221	EFTU_BRAHO Elongation factor Tu OS=Brachyspira hyodysenteriae GN=tuf PE=3 SV=1
10.	R5LAV7	44698	143	R5LAV7_9SPIR Elongation factor Tu OS=Brachyspira sp. CAG:700 GN=tuf PE=3 SV=1
11.	L8XS56	44815	143	L8XS56_9SPIR Elongation factor Tu OS=Brachyspira hampsonii 30599 GN=tuf PE=3 SV=1
12.	D5UB43	44823	104	D5UB43_BRAM5 Elongation factor Tu OS=Brachyspira murdochii (strain ATCC 51284 / DSM 12563 / 56-150) GN=tuf PE=
13.	L0X3D8	44918	104	L0X3D8_9SPIR Elongation factor Tu OS=Brachyspira hampsonii 30446 GN=tuf PE=3 SV=1
14.	A0A0H0XJZ0	16086	35	A0A0H0XJZ0_BRAHO Uncharacterized protein OS=Brachyspira hyodysenteriae GN=SU45_06435 PE=4 SV=1

Results List

1. Q9FA06 **Mass:** 16290 **Score:** 230 **Expect:** 4.1e-19 **Queries matched:** 2
Q9FA06_BRAPL Putative elongation factor OS=Brachyspira pilosicoli GN=tuf PE=4 SV=1

Observed	Mr(expt)	Mr(calc)	ppm	Start	End	Miss	Ions	Peptide
1619.8033	1618.7960	1618.8501	-33.41	53 -	67	0	102	K.EVVGIAGYNVGCLLR.G
1831.8177	1830.8104	1830.8624	-28.39	107 -	121	0	93	R.HSGFVSGYRPQMYFR.T

No match to: 1633.8173, 1814.8945, 1845.8223

2. C0QVZ4 **Mass:** 44776 **Score:** 221 **Expect:** 3.2e-18 **Queries matched:** 2
EFTU_BRAHW Elongation factor Tu OS=Brachyspira hyodysenteriae (strain ATCC 49526 / WA1) GN=tuf PE=3 SV=1

Observed	Mr(expt)	Mr(calc)	ppm	Start	End	Miss	Ions	Peptide
1619.8033	1618.7960	1618.8501	-33.41	280 -	294	0	102	K.EVVGIAGYNVGCLLR.G
1831.8177	1830.8104	1830.8624	-28.39	334 -	348	0	93	R.HSGFVSGYRPQMYFR.T

No match to: 1633.8173, 1814.8945, 1845.8223

3. A0A0H0TPC4 **Mass:** 44776 **Score:** 221 **Expect:** 3.2e-18 **Queries matched:** 2
A0A0H0TPC4_BRAHO Elongation factor Tu OS=Brachyspira hyodysenteriae GN=tuf PE=3 SV=1

Observed	Mr(expt)	Mr(calc)	ppm	Start	End	Miss	Ions	Peptide
1619.8033	1618.7960	1618.8501	-33.41	280 -	294	0	102	K.EVVGIAGYNVGCLLR.G
1831.8177	1830.8104	1830.8624	-28.39	334 -	348	0	93	R.HSGFVSGYRPQMYFR.T

No match to: 1633.8173, 1814.8945, 1845.8223

4. A0A0G4K3M8 **Mass:** 44733 **Score:** 221 **Expect:** 3.2e-18 **Queries matched:** 2
A0A0G4K3M8_9SPIR Elongation factor Tu OS=Brachyspira suanatina GN=tuf PE=3 SV=1

Observed	Mr(expt)	Mr(calc)	ppm	Start	End	Miss	Ions	Peptide
1619.8033	1618.7960	1618.8501	-33.41	280 -	294	0	102	K.EVVGIAGYNVGCLLR.G
1831.8177	1830.8104	1830.8624	-28.39	334 -	348	0	93	R.HSGFVSGYRPQMYFR.T

```
      No match to: 1633.8173, 1814.8945, 1845.8223

5.    G0ENS4     Mass: 44733    Score: 221    Expect: 3.2e-18  Queries matched: 2
      G0ENS4_BRAIP Elongation factor Tu OS=Brachyspira intermedia (strain ATCC 51140 / PWS/A) GN=tufA PE=3 SV=1
       Observed    Mr(expt)   Mr(calc)     ppm  Start    End Miss Ions  Peptide
      1619.8033  1618.7960  1618.8501  -33.41    280 - 294    0  102    K.EVVGIAGYNVGCLLR.G
      1831.8177  1830.8104  1830.8624  -28.39    334 - 348    0   93    R.HSGFVSGYRPQMYFR.T
      No match to: 1633.8173, 1814.8945, 1845.8223

6.    J9UB96     Mass: 44777    Score: 221    Expect: 3.2e-18  Queries matched: 2
      J9UB96_BRAPL Elongation factor Tu OS=Brachyspira pilosicoli B2904 GN=el-tu PE=3 SV=1
       Observed    Mr(expt)   Mr(calc)     ppm  Start    End Miss Ions  Peptide
      1619.8033  1618.7960  1618.8501  -33.41    280 - 294    0  102    K.EVVGIAGYNVGCLLR.G
      1831.8177  1830.8104  1830.8624  -28.39    334 - 348    0   93    R.HSGFVSGYRPQMYFR.T
      No match to: 1633.8173, 1814.8945, 1845.8223

7.    D8ICZ6     Mass: 44826    Score: 221    Expect: 3.2e-18  Queries matched: 2
      D8ICZ6_BRAP9 Elongation factor Tu OS=Brachyspira pilosicoli (strain ATCC BAA-1826 / 95/1000) GN=tuf PE=3 SV=1
       Observed    Mr(expt)   Mr(calc)     ppm  Start    End Miss Ions  Peptide
      1619.8033  1618.7960  1618.8501  -33.41    280 - 294    0  102    K.EVVGIAGYNVGCLLR.G
      1831.8177  1830.8104  1830.8624  -28.39    334 - 348    0   93    R.HSGFVSGYRPQMYFR.T
      No match to: 1633.8173, 1814.8945, 1845.8223

8.    K0JL99     Mass: 44826    Score: 221    Expect: 3.2e-18  Queries matched: 2
      K0JL99_BRAPL Elongation factor Tu OS=Brachyspira pilosicoli WesB GN=tufA PE=3 SV=1
       Observed    Mr(expt)   Mr(calc)     ppm  Start    End Miss Ions  Peptide
      1619.8033  1618.7960  1618.8501  -33.41    280 - 294    0  102    K.EVVGIAGYNVGCLLR.G
      1831.8177  1830.8104  1830.8624  -28.39    334 - 348    0   93    R.HSGFVSGYRPQMYFR.T
      No match to: 1633.8173, 1814.8945, 1845.8223

9.    P52854     Mass: 45030    Score: 221    Expect: 3.2e-18  Queries matched: 2
      EFTU_BRAHO Elongation factor Tu OS=Brachyspira hyodysenteriae GN=tuf PE=3 SV=1
       Observed    Mr(expt)   Mr(calc)     ppm  Start    End Miss Ions  Peptide
      1619.8033  1618.7960  1618.8501  -33.41    280 - 294    0  102    K.EVVGIAGYNVGCLLR.G
      1831.8177  1830.8104  1830.8624  -28.39    334 - 348    0   93    R.HSGFVSGYRPQMYFR.T
      No match to: 1633.8173, 1814.8945, 1845.8223

10.   R5LAV7     Mass: 44698    Score: 143    Expect: 2e-10  Queries matched: 2
      R5LAV7_9SPIR Elongation factor Tu OS=Brachyspira sp. CAG:700 GN=tuf PE=3 SV=1
       Observed    Mr(expt)   Mr(calc)     ppm  Start    End Miss Ions  Peptide
      1619.8033  1618.7960  1618.8501  -33.41    279 - 293    0  102    K.EVVGIAGYNVGCLLR.G
      1845.8223  1844.8150  1844.8781  -34.17    333 - 347    0   15    R.HSGFVTGYRPQMYFR.T
      No match to: 1633.8173, 1814.8945, 1831.8177

11.   L8XS56     Mass: 44815    Score: 143    Expect: 2e-10  Queries matched: 2
      L8XS56_9SPIR Elongation factor Tu OS=Brachyspira hampsonii 30599 GN=tuf PE=3 SV=1
       Observed    Mr(expt)   Mr(calc)     ppm  Start    End Miss Ions  Peptide
      1619.8033  1618.7960  1618.8501  -33.41    280 - 294    0  102    K.EVVGIAGYNVGCLLR.G
      1845.8223  1844.8150  1844.8781  -34.17    334 - 348    0   15    R.HSGFVTGYRPQMYFR.T
      No match to: 1633.8173, 1814.8945, 1831.8177

12.   D5UB43     Mass: 44823    Score: 104    Expect: 1.6e-06  Queries matched: 1
      D5UB43_BRAM5 Elongation factor Tu OS=Brachyspira murdochii (strain ATCC 51284 / DSM 12563 / 56-150) GN=tuf PE=3 SV=1
       Observed    Mr(expt)   Mr(calc)     ppm  Start    End Miss Ions  Peptide
      1831.8177  1830.8104  1830.8624  -28.39    334 - 348    0   93    R.HSGFVSGYRPQMYFR.T
      No match to: 1619.8033, 1633.8173, 1814.8945, 1845.8223

13.   L0X3D8     Mass: 44918    Score: 104    Expect: 1.6e-06  Queries matched: 1
      L0X3D8_9SPIR Elongation factor Tu OS=Brachyspira hampsonii 30446 GN=tuf PE=3 SV=1
       Observed    Mr(expt)   Mr(calc)     ppm  Start    End Miss Ions  Peptide
      1831.8177  1830.8104  1830.8624  -28.39    334 - 348    0   93    R.HSGFVSGYRPQMYFR.T
      No match to: 1619.8033, 1633.8173, 1814.8945, 1845.8223

14.   A0A0H0XJZ0     Mass: 16086    Score: 35     Expect: 12  Queries matched: 2
      A0A0H0XJZ0_BRAHO Uncharacterized protein OS=Brachyspira hyodysenteriae GN=SU45_06435 PE=4 SV=1
       Observed    Mr(expt)   Mr(calc)     ppm  Start    End Miss Ions  Peptide
      1619.8033  1618.7960  1618.7641    19.8     12 -  23    1  ---    K.NNNVNFYERFFR.I
      1845.8223  1844.8150  1844.9057  -49.15     97 - 113    1  ---    K.IDDNSFSGARSFAFALK.D
      No match to: 1633.8173, 1814.8945, 1831.8177
```

Search Parameters

```
Type of search         : Sequence Query
Enzyme                 : Trypsin
Fixed modifications    : Carbamidomethyl (C)
Variable modifications : Oxidation (M)
Mass values            : Monoisotopic
Protein Mass           : Unrestricted
Peptide Mass Tolerance : ± 50 ppm
Fragment Mass Tolerance: ± 0.5 Da
Max Missed Cleavages   : 1
Instrument type        : MALDI-TOF-TOF
Query1 (1619.8033,1+)  : Locus:3.1.1.211131.56526
Query2 (1633.8173,1+)  : Locus:3.1.1.211131.56527
Query3 (1814.8945,1+)  : Locus:3.1.1.211131.56529
Query4 (1831.8177,1+)  : Locus:3.1.1.211131.56525
Query5 (1845.8223,1+)  : Locus:3.1.1.211131.56528
```

Mascot: http://www.matrixscience.com/

Mascot Search Results

**User**	:	
**Email**	:	
**Search title**	:	**F:\ROD_inmunoproteoma\MALDI\160223_2016_561_562_ROD\ppw_G1_145631467808.txt**
**MS data file**	:	**F:\ROD_inmunoproteoma\MALDI\160223_2016_561_562_ROD\ppw_G1_145631467808.txt**
**Database**	:	**Brachyspiraceae Brachyspiraceae_20160127-151122 (40573 sequences; 13470793 residues)**
**Timestamp**	:	**24 Feb 2016 at 12:02:42 GMT**
**Warning**	:	**A Peptide summary report will usually give a much clearer picture of MS/MS search results.**
**Top Score**	:	**210 for D8ICG3, D8ICG3_BRAP9 Uncharacterized protein OS=Brachyspira pilosicoli (strain ATCC BAA-1826 / 95/1000) GN=BP95100**

Probability Based Mowse Score

Protein score is -10*Log(P), where P is the probability that the observed match is a random event.
Protein scores greater than 59 are significant (p<0.05).
Protein scores are derived from ions scores as a non-probabilistic basis for ranking protein hits.

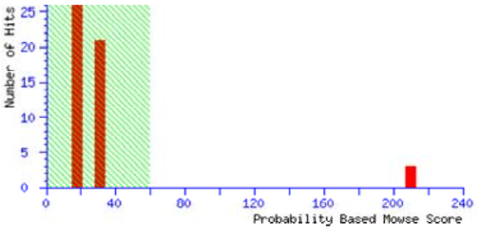

Protein Summary Report

Format As | Protein Summary (deprecated) | Help

Significance threshold p< 0.05 | Max. number of hits AUTO

Standard scoring ◉ MudPIT scoring ○ | Ions score or expect cut-off 0 | Show sub-sets 0

Show pop-ups ◉ Suppress pop-ups ○ | Sort unassigned Decreasing Score | Require bold red ☐

Re-Search All | Search Unmatched

Index

	Accession	Mass	Score	Description
1.	D8ICG3	38964	210	D8ICG3_BRAP9 Uncharacterized protein OS=Brachyspira pilosicoli (strain ATCC BAA-1826 / 95/1000) GN=BP951000_0838
2.	K0JML9	38964	210	K0JML9_BRAPL Uncharacterized protein OS=Brachyspira pilosicoli WesB GN=WESB_2163 PE=4 SV=1
3.	J9UR86	39254	209	J9UR86_BRAPL Uncharacterized protein OS=Brachyspira pilosicoli B2904 GN=B2904_orf530 PE=4 SV=1
4.	D5U842	90003	35	D5U842_BRAM5 Histidine kinase OS=Brachyspira murdochii (strain ATCC 51284 / DSM 12563 / 56-150) GN=Bmur_0766 PE=

Results List

1. D8ICG3 **Mass:** 38964 **Score:** 210 **Expect:** 4.1e-17 **Queries matched:** 3
D8ICG3_BRAP9 Uncharacterized protein OS=Brachyspira pilosicoli (strain ATCC BAA-1826 / 95/1000) GN=BP951000_0838 PE=4 SV=1

Observed	Mr(expt)	Mr(calc)	ppm	Start - End	Miss	Ions	Peptide
1134.4781	1133.4708	1133.4811	-9.10	183 - 192	0	8	K.GSPSYYSMSR.Y
1229.6295	1228.6222	1228.6452	-18.69	276 - 285	1	7	K.VLYTTSDRFK.A
2283.0137	2282.0064	2282.0379	-13.79	291 - 309	0	149	K.YYDELGFVYANTEDEVLSR.D

No match to: 1057.6755, 1086.4725

2. K0JML9 **Mass:** 38964 **Score:** 210 **Expect:** 4.1e-17 **Queries matched:** 3
K0JML9_BRAPL Uncharacterized protein OS=Brachyspira pilosicoli WesB GN=WESB_2163 PE=4 SV=1

Observed	Mr(expt)	Mr(calc)	ppm	Start - End	Miss	Ions	Peptide
1134.4781	1133.4708	1133.4811	-9.10	183 - 192	0	8	K.GSPSYYSMSR.Y
1229.6295	1228.6222	1228.6452	-18.69	276 - 285	1	7	K.VLYTTSDRFK.A
2283.0137	2282.0064	2282.0379	-13.79	291 - 309	0	149	K.YYDELGFVYANTEDEVLSR.D

No match to: 1057.6755, 1086.4725

3. J9UR86 **Mass:** 39254 **Score:** 209 **Expect:** 5.1e-17 **Queries matched:** 3
J9UR86_BRAPL Uncharacterized protein OS=Brachyspira pilosicoli B2904 GN=B2904_orf530 PE=4 SV=1

Observed	Mr(expt)	Mr(calc)	ppm	Start - End	Miss	Ions	Peptide
1134.4781	1133.4708	1133.4811	-9.10	183 - 192	0	8	K.GSPSYYSMSR.Y
1229.6295	1228.6222	1228.6452	-18.69	276 - 285	1	7	K.VLYTTSDRFK.A
2283.0137	2282.0064	2282.0379	-13.79	291 - 309	0	149	K.YYDELGFVYANTEDEVLSR.D

No match to: 1057.6755, 1086.4725

4. D5U842 **Mass:** 90003 **Score:** 35 **Expect:** 12 **Queries matched:** 3
D5U842_BRAM5 Histidine kinase OS=Brachyspira murdochii (strain ATCC 51284 / DSM 12563 / 56-150) GN=Bmur_0766 PE=4 SV=1

Observed	Mr(expt)	Mr(calc)	ppm	Start - End	Miss	Ions	Peptide
1057.6755	1056.6682	1056.6291	37.0	370 - 378	1	---	R.DSIVQVLRK.F
1229.6295	1228.6222	1228.6411	-15.37	287 - 299	1	---	K.AKEAAAPSANTAK.K
2283.0137	2282.0064	2282.0437	-16.35	100 - 120	0	---	K.DLINTASEEGEYSGDIEAEIK.K

No match to: 1086.4725, 1134.4781

Search Parameters

```
Type of search         : Sequence Query
Enzyme                 : Trypsin
Fixed modifications    : Carbamidomethyl (C)
Variable modifications : Oxidation (M)
Mass values            : Monoisotopic
Protein Mass           : Unrestricted
Peptide Mass Tolerance : ± 50 ppm
Fragment Mass Tolerance: ± 0.5 Da
Max Missed Cleavages   : 1
Instrument type        : MALDI-TOF-TOF
Query1 (1057.6755,1+)  : Locus:9.1.1.213508.57280
Query2 (1086.4725,1+)  : Locus:9.1.1.213508.57279
Query3 (1134.4781,1+)  : Locus:9.1.1.213508.57278
Query4 (1229.6295,1+)  : Locus:9.1.1.213508.57277
Query5 (2283.0137,1+)  : Locus:9.1.1.213508.57276
```

Mascot: http://www.matrixscience.com/

MATRIX SCIENCE Mascot Search Results

User	:
Email	:
Search title	: F:\ROD_inmunoproteoma\MALDI\ppw_2014_442_ROD\ppw_G10_139601421409.txt
MS data file	: F:\ROD_inmunoproteoma\MALDI\ppw_2014_442_ROD\ppw_G10_139601421409.txt
Database	: Brachyspiraceae Brachyspiraceae_20160127-151122 (40573 sequences; 13470793 residues)
Timestamp	: 22 Feb 2016 at 15:14:11 GMT
Warning	: A Peptide summary report will usually give a much clearer picture of MS/MS search results.
Top Score	: 236 for J9UWR4, J9UWR4_BRAPL Flagellar filament outer layer protein FlaA OS=Brachyspira pilosicoli B2904 GN=B2904_orf2029

Probability Based Mowse Score

Protein score is -10*Log(P), where P is the probability that the observed match is a random event.
Protein scores greater than 59 are significant (p<0.05).
Protein scores are derived from ions scores as a non-probabilistic basis for ranking protein hits.

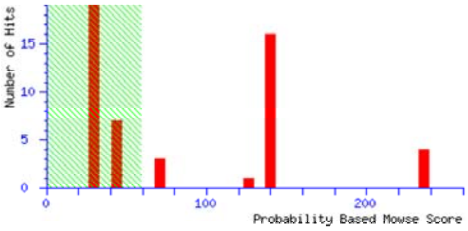

Protein Summary Report

Format As | Protein Summary (deprecated) | Help

Significance threshold p< 0.05 | Max. number of hits AUTO

Standard scoring ◉ MudPIT scoring ○ | Ions score or expect cut-off 0 | Show sub-sets 0

Show pop-ups ◉ Suppress pop-ups ○ | Sort unassigned Decreasing Score | Require bold red ☐

Re-Search All | Search Unmatched

Index

	Accession	Mass	Score	Description
1.	J9UWR4	35678	236	J9UWR4_BRAPL Flagellar filament outer layer protein FlaA OS=Brachyspira pilosicoli B2904 GN=B2904_orf2029 PE=4
2.	D8IBY6	35707	236	D8IBY6_BRAP9 Flagellar filament outer layer protein, FlaA OS=Brachyspira pilosicoli (strain ATCC BAA-1826 / 95
3.	K0JH04	35709	236	K0JH04_BRAPL Flagellar filament outer layer protein FlaA OS=Brachyspira pilosicoli WesB GN=flaA PE=4 SV=1
4.	Q5XPB3	36034	235	Q5XPB3_BRAPL FlaA1 (Fragment) OS=Brachyspira pilosicoli PE=4 SV=1
5.	B7T1P1	24517	137	B7T1P1_9SPIR FlaA (Fragment) OS=Brachyspira sp. SRBP205IR PE=4 SV=1
6.	B7T1P2	24517	137	B7T1P2_9SPIR FlaA (Fragment) OS=Brachyspira sp. SRBP5IR PE=4 SV=1
7.	B7T1P3	24499	137	B7T1P3_9SPIR FlaA (Fragment) OS=Brachyspira innocens PE=4 SV=1
8.	B7T1Q0	24499	137	B7T1Q0_9SPIR FlaA (Fragment) OS=Brachyspira alvinipulli PE=4 SV=1
9.	B7T1P9	24499	137	B7T1P9_9SPIR FlaA (Fragment) OS=Brachyspira intermedia PE=4 SV=1
10.	R5LCF6	35187	134	R5LCF6_9SPIR Flagellar filament outer layer protein FlaA OS=Brachyspira sp. CAG:700 GN=BN758_01892 PE=4 SV=1
11.	B7T1P4	24426	134	B7T1P4_9SPIR FlaA (Fragment) OS=Brachyspira innocens PE=4 SV=1
12.	B7T1P5	24371	134	B7T1P5_9SPIR FlaA (Fragment) OS=Brachyspira innocens PE=4 SV=1
13.	L8XQZ8	35741	133	L8XQZ8_9SPIR Flagellar filament outer layer protein FlaA OS=Brachyspira hampsonii 30599 GN=H263_11839 PE=4 SV=
14.	P32520	35964	133	FLAA1_BRAHO Flagellar filament outer layer protein flaA1 OS=Brachyspira hyodysenteriae GN=flaA1 PE=1 SV=1
15.	A0A0H0TRE1	35964	133	A0A0H0TRE1_BRAHO Flagellar filament protein FlaA OS=Brachyspira hyodysenteriae GN=SR30_03350 PE=4 SV=1
16.	A0A0G4K8P6	35920	133	A0A0G4K8P6_9SPIR Flagellar filament outer layer protein flaA1 OS=Brachyspira suanatina GN=flaA1 PE=4 SV=1
17.	C0R0T5	35964	133	C0R0T5_BRAHW Flagellar filament outer layer protein FlaA OS=Brachyspira hyodysenteriae (strain ATCC 49526 / WA
18.	A0A0H0V489	35934	133	A0A0H0V489_BRAHO Flagellar filament protein FlaA OS=Brachyspira hyodysenteriae GN=SZ48_11445 PE=4 SV=1
19.	A0A0H0WR23	35965	133	A0A0H0WR23_BRAHO Flagellar filament protein FlaA OS=Brachyspira hyodysenteriae GN=SZ42_05145 PE=4 SV=1
20.	G0EJW8	35935	133	G0EJW8_BRAIP Flagellar filament outer layer protein FlaA OS=Brachyspira intermedia (strain ATCC 51140 / PWS/A)
21.	D5U8F3	35700	130	D5U8F3_BRAM5 Flagellar filament outer layer protein FlaA OS=Brachyspira murdochii (strain ATCC 51284 / DSM 125
22.	B7T1P7	24514	77	B7T1P7_9SPIR FlaA (Fragment) OS=Brachyspira canis PE=4 SV=1
23.	B7T1P6	24483	77	B7T1P6_9SPIR FlaA (Fragment) OS=Brachyspira intermedia PE=4 SV=1
24.	L0X4M2	36070	75	L0X4M2_9SPIR Flagellar filament outer layer protein FlaA OS=Brachyspira hampsonii 30446 GN=A966_04095 PE=4 SV=
25.	R5H3G1	12215	41	R5H3G1_9SPIR Uncharacterized protein OS=Brachyspira sp. CAG:484 GN=BN676_00850 PE=4 SV=1

Results List

1. J9UWR4 **Mass:** 35678 **Score:** 236 **Expect:** 1e-19 **Queries matched:** 3
J9UWR4_BRAPL Flagellar filament outer layer protein FlaA OS=Brachyspira pilosicoli B2904 GN=B2904_orf2029 PE=4 SV=1

Observed	Mr(expt)	Mr(calc)	ppm	Start - End	Miss	Ions	Peptide
970.5158	969.5085	969.4920	17.0	217 - 224	0	62	K.LDSLGFYR.T
1569.8662	1568.8589	1568.8270	20.3	279 - 291	1	79	R.VREAAELQQLEQR.R
1625.8986	1624.8913	1624.8647	16.4	211 - 224	1	40	K.MIPSVKLDSLGFYR.T

2. D8IBY6 **Mass:** 35707 **Score:** 236 **Expect:** 1e-19 **Queries matched:** 3
D8IBY6_BRAP9 Flagellar filament outer layer protein, FlaA OS=Brachyspira pilosicoli (strain ATCC BAA-1826 / 95/1000) GN=flaA PE=4 SV=1

Observed	Mr(expt)	Mr(calc)	ppm	Start - End	Miss	Ions	Peptide
970.5158	969.5085	969.4920	17.0	217 - 224	0	62	K.LDSLGFYR.T
1569.8662	1568.8589	1568.8270	20.3	279 - 291	1	79	R.VREAAELQQLEQR.R
1625.8986	1624.8913	1624.8647	16.4	211 - 224	1	40	K.MIPSVKLDSLGFYR.T

3. K0JH04 **Mass:** 35709 **Score:** 236 **Expect:** 1e-19 **Queries matched:** 3
K0JH04_BRAPL Flagellar filament outer layer protein FlaA OS=Brachyspira pilosicoli WesB GN=flaA PE=4 SV=1

Observed	Mr(expt)	Mr(calc)	ppm	Start	End	Miss	Ions	Peptide
970.5158	969.5085	969.4920	17.0	217 -	224	0	62	K.LDSLGFYR.T
1569.8662	1568.8589	1568.8270	20.3	279 -	291	1	79	R.VREAAELQQLEQR.R
1625.8986	1624.8913	1624.8647	16.4	211 -	224	1	40	K.MIPSVKLDSLGFYR.T

4. Q5XPB3 **Mass:** 36034 **Score:** 235 **Expect:** 1.3e-19 **Queries matched:** 3
Q5XPB3_BRAPL FlaA1 (Fragment) OS=Brachyspira pilosicoli PE=4 SV=1

Observed	Mr(expt)	Mr(calc)	ppm	Start	End	Miss	Ions	Peptide
970.5158	969.5085	969.4920	17.0	217 -	224	0	62	K.LDSLGFYR.T
1569.8662	1568.8589	1568.8270	20.3	279 -	291	1	79	R.VREAAELQQLEQR.R
1625.8986	1624.8913	1624.8647	16.4	211 -	224	1	40	K.MIPSVKLDSLGFYR.T

5. B7T1P1 **Mass:** 24517 **Score:** 137 **Expect:** 8.1e-10 **Queries matched:** 2
B7T1P1_9SPIR FlaA (Fragment) OS=Brachyspira sp. SRBP205IR PE=4 SV=1

Observed	Mr(expt)	Mr(calc)	ppm	Start	End	Miss	Ions	Peptide
970.5158	969.5085	969.4920	17.0	186 -	193	0	62	K.LDSLGFYR.T
1625.8986	1624.8913	1624.8647	16.4	180 -	193	1	40	K.MIPSVKLDSLGFYR.T

No match to: 1569.8662

6. B7T1P2 **Mass:** 24517 **Score:** 137 **Expect:** 8.1e-10 **Queries matched:** 2
B7T1P2_9SPIR FlaA (Fragment) OS=Brachyspira sp. SRBP5IR PE=4 SV=1

Observed	Mr(expt)	Mr(calc)	ppm	Start	End	Miss	Ions	Peptide
970.5158	969.5085	969.4920	17.0	186 -	193	0	62	K.LDSLGFYR.T
1625.8986	1624.8913	1624.8647	16.4	180 -	193	1	40	K.MIPSVKLDSLGFYR.T

No match to: 1569.8662

7. B7T1P3 **Mass:** 24499 **Score:** 137 **Expect:** 8.1e-10 **Queries matched:** 2
B7T1P3_9SPIR FlaA (Fragment) OS=Brachyspira innocens PE=4 SV=1

Observed	Mr(expt)	Mr(calc)	ppm	Start	End	Miss	Ions	Peptide
970.5158	969.5085	969.4920	17.0	186 -	193	0	62	K.LDSLGFYR.T
1625.8986	1624.8913	1624.8647	16.4	180 -	193	1	40	R.MIPSVKLDSLGFYR.T

No match to: 1569.8662

8. B7T1Q0 **Mass:** 24499 **Score:** 137 **Expect:** 8.1e-10 **Queries matched:** 2
B7T1Q0_9SPIR FlaA (Fragment) OS=Brachyspira alvinipulli PE=4 SV=1

Observed	Mr(expt)	Mr(calc)	ppm	Start	End	Miss	Ions	Peptide
970.5158	969.5085	969.4920	17.0	186 -	193	0	62	K.LDSLGFYR.T
1625.8986	1624.8913	1624.8647	16.4	180 -	193	1	40	R.MIPSVKLDSLGFYR.T

No match to: 1569.8662

9. B7T1P9 **Mass:** 24499 **Score:** 137 **Expect:** 8.1e-10 **Queries matched:** 2
B7T1P9_9SPIR FlaA (Fragment) OS=Brachyspira intermedia PE=4 SV=1

Observed	Mr(expt)	Mr(calc)	ppm	Start	End	Miss	Ions	Peptide
970.5158	969.5085	969.4920	17.0	186 -	193	0	62	K.LDSLGFYR.T
1625.8986	1624.8913	1624.8647	16.4	180 -	193	1	40	R.MIPSVKLDSLGFYR.T

No match to: 1569.8662

10. R5LCF6 **Mass:** 35187 **Score:** 134 **Expect:** 1.6e-09 **Queries matched:** 2
R5LCF6_9SPIR Flagellar filament outer layer protein FlaA OS=Brachyspira sp. CAG:700 GN=BN758_01892 PE=4 SV=1

Observed	Mr(expt)	Mr(calc)	ppm	Start	End	Miss	Ions	Peptide
970.5158	969.5085	969.4920	17.0	217 -	224	0	62	K.IDSIGFYR.T
1625.8986	1624.8913	1624.8647	16.4	211 -	224	1	40	K.LMPSVKIDSIGFYR.T

No match to: 1569.8662

11. B7T1P4 **Mass:** 24426 **Score:** 134 **Expect:** 1.6e-09 **Queries matched:** 2
B7T1P4_9SPIR FlaA (Fragment) OS=Brachyspira innocens PE=4 SV=1

Observed	Mr(expt)	Mr(calc)	ppm	Start	End	Miss	Ions	Peptide
970.5158	969.5085	969.4920	17.0	186 -	193	0	62	K.LDSLGFYR.T
1625.8986	1624.8913	1624.8647	16.4	180 -	193	1	36	R.MVPSIKLDSLGFYR.T

No match to: 1569.8662

12. B7T1P5 **Mass:** 24371 **Score:** 134 **Expect:** 1.6e-09 **Queries matched:** 2
B7T1P5_9SPIR FlaA (Fragment) OS=Brachyspira innocens PE=4 SV=1

Observed	Mr(expt)	Mr(calc)	ppm	Start	End	Miss	Ions	Peptide
970.5158	969.5085	969.4920	17.0	186 -	193	0	62	K.LDSLGFYR.T
1625.8986	1624.8913	1624.8647	16.4	180 -	193	1	36	K.MVPSIKLDSLGFYR.T

No match to: 1569.8662

13. L8XQZ8 **Mass:** 35741 **Score:** 133 **Expect:** 2e-09 **Queries matched:** 2
L8XQZ8_9SPIR Flagellar filament outer layer protein FlaA OS=Brachyspira hampsonii 30599 GN=H263_11839 PE=4 SV=1

Observed	Mr(expt)	Mr(calc)	ppm	Start	End	Miss	Ions	Peptide
970.5158	969.5085	969.4920	17.0	217 -	224	0	62	K.LDSLGFYR.T
1625.8986	1624.8913	1624.8647	16.4	211 -	224	1	40	R.MIPSVKLDSLGFYR.T

No match to: 1569.8662

14. P32520 **Mass:** 35964 **Score:** 133 **Expect:** 2e-09 **Queries matched:** 2
FLAA1_BRAHO Flagellar filament outer layer protein flaA1 OS=Brachyspira hyodysenteriae GN=flaA1 PE=1 SV=1

Observed	Mr(expt)	Mr(calc)	ppm	Start	End	Miss	Ions	Peptide
970.5158	969.5085	969.4920	17.0	217 -	224	0	62	K.LDSLGFYR.T
1625.8986	1624.8913	1624.8647	16.4	211 -	224	1	40	R.MIPSVKLDSLGFYR.T

No match to: 1569.8662

15. A0A0H0TRE1 **Mass:** 35964 **Score:** 133 **Expect:** 2e-09 **Queries matched:** 2
A0A0H0TRE1_BRAHO Flagellar filament protein FlaA OS=Brachyspira hyodysenteriae GN=SR30_03350 PE=4 SV=1

Observed	Mr(expt)	Mr(calc)	ppm	Start	End	Miss	Ions	Peptide
970.5158	969.5085	969.4920	17.0	217 -	224	0	62	K.LDSLGFYR.T
1625.8986	1624.8913	1624.8647	16.4	211 -	224	1	40	R.MIPSVKLDSLGFYR.T

No match to: 1569.8662

16. A0A0G4K8P6 **Mass:** 35920 **Score:** 133 **Expect:** 2e-09 **Queries matched:** 2
A0A0G4K8P6_9SPIR Flagellar filament outer layer protein flaA1 OS=Brachyspira suanatina GN=flaA1 PE=4 SV=1

Observed	Mr(expt)	Mr(calc)	ppm	Start		End	Miss	Ions	Peptide
970.5158	969.5085	969.4920	17.0	217	-	224	0	62	K.LDSLGFYR.T
1625.8986	1624.8913	1624.8647	16.4	211	-	224	1	40	R.MIPSVKLDSLGFYR.T

No match to: 1569.8662

17. C0R0T5 **Mass:** 35964 **Score:** 133 **Expect:** 2e-09 **Queries matched:** 2
C0R0T5_BRAHW Flagellar filament outer layer protein FlaA OS=Brachyspira hyodysenteriae (strain ATCC 49526 / WA1) GN=flaA PE=4 SV=1

Observed	Mr(expt)	Mr(calc)	ppm	Start		End	Miss	Ions	Peptide
970.5158	969.5085	969.4920	17.0	217	-	224	0	62	K.LDSLGFYR.T
1625.8986	1624.8913	1624.8647	16.4	211	-	224	1	40	R.MIPSVKLDSLGFYR.T

No match to: 1569.8662

18. A0A0H0V489 **Mass:** 35934 **Score:** 133 **Expect:** 2e-09 **Queries matched:** 2
A0A0H0V489_BRAHO Flagellar filament protein FlaA OS=Brachyspira hyodysenteriae GN=SZ48_11445 PE=4 SV=1

Observed	Mr(expt)	Mr(calc)	ppm	Start		End	Miss	Ions	Peptide
970.5158	969.5085	969.4920	17.0	217	-	224	0	62	K.LDSLGFYR.T
1625.8986	1624.8913	1624.8647	16.4	211	-	224	1	40	R.MIPSVKLDSLGFYR.T

No match to: 1569.8662

19. A0A0H0WR23 **Mass:** 35965 **Score:** 133 **Expect:** 2e-09 **Queries matched:** 2
A0A0H0WR23_BRAHO Flagellar filament protein FlaA OS=Brachyspira hyodysenteriae GN=SZ42_05145 PE=4 SV=1

Observed	Mr(expt)	Mr(calc)	ppm	Start		End	Miss	Ions	Peptide
970.5158	969.5085	969.4920	17.0	217	-	224	0	62	K.LDSLGFYR.T
1625.8986	1624.8913	1624.8647	16.4	211	-	224	1	40	R.MIPSVKLDSLGFYR.T

No match to: 1569.8662

20. G0EJW8 **Mass:** 35935 **Score:** 133 **Expect:** 2e-09 **Queries matched:** 2
G0EJW8_BRAIP Flagellar filament outer layer protein FlaA OS=Brachyspira intermedia (strain ATCC 51140 / PWS/A) GN=Bint_0608 PE=4 SV=1

Observed	Mr(expt)	Mr(calc)	ppm	Start		End	Miss	Ions	Peptide
970.5158	969.5085	969.4920	17.0	217	-	224	0	62	K.LDSLGFYR.T
1625.8986	1624.8913	1624.8647	16.4	211	-	224	1	40	R.MIPSVKLDSLGFYR.T

No match to: 1569.8662

21. D5U8F3 **Mass:** 35700 **Score:** 130 **Expect:** 4.1e-09 **Queries matched:** 2
D5U8F3_BRAM5 Flagellar filament outer layer protein FlaA OS=Brachyspira murdochii (strain ATCC 51284 / DSM 12563 / 56-150) GN=Bmur_087

Observed	Mr(expt)	Mr(calc)	ppm	Start		End	Miss	Ions	Peptide
970.5158	969.5085	969.4920	17.0	217	-	224	0	62	K.LDSLGFYR.T
1625.8986	1624.8913	1624.8647	16.4	211	-	224	1	36	R.MVPSIKLDSLGFYR.T

No match to: 1569.8662

22. B7T1P7 **Mass:** 24514 **Score:** 77 **Expect:** 0.00087 **Queries matched:** 1
B7T1P7_9SPIR FlaA (Fragment) OS=Brachyspira canis PE=4 SV=1

Observed	Mr(expt)	Mr(calc)	ppm	Start		End	Miss	Ions	Peptide
970.5158	969.5085	969.4920	17.0	186	-	193	0	62	K.LDSLGFYR.T

No match to: 1569.8662, 1625.8986

23. B7T1P6 **Mass:** 24483 **Score:** 77 **Expect:** 0.00087 **Queries matched:** 1
B7T1P6_9SPIR FlaA (Fragment) OS=Brachyspira intermedia PE=4 SV=1

Observed	Mr(expt)	Mr(calc)	ppm	Start		End	Miss	Ions	Peptide
970.5158	969.5085	969.4920	17.0	186	-	193	0	62	K.LDSLGFYR.T

No match to: 1569.8662, 1625.8986

24. L0X4M2 **Mass:** 36070 **Score:** 75 **Expect:** 0.0013 **Queries matched:** 1
L0X4M2_9SPIR Flagellar filament outer layer protein FlaA OS=Brachyspira hampsonii 30446 GN=A966_04095 PE=4 SV=1

Observed	Mr(expt)	Mr(calc)	ppm	Start		End	Miss	Ions	Peptide
970.5158	969.5085	969.4920	17.0	217	-	224	0	62	K.LDSLGFYR.T

No match to: 1569.8662, 1625.8986

25. R5H3G1 **Mass:** 12215 **Score:** 41 **Expect:** 3.5 **Queries matched:** 2
R5H3G1_9SPIR Uncharacterized protein OS=Brachyspira sp. CAG:484 GN=BN676_00850 PE=4 SV=1

Observed	Mr(expt)	Mr(calc)	ppm	Start		End	Miss	Ions	Peptide
970.5158	969.5085	969.4841	25.1	1	-	8	0	---	-.MILSEFSK.F + Oxidation (M)
1569.8662	1568.8589	1568.8100	31.2	93	-	104	1	---	R.WLQDKKPHDFQK.-

No match to: 1625.8986

Search Parameters

Type of search : Sequence Query
Enzyme : Trypsin
Fixed modifications : Carbamidomethyl (C)
Variable modifications : Oxidation (M)
Mass values : Monoisotopic
Protein Mass : Unrestricted
Peptide Mass Tolerance : ± 50 ppm
Fragment Mass Tolerance: ± 0.5 Da
Max Missed Cleavages : 1
Instrument type : MALDI-TOF-TOF
Query1 (970.5158,1+) : Locus:10.1.1.170608.52764
Query2 (1569.8662,1+) : Locus:10.1.1.170608.52762
Query3 (1625.8986,1+) : Locus:10.1.1.170608.52763

Mascot: http://www.matrixscience.com/

Mascot Search Results

User	:
Email	:
Search title	: F:\ROD_inmunoproteoma\MALDI\ppw_2014_442_ROD\ppw_G18_139601421617.txt
MS data file	: F:\ROD_inmunoproteoma\MALDI\ppw_2014_442_ROD\ppw_G18_139601421617.txt
Database	: Brachyspiraceae Brachyspiraceae_20160127-151122 (40573 sequences; 13470793 residues)
Timestamp	: 22 Feb 2016 at 15:14:31 GMT
Warning	: A Peptide summary report will usually give a much clearer picture of MS/MS search results.
Top Score	: 210 for J9UWR4, J9UWR4_BRAPL Flagellar filament outer layer protein FlaA OS=Brachyspira pilosicoli B2904 GN=B2904_orf2029

Probability Based Mowse Score

Protein score is -10*Log(P), where P is the probability that the observed match is a random event.
Protein scores greater than 59 are significant (p<0.05).
Protein scores are derived from ions scores as a non-probabilistic basis for ranking protein hits.

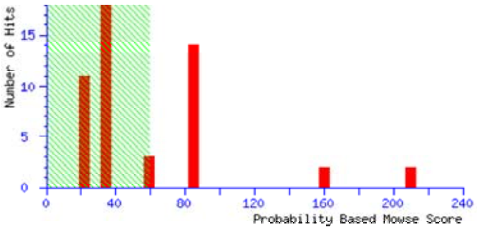

Protein Summary Report

Format As [Protein Summary (deprecated)] Help

Significance threshold p< [0.05] Max. number of hits [AUTO]

Standard scoring ◉ MudPIT scoring ○ Ions score or expect cut-off [0] Show sub-sets [0]

Show pop-ups ◉ Suppress pop-ups ○ Sort unassigned [Decreasing Score] Require bold red ☐

[Re-Search All] [Search Unmatched]

Index

	Accession	Mass	Score	Description
1.	J9UWR4	35678	210	J9UWR4_BRAPL Flagellar filament outer layer protein FlaA OS=Brachyspira pilosicoli B2904 GN=B2904_orf2029 PE=4
2.	D8IBY6	35707	210	D8IBY6_BRAP9 Flagellar filament outer layer protein, FlaA OS=Brachyspira pilosicoli (strain ATCC BAA-1826 / 95
3.	K0JH04	35709	161	K0JH04_BRAPL Flagellar filament outer layer protein FlaA OS=Brachyspira pilosicoli WesB GN=flaA PE=4 SV=1
4.	Q5XPB3	36034	161	Q5XPB3_BRAPL FlaA1 (Fragment) OS=Brachyspira pilosicoli PE=4 SV=1
5.	B7T1P1	24517	86	B7T1P1_9SPIR FlaA (Fragment) OS=Brachyspira sp. SRBP205IR PE=4 SV=1
6.	B7T1P2	24517	86	B7T1P2_9SPIR FlaA (Fragment) OS=Brachyspira sp. SRBP5IR PE=4 SV=1
7.	B7T1P3	24499	86	B7T1P3_9SPIR FlaA (Fragment) OS=Brachyspira innocens PE=4 SV=1
8.	B7T1Q0	24499	86	B7T1Q0_9SPIR FlaA (Fragment) OS=Brachyspira alvinipulli PE=4 SV=1
9.	B7T1P9	24499	86	B7T1P9_9SPIR FlaA (Fragment) OS=Brachyspira intermedia PE=4 SV=1
10.	R5LCF6	35187	84	R5LCF6_9SPIR Flagellar filament outer layer protein FlaA OS=Brachyspira sp. CAG:700 GN=BN758_01892 PE=4 SV=1
11.	L8XQZ8	35741	84	L8XQZ8_9SPIR Flagellar filament outer layer protein FlaA OS=Brachyspira hampsonii 30599 GN=H263_11839 PE=4 SV=
12.	P32520	35964	84	FLAA1_BRAHO Flagellar filament outer layer protein flaA1 OS=Brachyspira hyodysenteriae GN=flaA1 PE=1 SV=1
13.	A0A0H0TRE1	35964	84	A0A0H0TRE1_BRAHO Flagellar filament protein FlaA OS=Brachyspira hyodysenteriae GN=SR30_03350 PE=4 SV=1
14.	A0A0G4K8P6	35920	84	A0A0G4K8P6_9SPIR Flagellar filament outer layer protein flaA1 OS=Brachyspira suanatina GN=flaA1 PE=4 SV=1
15.	C0R0T5	35964	84	C0R0T5_BRAHW Flagellar filament outer layer protein FlaA OS=Brachyspira hyodysenteriae (strain ATCC 49526 / WA
16.	A0A0H0V489	35934	84	A0A0H0V489_BRAHO Flagellar filament protein FlaA OS=Brachyspira hyodysenteriae GN=SZ48_11445 PE=4 SV=1
17.	A0A0H0WR23	35965	84	A0A0H0WR23_BRAHO Flagellar filament protein FlaA OS=Brachyspira hyodysenteriae GN=SZ42_05145 PE=4 SV=1
18.	G0EJW8	35935	84	G0EJW8_BRAIP Flagellar filament outer layer protein FlaA OS=Brachyspira intermedia (strain ATCC 51140 / PWS/A)
19.	B7T1P4	24426	65	B7T1P4_9SPIR FlaA (Fragment) OS=Brachyspira innocens PE=4 SV=1
20.	B7T1P5	24371	65	B7T1P5_9SPIR FlaA (Fragment) OS=Brachyspira innocens PE=4 SV=1
21.	D5U8F3	35700	63	D5U8F3_BRAM5 Flagellar filament outer layer protein FlaA OS=Brachyspira murdochii (strain ATCC 51284 / DSM 125
22.	J9USH8	28003	36	J9USH8_BRAPL GntR family transcriptional regulator OS=Brachyspira pilosicoli B2904 GN=gntR PE=4 SV=1

Results List

1. J9UWR4 **Mass:** 35678 **Score:** 210 **Expect:** 4.1e-17 **Queries matched:** 3
J9UWR4_BRAPL Flagellar filament outer layer protein FlaA OS=Brachyspira pilosicoli B2904 GN=B2904_orf2029 PE=4 SV=1

Observed	Mr(expt)	Mr(calc)	ppm	Start		End	Miss	Ions	Peptide
1569.8810	1568.8737	1568.8270	29.8	279	-	291	1	59	R.VREAAELQQLEQR.R
1625.9152	1624.9079	1624.8647	26.6	211	-	224	1	70	K.MIPSVKLDSLGFYR.T
2544.2473	2543.2400	2543.1596	31.6	292	-	316	1	22	R.RINGGDAQQDAGAAQNTQTQETAAQ.-

2. D8IBY6 **Mass:** 35707 **Score:** 210 **Expect:** 4.1e-17 **Queries matched:** 3
D8IBY6_BRAP9 Flagellar filament outer layer protein, FlaA OS=Brachyspira pilosicoli (strain ATCC BAA-1826 / 95/1000) GN=flaA PE=4 SV=1

Observed	Mr(expt)	Mr(calc)	ppm	Start		End	Miss	Ions	Peptide
1569.8810	1568.8737	1568.8270	29.8	279	-	291	1	59	R.VREAAELQQLEQR.R
1625.9152	1624.9079	1624.8647	26.6	211	-	224	1	70	K.MIPSVKLDSLGFYR.T
2544.2473	2543.2400	2543.1596	31.6	292	-	316	1	22	R.RINGGDAQQDAGAAQNTQTQETAAQ.-

3. K0JH04 **Mass:** 35709 **Score:** 161 **Expect:** 3.2e-12 **Queries matched:** 2
K0JH04_BRAPL Flagellar filament outer layer protein FlaA OS=Brachyspira pilosicoli WesB GN=flaA PE=4 SV=1

Observed	Mr(expt)	Mr(calc)	ppm	Start		End	Miss	Ions	Peptide
1569.8810	1568.8737	1568.8270	29.8	279	-	291	1	59	R.VREAAELQQLEQR.R
1625.9152	1624.9079	1624.8647	26.6	211	-	224	1	70	K.MIPSVKLDSLGFYR.T

No match to: 2544.2473

4. Q5XPB3 **Mass:** 36034 **Score:** 161 **Expect:** 3.2e-12 **Queries matched:** 2
Q5XPB3_BRAPL FlaA1 (Fragment) OS=Brachyspira pilosicoli PE=4 SV=1

Observed	Mr(expt)	Mr(calc)	ppm	Start	End	Miss	Ions	Peptide
1569.8810	1568.8737	1568.8270	29.8	279 -	291	1	59	R.VREAAELQQLEQR.R
1625.9152	1624.9079	1624.8647	26.6	211 -	224	1	70	K.MIPSVKLDSLGFYR.T

No match to: 2544.2473

5. B7T1P1 **Mass:** 24517 **Score:** 86 **Expect:** 0.00011 **Queries matched:** 1
B7T1P1_9SPIR FlaA (Fragment) OS=Brachyspira sp. SRBP205IR PE=4 SV=1

Observed	Mr(expt)	Mr(calc)	ppm	Start	End	Miss	Ions	Peptide
1625.9152	1624.9079	1624.8647	26.6	180 -	193	1	70	K.MIPSVKLDSLGFYR.T

No match to: 1569.8810, 2544.2473

6. B7T1P2 **Mass:** 24517 **Score:** 86 **Expect:** 0.00011 **Queries matched:** 1
B7T1P2_9SPIR FlaA (Fragment) OS=Brachyspira sp. SRBP5IR PE=4 SV=1

Observed	Mr(expt)	Mr(calc)	ppm	Start	End	Miss	Ions	Peptide
1625.9152	1624.9079	1624.8647	26.6	180 -	193	1	70	K.MIPSVKLDSLGFYR.T

No match to: 1569.8810, 2544.2473

7. B7T1P3 **Mass:** 24499 **Score:** 86 **Expect:** 0.00011 **Queries matched:** 1
B7T1P3_9SPIR FlaA (Fragment) OS=Brachyspira innocens PE=4 SV=1

Observed	Mr(expt)	Mr(calc)	ppm	Start	End	Miss	Ions	Peptide
1625.9152	1624.9079	1624.8647	26.6	180 -	193	1	70	R.MIPSVKLDSLGFYR.T

No match to: 1569.8810, 2544.2473

8. B7T1Q0 **Mass:** 24499 **Score:** 86 **Expect:** 0.00011 **Queries matched:** 1
B7T1Q0_9SPIR FlaA (Fragment) OS=Brachyspira alvinipulli PE=4 SV=1

Observed	Mr(expt)	Mr(calc)	ppm	Start	End	Miss	Ions	Peptide
1625.9152	1624.9079	1624.8647	26.6	180 -	193	1	70	R.MIPSVKLDSLGFYR.T

No match to: 1569.8810, 2544.2473

9. B7T1P9 **Mass:** 24499 **Score:** 86 **Expect:** 0.00011 **Queries matched:** 1
B7T1P9_9SPIR FlaA (Fragment) OS=Brachyspira intermedia PE=4 SV=1

Observed	Mr(expt)	Mr(calc)	ppm	Start	End	Miss	Ions	Peptide
1625.9152	1624.9079	1624.8647	26.6	180 -	193	1	70	R.MIPSVKLDSLGFYR.T

No match to: 1569.8810, 2544.2473

10. R5LCF6 **Mass:** 35187 **Score:** 84 **Expect:** 0.00015 **Queries matched:** 1
R5LCF6_9SPIR Flagellar filament outer layer protein FlaA OS=Brachyspira sp. CAG:700 GN=BN758_01892 PE=4 SV=1

Observed	Mr(expt)	Mr(calc)	ppm	Start	End	Miss	Ions	Peptide
1625.9152	1624.9079	1624.8647	26.6	211 -	224	1	70	K.LMPSVKIDSIGFYR.T

No match to: 1569.8810, 2544.2473

11. L8XQZ8 **Mass:** 35741 **Score:** 84 **Expect:** 0.00016 **Queries matched:** 1
L8XQZ8_9SPIR Flagellar filament outer layer protein FlaA OS=Brachyspira hampsonii 30599 GN=H263_11839 PE=4 SV=1

Observed	Mr(expt)	Mr(calc)	ppm	Start	End	Miss	Ions	Peptide
1625.9152	1624.9079	1624.8647	26.6	211 -	224	1	70	R.MIPSVKLDSLGFYR.T

No match to: 1569.8810, 2544.2473

12. P32520 **Mass:** 35964 **Score:** 84 **Expect:** 0.00016 **Queries matched:** 1
FLAA1_BRAHO Flagellar filament outer layer protein flaA1 OS=Brachyspira hyodysenteriae GN=flaA1 PE=1 SV=1

Observed	Mr(expt)	Mr(calc)	ppm	Start	End	Miss	Ions	Peptide
1625.9152	1624.9079	1624.8647	26.6	211 -	224	1	70	R.MIPSVKLDSLGFYR.T

No match to: 1569.8810, 2544.2473

13. A0A0H0TRE1 **Mass:** 35964 **Score:** 84 **Expect:** 0.00016 **Queries matched:** 1
A0A0H0TRE1_BRAHO Flagellar filament protein FlaA OS=Brachyspira hyodysenteriae GN=SR30_03350 PE=4 SV=1

Observed	Mr(expt)	Mr(calc)	ppm	Start	End	Miss	Ions	Peptide
1625.9152	1624.9079	1624.8647	26.6	211 -	224	1	70	R.MIPSVKLDSLGFYR.T

No match to: 1569.8810, 2544.2473

14. A0A0G4K8P6 **Mass:** 35920 **Score:** 84 **Expect:** 0.00016 **Queries matched:** 1
A0A0G4K8P6_9SPIR Flagellar filament outer layer protein flaA1 OS=Brachyspira suanatina GN=flaA1 PE=4 SV=1

Observed	Mr(expt)	Mr(calc)	ppm	Start	End	Miss	Ions	Peptide
1625.9152	1624.9079	1624.8647	26.6	211 -	224	1	70	R.MIPSVKLDSLGFYR.T

No match to: 1569.8810, 2544.2473

15. C0R0T5 **Mass:** 35964 **Score:** 84 **Expect:** 0.00016 **Queries matched:** 1
C0R0T5_BRAHW Flagellar filament outer layer protein FlaA OS=Brachyspira hyodysenteriae (strain ATCC 49526 / WA1) GN=flaA PE=4 SV=1

Observed	Mr(expt)	Mr(calc)	ppm	Start	End	Miss	Ions	Peptide
1625.9152	1624.9079	1624.8647	26.6	211 -	224	1	70	R.MIPSVKLDSLGFYR.T

No match to: 1569.8810, 2544.2473

16. A0A0H0V489 **Mass:** 35934 **Score:** 84 **Expect:** 0.00016 **Queries matched:** 1
A0A0H0V489_BRAHO Flagellar filament protein FlaA OS=Brachyspira hyodysenteriae GN=SZ48_11445 PE=4 SV=1

Observed	Mr(expt)	Mr(calc)	ppm	Start	End	Miss	Ions	Peptide
1625.9152	1624.9079	1624.8647	26.6	211 -	224	1	70	R.MIPSVKLDSLGFYR.T

No match to: 1569.8810, 2544.2473

17. A0A0H0WR23 **Mass:** 35965 **Score:** 84 **Expect:** 0.00016 **Queries matched:** 1
A0A0H0WR23_BRAHO Flagellar filament protein FlaA OS=Brachyspira hyodysenteriae GN=SZ42_05145 PE=4 SV=1

Observed	Mr(expt)	Mr(calc)	ppm	Start	End	Miss	Ions	Peptide
1625.9152	1624.9079	1624.8647	26.6	211 -	224	1	70	R.MIPSVKLDSLGFYR.T

No match to: 1569.8810, 2544.2473

18. G0EJW8 **Mass:** 35935 **Score:** 84 **Expect:** 0.00016 **Queries matched:** 1
G0EJW8_BRAIP Flagellar filament outer layer protein FlaA OS=Brachyspira intermedia (strain ATCC 51140 / PWS/A) GN=Bint_0608 PE=4 SV=1

Observed	Mr(expt)	Mr(calc)	ppm	Start	End	Miss	Ions	Peptide
1625.9152	1624.9079	1624.8647	26.6	211 -	224	1	70	R.MIPSVKLDSLGFYR.T

No match to: 1569.8810, 2544.2473

19. B7T1P4 **Mass:** 24426 **Score:** 65 **Expect:** 0.013 **Queries matched:** 1
B7T1P4_9SPIR FlaA (Fragment) OS=Brachyspira innocens PE=4 SV=1

Observed	Mr(expt)	Mr(calc)	ppm	Start	End	Miss	Ions	Peptide
1625.9152	1624.9079	1624.8647	26.6	180 -	193	1	49	R.MVPSIKLDSLGFYR.T

No match to: 1569.8810, 2544.2473

20. B7T1P5 **Mass:** 24371 **Score:** 65 **Expect:** 0.013 **Queries matched:** 1
B7T1P5_9SPIR FlaA (Fragment) OS=Brachyspira innocens PE=4 SV=1

Observed	Mr(expt)	Mr(calc)	ppm	Start	End	Miss	Ions	Peptide
1625.9152	1624.9079	1624.8647	26.6	180 -	193	1	49	K.MVPSIKLDSLGFYR.T

No match to: 1569.8810, 2544.2473

21. D5U8F3 **Mass:** 35700 **Score:** 63 **Expect:** 0.019 **Queries matched:** 1
D5U8F3_BRAM5 Flagellar filament outer layer protein FlaA OS=Brachyspira murdochii (strain ATCC 51284 / DSM 12563 / 56-150) GN=Bmur_087

Observed	Mr(expt)	Mr(calc)	ppm	Start	End	Miss	Ions	Peptide
1625.9152	1624.9079	1624.8647	26.6	211 -	224	1	49	R.MVPSIKLDSLGFYR.T

No match to: 1569.8810, 2544.2473

22. J9USH8 **Mass:** 28003 **Score:** 36 **Expect:** 11 **Queries matched:** 2
J9USH8_BRAPL GntR family transcriptional regulator OS=Brachyspira pilosicoli B2904 GN=gntR PE=4 SV=1

Observed	Mr(expt)	Mr(calc)	ppm	Start	End	Miss	Ions	Peptide
1569.8810	1568.8737	1568.9250	-32.67	50 -	63	1	---	K.IVSEVLSISRTPIR.E
1625.9152	1624.9079	1624.8896	11.3	4 -	17	1	0	K.LKNNNADNLIILDR.E

No match to: 2544.2473

Search Parameters

Type of search : Sequence Query
Enzyme : Trypsin
Fixed modifications : Carbamidomethyl (C)
Variable modifications : Oxidation (M)
Mass values : Monoisotopic
Protein Mass : Unrestricted
Peptide Mass Tolerance : ± 50 ppm
Fragment Mass Tolerance: ± 0.5 Da
Max Missed Cleavages : 1
Instrument type : MALDI-TOF-TOF
Query1 (1569.8810,1+) : Locus:18.1.1.170616.52786
Query2 (1625.9152,1+) : Locus:18.1.1.170616.52787
Query3 (2544.2473,1+) : Locus:18.1.1.170616.52788

Mascot: http://www.matrixscience.com/

MATRIX SCIENCE Mascot Search Results

User :
Email :
Search title : F:\ROD_inmunoproteoma\MALDI\160223_2016_561_562_ROD\ppw_F3_145631467402.txt
MS data file : F:\ROD_inmunoproteoma\MALDI\160223_2016_561_562_ROD\ppw_F3_145631467402.txt
Database : Brachyspiraceae Brachyspiraceae_20160127-151122 (40573 sequences; 13470793 residues)
Timestamp : 24 Feb 2016 at 12:02:27 GMT
Warning : A Peptide summary report will usually give a much clearer picture of MS/MS search results.
Top Score : 183 for J9UWR4, J9UWR4_BRAPL Flagellar filament outer layer protein FlaA OS=Brachyspira pilosicoli B2904 GN=B2904_orf2029

Probability Based Mowse Score

Protein score is -10*Log(P), where P is the probability that the observed match is a random event.
Protein scores greater than 59 are significant (p<0.05).
Protein scores are derived from ions scores as a non-probabilistic basis for ranking protein hits.

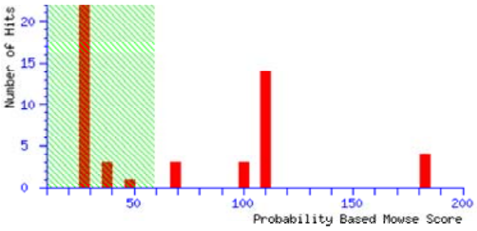

Protein Summary Report

Format As | Protein Summary (deprecated) | Help

Significance threshold p< 0.05 | Max. number of hits AUTO

Standard scoring ◉ MudPIT scoring ○ | Ions score or expect cut-off 0 | Show sub-sets 0

Show pop-ups ◉ Suppress pop-ups ○ | Sort unassigned Decreasing Score | Require bold red ☐

Re-Search All | Search Unmatched

Index

	Accession	Mass	Score	Description
1.	J9UWR4	35678	183	J9UWR4_BRAPL Flagellar filament outer layer protein FlaA OS=Brachyspira pilosicoli B2904 GN=B2904_orf2029 PE=4
2.	D8IBY6	35707	183	D8IBY6_BRAP9 Flagellar filament outer layer protein, FlaA OS=Brachyspira pilosicoli (strain ATCC BAA-1826 / 95
3.	K0JH04	35709	183	K0JH04_BRAPL Flagellar filament outer layer protein FlaA OS=Brachyspira pilosicoli WesB GN=flaA PE=4 SV=1
4.	Q5XPB3	36034	183	Q5XPB3_BRAPL FlaA1 (Fragment) OS=Brachyspira pilosicoli PE=4 SV=1
5.	B7T1P1	24517	108	B7T1P1_9SPIR FlaA (Fragment) OS=Brachyspira sp. SRBP205IR PE=4 SV=1
6.	B7T1P2	24517	108	B7T1P2_9SPIR FlaA (Fragment) OS=Brachyspira sp. SRBP5IR PE=4 SV=1
7.	B7T1P3	24499	108	B7T1P3_9SPIR FlaA (Fragment) OS=Brachyspira innocens PE=4 SV=1
8.	B7T1Q0	24499	108	B7T1Q0_9SPIR FlaA (Fragment) OS=Brachyspira alvinipulli PE=4 SV=1
9.	B7T1P9	24499	108	B7T1P9_9SPIR FlaA (Fragment) OS=Brachyspira intermedia PE=4 SV=1
10.	R5LCF6	35187	107	R5LCF6_9SPIR Flagellar filament outer layer protein FlaA OS=Brachyspira sp. CAG:700 GN=BN758_01892 PE=4 SV=1
11.	L8XQZ8	35741	105	L8XQZ8_9SPIR Flagellar filament outer layer protein FlaA OS=Brachyspira hampsonii 30599 GN=H263_11839 PE=4 SV=
12.	P32520	35964	105	FLAA1_BRAHO Flagellar filament outer layer protein flaA1 OS=Brachyspira hyodysenteriae GN=flaA1 PE=1 SV=1
13.	A0A0H0TRE1	35964	105	A0A0H0TRE1_BRAHO Flagellar filament protein FlaA OS=Brachyspira hyodysenteriae GN=SR30_03350 PE=4 SV=1
14.	A0A0G4K8P6	35920	105	A0A0G4K8P6_9SPIR Flagellar filament outer layer protein flaA1 OS=Brachyspira suanatina GN=flaA1 PE=4 SV=1
15.	C0R0T5	35964	105	C0R0T5_BRAHW Flagellar filament outer layer protein FlaA OS=Brachyspira hyodysenteriae (strain ATCC 49526 / WA
16.	A0A0H0V489	35934	105	A0A0H0V489_BRAHO Flagellar filament protein FlaA OS=Brachyspira hyodysenteriae GN=SZ48_11445 PE=4 SV=1
17.	A0A0H0WR23	35965	105	A0A0H0WR23_BRAHO Flagellar filament protein FlaA OS=Brachyspira hyodysenteriae GN=SZ42_05145 PE=4 SV=1
18.	G0EJW8	35935	105	G0EJW8_BRAIP Flagellar filament outer layer protein FlaA OS=Brachyspira intermedia (strain ATCC 51140 / PWS/A)
19.	B7T1P4	24426	104	B7T1P4_9SPIR FlaA (Fragment) OS=Brachyspira innocens PE=4 SV=1
20.	B7T1P5	24371	104	B7T1P5_9SPIR FlaA (Fragment) OS=Brachyspira innocens PE=4 SV=1
21.	D5U8F3	35700	101	D5U8F3_BRAM5 Flagellar filament outer layer protein FlaA OS=Brachyspira murdochii (strain ATCC 51284 / DSM 125
22.	B7T1P7	24514	73	B7T1P7_9SPIR FlaA (Fragment) OS=Brachyspira canis PE=4 SV=1
23.	B7T1P6	24483	73	B7T1P6_9SPIR FlaA (Fragment) OS=Brachyspira intermedia PE=4 SV=1
24.	L0X4M2	36070	71	L0X4M2_9SPIR Flagellar filament outer layer protein FlaA OS=Brachyspira hampsonii 30446 GN=A966_04095 PE=4 SV=
25.	D5U6R3	35736	50	D5U6R3_BRAM5 Glycosyl transferase family 2 OS=Brachyspira murdochii (strain ATCC 51284 / DSM 12563 / 56-150) G

Results List

1. J9UWR4 **Mass:** 35678 **Score:** 183 **Expect:** 2e-14 **Queries matched:** 3
J9UWR4_BRAPL Flagellar filament outer layer protein FlaA OS=Brachyspira pilosicoli B2904 GN=B2904_orf2029 PE=4 SV=1

Observed	Mr(expt)	Mr(calc)	ppm	Start - End	Miss	Ions	Peptide
970.5081	969.5008	969.4920	9.15	217 - 224	0	60	K.LDSLGFYR.T
1569.8347	1568.8274	1568.8270	0.25	279 - 291	1	60	R.VREAAELQQLEQR.R
1625.8740	1624.8667	1624.8647	1.25	211 - 224	1	18	K.MIPSVKLDSLGFYR.T

No match to: 1275.5588, 1583.8528

2. D8IBY6 **Mass:** 35707 **Score:** 183 **Expect:** 2e-14 **Queries matched:** 3
D8IBY6_BRAP9 Flagellar filament outer layer protein, FlaA OS=Brachyspira pilosicoli (strain ATCC BAA-1826 / 95/1000) GN=flaA PE=4 SV=1

Observed	Mr(expt)	Mr(calc)	ppm	Start - End	Miss	Ions	Peptide
970.5081	969.5008	969.4920	9.15	217 - 224	0	60	K.LDSLGFYR.T
1569.8347	1568.8274	1568.8270	0.25	279 - 291	1	60	R.VREAAELQQLEQR.R
1625.8740	1624.8667	1624.8647	1.25	211 - 224	1	18	K.MIPSVKLDSLGFYR.T

No match to: 1275.5588, 1583.8528

3. K0JH04 **Mass:** 35709 **Score:** 183 **Expect:** 2e-14 **Queries matched:** 3
K0JH04_BRAPL Flagellar filament outer layer protein FlaA OS=Brachyspira pilosicoli WesB GN=flaA PE=4 SV=1

Observed	Mr(expt)	Mr(calc)	ppm	Start	End	Miss	Ions	Peptide
970.5081	969.5008	969.4920	9.15	217	224	0	60	K.LDSLGFYR.T
1569.8347	1568.8274	1568.8270	0.25	279	291	1	60	R.VREAAELQQLEQR.R
1625.8740	1624.8667	1624.8647	1.25	211	224	1	18	K.MIPSVKLDSLGFYR.T

No match to: 1275.5588, 1583.8528

4. Q5XPB3 **Mass:** 36034 **Score:** 183 **Expect:** 2e-14 **Queries matched:** 3
Q5XPB3_BRAPL FlaA1 (Fragment) OS=Brachyspira pilosicoli PE=4 SV=1

Observed	Mr(expt)	Mr(calc)	ppm	Start	End	Miss	Ions	Peptide
970.5081	969.5008	969.4920	9.15	217	224	0	60	K.LDSLGFYR.T
1569.8347	1568.8274	1568.8270	0.25	279	291	1	60	R.VREAAELQQLEQR.R
1625.8740	1624.8667	1624.8647	1.25	211	224	1	18	K.MIPSVKLDSLGFYR.T

No match to: 1275.5588, 1583.8528

5. B7T1P1 **Mass:** 24517 **Score:** 108 **Expect:** 6.4e-07 **Queries matched:** 2
B7T1P1_9SPIR FlaA (Fragment) OS=Brachyspira sp. SRBP205IR PE=4 SV=1

Observed	Mr(expt)	Mr(calc)	ppm	Start	End	Miss	Ions	Peptide
970.5081	969.5008	969.4920	9.15	186	193	0	60	K.LDSLGFYR.T
1625.8740	1624.8667	1624.8647	1.25	180	193	1	18	K.MIPSVKLDSLGFYR.T

No match to: 1275.5588, 1569.8347, 1583.8528

6. B7T1P2 **Mass:** 24517 **Score:** 108 **Expect:** 6.4e-07 **Queries matched:** 2
B7T1P2_9SPIR FlaA (Fragment) OS=Brachyspira sp. SRBP5IR PE=4 SV=1

Observed	Mr(expt)	Mr(calc)	ppm	Start	End	Miss	Ions	Peptide
970.5081	969.5008	969.4920	9.15	186	193	0	60	K.LDSLGFYR.T
1625.8740	1624.8667	1624.8647	1.25	180	193	1	18	K.MIPSVKLDSLGFYR.T

No match to: 1275.5588, 1569.8347, 1583.8528

7. B7T1P3 **Mass:** 24499 **Score:** 108 **Expect:** 6.4e-07 **Queries matched:** 2
B7T1P3_9SPIR FlaA (Fragment) OS=Brachyspira innocens PE=4 SV=1

Observed	Mr(expt)	Mr(calc)	ppm	Start	End	Miss	Ions	Peptide
970.5081	969.5008	969.4920	9.15	186	193	0	60	K.LDSLGFYR.T
1625.8740	1624.8667	1624.8647	1.25	180	193	1	18	R.MIPSVKLDSLGFYR.T

No match to: 1275.5588, 1569.8347, 1583.8528

8. B7T1Q0 **Mass:** 24499 **Score:** 108 **Expect:** 6.4e-07 **Queries matched:** 2
B7T1Q0_9SPIR FlaA (Fragment) OS=Brachyspira alvinipulli PE=4 SV=1

Observed	Mr(expt)	Mr(calc)	ppm	Start	End	Miss	Ions	Peptide
970.5081	969.5008	969.4920	9.15	186	193	0	60	K.LDSLGFYR.T
1625.8740	1624.8667	1624.8647	1.25	180	193	1	18	R.MIPSVKLDSLGFYR.T

No match to: 1275.5588, 1569.8347, 1583.8528

9. B7T1P9 **Mass:** 24499 **Score:** 108 **Expect:** 6.4e-07 **Queries matched:** 2
B7T1P9_9SPIR FlaA (Fragment) OS=Brachyspira intermedia PE=4 SV=1

Observed	Mr(expt)	Mr(calc)	ppm	Start	End	Miss	Ions	Peptide
970.5081	969.5008	969.4920	9.15	186	193	0	60	K.LDSLGFYR.T
1625.8740	1624.8667	1624.8647	1.25	180	193	1	18	R.MIPSVKLDSLGFYR.T

No match to: 1275.5588, 1569.8347, 1583.8528

10. R5LCF6 **Mass:** 35187 **Score:** 107 **Expect:** 8.1e-07 **Queries matched:** 2
R5LCF6_9SPIR Flagellar filament outer layer protein FlaA OS=Brachyspira sp. CAG:700 GN=BN758_01892 PE=4 SV=1

Observed	Mr(expt)	Mr(calc)	ppm	Start	End	Miss	Ions	Peptide
970.5081	969.5008	969.4920	9.15	217	224	0	60	K.IDSIGFYR.T
1625.8740	1624.8667	1624.8647	1.25	211	224	1	19	K.LMPSVKIDSIGFYR.T

No match to: 1275.5588, 1569.8347, 1583.8528

11. L8XQZ8 **Mass:** 35741 **Score:** 105 **Expect:** 1.3e-06 **Queries matched:** 2
L8XQZ8_9SPIR Flagellar filament outer layer protein FlaA OS=Brachyspira hampsonii 30599 GN=H263_11839 PE=4 SV=1

Observed	Mr(expt)	Mr(calc)	ppm	Start	End	Miss	Ions	Peptide
970.5081	969.5008	969.4920	9.15	217	224	0	60	K.LDSLGFYR.T
1625.8740	1624.8667	1624.8647	1.25	211	224	1	18	R.MIPSVKLDSLGFYR.T

No match to: 1275.5588, 1569.8347, 1583.8528

12. P32520 **Mass:** 35964 **Score:** 105 **Expect:** 1.3e-06 **Queries matched:** 2
FLAA1_BRAHO Flagellar filament outer layer protein flaA1 OS=Brachyspira hyodysenteriae GN=flaA1 PE=1 SV=1

Observed	Mr(expt)	Mr(calc)	ppm	Start	End	Miss	Ions	Peptide
970.5081	969.5008	969.4920	9.15	217	224	0	60	K.LDSLGFYR.T
1625.8740	1624.8667	1624.8647	1.25	211	224	1	18	R.MIPSVKLDSLGFYR.T

No match to: 1275.5588, 1569.8347, 1583.8528

13. A0A0H0TRE1 **Mass:** 35964 **Score:** 105 **Expect:** 1.3e-06 **Queries matched:** 2
A0A0H0TRE1_BRAHO Flagellar filament protein FlaA OS=Brachyspira hyodysenteriae GN=SR30_03350 PE=4 SV=1

Observed	Mr(expt)	Mr(calc)	ppm	Start	End	Miss	Ions	Peptide
970.5081	969.5008	969.4920	9.15	217	224	0	60	K.LDSLGFYR.T
1625.8740	1624.8667	1624.8647	1.25	211	224	1	18	R.MIPSVKLDSLGFYR.T

No match to: 1275.5588, 1569.8347, 1583.8528

14. A0A0G4K8P6 **Mass:** 35920 **Score:** 105 **Expect:** 1.3e-06 **Queries matched:** 2
A0A0G4K8P6_9SPIR Flagellar filament outer layer protein flaA1 OS=Brachyspira suanatina GN=flaA1 PE=4 SV=1

Observed	Mr(expt)	Mr(calc)	ppm	Start	End	Miss	Ions	Peptide
970.5081	969.5008	969.4920	9.15	217	224	0	60	K.LDSLGFYR.T
1625.8740	1624.8667	1624.8647	1.25	211	224	1	18	R.MIPSVKLDSLGFYR.T

No match to: 1275.5588, 1569.8347, 1583.8528

15. C0R0T5 **Mass:** 35964 **Score:** 105 **Expect:** 1.3e-06 **Queries matched:** 2

C0R0T5_BRAHW Flagellar filament outer layer protein FlaA OS=Brachyspira hyodysenteriae (strain ATCC 49526 / WA1) GN=flaA PE=4 SV=1

Observed	Mr(expt)	Mr(calc)	ppm	Start - End	Miss	Ions	Peptide
970.5081	969.5008	969.4920	9.15	217 - 224	0	60	K.LDSLGFYR.T
1625.8740	1624.8667	1624.8647	1.25	211 - 224	1	18	R.MIPSVKLDSLGFYR.T

No match to: 1275.5588, 1569.8347, 1583.8528

16. A0A0H0V489 **Mass:** 35934 **Score:** 105 **Expect:** 1.3e-06 **Queries matched:** 2

A0A0H0V489_BRAHO Flagellar filament protein FlaA OS=Brachyspira hyodysenteriae GN=SZ48_11445 PE=4 SV=1

Observed	Mr(expt)	Mr(calc)	ppm	Start - End	Miss	Ions	Peptide
970.5081	969.5008	969.4920	9.15	217 - 224	0	60	K.LDSLGFYR.T
1625.8740	1624.8667	1624.8647	1.25	211 - 224	1	18	R.MIPSVKLDSLGFYR.T

No match to: 1275.5588, 1569.8347, 1583.8528

17. A0A0H0WR23 **Mass:** 35965 **Score:** 105 **Expect:** 1.3e-06 **Queries matched:** 2

A0A0H0WR23_BRAHO Flagellar filament protein FlaA OS=Brachyspira hyodysenteriae GN=SZ42_05145 PE=4 SV=1

Observed	Mr(expt)	Mr(calc)	ppm	Start - End	Miss	Ions	Peptide
970.5081	969.5008	969.4920	9.15	217 - 224	0	60	K.LDSLGFYR.T
1625.8740	1624.8667	1624.8647	1.25	211 - 224	1	18	R.MIPSVKLDSLGFYR.T

No match to: 1275.5588, 1569.8347, 1583.8528

18. G0EJW8 **Mass:** 35935 **Score:** 105 **Expect:** 1.3e-06 **Queries matched:** 2

G0EJW8_BRAIP Flagellar filament outer layer protein FlaA OS=Brachyspira intermedia (strain ATCC 51140 / PWS/A) GN=Bint_0608 PE=4 SV=1

Observed	Mr(expt)	Mr(calc)	ppm	Start - End	Miss	Ions	Peptide
970.5081	969.5008	969.4920	9.15	217 - 224	0	60	K.LDSLGFYR.T
1625.8740	1624.8667	1624.8647	1.25	211 - 224	1	18	R.MIPSVKLDSLGFYR.T

No match to: 1275.5588, 1569.8347, 1583.8528

19. B7T1P4 **Mass:** 24426 **Score:** 104 **Expect:** 1.6e-06 **Queries matched:** 2

B7T1P4_9SPIR FlaA (Fragment) OS=Brachyspira innocens PE=4 SV=1

Observed	Mr(expt)	Mr(calc)	ppm	Start - End	Miss	Ions	Peptide
970.5081	969.5008	969.4920	9.15	186 - 193	0	60	K.LDSLGFYR.T
1625.8740	1624.8667	1624.8647	1.25	180 - 193	1	13	R.MVPSIKLDSLGFYR.T

No match to: 1275.5588, 1569.8347, 1583.8528

20. B7T1P5 **Mass:** 24371 **Score:** 104 **Expect:** 1.6e-06 **Queries matched:** 2

B7T1P5_9SPIR FlaA (Fragment) OS=Brachyspira innocens PE=4 SV=1

Observed	Mr(expt)	Mr(calc)	ppm	Start - End	Miss	Ions	Peptide
970.5081	969.5008	969.4920	9.15	186 - 193	0	60	K.LDSLGFYR.T
1625.8740	1624.8667	1624.8647	1.25	180 - 193	1	13	K.MVPSIKLDSLGFYR.T

No match to: 1275.5588, 1569.8347, 1583.8528

21. D5U8F3 **Mass:** 35700 **Score:** 101 **Expect:** 3.2e-06 **Queries matched:** 2

D5U8F3_BRAM5 Flagellar filament outer layer protein FlaA OS=Brachyspira murdochii (strain ATCC 51284 / DSM 12563 / 56-150) GN=Bmur_087

Observed	Mr(expt)	Mr(calc)	ppm	Start - End	Miss	Ions	Peptide
970.5081	969.5008	969.4920	9.15	217 - 224	0	60	K.LDSLGFYR.T
1625.8740	1624.8667	1624.8647	1.25	211 - 224	1	13	R.MVPSIKLDSLGFYR.T

No match to: 1275.5588, 1569.8347, 1583.8528

22. B7T1P7 **Mass:** 24514 **Score:** 73 **Expect:** 0.002 **Queries matched:** 1

B7T1P7_9SPIR FlaA (Fragment) OS=Brachyspira canis PE=4 SV=1

Observed	Mr(expt)	Mr(calc)	ppm	Start - End	Miss	Ions	Peptide
970.5081	969.5008	969.4920	9.15	186 - 193	0	60	K.LDSLGFYR.T

No match to: 1275.5588, 1569.8347, 1583.8528, 1625.8740

23. B7T1P6 **Mass:** 24483 **Score:** 73 **Expect:** 0.002 **Queries matched:** 1

B7T1P6_9SPIR FlaA (Fragment) OS=Brachyspira intermedia PE=4 SV=1

Observed	Mr(expt)	Mr(calc)	ppm	Start - End	Miss	Ions	Peptide
970.5081	969.5008	969.4920	9.15	186 - 193	0	60	K.LDSLGFYR.T

No match to: 1275.5588, 1569.8347, 1583.8528, 1625.8740

24. L0X4M2 **Mass:** 36070 **Score:** 71 **Expect:** 0.0029 **Queries matched:** 1

L0X4M2_9SPIR Flagellar filament outer layer protein FlaA OS=Brachyspira hampsonii 30446 GN=A966_04095 PE=4 SV=1

Observed	Mr(expt)	Mr(calc)	ppm	Start - End	Miss	Ions	Peptide
970.5081	969.5008	969.4920	9.15	217 - 224	0	60	K.LDSLGFYR.T

No match to: 1275.5588, 1569.8347, 1583.8528, 1625.8740

25. D5U6R3 **Mass:** 35736 **Score:** 50 **Expect:** 0.36 **Queries matched:** 2

D5U6R3_BRAM5 Glycosyl transferase family 2 OS=Brachyspira murdochii (strain ATCC 51284 / DSM 12563 / 56-150) GN=Bmur_0526 PE=4 SV=1

Observed	Mr(expt)	Mr(calc)	ppm	Start - End	Miss	Ions	Peptide
970.5081	969.5008	969.5284	-28.37	134 - 141	0	24	K.LTASLFYR.L
1625.8740	1624.8667	1624.8096	35.1	164 - 177	1	---	K.NVIEAYKSFSENPK.Y

No match to: 1275.5588, 1569.8347, 1583.8528

Search Parameters

```
Type of search         : Sequence Query
Enzyme                 : Trypsin
Fixed modifications    : Carbamidomethyl (C)
Variable modifications : Oxidation (M)
Mass values            : Monoisotopic
Protein Mass           : Unrestricted
Peptide Mass Tolerance : ± 50 ppm
Fragment Mass Tolerance: ± 0.5 Da
Max Missed Cleavages   : 1
Instrument type        : MALDI-TOF-TOF
Query1 (970.5081,1+)   : Locus:3.1.1.213486.57246
Query2 (1275.5588,1+)  : Locus:3.1.1.213486.57248
Query3 (1569.8347,1+)  : Locus:3.1.1.213486.57247
```

```
Query4 (1583.8528,1+)  : Locus:3.1.1.213486.57249
Query5 (1625.8740,1+)  : Locus:3.1.1.213486.57250
```

Mascot: http://www.matrixscience.com/

MATRIX SCIENCE Mascot Search Results

```
User          :
Email         :
Search title  : F:\ROD_inmunoproteoma\MALDI\ppw_2016_562_563_ROD\ppw_I22_145397051038.txt
MS data file  : F:\ROD_inmunoproteoma\MALDI\ppw_2016_562_563_ROD\ppw_I22_145397051038.txt
Database      : Brachyspiraceae Brachyspiraceae_20160127-151122 (40573 sequences; 13470793 residues)
Timestamp     : 23 Feb 2016 at 16:06:39 GMT
Warning       : A Peptide summary report will usually give a much clearer picture of MS/MS search results.
Top Score     : 235 for Q9FA06, Q9FA06_BRAPL Putative elongation factor OS=Brachyspira pilosicoli GN=tuf PE=4 SV=1
```

Probability Based Mowse Score

Protein score is -10*Log(P), where P is the probability that the observed match is a random event.
Protein scores greater than 59 are significant (p<0.05).
Protein scores are derived from ions scores as a non-probabilistic basis for ranking protein hits.

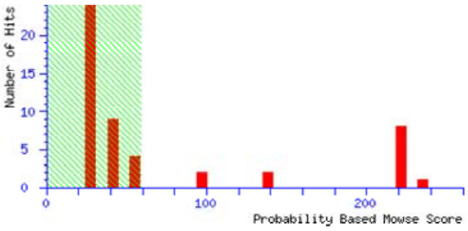

Protein Summary Report

Format As | Protein Summary (deprecated) | Help

Significance threshold p< 0.05 Max. number of hits AUTO

Standard scoring ◉ MudPIT scoring ○ Ions score or expect cut-off 0 Show sub-sets 0

Show pop-ups ◉ Suppress pop-ups ○ Sort unassigned Decreasing Score Require bold red ☐

Re-Search All | Search Unmatched

Index

	Accession	Mass	Score	Description
1.	Q9FA06	16290	235	Q9FA06_BRAPL Putative elongation factor OS=Brachyspira pilosicoli GN=tuf PE=4 SV=1
2.	C0QVZ4	44776	226	EFTU_BRAHW Elongation factor Tu OS=Brachyspira hyodysenteriae (strain ATCC 49526 / WA1) GN=tuf PE=3 SV=1
3.	A0A0H0TPC4	44776	226	A0A0H0TPC4_BRAHO Elongation factor Tu OS=Brachyspira hyodysenteriae GN=tuf PE=3 SV=1
4.	A0A0G4K3M8	44733	226	A0A0G4K3M8_9SPIR Elongation factor Tu OS=Brachyspira suanatina GN=tuf PE=3 SV=1
5.	G0ENS4	44733	226	G0ENS4_BRAIP Elongation factor Tu OS=Brachyspira intermedia (strain ATCC 51140 / PWS/A) GN=tufA PE=3 SV=1
6.	J9UB96	44777	226	J9UB96_BRAPL Elongation factor Tu OS=Brachyspira pilosicoli B2904 GN=el-tu PE=3 SV=1
7.	D8ICZ6	44826	226	D8ICZ6_BRAP9 Elongation factor Tu OS=Brachyspira pilosicoli (strain ATCC BAA-1826 / 95/1000) GN=tuf PE=3 SV=1
8.	K0JL99	44826	226	K0JL99_BRAPL Elongation factor Tu OS=Brachyspira pilosicoli WesB GN=tufA PE=3 SV=1
9.	P52854	45030	226	EFTU_BRAHO Elongation factor Tu OS=Brachyspira hyodysenteriae GN=tuf PE=3 SV=1
10.	R5LAV7	44698	132	R5LAV7_9SPIR Elongation factor Tu OS=Brachyspira sp. CAG:700 GN=tuf PE=3 SV=1
11.	L8XS56	44815	132	L8XS56_9SPIR Elongation factor Tu OS=Brachyspira hampsonii 30599 GN=tuf PE=3 SV=1
12.	D5UB43	44823	91	D5UB43_BRAM5 Elongation factor Tu OS=Brachyspira murdochii (strain ATCC 51284 / DSM 12563 / 56-150) GN=tuf PE=
13.	L0X3D8	44918	91	L0X3D8_9SPIR Elongation factor Tu OS=Brachyspira hampsonii 30446 GN=tuf PE=3 SV=1
14.	S4UT19	31706	50	S4UT19_BRAHO NADH oxidase (Fragment) OS=Brachyspira hyodysenteriae GN=nox PE=4 SV=1

Results List

1. Q9FA06 **Mass:** 16290 **Score:** 235 **Expect:** 1.3e-19 **Queries matched:** 2
Q9FA06_BRAPL Putative elongation factor OS=Brachyspira pilosicoli GN=tuf PE=4 SV=1

Observed	Mr(expt)	Mr(calc)	ppm	Start	End	Miss	Ions	Peptide
1619.7781	1618.7708	1618.8501	-48.98	53 -	67	0	120	K.EVVGIAGYNVGCLLR.G
1831.7817	1830.7744	1830.8624	-48.06	107 -	121	0	80	R.HSGFVSGYRPQMYFR.T

No match to: 1239.6116, 1633.7905, 1654.8632

2. C0QVZ4 **Mass:** 44776 **Score:** 226 **Expect:** 1e-18 **Queries matched:** 2
EFTU_BRAHW Elongation factor Tu OS=Brachyspira hyodysenteriae (strain ATCC 49526 / WA1) GN=tuf PE=3 SV=1

Observed	Mr(expt)	Mr(calc)	ppm	Start	End	Miss	Ions	Peptide
1619.7781	1618.7708	1618.8501	-48.98	280 -	294	0	120	K.EVVGIAGYNVGCLLR.G
1831.7817	1830.7744	1830.8624	-48.06	334 -	348	0	80	R.HSGFVSGYRPQMYFR.T

No match to: 1239.6116, 1633.7905, 1654.8632

3. A0A0H0TPC4 **Mass:** 44776 **Score:** 226 **Expect:** 1e-18 **Queries matched:** 2
A0A0H0TPC4_BRAHO Elongation factor Tu OS=Brachyspira hyodysenteriae GN=tuf PE=3 SV=1

Observed	Mr(expt)	Mr(calc)	ppm	Start	End	Miss	Ions	Peptide
1619.7781	1618.7708	1618.8501	-48.98	280 -	294	0	120	K.EVVGIAGYNVGCLLR.G
1831.7817	1830.7744	1830.8624	-48.06	334 -	348	0	80	R.HSGFVSGYRPQMYFR.T

No match to: 1239.6116, 1633.7905, 1654.8632

4. A0A0G4K3M8 **Mass:** 44733 **Score:** 226 **Expect:** 1e-18 **Queries matched:** 2
A0A0G4K3M8_9SPIR Elongation factor Tu OS=Brachyspira suanatina GN=tuf PE=3 SV=1

Observed	Mr(expt)	Mr(calc)	ppm	Start	End	Miss	Ions	Peptide
1619.7781	1618.7708	1618.8501	-48.98	280 -	294	0	120	K.EVVGIAGYNVGCLLR.G
1831.7817	1830.7744	1830.8624	-48.06	334 -	348	0	80	R.HSGFVSGYRPQMYFR.T

No match to: 1239.6116, 1633.7905, 1654.8632

5. G0ENS4 **Mass:** 44733 **Score:** 226 **Expect:** 1e-18 **Queries matched:** 2
G0ENS4_BRAIP Elongation factor Tu OS=Brachyspira intermedia (strain ATCC 51140 / PWS/A) GN=tufA PE=3 SV=1

Observed	Mr(expt)	Mr(calc)	ppm	Start	End	Miss	Ions	Peptide
1619.7781	1618.7708	1618.8501	-48.98	280 -	294	0	120	K.EVVGIAGYNVGCLLR.G
1831.7817	1830.7744	1830.8624	-48.06	334 -	348	0	80	R.HSGFVSGYRPQMYFR.T

No match to: 1239.6116, 1633.7905, 1654.8632

6. J9UB96 **Mass:** 44777 **Score:** 226 **Expect:** 1e-18 **Queries matched:** 2
J9UB96_BRAPL Elongation factor Tu OS=Brachyspira pilosicoli B2904 GN=el-tu PE=3 SV=1

Observed	Mr(expt)	Mr(calc)	ppm	Start	End	Miss	Ions	Peptide
1619.7781	1618.7708	1618.8501	-48.98	280 -	294	0	120	K.EVVGIAGYNVGCLLR.G
1831.7817	1830.7744	1830.8624	-48.06	334 -	348	0	80	R.HSGFVSGYRPQMYFR.T

No match to: 1239.6116, 1633.7905, 1654.8632

7. D8ICZ6 **Mass:** 44826 **Score:** 226 **Expect:** 1e-18 **Queries matched:** 2
D8ICZ6_BRAP9 Elongation factor Tu OS=Brachyspira pilosicoli (strain ATCC BAA-1826 / 95/1000) GN=tuf PE=3 SV=1

Observed	Mr(expt)	Mr(calc)	ppm	Start	End	Miss	Ions	Peptide
1619.7781	1618.7708	1618.8501	-48.98	280 -	294	0	120	K.EVVGIAGYNVGCLLR.G
1831.7817	1830.7744	1830.8624	-48.06	334 -	348	0	80	R.HSGFVSGYRPQMYFR.T

No match to: 1239.6116, 1633.7905, 1654.8632

8. K0JL99 **Mass:** 44826 **Score:** 226 **Expect:** 1e-18 **Queries matched:** 2
K0JL99_BRAPL Elongation factor Tu OS=Brachyspira pilosicoli WesB GN=tufA PE=3 SV=1

Observed	Mr(expt)	Mr(calc)	ppm	Start	End	Miss	Ions	Peptide
1619.7781	1618.7708	1618.8501	-48.98	280 -	294	0	120	K.EVVGIAGYNVGCLLR.G
1831.7817	1830.7744	1830.8624	-48.06	334 -	348	0	80	R.HSGFVSGYRPQMYFR.T

No match to: 1239.6116, 1633.7905, 1654.8632

9. P52854 **Mass:** 45030 **Score:** 226 **Expect:** 1e-18 **Queries matched:** 2
EFTU_BRAHO Elongation factor Tu OS=Brachyspira hyodysenteriae GN=tuf PE=3 SV=1

Observed	Mr(expt)	Mr(calc)	ppm	Start	End	Miss	Ions	Peptide
1619.7781	1618.7708	1618.8501	-48.98	280 -	294	0	120	K.EVVGIAGYNVGCLLR.G
1831.7817	1830.7744	1830.8624	-48.06	334 -	348	0	80	R.HSGFVSGYRPQMYFR.T

No match to: 1239.6116, 1633.7905, 1654.8632

10. R5LAV7 **Mass:** 44698 **Score:** 132 **Expect:** 2.6e-09 **Queries matched:** 1
R5LAV7_9SPIR Elongation factor Tu OS=Brachyspira sp. CAG:700 GN=tuf PE=3 SV=1

Observed	Mr(expt)	Mr(calc)	ppm	Start	End	Miss	Ions	Peptide
1619.7781	1618.7708	1618.8501	-48.98	279 -	293	0	120	K.EVVGIAGYNVGCLLR.G

No match to: 1239.6116, 1633.7905, 1654.8632, 1831.7817

11. L8XS56 **Mass:** 44815 **Score:** 132 **Expect:** 2.6e-09 **Queries matched:** 1
L8XS56_9SPIR Elongation factor Tu OS=Brachyspira hampsonii 30599 GN=tuf PE=3 SV=1

Observed	Mr(expt)	Mr(calc)	ppm	Start	End	Miss	Ions	Peptide
1619.7781	1618.7708	1618.8501	-48.98	280 -	294	0	120	K.EVVGIAGYNVGCLLR.G

No match to: 1239.6116, 1633.7905, 1654.8632, 1831.7817

12. D5UB43 **Mass:** 44823 **Score:** 91 **Expect:** 3e-05 **Queries matched:** 1
D5UB43_BRAM5 Elongation factor Tu OS=Brachyspira murdochii (strain ATCC 51284 / DSM 12563 / 56-150) GN=tuf PE=3 SV=1

Observed	Mr(expt)	Mr(calc)	ppm	Start	End	Miss	Ions	Peptide
1831.7817	1830.7744	1830.8624	-48.06	334 -	348	0	80	R.HSGFVSGYRPQMYFR.T

No match to: 1239.6116, 1619.7781, 1633.7905, 1654.8632

13. L0X3D8 **Mass:** 44918 **Score:** 91 **Expect:** 3e-05 **Queries matched:** 1
L0X3D8_9SPIR Elongation factor Tu OS=Brachyspira hampsonii 30446 GN=tuf PE=3 SV=1

Observed	Mr(expt)	Mr(calc)	ppm	Start	End	Miss	Ions	Peptide
1831.7817	1830.7744	1830.8624	-48.06	334 -	348	0	80	R.HSGFVSGYRPQMYFR.T

No match to: 1239.6116, 1619.7781, 1633.7905, 1654.8632

14. S4UT19 **Mass:** 31706 **Score:** 50 **Expect:** 0.37 **Queries matched:** 2
S4UT19_BRAHO NADH oxidase (Fragment) OS=Brachyspira hyodysenteriae GN=nox PE=4 SV=1

Observed	Mr(expt)	Mr(calc)	ppm	Start	End	Miss	Ions	Peptide
1239.6116	1238.6043	1238.6295	-20.34	10 -	20	0	22	K.GLFYASPESLR.G
1654.8632	1653.8559	1653.8760	-12.12	162 -	176	1	---	R.IKEAGIEMHLGETVK.K

No match to: 1619.7781, 1633.7905, 1831.7817

Search Parameters

Type of search : Sequence Query
Enzyme : Trypsin
Fixed modifications : Carbamidomethyl (C)
Variable modifications : Oxidation (M)
Mass values : Monoisotopic
Protein Mass : Unrestricted
Peptide Mass Tolerance : ± 50 ppm
Fragment Mass Tolerance: ± 0.5 Da
Max Missed Cleavages : 1
Instrument type : MALDI-TOF-TOF
Query1 (1239.6116,1+) : Locus:39.1.1.211186.56708
Query2 (1619.7781,1+) : Locus:39.1.1.211186.56706
Query3 (1633.7905,1+) : Locus:39.1.1.211186.56707
Query4 (1654.8632,1+) : Locus:39.1.1.211186.56709
Query5 (1831.7817,1+) : Locus:39.1.1.211186.56705

Mascot: http://www.matrixscience.com/

Mascot Search Results

User : VANESSA CASAS
Email : vanessa.casas.csic@uab.cat
Search title : Project: _SERVEI, Spot Set: _SERVEI\160127_2016_562_563_ROD, Label: I24, Spot Id: 211188, Peak List Id: 56513, MS Job Run Id: 13133
Database : NCBIprot 20160920 (95963614 sequences; 35367425320 residues)
Taxonomy : Other Bacteria (8703853 sequences)
Timestamp : 8 Nov 2016 at 16:34:22 GMT
Protein hits : AAG27262.1 putative elongation factor [Brachyspira pilosicoli P43/6/78]
ADY17422.1 NADH oxidase, partial [Brachyspira hyodysenteriae]
WP_051234385.1 hypothetical protein [Flavobacterium denitrificans]

Mascot Score Histogram

Ions score is -10*Log(P), where P is the probability that the observed match is a random event.
Individual ions scores > 51 indicate identity or extensive homology (p<0.05).
Protein scores are derived from ions scores as a non-probabilistic basis for ranking protein hits.

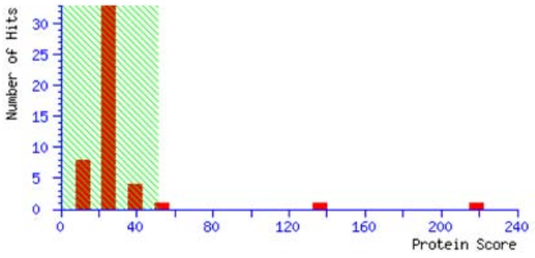

Peptide Summary Report

Format As | Protein Summary (deprecated) | Help

Significance threshold p< 0.05 | Max. number of hits AUTO

Standard scoring ◉ MudPIT scoring ○ | Ions score or expect cut-off 0 | Show sub-sets 0

Show pop-ups ◉ Suppress pop-ups ○ | Sort unassigned Decreasing Score | Require bold red ☐

Preferred taxonomy All entries

Select All | Select None | Search Selected | ☐ **Error tolerant**

1. AAG27262.1 **Mass:** 16290 **Score:** 219 **Matches:** 2(2) **Sequences:** 2(2)
putative elongation factor [Brachyspira pilosicoli P43/6/78]
☐ Check to include this hit in error tolerant search

	Query	Observed	Mr(expt)	Mr(calc)	ppm	Miss	Score	Expect	Rank	Unique	Peptide
☑	31	1619.7588	1618.7515	1618.8501	-60.90	0	131	5.7e-10	1	U	K.EVVGIAGYNVGCLLR.G
☑	49	1831.7595	1830.7522	1830.8624	-60.18	0	87	4.3e-06	1	U	R.HSGFVSGYRPQMYFR.T

Proteins matching the same set of peptides:
P52854.1 **Mass:** 45030 **Score:** 219 **Matches:** 2(2) **Sequences:** 2(2)
RecName: Full=Elongation factor Tu; Short=EF-Tu
WP_012671618.1 **Mass:** 44776 **Score:** 219 **Matches:** 2(2) **Sequences:** 2(2)
elongation factor Tu [Brachyspira hyodysenteriae]
WP_013243972.1 **Mass:** 44826 **Score:** 219 **Matches:** 2(2) **Sequences:** 2(2)
elongation factor Tu [Brachyspira pilosicoli]
WP_014488947.1 **Mass:** 44733 **Score:** 219 **Matches:** 2(2) **Sequences:** 2(2)
MULTISPECIES: elongation factor Tu [Brachyspira]
WP_014935284.1 **Mass:** 44777 **Score:** 219 **Matches:** 2(2) **Sequences:** 2(2)
elongation factor Tu [Brachyspira pilosicoli]

---

2. ADY17422.1 **Mass:** 34637 **Score:** 132 **Matches:** 2(1) **Sequences:** 2(1)
NADH oxidase, partial [Brachyspira hyodysenteriae]
☐ Check to include this hit in error tolerant search

	Query	Observed	Mr(expt)	Mr(calc)	ppm	Miss	Score	Expect	Rank	Unique	Peptide
☑	11	1239.5878	1238.5805	1238.6295	-39.55	0	35	2.5	1	U	K.GLFYASPESLR.G
☑	41	1763.8203	1762.8130	1762.9213	-61.42	1	97	1.5e-06	1	U	R.ASEKQEYIALATNAVR.M

Proteins matching the same set of peptides:
ABF38935.1 **Mass:** 33017 **Score:** 132 **Matches:** 2(1) **Sequences:** 2(1)
NADH oxidase, partial [Brachyspira hyodysenteriae]
AAC78809.1 **Mass:** 46179 **Score:** 132 **Matches:** 2(1) **Sequences:** 2(1)
NADH oxidase, partial [Brachyspira hyodysenteriae]
AAC78810.1 **Mass:** 46713 **Score:** 132 **Matches:** 2(1) **Sequences:** 2(1)
NADH oxidase, partial [Brachyspira hyodysenteriae]
AGT96409.1 **Mass:** 32536 **Score:** 132 **Matches:** 2(1) **Sequences:** 2(1)
NADH oxidase, partial [Brachyspira hyodysenteriae ATCC 27164]
AGL98113.1 **Mass:** 31706 **Score:** 132 **Matches:** 2(1) **Sequences:** 2(1)
NADH oxidase, partial [Brachyspira hyodysenteriae]

[WP_020064195.1](#) **Mass:** 50538 **Score:** 132 **Matches:** 2(1) **Sequences:** 2(1)
NADH oxidase [Brachyspira hyodysenteriae]

---

3. [WP_051234385.1](#) **Score:** 52 **Matches:** 1(1) **Sequences:** 1(1)
hypothetical protein [Flavobacterium denitrificans]
☐ Check to include this hit in error tolerant search

Query	Observed	Mr(expt)	Mr(calc)	ppm	Miss	Score	Expect	Rank	Unique	Peptide
31	1619.7588	1618.7515	1618.8427	-56.31	1	52	0.05	2	U	K.ILRDAGYNTGAEALR.V

Peptide matches not assigned to protein hits: (no details means no match)

	Query	Observed	Mr(expt)	Mr(calc)	ppm	Miss	Score	Expect	Rank	Unique	Peptide
☑	33	1633.7716	1632.7643	1632.6751	54.7	0	36	2	1		EEDDLEDGVDNDIR
☑	1	1085.5281	1084.5208								
☑	2	1098.4585	1097.4512								
☑	3	1099.5371	1098.5298								
☑	4	1112.4869	1111.4796								
☑	5	1122.5157	1121.5084								
☑	6	1133.5273	1132.5200								
☑	7	1174.5845	1173.5772								
☑	8	1188.5500	1187.5427								
☑	9	1225.5327	1224.5254								
☑	10	1239.5878	1238.5805								
☑	12	1253.5980	1252.5907								
☑	13	1268.6134	1267.6061								
☑	14	1282.6237	1281.6164								
☑	15	1293.5377	1292.5304								
☑	16	1310.6257	1309.6184								
☑	17	1320.5023	1319.4950								
☑	18	1337.5541	1336.5468								
☑	19	1439.6378	1438.6305								
☑	20	1446.7356	1445.7283								
☑	21	1475.6736	1474.6663								
☑	22	1486.7246	1485.7173								
☑	23	1492.7498	1491.7425								
☑	24	1510.6757	1509.6684								
☑	25	1528.6064	1527.5991								
☑	26	1554.7181	1553.7108								
☑	27	1584.7322	1583.7249								
☑	28	1587.7841	1586.7768								
☑	29	1601.7826	1600.7753								
☑	30	1619.7588	1618.7515								
☑	32	1633.7716	1632.7643								
☑	34	1638.7588	1637.7515								
☑	35	1640.8264	1639.8191								
☑	36	1647.7831	1646.7758								
☑	37	1654.8409	1653.8336								
☑	38	1668.8459	1667.8386								
☑	39	1704.7662	1703.7589								
☑	40	1763.8203	1762.8130								
☑	42	1777.8351	1776.8278								
☑	43	1783.7593	1782.7520								
☑	44	1789.8940	1788.8867								
☑	45	1813.8254	1812.8181								
☑	46	1814.8396	1813.8323								
☑	47	1829.8109	1828.8036								
☑	48	1831.7595	1830.7522								
☑	50	1836.7936	1835.7863								
☑	51	1843.7834	1842.7761								
☑	52	1845.7725	1844.7652								
☑	53	1847.7859	1846.7786								
☑	54	1854.7991	1853.7918								
☑	55	1863.7876	1862.7803								
☑	56	1875.7799	1874.7726								
☑	57	1886.7743	1885.7670								
☑	58	1895.8973	1894.8900								
☑	59	1900.7914	1899.7841								
☑	60	2005.8354	2004.8281								
☑	61	2058.8596	2057.8523								
☑	62	2072.8752	2071.8679								
☑	63	2115.8896	2114.8823								
☑	64	2125.0005	2123.9932								
☑	65	2129.8982	2128.8909								
☑	66	2134.9817	2133.9744								
☑	67	2210.9929	2209.9856								
☑	68	2320.0193	2319.0120								
☑	69	2398.8877	2397.8804								
☑	70	2629.1228	2628.1155								
☑	71	2642.2373	2641.2300								

Search Parameters

```
Type of search         : Sequence Query
Enzyme                 : Trypsin
Fixed modifications    : Carbamidomethyl (C)
Variable modifications : Oxidation (M)
Mass values            : Monoisotopic
Protein Mass           : Unrestricted
Peptide Mass Tolerance : ± 70 ppm
Fragment Mass Tolerance: ± 0.6 Da
Max Missed Cleavages   : 1
Instrument type        : MALDI-TOF-TOF
Number of queries      : 71
```

Mascot: http://www.matrixscience.com/

Mascot Search Results

**User**	:
**Email**	:
**Search title**	: **F:\ROD_inmunoproteoma\MALDI\ppw_2016_562_563_ROD\ppw_I6_145397049722.txt**
**MS data file**	: **F:\ROD_inmunoproteoma\MALDI\ppw_2016_562_563_ROD\ppw_I6_145397049722.txt**
**Database**	: **Brachyspiraceae Brachyspiraceae_20160127-151122 (40573 sequences; 13470793 residues)**
**Timestamp**	: **23 Feb 2016 at 16:05:59 GMT**
**Warning**	: **A Peptide summary report will usually give a much clearer picture of MS/MS search results.**
**Top Score**	: **82 for B7T1P7, B7T1P7_9SPIR FlaA (Fragment) OS=Brachyspira canis PE=4 SV=1**

Probability Based Mowse Score

Protein score is -10*Log(P), where P is the probability that the observed match is a random event.
Protein scores greater than 59 are significant (p<0.05).
Protein scores are derived from ions scores as a non-probabilistic basis for ranking protein hits.

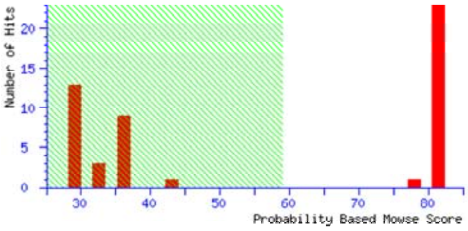

Protein Summary Report

Format As | Protein Summary (deprecated) | Help

Significance threshold p< 0.05 | Max. number of hits AUTO

Standard scoring ◉ MudPIT scoring ○ | Ions score or expect cut-off 0 | Show sub-sets 0

Show pop-ups ◉ Suppress pop-ups ○ | Sort unassigned Decreasing Score | Require bold red ☐

Re-Search All | Search Unmatched

Index

	Accession	Mass	Score	Description
1.	B7T1P7	24514	82	B7T1P7_9SPIR FlaA (Fragment) OS=Brachyspira canis PE=4 SV=1
2.	B7T1P1	24517	82	B7T1P1_9SPIR FlaA (Fragment) OS=Brachyspira sp. SRBP205IR PE=4 SV=1
3.	B7T1P2	24517	82	B7T1P2_9SPIR FlaA (Fragment) OS=Brachyspira sp. SRBP5IR PE=4 SV=1
4.	B7T1P3	24499	82	B7T1P3_9SPIR FlaA (Fragment) OS=Brachyspira innocens PE=4 SV=1
5.	B7T1P4	24426	82	B7T1P4_9SPIR FlaA (Fragment) OS=Brachyspira innocens PE=4 SV=1
6.	B7T1P5	24371	82	B7T1P5_9SPIR FlaA (Fragment) OS=Brachyspira innocens PE=4 SV=1
7.	B7T1Q0	24499	82	B7T1Q0_9SPIR FlaA (Fragment) OS=Brachyspira alvinipulli PE=4 SV=1
8.	B7T1P6	24483	82	B7T1P6_9SPIR FlaA (Fragment) OS=Brachyspira intermedia PE=4 SV=1
9.	B7T1P9	24499	82	B7T1P9_9SPIR FlaA (Fragment) OS=Brachyspira intermedia PE=4 SV=1
10.	D5U8F3	35700	80	D5U8F3_BRAM5 Flagellar filament outer layer protein FlaA OS=Brachyspira murdochii (strain ATCC 51284 / DSM 125
11.	J9UWR4	35678	80	J9UWR4_BRAPL Flagellar filament outer layer protein FlaA OS=Brachyspira pilosicoli B2904 GN=B2904_orf2029 PE=4
12.	D8IBY6	35707	80	D8IBY6_BRAP9 Flagellar filament outer layer protein, FlaA OS=Brachyspira pilosicoli (strain ATCC BAA-1826 / 95
13.	K0JH04	35709	80	K0JH04_BRAPL Flagellar filament outer layer protein FlaA OS=Brachyspira pilosicoli WesB GN=flaA PE=4 SV=1
14.	L8XQZ8	35741	80	L8XQZ8_9SPIR Flagellar filament outer layer protein FlaA OS=Brachyspira hampsonii 30599 GN=H263_11839 PE=4 SV=
15.	Q5XPB3	36034	80	Q5XPB3_BRAPL FlaA1 (Fragment) OS=Brachyspira pilosicoli PE=4 SV=1
16.	P32520	35964	80	FLAA1_BRAHO Flagellar filament outer layer protein flaA1 OS=Brachyspira hyodysenteriae GN=flaA1 PE=1 SV=1
17.	A0A0H0TRE1	35964	80	A0A0H0TRE1_BRAHO Flagellar filament protein FlaA OS=Brachyspira hyodysenteriae GN=SR30_03350 PE=4 SV=1
18.	A0A0G4K8P6	35920	80	A0A0G4K8P6_9SPIR Flagellar filament outer layer protein flaA1 OS=Brachyspira suanatina GN=flaA1 PE=4 SV=1
19.	C0R0T5	35964	80	C0R0T5_BRAHW Flagellar filament outer layer protein FlaA OS=Brachyspira hyodysenteriae (strain ATCC 49526 / WA
20.	A0A0H0V489	35934	80	A0A0H0V489_BRAHO Flagellar filament protein FlaA OS=Brachyspira hyodysenteriae GN=SZ48_11445 PE=4 SV=1
21.	A0A0H0WR23	35965	80	A0A0H0WR23_BRAHO Flagellar filament protein FlaA OS=Brachyspira hyodysenteriae GN=SZ42_05145 PE=4 SV=1
22.	G0EJW8	35935	80	G0EJW8_BRAIP Flagellar filament outer layer protein FlaA OS=Brachyspira intermedia (strain ATCC 51140 / PWS/A)
23.	L0X4M2	36070	80	L0X4M2_9SPIR Flagellar filament outer layer protein FlaA OS=Brachyspira hampsonii 30446 GN=A966_04095 PE=4 SV=
24.	R5LCF6	35187	78	R5LCF6_9SPIR Flagellar filament outer layer protein FlaA OS=Brachyspira sp. CAG:700 GN=BN758_01892 PE=4 SV=1
25.	D5U459	52256	42	D5U459_BRAM5 AAA ATPase OS=Brachyspira murdochii (strain ATCC 51284 / DSM 12563 / 56-150) GN=Bmur_2166 PE=4 SV

Results List

1. B7T1P7 **Mass:** 24514 **Score:** 82 **Expect:** 0.00029 **Queries matched:** 1
B7T1P7_9SPIR FlaA (Fragment) OS=Brachyspira canis PE=4 SV=1

Observed	Mr(expt)	Mr(calc)	ppm	Start	End	Miss	Ions	Peptide
970.4528	969.4455	969.4920	-47.89	186 -	193	0	69	K.LDSLGFYR.T

No match to: 1241.6726, 1255.6830, 1625.7828, 1811.8773

2. B7T1P1 **Mass:** 24517 **Score:** 82 **Expect:** 0.00029 **Queries matched:** 1
B7T1P1_9SPIR FlaA (Fragment) OS=Brachyspira sp. SRBP205IR PE=4 SV=1

Observed	Mr(expt)	Mr(calc)	ppm	Start	End	Miss	Ions	Peptide
970.4528	969.4455	969.4920	-47.89	186 -	193	0	69	K.LDSLGFYR.T

No match to: 1241.6726, 1255.6830, 1625.7828, 1811.8773

3. B7T1P2 **Mass:** 24517 **Score:** 82 **Expect:** 0.00029 **Queries matched:** 1
B7T1P2_9SPIR FlaA (Fragment) OS=Brachyspira sp. SRBP5IR PE=4 SV=1

Observed	Mr(expt)	Mr(calc)	ppm	Start	End	Miss	Ions	Peptide
970.4528	969.4455	969.4920	-47.89	186 -	193	0	69	K.LDSLGFYR.T

No match to: 1241.6726, 1255.6830, 1625.7828, 1811.8773

4. B7T1P3 **Mass:** 24499 **Score:** 82 **Expect:** 0.00029 **Queries matched:** 1
B7T1P3_9SPIR FlaA (Fragment) OS=Brachyspira innocens PE=4 SV=1

Observed	Mr(expt)	Mr(calc)	ppm	Start - End	Miss	Ions	Peptide
970.4528	969.4455	969.4920	-47.89	186 - 193	0	69	K.LDSLGFYR.T

No match to: 1241.6726, 1255.6830, 1625.7828, 1811.8773

5. B7T1P4 **Mass:** 24426 **Score:** 82 **Expect:** 0.00029 **Queries matched:** 1
B7T1P4_9SPIR FlaA (Fragment) OS=Brachyspira innocens PE=4 SV=1

Observed	Mr(expt)	Mr(calc)	ppm	Start - End	Miss	Ions	Peptide
970.4528	969.4455	969.4920	-47.89	186 - 193	0	69	K.LDSLGFYR.T

No match to: 1241.6726, 1255.6830, 1625.7828, 1811.8773

6. B7T1P5 **Mass:** 24371 **Score:** 82 **Expect:** 0.00029 **Queries matched:** 1
B7T1P5_9SPIR FlaA (Fragment) OS=Brachyspira innocens PE=4 SV=1

Observed	Mr(expt)	Mr(calc)	ppm	Start - End	Miss	Ions	Peptide
970.4528	969.4455	969.4920	-47.89	186 - 193	0	69	K.LDSLGFYR.T

No match to: 1241.6726, 1255.6830, 1625.7828, 1811.8773

7. B7T1Q0 **Mass:** 24499 **Score:** 82 **Expect:** 0.00029 **Queries matched:** 1
B7T1Q0_9SPIR FlaA (Fragment) OS=Brachyspira alvinipulli PE=4 SV=1

Observed	Mr(expt)	Mr(calc)	ppm	Start - End	Miss	Ions	Peptide
970.4528	969.4455	969.4920	-47.89	186 - 193	0	69	K.LDSLGFYR.T

No match to: 1241.6726, 1255.6830, 1625.7828, 1811.8773

8. B7T1P6 **Mass:** 24483 **Score:** 82 **Expect:** 0.00029 **Queries matched:** 1
B7T1P6_9SPIR FlaA (Fragment) OS=Brachyspira intermedia PE=4 SV=1

Observed	Mr(expt)	Mr(calc)	ppm	Start - End	Miss	Ions	Peptide
970.4528	969.4455	969.4920	-47.89	186 - 193	0	69	K.LDSLGFYR.T

No match to: 1241.6726, 1255.6830, 1625.7828, 1811.8773

9. B7T1P9 **Mass:** 24499 **Score:** 82 **Expect:** 0.00029 **Queries matched:** 1
B7T1P9_9SPIR FlaA (Fragment) OS=Brachyspira intermedia PE=4 SV=1

Observed	Mr(expt)	Mr(calc)	ppm	Start - End	Miss	Ions	Peptide
970.4528	969.4455	969.4920	-47.89	186 - 193	0	69	K.LDSLGFYR.T

No match to: 1241.6726, 1255.6830, 1625.7828, 1811.8773

10. D5U8F3 **Mass:** 35700 **Score:** 80 **Expect:** 0.00042 **Queries matched:** 1
D5U8F3_BRAM5 Flagellar filament outer layer protein FlaA OS=Brachyspira murdochii (strain ATCC 51284 / DSM 12563 / 56-150) GN=Bmur_087

Observed	Mr(expt)	Mr(calc)	ppm	Start - End	Miss	Ions	Peptide
970.4528	969.4455	969.4920	-47.89	217 - 224	0	69	K.LDSLGFYR.T

No match to: 1241.6726, 1255.6830, 1625.7828, 1811.8773

11. J9UWR4 **Mass:** 35678 **Score:** 80 **Expect:** 0.00042 **Queries matched:** 1
J9UWR4_BRAPL Flagellar filament outer layer protein FlaA OS=Brachyspira pilosicoli B2904 GN=B2904_orf2029 PE=4 SV=1

Observed	Mr(expt)	Mr(calc)	ppm	Start - End	Miss	Ions	Peptide
970.4528	969.4455	969.4920	-47.89	217 - 224	0	69	K.LDSLGFYR.T

No match to: 1241.6726, 1255.6830, 1625.7828, 1811.8773

12. D8IBY6 **Mass:** 35707 **Score:** 80 **Expect:** 0.00042 **Queries matched:** 1
D8IBY6_BRAP9 Flagellar filament outer layer protein, FlaA OS=Brachyspira pilosicoli (strain ATCC BAA-1826 / 95/1000) GN=flaA PE=4 SV=1

Observed	Mr(expt)	Mr(calc)	ppm	Start - End	Miss	Ions	Peptide
970.4528	969.4455	969.4920	-47.89	217 - 224	0	69	K.LDSLGFYR.T

No match to: 1241.6726, 1255.6830, 1625.7828, 1811.8773

13. K0JH04 **Mass:** 35709 **Score:** 80 **Expect:** 0.00042 **Queries matched:** 1
K0JH04_BRAPL Flagellar filament outer layer protein FlaA OS=Brachyspira pilosicoli WesB GN=flaA PE=4 SV=1

Observed	Mr(expt)	Mr(calc)	ppm	Start - End	Miss	Ions	Peptide
970.4528	969.4455	969.4920	-47.89	217 - 224	0	69	K.LDSLGFYR.T

No match to: 1241.6726, 1255.6830, 1625.7828, 1811.8773

14. L8XQZ8 **Mass:** 35741 **Score:** 80 **Expect:** 0.00042 **Queries matched:** 1
L8XQZ8_9SPIR Flagellar filament outer layer protein FlaA OS=Brachyspira hampsonii 30599 GN=H263_11839 PE=4 SV=1

Observed	Mr(expt)	Mr(calc)	ppm	Start - End	Miss	Ions	Peptide
970.4528	969.4455	969.4920	-47.89	217 - 224	0	69	K.LDSLGFYR.T

No match to: 1241.6726, 1255.6830, 1625.7828, 1811.8773

15. Q5XPB3 **Mass:** 36034 **Score:** 80 **Expect:** 0.00042 **Queries matched:** 1
Q5XPB3_BRAPL FlaA1 (Fragment) OS=Brachyspira pilosicoli PE=4 SV=1

Observed	Mr(expt)	Mr(calc)	ppm	Start - End	Miss	Ions	Peptide
970.4528	969.4455	969.4920	-47.89	217 - 224	0	69	K.LDSLGFYR.T

No match to: 1241.6726, 1255.6830, 1625.7828, 1811.8773

16. P32520 **Mass:** 35964 **Score:** 80 **Expect:** 0.00042 **Queries matched:** 1
FLAA1_BRAHO Flagellar filament outer layer protein flaA1 OS=Brachyspira hyodysenteriae GN=flaA1 PE=1 SV=1

Observed	Mr(expt)	Mr(calc)	ppm	Start - End	Miss	Ions	Peptide
970.4528	969.4455	969.4920	-47.89	217 - 224	0	69	K.LDSLGFYR.T

No match to: 1241.6726, 1255.6830, 1625.7828, 1811.8773

17. A0A0H0TRE1 **Mass:** 35964 **Score:** 80 **Expect:** 0.00042 **Queries matched:** 1
A0A0H0TRE1_BRAHO Flagellar filament protein FlaA OS=Brachyspira hyodysenteriae GN=SR30_03350 PE=4 SV=1

Observed	Mr(expt)	Mr(calc)	ppm	Start - End	Miss	Ions	Peptide
970.4528	969.4455	969.4920	-47.89	217 - 224	0	69	K.LDSLGFYR.T

No match to: 1241.6726, 1255.6830, 1625.7828, 1811.8773

18. A0A0G4K8P6 **Mass:** 35920 **Score:** 80 **Expect:** 0.00042 **Queries matched:** 1

A0A0G4K8P6_9SPIR Flagellar filament outer layer protein flaA1 OS=Brachyspira suanatina GN=flaA1 PE=4 SV=1

Observed	Mr(expt)	Mr(calc)	ppm	Start	End	Miss	Ions	Peptide
970.4528	969.4455	969.4920	-47.89	217 -	224	0	69	K.LDSLGFYR.T

No match to: 1241.6726, 1255.6830, 1625.7828, 1811.8773

19. C0R0T5 **Mass:** 35964 **Score:** 80 **Expect:** 0.00042 **Queries matched:** 1

C0R0T5_BRAHW Flagellar filament outer layer protein FlaA OS=Brachyspira hyodysenteriae (strain ATCC 49526 / WA1) GN=flaA PE=4 SV=1

Observed	Mr(expt)	Mr(calc)	ppm	Start	End	Miss	Ions	Peptide
970.4528	969.4455	969.4920	-47.89	217 -	224	0	69	K.LDSLGFYR.T

No match to: 1241.6726, 1255.6830, 1625.7828, 1811.8773

20. A0A0H0V489 **Mass:** 35934 **Score:** 80 **Expect:** 0.00042 **Queries matched:** 1

A0A0H0V489_BRAHO Flagellar filament protein FlaA OS=Brachyspira hyodysenteriae GN=SZ48_11445 PE=4 SV=1

Observed	Mr(expt)	Mr(calc)	ppm	Start	End	Miss	Ions	Peptide
970.4528	969.4455	969.4920	-47.89	217 -	224	0	69	K.LDSLGFYR.T

No match to: 1241.6726, 1255.6830, 1625.7828, 1811.8773

21. A0A0H0WR23 **Mass:** 35965 **Score:** 80 **Expect:** 0.00042 **Queries matched:** 1

A0A0H0WR23_BRAHO Flagellar filament protein FlaA OS=Brachyspira hyodysenteriae GN=SZ42_05145 PE=4 SV=1

Observed	Mr(expt)	Mr(calc)	ppm	Start	End	Miss	Ions	Peptide
970.4528	969.4455	969.4920	-47.89	217 -	224	0	69	K.LDSLGFYR.T

No match to: 1241.6726, 1255.6830, 1625.7828, 1811.8773

22. G0EJW8 **Mass:** 35935 **Score:** 80 **Expect:** 0.00042 **Queries matched:** 1

G0EJW8_BRAIP Flagellar filament outer layer protein FlaA OS=Brachyspira intermedia (strain ATCC 51140 / PWS/A) GN=Bint_0608 PE=4 SV=1

Observed	Mr(expt)	Mr(calc)	ppm	Start	End	Miss	Ions	Peptide
970.4528	969.4455	969.4920	-47.89	217 -	224	0	69	K.LDSLGFYR.T

No match to: 1241.6726, 1255.6830, 1625.7828, 1811.8773

23. L0X4M2 **Mass:** 36070 **Score:** 80 **Expect:** 0.00042 **Queries matched:** 1

L0X4M2_9SPIR Flagellar filament outer layer protein FlaA OS=Brachyspira hampsonii 30446 GN=A966_04095 PE=4 SV=1

Observed	Mr(expt)	Mr(calc)	ppm	Start	End	Miss	Ions	Peptide
970.4528	969.4455	969.4920	-47.89	217 -	224	0	69	K.LDSLGFYR.T

No match to: 1241.6726, 1255.6830, 1625.7828, 1811.8773

24. R5LCF6 **Mass:** 35187 **Score:** 78 **Expect:** 0.00067 **Queries matched:** 1

R5LCF6_9SPIR Flagellar filament outer layer protein FlaA OS=Brachyspira sp. CAG:700 GN=BN758_01892 PE=4 SV=1

Observed	Mr(expt)	Mr(calc)	ppm	Start	End	Miss	Ions	Peptide
970.4528	969.4455	969.4920	-47.89	217 -	224	0	67	K.IDSIGFYR.T

No match to: 1241.6726, 1255.6830, 1625.7828, 1811.8773

25. D5U459 **Mass:** 52256 **Score:** 42 **Expect:** 2.7 **Queries matched:** 3

D5U459_BRAM5 AAA ATPase OS=Brachyspira murdochii (strain ATCC 51284 / DSM 12563 / 56-150) GN=Bmur_2166 PE=4 SV=1

Observed	Mr(expt)	Mr(calc)	ppm	Start	End	Miss	Ions	Peptide
1241.6726	1240.6653	1240.6088	45.6	163 -	172	0	---	K.DNYINQSLFK.S
1625.7828	1624.7755	1624.8283	-32.48	112 -	125	1	---	K.DNMILEFGFKLNGK.N
1811.8773	1810.8700	1810.8155	30.1	68 -	82	1	---	R.ISDFDKMINNNENNK.S + Oxidation (M)

No match to: 970.4528, 1255.6830

Search Parameters

```
Type of search         : Sequence Query
Enzyme                 : Trypsin
Fixed modifications    : Carbamidomethyl (C)
Variable modifications : Oxidation (M)
Mass values            : Monoisotopic
Protein Mass           : Unrestricted
Peptide Mass Tolerance : ± 50 ppm
Fragment Mass Tolerance: ± 0.5 Da
Max Missed Cleavages   : 1
Instrument type        : MALDI-TOF-TOF
Query1 (970.4528,1+)   : Locus:23.1.1.211170.56628
Query2 (1241.6726,1+)  : Locus:23.1.1.211170.56626
Query3 (1255.6830,1+)  : Locus:23.1.1.211170.56627
Query4 (1625.7828,1+)  : Locus:23.1.1.211170.56625
Query5 (1811.8773,1+)  : Locus:23.1.1.211170.56629
```

Mascot: http://www.matrixscience.com/

(MATRIX SCIENCE) Mascot Search Results

```
User          :
Email         :
Search title  : F:\ROD_inmunoproteoma\MALDI\160223_2016_561_562_ROD\ppw_G7_145631468214.txt
MS data file  : F:\ROD_inmunoproteoma\MALDI\160223_2016_561_562_ROD\ppw_G7_145631468214.txt
Database      : Brachyspiraceae Brachyspiraceae_20160127-151122 (40573 sequences; 13470793 residues)
Timestamp     : 24 Feb 2016 at 12:02:58 GMT
Warning       : A Peptide summary report will usually give a much clearer picture of MS/MS search results.
Top Score     : 368 for J9UWR4, J9UWR4_BRAPL Flagellar filament outer layer protein FlaA OS=Brachyspira pilosicoli B2904 GN=B2904_orf2029
```

Probability Based Mowse Score

Protein score is -10*Log(P), where P is the probability that the observed match is a random event.
Protein scores greater than 59 are significant (p<0.05).
Protein scores are derived from ions scores as a non-probabilistic basis for ranking protein hits.

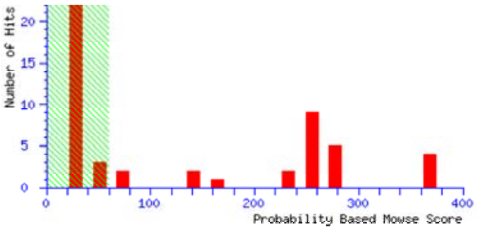

Protein Summary Report

Format As | Protein Summary (deprecated) | Help

Significance threshold p< 0.05 | Max. number of hits AUTO

Standard scoring ◉ MudPIT scoring ○ Ions score or expect cut-off 0 | Show sub-sets 0

Show pop-ups ◉ Suppress pop-ups ○ Sort unassigned Decreasing Score | Require bold red ☐

Re-Search All | Search Unmatched

Index

	Accession	Mass	Score	Description
1.	J9UWR4	35678	368	J9UWR4_BRAPL Flagellar filament outer layer protein FlaA OS=Brachyspira pilosicoli B2904 GN=B2904_orf2029 PE=4
2.	D8IBY6	35707	368	D8IBY6_BRAP9 Flagellar filament outer layer protein, FlaA OS=Brachyspira pilosicoli (strain ATCC BAA-1826 / 95
3.	K0JH04	35709	368	K0JH04_BRAPL Flagellar filament outer layer protein FlaA OS=Brachyspira pilosicoli WesB GN=flaA PE=4 SV=1
4.	Q5XPB3	36034	368	Q5XPB3_BRAPL FlaA1 (Fragment) OS=Brachyspira pilosicoli PE=4 SV=1
5.	B7T1P1	24517	269	B7T1P1_9SPIR FlaA (Fragment) OS=Brachyspira sp. SRBP205IR PE=4 SV=1
6.	B7T1P2	24517	269	B7T1P2_9SPIR FlaA (Fragment) OS=Brachyspira sp. SRBP5IR PE=4 SV=1
7.	B7T1P3	24499	269	B7T1P3_9SPIR FlaA (Fragment) OS=Brachyspira innocens PE=4 SV=1
8.	B7T1Q0	24499	269	B7T1Q0_9SPIR FlaA (Fragment) OS=Brachyspira alvinipulli PE=4 SV=1
9.	B7T1P9	24499	269	B7T1P9_9SPIR FlaA (Fragment) OS=Brachyspira intermedia PE=4 SV=1
10.	R5LCF6	35187	264	R5LCF6_9SPIR Flagellar filament outer layer protein FlaA OS=Brachyspira sp. CAG:700 GN=BN758_01892 PE=4 SV=1
11.	L8XQZ8	35741	264	L8XQZ8_9SPIR Flagellar filament outer layer protein FlaA OS=Brachyspira hampsonii 30599 GN=H263_11839 PE=4 SV=
12.	P32520	35964	264	FLAA1_BRAHO Flagellar filament outer layer protein flaA1 OS=Brachyspira hyodysenteriae GN=flaA1 PE=1 SV=1
13.	A0A0H0TRE1	35964	264	A0A0H0TRE1_BRAHO Flagellar filament protein FlaA OS=Brachyspira hyodysenteriae GN=SR30_03350 PE=4 SV=1
14.	A0A0G4K8P6	35920	264	A0A0G4K8P6_9SPIR Flagellar filament outer layer protein flaA1 OS=Brachyspira suanatina GN=flaA1 PE=4 SV=1
15.	C0R0T5	35964	264	C0R0T5_BRAHW Flagellar filament outer layer protein FlaA OS=Brachyspira hyodysenteriae (strain ATCC 49526 / WA
16.	A0A0H0V489	35934	264	A0A0H0V489_BRAHO Flagellar filament protein FlaA OS=Brachyspira hyodysenteriae GN=SZ48_11445 PE=4 SV=1
17.	A0A0H0WR23	35965	264	A0A0H0WR23_BRAHO Flagellar filament protein FlaA OS=Brachyspira hyodysenteriae GN=SZ42_05145 PE=4 SV=1
18.	G0EJW8	35935	264	G0EJW8_BRAIP Flagellar filament outer layer protein FlaA OS=Brachyspira intermedia (strain ATCC 51140 / PWS/A)
19.	B7T1P4	24426	231	B7T1P4_9SPIR FlaA (Fragment) OS=Brachyspira innocens PE=4 SV=1
20.	B7T1P5	24371	231	B7T1P5_9SPIR FlaA (Fragment) OS=Brachyspira innocens PE=4 SV=1
21.	D5U8F3	35700	153	D5U8F3_BRAM5 Flagellar filament outer layer protein FlaA OS=Brachyspira murdochii (strain ATCC 51284 / DSM 125
22.	B7T1P7	24514	149	B7T1P7_9SPIR FlaA (Fragment) OS=Brachyspira canis PE=4 SV=1
23.	B7T1P6	24483	149	B7T1P6_9SPIR FlaA (Fragment) OS=Brachyspira intermedia PE=4 SV=1
24.	L0X4M2	36070	76	L0X4M2_9SPIR Flagellar filament outer layer protein FlaA OS=Brachyspira hampsonii 30446 GN=A966_04095 PE=4 SV=
25.	K0JLE6	27960	64	K0JLE6_BRAPL Transcriptional regulator, GntR family OS=Brachyspira pilosicoli WesB GN=gntR2 PE=4 SV=1
26.	J9USH8	28003	51	J9USH8_BRAPL GntR family transcriptional regulator OS=Brachyspira pilosicoli B2904 GN=gntR PE=4 SV=1

Results List

1. J9UWR4 **Mass:** 35678 **Score:** 368 **Expect:** 6.4e-33 **Queries matched:** 4
J9UWR4_BRAPL Flagellar filament outer layer protein FlaA OS=Brachyspira pilosicoli B2904 GN=B2904_orf2029 PE=4 SV=1

Observed	Mr(expt)	Mr(calc)	ppm	Start - End	Miss	Ions	Peptide
970.5283	969.5210	969.4920	29.9	217 - 224	0	65	K.LDSLGFYR.T
1054.5616	1053.5543	1053.5243	28.5	145 - 153	0	55	K.SISSWVYGR.N
1569.8820	1568.8747	1568.8270	30.4	279 - 291	1	83	R.VREAAELQQLEQR.R
1625.9183	1624.9110	1624.8647	28.5	211 - 224	1	99	K.MIPSVKLDSLGFYR.T

No match to: 1577.9104

2. D8IBY6 **Mass:** 35707 **Score:** 368 **Expect:** 6.4e-33 **Queries matched:** 4
D8IBY6_BRAP9 Flagellar filament outer layer protein, FlaA OS=Brachyspira pilosicoli (strain ATCC BAA-1826 / 95/1000) GN=flaA PE=4 SV=1

Observed	Mr(expt)	Mr(calc)	ppm	Start - End	Miss	Ions	Peptide
970.5283	969.5210	969.4920	29.9	217 - 224	0	65	K.LDSLGFYR.T
1054.5616	1053.5543	1053.5243	28.5	145 - 153	0	55	K.SISSWVYGR.N
1569.8820	1568.8747	1568.8270	30.4	279 - 291	1	83	R.VREAAELQQLEQR.R

Observed	Mr(expt)	Mr(calc)	ppm	Start	End	Miss	Ions	Peptide
1625.9183	1624.9110	1624.8647	28.5	211 -	224	1	99	K.MIPSVKLDSLGFYR.T

No match to: 1577.9104

3. K0JH04 **Mass:** 35709 **Score:** 368 **Expect:** 6.4e-33 **Queries matched:** 4
K0JH04_BRAPL Flagellar filament outer layer protein FlaA OS=Brachyspira pilosicoli WesB GN=flaA PE=4 SV=1

Observed	Mr(expt)	Mr(calc)	ppm	Start	End	Miss	Ions	Peptide
970.5283	969.5210	969.4920	29.9	217 -	224	0	65	K.LDSLGFYR.T
1054.5616	1053.5543	1053.5243	28.5	145 -	153	0	55	K.SISSWVYGR.N
1569.8820	1568.8747	1568.8270	30.4	279 -	291	1	83	R.VREAAELQQLEQR.R
1625.9183	1624.9110	1624.8647	28.5	211 -	224	1	99	K.MIPSVKLDSLGFYR.T

No match to: 1577.9104

4. Q5XPB3 **Mass:** 36034 **Score:** 368 **Expect:** 6.4e-33 **Queries matched:** 4
Q5XPB3_BRAPL FlaA1 (Fragment) OS=Brachyspira pilosicoli PE=4 SV=1

Observed	Mr(expt)	Mr(calc)	ppm	Start	End	Miss	Ions	Peptide
970.5283	969.5210	969.4920	29.9	217 -	224	0	65	K.LDSLGFYR.T
1054.5616	1053.5543	1053.5243	28.5	145 -	153	0	55	K.SISSWVYGR.N
1569.8820	1568.8747	1568.8270	30.4	279 -	291	1	83	R.VREAAELQQLEQR.R
1625.9183	1624.9110	1624.8647	28.5	211 -	224	1	99	K.MIPSVKLDSLGFYR.T

No match to: 1577.9104

5. B7T1P1 **Mass:** 24517 **Score:** 269 **Expect:** 5.1e-23 **Queries matched:** 3
B7T1P1_9SPIR FlaA (Fragment) OS=Brachyspira sp. SRBP205IR PE=4 SV=1

Observed	Mr(expt)	Mr(calc)	ppm	Start	End	Miss	Ions	Peptide
970.5283	969.5210	969.4920	29.9	186 -	193	0	65	K.LDSLGFYR.T
1054.5616	1053.5543	1053.5243	28.5	114 -	122	0	55	K.SISSWVYGR.N
1625.9183	1624.9110	1624.8647	28.5	180 -	193	1	99	K.MIPSVKLDSLGFYR.T

No match to: 1569.8820, 1577.9104

6. B7T1P2 **Mass:** 24517 **Score:** 269 **Expect:** 5.1e-23 **Queries matched:** 3
B7T1P2_9SPIR FlaA (Fragment) OS=Brachyspira sp. SRBP5IR PE=4 SV=1

Observed	Mr(expt)	Mr(calc)	ppm	Start	End	Miss	Ions	Peptide
970.5283	969.5210	969.4920	29.9	186 -	193	0	65	K.LDSLGFYR.T
1054.5616	1053.5543	1053.5243	28.5	114 -	122	0	55	K.SISSWVYGR.N
1625.9183	1624.9110	1624.8647	28.5	180 -	193	1	99	K.MIPSVKLDSLGFYR.T

No match to: 1569.8820, 1577.9104

7. B7T1P3 **Mass:** 24499 **Score:** 269 **Expect:** 5.1e-23 **Queries matched:** 3
B7T1P3_9SPIR FlaA (Fragment) OS=Brachyspira innocens PE=4 SV=1

Observed	Mr(expt)	Mr(calc)	ppm	Start	End	Miss	Ions	Peptide
970.5283	969.5210	969.4920	29.9	186 -	193	0	65	K.LDSLGFYR.T
1054.5616	1053.5543	1053.5243	28.5	114 -	122	0	55	K.SISSWVYGR.N
1625.9183	1624.9110	1624.8647	28.5	180 -	193	1	99	R.MIPSVKLDSLGFYR.T

No match to: 1569.8820, 1577.9104

8. B7T1Q0 **Mass:** 24499 **Score:** 269 **Expect:** 5.1e-23 **Queries matched:** 3
B7T1Q0_9SPIR FlaA (Fragment) OS=Brachyspira alvinipulli PE=4 SV=1

Observed	Mr(expt)	Mr(calc)	ppm	Start	End	Miss	Ions	Peptide
970.5283	969.5210	969.4920	29.9	186 -	193	0	65	K.LDSLGFYR.T
1054.5616	1053.5543	1053.5243	28.5	114 -	122	0	55	K.SISSWVYGR.N
1625.9183	1624.9110	1624.8647	28.5	180 -	193	1	99	R.MIPSVKLDSLGFYR.T

No match to: 1569.8820, 1577.9104

9. B7T1P9 **Mass:** 24499 **Score:** 269 **Expect:** 5.1e-23 **Queries matched:** 3
B7T1P9_9SPIR FlaA (Fragment) OS=Brachyspira intermedia PE=4 SV=1

Observed	Mr(expt)	Mr(calc)	ppm	Start	End	Miss	Ions	Peptide
970.5283	969.5210	969.4920	29.9	186 -	193	0	65	K.LDSLGFYR.T
1054.5616	1053.5543	1053.5243	28.5	114 -	122	0	55	K.SISSWVYGR.N
1625.9183	1624.9110	1624.8647	28.5	180 -	193	1	99	R.MIPSVKLDSLGFYR.T

No match to: 1569.8820, 1577.9104

10. R5LCF6 **Mass:** 35187 **Score:** 264 **Expect:** 1.6e-22 **Queries matched:** 3
R5LCF6_9SPIR Flagellar filament outer layer protein FlaA OS=Brachyspira sp. CAG:700 GN=BN758_01892 PE=4 SV=1

Observed	Mr(expt)	Mr(calc)	ppm	Start	End	Miss	Ions	Peptide
970.5283	969.5210	969.4920	29.9	217 -	224	0	65	K.IDSIGFYR.T
1054.5616	1053.5543	1053.5243	28.5	145 -	153	0	55	R.SISSWVYGR.N
1625.9183	1624.9110	1624.8647	28.5	211 -	224	1	99	K.LMPSVKIDSIGFYR.T

No match to: 1569.8820, 1577.9104

11. L8XQZ8 **Mass:** 35741 **Score:** 264 **Expect:** 1.6e-22 **Queries matched:** 3
L8XQZ8_9SPIR Flagellar filament outer layer protein FlaA OS=Brachyspira hampsonii 30599 GN=H263_11839 PE=4 SV=1

Observed	Mr(expt)	Mr(calc)	ppm	Start	End	Miss	Ions	Peptide
970.5283	969.5210	969.4920	29.9	217 -	224	0	65	K.LDSLGFYR.T
1054.5616	1053.5543	1053.5243	28.5	145 -	153	0	55	K.SISSWVYGR.N
1625.9183	1624.9110	1624.8647	28.5	211 -	224	1	99	R.MIPSVKLDSLGFYR.T

No match to: 1569.8820, 1577.9104

12. P32520 **Mass:** 35964 **Score:** 264 **Expect:** 1.6e-22 **Queries matched:** 3
FLAA1_BRAHO Flagellar filament outer layer protein flaA1 OS=Brachyspira hyodysenteriae GN=flaA1 PE=1 SV=1

Observed	Mr(expt)	Mr(calc)	ppm	Start	End	Miss	Ions	Peptide
970.5283	969.5210	969.4920	29.9	217 -	224	0	65	K.LDSLGFYR.T
1054.5616	1053.5543	1053.5243	28.5	145 -	153	0	55	K.SISSWVYGR.N
1625.9183	1624.9110	1624.8647	28.5	211 -	224	1	99	R.MIPSVKLDSLGFYR.T

No match to: 1569.8820, 1577.9104

13. A0A0H0TRE1 **Mass:** 35964 **Score:** 264 **Expect:** 1.6e-22 **Queries matched:** 3

A0A0H0TRE1_BRAHO Flagellar filament protein FlaA OS=Brachyspira hyodysenteriae GN=SR30_03350 PE=4 SV=1

Observed	Mr(expt)	Mr(calc)	ppm	Start	End	Miss	Ions	Peptide
970.5283	969.5210	969.4920	29.9	217	224	0	65	K.LDSLGFYR.T
1054.5616	1053.5543	1053.5243	28.5	145	153	0	55	K.SISSWVYGR.N
1625.9183	1624.9110	1624.8647	28.5	211	224	1	99	R.MIPSVKLDSLGFYR.T

No match to: 1569.8820, 1577.9104

14. A0A0G4K8P6 **Mass:** 35920 **Score:** 264 **Expect:** 1.6e-22 **Queries matched:** 3
A0A0G4K8P6_9SPIR Flagellar filament outer layer protein flaA1 OS=Brachyspira suanatina GN=flaA1 PE=4 SV=1

Observed	Mr(expt)	Mr(calc)	ppm	Start	End	Miss	Ions	Peptide
970.5283	969.5210	969.4920	29.9	217	224	0	65	K.LDSLGFYR.T
1054.5616	1053.5543	1053.5243	28.5	145	153	0	55	K.SISSWVYGR.N
1625.9183	1624.9110	1624.8647	28.5	211	224	1	99	R.MIPSVKLDSLGFYR.T

No match to: 1569.8820, 1577.9104

15. C0R0T5 **Mass:** 35964 **Score:** 264 **Expect:** 1.6e-22 **Queries matched:** 3
C0R0T5_BRAHW Flagellar filament outer layer protein FlaA OS=Brachyspira hyodysenteriae (strain ATCC 49526 / WA1) GN=flaA PE=4 SV=1

Observed	Mr(expt)	Mr(calc)	ppm	Start	End	Miss	Ions	Peptide
970.5283	969.5210	969.4920	29.9	217	224	0	65	K.LDSLGFYR.T
1054.5616	1053.5543	1053.5243	28.5	145	153	0	55	K.SISSWVYGR.N
1625.9183	1624.9110	1624.8647	28.5	211	224	1	99	R.MIPSVKLDSLGFYR.T

No match to: 1569.8820, 1577.9104

16. A0A0H0V489 **Mass:** 35934 **Score:** 264 **Expect:** 1.6e-22 **Queries matched:** 3
A0A0H0V489_BRAHO Flagellar filament protein FlaA OS=Brachyspira hyodysenteriae GN=SZ48_11445 PE=4 SV=1

Observed	Mr(expt)	Mr(calc)	ppm	Start	End	Miss	Ions	Peptide
970.5283	969.5210	969.4920	29.9	217	224	0	65	K.LDSLGFYR.T
1054.5616	1053.5543	1053.5243	28.5	145	153	0	55	K.SISSWVYGR.N
1625.9183	1624.9110	1624.8647	28.5	211	224	1	99	R.MIPSVKLDSLGFYR.T

No match to: 1569.8820, 1577.9104

17. A0A0H0WR23 **Mass:** 35965 **Score:** 264 **Expect:** 1.6e-22 **Queries matched:** 3
A0A0H0WR23_BRAHO Flagellar filament protein FlaA OS=Brachyspira hyodysenteriae GN=SZ42_05145 PE=4 SV=1

Observed	Mr(expt)	Mr(calc)	ppm	Start	End	Miss	Ions	Peptide
970.5283	969.5210	969.4920	29.9	217	224	0	65	K.LDSLGFYR.T
1054.5616	1053.5543	1053.5243	28.5	145	153	0	55	K.SISSWVYGR.N
1625.9183	1624.9110	1624.8647	28.5	211	224	1	99	R.MIPSVKLDSLGFYR.T

No match to: 1569.8820, 1577.9104

18. G0EJW8 **Mass:** 35935 **Score:** 264 **Expect:** 1.6e-22 **Queries matched:** 3
G0EJW8_BRAIP Flagellar filament outer layer protein FlaA OS=Brachyspira intermedia (strain ATCC 51140 / PWS/A) GN=Bint_0608 PE=4 SV=1

Observed	Mr(expt)	Mr(calc)	ppm	Start	End	Miss	Ions	Peptide
970.5283	969.5210	969.4920	29.9	217	224	0	65	K.LDSLGFYR.T
1054.5616	1053.5543	1053.5243	28.5	145	153	0	55	K.SISSWVYGR.N
1625.9183	1624.9110	1624.8647	28.5	211	224	1	99	R.MIPSVKLDSLGFYR.T

No match to: 1569.8820, 1577.9104

19. B7T1P4 **Mass:** 24426 **Score:** 231 **Expect:** 3.2e-19 **Queries matched:** 3
B7T1P4_9SPIR FlaA (Fragment) OS=Brachyspira innocens PE=4 SV=1

Observed	Mr(expt)	Mr(calc)	ppm	Start	End	Miss	Ions	Peptide
970.5283	969.5210	969.4920	29.9	186	193	0	65	K.LDSLGFYR.T
1054.5616	1053.5543	1053.5243	28.5	114	122	0	55	K.SISSWVYGR.N
1625.9183	1624.9110	1624.8647	28.5	180	193	1	61	R.MVPSIKLDSLGFYR.T

No match to: 1569.8820, 1577.9104

20. B7T1P5 **Mass:** 24371 **Score:** 231 **Expect:** 3.2e-19 **Queries matched:** 3
B7T1P5_9SPIR FlaA (Fragment) OS=Brachyspira innocens PE=4 SV=1

Observed	Mr(expt)	Mr(calc)	ppm	Start	End	Miss	Ions	Peptide
970.5283	969.5210	969.4920	29.9	186	193	0	65	K.LDSLGFYR.T
1054.5616	1053.5543	1053.5243	28.5	114	122	0	55	K.SISSWVYGR.N
1625.9183	1624.9110	1624.8647	28.5	180	193	1	61	K.MVPSIKLDSLGFYR.T

No match to: 1569.8820, 1577.9104

21. D5U8F3 **Mass:** 35700 **Score:** 153 **Expect:** 2e-11 **Queries matched:** 2
D5U8F3_BRAM5 Flagellar filament outer layer protein FlaA OS=Brachyspira murdochii (strain ATCC 51284 / DSM 12563 / 56-150) GN=Bmur_087

Observed	Mr(expt)	Mr(calc)	ppm	Start	End	Miss	Ions	Peptide
970.5283	969.5210	969.4920	29.9	217	224	0	65	K.LDSLGFYR.T
1625.9183	1624.9110	1624.8647	28.5	211	224	1	61	R.MVPSIKLDSLGFYR.T

No match to: 1054.5616, 1569.8820, 1577.9104

22. B7T1P7 **Mass:** 24514 **Score:** 149 **Expect:** 5.1e-11 **Queries matched:** 2
B7T1P7_9SPIR FlaA (Fragment) OS=Brachyspira canis PE=4 SV=1

Observed	Mr(expt)	Mr(calc)	ppm	Start	End	Miss	Ions	Peptide
970.5283	969.5210	969.4920	29.9	186	193	0	65	K.LDSLGFYR.T
1054.5616	1053.5543	1053.5243	28.5	114	122	0	55	K.SISSWVYGR.N

No match to: 1569.8820, 1577.9104, 1625.9183

23. B7T1P6 **Mass:** 24483 **Score:** 149 **Expect:** 5.1e-11 **Queries matched:** 2
B7T1P6_9SPIR FlaA (Fragment) OS=Brachyspira intermedia PE=4 SV=1

Observed	Mr(expt)	Mr(calc)	ppm	Start	End	Miss	Ions	Peptide
970.5283	969.5210	969.4920	29.9	186	193	0	65	K.LDSLGFYR.T
1054.5616	1053.5543	1053.5243	28.5	114	122	0	55	K.SISSWVYGR.N

No match to: 1569.8820, 1577.9104, 1625.9183

24. L0X4M2 **Mass:** 36070 **Score:** 76 **Expect:** 0.0011 **Queries matched:** 1
L0X4M2_9SPIR Flagellar filament outer layer protein FlaA OS=Brachyspira hampsonii 30446 GN=A966_04095 PE=4 SV=1

Observed	Mr(expt)	Mr(calc)	ppm	Start	End	Miss	Ions	Peptide

```
      970.5283  969.5210  969.4920   29.9   217 - 224   0   65   K.LDSLGFYR.T
       No match to: 1054.5616, 1569.8820, 1577.9104, 1625.9183

25.    K0JLE6    Mass: 27960    Score: 64     Expect: 0.015  Queries matched: 3
       K0JLE6_BRAPL Transcriptional regulator, GntR family OS=Brachyspira pilosicoli WesB GN=gntR2 PE=4 SV=1
        Observed   Mr(expt)   Mr(calc)    ppm  Start    End Miss Ions  Peptide
        970.5283   969.5210   969.4879    34.1   114 - 121   1   ---   K.NHSEKDLK.L
      1569.8820  1568.8747  1568.9250  -32.03    50 -  63   1   15    K.IVSEVLSISRTPIR.E
      1577.9104  1576.9031  1576.8469    35.6   208 - 220   0   ---   K.LLPIMEMHLTHVK.D + Oxidation (M)
       No match to: 1054.5616, 1625.9183

26.    J9USH8    Mass: 28003    Score: 51     Expect: 0.35  Queries matched: 2
       J9USH8_BRAPL GntR family transcriptional regulator OS=Brachyspira pilosicoli B2904 GN=gntR PE=4 SV=1
        Observed   Mr(expt)   Mr(calc)    ppm  Start    End Miss Ions  Peptide
      1569.8820  1568.8747  1568.9250  -32.03    50 -  63   1   15    K.IVSEVLSISRTPIR.E
      1625.9183  1624.9110  1624.8896    13.2     4 -  17   1    5    K.LKNNNADNLIILDR.E
       No match to: 970.5283, 1054.5616, 1577.9104
```

Search Parameters

```
Type of search         : Sequence Query
Enzyme                 : Trypsin
Fixed modifications    : Carbamidomethyl (C)
Variable modifications : Oxidation (M)
Mass values            : Monoisotopic
Protein Mass           : Unrestricted
Peptide Mass Tolerance : ± 50 ppm
Fragment Mass Tolerance: ± 0.5 Da
Max Missed Cleavages   : 1
Instrument type        : MALDI-TOF-TOF
Query1 (970.5283,1+)   : Locus:15.1.1.213514.57308
Query2 (1054.5616,1+)  : Locus:15.1.1.213514.57309
Query3 (1569.8820,1+)  : Locus:15.1.1.213514.57307
Query4 (1577.9104,1+)  : Locus:15.1.1.213514.57310
Query5 (1625.9183,1+)  : Locus:15.1.1.213514.57306
```

Mascot: http://www.matrixscience.com/

Mascot Search Results

```
User          :
Email         :
Search title  : F:\ROD_inmunoproteoma\MALDI\ppw_2016_562_563_ROD\ppw_G13_145397048000.txt
MS data file  : F:\ROD_inmunoproteoma\MALDI\ppw_2016_562_563_ROD\ppw_G13_145397048000.txt
Database      : Brachyspiraceae Brachyspiraceae_20160127-151122 (40573 sequences; 13470793 residues)
Timestamp     : 23 Feb 2016 at 16:05:02 GMT
Warning       : A Peptide summary report will usually give a much clearer picture of MS/MS search results.
Top Score     : 261 for J9UWR4, J9UWR4_BRAPL Flagellar filament outer layer protein FlaA OS=Brachyspira pilosicoli B2904 GN=B2904_orf2029
```

Probability Based Mowse Score

Protein score is -10*Log(P), where P is the probability that the observed match is a random event.
Protein scores greater than 59 are significant (p<0.05).
Protein scores are derived from ions scores as a non-probabilistic basis for ranking protein hits.

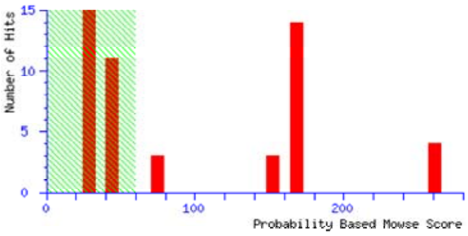

Protein Summary Report

Format As | Protein Summary (deprecated) | Help

Significance threshold p< 0.05 | Max. number of hits AUTO

Standard scoring ◉ MudPIT scoring ○ | Ions score or expect cut-off 0 | Show sub-sets 0

Show pop-ups ◉ Suppress pop-ups ○ | Sort unassigned Decreasing Score | Require bold red ☐

Re-Search All | Search Unmatched

Index

	Accession	Mass	Score	Description
1.	J9UWR4	35678	261	J9UWR4_BRAPL Flagellar filament outer layer protein FlaA OS=Brachyspira pilosicoli B2904 GN=B2904_orf2029 PE=4
2.	D8IBY6	35707	261	D8IBY6_BRAP9 Flagellar filament outer layer protein, FlaA OS=Brachyspira pilosicoli (strain ATCC BAA-1826 / 95
3.	K0JH04	35709	261	K0JH04_BRAPL Flagellar filament outer layer protein FlaA OS=Brachyspira pilosicoli WesB GN=flaA PE=4 SV=1
4.	Q5XPB3	36034	261	Q5XPB3_BRAPL FlaA1 (Fragment) OS=Brachyspira pilosicoli PE=4 SV=1
5.	B7T1P1	24517	173	B7T1P1_9SPIR FlaA (Fragment) OS=Brachyspira sp. SRBP205IR PE=4 SV=1
6.	B7T1P2	24517	173	B7T1P2_9SPIR FlaA (Fragment) OS=Brachyspira sp. SRBP5IR PE=4 SV=1
7.	B7T1P3	24499	173	B7T1P3_9SPIR FlaA (Fragment) OS=Brachyspira innocens PE=4 SV=1
8.	B7T1Q0	24499	173	B7T1Q0_9SPIR FlaA (Fragment) OS=Brachyspira alvinipulli PE=4 SV=1
9.	B7T1P9	24499	173	B7T1P9_9SPIR FlaA (Fragment) OS=Brachyspira intermedia PE=4 SV=1
10.	L8XQZ8	35741	169	L8XQZ8_9SPIR Flagellar filament outer layer protein FlaA OS=Brachyspira hampsonii 30599 GN=H263_11839 PE=4 SV=
11.	P32520	35964	169	FLAA1_BRAHO Flagellar filament outer layer protein flaA1 OS=Brachyspira hyodysenteriae GN=flaA1 PE=1 SV=1
12.	A0A0H0TRE1	35964	169	A0A0H0TRE1_BRAHO Flagellar filament protein FlaA OS=Brachyspira hyodysenteriae GN=SR30_03350 PE=4 SV=1
13.	A0A0G4K8P6	35920	169	A0A0G4K8P6_9SPIR Flagellar filament outer layer protein flaA1 OS=Brachyspira suanatina GN=flaA1 PE=4 SV=1
14.	C0R0T5	35964	169	C0R0T5_BRAHW Flagellar filament outer layer protein FlaA OS=Brachyspira hyodysenteriae (strain ATCC 49526 / WA
15.	A0A0H0V489	35934	169	A0A0H0V489_BRAHO Flagellar filament protein FlaA OS=Brachyspira hyodysenteriae GN=SZ48_11445 PE=4 SV=1
16.	A0A0H0WR23	35965	169	A0A0H0WR23_BRAHO Flagellar filament protein FlaA OS=Brachyspira hyodysenteriae GN=SZ42_05145 PE=4 SV=1
17.	G0EJW8	35935	169	G0EJW8_BRAIP Flagellar filament outer layer protein FlaA OS=Brachyspira intermedia (strain ATCC 51140 / PWS/A)
18.	R5LCF6	35187	168	R5LCF6_9SPIR Flagellar filament outer layer protein FlaA OS=Brachyspira sp. CAG:700 GN=BN758_01892 PE=4 SV=1
19.	B7T1P4	24426	152	B7T1P4_9SPIR FlaA (Fragment) OS=Brachyspira innocens PE=4 SV=1
20.	B7T1P5	24371	152	B7T1P5_9SPIR FlaA (Fragment) OS=Brachyspira innocens PE=4 SV=1
21.	D5U8F3	35700	148	D5U8F3_BRAM5 Flagellar filament outer layer protein FlaA OS=Brachyspira murdochii (strain ATCC 51284 / DSM 125
22.	B7T1P7	24514	75	B7T1P7_9SPIR FlaA (Fragment) OS=Brachyspira canis PE=4 SV=1
23.	B7T1P6	24483	75	B7T1P6_9SPIR FlaA (Fragment) OS=Brachyspira intermedia PE=4 SV=1
24.	L0X4M2	36070	74	L0X4M2_9SPIR Flagellar filament outer layer protein FlaA OS=Brachyspira hampsonii 30446 GN=A966_04095 PE=4 SV=
25.	K0JHD1	13853	49	K0JHD1_BRAPL UPF0102 protein WESB_2170 OS=Brachyspira pilosicoli WesB GN=WESB_2170 PE=3 SV=1

Results List

1. J9UWR4 **Mass:** 35678 **Score:** 261 **Expect:** 3.2e-22 **Queries matched:** 3
J9UWR4_BRAPL Flagellar filament outer layer protein FlaA OS=Brachyspira pilosicoli B2904 GN=B2904_orf2029 PE=4 SV=1

Observed	Mr(expt)	Mr(calc)	ppm	Start		End	Miss	Ions	Peptide
970.4814	969.4741	969.4920	-18.43	217	-	224	0	63	K.LDSLGFYR.T
1569.8070	1568.7997	1568.8270	-17.40	279	-	291	1	73	R.VREAAELQQLEQR.R
1625.8350	1624.8277	1624.8647	-22.75	211	-	224	1	80	K.MIPSVKLDSLGFYR.T

No match to: 1583.8242, 1597.8295

2. D8IBY6 **Mass:** 35707 **Score:** 261 **Expect:** 3.2e-22 **Queries matched:** 3
D8IBY6_BRAP9 Flagellar filament outer layer protein, FlaA OS=Brachyspira pilosicoli (strain ATCC BAA-1826 / 95/1000) GN=flaA PE=4 SV=1

Observed	Mr(expt)	Mr(calc)	ppm	Start		End	Miss	Ions	Peptide
970.4814	969.4741	969.4920	-18.43	217	-	224	0	63	K.LDSLGFYR.T
1569.8070	1568.7997	1568.8270	-17.40	279	-	291	1	73	R.VREAAELQQLEQR.R
1625.8350	1624.8277	1624.8647	-22.75	211	-	224	1	80	K.MIPSVKLDSLGFYR.T

No match to: 1583.8242, 1597.8295

3. K0JH04 **Mass:** 35709 **Score:** 261 **Expect:** 3.2e-22 **Queries matched:** 3
K0JH04_BRAPL Flagellar filament outer layer protein FlaA OS=Brachyspira pilosicoli WesB GN=flaA PE=4 SV=1

Observed	Mr(expt)	Mr(calc)	ppm	Start	End	Miss	Ions	Peptide
970.4814	969.4741	969.4920	-18.43	217	224	0	63	K.LDSLGFYR.T
1569.8070	1568.7997	1568.8270	-17.40	279	291	1	73	R.VREAAELQQLEQR.R
1625.8350	1624.8277	1624.8647	-22.75	211	224	1	80	K.MIPSVKLDSLGFYR.T

No match to: 1583.8242, 1597.8295

4. Q5XPB3 **Mass:** 36034 **Score:** 261 **Expect:** 3.2e-22 **Queries matched:** 3
Q5XPB3_BRAPL FlaA1 (Fragment) OS=Brachyspira pilosicoli PE=4 SV=1

Observed	Mr(expt)	Mr(calc)	ppm	Start	End	Miss	Ions	Peptide
970.4814	969.4741	969.4920	-18.43	217	224	0	63	K.LDSLGFYR.T
1569.8070	1568.7997	1568.8270	-17.40	279	291	1	73	R.VREAAELQQLEQR.R
1625.8350	1624.8277	1624.8647	-22.75	211	224	1	80	K.MIPSVKLDSLGFYR.T

No match to: 1583.8242, 1597.8295

5. B7T1P1 **Mass:** 24517 **Score:** 173 **Expect:** 2e-13 **Queries matched:** 2
B7T1P1_9SPIR FlaA (Fragment) OS=Brachyspira sp. SRBP205IR PE=4 SV=1

Observed	Mr(expt)	Mr(calc)	ppm	Start	End	Miss	Ions	Peptide
970.4814	969.4741	969.4920	-18.43	186	193	0	63	K.LDSLGFYR.T
1625.8350	1624.8277	1624.8647	-22.75	180	193	1	80	K.MIPSVKLDSLGFYR.T

No match to: 1569.8070, 1583.8242, 1597.8295

6. B7T1P2 **Mass:** 24517 **Score:** 173 **Expect:** 2e-13 **Queries matched:** 2
B7T1P2_9SPIR FlaA (Fragment) OS=Brachyspira sp. SRBP5IR PE=4 SV=1

Observed	Mr(expt)	Mr(calc)	ppm	Start	End	Miss	Ions	Peptide
970.4814	969.4741	969.4920	-18.43	186	193	0	63	K.LDSLGFYR.T
1625.8350	1624.8277	1624.8647	-22.75	180	193	1	80	K.MIPSVKLDSLGFYR.T

No match to: 1569.8070, 1583.8242, 1597.8295

7. B7T1P3 **Mass:** 24499 **Score:** 173 **Expect:** 2e-13 **Queries matched:** 2
B7T1P3_9SPIR FlaA (Fragment) OS=Brachyspira innocens PE=4 SV=1

Observed	Mr(expt)	Mr(calc)	ppm	Start	End	Miss	Ions	Peptide
970.4814	969.4741	969.4920	-18.43	186	193	0	63	K.LDSLGFYR.T
1625.8350	1624.8277	1624.8647	-22.75	180	193	1	80	R.MIPSVKLDSLGFYR.T

No match to: 1569.8070, 1583.8242, 1597.8295

8. B7T1Q0 **Mass:** 24499 **Score:** 173 **Expect:** 2e-13 **Queries matched:** 2
B7T1Q0_9SPIR FlaA (Fragment) OS=Brachyspira alvinipulli PE=4 SV=1

Observed	Mr(expt)	Mr(calc)	ppm	Start	End	Miss	Ions	Peptide
970.4814	969.4741	969.4920	-18.43	186	193	0	63	K.LDSLGFYR.T
1625.8350	1624.8277	1624.8647	-22.75	180	193	1	80	R.MIPSVKLDSLGFYR.T

No match to: 1569.8070, 1583.8242, 1597.8295

9. B7T1P9 **Mass:** 24499 **Score:** 173 **Expect:** 2e-13 **Queries matched:** 2
B7T1P9_9SPIR FlaA (Fragment) OS=Brachyspira intermedia PE=4 SV=1

Observed	Mr(expt)	Mr(calc)	ppm	Start	End	Miss	Ions	Peptide
970.4814	969.4741	969.4920	-18.43	186	193	0	63	K.LDSLGFYR.T
1625.8350	1624.8277	1624.8647	-22.75	180	193	1	80	R.MIPSVKLDSLGFYR.T

No match to: 1569.8070, 1583.8242, 1597.8295

10. L8XQZ8 **Mass:** 35741 **Score:** 169 **Expect:** 5.1e-13 **Queries matched:** 2
L8XQZ8_9SPIR Flagellar filament outer layer protein FlaA OS=Brachyspira hampsonii 30599 GN=H263_11839 PE=4 SV=1

Observed	Mr(expt)	Mr(calc)	ppm	Start	End	Miss	Ions	Peptide
970.4814	969.4741	969.4920	-18.43	217	224	0	63	K.LDSLGFYR.T
1625.8350	1624.8277	1624.8647	-22.75	211	224	1	80	R.MIPSVKLDSLGFYR.T

No match to: 1569.8070, 1583.8242, 1597.8295

11. P32520 **Mass:** 35964 **Score:** 169 **Expect:** 5.1e-13 **Queries matched:** 2
FLAA1_BRAHO Flagellar filament outer layer protein flaA1 OS=Brachyspira hyodysenteriae GN=flaA1 PE=1 SV=1

Observed	Mr(expt)	Mr(calc)	ppm	Start	End	Miss	Ions	Peptide
970.4814	969.4741	969.4920	-18.43	217	224	0	63	K.LDSLGFYR.T
1625.8350	1624.8277	1624.8647	-22.75	211	224	1	80	R.MIPSVKLDSLGFYR.T

No match to: 1569.8070, 1583.8242, 1597.8295

12. A0A0H0TRE1 **Mass:** 35964 **Score:** 169 **Expect:** 5.1e-13 **Queries matched:** 2
A0A0H0TRE1_BRAHO Flagellar filament protein FlaA OS=Brachyspira hyodysenteriae GN=SR30_03350 PE=4 SV=1

Observed	Mr(expt)	Mr(calc)	ppm	Start	End	Miss	Ions	Peptide
970.4814	969.4741	969.4920	-18.43	217	224	0	63	K.LDSLGFYR.T
1625.8350	1624.8277	1624.8647	-22.75	211	224	1	80	R.MIPSVKLDSLGFYR.T

No match to: 1569.8070, 1583.8242, 1597.8295

13. A0A0G4K8P6 **Mass:** 35920 **Score:** 169 **Expect:** 5.1e-13 **Queries matched:** 2
A0A0G4K8P6_9SPIR Flagellar filament outer layer protein flaA1 OS=Brachyspira suanatina GN=flaA1 PE=4 SV=1

Observed	Mr(expt)	Mr(calc)	ppm	Start	End	Miss	Ions	Peptide
970.4814	969.4741	969.4920	-18.43	217	224	0	63	K.LDSLGFYR.T
1625.8350	1624.8277	1624.8647	-22.75	211	224	1	80	R.MIPSVKLDSLGFYR.T

No match to: 1569.8070, 1583.8242, 1597.8295

14. C0R0T5 **Mass:** 35964 **Score:** 169 **Expect:** 5.1e-13 **Queries matched:** 2
C0R0T5_BRAHW Flagellar filament outer layer protein FlaA OS=Brachyspira hyodysenteriae (strain ATCC 49526 / WA1) GN=flaA PE=4 SV=1

Observed	Mr(expt)	Mr(calc)	ppm	Start	End	Miss	Ions	Peptide
970.4814	969.4741	969.4920	-18.43	217	224	0	63	K.LDSLGFYR.T
1625.8350	1624.8277	1624.8647	-22.75	211	224	1	80	R.MIPSVKLDSLGFYR.T

No match to: 1569.8070, 1583.8242, 1597.8295

15. A0A0H0V489 **Mass:** 35934 **Score:** 169 **Expect:** 5.1e-13 **Queries matched:** 2

```
       A0A0H0V489_BRAHO Flagellar filament protein FlaA OS=Brachyspira hyodysenteriae GN=SZ48_11445 PE=4 SV=1
        Observed   Mr(expt)   Mr(calc)     ppm  Start    End Miss Ions  Peptide
        970.4814   969.4741   969.4920  -18.43   217 - 224    0   63    K.LDSLGFYR.T
       1625.8350  1624.8277  1624.8647  -22.75   211 - 224    1   80    R.MIPSVKLDSLGFYR.T
       No match to: 1569.8070, 1583.8242, 1597.8295

16.    A0A0H0WR23     Mass: 35965     Score: 169     Expect: 5.1e-13  Queries matched: 2
       A0A0H0WR23_BRAHO Flagellar filament protein FlaA OS=Brachyspira hyodysenteriae GN=SZ42_05145 PE=4 SV=1
        Observed   Mr(expt)   Mr(calc)     ppm  Start    End Miss Ions  Peptide
        970.4814   969.4741   969.4920  -18.43   217 - 224    0   63    K.LDSLGFYR.T
       1625.8350  1624.8277  1624.8647  -22.75   211 - 224    1   80    R.MIPSVKLDSLGFYR.T
       No match to: 1569.8070, 1583.8242, 1597.8295

17.    G0EJW8     Mass: 35935     Score: 169     Expect: 5.1e-13  Queries matched: 2
       G0EJW8_BRAIP Flagellar filament outer layer protein FlaA OS=Brachyspira intermedia (strain ATCC 51140 / PWS/A) GN=Bint_0608 PE=4 SV=1
        Observed   Mr(expt)   Mr(calc)     ppm  Start    End Miss Ions  Peptide
        970.4814   969.4741   969.4920  -18.43   217 - 224    0   63    K.LDSLGFYR.T
       1625.8350  1624.8277  1624.8647  -22.75   211 - 224    1   80    R.MIPSVKLDSLGFYR.T
       No match to: 1569.8070, 1583.8242, 1597.8295

18.    R5LCF6     Mass: 35187     Score: 168     Expect: 6.4e-13  Queries matched: 2
       R5LCF6_9SPIR Flagellar filament outer layer protein FlaA OS=Brachyspira sp. CAG:700 GN=BN758_01892 PE=4 SV=1
        Observed   Mr(expt)   Mr(calc)     ppm  Start    End Miss Ions  Peptide
        970.4814   969.4741   969.4920  -18.43   217 - 224    0   63    K.IDSIGFYR.T
       1625.8350  1624.8277  1624.8647  -22.75   211 - 224    1   78    K.LMPSVKIDSIGFYR.T
       No match to: 1569.8070, 1583.8242, 1597.8295

19.    B7T1P4     Mass: 24426     Score: 152     Expect: 2.6e-11  Queries matched: 2
       B7T1P4_9SPIR FlaA (Fragment) OS=Brachyspira innocens PE=4 SV=1
        Observed   Mr(expt)   Mr(calc)     ppm  Start    End Miss Ions  Peptide
        970.4814   969.4741   969.4920  -18.43   186 - 193    0   63    K.LDSLGFYR.T
       1625.8350  1624.8277  1624.8647  -22.75   180 - 193    1   59    R.MVPSIKLDSLGFYR.T
       No match to: 1569.8070, 1583.8242, 1597.8295

20.    B7T1P5     Mass: 24371     Score: 152     Expect: 2.6e-11  Queries matched: 2
       B7T1P5_9SPIR FlaA (Fragment) OS=Brachyspira innocens PE=4 SV=1
        Observed   Mr(expt)   Mr(calc)     ppm  Start    End Miss Ions  Peptide
        970.4814   969.4741   969.4920  -18.43   186 - 193    0   63    K.LDSLGFYR.T
       1625.8350  1624.8277  1624.8647  -22.75   180 - 193    1   59    K.MVPSIKLDSLGFYR.T
       No match to: 1569.8070, 1583.8242, 1597.8295

21.    D5U8F3     Mass: 35700     Score: 148     Expect: 6.4e-11  Queries matched: 2
       D5U8F3_BRAM5 Flagellar filament outer layer protein FlaA OS=Brachyspira murdochii (strain ATCC 51284 / DSM 12563 / 56-150) GN=Bmur_087
        Observed   Mr(expt)   Mr(calc)     ppm  Start    End Miss Ions  Peptide
        970.4814   969.4741   969.4920  -18.43   217 - 224    0   63    K.LDSLGFYR.T
       1625.8350  1624.8277  1624.8647  -22.75   211 - 224    1   59    R.MVPSIKLDSLGFYR.T
       No match to: 1569.8070, 1583.8242, 1597.8295

22.    B7T1P7     Mass: 24514     Score: 75     Expect: 0.0012   Queries matched: 1
       B7T1P7_9SPIR FlaA (Fragment) OS=Brachyspira canis PE=4 SV=1
       Observed  Mr(expt)  Mr(calc)     ppm  Start    End Miss Ions  Peptide
       970.4814  969.4741  969.4920  -18.43   186 - 193    0   63    K.LDSLGFYR.T
       No match to: 1569.8070, 1583.8242, 1597.8295, 1625.8350

23.    B7T1P6     Mass: 24483     Score: 75     Expect: 0.0012   Queries matched: 1
       B7T1P6_9SPIR FlaA (Fragment) OS=Brachyspira intermedia PE=4 SV=1
       Observed  Mr(expt)  Mr(calc)     ppm  Start    End Miss Ions  Peptide
       970.4814  969.4741  969.4920  -18.43   186 - 193    0   63    K.LDSLGFYR.T
       No match to: 1569.8070, 1583.8242, 1597.8295, 1625.8350

24.    L0X4M2     Mass: 36070     Score: 74     Expect: 0.0017   Queries matched: 1
       L0X4M2_9SPIR Flagellar filament outer layer protein FlaA OS=Brachyspira hampsonii 30446 GN=A966_04095 PE=4 SV=1
       Observed  Mr(expt)  Mr(calc)     ppm  Start    End Miss Ions  Peptide
       970.4814  969.4741  969.4920  -18.43   217 - 224    0   63    K.LDSLGFYR.T
       No match to: 1569.8070, 1583.8242, 1597.8295, 1625.8350

25.    K0JHD1     Mass: 13853     Score: 49     Expect: 0.47   Queries matched: 2
       K0JHD1_BRAPL UPF0102 protein WESB_2170 OS=Brachyspira pilosicoli WesB GN=WESB_2170 PE=3 SV=1
        Observed   Mr(expt)   Mr(calc)    ppm  Start    End Miss Ions  Peptide
       1569.8070  1568.7997  1568.7947   3.22    60 -  74    1   12   R.QGSFGYAAAAITERK.K
       1597.8295  1596.8222  1596.8008   13.4    59 -  73    1    1   R.RQGSFGYAAAAITER.K
       No match to: 970.4814, 1583.8242, 1625.8350
```

Search Parameters

```
Type of search         : Sequence Query
Enzyme                 : Trypsin
Fixed modifications    : Carbamidomethyl (C)
Variable modifications : Oxidation (M)
Mass values            : Monoisotopic
Protein Mass           : Unrestricted
Peptide Mass Tolerance : ± 50 ppm
Fragment Mass Tolerance: ± 0.5 Da
Max Missed Cleavages   : 1
Instrument type        : MALDI-TOF-TOF
Query1 (970.4814,1+)   : Locus:1.1.1.211129.56519
Query2 (1569.8070,1+)  : Locus:1.1.1.211129.56517
Query3 (1583.8242,1+)  : Locus:1.1.1.211129.56515
```

```
Query4 (1597.8295,1+)  : Locus:1.1.1.211129.56518
Query5 (1625.8350,1+)  : Locus:1.1.1.211129.56516
```

**Mascot:** http://www.matrixscience.com/

Mascot Search Results

```
User          :
Email         :
Search title  : F:\ROD_inmunoproteoma\MALDI\160223_2016_561_562_ROD\ppw_F2_145631467401.txt
MS data file  : F:\ROD_inmunoproteoma\MALDI\160223_2016_561_562_ROD\ppw_F2_145631467401.txt
Database      : Brachyspiraceae Brachyspiraceae_20160127-151122 (40573 sequences; 13470793 residues)
Timestamp     : 24 Feb 2016 at 12:02:24 GMT
Warning       : A Peptide summary report will usually give a much clearer picture of MS/MS search results.
Top Score     : 75 for D8ICU0, D8ICU0_BRAP9 Variable surface protein, VspD OS=Brachyspira pilosicoli (strain ATCC BAA-1826 / 95/1000) GN=v
```

Probability Based Mowse Score

Protein score is -10*Log(P), where P is the probability that the observed match is a random event.
Protein scores greater than 59 are significant (p<0.05).
Protein scores are derived from ions scores as a non-probabilistic basis for ranking protein hits.

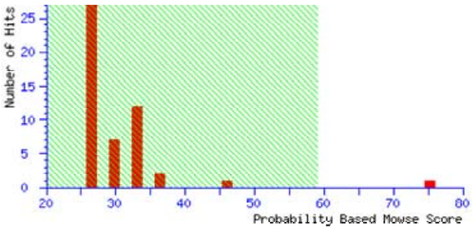

Protein Summary Report

Format As | Protein Summary (deprecated) | Help

Significance threshold p< 0.05 | Max. number of hits AUTO

Standard scoring ◉ MudPIT scoring ○ | Ions score or expect cut-off 0 | Show sub-sets 0

Show pop-ups ◉ Suppress pop-ups ○ | Sort unassigned Decreasing Score | Require bold red ☐

Re-Search All | Search Unmatched

Index

	Accession	Mass	Score	Description
1.	D8ICU0	42753	75	D8ICU0_BRAP9 Variable surface protein, VspD OS=Brachyspira pilosicoli (strain ATCC BAA-1826 / 95/1000) GN=vspD
2.	A0A0G4K6S9	33716	46	A0A0G4K6S9_9SPIR Uncharacterized protein OS=Brachyspira suanatina GN=BRSU_1392 PE=4 SV=1

Results List

1. D8ICU0 **Mass:** 42753 **Score:** 75 **Expect:** 0.0012 **Queries matched:** 1
D8ICU0_BRAP9 Variable surface protein, VspD OS=Brachyspira pilosicoli (strain ATCC BAA-1826 / 95/1000) GN=vspD PE=4 SV=1

Observed	Mr(expt)	Mr(calc)	ppm	Start	End	Miss	Ions	Peptide
1380.6721	1379.6648	1379.6690	-3.01	45 -	56	0	64	R.QMGFVLGNDMIR.G

No match to: 1278.6454, 1300.5961, 1317.6182, 1489.7308

2. A0A0G4K6S9 **Mass:** 33716 **Score:** 46 **Expect:** 0.91 **Queries matched:** 3
A0A0G4K6S9_9SPIR Uncharacterized protein OS=Brachyspira suanatina GN=BRSU_1392 PE=4 SV=1

Observed	Mr(expt)	Mr(calc)	ppm	Start	End	Miss	Ions	Peptide
1300.5961	1299.5888	1299.5760	9.85	188 -	197	0	---	K.MEEIMEMLVK.K + 3 Oxidation (M)
1380.6721	1379.6648	1379.6862	-15.51	188 -	198	1	---	K.MEEIMEMLVKK.N
1489.7308	1488.7235	1488.7784	-36.85	54 -	66	1	---	K.ETISDKQSIVDVR.A

No match to: 1278.6454, 1317.6182

Search Parameters

```
Type of search         : Sequence Query
Enzyme                 : Trypsin
Fixed modifications    : Carbamidomethyl (C)
Variable modifications : Oxidation (M)
Mass values            : Monoisotopic
Protein Mass           : Unrestricted
Peptide Mass Tolerance : ± 50 ppm
Fragment Mass Tolerance: ± 0.5 Da
Max Missed Cleavages   : 1
Instrument type        : MALDI-TOF-TOF
Query1 (1278.6454,1+)  : Locus:2.1.1.213485.57243
Query2 (1300.5961,1+)  : Locus:2.1.1.213485.57245
Query3 (1317.6182,1+)  : Locus:2.1.1.213485.57241
Query4 (1380.6721,1+)  : Locus:2.1.1.213485.57242
Query5 (1489.7308,1+)  : Locus:2.1.1.213485.57244
```

Mascot: http://www.matrixscience.com/

Mascot Search Results

```
User          :
Email         :
Search title  : F:\ROD_inmunoproteoma\MALDI\ppw_2016_561_562_563_565\ppw_H6_145467558718.txt
MS data file  : F:\ROD_inmunoproteoma\MALDI\ppw_2016_561_562_563_565\ppw_H6_145467558718.txt
Database      : Brachyspiraceae Brachyspiraceae_20160127-151122 (40573 sequences; 13470793 residues)
Timestamp     : 23 Feb 2016 at 16:36:57 GMT
Warning       : A Peptide summary report will usually give a much clearer picture of MS/MS search results.
Top Score     : 210 for D8ICU0, D8ICU0_BRAP9 Variable surface protein, VspD OS=Brachyspira pilosicoli (strain ATCC BAA-1826 / 95/1000) GN=
```

Probability Based Mowse Score

Protein score is -10*Log(P), where P is the probability that the observed match is a random event.
Protein scores greater than 59 are significant (p<0.05).
Protein scores are derived from ions scores as a non-probabilistic basis for ranking protein hits.

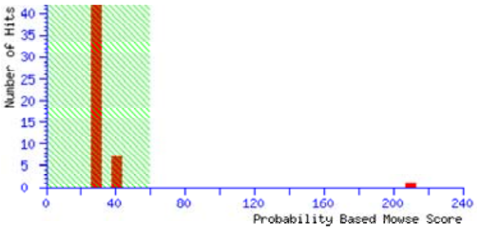

Protein Summary Report

Format As | Protein Summary (deprecated) | Help

Significance threshold p< 0.05 | Max. number of hits AUTO

Standard scoring ◉ MudPIT scoring ○ | Ions score or expect cut-off 0 | Show sub-sets 0

Show pop-ups ◉ Suppress pop-ups ○ | Sort unassigned Decreasing Score | Require bold red ☐

Re-Search All | Search Unmatched

Index

	Accession	Mass	Score	Description
1.	D8ICU0	42753	210	D8ICU0_BRAP9 Variable surface protein, VspD OS=Brachyspira pilosicoli (strain ATCC BAA-1826 / 95/1000) GN=vspD P
2.	R5LYY0	57957	42	R5LYY0_9SPIR Uncharacterized protein OS=Brachyspira sp. CAG:700 GN=BN758_00267 PE=4 SV=1

Results List

1. D8ICU0 **Mass:** 42753 **Score:** 210 **Expect:** 4.1e-17 **Queries matched:** 3
D8ICU0_BRAP9 Variable surface protein, VspD OS=Brachyspira pilosicoli (strain ATCC BAA-1826 / 95/1000) GN=vspD PE=4 SV=1

Observed	Mr(expt)	Mr(calc)	ppm	Start		End	Miss	Ions	Peptide
1380.7081	1379.7008	1379.6690	23.1	45	-	56	0	96	R.QMGFVLGNDMIR.G
1635.8785	1634.8712	1634.8385	20.0	43	-	56	1	35	R.VRQMGFVLGNDMIR.G
2407.1880	2406.1807	2406.1308	20.8	126	-	147	0	34	K.NGYGLSEHTPVLTMTAMNDSLR.I

No match to: 1278.6777, 1363.6854

2. R5LYY0 **Mass:** 57957 **Score:** 42 **Expect:** 2.4 **Queries matched:** 3
R5LYY0_9SPIR Uncharacterized protein OS=Brachyspira sp. CAG:700 GN=BN758_00267 PE=4 SV=1

Observed	Mr(expt)	Mr(calc)	ppm	Start		End	Miss	Ions	Peptide
1363.6854	1362.6781	1362.6932	-11.05	414	-	424	1	---	K.DIFEAGRELWK.Y
1635.8785	1634.8712	1634.8627	5.19	459	-	472	1	---	K.SIDETYNKLIGNLR.E
2407.1880	2406.1807	2406.2543	-30.57	34	-	56	1	---	K.LIIYINPPYAEATSGNTRAGTGK.N

No match to: 1278.6777, 1380.7081

Search Parameters

```
Type of search         : Sequence Query
Enzyme                 : Trypsin
Fixed modifications    : Carbamidomethyl (C)
Variable modifications : Oxidation (M)
Mass values            : Monoisotopic
Protein Mass           : Unrestricted
Peptide Mass Tolerance : ± 50 ppm
Fragment Mass Tolerance: ± 0.5 Da
Max Missed Cleavages   : 1
Instrument type        : MALDI-TOF-TOF
Query1 (1278.6777,1+)  : Locus:6.1.1.212740.57027
Query2 (1363.6854,1+)  : Locus:6.1.1.212740.57025
Query3 (1380.7081,1+)  : Locus:6.1.1.212740.57024
Query4 (1635.8785,1+)  : Locus:6.1.1.212740.57026
Query5 (2407.1880,1+)  : Locus:6.1.1.212740.57028
```

Mascot: http://www.matrixscience.com/

Mascot Search Results

```
User          :
Email         :
Search title  : F:\ROD_inmunoproteoma\MALDI\ppw_2014_442_ROD\ppw_G17_139601421616.txt
MS data file  : F:\ROD_inmunoproteoma\MALDI\ppw_2014_442_ROD\ppw_G17_139601421616.txt
Database      : Brachyspiraceae Brachyspiraceae_20160127-151122 (40573 sequences; 13470793 residues)
Timestamp     : 22 Feb 2016 at 15:14:29 GMT
Warning       : A Peptide summary report will usually give a much clearer picture of MS/MS search results.
Top Score     : 299 for Q26501, Q26501_BRAHO Flagellin OS=Brachyspira hyodysenteriae GN=flaB1 PE=3 SV=1
```

Probability Based Mowse Score

Protein score is -10*Log(P), where P is the probability that the observed match is a random event.
Protein scores greater than 59 are significant (p<0.05).
Protein scores are derived from ions scores as a non-probabilistic basis for ranking protein hits.

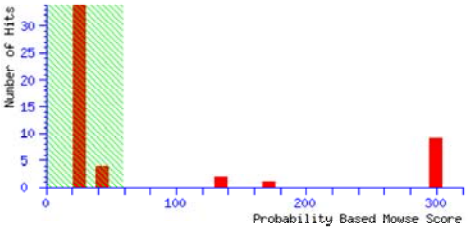

Protein Summary Report

Format As [Protein Summary (deprecated)] Help

Significance threshold p< 0.05 Max. number of hits AUTO

Standard scoring ◉ MudPIT scoring ○ Ions score or expect cut-off 0 Show sub-sets 0

Show pop-ups ◉ Suppress pop-ups ○ Sort unassigned [Decreasing Score] Require bold red ☐

Re-Search All Search Unmatched

Index

	Accession	Mass	Score	Description
1.	Q26501	31952	299	Q26501_BRAHO Flagellin OS=Brachyspira hyodysenteriae GN=flaB1 PE=3 SV=1
2.	A0A0G4K7S6	31933	299	A0A0G4K7S6_9SPIR Flagellin OS=Brachyspira suanatina GN=flaB2 PE=3 SV=1
3.	G0EKN9	31889	299	G0EKN9_BRAIP Flagellin OS=Brachyspira intermedia (strain ATCC 51140 / PWS/A) GN=Bint_2009 PE=3 SV=1
4.	J9UV65	32001	299	J9UV65_BRAPL Flagellin OS=Brachyspira pilosicoli B2904 GN=flaB2 PE=3 SV=1
5.	D8IAP2	32001	299	D8IAP2_BRAP9 Flagellin OS=Brachyspira pilosicoli (strain ATCC BAA-1826 / 95/1000) GN=flaB2 PE=3 SV=1
6.	K0JFK3	32001	299	K0JFK3_BRAPL Flagellin OS=Brachyspira pilosicoli WesB GN=flaB2-1 PE=3 SV=1
7.	D5U7D6	31944	299	D5U7D6_BRAM5 Flagellin OS=Brachyspira murdochii (strain ATCC 51284 / DSM 12563 / 56-150) GN=Bmur_0623 PE=3 SV=
8.	L8XRQ5	31949	299	L8XRQ5_9SPIR Flagellin OS=Brachyspira hampsonii 30599 GN=H263_12719 PE=3 SV=1
9.	L0X542	31951	299	L0X542_9SPIR Flagellin OS=Brachyspira hampsonii 30446 GN=A966_08614 PE=3 SV=1
10.	C0R027	28784	169	C0R027_BRAHW Flagellin OS=Brachyspira hyodysenteriae (strain ATCC 49526 / WA1) GN=BHWA1_00982 PE=3 SV=1
11.	P80158	2914	136	FLAB1_BRAHO Flagellar filament core protein flaB1 (Fragment) OS=Brachyspira hyodysenteriae GN=flaB1 PE=1 SV=1
12.	R5LFD4	32029	126	R5LFD4_9SPIR Flagellin OS=Brachyspira sp. CAG:700 GN=BN758_02216 PE=3 SV=1
13.	L0X3N1	17746	39	L0X3N1_9SPIR 30S ribosomal protein S7 OS=Brachyspira hampsonii 30446 GN=rpsG PE=3 SV=1

Results List

1. Q26501 **Mass:** 31952 **Score:** 299 **Expect:** 5.1e-26 **Queries matched:** 2
Q26501_BRAHO Flagellin OS=Brachyspira hyodysenteriae GN=flaB1 PE=3 SV=1

Observed	Mr(expt)	Mr(calc)	ppm	Start	End	Miss	Ions	Peptide
1557.8629	1556.8556	1556.8093	29.8	1 -	14	0	111	-.MVINNNISAINAQR.T
1861.9611	1860.9538	1860.8999	29.0	36 -	53	1	154	R.INQAGDDASGLAVSEKMR.T

No match to: 1774.9691

2. A0A0G4K7S6 **Mass:** 31933 **Score:** 299 **Expect:** 5.1e-26 **Queries matched:** 2
A0A0G4K7S6_9SPIR Flagellin OS=Brachyspira suanatina GN=flaB2 PE=3 SV=1

Observed	Mr(expt)	Mr(calc)	ppm	Start	End	Miss	Ions	Peptide
1557.8629	1556.8556	1556.8093	29.8	1 -	14	0	111	-.MVINNNISAINAQR.T
1861.9611	1860.9538	1860.8999	29.0	36 -	53	1	154	R.INQAGDDASGLAVSEKMR.T

No match to: 1774.9691

3. G0EKN9 **Mass:** 31889 **Score:** 299 **Expect:** 5.1e-26 **Queries matched:** 2
G0EKN9_BRAIP Flagellin OS=Brachyspira intermedia (strain ATCC 51140 / PWS/A) GN=Bint_2009 PE=3 SV=1

Observed	Mr(expt)	Mr(calc)	ppm	Start	End	Miss	Ions	Peptide
1557.8629	1556.8556	1556.8093	29.8	1 -	14	0	111	-.MVINNNISAINAQR.T
1861.9611	1860.9538	1860.8999	29.0	36 -	53	1	154	R.INQAGDDASGLAVSEKMR.T

No match to: 1774.9691

4. J9UV65 **Mass:** 32001 **Score:** 299 **Expect:** 5.1e-26 **Queries matched:** 2
J9UV65_BRAPL Flagellin OS=Brachyspira pilosicoli B2904 GN=flaB2 PE=3 SV=1

Observed	Mr(expt)	Mr(calc)	ppm	Start	End	Miss	Ions	Peptide
1557.8629	1556.8556	1556.8093	29.8	1 -	14	0	111	-.MVINNNISAINAQR.T
1861.9611	1860.9538	1860.8999	29.0	36 -	53	1	154	R.INQAGDDASGLAVSEKMR.T

```
       No match to: 1774.9691

5.     D8IAP2     Mass: 32001    Score: 299    Expect: 5.1e-26  Queries matched: 2
       D8IAP2_BRAP9 Flagellin OS=Brachyspira pilosicoli (strain ATCC BAA-1826 / 95/1000) GN=flaB2 PE=3 SV=1
        Observed    Mr(expt)   Mr(calc)    ppm  Start    End Miss Ions  Peptide
      1557.8629  1556.8556  1556.8093   29.8     1 -  14    0  111   -.MVINNNISAINAQR.T
      1861.9611  1860.9538  1860.8999   29.0    36 -  53    1  154   R.INQAGDDASGLAVSEKMR.T
       No match to: 1774.9691

6.     K0JFK3     Mass: 32001    Score: 299    Expect: 5.1e-26  Queries matched: 2
       K0JFK3_BRAPL Flagellin OS=Brachyspira pilosicoli WesB GN=flaB2-1 PE=3 SV=1
        Observed    Mr(expt)   Mr(calc)    ppm  Start    End Miss Ions  Peptide
      1557.8629  1556.8556  1556.8093   29.8     1 -  14    0  111   -.MVINNNISAINAQR.T
      1861.9611  1860.9538  1860.8999   29.0    36 -  53    1  154   R.INQAGDDASGLAVSEKMR.T
       No match to: 1774.9691

7.     D5U7D6     Mass: 31944    Score: 299    Expect: 5.1e-26  Queries matched: 2
       D5U7D6_BRAM5 Flagellin OS=Brachyspira murdochii (strain ATCC 51284 / DSM 12563 / 56-150) GN=Bmur_0623 PE=3 SV=1
        Observed    Mr(expt)   Mr(calc)    ppm  Start    End Miss Ions  Peptide
      1557.8629  1556.8556  1556.8093   29.8     1 -  14    0  111   -.MVINNNISAINAQR.T
      1861.9611  1860.9538  1860.8999   29.0    36 -  53    1  154   R.INQAGDDASGLAVSEKMR.T
       No match to: 1774.9691

8.     L8XRQ5     Mass: 31949    Score: 299    Expect: 5.1e-26  Queries matched: 2
       L8XRQ5_9SPIR Flagellin OS=Brachyspira hampsonii 30599 GN=H263_12719 PE=3 SV=1
        Observed    Mr(expt)   Mr(calc)    ppm  Start    End Miss Ions  Peptide
      1557.8629  1556.8556  1556.8093   29.8     1 -  14    0  111   -.MVINNNISAINAQR.T
      1861.9611  1860.9538  1860.8999   29.0    36 -  53    1  154   R.INQAGDDASGLAVSEKMR.T
       No match to: 1774.9691

9.     L0X542     Mass: 31951    Score: 299    Expect: 5.1e-26  Queries matched: 2
       L0X542_9SPIR Flagellin OS=Brachyspira hampsonii 30446 GN=A966_08614 PE=3 SV=1
        Observed    Mr(expt)   Mr(calc)    ppm  Start    End Miss Ions  Peptide
      1557.8629  1556.8556  1556.8093   29.8     1 -  14    0  111   -.MVINNNISAINAQR.T
      1861.9611  1860.9538  1860.8999   29.0    36 -  53    1  154   R.INQAGDDASGLAVSEKMR.T
       No match to: 1774.9691

10.    C0R027     Mass: 28784    Score: 169    Expect: 5.1e-13  Queries matched: 1
       C0R027_BRAHW Flagellin OS=Brachyspira hyodysenteriae (strain ATCC 49526 / WA1) GN=BHWA1_00982 PE=3 SV=1
        Observed    Mr(expt)   Mr(calc)    ppm  Start    End Miss Ions  Peptide
      1861.9611  1860.9538  1860.8999   29.0     8 -  25    1  154   R.INQAGDDASGLAVSEKMR.T
       No match to: 1557.8629, 1774.9691

11.    P80158     Mass: 2914     Score: 136    Expect: 1e-09  Queries matched: 1
       FLAB1_BRAHO Flagellar filament core protein flaB1 (Fragment) OS=Brachyspira hyodysenteriae GN=flaB1 PE=1 SV=1
        Observed    Mr(expt)   Mr(calc)    ppm  Start    End Miss Ions  Peptide
      1557.8629  1556.8556  1556.8093   29.8     1 -  14    0  111   -.MVINNNISAINAQR.T
       No match to: 1774.9691, 1861.9611

12.    R5LFD4     Mass: 32029    Score: 126    Expect: 1e-08  Queries matched: 1
       R5LFD4_9SPIR Flagellin OS=Brachyspira sp. CAG:700 GN=BN758_02216 PE=3 SV=1
        Observed    Mr(expt)   Mr(calc)    ppm  Start    End Miss Ions  Peptide
      1557.8629  1556.8556  1556.8093   29.8     1 -  14    0  111   -.MVINNNISAINAQR.T
       No match to: 1774.9691, 1861.9611

13.    L0X3N1     Mass: 17746    Score: 39     Expect: 4.8  Queries matched: 2
       L0X3N1_9SPIR 30S ribosomal protein S7 OS=Brachyspira hampsonii 30446 GN=rpsG PE=3 SV=1
        Observed    Mr(expt)   Mr(calc)    ppm  Start    End Miss Ions  Peptide
      1557.8629  1556.8556  1556.7908   41.6    39 -  51    1  ---   K.AENIFYKAMDLVK.Q + Oxidation (M)
      1861.9611  1860.9538  1860.9251   15.4   115 - 131    1  ---   R.SMIERLSNEIADAIDGK.G
       No match to: 1774.9691
```

Search Parameters

```
Type of search         : Sequence Query
Enzyme                 : Trypsin
Fixed modifications    : Carbamidomethyl (C)
Variable modifications : Oxidation (M)
Mass values            : Monoisotopic
Protein Mass           : Unrestricted
Peptide Mass Tolerance : ± 50 ppm
Fragment Mass Tolerance: ± 0.5 Da
Max Missed Cleavages   : 1
Instrument type        : MALDI-TOF-TOF
Query1 (1557.8629,1+)  : Locus:17.1.1.170615.52783
Query2 (1774.9691,1+)  : Locus:17.1.1.170615.52785
Query3 (1861.9611,1+)  : Locus:17.1.1.170615.52784
```

Mascot: http://www.matrixscience.com/

Mascot Search Results

**User**	:
**Email**	:
**Search title**	: F:\ROD_inmunoproteoma\MALDI\160223_2016_561_562_ROD\ppw_F1_145631467300.txt
**MS data file**	: F:\ROD_inmunoproteoma\MALDI\160223_2016_561_562_ROD\ppw_F1_145631467300.txt
**Database**	: Brachyspiraceae Brachyspiraceae_20160127-151122 (40573 sequences; 13470793 residues)
**Timestamp**	: 24 Feb 2016 at 12:02:22 GMT
**Warning**	: A Peptide summary report will usually give a much clearer picture of MS/MS search results.
**Top Score**	: 641 for R5LFD4, R5LFD4_9SPIR Flagellin OS=Brachyspira sp. CAG:700 GN=BN758_02216 PE=3 SV=1

Probability Based Mowse Score

Protein score is -10*Log(P), where P is the probability that the observed match is a random event.
Protein scores greater than 59 are significant (p<0.05).
Protein scores are derived from ions scores as a non-probabilistic basis for ranking protein hits.

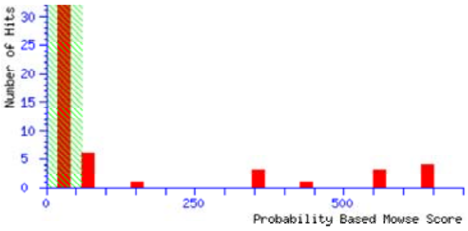

Protein Summary Report

Format As | Protein Summary (deprecated) | Help

Significance threshold p< 0.05 | Max. number of hits AUTO

Standard scoring ◉ MudPIT scoring ○ | Ions score or expect cut-off 0 | Show sub-sets 0

Show pop-ups ◉ Suppress pop-ups ○ | Sort unassigned Decreasing Score | Require bold red ☐

Re-Search All | Search Unmatched

Index

	Accession	Mass	Score	Description
1.	R5LFD4	32029	641	R5LFD4_9SPIR Flagellin OS=Brachyspira sp. CAG:700 GN=BN758_02216 PE=3 SV=1
2.	J9UV65	32001	639	J9UV65_BRAPL Flagellin OS=Brachyspira pilosicoli B2904 GN=flaB2 PE=3 SV=1
3.	D8IAP2	32001	639	D8IAP2_BRAP9 Flagellin OS=Brachyspira pilosicoli (strain ATCC BAA-1826 / 95/1000) GN=flaB2 PE=3 SV=1
4.	K0JFK3	32001	639	K0JFK3_BRAPL Flagellin OS=Brachyspira pilosicoli WesB GN=flaB2-1 PE=3 SV=1
5.	Q26501	31952	560	Q26501_BRAHO Flagellin OS=Brachyspira hyodysenteriae GN=flaB1 PE=3 SV=1
6.	A0A0G4K7S6	31933	558	A0A0G4K7S6_9SPIR Flagellin OS=Brachyspira suanatina GN=flaB2 PE=3 SV=1
7.	G0EKN9	31889	558	G0EKN9_BRAIP Flagellin OS=Brachyspira intermedia (strain ATCC 51140 / PWS/A) GN=Bint_2009 PE=3 SV=1
8.	C0R027	28784	427	C0R027_BRAHW Flagellin OS=Brachyspira hyodysenteriae (strain ATCC 49526 / WA1) GN=BHWA1_00982 PE=3 SV=1
9.	D5U7D6	31944	347	D5U7D6_BRAM5 Flagellin OS=Brachyspira murdochii (strain ATCC 51284 / DSM 12563 / 56-150) GN=Bmur_0623 PE=3 SV=
10.	L8XRQ5	31949	345	L8XRQ5_9SPIR Flagellin OS=Brachyspira hampsonii 30599 GN=H263_12719 PE=3 SV=1
11.	L0X542	31951	345	L0X542_9SPIR Flagellin OS=Brachyspira hampsonii 30446 GN=A966_08614 PE=3 SV=1
12.	P80158	2914	137	FLAB1_BRAHO Flagellar filament core protein flaB1 (Fragment) OS=Brachyspira hyodysenteriae GN=flaB1 PE=1 SV=1
13.	A0A0H0WE73	29866	72	A0A0H0WE73_BRAHO Flagellin OS=Brachyspira hyodysenteriae GN=SR30_08160 PE=3 SV=1
14.	A0A0G4K614	29880	72	A0A0G4K614_9SPIR Flagellin OS=Brachyspira suanatina GN=flaB2 PE=3 SV=1
15.	C0QYV8	29866	72	C0QYV8_BRAHW Flagellin OS=Brachyspira hyodysenteriae (strain ATCC 49526 / WA1) GN=BHWA1_00550 PE=3 SV=1
16.	A0A0H0XEC1	29841	72	A0A0H0XEC1_BRAHO Flagellin OS=Brachyspira hyodysenteriae GN=SZ44_10970 PE=3 SV=1
17.	A0A0H0V712	30193	72	A0A0H0V712_BRAHO Flagellin OS=Brachyspira hyodysenteriae GN=SZ49_03245 PE=3 SV=1
18.	A0A0G4K389	98191	57	A0A0G4K389_9SPIR Uncharacterized protein OS=Brachyspira suanatina GN=BRSU_0100 PE=4 SV=1

Results List

1. R5LFD4 **Mass:** 32029 **Score:** 641 **Expect:** 3.2e-60 **Queries matched:** 4
R5LFD4_9SPIR Flagellin OS=Brachyspira sp. CAG:700 GN=BN758_02216 PE=3 SV=1

Observed	Mr(expt)	Mr(calc)	ppm	Start		End	Miss	Ions	Peptide
1007.4768	1006.4695	1006.4832	-13.57	219	-	227	0	60	R.ADLGAYQNR.L
1557.8016	1556.7943	1556.8093	-9.60	1	-	14	0	115	-.MVINNNISAINAQR.T
2329.0261	2328.0188	2328.0548	-15.46	228	-	248	0	193	R.LEMTAQGLMVGFENMQASESR.I
2607.3367	2606.3294	2606.3598	-11.66	264	-	287	0	201	K.DQILNQANLSMLAQANQLPQGALR.L

No match to: 2343.0417

2. J9UV65 **Mass:** 32001 **Score:** 639 **Expect:** 5.1e-60 **Queries matched:** 4
J9UV65_BRAPL Flagellin OS=Brachyspira pilosicoli B2904 GN=flaB2 PE=3 SV=1

Observed	Mr(expt)	Mr(calc)	ppm	Start		End	Miss	Ions	Peptide
1007.4768	1006.4695	1006.4832	-13.57	219	-	227	0	60	R.ADLGAYQNR.L
1557.8016	1556.7943	1556.8093	-9.60	1	-	14	0	115	-.MVINNNISAINAQR.T
2329.0261	2328.0188	2328.0548	-15.46	228	-	248	0	193	R.LEMTAQGLMVGFENMQASESR.I
2607.3367	2606.3294	2606.3598	-11.66	264	-	287	0	201	K.DQILNQANLSMLAQANQLPQGALR.L

No match to: 2343.0417

3. D8IAP2 **Mass:** 32001 **Score:** 639 **Expect:** 5.1e-60 **Queries matched:** 4
D8IAP2_BRAP9 Flagellin OS=Brachyspira pilosicoli (strain ATCC BAA-1826 / 95/1000) GN=flaB2 PE=3 SV=1

Observed	Mr(expt)	Mr(calc)	ppm	Start		End	Miss	Ions	Peptide
1007.4768	1006.4695	1006.4832	-13.57	219	-	227	0	60	R.ADLGAYQNR.L

1557.8016	1556.7943	1556.8093	-9.60	1	14	0	115	-.MVINNNISAINAQR.T
2329.0261	2328.0188	2328.0548	-15.46	228	248	0	193	R.LEMTAQGLMVGFENMQASESR.I
2607.3367	2606.3294	2606.3598	-11.66	264	287	0	201	K.DQILNQANLSMLAQANQLPQGALR.L

No match to: 2343.0417

4. K0JFK3 **Mass:** 32001 **Score:** 639 **Expect:** 5.1e-60 **Queries matched:** 4
K0JFK3_BRAPL Flagellin OS=Brachyspira pilosicoli WesB GN=flaB2-1 PE=3 SV=1

Observed	Mr(expt)	Mr(calc)	ppm	Start	End	Miss	Ions	Peptide
1007.4768	1006.4695	1006.4832	-13.57	219	227	0	60	R.ADLGAYQNR.L
1557.8016	1556.7943	1556.8093	-9.60	1	14	0	115	-.MVINNNISAINAQR.T
2329.0261	2328.0188	2328.0548	-15.46	228	248	0	193	R.LEMTAQGLMVGFENMQASESR.I
2607.3367	2606.3294	2606.3598	-11.66	264	287	0	201	K.DQILNQANLSMLAQANQLPQGALR.L

No match to: 2343.0417

5. Q26501 **Mass:** 31952 **Score:** 560 **Expect:** 4.1e-52 **Queries matched:** 3
Q26501_BRAHO Flagellin OS=Brachyspira hyodysenteriae GN=flaB1 PE=3 SV=1

Observed	Mr(expt)	Mr(calc)	ppm	Start	End	Miss	Ions	Peptide
1557.8016	1556.7943	1556.8093	-9.60	1	14	0	115	-.MVINNNISAINAQR.T
2329.0261	2328.0188	2328.0548	-15.46	228	248	0	193	R.LEMTAQGLMVGFENMQASESR.I
2607.3367	2606.3294	2606.3598	-11.66	264	287	0	201	K.DQILNQANLSMLAQANQLPQGALR.L

No match to: 1007.4768, 2343.0417

6. A0A0G4K7S6 **Mass:** 31933 **Score:** 558 **Expect:** 6.4e-52 **Queries matched:** 3
A0A0G4K7S6_9SPIR Flagellin OS=Brachyspira suanatina GN=flaB2 PE=3 SV=1

Observed	Mr(expt)	Mr(calc)	ppm	Start	End	Miss	Ions	Peptide
1557.8016	1556.7943	1556.8093	-9.60	1	14	0	115	-.MVINNNISAINAQR.T
2329.0261	2328.0188	2328.0548	-15.46	228	248	0	193	R.LEMTAQGLMVGFENMQASESR.I
2607.3367	2606.3294	2606.3598	-11.66	264	287	0	201	K.DQILNQANLSMLAQANQLPQGALR.L

No match to: 1007.4768, 2343.0417

7. G0EKN9 **Mass:** 31889 **Score:** 558 **Expect:** 6.4e-52 **Queries matched:** 3
G0EKN9_BRAIP Flagellin OS=Brachyspira intermedia (strain ATCC 51140 / PWS/A) GN=Bint_2009 PE=3 SV=1

Observed	Mr(expt)	Mr(calc)	ppm	Start	End	Miss	Ions	Peptide
1557.8016	1556.7943	1556.8093	-9.60	1	14	0	115	-.MVINNNISAINAQR.T
2329.0261	2328.0188	2328.0548	-15.46	228	248	0	193	R.LEMTAQGLMVGFENMQASESR.I
2607.3367	2606.3294	2606.3598	-11.66	264	287	0	201	K.DQILNQANLSMLAQANQLPQGALR.L

No match to: 1007.4768, 2343.0417

8. C0R027 **Mass:** 28784 **Score:** 427 **Expect:** 8.1e-39 **Queries matched:** 2
C0R027_BRAHW Flagellin OS=Brachyspira hyodysenteriae (strain ATCC 49526 / WA1) GN=BHWA1_00982 PE=3 SV=1

Observed	Mr(expt)	Mr(calc)	ppm	Start	End	Miss	Ions	Peptide
2329.0261	2328.0188	2328.0548	-15.46	200	220	0	193	R.LEMTAQGLMVGFENMQASESR.I
2607.3367	2606.3294	2606.3598	-11.66	236	259	0	201	K.DQILNQANLSMLAQANQLPQGALR.L

No match to: 1007.4768, 1557.8016, 2343.0417

9. D5U7D6 **Mass:** 31944 **Score:** 347 **Expect:** 8.1e-31 **Queries matched:** 2
D5U7D6_BRAM5 Flagellin OS=Brachyspira murdochii (strain ATCC 51284 / DSM 12563 / 56-150) GN=Bmur_0623 PE=3 SV=1

Observed	Mr(expt)	Mr(calc)	ppm	Start	End	Miss	Ions	Peptide
1557.8016	1556.7943	1556.8093	-9.60	1	14	0	115	-.MVINNNISAINAQR.T
2607.3367	2606.3294	2606.3598	-11.66	264	287	0	201	K.DQILNQANLSMLAQANQLPQGALR.L

No match to: 1007.4768, 2329.0261, 2343.0417

10. L8XRQ5 **Mass:** 31949 **Score:** 345 **Expect:** 1.3e-30 **Queries matched:** 2
L8XRQ5_9SPIR Flagellin OS=Brachyspira hampsonii 30599 GN=H263_12719 PE=3 SV=1

Observed	Mr(expt)	Mr(calc)	ppm	Start	End	Miss	Ions	Peptide
1557.8016	1556.7943	1556.8093	-9.60	1	14	0	115	-.MVINNNISAINAQR.T
2607.3367	2606.3294	2606.3598	-11.66	264	287	0	201	K.DQILNQANLSMLAQANQLPQGALR.L

No match to: 1007.4768, 2329.0261, 2343.0417

11. L0X542 **Mass:** 31951 **Score:** 345 **Expect:** 1.3e-30 **Queries matched:** 2
L0X542_9SPIR Flagellin OS=Brachyspira hampsonii 30446 GN=A966_08614 PE=3 SV=1

Observed	Mr(expt)	Mr(calc)	ppm	Start	End	Miss	Ions	Peptide
1557.8016	1556.7943	1556.8093	-9.60	1	14	0	115	-.MVINNNISAINAQR.T
2607.3367	2606.3294	2606.3598	-11.66	264	287	0	201	K.DQILNQANLSMLAQANQLPQGALR.L

No match to: 1007.4768, 2329.0261, 2343.0417

12. P80158 **Mass:** 2914 **Score:** 137 **Expect:** 8.1e-10 **Queries matched:** 1
FLAB1_BRAHO Flagellar filament core protein flaB1 (Fragment) OS=Brachyspira hyodysenteriae GN=flaB1 PE=1 SV=1

Observed	Mr(expt)	Mr(calc)	ppm	Start	End	Miss	Ions	Peptide
1557.8016	1556.7943	1556.8093	-9.60	1	14	0	115	-.MVINNNISAINAQR.T

No match to: 1007.4768, 2329.0261, 2343.0417, 2607.3367

13. A0A0H0WE73 **Mass:** 29866 **Score:** 72 **Expect:** 0.0029 **Queries matched:** 1
A0A0H0WE73_BRAHO Flagellin OS=Brachyspira hyodysenteriae GN=SR30_08160 PE=3 SV=1

Observed	Mr(expt)	Mr(calc)	ppm	Start	End	Miss	Ions	Peptide
1007.4768	1006.4695	1006.5196	-49.71	196	204	1	60	R.ADLGAYKNR.F

No match to: 1557.8016, 2329.0261, 2343.0417, 2607.3367

14. A0A0G4K614 **Mass:** 29880 **Score:** 72 **Expect:** 0.0029 **Queries matched:** 1
A0A0G4K614_9SPIR Flagellin OS=Brachyspira suanatina GN=flaB2 PE=3 SV=1

Observed	Mr(expt)	Mr(calc)	ppm	Start	End	Miss	Ions	Peptide
1007.4768	1006.4695	1006.5196	-49.71	196	204	1	60	R.ADLGAYKNR.F

No match to: 1557.8016, 2329.0261, 2343.0417, 2607.3367

15. C0QYV8 **Mass:** 29866 **Score:** 72 **Expect:** 0.0029 **Queries matched:** 1
C0QYV8_BRAHW Flagellin OS=Brachyspira hyodysenteriae (strain ATCC 49526 / WA1) GN=BHWA1_00550 PE=3 SV=1

Observed	Mr(expt)	Mr(calc)	ppm	Start	End	Miss	Ions	Peptide

```
      1007.4768  1006.4695  1006.5196  -49.71   196 - 204   1   60   R.ADLGAYKNR.F
      No match to: 1557.8016, 2329.0261, 2343.0417, 2607.3367

16.   A0A0H0XEC1    Mass: 29841    Score: 72     Expect: 0.0029  Queries matched: 1
      A0A0H0XEC1_BRAHO Flagellin OS=Brachyspira hyodysenteriae GN=SZ44_10970 PE=3 SV=1
       Observed   Mr(expt)   Mr(calc)    ppm  Start   End Miss Ions  Peptide
      1007.4768  1006.4695  1006.5196  -49.71   196 - 204   1   60   R.ADLGAYKNR.F
      No match to: 1557.8016, 2329.0261, 2343.0417, 2607.3367

17.   A0A0H0V712    Mass: 30193    Score: 72     Expect: 0.0029  Queries matched: 1
      A0A0H0V712_BRAHO Flagellin OS=Brachyspira hyodysenteriae GN=SZ49_03245 PE=3 SV=1
       Observed   Mr(expt)   Mr(calc)    ppm  Start   End Miss Ions  Peptide
      1007.4768  1006.4695  1006.5196  -49.71   196 - 204   1   60   R.ADLGAYKNR.F
      No match to: 1557.8016, 2329.0261, 2343.0417, 2607.3367

18.   A0A0G4K389    Mass: 98191    Score: 57     Expect: 0.083  Queries matched: 3
      A0A0G4K389_9SPIR Uncharacterized protein OS=Brachyspira suanatina GN=BRSU_0100 PE=4 SV=1
       Observed   Mr(expt)   Mr(calc)    ppm  Start   End Miss Ions  Peptide
      1007.4768  1006.4695  1006.5195  -49.70   253 - 260   1   16   K.NALEKYNR.V
      2329.0261  2328.0188  2328.1341  -49.52   409 - 429   1   ---  K.SAFVKGDISLENTSNLDLMMK.L + Oxidation (M)
      2343.0417  2342.0344  2342.1464  -47.79   622 - 641   1    5   K.ERESLFDNYDIMAVIESLAK.Q
      No match to: 1557.8016, 2607.3367
```

Search Parameters

```
Type of search         : Sequence Query
Enzyme                 : Trypsin
Fixed modifications    : Carbamidomethyl (C)
Variable modifications : Oxidation (M)
Mass values            : Monoisotopic
Protein Mass           : Unrestricted
Peptide Mass Tolerance : ± 50 ppm
Fragment Mass Tolerance: ± 0.5 Da
Max Missed Cleavages   : 1
Instrument type        : MALDI-TOF-TOF
Query1 (1007.4768,1+)  : Locus:1.1.1.213484.57239
Query2 (1557.8016,1+)  : Locus:1.1.1.213484.57236
Query3 (2329.0261,1+)  : Locus:1.1.1.213484.57238
Query4 (2343.0417,1+)  : Locus:1.1.1.213484.57240
Query5 (2607.3367,1+)  : Locus:1.1.1.213484.57237
```

Mascot: http://www.matrixscience.com/

MATRIX SCIENCE Mascot Search Results

**User**	:	
**Email**	:	
**Search title**	:	F:\ROD_inmunoproteoma\MALDI\160223_2016_561_562_ROD\ppw_F5_145631467604.txt
**MS data file**	:	F:\ROD_inmunoproteoma\MALDI\160223_2016_561_562_ROD\ppw_F5_145631467604.txt
**Database**	:	Brachyspiraceae Brachyspiraceae_20160127-151122 (40573 sequences; 13470793 residues)
**Timestamp**	:	24 Feb 2016 at 12:02:32 GMT
**Warning**	:	A Peptide summary report will usually give a much clearer picture of MS/MS search results.
**Top Score**	:	233 for R5LFD4, R5LFD4_9SPIR Flagellin OS=Brachyspira sp. CAG:700 GN=BN758_02216 PE=3 SV=1

Probability Based Mowse Score

Protein score is -10*Log(P), where P is the probability that the observed match is a random event.
Protein scores greater than 59 are significant (p<0.05).
Protein scores are derived from ions scores as a non-probabilistic basis for ranking protein hits.

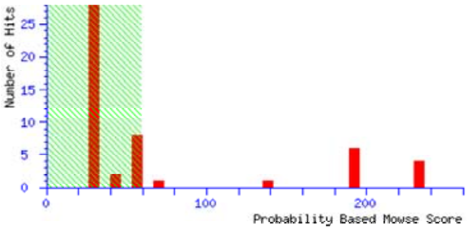

Protein Summary Report

Format As | Protein Summary (deprecated) | Help

Significance threshold p< 0.05 | Max. number of hits AUTO

Standard scoring ◉ MudPIT scoring ○ | Ions score or expect cut-off 0 | Show sub-sets 0

Show pop-ups ◉ Suppress pop-ups ○ | Sort unassigned Decreasing Score | Require bold red ☐

Re-Search All | Search Unmatched

Index

	Accession	Mass	Score	Description
1.	R5LFD4	32029	233	R5LFD4_9SPIR Flagellin OS=Brachyspira sp. CAG:700 GN=BN758_02216 PE=3 SV=1
2.	J9UV65	32001	233	J9UV65_BRAPL Flagellin OS=Brachyspira pilosicoli B2904 GN=flaB2 PE=3 SV=1
3.	D8IAP2	32001	233	D8IAP2_BRAP9 Flagellin OS=Brachyspira pilosicoli (strain ATCC BAA-1826 / 95/1000) GN=flaB2 PE=3 SV=1
4.	K0JFK3	32001	233	K0JFK3_BRAPL Flagellin OS=Brachyspira pilosicoli WesB GN=flaB2-1 PE=3 SV=1
5.	Q26501	31952	192	Q26501_BRAHO Flagellin OS=Brachyspira hyodysenteriae GN=flaB1 PE=3 SV=1
6.	A0A0G4K7S6	31933	192	A0A0G4K7S6_9SPIR Flagellin OS=Brachyspira suanatina GN=flaB2 PE=3 SV=1
7.	G0EKN9	31889	192	G0EKN9_BRAIP Flagellin OS=Brachyspira intermedia (strain ATCC 51140 / PWS/A) GN=Bint_2009 PE=3 SV=1
8.	D5U7D6	31944	192	D5U7D6_BRAM5 Flagellin OS=Brachyspira murdochii (strain ATCC 51284 / DSM 12563 / 56-150) GN=Bmur_0623 PE=3 SV=
9.	L8XRQ5	31949	192	L8XRQ5_9SPIR Flagellin OS=Brachyspira hampsonii 30599 GN=H263_12719 PE=3 SV=1
10.	L0X542	31951	192	L0X542_9SPIR Flagellin OS=Brachyspira hampsonii 30446 GN=A966_08614 PE=3 SV=1
11.	P80158	2914	137	FLAB1_BRAHO Flagellar filament core protein flaB1 (Fragment) OS=Brachyspira hyodysenteriae GN=flaB1 PE=1 SV=1
12.	Q7M132	2814	70	Q7M132_BRAHO Flagellar core protein, 34K (Fragment) OS=Brachyspira hyodysenteriae PE=1 SV=1
13.	C0R027	28784	62	C0R027_BRAHW Flagellin OS=Brachyspira hyodysenteriae (strain ATCC 49526 / WA1) GN=BHWA1_00982 PE=3 SV=1
14.	P80160	31113	60	FLAB2_BRAHO Flagellar filament core protein flaB2 OS=Brachyspira hyodysenteriae GN=flaB2 PE=1 SV=1
15.	A0A0H0X544	31129	60	A0A0H0X544_BRAHO Flagellin OS=Brachyspira hyodysenteriae GN=SU43_01870 PE=3 SV=1
16.	A0A0G4K9G4	31184	60	A0A0G4K9G4_9SPIR Flagellin OS=Brachyspira suanatina GN=flaB2 PE=3 SV=1
17.	A0A0H0ULZ7	31057	60	A0A0H0ULZ7_BRAHO Flagellin OS=Brachyspira hyodysenteriae GN=SR30_04500 PE=3 SV=1
18.	D5UB01	31218	60	D5UB01_BRAM5 Flagellin OS=Brachyspira murdochii (strain ATCC 51284 / DSM 12563 / 56-150) GN=Bmur_1792 PE=3 SV=
19.	L0X2I6	31181	60	L0X2I6_9SPIR Flagellin OS=Brachyspira hampsonii 30446 GN=A966_05778 PE=3 SV=1
20.	L8XTT2	31194	60	L8XTT2_9SPIR Flagellin OS=Brachyspira hampsonii 30599 GN=H263_08389 PE=3 SV=1
21.	L0WXW3	43707	43	L0WXW3_9SPIR Stage II sporulation protein E OS=Brachyspira hampsonii 30446 GN=A966_13275 PE=4 SV=1

Results List

1. R5LFD4 **Mass:** 32029 **Score:** 233 **Expect:** 2e-19 **Queries matched:** 3
R5LFD4_9SPIR Flagellin OS=Brachyspira sp. CAG:700 GN=BN758_02216 PE=3 SV=1

Observed	Mr(expt)	Mr(calc)	ppm	Start	End	Miss	Ions	Peptide
1007.5102	1006.5029	1006.4832	19.6	219 -	227	0	23	R.ADLGAYQNR.L
1557.8322	1556.8249	1556.8093	10.1	1 -	14	0	115	-.MVINNNISAINAQR.T
2607.3867	2606.3794	2606.3598	7.52	264 -	287	0	46	K.DQILNQANLSMLAQANQLPQGALR.L

No match to: 1509.8357, 1571.8474

2. J9UV65 **Mass:** 32001 **Score:** 233 **Expect:** 2e-19 **Queries matched:** 3
J9UV65_BRAPL Flagellin OS=Brachyspira pilosicoli B2904 GN=flaB2 PE=3 SV=1

Observed	Mr(expt)	Mr(calc)	ppm	Start	End	Miss	Ions	Peptide
1007.5102	1006.5029	1006.4832	19.6	219 -	227	0	23	R.ADLGAYQNR.L
1557.8322	1556.8249	1556.8093	10.1	1 -	14	0	115	-.MVINNNISAINAQR.T
2607.3867	2606.3794	2606.3598	7.52	264 -	287	0	46	K.DQILNQANLSMLAQANQLPQGALR.L

No match to: 1509.8357, 1571.8474

3. D8IAP2 **Mass:** 32001 **Score:** 233 **Expect:** 2e-19 **Queries matched:** 3
D8IAP2_BRAP9 Flagellin OS=Brachyspira pilosicoli (strain ATCC BAA-1826 / 95/1000) GN=flaB2 PE=3 SV=1

Observed	Mr(expt)	Mr(calc)	ppm	Start	End	Miss	Ions	Peptide
1007.5102	1006.5029	1006.4832	19.6	219 -	227	0	23	R.ADLGAYQNR.L

1557.8322	1556.8249	1556.8093	10.1	1 - 14	0	115		-.MVINNNISAINAQR.T
2607.3867	2606.3794	2606.3598	7.52	264 - 287	0	46		K.DQILNQANLSMLAQANQLPQGALR.L

No match to: 1509.8357, 1571.8474

4. K0JFK3 **Mass:** 32001 **Score:** 233 **Expect:** 2e-19 **Queries matched:** 3
K0JFK3_BRAPL Flagellin OS=Brachyspira pilosicoli WesB GN=flaB2-1 PE=3 SV=1

Observed	Mr(expt)	Mr(calc)	ppm	Start - End	Miss	Ions	Peptide
1007.5102	1006.5029	1006.4832	19.6	219 - 227	0	23	R.ADLGAYQNR.L
1557.8322	1556.8249	1556.8093	10.1	1 - 14	0	115	-.MVINNNISAINAQR.T
2607.3867	2606.3794	2606.3598	7.52	264 - 287	0	46	K.DQILNQANLSMLAQANQLPQGALR.L

No match to: 1509.8357, 1571.8474

5. Q26501 **Mass:** 31952 **Score:** 192 **Expect:** 2.6e-15 **Queries matched:** 2
Q26501_BRAHO Flagellin OS=Brachyspira hyodysenteriae GN=flaB1 PE=3 SV=1

Observed	Mr(expt)	Mr(calc)	ppm	Start - End	Miss	Ions	Peptide
1557.8322	1556.8249	1556.8093	10.1	1 - 14	0	115	-.MVINNNISAINAQR.T
2607.3867	2606.3794	2606.3598	7.52	264 - 287	0	46	K.DQILNQANLSMLAQANQLPQGALR.L

No match to: 1007.5102, 1509.8357, 1571.8474

6. A0A0G4K7S6 **Mass:** 31933 **Score:** 192 **Expect:** 2.6e-15 **Queries matched:** 2
A0A0G4K7S6_9SPIR Flagellin OS=Brachyspira suanatina GN=flaB2 PE=3 SV=1

Observed	Mr(expt)	Mr(calc)	ppm	Start - End	Miss	Ions	Peptide
1557.8322	1556.8249	1556.8093	10.1	1 - 14	0	115	-.MVINNNISAINAQR.T
2607.3867	2606.3794	2606.3598	7.52	264 - 287	0	46	K.DQILNQANLSMLAQANQLPQGALR.L

No match to: 1007.5102, 1509.8357, 1571.8474

7. G0EKN9 **Mass:** 31889 **Score:** 192 **Expect:** 2.6e-15 **Queries matched:** 2
G0EKN9_BRAIP Flagellin OS=Brachyspira intermedia (strain ATCC 51140 / PWS/A) GN=Bint_2009 PE=3 SV=1

Observed	Mr(expt)	Mr(calc)	ppm	Start - End	Miss	Ions	Peptide
1557.8322	1556.8249	1556.8093	10.1	1 - 14	0	115	-.MVINNNISAINAQR.T
2607.3867	2606.3794	2606.3598	7.52	264 - 287	0	46	K.DQILNQANLSMLAQANQLPQGALR.L

No match to: 1007.5102, 1509.8357, 1571.8474

8. D5U7D6 **Mass:** 31944 **Score:** 192 **Expect:** 2.6e-15 **Queries matched:** 2
D5U7D6_BRAM5 Flagellin OS=Brachyspira murdochii (strain ATCC 51284 / DSM 12563 / 56-150) GN=Bmur_0623 PE=3 SV=1

Observed	Mr(expt)	Mr(calc)	ppm	Start - End	Miss	Ions	Peptide
1557.8322	1556.8249	1556.8093	10.1	1 - 14	0	115	-.MVINNNISAINAQR.T
2607.3867	2606.3794	2606.3598	7.52	264 - 287	0	46	K.DQILNQANLSMLAQANQLPQGALR.L

No match to: 1007.5102, 1509.8357, 1571.8474

9. L8XRQ5 **Mass:** 31949 **Score:** 192 **Expect:** 2.6e-15 **Queries matched:** 2
L8XRQ5_9SPIR Flagellin OS=Brachyspira hampsonii 30599 GN=H263_12719 PE=3 SV=1

Observed	Mr(expt)	Mr(calc)	ppm	Start - End	Miss	Ions	Peptide
1557.8322	1556.8249	1556.8093	10.1	1 - 14	0	115	-.MVINNNISAINAQR.T
2607.3867	2606.3794	2606.3598	7.52	264 - 287	0	46	K.DQILNQANLSMLAQANQLPQGALR.L

No match to: 1007.5102, 1509.8357, 1571.8474

10. L0X542 **Mass:** 31951 **Score:** 192 **Expect:** 2.6e-15 **Queries matched:** 2
L0X542_9SPIR Flagellin OS=Brachyspira hampsonii 30446 GN=A966_08614 PE=3 SV=1

Observed	Mr(expt)	Mr(calc)	ppm	Start - End	Miss	Ions	Peptide
1557.8322	1556.8249	1556.8093	10.1	1 - 14	0	115	-.MVINNNISAINAQR.T
2607.3867	2606.3794	2606.3598	7.52	264 - 287	0	46	K.DQILNQANLSMLAQANQLPQGALR.L

No match to: 1007.5102, 1509.8357, 1571.8474

11. P80158 **Mass:** 2914 **Score:** 137 **Expect:** 8.1e-10 **Queries matched:** 1
FLAB1_BRAHO Flagellar filament core protein flaB1 (Fragment) OS=Brachyspira hyodysenteriae GN=flaB1 PE=1 SV=1

Observed	Mr(expt)	Mr(calc)	ppm	Start - End	Miss	Ions	Peptide
1557.8322	1556.8249	1556.8093	10.1	1 - 14	0	115	-.MVINNNISAINAQR.T

No match to: 1007.5102, 1509.8357, 1571.8474, 2607.3867

12. Q7M132 **Mass:** 2814 **Score:** 70 **Expect:** 0.0036 **Queries matched:** 1
Q7M132_BRAHO Flagellar core protein, 34K (Fragment) OS=Brachyspira hyodysenteriae PE=1 SV=1

Observed	Mr(expt)	Mr(calc)	ppm	Start - End	Miss	Ions	Peptide
1571.8474	1570.8401	1570.8249	9.69	1 - 14	0	48	-.MIINNNISAINAQR.T

No match to: 1007.5102, 1509.8357, 1557.8322, 2607.3867

13. C0R027 **Mass:** 28784 **Score:** 62 **Expect:** 0.028 **Queries matched:** 1
C0R027_BRAHW Flagellin OS=Brachyspira hyodysenteriae (strain ATCC 49526 / WA1) GN=BHWA1_00982 PE=3 SV=1

Observed	Mr(expt)	Mr(calc)	ppm	Start - End	Miss	Ions	Peptide
2607.3867	2606.3794	2606.3598	7.52	236 - 259	0	46	K.DQILNQANLSMLAQANQLPQGALR.L

No match to: 1007.5102, 1509.8357, 1557.8322, 1571.8474

14. P80160 **Mass:** 31113 **Score:** 60 **Expect:** 0.036 **Queries matched:** 1
FLAB2_BRAHO Flagellar filament core protein flaB2 OS=Brachyspira hyodysenteriae GN=flaB2 PE=1 SV=1

Observed	Mr(expt)	Mr(calc)	ppm	Start - End	Miss	Ions	Peptide
1571.8474	1570.8401	1570.8249	9.69	1 - 14	0	48	-.MIINNNISAINAQR.T

No match to: 1007.5102, 1509.8357, 1557.8322, 2607.3867

15. A0A0H0X544 **Mass:** 31129 **Score:** 60 **Expect:** 0.036 **Queries matched:** 1
A0A0H0X544_BRAHO Flagellin OS=Brachyspira hyodysenteriae GN=SU43_01870 PE=3 SV=1

Observed	Mr(expt)	Mr(calc)	ppm	Start - End	Miss	Ions	Peptide
1571.8474	1570.8401	1570.8249	9.69	1 - 14	0	48	-.MIINNNISAINAQR.T

No match to: 1007.5102, 1509.8357, 1557.8322, 2607.3867

16. A0A0G4K9G4 **Mass:** 31184 **Score:** 60 **Expect:** 0.036 **Queries matched:** 1
A0A0G4K9G4_9SPIR Flagellin OS=Brachyspira suanatina GN=flaB2 PE=3 SV=1

```
 Observed   Mr(expt)   Mr(calc)    ppm  Start    End Miss Ions  Peptide
1571.8474  1570.8401  1570.8249   9.69     1 -  14   0   48   -.MIINNNISAINAQR.T
No match to: 1007.5102, 1509.8357, 1557.8322, 2607.3867
```

17. A0A0H0ULZ7 **Mass:** 31057 **Score:** 60 **Expect:** 0.036 **Queries matched:** 1
A0A0H0ULZ7_BRAHO Flagellin OS=Brachyspira hyodysenteriae GN=SR30_04500 PE=3 SV=1

```
 Observed   Mr(expt)   Mr(calc)    ppm  Start    End Miss Ions  Peptide
1571.8474  1570.8401  1570.8249   9.69     1 -  14   0   48   -.MIINNNISAINAQR.T
No match to: 1007.5102, 1509.8357, 1557.8322, 2607.3867
```

18. D5UB01 **Mass:** 31218 **Score:** 60 **Expect:** 0.036 **Queries matched:** 1
D5UB01_BRAM5 Flagellin OS=Brachyspira murdochii (strain ATCC 51284 / DSM 12563 / 56-150) GN=Bmur_1792 PE=3 SV=1

```
 Observed   Mr(expt)   Mr(calc)    ppm  Start    End Miss Ions  Peptide
1571.8474  1570.8401  1570.8249   9.69     1 -  14   0   48   -.MIINNNISAINAQR.T
No match to: 1007.5102, 1509.8357, 1557.8322, 2607.3867
```

19. L0X2I6 **Mass:** 31181 **Score:** 60 **Expect:** 0.036 **Queries matched:** 1
L0X2I6_9SPIR Flagellin OS=Brachyspira hampsonii 30446 GN=A966_05778 PE=3 SV=1

```
 Observed   Mr(expt)   Mr(calc)    ppm  Start    End Miss Ions  Peptide
1571.8474  1570.8401  1570.8249   9.69     1 -  14   0   48   -.MIINNNISAINAQR.T
No match to: 1007.5102, 1509.8357, 1557.8322, 2607.3867
```

20. L8XTT2 **Mass:** 31194 **Score:** 60 **Expect:** 0.036 **Queries matched:** 1
L8XTT2_9SPIR Flagellin OS=Brachyspira hampsonii 30599 GN=H263_08389 PE=3 SV=1

```
 Observed   Mr(expt)   Mr(calc)    ppm  Start    End Miss Ions  Peptide
1571.8474  1570.8401  1570.8249   9.69     1 -  14   0   48   -.MIINNNISAINAQR.T
No match to: 1007.5102, 1509.8357, 1557.8322, 2607.3867
```

21. L0WXW3 **Mass:** 43707 **Score:** 43 **Expect:** 2.2 **Queries matched:** 3
L0WXW3_9SPIR Stage II sporulation protein E OS=Brachyspira hampsonii 30446 GN=A966_13275 PE=4 SV=1

```
 Observed   Mr(expt)   Mr(calc)    ppm  Start    End Miss Ions  Peptide
1007.5102  1006.5029  1006.5083  -5.39   231 - 239   0  ---   K.NTSPSNFIK.E
1509.8357  1508.8284  1508.7908   24.9   336 - 348   1  ---   K.MLGDTELKNIFTK.N
1571.8474  1570.8401  1570.7735   42.4   133 - 145   0  ---   K.AIESNIMYMNILK.Q + 2 Oxidation (M)
No match to: 1557.8322, 2607.3867
```

Search Parameters

```
Type of search         : Sequence Query
Enzyme                 : Trypsin
Fixed modifications    : Carbamidomethyl (C)
Variable modifications : Oxidation (M)
Mass values            : Monoisotopic
Protein Mass           : Unrestricted
Peptide Mass Tolerance : ± 50 ppm
Fragment Mass Tolerance: ± 0.5 Da
Max Missed Cleavages   : 1
Instrument type        : MALDI-TOF-TOF
Query1 (1007.5102,1+)  : Locus:5.1.1.213488.57258
Query2 (1509.8357,1+)  : Locus:5.1.1.213488.57260
Query3 (1557.8322,1+)  : Locus:5.1.1.213488.57256
Query4 (1571.8474,1+)  : Locus:5.1.1.213488.57257
Query5 (2607.3867,1+)  : Locus:5.1.1.213488.57259
```

Mascot: http://www.matrixscience.com/

Mascot Search Results

```
User          :
Email         :
Search title  : F:\ROD_inmunoproteoma\MALDI\ppw_2016_561_562_563_565\ppw_H12_145467559224.txt
MS data file  : F:\ROD_inmunoproteoma\MALDI\ppw_2016_561_562_563_565\ppw_H12_145467559224.txt
Database      : Brachyspiraceae Brachyspiraceae_20160127-151122 (40573 sequences; 13470793 residues)
Timestamp     : 23 Feb 2016 at 16:37:12 GMT
Warning       : A Peptide summary report will usually give a much clearer picture of MS/MS search results.
Top Score     : 134 for A0A0H0UW69, A0A0H0UW69_BRAHO UDP-glucose 4-epimerase OS=Brachyspira hyodysenteriae GN=SZ49_10050 PE=3 SV=1
```

Probability Based Mowse Score

Protein score is -10*Log(P), where P is the probability that the observed match is a random event.
Protein scores greater than 59 are significant (p<0.05).
Protein scores are derived from ions scores as a non-probabilistic basis for ranking protein hits.

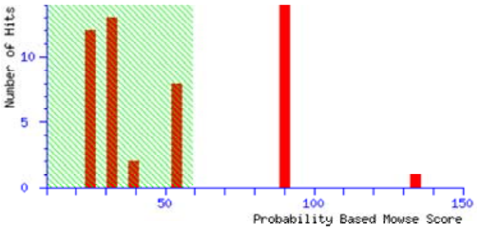

Protein Summary Report

Format As [Protein Summary (deprecated)] Help

Significance threshold p< [0.05] Max. number of hits [AUTO]

Standard scoring ◉ MudPIT scoring ○ Ions score or expect cut-off [0] Show sub-sets [0]

Show pop-ups ◉ Suppress pop-ups ○ Sort unassigned [Decreasing Score] Require bold red ☐

[Re-Search All] [Search Unmatched]

Index

	Accession	Mass	Score	Description
1.	A0A0H0UW69	36957	134	A0A0H0UW69_BRAHO UDP-glucose 4-epimerase OS=Brachyspira hyodysenteriae GN=SZ49_10050 PE=3 SV=1
2.	A0A0G4K4S5	22982	93	A0A0G4K4S5_9SPIR D-galactose-binding periplasmic protein OS=Brachyspira suanatina GN=mglB PE=4 SV=1
3.	G0EKU8	37351	91	G0EKU8_BRAIP Galactose/glucose-binding protein OS=Brachyspira intermedia (strain ATCC 51140 / PWS/A) GN=Bint_0
4.	L0X281	38186	91	L0X281_9SPIR Galactose/glucose-binding protein OS=Brachyspira hampsonii 30446 GN=A966_07749 PE=4 SV=1
5.	L8XQS2	38369	91	L8XQS2_9SPIR Galactose/glucose-binding protein OS=Brachyspira hampsonii 30599 GN=H263_14692 PE=4 SV=1
6.	A0A0H0UYR6	38326	91	A0A0H0UYR6_BRAHO Methyl-galactoside ABC transporter substrate-binding protein OS=Brachyspira hyodysenteriae GN
7.	A0A0H0TXR2	38326	91	A0A0H0TXR2_BRAHO Methyl-galactoside ABC transporter substrate-binding protein OS=Brachyspira hyodysenteriae GN
8.	A0A0H0XA19	38312	91	A0A0H0XA19_BRAHO Methyl-galactoside ABC transporter substrate-binding protein OS=Brachyspira hyodysenteriae GN
9.	C0QYC2	38326	91	C0QYC2_BRAHW Galactose/glucose-binding protein OS=Brachyspira hyodysenteriae (strain ATCC 49526 / WA1) GN=mglB
10.	A0A0H0UQZ8	38340	91	A0A0H0UQZ8_BRAHO Methyl-galactoside ABC transporter substrate-binding protein OS=Brachyspira hyodysenteriae GN
11.	A0A0H0UHD0	38300	91	A0A0H0UHD0_BRAHO Methyl-galactoside ABC transporter substrate-binding protein OS=Brachyspira hyodysenteriae GN
12.	J9UL96	38031	91	J9UL96_BRAPL Galactose glucose-binding protein OS=Brachyspira pilosicoli B2904 GN=B2904_orf2293 PE=4 SV=1
13.	D8IEY4	38017	91	D8IEY4_BRAP9 Galactose glucose binding protein OS=Brachyspira pilosicoli (strain ATCC BAA-1826 / 95/1000) GN=m
14.	K0JGQ0	38017	91	K0JGQ0_BRAPL Galactose glucose-binding protein OS=Brachyspira pilosicoli WesB GN=WESB_0562 PE=4 SV=1
15.	D5U4Z5	38254	91	D5U4Z5_BRAM5 Galactose/glucose-binding protein OS=Brachyspira murdochii (strain ATCC 51284 / DSM 12563 / 56-15
16.	A0A0H0XPY9	36921	55	A0A0H0XPY9_BRAHO UDP-glucose 4-epimerase OS=Brachyspira hyodysenteriae GN=SU45_07725 PE=3 SV=1

Results List

1. A0A0H0UW69 **Mass:** 36957 **Score:** 134 **Expect:** 1.6e-09 **Queries matched:** 2
A0A0H0UW69_BRAHO UDP-glucose 4-epimerase OS=Brachyspira hyodysenteriae GN=SZ49_10050 PE=3 SV=1

Observed	Mr(expt)	Mr(calc)	ppm	Start	End	Miss	Ions	Peptide
1280.6786	1279.6713	1279.6237	37.2	158 -	167	0	64	K.AYDFYYVALR.Y
1560.8831	1559.8758	1559.8242	33.1	268 -	281	1	43	R.KVTGHPIPAEVCPR.R

No match to: 1184.7008, 1357.6033, 2384.0322

2. A0A0G4K4S5 **Mass:** 22982 **Score:** 93 **Expect:** 2.2e-05 **Queries matched:** 1
A0A0G4K4S5_9SPIR D-galactose-binding periplasmic protein OS=Brachyspira suanatina GN=mglB PE=4 SV=1

Observed	Mr(expt)	Mr(calc)	ppm	Start	End	Miss	Ions	Peptide
1357.6033	1356.5960	1356.5445	38.0	45 -	54	0	80	R.YDDNFMSFYR.R

No match to: 1184.7008, 1280.6786, 1560.8831, 2384.0322

3. G0EKU8 **Mass:** 37351 **Score:** 91 **Expect:** 3.5e-05 **Queries matched:** 1
G0EKU8_BRAIP Galactose/glucose-binding protein OS=Brachyspira intermedia (strain ATCC 51140 / PWS/A) GN=Bint_0777 PE=4 SV=1

Observed	Mr(expt)	Mr(calc)	ppm	Start	End	Miss	Ions	Peptide
1357.6033	1356.5960	1356.5445	38.0	37 -	46	0	80	R.YDDNFMSFYR.R

No match to: 1184.7008, 1280.6786, 1560.8831, 2384.0322

4. L0X281 **Mass:** 38186 **Score:** 91 **Expect:** 3.5e-05 **Queries matched:** 1
L0X281_9SPIR Galactose/glucose-binding protein OS=Brachyspira hampsonii 30446 GN=A966_07749 PE=4 SV=1

Observed	Mr(expt)	Mr(calc)	ppm	Start	End	Miss	Ions	Peptide
1357.6033	1356.5960	1356.5445	38.0	44 -	53	0	80	R.YDDNFMSFYR.R

No match to: 1184.7008, 1280.6786, 1560.8831, 2384.0322

```
5.    L8XQS2     Mass: 38369     Score: 91     Expect: 3.5e-05  Queries matched: 1
      L8XQS2_9SPIR Galactose/glucose-binding protein OS=Brachyspira hampsonii 30599 GN=H263_14692 PE=4 SV=1
       Observed    Mr(expt)    Mr(calc)    ppm  Start    End Miss Ions  Peptide
      1357.6033  1356.5960  1356.5445   38.0     45 -   54    0   80   R.YDDNFMSFYR.R
      No match to: 1184.7008, 1280.6786, 1560.8831, 2384.0322

6.    A0A0H0UYR6     Mass: 38326     Score: 91     Expect: 3.5e-05  Queries matched: 1
      A0A0H0UYR6_BRAHO Methyl-galactoside ABC transporter substrate-binding protein OS=Brachyspira hyodysenteriae GN=SU43_00350 PE=4 SV=1
       Observed    Mr(expt)    Mr(calc)    ppm  Start    End Miss Ions  Peptide
      1357.6033  1356.5960  1356.5445   38.0     46 -   55    0   80   R.YDDNFMSFYR.R
      No match to: 1184.7008, 1280.6786, 1560.8831, 2384.0322

7.    A0A0H0TXR2     Mass: 38326     Score: 91     Expect: 3.5e-05  Queries matched: 1
      A0A0H0TXR2_BRAHO Methyl-galactoside ABC transporter substrate-binding protein OS=Brachyspira hyodysenteriae GN=SZ42_09360 PE=4 SV=1
       Observed    Mr(expt)    Mr(calc)    ppm  Start    End Miss Ions  Peptide
      1357.6033  1356.5960  1356.5445   38.0     46 -   55    0   80   R.YDDNFMSFYR.R
      No match to: 1184.7008, 1280.6786, 1560.8831, 2384.0322

8.    A0A0H0XA19     Mass: 38312     Score: 91     Expect: 3.5e-05  Queries matched: 1
      A0A0H0XA19_BRAHO Methyl-galactoside ABC transporter substrate-binding protein OS=Brachyspira hyodysenteriae GN=SU45_13560 PE=4 SV=1
       Observed    Mr(expt)    Mr(calc)    ppm  Start    End Miss Ions  Peptide
      1357.6033  1356.5960  1356.5445   38.0     46 -   55    0   80   R.YDDNFMSFYR.R
      No match to: 1184.7008, 1280.6786, 1560.8831, 2384.0322

9.    C0QYC2     Mass: 38326     Score: 91     Expect: 3.5e-05  Queries matched: 1
      C0QYC2_BRAHW Galactose/glucose-binding protein OS=Brachyspira hyodysenteriae (strain ATCC 49526 / WA1) GN=mglB PE=4 SV=1
       Observed    Mr(expt)    Mr(calc)    ppm  Start    End Miss Ions  Peptide
      1357.6033  1356.5960  1356.5445   38.0     46 -   55    0   80   R.YDDNFMSFYR.R
      No match to: 1184.7008, 1280.6786, 1560.8831, 2384.0322

10.   A0A0H0UQZ8     Mass: 38340     Score: 91     Expect: 3.5e-05  Queries matched: 1
      A0A0H0UQZ8_BRAHO Methyl-galactoside ABC transporter substrate-binding protein OS=Brachyspira hyodysenteriae GN=SR30_00780 PE=4 SV=1
       Observed    Mr(expt)    Mr(calc)    ppm  Start    End Miss Ions  Peptide
      1357.6033  1356.5960  1356.5445   38.0     46 -   55    0   80   R.YDDNFMSFYR.R
      No match to: 1184.7008, 1280.6786, 1560.8831, 2384.0322

11.   A0A0H0UHD0     Mass: 38300     Score: 91     Expect: 3.5e-05  Queries matched: 1
      A0A0H0UHD0_BRAHO Methyl-galactoside ABC transporter substrate-binding protein OS=Brachyspira hyodysenteriae GN=SZ47_08380 PE=4 SV=1
       Observed    Mr(expt)    Mr(calc)    ppm  Start    End Miss Ions  Peptide
      1357.6033  1356.5960  1356.5445   38.0     46 -   55    0   80   R.YDDNFMSFYR.R
      No match to: 1184.7008, 1280.6786, 1560.8831, 2384.0322

12.   J9UL96     Mass: 38031     Score: 91     Expect: 3.5e-05  Queries matched: 1
      J9UL96_BRAPL Galactose glucose-binding protein OS=Brachyspira pilosicoli B2904 GN=B2904_orf2293 PE=4 SV=1
       Observed    Mr(expt)    Mr(calc)    ppm  Start    End Miss Ions  Peptide
      1357.6033  1356.5960  1356.5445   38.0     46 -   55    0   80   R.YDDNFMSFYR.R
      No match to: 1184.7008, 1280.6786, 1560.8831, 2384.0322

13.   D8IEY4     Mass: 38017     Score: 91     Expect: 3.5e-05  Queries matched: 1
      D8IEY4_BRAP9 Galactose glucose binding protein OS=Brachyspira pilosicoli (strain ATCC BAA-1826 / 95/1000) GN=mglB PE=4 SV=1
       Observed    Mr(expt)    Mr(calc)    ppm  Start    End Miss Ions  Peptide
      1357.6033  1356.5960  1356.5445   38.0     46 -   55    0   80   R.YDDNFMSFYR.R
      No match to: 1184.7008, 1280.6786, 1560.8831, 2384.0322

14.   K0JGQ0     Mass: 38017     Score: 91     Expect: 3.5e-05  Queries matched: 1
      K0JGQ0_BRAPL Galactose glucose-binding protein OS=Brachyspira pilosicoli WesB GN=WESB_0562 PE=4 SV=1
       Observed    Mr(expt)    Mr(calc)    ppm  Start    End Miss Ions  Peptide
      1357.6033  1356.5960  1356.5445   38.0     46 -   55    0   80   R.YDDNFMSFYR.R
      No match to: 1184.7008, 1280.6786, 1560.8831, 2384.0322

15.   D5U4Z5     Mass: 38254     Score: 91     Expect: 3.5e-05  Queries matched: 1
      D5U4Z5_BRAM5 Galactose/glucose-binding protein OS=Brachyspira murdochii (strain ATCC 51284 / DSM 12563 / 56-150) GN=Bmur_2327 PE=4 SV=
       Observed    Mr(expt)    Mr(calc)    ppm  Start    End Miss Ions  Peptide
      1357.6033  1356.5960  1356.5445   38.0     45 -   54    0   80   R.YDDNFMSFYR.R
      No match to: 1184.7008, 1280.6786, 1560.8831, 2384.0322

16.   A0A0H0XPY9     Mass: 36921     Score: 55     Expect: 0.13   Queries matched: 1
      A0A0H0XPY9_BRAHO UDP-glucose 4-epimerase OS=Brachyspira hyodysenteriae GN=SU45_07725 PE=3 SV=1
       Observed    Mr(expt)    Mr(calc)    ppm  Start    End Miss Ions  Peptide
      1560.8831  1559.8758  1559.8242   33.1    268 -  281    1   43   R.KVTGHPIPAEVCPR.R
      No match to: 1184.7008, 1280.6786, 1357.6033, 2384.0322
```

Search Parameters

```
Type of search         : Sequence Query
Enzyme                 : Trypsin
Fixed modifications    : Carbamidomethyl (C)
Variable modifications : Oxidation (M)
Mass values            : Monoisotopic
Protein Mass           : Unrestricted
Peptide Mass Tolerance : ± 50 ppm
Fragment Mass Tolerance: ± 0.5 Da
Max Missed Cleavages   : 1
Instrument type        : MALDI-TOF-TOF
Query1 (1184.7008,1+)  : Locus:12.1.1.212746.57056
Query2 (1280.6786,1+)  : Locus:12.1.1.212746.57055
Query3 (1357.6033,1+)  : Locus:12.1.1.212746.57054
```

```
Query4 (1560.8831,1+)  : Locus:12.1.1.212746.57057
Query5 (2384.0322,1+)  : Locus:12.1.1.212746.57058
```

**Mascot:** http://www.matrixscience.com/

Mascot Search Results

User	:
Email	:
Search title	: F:\ROD_inmunoproteoma\MALDI\160223_2016_561_562_ROD\ppw_G12_145631468519.txt
MS data file	: F:\ROD_inmunoproteoma\MALDI\160223_2016_561_562_ROD\ppw_G12_145631468519.txt
Database	: Brachyspiraceae Brachyspiraceae_20160127-151122 (40573 sequences; 13470793 residues)
Timestamp	: 24 Feb 2016 at 12:03:10 GMT
Warning	: A Peptide summary report will usually give a much clearer picture of MS/MS search results.
Top Score	: 326 for Q26501, Q26501_BRAHO Flagellin OS=Brachyspira hyodysenteriae GN=flaB1 PE=3 SV=1

Probability Based Mowse Score

Protein score is -10*Log(P), where P is the probability that the observed match is a random event.
Protein scores greater than 59 are significant (p<0.05).
Protein scores are derived from ions scores as a non-probabilistic basis for ranking protein hits.

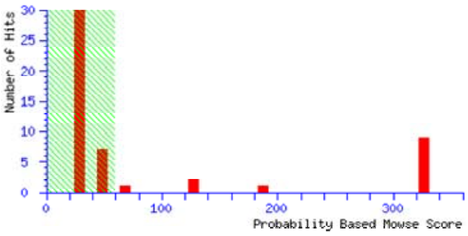

Protein Summary Report

Format As | Protein Summary (deprecated) | Help

Significance threshold p< 0.05 | Max. number of hits AUTO

Standard scoring ◉ MudPIT scoring ○ | Ions score or expect cut-off 0 | Show sub-sets 0

Show pop-ups ◉ Suppress pop-ups ○ | Sort unassigned Decreasing Score | Require bold red ☐

Re-Search All | Search Unmatched

Index

	Accession	Mass	Score	Description
1.	Q26501	31952	326	Q26501_BRAHO Flagellin OS=Brachyspira hyodysenteriae GN=flaB1 PE=3 SV=1
2.	A0A0G4K7S6	31933	326	A0A0G4K7S6_9SPIR Flagellin OS=Brachyspira suanatina GN=flaB2 PE=3 SV=1
3.	G0EKN9	31889	326	G0EKN9_BRAIP Flagellin OS=Brachyspira intermedia (strain ATCC 51140 / PWS/A) GN=Bint_2009 PE=3 SV=1
4.	J9UV65	32001	326	J9UV65_BRAPL Flagellin OS=Brachyspira pilosicoli B2904 GN=flaB2 PE=3 SV=1
5.	D8IAP2	32001	326	D8IAP2_BRAP9 Flagellin OS=Brachyspira pilosicoli (strain ATCC BAA-1826 / 95/1000) GN=flaB2 PE=3 SV=1
6.	K0JFK3	32001	326	K0JFK3_BRAPL Flagellin OS=Brachyspira pilosicoli WesB GN=flaB2-1 PE=3 SV=1
7.	D5U7D6	31944	326	D5U7D6_BRAM5 Flagellin OS=Brachyspira murdochii (strain ATCC 51284 / DSM 12563 / 56-150) GN=Bmur_0623 PE=3 SV=
8.	L8XRQ5	31949	326	L8XRQ5_9SPIR Flagellin OS=Brachyspira hampsonii 30599 GN=H263_12719 PE=3 SV=1
9.	L0X542	31951	326	L0X542_9SPIR Flagellin OS=Brachyspira hampsonii 30446 GN=A966_08614 PE=3 SV=1
10.	C0R027	28784	196	C0R027_BRAHW Flagellin OS=Brachyspira hyodysenteriae (strain ATCC 49526 / WA1) GN=BHWA1_00982 PE=3 SV=1
11.	P80158	2914	137	FLAB1_BRAHO Flagellar filament core protein flaB1 (Fragment) OS=Brachyspira hyodysenteriae GN=flaB1 PE=1 SV=1
12.	R5LFD4	32029	127	R5LFD4_9SPIR Flagellin OS=Brachyspira sp. CAG:700 GN=BN758_02216 PE=3 SV=1
13.	Q7M132	2814	60	Q7M132_BRAHO Flagellar core protein, 34K (Fragment) OS=Brachyspira hyodysenteriae PE=1 SV=1
14.	P80160	31113	50	FLAB2_BRAHO Flagellar filament core protein flaB2 OS=Brachyspira hyodysenteriae GN=flaB2 PE=1 SV=1

Results List

1. Q26501 **Mass:** 31952 **Score:** 326 **Expect:** 1e-28 **Queries matched:** 2
Q26501_BRAHO Flagellin OS=Brachyspira hyodysenteriae GN=flaB1 PE=3 SV=1

Observed	Mr(expt)	Mr(calc)	ppm	Start		End	Miss	Ions	Peptide
1557.8829	1556.8756	1556.8093	42.6	1	-	14	0	115	-.MVINNNISAINAQR.T
1861.9628	1860.9555	1860.8999	29.9	36	-	53	1	182	R.INQAGDDASGLAVSEKMR.T

No match to: 1571.8792, 1774.9723, 1875.9780

2. A0A0G4K7S6 **Mass:** 31933 **Score:** 326 **Expect:** 1e-28 **Queries matched:** 2
A0A0G4K7S6_9SPIR Flagellin OS=Brachyspira suanatina GN=flaB2 PE=3 SV=1

Observed	Mr(expt)	Mr(calc)	ppm	Start		End	Miss	Ions	Peptide
1557.8829	1556.8756	1556.8093	42.6	1	-	14	0	115	-.MVINNNISAINAQR.T
1861.9628	1860.9555	1860.8999	29.9	36	-	53	1	182	R.INQAGDDASGLAVSEKMR.T

No match to: 1571.8792, 1774.9723, 1875.9780

3. G0EKN9 **Mass:** 31889 **Score:** 326 **Expect:** 1e-28 **Queries matched:** 2
G0EKN9_BRAIP Flagellin OS=Brachyspira intermedia (strain ATCC 51140 / PWS/A) GN=Bint_2009 PE=3 SV=1

Observed	Mr(expt)	Mr(calc)	ppm	Start		End	Miss	Ions	Peptide
1557.8829	1556.8756	1556.8093	42.6	1	-	14	0	115	-.MVINNNISAINAQR.T
1861.9628	1860.9555	1860.8999	29.9	36	-	53	1	182	R.INQAGDDASGLAVSEKMR.T

No match to: 1571.8792, 1774.9723, 1875.9780

4. J9UV65 **Mass:** 32001 **Score:** 326 **Expect:** 1e-28 **Queries matched:** 2
J9UV65_BRAPL Flagellin OS=Brachyspira pilosicoli B2904 GN=flaB2 PE=3 SV=1

Observed	Mr(expt)	Mr(calc)	ppm	Start		End	Miss	Ions	Peptide
1557.8829	1556.8756	1556.8093	42.6	1	-	14	0	115	-.MVINNNISAINAQR.T
1861.9628	1860.9555	1860.8999	29.9	36	-	53	1	182	R.INQAGDDASGLAVSEKMR.T

No match to: 1571.8792, 1774.9723, 1875.9780

5. D8IAP2 **Mass:** 32001 **Score:** 326 **Expect:** 1e-28 **Queries matched:** 2
D8IAP2_BRAP9 Flagellin OS=Brachyspira pilosicoli (strain ATCC BAA-1826 / 95/1000) GN=flaB2 PE=3 SV=1

Observed	Mr(expt)	Mr(calc)	ppm	Start		End	Miss	Ions	Peptide
1557.8829	1556.8756	1556.8093	42.6	1	-	14	0	115	-.MVINNNISAINAQR.T
1861.9628	1860.9555	1860.8999	29.9	36	-	53	1	182	R.INQAGDDASGLAVSEKMR.T

No match to: 1571.8792, 1774.9723, 1875.9780

6. K0JFK3 **Mass:** 32001 **Score:** 326 **Expect:** 1e-28 **Queries matched:** 2
K0JFK3_BRAPL Flagellin OS=Brachyspira pilosicoli WesB GN=flaB2-1 PE=3 SV=1

Observed	Mr(expt)	Mr(calc)	ppm	Start		End	Miss	Ions	Peptide
1557.8829	1556.8756	1556.8093	42.6	1	-	14	0	115	-.MVINNNISAINAQR.T
1861.9628	1860.9555	1860.8999	29.9	36	-	53	1	182	R.INQAGDDASGLAVSEKMR.T

No match to: 1571.8792, 1774.9723, 1875.9780

7. D5U7D6 **Mass:** 31944 **Score:** 326 **Expect:** 1e-28 **Queries matched:** 2
D5U7D6_BRAM5 Flagellin OS=Brachyspira murdochii (strain ATCC 51284 / DSM 12563 / 56-150) GN=Bmur_0623 PE=3 SV=1

Observed	Mr(expt)	Mr(calc)	ppm	Start		End	Miss	Ions	Peptide
1557.8829	1556.8756	1556.8093	42.6	1	-	14	0	115	-.MVINNNISAINAQR.T
1861.9628	1860.9555	1860.8999	29.9	36	-	53	1	182	R.INQAGDDASGLAVSEKMR.T

No match to: 1571.8792, 1774.9723, 1875.9780

8. L8XRQ5 **Mass:** 31949 **Score:** 326 **Expect:** 1e-28 **Queries matched:** 2
L8XRQ5_9SPIR Flagellin OS=Brachyspira hampsonii 30599 GN=H263_12719 PE=3 SV=1

Observed	Mr(expt)	Mr(calc)	ppm	Start		End	Miss	Ions	Peptide
1557.8829	1556.8756	1556.8093	42.6	1	-	14	0	115	-.MVINNNISAINAQR.T
1861.9628	1860.9555	1860.8999	29.9	36	-	53	1	182	R.INQAGDDASGLAVSEKMR.T

No match to: 1571.8792, 1774.9723, 1875.9780

9. L0X542 **Mass:** 31951 **Score:** 326 **Expect:** 1e-28 **Queries matched:** 2
L0X542_9SPIR Flagellin OS=Brachyspira hampsonii 30446 GN=A966_08614 PE=3 SV=1

Observed	Mr(expt)	Mr(calc)	ppm	Start		End	Miss	Ions	Peptide
1557.8829	1556.8756	1556.8093	42.6	1	-	14	0	115	-.MVINNNISAINAQR.T
1861.9628	1860.9555	1860.8999	29.9	36	-	53	1	182	R.INQAGDDASGLAVSEKMR.T

No match to: 1571.8792, 1774.9723, 1875.9780

10. C0R027 **Mass:** 28784 **Score:** 196 **Expect:** 1e-15 **Queries matched:** 1
C0R027_BRAHW Flagellin OS=Brachyspira hyodysenteriae (strain ATCC 49526 / WA1) GN=BHWA1_00982 PE=3 SV=1

Observed	Mr(expt)	Mr(calc)	ppm	Start		End	Miss	Ions	Peptide
1861.9628	1860.9555	1860.8999	29.9	8	-	25	1	182	R.INQAGDDASGLAVSEKMR.T

No match to: 1557.8829, 1571.8792, 1774.9723, 1875.9780

11. P80158 **Mass:** 2914 **Score:** 137 **Expect:** 8.1e-10 **Queries matched:** 1
FLAB1_BRAHO Flagellar filament core protein flaB1 (Fragment) OS=Brachyspira hyodysenteriae GN=flaB1 PE=1 SV=1

Observed	Mr(expt)	Mr(calc)	ppm	Start		End	Miss	Ions	Peptide
1557.8829	1556.8756	1556.8093	42.6	1	-	14	0	115	-.MVINNNISAINAQR.T

No match to: 1571.8792, 1774.9723, 1861.9628, 1875.9780

12. R5LFD4 **Mass:** 32029 **Score:** 127 **Expect:** 8.1e-09 **Queries matched:** 1
R5LFD4_9SPIR Flagellin OS=Brachyspira sp. CAG:700 GN=BN758_02216 PE=3 SV=1

Observed	Mr(expt)	Mr(calc)	ppm	Start		End	Miss	Ions	Peptide
1557.8829	1556.8756	1556.8093	42.6	1	-	14	0	115	-.MVINNNISAINAQR.T

No match to: 1571.8792, 1774.9723, 1861.9628, 1875.9780

13. Q7M132 **Mass:** 2814 **Score:** 60 **Expect:** 0.046 **Queries matched:** 1
Q7M132_BRAHO Flagellar core protein, 34K (Fragment) OS=Brachyspira hyodysenteriae PE=1 SV=1

Observed	Mr(expt)	Mr(calc)	ppm	Start		End	Miss	Ions	Peptide
1571.8792	1570.8719	1570.8249	29.9	1	-	14	0	37	-.MIINNNISAINAQR.T

No match to: 1557.8829, 1774.9723, 1861.9628, 1875.9780

14. P80160 **Mass:** 31113 **Score:** 50 **Expect:** 0.44 **Queries matched:** 1
FLAB2_BRAHO Flagellar filament core protein flaB2 OS=Brachyspira hyodysenteriae GN=flaB2 PE=1 SV=1

Observed	Mr(expt)	Mr(calc)	ppm	Start		End	Miss	Ions	Peptide
1571.8792	1570.8719	1570.8249	29.9	1	-	14	0	37	-.MIINNNISAINAQR.T

No match to: 1557.8829, 1774.9723, 1861.9628, 1875.9780

Search Parameters

Type of search : Sequence Query
Enzyme : Trypsin
Fixed modifications : Carbamidomethyl (C)
Variable modifications : Oxidation (M)
Mass values : Monoisotopic
Protein Mass : Unrestricted
Peptide Mass Tolerance : ± 50 ppm
Fragment Mass Tolerance: ± 0.5 Da
Max Missed Cleavages : 1
Instrument type : MALDI-TOF-TOF
Query1 (1557.8829,1+) : Locus:20.1.1.213519.57331
Query2 (1571.8792,1+) : Locus:20.1.1.213519.57333
Query3 (1774.9723,1+) : Locus:20.1.1.213519.57335
Query4 (1861.9628,1+) : Locus:20.1.1.213519.57332
Query5 (1875.9780,1+) : Locus:20.1.1.213519.57334

Mascot: http://www.matrixscience.com/

MATRIX SCIENCE Mascot Search Results

User :
Email :
Search title : F:\ROD_inmunoproteoma\MALDI\ppw_2016_561_562_563_565\ppw_H5_145467558717.txt
MS data file : F:\ROD_inmunoproteoma\MALDI\ppw_2016_561_562_563_565\ppw_H5_145467558717.txt
Database : Brachyspiraceae Brachyspiraceae_20160127-151122 (40573 sequences; 13470793 residues)
Timestamp : 23 Feb 2016 at 16:36:54 GMT
Warning : A Peptide summary report will usually give a much clearer picture of MS/MS search results.
Top Score : 332 for Q26501, Q26501_BRAHO Flagellin OS=Brachyspira hyodysenteriae GN=flaB1 PE=3 SV=1

Probability Based Mowse Score

Protein score is -10*Log(P), where P is the probability that the observed match is a random event.
Protein scores greater than 59 are significant (p<0.05).
Protein scores are derived from ions scores as a non-probabilistic basis for ranking protein hits.

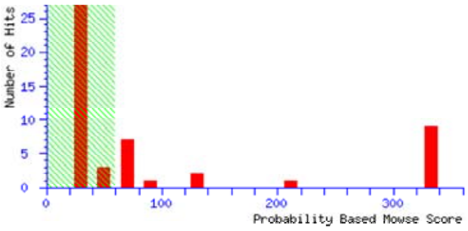

Protein Summary Report

Format As | Protein Summary (deprecated) | Help

Significance threshold p< 0.05 | Max. number of hits AUTO

Standard scoring ◉ MudPIT scoring ○ | Ions score or expect cut-off 0 | Show sub-sets 0

Show pop-ups ◉ Suppress pop-ups ○ | Sort unassigned Decreasing Score | Require bold red ☐

Re-Search All | Search Unmatched

Index

	Accession	Mass	Score	Description
1.	Q26501	31952	332	Q26501_BRAHO Flagellin OS=Brachyspira hyodysenteriae GN=flaB1 PE=3 SV=1
2.	A0A0G4K7S6	31933	332	A0A0G4K7S6_9SPIR Flagellin OS=Brachyspira suanatina GN=flaB2 PE=3 SV=1
3.	G0EKN9	31889	332	G0EKN9_BRAIP Flagellin OS=Brachyspira intermedia (strain ATCC 51140 / PWS/A) GN=Bint_2009 PE=3 SV=1
4.	J9UV65	32001	332	J9UV65_BRAPL Flagellin OS=Brachyspira pilosicoli B2904 GN=flaB2 PE=3 SV=1
5.	D8IAP2	32001	332	D8IAP2_BRAP9 Flagellin OS=Brachyspira pilosicoli (strain ATCC BAA-1826 / 95/1000) GN=flaB2 PE=3 SV=1
6.	K0JFK3	32001	332	K0JFK3_BRAPL Flagellin OS=Brachyspira pilosicoli WesB GN=flaB2-1 PE=3 SV=1
7.	D5U7D6	31944	332	D5U7D6_BRAM5 Flagellin OS=Brachyspira murdochii (strain ATCC 51284 / DSM 12563 / 56-150) GN=Bmur_0623 PE=3 SV=
8.	L8XRQ5	31949	332	L8XRQ5_9SPIR Flagellin OS=Brachyspira hampsonii 30599 GN=H263_12719 PE=3 SV=1
9.	L0X542	31951	332	L0X542_9SPIR Flagellin OS=Brachyspira hampsonii 30446 GN=A966_08614 PE=3 SV=1
10.	C0R027	28784	202	C0R027_BRAHW Flagellin OS=Brachyspira hyodysenteriae (strain ATCC 49526 / WA1) GN=BHWA1_00982 PE=3 SV=1
11.	P80158	2914	137	FLAB1_BRAHO Flagellar filament core protein flaB1 (Fragment) OS=Brachyspira hyodysenteriae GN=flaB1 PE=1 SV=1
12.	R5LFD4	32029	127	R5LFD4_9SPIR Flagellin OS=Brachyspira sp. CAG:700 GN=BN758_02216 PE=3 SV=1
13.	Q7M132	2814	85	Q7M132_BRAHO Flagellar core protein, 34K (Fragment) OS=Brachyspira hyodysenteriae PE=1 SV=1
14.	P80160	31113	75	FLAB2_BRAHO Flagellar filament core protein flaB2 OS=Brachyspira hyodysenteriae GN=flaB2 PE=1 SV=1
15.	A0A0H0X544	31129	75	A0A0H0X544_BRAHO Flagellin OS=Brachyspira hyodysenteriae GN=SU43_01870 PE=3 SV=1
16.	A0A0G4K9G4	31184	75	A0A0G4K9G4_9SPIR Flagellin OS=Brachyspira suanatina GN=flaB2 PE=3 SV=1
17.	A0A0H0ULZ7	31057	75	A0A0H0ULZ7_BRAHO Flagellin OS=Brachyspira hyodysenteriae GN=SR30_04500 PE=3 SV=1
18.	D5UB01	31218	75	D5UB01_BRAM5 Flagellin OS=Brachyspira murdochii (strain ATCC 51284 / DSM 12563 / 56-150) GN=Bmur_1792 PE=3 SV=
19.	L0X2I6	31181	75	L0X2I6_9SPIR Flagellin OS=Brachyspira hampsonii 30446 GN=A966_05778 PE=3 SV=1
20.	L8XTT2	31194	75	L8XTT2_9SPIR Flagellin OS=Brachyspira hampsonii 30599 GN=H263_08389 PE=3 SV=1
21.	R5H2T9	50225	43	R5H2T9_9SPIR Glutamate synthase (NADPH) homotetrameric OS=Brachyspira sp. CAG:484 GN=BN676_00441 PE=4 SV=1

Results List

1. Q26501 **Mass:** 31952 **Score:** 332 **Expect:** 2.6e-29 **Queries matched:** 2
Q26501_BRAHO Flagellin OS=Brachyspira hyodysenteriae GN=flaB1 PE=3 SV=1

Observed	Mr(expt)	Mr(calc)	ppm	Start	End	Miss	Ions	Peptide
1557.8590	1556.8517	1556.8093	27.3	1 -	14	0	115	-.MVINNNISAINAQR.T
1861.9403	1860.9330	1860.8999	17.8	36 -	53	1	188	R.INQAGDDASGLAVSEKMR.T

No match to: 1571.8634, 1774.9518, 1875.9542

2. A0A0G4K7S6 **Mass:** 31933 **Score:** 332 **Expect:** 2.6e-29 **Queries matched:** 2
A0A0G4K7S6_9SPIR Flagellin OS=Brachyspira suanatina GN=flaB2 PE=3 SV=1

Observed	Mr(expt)	Mr(calc)	ppm	Start	End	Miss	Ions	Peptide
1557.8590	1556.8517	1556.8093	27.3	1 -	14	0	115	-.MVINNNISAINAQR.T
1861.9403	1860.9330	1860.8999	17.8	36 -	53	1	188	R.INQAGDDASGLAVSEKMR.T

No match to: 1571.8634, 1774.9518, 1875.9542

3. G0EKN9 **Mass:** 31889 **Score:** 332 **Expect:** 2.6e-29 **Queries matched:** 2
G0EKN9_BRAIP Flagellin OS=Brachyspira intermedia (strain ATCC 51140 / PWS/A) GN=Bint_2009 PE=3 SV=1

Observed	Mr(expt)	Mr(calc)	ppm	Start	End	Miss	Ions	Peptide
1557.8590	1556.8517	1556.8093	27.3	1 -	14	0	115	-.MVINNNISAINAQR.T
1861.9403	1860.9330	1860.8999	17.8	36 -	53	1	188	R.INQAGDDASGLAVSEKMR.T

No match to: 1571.8634, 1774.9518, 1875.9542

4. J9UV65 **Mass:** 32001 **Score:** 332 **Expect:** 2.6e-29 **Queries matched:** 2
J9UV65_BRAPL Flagellin OS=Brachyspira pilosicoli B2904 GN=flaB2 PE=3 SV=1

Observed	Mr(expt)	Mr(calc)	ppm	Start		End	Miss	Ions	Peptide
1557.8590	1556.8517	1556.8093	27.3	1	-	14	0	115	-.MVINNNISAINAQR.T
1861.9403	1860.9330	1860.8999	17.8	36	-	53	1	188	R.INQAGDDASGLAVSEKMR.T

No match to: 1571.8634, 1774.9518, 1875.9542

5. D8IAP2 **Mass:** 32001 **Score:** 332 **Expect:** 2.6e-29 **Queries matched:** 2
D8IAP2_BRAP9 Flagellin OS=Brachyspira pilosicoli (strain ATCC BAA-1826 / 95/1000) GN=flaB2 PE=3 SV=1

Observed	Mr(expt)	Mr(calc)	ppm	Start		End	Miss	Ions	Peptide
1557.8590	1556.8517	1556.8093	27.3	1	-	14	0	115	-.MVINNNISAINAQR.T
1861.9403	1860.9330	1860.8999	17.8	36	-	53	1	188	R.INQAGDDASGLAVSEKMR.T

No match to: 1571.8634, 1774.9518, 1875.9542

6. K0JFK3 **Mass:** 32001 **Score:** 332 **Expect:** 2.6e-29 **Queries matched:** 2
K0JFK3_BRAPL Flagellin OS=Brachyspira pilosicoli WesB GN=flaB2-1 PE=3 SV=1

Observed	Mr(expt)	Mr(calc)	ppm	Start		End	Miss	Ions	Peptide
1557.8590	1556.8517	1556.8093	27.3	1	-	14	0	115	-.MVINNNISAINAQR.T
1861.9403	1860.9330	1860.8999	17.8	36	-	53	1	188	R.INQAGDDASGLAVSEKMR.T

No match to: 1571.8634, 1774.9518, 1875.9542

7. D5U7D6 **Mass:** 31944 **Score:** 332 **Expect:** 2.6e-29 **Queries matched:** 2
D5U7D6_BRAM5 Flagellin OS=Brachyspira murdochii (strain ATCC 51284 / DSM 12563 / 56-150) GN=Bmur_0623 PE=3 SV=1

Observed	Mr(expt)	Mr(calc)	ppm	Start		End	Miss	Ions	Peptide
1557.8590	1556.8517	1556.8093	27.3	1	-	14	0	115	-.MVINNNISAINAQR.T
1861.9403	1860.9330	1860.8999	17.8	36	-	53	1	188	R.INQAGDDASGLAVSEKMR.T

No match to: 1571.8634, 1774.9518, 1875.9542

8. L8XRQ5 **Mass:** 31949 **Score:** 332 **Expect:** 2.6e-29 **Queries matched:** 2
L8XRQ5_9SPIR Flagellin OS=Brachyspira hampsonii 30599 GN=H263_12719 PE=3 SV=1

Observed	Mr(expt)	Mr(calc)	ppm	Start		End	Miss	Ions	Peptide
1557.8590	1556.8517	1556.8093	27.3	1	-	14	0	115	-.MVINNNISAINAQR.T
1861.9403	1860.9330	1860.8999	17.8	36	-	53	1	188	R.INQAGDDASGLAVSEKMR.T

No match to: 1571.8634, 1774.9518, 1875.9542

9. L0X542 **Mass:** 31951 **Score:** 332 **Expect:** 2.6e-29 **Queries matched:** 2
L0X542_9SPIR Flagellin OS=Brachyspira hampsonii 30446 GN=A966_08614 PE=3 SV=1

Observed	Mr(expt)	Mr(calc)	ppm	Start		End	Miss	Ions	Peptide
1557.8590	1556.8517	1556.8093	27.3	1	-	14	0	115	-.MVINNNISAINAQR.T
1861.9403	1860.9330	1860.8999	17.8	36	-	53	1	188	R.INQAGDDASGLAVSEKMR.T

No match to: 1571.8634, 1774.9518, 1875.9542

10. C0R027 **Mass:** 28784 **Score:** 202 **Expect:** 2.6e-16 **Queries matched:** 1
C0R027_BRAHW Flagellin OS=Brachyspira hyodysenteriae (strain ATCC 49526 / WA1) GN=BHWA1_00982 PE=3 SV=1

Observed	Mr(expt)	Mr(calc)	ppm	Start		End	Miss	Ions	Peptide
1861.9403	1860.9330	1860.8999	17.8	8	-	25	1	188	R.INQAGDDASGLAVSEKMR.T

No match to: 1557.8590, 1571.8634, 1774.9518, 1875.9542

11. P80158 **Mass:** 2914 **Score:** 137 **Expect:** 8.1e-10 **Queries matched:** 1
FLAB1_BRAHO Flagellar filament core protein flaB1 (Fragment) OS=Brachyspira hyodysenteriae GN=flaB1 PE=1 SV=1

Observed	Mr(expt)	Mr(calc)	ppm	Start		End	Miss	Ions	Peptide
1557.8590	1556.8517	1556.8093	27.3	1	-	14	0	115	-.MVINNNISAINAQR.T

No match to: 1571.8634, 1774.9518, 1861.9403, 1875.9542

12. R5LFD4 **Mass:** 32029 **Score:** 127 **Expect:** 8.1e-09 **Queries matched:** 1
R5LFD4_9SPIR Flagellin OS=Brachyspira sp. CAG:700 GN=BN758_02216 PE=3 SV=1

Observed	Mr(expt)	Mr(calc)	ppm	Start		End	Miss	Ions	Peptide
1557.8590	1556.8517	1556.8093	27.3	1	-	14	0	115	-.MVINNNISAINAQR.T

No match to: 1571.8634, 1774.9518, 1861.9403, 1875.9542

13. Q7M132 **Mass:** 2814 **Score:** 85 **Expect:** 0.00013 **Queries matched:** 1
Q7M132_BRAHO Flagellar core protein, 34K (Fragment) OS=Brachyspira hyodysenteriae PE=1 SV=1

Observed	Mr(expt)	Mr(calc)	ppm	Start		End	Miss	Ions	Peptide
1571.8634	1570.8561	1570.8249	19.9	1	-	14	0	63	-.MIINNNISAINAQR.T

No match to: 1557.8590, 1774.9518, 1861.9403, 1875.9542

14. P80160 **Mass:** 31113 **Score:** 75 **Expect:** 0.0013 **Queries matched:** 1
FLAB2_BRAHO Flagellar filament core protein flaB2 OS=Brachyspira hyodysenteriae GN=flaB2 PE=1 SV=1

Observed	Mr(expt)	Mr(calc)	ppm	Start		End	Miss	Ions	Peptide
1571.8634	1570.8561	1570.8249	19.9	1	-	14	0	63	-.MIINNNISAINAQR.T

No match to: 1557.8590, 1774.9518, 1861.9403, 1875.9542

15. A0A0H0X544 **Mass:** 31129 **Score:** 75 **Expect:** 0.0013 **Queries matched:** 1
A0A0H0X544_BRAHO Flagellin OS=Brachyspira hyodysenteriae GN=SU43_01870 PE=3 SV=1

Observed	Mr(expt)	Mr(calc)	ppm	Start		End	Miss	Ions	Peptide
1571.8634	1570.8561	1570.8249	19.9	1	-	14	0	63	-.MIINNNISAINAQR.T

No match to: 1557.8590, 1774.9518, 1861.9403, 1875.9542

16. A0A0G4K9G4 **Mass:** 31184 **Score:** 75 **Expect:** 0.0013 **Queries matched:** 1
A0A0G4K9G4_9SPIR Flagellin OS=Brachyspira suanatina GN=flaB2 PE=3 SV=1

Observed	Mr(expt)	Mr(calc)	ppm	Start		End	Miss	Ions	Peptide
1571.8634	1570.8561	1570.8249	19.9	1	-	14	0	63	-.MIINNNISAINAQR.T

No match to: 1557.8590, 1774.9518, 1861.9403, 1875.9542

17. A0A0H0ULZ7 **Mass:** 31057 **Score:** 75 **Expect:** 0.0013 **Queries matched:** 1
A0A0H0ULZ7_BRAHO Flagellin OS=Brachyspira hyodysenteriae GN=SR30_04500 PE=3 SV=1

```
 Observed   Mr(expt)   Mr(calc)   ppm  Start   End Miss Ions  Peptide
1571.8634  1570.8561  1570.8249   19.9     1 -  14   0   63   -.MIINNNISAINAQR.T
No match to: 1557.8590, 1774.9518, 1861.9403, 1875.9542

18.  D5UB01    Mass: 31218    Score: 75     Expect: 0.0013  Queries matched: 1
     D5UB01_BRAM5 Flagellin OS=Brachyspira murdochii (strain ATCC 51284 / DSM 12563 / 56-150) GN=Bmur_1792 PE=3 SV=1
      Observed   Mr(expt)   Mr(calc)   ppm  Start   End Miss Ions  Peptide
     1571.8634  1570.8561  1570.8249   19.9     1 -  14   0   63   -.MIINNNISAINAQR.T
     No match to: 1557.8590, 1774.9518, 1861.9403, 1875.9542

19.  L0X2I6    Mass: 31181    Score: 75     Expect: 0.0013  Queries matched: 1
     L0X2I6_9SPIR Flagellin OS=Brachyspira hampsonii 30446 GN=A966_05778 PE=3 SV=1
      Observed   Mr(expt)   Mr(calc)   ppm  Start   End Miss Ions  Peptide
     1571.8634  1570.8561  1570.8249   19.9     1 -  14   0   63   -.MIINNNISAINAQR.T
     No match to: 1557.8590, 1774.9518, 1861.9403, 1875.9542

20.  L8XTT2    Mass: 31194    Score: 75     Expect: 0.0013  Queries matched: 1
     L8XTT2_9SPIR Flagellin OS=Brachyspira hampsonii 30599 GN=H263_08389 PE=3 SV=1
      Observed   Mr(expt)   Mr(calc)   ppm  Start   End Miss Ions  Peptide
     1571.8634  1570.8561  1570.8249   19.9     1 -  14   0   63   -.MIINNNISAINAQR.T
     No match to: 1557.8590, 1774.9518, 1861.9403, 1875.9542

21.  R5H2T9    Mass: 50225    Score: 43     Expect: 2  Queries matched: 3
     R5H2T9_9SPIR Glutamate synthase (NADPH) homotetrameric OS=Brachyspira sp. CAG:484 GN=BN676_00441 PE=4 SV=1
      Observed   Mr(expt)   Mr(calc)   ppm  Start   End Miss Ions  Peptide
     1557.8590  1556.8517  1556.8497   1.28   195 - 208   1   ---   K.QMGVKFHTNVIVGK.S
     1861.9403  1860.9330  1860.8411   49.4   345 - 361   0   ---   K.AVDLEVMELGEPDESGR.R + Oxidation (M)
     1875.9542  1874.9469  1874.9342   6.77    72 -  88   1   ---   K.AGEIIRETSMLPSVCGR.V
     No match to: 1571.8634, 1774.9518
```

Search Parameters

```
Type of search         : Sequence Query
Enzyme                 : Trypsin
Fixed modifications    : Carbamidomethyl (C)
Variable modifications : Oxidation (M)
Mass values            : Monoisotopic
Protein Mass           : Unrestricted
Peptide Mass Tolerance : ± 50 ppm
Fragment Mass Tolerance: ± 0.5 Da
Max Missed Cleavages   : 1
Instrument type        : MALDI-TOF-TOF
Query1 (1557.8590,1+)  : Locus:5.1.1.212739.57019
Query2 (1571.8634,1+)  : Locus:5.1.1.212739.57021
Query3 (1774.9518,1+)  : Locus:5.1.1.212739.57022
Query4 (1861.9403,1+)  : Locus:5.1.1.212739.57020
Query5 (1875.9542,1+)  : Locus:5.1.1.212739.57023
```

Mascot: http://www.matrixscience.com/

MATRIX SCIENCE Mascot Search Results

```
User          :
Email         :
Search title  : F:\ROD_inmunoproteoma\MALDI\ppw_2016_562_563_ROD\ppw_I10_145397050026.txt
MS data file  : F:\ROD_inmunoproteoma\MALDI\ppw_2016_562_563_ROD\ppw_I10_145397050026.txt
Database      : Brachyspiraceae Brachyspiraceae_20160127-151122 (40573 sequences; 13470793 residues)
Timestamp     : 23 Feb 2016 at 16:06:09 GMT
Warning       : A Peptide summary report will usually give a much clearer picture of MS/MS search results.
Top Score     : 230 for Q26501, Q26501_BRAHO Flagellin OS=Brachyspira hyodysenteriae GN=flaB1 PE=3 SV=1
```

Probability Based Mowse Score

Protein score is -10*Log(P), where P is the probability that the observed match is a random event.
Protein scores greater than 59 are significant (p<0.05).
Protein scores are derived from ions scores as a non-probabilistic basis for ranking protein hits.

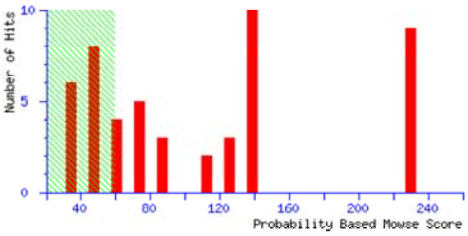

Protein Summary Report

Format As | Protein Summary (deprecated) | Help

Significance threshold p< 0.05 | Max. number of hits AUTO

Standard scoring ◉ MudPIT scoring ○ | Ions score or expect cut-off 0 | Show sub-sets 0

Show pop-ups ◉ Suppress pop-ups ○ | Sort unassigned Decreasing Score | Require bold red ☐

Re-Search All | Search Unmatched

Index

	Accession	Mass	Score	Description
1.	Q26501	31952	230	Q26501_BRAHO Flagellin OS=Brachyspira hyodysenteriae GN=flaB1 PE=3 SV=1
2.	A0A0G4K7S6	31933	230	A0A0G4K7S6_9SPIR Flagellin OS=Brachyspira suanatina GN=flaB2 PE=3 SV=1
3.	G0EKN9	31889	230	G0EKN9_BRAIP Flagellin OS=Brachyspira intermedia (strain ATCC 51140 / PWS/A) GN=Bint_2009 PE=3 SV=1
4.	J9UV65	32001	230	J9UV65_BRAPL Flagellin OS=Brachyspira pilosicoli B2904 GN=flaB2 PE=3 SV=1
5.	D8IAP2	32001	230	D8IAP2_BRAP9 Flagellin OS=Brachyspira pilosicoli (strain ATCC BAA-1826 / 95/1000) GN=flaB2 PE=3 SV=1
6.	K0JFK3	32001	230	K0JFK3_BRAPL Flagellin OS=Brachyspira pilosicoli WesB GN=flaB2-1 PE=3 SV=1
7.	D5U7D6	31944	230	D5U7D6_BRAM5 Flagellin OS=Brachyspira murdochii (strain ATCC 51284 / DSM 12563 / 56-150) GN=Bmur_0623 PE=3 SV=
8.	L8XRQ5	31949	230	L8XRQ5_9SPIR Flagellin OS=Brachyspira hampsonii 30599 GN=H263_12719 PE=3 SV=1
9.	L0X542	31951	230	L0X542_9SPIR Flagellin OS=Brachyspira hampsonii 30446 GN=A966_08614 PE=3 SV=1
10.	B7T1P3	24499	140	B7T1P3_9SPIR FlaA (Fragment) OS=Brachyspira innocens PE=4 SV=1
11.	B7T1Q0	24499	140	B7T1Q0_9SPIR FlaA (Fragment) OS=Brachyspira alvinipulli PE=4 SV=1
12.	B7T1P9	24499	140	B7T1P9_9SPIR FlaA (Fragment) OS=Brachyspira intermedia PE=4 SV=1
13.	P32520	35964	136	FLAA1_BRAHO Flagellar filament outer layer protein flaA1 OS=Brachyspira hyodysenteriae GN=flaA1 PE=1 SV=1
14.	A0A0H0TRE1	35964	136	A0A0H0TRE1_BRAHO Flagellar filament protein FlaA OS=Brachyspira hyodysenteriae GN=SR30_03350 PE=4 SV=1
15.	A0A0G4K8P6	35920	136	A0A0G4K8P6_9SPIR Flagellar filament outer layer protein flaA1 OS=Brachyspira suanatina GN=flaA1 PE=4 SV=1
16.	C0R0T5	35964	136	C0R0T5_BRAHW Flagellar filament outer layer protein FlaA OS=Brachyspira hyodysenteriae (strain ATCC 49526 / WA
17.	A0A0H0V489	35934	136	A0A0H0V489_BRAHO Flagellar filament protein FlaA OS=Brachyspira hyodysenteriae GN=SZ48_11445 PE=4 SV=1
18.	A0A0H0WR23	35965	136	A0A0H0WR23_BRAHO Flagellar filament protein FlaA OS=Brachyspira hyodysenteriae GN=SZ42_05145 PE=4 SV=1
19.	G0EJW8	35935	136	G0EJW8_BRAIP Flagellar filament outer layer protein FlaA OS=Brachyspira intermedia (strain ATCC 51140 / PWS/A)
20.	P80158	2914	130	FLAB1_BRAHO Flagellar filament core protein flaB1 (Fragment) OS=Brachyspira hyodysenteriae GN=flaB1 PE=1 SV=1
21.	B7T1P4	24426	122	B7T1P4_9SPIR FlaA (Fragment) OS=Brachyspira innocens PE=4 SV=1
22.	R5LFD4	32029	120	R5LFD4_9SPIR Flagellin OS=Brachyspira sp. CAG:700 GN=BN758_02216 PE=3 SV=1
23.	D5U8F3	35700	118	D5U8F3_BRAM5 Flagellar filament outer layer protein FlaA OS=Brachyspira murdochii (strain ATCC 51284 / DSM 125
24.	C0R027	28784	107	C0R027_BRAHW Flagellin OS=Brachyspira hyodysenteriae (strain ATCC 49526 / WA1) GN=BHWA1_00982 PE=3 SV=1
25.	R5LCF6	35187	82	R5LCF6_9SPIR Flagellar filament outer layer protein FlaA OS=Brachyspira sp. CAG:700 GN=BN758_01892 PE=4 SV=1
26.	B7T1P1	24517	81	B7T1P1_9SPIR FlaA (Fragment) OS=Brachyspira sp. SRBP205IR PE=4 SV=1
27.	B7T1P2	24517	81	B7T1P2_9SPIR FlaA (Fragment) OS=Brachyspira sp. SRBP5IR PE=4 SV=1
28.	J9UWR4	35678	79	J9UWR4_BRAPL Flagellar filament outer layer protein FlaA OS=Brachyspira pilosicoli B2904 GN=B2904_orf2029 PE=4
29.	D8IBY6	35707	79	D8IBY6_BRAP9 Flagellar filament outer layer protein, FlaA OS=Brachyspira pilosicoli (strain ATCC BAA-1826 / 95
30.	K0JH04	35709	79	K0JH04_BRAPL Flagellar filament outer layer protein FlaA OS=Brachyspira pilosicoli WesB GN=flaA PE=4 SV=1
31.	L8XQZ8	35741	79	L8XQZ8_9SPIR Flagellar filament outer layer protein FlaA OS=Brachyspira hampsonii 30599 GN=H263_11839 PE=4 SV=
32.	Q5XPB3	36034	79	Q5XPB3_BRAPL FlaA1 (Fragment) OS=Brachyspira pilosicoli PE=4 SV=1
33.	B7T1P5	24371	63	B7T1P5_9SPIR FlaA (Fragment) OS=Brachyspira innocens PE=4 SV=1
34.	L8XVU3	52619	62	L8XVU3_9SPIR Uncharacterized protein OS=Brachyspira hampsonii 30599 GN=H263_01570 PE=4 SV=1
35.	L0X013	52663	62	L0X013_9SPIR Uncharacterized protein OS=Brachyspira hampsonii 30446 GN=A966_10572 PE=4 SV=1
36.	B7T1P7	24514	55	B7T1P7_9SPIR FlaA (Fragment) OS=Brachyspira canis PE=4 SV=1

Results List

1. Q26501 **Mass:** 31952 **Score:** 230 **Expect:** 4.1e-19 **Queries matched:** 2

Q26501_BRAHO Flagellin OS=Brachyspira hyodysenteriae GN=flaB1 PE=3 SV=1

Observed	Mr(expt)	Mr(calc)	ppm	Start	End	Miss	Ions	Peptide
1557.7646	1556.7573	1556.8093	-33.36	1 -	14	0	107	-.MVINNNISAINAQR.T
1861.8424	1860.8351	1860.8999	-34.83	36 -	53	1	94	R.INQAGDDASGLAVSEKMR.T

No match to: 1241.6952, 1625.8173, 1765.8285

2. A0A0G4K7S6 **Mass:** 31933 **Score:** 230 **Expect:** 4.1e-19 **Queries matched:** 2
A0A0G4K7S6_9SPIR Flagellin OS=Brachyspira suanatina GN=flaB2 PE=3 SV=1

Observed	Mr(expt)	Mr(calc)	ppm	Start	End	Miss	Ions	Peptide
1557.7646	1556.7573	1556.8093	-33.36	1 -	14	0	107	-.MVINNNISAINAQR.T
1861.8424	1860.8351	1860.8999	-34.83	36 -	53	1	94	R.INQAGDDASGLAVSEKMR.T

No match to: 1241.6952, 1625.8173, 1765.8285

3. G0EKN9 **Mass:** 31889 **Score:** 230 **Expect:** 4.1e-19 **Queries matched:** 2
G0EKN9_BRAIP Flagellin OS=Brachyspira intermedia (strain ATCC 51140 / PWS/A) GN=Bint_2009 PE=3 SV=1

Observed	Mr(expt)	Mr(calc)	ppm	Start	End	Miss	Ions	Peptide
1557.7646	1556.7573	1556.8093	-33.36	1 -	14	0	107	-.MVINNNISAINAQR.T
1861.8424	1860.8351	1860.8999	-34.83	36 -	53	1	94	R.INQAGDDASGLAVSEKMR.T

No match to: 1241.6952, 1625.8173, 1765.8285

4. J9UV65 **Mass:** 32001 **Score:** 230 **Expect:** 4.1e-19 **Queries matched:** 2
J9UV65_BRAPL Flagellin OS=Brachyspira pilosicoli B2904 GN=flaB2 PE=3 SV=1

Observed	Mr(expt)	Mr(calc)	ppm	Start	End	Miss	Ions	Peptide
1557.7646	1556.7573	1556.8093	-33.36	1 -	14	0	107	-.MVINNNISAINAQR.T
1861.8424	1860.8351	1860.8999	-34.83	36 -	53	1	94	R.INQAGDDASGLAVSEKMR.T

No match to: 1241.6952, 1625.8173, 1765.8285

5. D8IAP2 **Mass:** 32001 **Score:** 230 **Expect:** 4.1e-19 **Queries matched:** 2
D8IAP2_BRAP9 Flagellin OS=Brachyspira pilosicoli (strain ATCC BAA-1826 / 95/1000) GN=flaB2 PE=3 SV=1

Observed	Mr(expt)	Mr(calc)	ppm	Start	End	Miss	Ions	Peptide
1557.7646	1556.7573	1556.8093	-33.36	1 -	14	0	107	-.MVINNNISAINAQR.T
1861.8424	1860.8351	1860.8999	-34.83	36 -	53	1	94	R.INQAGDDASGLAVSEKMR.T

No match to: 1241.6952, 1625.8173, 1765.8285

6. K0JFK3 **Mass:** 32001 **Score:** 230 **Expect:** 4.1e-19 **Queries matched:** 2
K0JFK3_BRAPL Flagellin OS=Brachyspira pilosicoli WesB GN=flaB2-1 PE=3 SV=1

Observed	Mr(expt)	Mr(calc)	ppm	Start	End	Miss	Ions	Peptide
1557.7646	1556.7573	1556.8093	-33.36	1 -	14	0	107	-.MVINNNISAINAQR.T
1861.8424	1860.8351	1860.8999	-34.83	36 -	53	1	94	R.INQAGDDASGLAVSEKMR.T

No match to: 1241.6952, 1625.8173, 1765.8285

7. D5U7D6 **Mass:** 31944 **Score:** 230 **Expect:** 4.1e-19 **Queries matched:** 2
D5U7D6_BRAM5 Flagellin OS=Brachyspira murdochii (strain ATCC 51284 / DSM 12563 / 56-150) GN=Bmur_0623 PE=3 SV=1

Observed	Mr(expt)	Mr(calc)	ppm	Start	End	Miss	Ions	Peptide
1557.7646	1556.7573	1556.8093	-33.36	1 -	14	0	107	-.MVINNNISAINAQR.T
1861.8424	1860.8351	1860.8999	-34.83	36 -	53	1	94	R.INQAGDDASGLAVSEKMR.T

No match to: 1241.6952, 1625.8173, 1765.8285

8. L8XRQ5 **Mass:** 31949 **Score:** 230 **Expect:** 4.1e-19 **Queries matched:** 2
L8XRQ5_9SPIR Flagellin OS=Brachyspira hampsonii 30599 GN=H263_12719 PE=3 SV=1

Observed	Mr(expt)	Mr(calc)	ppm	Start	End	Miss	Ions	Peptide
1557.7646	1556.7573	1556.8093	-33.36	1 -	14	0	107	-.MVINNNISAINAQR.T
1861.8424	1860.8351	1860.8999	-34.83	36 -	53	1	94	R.INQAGDDASGLAVSEKMR.T

No match to: 1241.6952, 1625.8173, 1765.8285

9. L0X542 **Mass:** 31951 **Score:** 230 **Expect:** 4.1e-19 **Queries matched:** 2
L0X542_9SPIR Flagellin OS=Brachyspira hampsonii 30446 GN=A966_08614 PE=3 SV=1

Observed	Mr(expt)	Mr(calc)	ppm	Start	End	Miss	Ions	Peptide
1557.7646	1556.7573	1556.8093	-33.36	1 -	14	0	107	-.MVINNNISAINAQR.T
1861.8424	1860.8351	1860.8999	-34.83	36 -	53	1	94	R.INQAGDDASGLAVSEKMR.T

No match to: 1241.6952, 1625.8173, 1765.8285

10. B7T1P3 **Mass:** 24499 **Score:** 140 **Expect:** 4.1e-10 **Queries matched:** 2
B7T1P3_9SPIR FlaA (Fragment) OS=Brachyspira innocens PE=4 SV=1

Observed	Mr(expt)	Mr(calc)	ppm	Start	End	Miss	Ions	Peptide
1241.6952	1240.6879	1240.7292	-33.24	170 -	179	1	42	R.VLVREPLYPR.M
1625.8173	1624.8100	1624.8647	-33.64	180 -	193	1	67	R.MIPSVKLDSLGFYR.T

No match to: 1557.7646, 1765.8285, 1861.8424

11. B7T1Q0 **Mass:** 24499 **Score:** 140 **Expect:** 4.1e-10 **Queries matched:** 2
B7T1Q0_9SPIR FlaA (Fragment) OS=Brachyspira alvinipulli PE=4 SV=1

Observed	Mr(expt)	Mr(calc)	ppm	Start	End	Miss	Ions	Peptide
1241.6952	1240.6879	1240.7292	-33.24	170 -	179	1	42	R.VLVREPLYPR.M
1625.8173	1624.8100	1624.8647	-33.64	180 -	193	1	67	R.MIPSVKLDSLGFYR.T

No match to: 1557.7646, 1765.8285, 1861.8424

12. B7T1P9 **Mass:** 24499 **Score:** 140 **Expect:** 4.1e-10 **Queries matched:** 2
B7T1P9_9SPIR FlaA (Fragment) OS=Brachyspira intermedia PE=4 SV=1

Observed	Mr(expt)	Mr(calc)	ppm	Start	End	Miss	Ions	Peptide
1241.6952	1240.6879	1240.7292	-33.24	170 -	179	1	42	R.VLVREPLYPR.M
1625.8173	1624.8100	1624.8647	-33.64	180 -	193	1	67	R.MIPSVKLDSLGFYR.T

No match to: 1557.7646, 1765.8285, 1861.8424

13. P32520 **Mass:** 35964 **Score:** 136 **Expect:** 1e-09 **Queries matched:** 2
FLAA1_BRAHO Flagellar filament outer layer protein flaA1 OS=Brachyspira hyodysenteriae GN=flaA1 PE=1 SV=1

Observed	Mr(expt)	Mr(calc)	ppm	Start	End	Miss	Ions	Peptide
1241.6952	1240.6879	1240.7292	-33.24	201 -	210	1	42	R.VLVREPLYPR.M
1625.8173	1624.8100	1624.8647	-33.64	211 -	224	1	67	R.MIPSVKLDSLGFYR.T

No match to: 1557.7646, 1765.8285, 1861.8424

14. A0A0H0TRE1 **Mass:** 35964 **Score:** 136 **Expect:** 1e-09 **Queries matched:** 2
A0A0H0TRE1_BRAHO Flagellar filament protein FlaA OS=Brachyspira hyodysenteriae GN=SR30_03350 PE=4 SV=1

Observed	Mr(expt)	Mr(calc)	ppm	Start	End	Miss	Ions	Peptide

1241.6952	1240.6879	1240.7292	-33.24	201	210	1	42	R.VLVREPLYPR.M
1625.8173	1624.8100	1624.8647	-33.64	211	224	1	67	R.MIPSVKLDSLGFYR.T

No match to: 1557.7646, 1765.8285, 1861.8424

15. A0A0G4K8P6 **Mass:** 35920 **Score:** 136 **Expect:** 1e-09 **Queries matched:** 2
A0A0G4K8P6_9SPIR Flagellar filament outer layer protein flaA1 OS=Brachyspira suanatina GN=flaA1 PE=4 SV=1

Observed	Mr(expt)	Mr(calc)	ppm	Start	End	Miss	Ions	Peptide
1241.6952	1240.6879	1240.7292	-33.24	201	210	1	42	R.VLVREPLYPR.M
1625.8173	1624.8100	1624.8647	-33.64	211	224	1	67	R.MIPSVKLDSLGFYR.T

No match to: 1557.7646, 1765.8285, 1861.8424

16. C0R0T5 **Mass:** 35964 **Score:** 136 **Expect:** 1e-09 **Queries matched:** 2
C0R0T5_BRAHW Flagellar filament outer layer protein FlaA OS=Brachyspira hyodysenteriae (strain ATCC 49526 / WA1) GN=flaA PE=4 SV=1

Observed	Mr(expt)	Mr(calc)	ppm	Start	End	Miss	Ions	Peptide
1241.6952	1240.6879	1240.7292	-33.24	201	210	1	42	R.VLVREPLYPR.M
1625.8173	1624.8100	1624.8647	-33.64	211	224	1	67	R.MIPSVKLDSLGFYR.T

No match to: 1557.7646, 1765.8285, 1861.8424

17. A0A0H0V489 **Mass:** 35934 **Score:** 136 **Expect:** 1e-09 **Queries matched:** 2
A0A0H0V489_BRAHO Flagellar filament protein FlaA OS=Brachyspira hyodysenteriae GN=SZ48_11445 PE=4 SV=1

Observed	Mr(expt)	Mr(calc)	ppm	Start	End	Miss	Ions	Peptide
1241.6952	1240.6879	1240.7292	-33.24	201	210	1	42	R.VLVREPLYPR.M
1625.8173	1624.8100	1624.8647	-33.64	211	224	1	67	R.MIPSVKLDSLGFYR.T

No match to: 1557.7646, 1765.8285, 1861.8424

18. A0A0H0WR23 **Mass:** 35965 **Score:** 136 **Expect:** 1e-09 **Queries matched:** 2
A0A0H0WR23_BRAHO Flagellar filament protein FlaA OS=Brachyspira hyodysenteriae GN=SZ42_05145 PE=4 SV=1

Observed	Mr(expt)	Mr(calc)	ppm	Start	End	Miss	Ions	Peptide
1241.6952	1240.6879	1240.7292	-33.24	201	210	1	42	R.VLVREPLYPR.M
1625.8173	1624.8100	1624.8647	-33.64	211	224	1	67	R.MIPSVKLDSLGFYR.T

No match to: 1557.7646, 1765.8285, 1861.8424

19. G0EJW8 **Mass:** 35935 **Score:** 136 **Expect:** 1e-09 **Queries matched:** 2
G0EJW8_BRAIP Flagellar filament outer layer protein FlaA OS=Brachyspira intermedia (strain ATCC 51140 / PWS/A) GN=Bint_0608 PE=4 SV=1

Observed	Mr(expt)	Mr(calc)	ppm	Start	End	Miss	Ions	Peptide
1241.6952	1240.6879	1240.7292	-33.24	201	210	1	42	R.VLVREPLYPR.M
1625.8173	1624.8100	1624.8647	-33.64	211	224	1	67	R.MIPSVKLDSLGFYR.T

No match to: 1557.7646, 1765.8285, 1861.8424

20. P80158 **Mass:** 2914 **Score:** 130 **Expect:** 4.1e-09 **Queries matched:** 1
FLAB1_BRAHO Flagellar filament core protein flaB1 (Fragment) OS=Brachyspira hyodysenteriae GN=flaB1 PE=1 SV=1

Observed	Mr(expt)	Mr(calc)	ppm	Start	End	Miss	Ions	Peptide
1557.7646	1556.7573	1556.8093	-33.36	1	14	0	107	-.MVINNNISAINAQR.T

No match to: 1241.6952, 1625.8173, 1765.8285, 1861.8424

21. B7T1P4 **Mass:** 24426 **Score:** 122 **Expect:** 2.6e-08 **Queries matched:** 2
B7T1P4_9SPIR FlaA (Fragment) OS=Brachyspira innocens PE=4 SV=1

Observed	Mr(expt)	Mr(calc)	ppm	Start	End	Miss	Ions	Peptide
1241.6952	1240.6879	1240.7292	-33.24	170	179	1	42	R.VLVREPLYPR.M
1625.8173	1624.8100	1624.8647	-33.64	180	193	1	49	R.MVPSIKLDSLGFYR.T

No match to: 1557.7646, 1765.8285, 1861.8424

22. R5LFD4 **Mass:** 32029 **Score:** 120 **Expect:** 4.1e-08 **Queries matched:** 1
R5LFD4_9SPIR Flagellin OS=Brachyspira sp. CAG:700 GN=BN758_02216 PE=3 SV=1

Observed	Mr(expt)	Mr(calc)	ppm	Start	End	Miss	Ions	Peptide
1557.7646	1556.7573	1556.8093	-33.36	1	14	0	107	-.MVINNNISAINAQR.T

No match to: 1241.6952, 1625.8173, 1765.8285, 1861.8424

23. D5U8F3 **Mass:** 35700 **Score:** 118 **Expect:** 6.4e-08 **Queries matched:** 2
D5U8F3_BRAM5 Flagellar filament outer layer protein FlaA OS=Brachyspira murdochii (strain ATCC 51284 / DSM 12563 / 56-150) GN=Bmur_087

Observed	Mr(expt)	Mr(calc)	ppm	Start	End	Miss	Ions	Peptide
1241.6952	1240.6879	1240.7292	-33.24	201	210	1	42	R.VLVREPLYPR.M
1625.8173	1624.8100	1624.8647	-33.64	211	224	1	49	R.MVPSIKLDSLGFYR.T

No match to: 1557.7646, 1765.8285, 1861.8424

24. C0R027 **Mass:** 28784 **Score:** 107 **Expect:** 8.1e-07 **Queries matched:** 1
C0R027_BRAHW Flagellin OS=Brachyspira hyodysenteriae (strain ATCC 49526 / WA1) GN=BHWA1_00982 PE=3 SV=1

Observed	Mr(expt)	Mr(calc)	ppm	Start	End	Miss	Ions	Peptide
1861.8424	1860.8351	1860.8999	-34.83	8	25	1	94	R.INQAGDDASGLAVSEKMR.T

No match to: 1241.6952, 1557.7646, 1625.8173, 1765.8285

25. R5LCF6 **Mass:** 35187 **Score:** 82 **Expect:** 0.00027 **Queries matched:** 1
R5LCF6_9SPIR Flagellar filament outer layer protein FlaA OS=Brachyspira sp. CAG:700 GN=BN758_01892 PE=4 SV=1

Observed	Mr(expt)	Mr(calc)	ppm	Start	End	Miss	Ions	Peptide
1625.8173	1624.8100	1624.8647	-33.64	211	224	1	70	K.LMPSVKIDSIGFYR.T

No match to: 1241.6952, 1557.7646, 1765.8285, 1861.8424

26. B7T1P1 **Mass:** 24517 **Score:** 81 **Expect:** 0.00033 **Queries matched:** 1
B7T1P1_9SPIR FlaA (Fragment) OS=Brachyspira sp. SRBP205IR PE=4 SV=1

Observed	Mr(expt)	Mr(calc)	ppm	Start	End	Miss	Ions	Peptide
1625.8173	1624.8100	1624.8647	-33.64	180	193	1	67	K.MIPSVKLDSLGFYR.T

No match to: 1241.6952, 1557.7646, 1765.8285, 1861.8424

27. B7T1P2 **Mass:** 24517 **Score:** 81 **Expect:** 0.00033 **Queries matched:** 1
B7T1P2_9SPIR FlaA (Fragment) OS=Brachyspira sp. SRBP5IR PE=4 SV=1

Observed	Mr(expt)	Mr(calc)	ppm	Start	End	Miss	Ions	Peptide
1625.8173	1624.8100	1624.8647	-33.64	180	193	1	67	K.MIPSVKLDSLGFYR.T

No match to: 1241.6952, 1557.7646, 1765.8285, 1861.8424

28. J9UWR4 **Mass:** 35678 **Score:** 79 **Expect:** 0.00047 **Queries matched:** 1
J9UWR4_BRAPL Flagellar filament outer layer protein FlaA OS=Brachyspira pilosicoli B2904 GN=B2904_orf2029 PE=4 SV=1

Observed	Mr(expt)	Mr(calc)	ppm	Start - End	Miss	Ions	Peptide
1625.8173	1624.8100	1624.8647	-33.64	211 - 224	1	67	K.MIPSVKLDSLGFYR.T

No match to: 1241.6952, 1557.7646, 1765.8285, 1861.8424

29. D8IBY6 **Mass:** 35707 **Score:** 79 **Expect:** 0.00047 **Queries matched:** 1
D8IBY6_BRAP9 Flagellar filament outer layer protein, FlaA OS=Brachyspira pilosicoli (strain ATCC BAA-1826 / 95/1000) GN=flaA PE=4 SV=1

Observed	Mr(expt)	Mr(calc)	ppm	Start - End	Miss	Ions	Peptide
1625.8173	1624.8100	1624.8647	-33.64	211 - 224	1	67	K.MIPSVKLDSLGFYR.T

No match to: 1241.6952, 1557.7646, 1765.8285, 1861.8424

30. K0JH04 **Mass:** 35709 **Score:** 79 **Expect:** 0.00047 **Queries matched:** 1
K0JH04_BRAPL Flagellar filament outer layer protein FlaA OS=Brachyspira pilosicoli WesB GN=flaA PE=4 SV=1

Observed	Mr(expt)	Mr(calc)	ppm	Start - End	Miss	Ions	Peptide
1625.8173	1624.8100	1624.8647	-33.64	211 - 224	1	67	K.MIPSVKLDSLGFYR.T

No match to: 1241.6952, 1557.7646, 1765.8285, 1861.8424

31. L8XQZ8 **Mass:** 35741 **Score:** 79 **Expect:** 0.00048 **Queries matched:** 1
L8XQZ8_9SPIR Flagellar filament outer layer protein FlaA OS=Brachyspira hampsonii 30599 GN=H263_11839 PE=4 SV=1

Observed	Mr(expt)	Mr(calc)	ppm	Start - End	Miss	Ions	Peptide
1625.8173	1624.8100	1624.8647	-33.64	211 - 224	1	67	R.MIPSVKLDSLGFYR.T

No match to: 1241.6952, 1557.7646, 1765.8285, 1861.8424

32. Q5XPB3 **Mass:** 36034 **Score:** 79 **Expect:** 0.00048 **Queries matched:** 1
Q5XPB3_BRAPL FlaA1 (Fragment) OS=Brachyspira pilosicoli PE=4 SV=1

Observed	Mr(expt)	Mr(calc)	ppm	Start - End	Miss	Ions	Peptide
1625.8173	1624.8100	1624.8647	-33.64	211 - 224	1	67	K.MIPSVKLDSLGFYR.T

No match to: 1241.6952, 1557.7646, 1765.8285, 1861.8424

33. B7T1P5 **Mass:** 24371 **Score:** 63 **Expect:** 0.02 **Queries matched:** 1
B7T1P5_9SPIR FlaA (Fragment) OS=Brachyspira innocens PE=4 SV=1

Observed	Mr(expt)	Mr(calc)	ppm	Start - End	Miss	Ions	Peptide
1625.8173	1624.8100	1624.8647	-33.64	180 - 193	1	49	K.MVPSIKLDSLGFYR.T

No match to: 1241.6952, 1557.7646, 1765.8285, 1861.8424

34. L8XVU3 **Mass:** 52619 **Score:** 62 **Expect:** 0.026 **Queries matched:** 4
L8XVU3_9SPIR Uncharacterized protein OS=Brachyspira hampsonii 30599 GN=H263_01570 PE=4 SV=1

Observed	Mr(expt)	Mr(calc)	ppm	Start - End	Miss	Ions	Peptide
1557.7646	1556.7573	1556.8059	-31.20	182 - 194	1	---	K.IRSYHAAQQNIEK.A
1625.8173	1624.8100	1624.7468	38.9	225 - 238	0	---	K.ELEIAYESNEVSDK.D
1765.8285	1764.8212	1764.7851	20.5	61 - 74	0	---	K.DANILWMTANMYYK.Q + 2 Oxidation (M)
1861.8424	1860.8351	1860.8902	-29.60	60 - 74	1	---	K.KDANILWMTANMYYK.Q

No match to: 1241.6952

35. L0X013 **Mass:** 52663 **Score:** 62 **Expect:** 0.026 **Queries matched:** 4
L0X013_9SPIR Uncharacterized protein OS=Brachyspira hampsonii 30446 GN=A966_10572 PE=4 SV=1

Observed	Mr(expt)	Mr(calc)	ppm	Start - End	Miss	Ions	Peptide
1557.7646	1556.7573	1556.8059	-31.20	182 - 194	1	---	K.IRSYHAAQQNIEK.A
1625.8173	1624.8100	1624.7468	38.9	225 - 238	0	---	K.ELEIAYESNEVSDK.D
1765.8285	1764.8212	1764.7851	20.5	61 - 74	0	---	K.DANILWMTANMYYK.Q + 2 Oxidation (M)
1861.8424	1860.8351	1860.8902	-29.60	60 - 74	1	---	K.KDANILWMTANMYYK.Q

No match to: 1241.6952

36. B7T1P7 **Mass:** 24514 **Score:** 55 **Expect:** 0.13 **Queries matched:** 1
B7T1P7_9SPIR FlaA (Fragment) OS=Brachyspira canis PE=4 SV=1

Observed	Mr(expt)	Mr(calc)	ppm	Start - End	Miss	Ions	Peptide
1241.6952	1240.6879	1240.7292	-33.24	170 - 179	1	42	R.VLVREPLYPR.M

No match to: 1557.7646, 1625.8173, 1765.8285, 1861.8424

Search Parameters

Type of search : Sequence Query
Enzyme : Trypsin
Fixed modifications : Carbamidomethyl (C)
Variable modifications : Oxidation (M)
Mass values : Monoisotopic
Protein Mass : Unrestricted
Peptide Mass Tolerance : ± 50 ppm
Fragment Mass Tolerance: ± 0.5 Da
Max Missed Cleavages : 1
Instrument type : MALDI-TOF-TOF
Query1 (1241.6952,1+) : Locus:27.1.1.211174.56646
Query2 (1557.7646,1+) : Locus:27.1.1.211174.56645
Query3 (1625.8173,1+) : Locus:27.1.1.211174.56647
Query4 (1765.8285,1+) : Locus:27.1.1.211174.56649
Query5 (1861.8424,1+) : Locus:27.1.1.211174.56648

Mascot: http://www.matrixscience.com/

MATRIX SCIENCE Mascot Search Results

```
User          :
Email         :
Search title  : F:\ROD_inmunoproteoma\MALDI\ppw_2016_562_563_ROD\ppw_I15_145397050431.txt
MS data file  : F:\ROD_inmunoproteoma\MALDI\ppw_2016_562_563_ROD\ppw_I15_145397050431.txt
Database      : Brachyspiraceae Brachyspiraceae_20160127-151122 (40573 sequences; 13470793 residues)
Timestamp     : 23 Feb 2016 at 16:06:22 GMT
Warning       : A Peptide summary report will usually give a much clearer picture of MS/MS search results.
Top Score     : 752 for Q26501, Q26501_BRAHO Flagellin OS=Brachyspira hyodysenteriae GN=flaB1 PE=3 SV=1
```

Probability Based Mowse Score

Protein score is -10*Log(P), where P is the probability that the observed match is a random event.
Protein scores greater than 59 are significant (p<0.05).
Protein scores are derived from ions scores as a non-probabilistic basis for ranking protein hits.

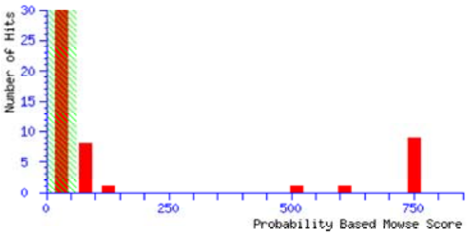

Protein Summary Report

Format As | Protein Summary (deprecated) | Help

Significance threshold p< 0.05 Max. number of hits AUTO

Standard scoring ◉ MudPIT scoring ○ Ions score or expect cut-off 0 Show sub-sets 0

Show pop-ups ◉ Suppress pop-ups ○ Sort unassigned Decreasing Score Require bold red ☐

Re-Search All | Search Unmatched

Index

	Accession	Mass	Score	Description
1.	Q26501	31952	752	Q26501_BRAHO Flagellin OS=Brachyspira hyodysenteriae GN=flaB1 PE=3 SV=1
2.	D5U7D6	31944	752	D5U7D6_BRAM5 Flagellin OS=Brachyspira murdochii (strain ATCC 51284 / DSM 12563 / 56-150) GN=Bmur_0623 PE=3 SV=
3.	A0A0G4K7S6	31933	751	A0A0G4K7S6_9SPIR Flagellin OS=Brachyspira suanatina GN=flaB2 PE=3 SV=1
4.	G0EKN9	31889	751	G0EKN9_BRAIP Flagellin OS=Brachyspira intermedia (strain ATCC 51140 / PWS/A) GN=Bint_2009 PE=3 SV=1
5.	J9UV65	32001	751	J9UV65_BRAPL Flagellin OS=Brachyspira pilosicoli B2904 GN=flaB2 PE=3 SV=1
6.	D8IAP2	32001	751	D8IAP2_BRAP9 Flagellin OS=Brachyspira pilosicoli (strain ATCC BAA-1826 / 95/1000) GN=flaB2 PE=3 SV=1
7.	K0JFK3	32001	751	K0JFK3_BRAPL Flagellin OS=Brachyspira pilosicoli WesB GN=flaB2-1 PE=3 SV=1
8.	L8XRQ5	31949	751	L8XRQ5_9SPIR Flagellin OS=Brachyspira hampsonii 30599 GN=H263_12719 PE=3 SV=1
9.	L0X542	31951	751	L0X542_9SPIR Flagellin OS=Brachyspira hampsonii 30446 GN=A966_08614 PE=3 SV=1
10.	C0R027	28784	617	C0R027_BRAHW Flagellin OS=Brachyspira hyodysenteriae (strain ATCC 49526 / WA1) GN=BHWA1_00982 PE=3 SV=1
11.	R5LFD4	32029	534	R5LFD4_9SPIR Flagellin OS=Brachyspira sp. CAG:700 GN=BN758_02216 PE=3 SV=1
12.	P80158	2914	137	FLAB1_BRAHO Flagellar filament core protein flaB1 (Fragment) OS=Brachyspira hyodysenteriae GN=flaB1 PE=1 SV=1
13.	Q7M132	2814	70	Q7M132_BRAHO Flagellar core protein, 34K (Fragment) OS=Brachyspira hyodysenteriae PE=1 SV=1
14.	P80160	31113	60	FLAB2_BRAHO Flagellar filament core protein flaB2 OS=Brachyspira hyodysenteriae GN=flaB2 PE=1 SV=1
15.	A0A0H0X544	31129	60	A0A0H0X544_BRAHO Flagellin OS=Brachyspira hyodysenteriae GN=SU43_01870 PE=3 SV=1
16.	A0A0G4K9G4	31184	60	A0A0G4K9G4_9SPIR Flagellin OS=Brachyspira suanatina GN=flaB2 PE=3 SV=1
17.	A0A0H0ULZ7	31057	60	A0A0H0ULZ7_BRAHO Flagellin OS=Brachyspira hyodysenteriae GN=SR30_04500 PE=3 SV=1
18.	D5UB01	31218	60	D5UB01_BRAM5 Flagellin OS=Brachyspira murdochii (strain ATCC 51284 / DSM 12563 / 56-150) GN=Bmur_1792 PE=3 SV=
19.	L0X2I6	31181	60	L0X2I6_9SPIR Flagellin OS=Brachyspira hampsonii 30446 GN=A966_05778 PE=3 SV=1
20.	L8XTT2	31194	60	L8XTT2_9SPIR Flagellin OS=Brachyspira hampsonii 30599 GN=H263_08389 PE=3 SV=1
21.	D5UAW5	36825	37	D5UAW5_BRAM5 Bifunctional ligase/repressor BirA OS=Brachyspira murdochii (strain ATCC 51284 / DSM 12563 / 56-1

Results List

1. Q26501 **Mass:** 31952 **Score:** 752 **Expect:** 2.6e-71 **Queries matched:** 4
Q26501_BRAHO Flagellin OS=Brachyspira hyodysenteriae GN=flaB1 PE=3 SV=1

Observed	Mr(expt)	Mr(calc)	ppm	Start	End	Miss	Ions	Peptide
1557.7769	1556.7696	1556.8093	-25.46	1 -	14	0	115	-.MVINNNISAINAQR.T
1861.8401	1860.8328	1860.8999	-36.07	36 -	53	1	196	R.INQAGDDASGLAVSEKMR.T
2607.2751	2606.2678	2606.3598	-35.30	264 -	287	0	166	K.DQILNQANLSMLAQANQLPQGALR.L
2919.4995	2918.4922	2918.5759	-28.68	261 -	287	1	200	K.LAKDQILNQANLSMLAQANQLPQGALR.L

No match to: 1571.7748

2. D5U7D6 **Mass:** 31944 **Score:** 752 **Expect:** 2.6e-71 **Queries matched:** 4
D5U7D6_BRAM5 Flagellin OS=Brachyspira murdochii (strain ATCC 51284 / DSM 12563 / 56-150) GN=Bmur_0623 PE=3 SV=1

Observed	Mr(expt)	Mr(calc)	ppm	Start	End	Miss	Ions	Peptide
1557.7769	1556.7696	1556.8093	-25.46	1 -	14	0	115	-.MVINNNISAINAQR.T
1861.8401	1860.8328	1860.8999	-36.07	36 -	53	1	196	R.INQAGDDASGLAVSEKMR.T
2607.2751	2606.2678	2606.3598	-35.30	264 -	287	0	166	K.DQILNQANLSMLAQANQLPQGALR.L
2919.4995	2918.4922	2918.5759	-28.68	261 -	287	1	200	K.LAKDQILNQANLSMLAQANQLPQGALR.L

No match to: 1571.7748

3. A0A0G4K7S6 **Mass:** 31933 **Score:** 751 **Expect:** 3.2e-71 **Queries matched:** 4

A0A0G4K7S6_9SPIR Flagellin OS=Brachyspira suanatina GN=flaB2 PE=3 SV=1

Observed	Mr(expt)	Mr(calc)	ppm	Start	End	Miss	Ions	Peptide
1557.7769	1556.7696	1556.8093	-25.46	1 -	14	0	115	-.MVINNNISAINAQR.T
1861.8401	1860.8328	1860.8999	-36.07	36 -	53	1	196	R.INQAGDDASGLAVSEKMR.T
2607.2751	2606.2678	2606.3598	-35.30	264 -	287	0	166	K.DQILNQANLSMLAQANQLPQGALR.L
2919.4995	2918.4922	2918.5759	-28.68	261 -	287	1	200	K.LAKDQILNQANLSMLAQANQLPQGALR.L

No match to: 1571.7748

4. G0EKN9 **Mass:** 31889 **Score:** 751 **Expect:** 3.2e-71 **Queries matched:** 4
G0EKN9_BRAIP Flagellin OS=Brachyspira intermedia (strain ATCC 51140 / PWS/A) GN=Bint_2009 PE=3 SV=1

Observed	Mr(expt)	Mr(calc)	ppm	Start	End	Miss	Ions	Peptide
1557.7769	1556.7696	1556.8093	-25.46	1 -	14	0	115	-.MVINNNISAINAQR.T
1861.8401	1860.8328	1860.8999	-36.07	36 -	53	1	196	R.INQAGDDASGLAVSEKMR.T
2607.2751	2606.2678	2606.3598	-35.30	264 -	287	0	166	K.DQILNQANLSMLAQANQLPQGALR.L
2919.4995	2918.4922	2918.5759	-28.68	261 -	287	1	200	K.LAKDQILNQANLSMLAQANQLPQGALR.L

No match to: 1571.7748

5. J9UV65 **Mass:** 32001 **Score:** 751 **Expect:** 3.2e-71 **Queries matched:** 4
J9UV65_BRAPL Flagellin OS=Brachyspira pilosicoli B2904 GN=flaB2 PE=3 SV=1

Observed	Mr(expt)	Mr(calc)	ppm	Start	End	Miss	Ions	Peptide
1557.7769	1556.7696	1556.8093	-25.46	1 -	14	0	115	-.MVINNNISAINAQR.T
1861.8401	1860.8328	1860.8999	-36.07	36 -	53	1	196	R.INQAGDDASGLAVSEKMR.T
2607.2751	2606.2678	2606.3598	-35.30	264 -	287	0	166	K.DQILNQANLSMLAQANQLPQGALR.L
2919.4995	2918.4922	2918.5759	-28.68	261 -	287	1	200	K.LAKDQILNQANLSMLAQANQLPQGALR.L

No match to: 1571.7748

6. D8IAP2 **Mass:** 32001 **Score:** 751 **Expect:** 3.2e-71 **Queries matched:** 4
D8IAP2_BRAP9 Flagellin OS=Brachyspira pilosicoli (strain ATCC BAA-1826 / 95/1000) GN=flaB2 PE=3 SV=1

Observed	Mr(expt)	Mr(calc)	ppm	Start	End	Miss	Ions	Peptide
1557.7769	1556.7696	1556.8093	-25.46	1 -	14	0	115	-.MVINNNISAINAQR.T
1861.8401	1860.8328	1860.8999	-36.07	36 -	53	1	196	R.INQAGDDASGLAVSEKMR.T
2607.2751	2606.2678	2606.3598	-35.30	264 -	287	0	166	K.DQILNQANLSMLAQANQLPQGALR.L
2919.4995	2918.4922	2918.5759	-28.68	261 -	287	1	200	K.LAKDQILNQANLSMLAQANQLPQGALR.L

No match to: 1571.7748

7. K0JFK3 **Mass:** 32001 **Score:** 751 **Expect:** 3.2e-71 **Queries matched:** 4
K0JFK3_BRAPL Flagellin OS=Brachyspira pilosicoli WesB GN=flaB2-1 PE=3 SV=1

Observed	Mr(expt)	Mr(calc)	ppm	Start	End	Miss	Ions	Peptide
1557.7769	1556.7696	1556.8093	-25.46	1 -	14	0	115	-.MVINNNISAINAQR.T
1861.8401	1860.8328	1860.8999	-36.07	36 -	53	1	196	R.INQAGDDASGLAVSEKMR.T
2607.2751	2606.2678	2606.3598	-35.30	264 -	287	0	166	K.DQILNQANLSMLAQANQLPQGALR.L
2919.4995	2918.4922	2918.5759	-28.68	261 -	287	1	200	K.LAKDQILNQANLSMLAQANQLPQGALR.L

No match to: 1571.7748

8. L8XRQ5 **Mass:** 31949 **Score:** 751 **Expect:** 3.2e-71 **Queries matched:** 4
L8XRQ5_9SPIR Flagellin OS=Brachyspira hampsonii 30599 GN=H263_12719 PE=3 SV=1

Observed	Mr(expt)	Mr(calc)	ppm	Start	End	Miss	Ions	Peptide
1557.7769	1556.7696	1556.8093	-25.46	1 -	14	0	115	-.MVINNNISAINAQR.T
1861.8401	1860.8328	1860.8999	-36.07	36 -	53	1	196	R.INQAGDDASGLAVSEKMR.T
2607.2751	2606.2678	2606.3598	-35.30	264 -	287	0	166	K.DQILNQANLSMLAQANQLPQGALR.L
2919.4995	2918.4922	2918.5759	-28.68	261 -	287	1	200	K.LAKDQILNQANLSMLAQANQLPQGALR.L

No match to: 1571.7748

9. L0X542 **Mass:** 31951 **Score:** 751 **Expect:** 3.2e-71 **Queries matched:** 4
L0X542_9SPIR Flagellin OS=Brachyspira hampsonii 30446 GN=A966_08614 PE=3 SV=1

Observed	Mr(expt)	Mr(calc)	ppm	Start	End	Miss	Ions	Peptide
1557.7769	1556.7696	1556.8093	-25.46	1 -	14	0	115	-.MVINNNISAINAQR.T
1861.8401	1860.8328	1860.8999	-36.07	36 -	53	1	196	R.INQAGDDASGLAVSEKMR.T
2607.2751	2606.2678	2606.3598	-35.30	264 -	287	0	166	K.DQILNQANLSMLAQANQLPQGALR.L
2919.4995	2918.4922	2918.5759	-28.68	261 -	287	1	200	K.LAKDQILNQANLSMLAQANQLPQGALR.L

No match to: 1571.7748

10. C0R027 **Mass:** 28784 **Score:** 617 **Expect:** 8.1e-58 **Queries matched:** 3
C0R027_BRAHW Flagellin OS=Brachyspira hyodysenteriae (strain ATCC 49526 / WA1) GN=BHWA1_00982 PE=3 SV=1

Observed	Mr(expt)	Mr(calc)	ppm	Start	End	Miss	Ions	Peptide
1861.8401	1860.8328	1860.8999	-36.07	8 -	25	1	196	R.INQAGDDASGLAVSEKMR.T
2607.2751	2606.2678	2606.3598	-35.30	236 -	259	0	166	K.DQILNQANLSMLAQANQLPQGALR.L
2919.4995	2918.4922	2918.5759	-28.68	233 -	259	1	200	K.LAKDQILNQANLSMLAQANQLPQGALR.L

No match to: 1557.7769, 1571.7748

11. R5LFD4 **Mass:** 32029 **Score:** 534 **Expect:** 1.6e-49 **Queries matched:** 3
R5LFD4_9SPIR Flagellin OS=Brachyspira sp. CAG:700 GN=BN758_02216 PE=3 SV=1

Observed	Mr(expt)	Mr(calc)	ppm	Start	End	Miss	Ions	Peptide
1557.7769	1556.7696	1556.8093	-25.46	1 -	14	0	115	-.MVINNNISAINAQR.T
2607.2751	2606.2678	2606.3598	-35.30	264 -	287	0	166	K.DQILNQANLSMLAQANQLPQGALR.L
2919.4995	2918.4922	2918.5759	-28.68	261 -	287	1	200	K.LAKDQILNQANLSMLAQANQLPQGALR.L

No match to: 1571.7748, 1861.8401

12. P80158 **Mass:** 2914 **Score:** 137 **Expect:** 8.1e-10 **Queries matched:** 1
FLAB1_BRAHO Flagellar filament core protein flaB1 (Fragment) OS=Brachyspira hyodysenteriae GN=flaB1 PE=1 SV=1

Observed	Mr(expt)	Mr(calc)	ppm	Start	End	Miss	Ions	Peptide
1557.7769	1556.7696	1556.8093	-25.46	1 -	14	0	115	-.MVINNNISAINAQR.T

No match to: 1571.7748, 1861.8401, 2607.2751, 2919.4995

13. Q7M132 **Mass:** 2814 **Score:** 70 **Expect:** 0.0046 **Queries matched:** 1
Q7M132_BRAHO Flagellar core protein, 34K (Fragment) OS=Brachyspira hyodysenteriae PE=1 SV=1

Observed	Mr(expt)	Mr(calc)	ppm	Start		End	Miss	Ions	Peptide
1571.7748	1570.7675	1570.8249	-36.53	1	-	14	0	47	-.MIINNNISAINAQR.T

No match to: 1557.7769, 1861.8401, 2607.2751, 2919.4995

14. P80160 **Mass:** 31113 **Score:** 60 **Expect:** 0.046 **Queries matched:** 1
FLAB2_BRAHO Flagellar filament core protein flaB2 OS=Brachyspira hyodysenteriae GN=flaB2 PE=1 SV=1

Observed	Mr(expt)	Mr(calc)	ppm	Start		End	Miss	Ions	Peptide
1571.7748	1570.7675	1570.8249	-36.53	1	-	14	0	47	-.MIINNNISAINAQR.T

No match to: 1557.7769, 1861.8401, 2607.2751, 2919.4995

15. A0A0H0X544 **Mass:** 31129 **Score:** 60 **Expect:** 0.046 **Queries matched:** 1
A0A0H0X544_BRAHO Flagellin OS=Brachyspira hyodysenteriae GN=SU43_01870 PE=3 SV=1

Observed	Mr(expt)	Mr(calc)	ppm	Start		End	Miss	Ions	Peptide
1571.7748	1570.7675	1570.8249	-36.53	1	-	14	0	47	-.MIINNNISAINAQR.T

No match to: 1557.7769, 1861.8401, 2607.2751, 2919.4995

16. A0A0G4K9G4 **Mass:** 31184 **Score:** 60 **Expect:** 0.046 **Queries matched:** 1
A0A0G4K9G4_9SPIR Flagellin OS=Brachyspira suanatina GN=flaB2 PE=3 SV=1

Observed	Mr(expt)	Mr(calc)	ppm	Start		End	Miss	Ions	Peptide
1571.7748	1570.7675	1570.8249	-36.53	1	-	14	0	47	-.MIINNNISAINAQR.T

No match to: 1557.7769, 1861.8401, 2607.2751, 2919.4995

17. A0A0H0ULZ7 **Mass:** 31057 **Score:** 60 **Expect:** 0.046 **Queries matched:** 1
A0A0H0ULZ7_BRAHO Flagellin OS=Brachyspira hyodysenteriae GN=SR30_04500 PE=3 SV=1

Observed	Mr(expt)	Mr(calc)	ppm	Start		End	Miss	Ions	Peptide
1571.7748	1570.7675	1570.8249	-36.53	1	-	14	0	47	-.MIINNNISAINAQR.T

No match to: 1557.7769, 1861.8401, 2607.2751, 2919.4995

18. D5UB01 **Mass:** 31218 **Score:** 60 **Expect:** 0.046 **Queries matched:** 1
D5UB01_BRAM5 Flagellin OS=Brachyspira murdochii (strain ATCC 51284 / DSM 12563 / 56-150) GN=Bmur_1792 PE=3 SV=1

Observed	Mr(expt)	Mr(calc)	ppm	Start		End	Miss	Ions	Peptide
1571.7748	1570.7675	1570.8249	-36.53	1	-	14	0	47	-.MIINNNISAINAQR.T

No match to: 1557.7769, 1861.8401, 2607.2751, 2919.4995

19. L0X2I6 **Mass:** 31181 **Score:** 60 **Expect:** 0.046 **Queries matched:** 1
L0X2I6_9SPIR Flagellin OS=Brachyspira hampsonii 30446 GN=A966_05778 PE=3 SV=1

Observed	Mr(expt)	Mr(calc)	ppm	Start		End	Miss	Ions	Peptide
1571.7748	1570.7675	1570.8249	-36.53	1	-	14	0	47	-.MIINNNISAINAQR.T

No match to: 1557.7769, 1861.8401, 2607.2751, 2919.4995

20. L8XTT2 **Mass:** 31194 **Score:** 60 **Expect:** 0.046 **Queries matched:** 1
L8XTT2_9SPIR Flagellin OS=Brachyspira hampsonii 30599 GN=H263_08389 PE=3 SV=1

Observed	Mr(expt)	Mr(calc)	ppm	Start		End	Miss	Ions	Peptide
1571.7748	1570.7675	1570.8249	-36.53	1	-	14	0	47	-.MIINNNISAINAQR.T

No match to: 1557.7769, 1861.8401, 2607.2751, 2919.4995

21. D5UAW5 **Mass:** 36825 **Score:** 37 **Expect:** 8.5 **Queries matched:** 2
D5UAW5_BRAM5 Bifunctional ligase/repressor BirA OS=Brachyspira murdochii (strain ATCC 51284 / DSM 12563 / 56-150) GN=birA PE=3 SV=1

Observed	Mr(expt)	Mr(calc)	ppm	Start		End	Miss	Ions	Peptide
1557.7769	1556.7696	1556.8311	-39.47	26	-	39	1	7	K.LADDLNVSRAAVWK.A
2607.2751	2606.2678	2606.2751	-2.81	291	-	313	0	---	K.ENIINVLDIDENFALVAEFEDGK.V

No match to: 1571.7748, 1861.8401, 2919.4995

Search Parameters

Type of search : Sequence Query
Enzyme : Trypsin
Fixed modifications : Carbamidomethyl (C)
Variable modifications : Oxidation (M)
Mass values : Monoisotopic
Protein Mass : Unrestricted
Peptide Mass Tolerance : ± 50 ppm
Fragment Mass Tolerance: ± 0.5 Da
Max Missed Cleavages : 1
Instrument type : MALDI-TOF-TOF
Query1 (1557.7769,1+) : Locus:32.1.1.211179.56670
Query2 (1571.7748,1+) : Locus:32.1.1.211179.56674
Query3 (1861.8401,1+) : Locus:32.1.1.211179.56672
Query4 (2607.2751,1+) : Locus:32.1.1.211179.56673
Query5 (2919.4995,1+) : Locus:32.1.1.211179.56671

Mascot: http://www.matrixscience.com/

MATRIX SCIENCE Mascot Search Results

User	:
Email	:
Search title	: F:\ROD_inmunoproteoma\MALDI\ppw_2016_562_563_ROD\ppw_I21_145397050937.txt
MS data file	: F:\ROD_inmunoproteoma\MALDI\ppw_2016_562_563_ROD\ppw_I21_145397050937.txt
Database	: Brachyspiraceae Brachyspiraceae_20160127-151122 (40573 sequences; 13470793 residues)
Timestamp	: 23 Feb 2016 at 16:06:37 GMT
Warning	: A Peptide summary report will usually give a much clearer picture of MS/MS search results.
Top Score	: 263 for Q26501, Q26501_BRAHO Flagellin OS=Brachyspira hyodysenteriae GN=flaB1 PE=3 SV=1

Probability Based Mowse Score

Protein score is -10*Log(P), where P is the probability that the observed match is a random event.
Protein scores greater than 59 are significant (p<0.05).
Protein scores are derived from ions scores as a non-probabilistic basis for ranking protein hits.

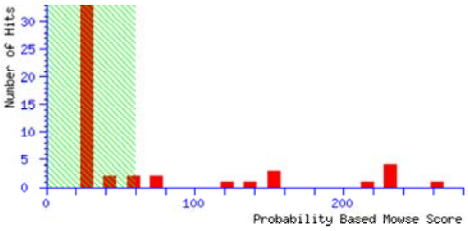

Protein Summary Report

Format As | Protein Summary (deprecated) | Help

Significance threshold p< 0.05 | Max. number of hits AUTO

Standard scoring ◉ MudPIT scoring ○ | Ions score or expect cut-off 0 | Show sub-sets 0

Show pop-ups ◉ Suppress pop-ups ○ | Sort unassigned Decreasing Score | Require bold red ☐

Re-Search All | Search Unmatched

Index

	Accession	Mass	Score	Description
1.	Q26501	31952	263	Q26501_BRAHO Flagellin OS=Brachyspira hyodysenteriae GN=flaB1 PE=3 SV=1
2.	A0A0G4K7S6	31933	232	A0A0G4K7S6_9SPIR Flagellin OS=Brachyspira suanatina GN=flaB2 PE=3 SV=1
3.	G0EKN9	31889	232	G0EKN9_BRAIP Flagellin OS=Brachyspira intermedia (strain ATCC 51140 / PWS/A) GN=Bint_2009 PE=3 SV=1
4.	L8XRQ5	31949	232	L8XRQ5_9SPIR Flagellin OS=Brachyspira hampsonii 30599 GN=H263_12719 PE=3 SV=1
5.	L0X542	31951	232	L0X542_9SPIR Flagellin OS=Brachyspira hampsonii 30446 GN=A966_08614 PE=3 SV=1
6.	D5U7D6	31944	210	D5U7D6_BRAM5 Flagellin OS=Brachyspira murdochii (strain ATCC 51284 / DSM 12563 / 56-150) GN=Bmur_0623 PE=3 SV=
7.	J9UV65	32001	146	J9UV65_BRAPL Flagellin OS=Brachyspira pilosicoli B2904 GN=flaB2 PE=3 SV=1
8.	D8IAP2	32001	146	D8IAP2_BRAP9 Flagellin OS=Brachyspira pilosicoli (strain ATCC BAA-1826 / 95/1000) GN=flaB2 PE=3 SV=1
9.	K0JFK3	32001	146	K0JFK3_BRAPL Flagellin OS=Brachyspira pilosicoli WesB GN=flaB2-1 PE=3 SV=1
10.	P80158	2914	137	FLAB1_BRAHO Flagellar filament core protein flaB1 (Fragment) OS=Brachyspira hyodysenteriae GN=flaB1 PE=1 SV=1
11.	R5LFD4	32029	127	R5LFD4_9SPIR Flagellin OS=Brachyspira sp. CAG:700 GN=BN758_02216 PE=3 SV=1
12.	C0R027	28784	80	C0R027_BRAHW Flagellin OS=Brachyspira hyodysenteriae (strain ATCC 49526 / WA1) GN=BHWA1_00982 PE=3 SV=1
13.	D5U7R2	30130	80	D5U7R2_BRAM5 Flagellin OS=Brachyspira murdochii (strain ATCC 51284 / DSM 12563 / 56-150) GN=Bmur_2791 PE=3 SV=
14.	J9UYC2	31304	60	J9UYC2_BRAPL Flagellin OS=Brachyspira pilosicoli B2904 GN=B2904_orf2581 PE=3 SV=1
15.	K0JHQ4	31288	60	K0JHQ4_BRAPL Flagellin OS=Brachyspira pilosicoli WesB GN=flaB2 PE=3 SV=1
16.	R5LEF1	33306	50	R5LEF1_9SPIR Peptidase M23 OS=Brachyspira sp. CAG:700 GN=BN758_01473 PE=4 SV=1

Results List

1. Q26501 **Mass:** 31952 **Score:** 263 **Expect:** 2e-22 **Queries matched:** 3
Q26501_BRAHO Flagellin OS=Brachyspira hyodysenteriae GN=flaB1 PE=3 SV=1

Observed	Mr(expt)	Mr(calc)	ppm	Start	End	Miss	Ions	Peptide
1023.4379	1022.4306	1022.4781	-46.44	219 -	227	0	68	R.SDLGAYQNR.L
1054.4423	1053.4350	1053.4583	-22.09	26 -	35	0	34	K.DSAMISSGMR.I
1557.7401	1556.7328	1556.8093	-49.10	1 -	14	0	115	-.MVINNNISAINAQR.T

No match to: 1569.8116, 2607.2344

2. A0A0G4K7S6 **Mass:** 31933 **Score:** 232 **Expect:** 2.6e-19 **Queries matched:** 3
A0A0G4K7S6_9SPIR Flagellin OS=Brachyspira suanatina GN=flaB2 PE=3 SV=1

Observed	Mr(expt)	Mr(calc)	ppm	Start	End	Miss	Ions	Peptide
1023.4379	1022.4306	1022.4781	-46.44	219 -	227	0	68	R.SDLGAYQNR.L
1557.7401	1556.7328	1556.8093	-49.10	1 -	14	0	115	-.MVINNNISAINAQR.T
2607.2344	2606.2271	2606.2242	1.12	26 -	51	1	---	K.DAAQISSGMRINQAGDDASGLAVSEK.M + Oxidation (M)

No match to: 1054.4423, 1569.8116

3. G0EKN9 **Mass:** 31889 **Score:** 232 **Expect:** 2.6e-19 **Queries matched:** 3
G0EKN9_BRAIP Flagellin OS=Brachyspira intermedia (strain ATCC 51140 / PWS/A) GN=Bint_2009 PE=3 SV=1

Observed	Mr(expt)	Mr(calc)	ppm	Start	End	Miss	Ions	Peptide
1023.4379	1022.4306	1022.4781	-46.44	219 -	227	0	68	R.SDLGAYQNR.L
1557.7401	1556.7328	1556.8093	-49.10	1 -	14	0	115	-.MVINNNISAINAQR.T
2607.2344	2606.2271	2606.2242	1.12	26 -	51	1	---	K.DAAQISSGMRINQAGDDASGLAVSEK.M + Oxidation (M)

No match to: 1054.4423, 1569.8116

4. L8XRQ5 **Mass:** 31949 **Score:** 232 **Expect:** 2.6e-19 **Queries matched:** 3
L8XRQ5_9SPIR Flagellin OS=Brachyspira hampsonii 30599 GN=H263_12719 PE=3 SV=1

Observed	Mr(expt)	Mr(calc)	ppm	Start	End	Miss	Ions	Peptide
1023.4379	1022.4306	1022.4781	-46.44	219 -	227	0	68	R.SDLGAYQNR.L
1557.7401	1556.7328	1556.8093	-49.10	1 -	14	0	115	-.MVINNNISAINAQR.T
2607.2344	2606.2271	2606.2242	1.12	26 -	51	1	---	K.DAAQISSGMRINQAGDDASGLAVSEK.M + Oxidation (M)

No match to: 1054.4423, 1569.8116

5. L0X542 **Mass:** 31951 **Score:** 232 **Expect:** 2.6e-19 **Queries matched:** 3
L0X542_9SPIR Flagellin OS=Brachyspira hampsonii 30446 GN=A966_08614 PE=3 SV=1

Observed	Mr(expt)	Mr(calc)	ppm	Start	End	Miss	Ions	Peptide
1023.4379	1022.4306	1022.4781	-46.44	219 -	227	0	68	R.SDLGAYQNR.L
1557.7401	1556.7328	1556.8093	-49.10	1 -	14	0	115	-.MVINNNISAINAQR.T
2607.2344	2606.2271	2606.2242	1.12	26 -	51	1	---	K.DAAQISSGMRINQAGDDASGLAVSEK.M + Oxidation (M)

No match to: 1054.4423, 1569.8116

6. D5U7D6 **Mass:** 31944 **Score:** 210 **Expect:** 4.1e-17 **Queries matched:** 2
D5U7D6_BRAM5 Flagellin OS=Brachyspira murdochii (strain ATCC 51284 / DSM 12563 / 56-150) GN=Bmur_0623 PE=3 SV=1

Observed	Mr(expt)	Mr(calc)	ppm	Start	End	Miss	Ions	Peptide
1023.4379	1022.4306	1022.4781	-46.44	219 -	227	0	68	R.SDLGAYQNR.L
1557.7401	1556.7328	1556.8093	-49.10	1 -	14	0	115	-.MVINNNISAINAQR.T

No match to: 1054.4423, 1569.8116, 2607.2344

7. J9UV65 **Mass:** 32001 **Score:** 146 **Expect:** 1e-10 **Queries matched:** 2
J9UV65_BRAPL Flagellin OS=Brachyspira pilosicoli B2904 GN=flaB2 PE=3 SV=1

Observed	Mr(expt)	Mr(calc)	ppm	Start	End	Miss	Ions	Peptide
1557.7401	1556.7328	1556.8093	-49.10	1 -	14	0	115	-.MVINNNISAINAQR.T
2607.2344	2606.2271	2606.2242	1.12	26 -	51	1	---	K.DAAQISSGMRINQAGDDASGLAVSEK.M + Oxidation (M)

No match to: 1023.4379, 1054.4423, 1569.8116

8. D8IAP2 **Mass:** 32001 **Score:** 146 **Expect:** 1e-10 **Queries matched:** 2
D8IAP2_BRAP9 Flagellin OS=Brachyspira pilosicoli (strain ATCC BAA-1826 / 95/1000) GN=flaB2 PE=3 SV=1

Observed	Mr(expt)	Mr(calc)	ppm	Start	End	Miss	Ions	Peptide
1557.7401	1556.7328	1556.8093	-49.10	1 -	14	0	115	-.MVINNNISAINAQR.T
2607.2344	2606.2271	2606.2242	1.12	26 -	51	1	---	K.DAAQISSGMRINQAGDDASGLAVSEK.M + Oxidation (M)

No match to: 1023.4379, 1054.4423, 1569.8116

9. K0JFK3 **Mass:** 32001 **Score:** 146 **Expect:** 1e-10 **Queries matched:** 2
K0JFK3_BRAPL Flagellin OS=Brachyspira pilosicoli WesB GN=flaB2-1 PE=3 SV=1

Observed	Mr(expt)	Mr(calc)	ppm	Start	End	Miss	Ions	Peptide
1557.7401	1556.7328	1556.8093	-49.10	1 -	14	0	115	-.MVINNNISAINAQR.T
2607.2344	2606.2271	2606.2242	1.12	26 -	51	1	---	K.DAAQISSGMRINQAGDDASGLAVSEK.M + Oxidation (M)

No match to: 1023.4379, 1054.4423, 1569.8116

10. P80158 **Mass:** 2914 **Score:** 137 **Expect:** 8.1e-10 **Queries matched:** 1
FLAB1_BRAHO Flagellar filament core protein flaB1 (Fragment) OS=Brachyspira hyodysenteriae GN=flaB1 PE=1 SV=1

Observed	Mr(expt)	Mr(calc)	ppm	Start	End	Miss	Ions	Peptide
1557.7401	1556.7328	1556.8093	-49.10	1 -	14	0	115	-.MVINNNISAINAQR.T

No match to: 1023.4379, 1054.4423, 1569.8116, 2607.2344

11. R5LFD4 **Mass:** 32029 **Score:** 127 **Expect:** 8.1e-09 **Queries matched:** 1
R5LFD4_9SPIR Flagellin OS=Brachyspira sp. CAG:700 GN=BN758_02216 PE=3 SV=1

Observed	Mr(expt)	Mr(calc)	ppm	Start	End	Miss	Ions	Peptide
1557.7401	1556.7328	1556.8093	-49.10	1 -	14	0	115	-.MVINNNISAINAQR.T

No match to: 1023.4379, 1054.4423, 1569.8116, 2607.2344

12. C0R027 **Mass:** 28784 **Score:** 80 **Expect:** 0.00041 **Queries matched:** 1
C0R027_BRAHW Flagellin OS=Brachyspira hyodysenteriae (strain ATCC 49526 / WA1) GN=BHWA1_00982 PE=3 SV=1

Observed	Mr(expt)	Mr(calc)	ppm	Start	End	Miss	Ions	Peptide
1023.4379	1022.4306	1022.4781	-46.44	191 -	199	0	68	R.SDLGAYQNR.L

No match to: 1054.4423, 1557.7401, 1569.8116, 2607.2344

13. D5U7R2 **Mass:** 30130 **Score:** 80 **Expect:** 0.00041 **Queries matched:** 1
D5U7R2_BRAM5 Flagellin OS=Brachyspira murdochii (strain ATCC 51284 / DSM 12563 / 56-150) GN=Bmur_2791 PE=3 SV=1

Observed	Mr(expt)	Mr(calc)	ppm	Start	End	Miss	Ions	Peptide
1023.4379	1022.4306	1022.4781	-46.44	196 -	204	0	68	R.SDLGAYQNR.F

No match to: 1054.4423, 1557.7401, 1569.8116, 2607.2344

14. J9UYC2 **Mass:** 31304 **Score:** 60 **Expect:** 0.046 **Queries matched:** 2
J9UYC2_BRAPL Flagellin OS=Brachyspira pilosicoli B2904 GN=B2904_orf2581 PE=3 SV=1

Observed	Mr(expt)	Mr(calc)	ppm	Start	End	Miss	Ions	Peptide
1054.4423	1053.4350	1053.4583	-22.09	26 -	35	0	14	K.DMASLASGMR.I + Oxidation (M)
1557.7401	1556.7328	1556.8093	-49.10	1 -	14	0	18	-.MIINNNISAVNAQR.T

No match to: 1023.4379, 1569.8116, 2607.2344

15. K0JHQ4 **Mass:** 31288 **Score:** 60 **Expect:** 0.046 **Queries matched:** 2
K0JHQ4_BRAPL Flagellin OS=Brachyspira pilosicoli WesB GN=flaB2 PE=3 SV=1

Observed	Mr(expt)	Mr(calc)	ppm	Start	End	Miss	Ions	Peptide
1054.4423	1053.4350	1053.4583	-22.09	26 -	35	0	14	K.DMASLASGMR.I + Oxidation (M)
1557.7401	1556.7328	1556.8093	-49.10	1 -	14	0	18	-.MIINNNISAVNAQR.T

No match to: 1023.4379, 1569.8116, 2607.2344

16. R5LEF1 **Mass:** 33306 **Score:** 50 **Expect:** 0.42 **Queries matched:** 3
R5LEF1_9SPIR Peptidase M23 OS=Brachyspira sp. CAG:700 GN=BN758_01473 PE=4 SV=1

Observed	Mr(expt)	Mr(calc)	ppm	Start	End	Miss	Ions	Peptide
1557.7401	1556.7328	1556.7735	-26.15	230 -	241	1	---	K.RDNIYYIAPNYR.F
1569.8116	1568.8043	1568.8311	-17.05	157 -	170	0	---	K.GSEQSPLIHGIYLR.T

```
2607.2344  2606.2271  2606.3235  -36.97   117 - 141   1  ---   R.DIIALTGNSGRSGGAHLHLTVENMK.E + Oxidation (M)
```

No match to: 1023.4379, 1054.4423

Search Parameters

```
Type of search         : Sequence Query
Enzyme                 : Trypsin
Fixed modifications    : Carbamidomethyl (C)
Variable modifications : Oxidation (M)
Mass values            : Monoisotopic
Protein Mass           : Unrestricted
Peptide Mass Tolerance : ± 50 ppm
Fragment Mass Tolerance: ± 0.5 Da
Max Missed Cleavages   : 1
Instrument type        : MALDI-TOF-TOF
Query1 (1023.4379,1+)  : Locus:38.1.1.211185.56701
Query2 (1054.4423,1+)  : Locus:38.1.1.211185.56703
Query3 (1557.7401,1+)  : Locus:38.1.1.211185.56700
Query4 (1569.8116,1+)  : Locus:38.1.1.211185.56704
Query5 (2607.2344,1+)  : Locus:38.1.1.211185.56702
```

Mascot: http://www.matrixscience.com/

Mascot Search Results

```
User          :
Email         :
Search title  : F:\ROD_inmunoproteoma\MALDI\ppw_2014_442_ROD\ppw_G4_139601421203.txt
MS data file  : F:\ROD_inmunoproteoma\MALDI\ppw_2014_442_ROD\ppw_G4_139601421203.txt
Database      : Brachyspiraceae Brachyspiraceae_20160127-151122 (40573 sequences; 13470793 residues)
Timestamp     : 22 Feb 2016 at 15:13:55 GMT
Warning       : A Peptide summary report will usually give a much clearer picture of MS/MS search results.
Top Score     : 246 for D8ICA8, D8ICA8_BRAP9 Putative flagellar filament outer layer protein, FlaA OS=Brachyspira pilosicoli (strain ATCC
```

Probability Based Mowse Score

Protein score is -10*Log(P), where P is the probability that the observed match is a random event.
Protein scores greater than 59 are significant (p<0.05).
Protein scores are derived from ions scores as a non-probabilistic basis for ranking protein hits.

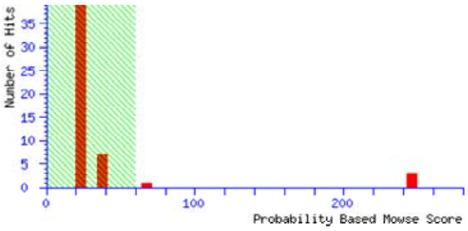

Protein Summary Report

Format As | Protein Summary (deprecated) | Help

Significance threshold p< 0.05 | Max. number of hits AUTO

Standard scoring ◉ MudPIT scoring ○ | Ions score or expect cut-off 0 | Show sub-sets 0

Show pop-ups ◉ Suppress pop-ups ○ | Sort unassigned Decreasing Score | Require bold red ☐

Re-Search All | Search Unmatched

Index

	Accession	Mass	Score	Description
1.	D8ICA8	26766	246	D8ICA8_BRAP9 Putative flagellar filament outer layer protein, FlaA OS=Brachyspira pilosicoli (strain ATCC BAA-18
2.	J9UX66	31445	245	J9UX66_BRAPL Flagellar filament outer layer protein FlaA OS=Brachyspira pilosicoli B2904 GN=B2904_orf2170 PE=4 S
3.	K0JM58	31546	245	K0JM58_BRAPL Flagellar filament outer layer protein FlaA OS=Brachyspira pilosicoli WesB GN=flaA1 PE=4 SV=1
4.	R5LT29	31130	70	R5LT29_9SPIR Flagellar filament outer layer protein FlaA OS=Brachyspira sp. CAG:700 GN=BN758_01591 PE=4 SV=1
5.	L8XPE1	18372	39	L8XPE1_9SPIR PTS system mannitol/fructose-specific transporter subunit IIA domain-containing protein OS=Brachysp

Results List

1. D8ICA8 **Mass:** 26766 **Score:** 246 **Expect:** 1e-20 **Queries matched:** 2
D8ICA8_BRAP9 Putative flagellar filament outer layer protein, FlaA OS=Brachyspira pilosicoli (strain ATCC BAA-1826 / 95/1000) GN=flaA

Observed	Mr(expt)	Mr(calc)	ppm	Start	End	Miss	Ions	Peptide
1021.4727	1020.4654	1020.4665	-1.03	159	166	0	56	R.FWASPEER.A
2144.1052	2143.0979	2143.0950	1.39	130	148	0	155	K.NLSTSVPAFLPQEEPYVPR.A

No match to: 3057.3723

2. J9UX66 **Mass:** 31445 **Score:** 245 **Expect:** 1.3e-20 **Queries matched:** 2
J9UX66_BRAPL Flagellar filament outer layer protein FlaA OS=Brachyspira pilosicoli B2904 GN=B2904_orf2170 PE=4 SV=1

Observed	Mr(expt)	Mr(calc)	ppm	Start	End	Miss	Ions	Peptide
1021.4727	1020.4654	1020.4665	-1.03	199	206	0	56	R.FWASPEER.A
2144.1052	2143.0979	2143.0950	1.39	170	188	0	155	K.NLSTSVPAFLPQEEPYVPR.A

No match to: 3057.3723

3. K0JM58 **Mass:** 31546 **Score:** 245 **Expect:** 1.3e-20 **Queries matched:** 2
K0JM58_BRAPL Flagellar filament outer layer protein FlaA OS=Brachyspira pilosicoli WesB GN=flaA1 PE=4 SV=1

Observed	Mr(expt)	Mr(calc)	ppm	Start	End	Miss	Ions	Peptide
1021.4727	1020.4654	1020.4665	-1.03	199	206	0	56	R.FWASPEER.A
2144.1052	2143.0979	2143.0950	1.39	170	188	0	155	K.NLSTSVPAFLPQEEPYVPR.A

No match to: 3057.3723

4. R5LT29 **Mass:** 31130 **Score:** 70 **Expect:** 0.004 **Queries matched:** 1
R5LT29_9SPIR Flagellar filament outer layer protein FlaA OS=Brachyspira sp. CAG:700 GN=BN758_01591 PE=4 SV=1

Observed	Mr(expt)	Mr(calc)	ppm	Start	End	Miss	Ions	Peptide
1021.4727	1020.4654	1020.4665	-1.03	199	206	0	56	R.FWASPEER.A

No match to: 2144.1052, 3057.3723

5. L8XPE1 **Mass:** 18372 **Score:** 39 **Expect:** 5.6 **Queries matched:** 2
L8XPE1_9SPIR PTS system mannitol/fructose-specific transporter subunit IIA domain-containing protein OS=Brachyspira hampsonii 30599 GN

Observed	Mr(expt)	Mr(calc)	ppm	Start	End	Miss	Ions	Peptide
1021.4727	1020.4654	1020.5161	-49.69	1	9	0	---	-.MISLSDVEK.Y
2144.1052	2143.0979	2143.0945	1.61	130	147	1	---	R.MSMYPVVLLESKTIEEFK.E

No match to: 3057.3723

Search Parameters

```
Type of search         : Sequence Query
Enzyme                 : Trypsin
Fixed modifications    : Carbamidomethyl (C)
Variable modifications : Oxidation (M)
Mass values            : Monoisotopic
Protein Mass           : Unrestricted
Peptide Mass Tolerance : ± 50 ppm
Fragment Mass Tolerance: ± 0.5 Da
Max Missed Cleavages   : 1
Instrument type        : MALDI-TOF-TOF
Query1 (1021.4727,1+)  : Locus:4.1.1.170602.52745
Query2 (2144.1052,1+)  : Locus:4.1.1.170602.52744
Query3 (3057.3723,1+)  : Locus:4.1.1.170602.52746
```

Mascot: http://www.matrixscience.com/

Mascot Search Results

User	:
Email	:
Search title	: F:\ROD_inmunoproteoma\MALDI\ppw_2014_442_ROD\ppw_G9_139601421308.txt
MS data file	: F:\ROD_inmunoproteoma\MALDI\ppw_2014_442_ROD\ppw_G9_139601421308.txt
Database	: Brachyspiraceae Brachyspiraceae_20160127-151122 (40573 sequences; 13470793 residues)
Timestamp	: 22 Feb 2016 at 15:14:08 GMT
Warning	: A Peptide summary report will usually give a much clearer picture of MS/MS search results.
Top Score	: 257 for J9UYC2, J9UYC2_BRAPL Flagellin OS=Brachyspira pilosicoli B2904 GN=B2904_orf2581 PE=3 SV=1

Probability Based Mowse Score

Protein score is -10*Log(P), where P is the probability that the observed match is a random event.
Protein scores greater than 59 are significant (p<0.05).
Protein scores are derived from ions scores as a non-probabilistic basis for ranking protein hits.

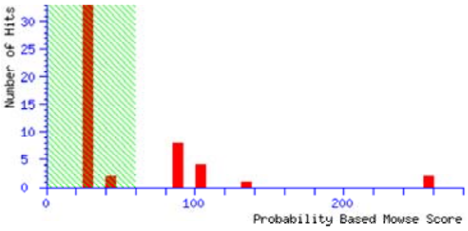

Protein Summary Report

Format As | Protein Summary (deprecated) | Help

Significance threshold p< 0.05 | Max. number of hits AUTO

Standard scoring ◉ MudPIT scoring ○ | Ions score or expect cut-off 0 | Show sub-sets 0

Show pop-ups ◉ Suppress pop-ups ○ | Sort unassigned Decreasing Score | Require bold red ☐

Re-Search All | Search Unmatched

Index

	Accession	Mass	Score	Description
1.	J9UYC2	31304	257	J9UYC2_BRAPL Flagellin OS=Brachyspira pilosicoli B2904 GN=B2904_orf2581 PE=3 SV=1
2.	K0JHQ4	31288	257	K0JHQ4_BRAPL Flagellin OS=Brachyspira pilosicoli WesB GN=flaB2 PE=3 SV=1
3.	Q5XPI2	31258	139	Q5XPI2_BRAPL Flagellin (Fragment) OS=Brachyspira pilosicoli PE=3 SV=1
4.	P80160	31113	105	FLAB2_BRAHO Flagellar filament core protein flaB2 OS=Brachyspira hyodysenteriae GN=flaB2 PE=1 SV=1
5.	A0A0H0X544	31129	105	A0A0H0X544_BRAHO Flagellin OS=Brachyspira hyodysenteriae GN=SU43_01870 PE=3 SV=1
6.	A0A0G4K9G4	31184	105	A0A0G4K9G4_9SPIR Flagellin OS=Brachyspira suanatina GN=flaB2 PE=3 SV=1
7.	A0A0H0ULZ7	31057	105	A0A0H0ULZ7_BRAHO Flagellin OS=Brachyspira hyodysenteriae GN=SR30_04500 PE=3 SV=1
8.	A0A0H0USH8	30059	95	A0A0H0USH8_BRAHO Flagellin OS=Brachyspira hyodysenteriae GN=SR30_01525 PE=3 SV=1
9.	A0A0G4K947	30002	95	A0A0G4K947_9SPIR Flagellin OS=Brachyspira suanatina GN=flaB2 PE=3 SV=1
10.	G0EIL9	30029	95	G0EIL9_BRAIP Flagellin OS=Brachyspira intermedia (strain ATCC 51140 / PWS/A) GN=flaB3 PE=3 SV=1
11.	D5U8W2	29993	95	D5U8W2_BRAM5 Flagellin OS=Brachyspira murdochii (strain ATCC 51284 / DSM 12563 / 56-150) GN=Bmur_1039 PE=3 SV=
12.	L0X7D1	30068	95	L0X7D1_9SPIR Flagellin OS=Brachyspira hampsonii 30446 GN=A966_03171 PE=3 SV=1
13.	L8XR86	30027	95	L8XR86_9SPIR Flagellin OS=Brachyspira hampsonii 30599 GN=H263_04258 PE=3 SV=1
14.	Q9F0F6	30445	95	Q9F0F6_BRAHO Flagellin OS=Brachyspira hyodysenteriae GN=flaB3 PE=3 SV=1
15.	P80161	2744	84	FLAB3_BRAHO Flagellar filament core protein flaB3 (Fragment) OS=Brachyspira hyodysenteriae GN=flaB3 PE=1 SV=1
16.	L0WZH8	60860	48	L0WZH8_9SPIR Propanediol dehydratase large subunit OS=Brachyspira hampsonii 30446 GN=pduC PE=4 SV=1

Results List

1. J9UYC2 **Mass:** 31304 **Score:** 257 **Expect:** 8.1e-22 **Queries matched:** 2
J9UYC2_BRAPL Flagellin OS=Brachyspira pilosicoli B2904 GN=B2904_orf2581 PE=3 SV=1

Observed	Mr(expt)	Mr(calc)	ppm	Start	End	Miss	Ions	Peptide
1557.8451	1556.8378	1556.8093	18.3	1 -	14	0	98	-.MIINNNISAVNAQR.T
1694.8512	1693.8439	1693.8127	18.4	20 -	35	1	124	R.NVDLSKDMASLASGMR.I

No match to: 998.5345

2. K0JHQ4 **Mass:** 31288 **Score:** 257 **Expect:** 8.1e-22 **Queries matched:** 2
K0JHQ4_BRAPL Flagellin OS=Brachyspira pilosicoli WesB GN=flaB2 PE=3 SV=1

Observed	Mr(expt)	Mr(calc)	ppm	Start	End	Miss	Ions	Peptide
1557.8451	1556.8378	1556.8093	18.3	1 -	14	0	98	-.MIINNNISAVNAQR.T
1694.8512	1693.8439	1693.8127	18.4	20 -	35	1	124	R.NVDLSKDMASLASGMR.I

No match to: 998.5345

3. Q5XPI2 **Mass:** 31258 **Score:** 139 **Expect:** 5.1e-10 **Queries matched:** 1
Q5XPI2_BRAPL Flagellin (Fragment) OS=Brachyspira pilosicoli PE=3 SV=1

Observed	Mr(expt)	Mr(calc)	ppm	Start	End	Miss	Ions	Peptide
1694.8512	1693.8439	1693.8127	18.4	20 -	35	1	124	R.NVDLSKDMASLASGMR.I

No match to: 998.5345, 1557.8451

4. P80160 **Mass:** 31113 **Score:** 105 **Expect:** 1.3e-06 **Queries matched:** 1
FLAB2_BRAHO Flagellar filament core protein flaB2 OS=Brachyspira hyodysenteriae GN=flaB2 PE=1 SV=1

Observed	Mr(expt)	Mr(calc)	ppm	Start	End	Miss	Ions	Peptide
1694.8512	1693.8439	1693.8127	18.4	20 -	35	1	90	R.NVDLSKDMAALSSGMR.I

No match to: 998.5345, 1557.8451

5. A0A0H0X544 **Mass:** 31129 **Score:** 105 **Expect:** 1.3e-06 **Queries matched:** 1
A0A0H0X544_BRAHO Flagellin OS=Brachyspira hyodysenteriae GN=SU43_01870 PE=3 SV=1

Observed	Mr(expt)	Mr(calc)	ppm	Start		End	Miss	Ions	Peptide
1694.8512	1693.8439	1693.8127	18.4	20	-	35	1	90	R.NVDLSKDMAALSSGMR.I

No match to: 998.5345, 1557.8451

6. A0A0G4K9G4 **Mass:** 31184 **Score:** 105 **Expect:** 1.3e-06 **Queries matched:** 1
A0A0G4K9G4_9SPIR Flagellin OS=Brachyspira suanatina GN=flaB2 PE=3 SV=1

Observed	Mr(expt)	Mr(calc)	ppm	Start		End	Miss	Ions	Peptide
1694.8512	1693.8439	1693.8127	18.4	20	-	35	1	90	R.NVDLSKDMAALSSGMR.I

No match to: 998.5345, 1557.8451

7. A0A0H0ULZ7 **Mass:** 31057 **Score:** 105 **Expect:** 1.3e-06 **Queries matched:** 1
A0A0H0ULZ7_BRAHO Flagellin OS=Brachyspira hyodysenteriae GN=SR30_04500 PE=3 SV=1

Observed	Mr(expt)	Mr(calc)	ppm	Start		End	Miss	Ions	Peptide
1694.8512	1693.8439	1693.8127	18.4	20	-	35	1	90	R.NVDLSKDMAALSSGMR.I

No match to: 998.5345, 1557.8451

8. A0A0H0USH8 **Mass:** 30059 **Score:** 95 **Expect:** 1.2e-05 **Queries matched:** 2
A0A0H0USH8_BRAHO Flagellin OS=Brachyspira hyodysenteriae GN=SR30_01525 PE=3 SV=1

Observed	Mr(expt)	Mr(calc)	ppm	Start		End	Miss	Ions	Peptide
1557.8451	1556.8378	1556.8093	18.4	1	-	14	0	60	-.MIINNNISALNANR.Q
1694.8512	1693.8439	1693.8127	18.4	207	-	221	1	2	R.AELGAVQNRMESMVK.S + 2 Oxidation (M)

No match to: 998.5345

9. A0A0G4K947 **Mass:** 30002 **Score:** 95 **Expect:** 1.2e-05 **Queries matched:** 2
A0A0G4K947_9SPIR Flagellin OS=Brachyspira suanatina GN=flaB2 PE=3 SV=1

Observed	Mr(expt)	Mr(calc)	ppm	Start		End	Miss	Ions	Peptide
1557.8451	1556.8378	1556.8093	18.4	1	-	14	0	60	-.MIINNNISALNANR.Q
1694.8512	1693.8439	1693.8127	18.4	207	-	221	1	2	R.AELGAVQNRMESMVK.S + 2 Oxidation (M)

No match to: 998.5345

10. G0EIL9 **Mass:** 30029 **Score:** 95 **Expect:** 1.2e-05 **Queries matched:** 2
G0EIL9_BRAIP Flagellin OS=Brachyspira intermedia (strain ATCC 51140 / PWS/A) GN=flaB3 PE=3 SV=1

Observed	Mr(expt)	Mr(calc)	ppm	Start		End	Miss	Ions	Peptide
1557.8451	1556.8378	1556.8093	18.4	1	-	14	0	60	-.MIINNNISALNANR.Q
1694.8512	1693.8439	1693.8127	18.4	207	-	221	1	2	R.AELGAVQNRMESMVK.S + 2 Oxidation (M)

No match to: 998.5345

11. D5U8W2 **Mass:** 29993 **Score:** 95 **Expect:** 1.2e-05 **Queries matched:** 2
D5U8W2_BRAM5 Flagellin OS=Brachyspira murdochii (strain ATCC 51284 / DSM 12563 / 56-150) GN=Bmur_1039 PE=3 SV=1

Observed	Mr(expt)	Mr(calc)	ppm	Start		End	Miss	Ions	Peptide
1557.8451	1556.8378	1556.8093	18.4	1	-	14	0	60	-.MIINNNISALNANR.Q
1694.8512	1693.8439	1693.8127	18.4	207	-	221	1	2	R.AELGAVQNRMESMVK.S + 2 Oxidation (M)

No match to: 998.5345

12. L0X7D1 **Mass:** 30068 **Score:** 95 **Expect:** 1.2e-05 **Queries matched:** 2
L0X7D1_9SPIR Flagellin OS=Brachyspira hampsonii 30446 GN=A966_03171 PE=3 SV=1

Observed	Mr(expt)	Mr(calc)	ppm	Start		End	Miss	Ions	Peptide
1557.8451	1556.8378	1556.8093	18.4	1	-	14	0	60	-.MIINNNISALNANR.Q
1694.8512	1693.8439	1693.8127	18.4	207	-	221	1	2	R.AELGAVQNRMESMVK.S + 2 Oxidation (M)

No match to: 998.5345

13. L8XR86 **Mass:** 30027 **Score:** 95 **Expect:** 1.2e-05 **Queries matched:** 2
L8XR86_9SPIR Flagellin OS=Brachyspira hampsonii 30599 GN=H263_04258 PE=3 SV=1

Observed	Mr(expt)	Mr(calc)	ppm	Start		End	Miss	Ions	Peptide
1557.8451	1556.8378	1556.8093	18.4	1	-	14	0	60	-.MIINNNISALNANR.Q
1694.8512	1693.8439	1693.8127	18.4	207	-	221	1	2	R.AELGAVQNRMESMVK.S + 2 Oxidation (M)

No match to: 998.5345

14. Q9F0F6 **Mass:** 30445 **Score:** 95 **Expect:** 1.2e-05 **Queries matched:** 2
Q9F0F6_BRAHO Flagellin OS=Brachyspira hyodysenteriae GN=flaB3 PE=3 SV=1

Observed	Mr(expt)	Mr(calc)	ppm	Start		End	Miss	Ions	Peptide
1557.8451	1556.8378	1556.8093	18.4	1	-	14	0	60	-.MIINNNISALNANR.Q
1694.8512	1693.8439	1693.8127	18.4	207	-	221	1	2	R.AELGAVQNRMESMVK.S + 2 Oxidation (M)

No match to: 998.5345

15. P80161 **Mass:** 2744 **Score:** 84 **Expect:** 0.00016 **Queries matched:** 1
FLAB3_BRAHO Flagellar filament core protein flaB3 (Fragment) OS=Brachyspira hyodysenteriae GN=flaB3 PE=1 SV=1

Observed	Mr(expt)	Mr(calc)	ppm	Start		End	Miss	Ions	Peptide
1557.8451	1556.8378	1556.8093	18.4	1	-	14	0	60	-.MIINNNISALNANR.Q

No match to: 998.5345, 1694.8512

16. L0WZH8 **Mass:** 60860 **Score:** 48 **Expect:** 0.59 **Queries matched:** 3
L0WZH8_9SPIR Propanediol dehydratase large subunit OS=Brachyspira hampsonii 30446 GN=pduC PE=4 SV=1

Observed	Mr(expt)	Mr(calc)	ppm	Start		End	Miss	Ions	Peptide
998.5345	997.5272	997.4902	37.1	275	-	282	0	---	K.SMLYLEAR.C + Oxidation (M)
1557.8451	1556.8378	1556.8167	13.6	86	-	98	1	---	K.KIALMLCDPNVQR.S
1694.8512	1693.8439	1693.8709	-15.92	480	-	494	1	---	R.KGFEDVAQSVLNMLK.Q + Oxidation (M)

Search Parameters

Type of search : Sequence Query
Enzyme : Trypsin
Fixed modifications : Carbamidomethyl (C)

```
Variable modifications : Oxidation (M)
Mass values            : Monoisotopic
Protein Mass           : Unrestricted
Peptide Mass Tolerance : ± 50 ppm
Fragment Mass Tolerance: ± 0.5 Da
Max Missed Cleavages   : 1
Instrument type        : MALDI-TOF-TOF
Query1 (998.5345,1+)   : Locus:9.1.1.170607.52761
Query2 (1557.8451,1+)  : Locus:9.1.1.170607.52759
Query3 (1694.8512,1+)  : Locus:9.1.1.170607.52760
```

Mascot: http://www.matrixscience.com/

Mascot Search Results

```
User          :
Email         :
Search title  : F:\ROD_inmunoproteoma\MALDI\ppw_2014_442_ROD\ppw_G16_139601421515.txt
MS data file  : F:\ROD_inmunoproteoma\MALDI\ppw_2014_442_ROD\ppw_G16_139601421515.txt
Database      : Brachyspiraceae Brachyspiraceae_20160127-151122 (40573 sequences; 13470793 residues)
Timestamp     : 22 Feb 2016 at 15:14:26 GMT
Warning       : A Peptide summary report will usually give a much clearer picture of MS/MS search results.
Top Score     : 72 for J9UYC2, J9UYC2_BRAPL Flagellin OS=Brachyspira pilosicoli B2904 GN=B2904_orf2581 PE=3 SV=1
```

Probability Based Mowse Score

Protein score is -10*Log(P), where P is the probability that the observed match is a random event.
Protein scores greater than 59 are significant (p<0.05).
Protein scores are derived from ions scores as a non-probabilistic basis for ranking protein hits.

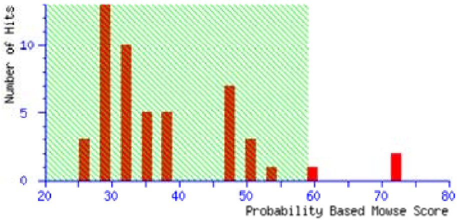

Protein Summary Report

Format As | Protein Summary (deprecated) | Help

Significance threshold p< 0.05 | Max. number of hits AUTO

Standard scoring ◉ MudPIT scoring ○ Ions score or expect cut-off 0 | Show sub-sets 0

Show pop-ups ◉ Suppress pop-ups ○ Sort unassigned Decreasing Score | Require bold red ☐

Re-Search All | Search Unmatched

Index

	Accession	Mass	Score	Description
1.	J9UYC2	31304	72	J9UYC2_BRAPL Flagellin OS=Brachyspira pilosicoli B2904 GN=B2904_orf2581 PE=3 SV=1
2.	K0JHQ4	31288	72	K0JHQ4_BRAPL Flagellin OS=Brachyspira pilosicoli WesB GN=flaB2 PE=3 SV=1
3.	P80161	2744	58	FLAB3_BRAHO Flagellar filament core protein flaB3 (Fragment) OS=Brachyspira hyodysenteriae GN=flaB3 PE=1 SV=1

Results List

```
1.   J9UYC2   Mass: 31304   Score: 72   Expect: 0.0025  Queries matched: 1
     J9UYC2_BRAPL Flagellin OS=Brachyspira pilosicoli B2904 GN=B2904_orf2581 PE=3 SV=1
      Observed   Mr(expt)   Mr(calc)   ppm  Start   End Miss Ions  Peptide
     1557.8585  1556.8512  1556.8093  27.0     1 -   14    0   58   -.MIINNNISAVNAQR.T
     No match to: 713.4074, 1127.5787

2.   K0JHQ4   Mass: 31288   Score: 72   Expect: 0.0025  Queries matched: 1
     K0JHQ4_BRAPL Flagellin OS=Brachyspira pilosicoli WesB GN=flaB2 PE=3 SV=1
      Observed   Mr(expt)   Mr(calc)   ppm  Start   End Miss Ions  Peptide
     1557.8585  1556.8512  1556.8093  27.0     1 -   14    0   58   -.MIINNNISAVNAQR.T
     No match to: 713.4074, 1127.5787

3.   P80161   Mass: 2744    Score: 58   Expect: 0.06  Queries matched: 1
     FLAB3_BRAHO Flagellar filament core protein flaB3 (Fragment) OS=Brachyspira hyodysenteriae GN=flaB3 PE=1 SV=1
      Observed   Mr(expt)   Mr(calc)   ppm  Start   End Miss Ions  Peptide
     1557.8585  1556.8512  1556.8093  27.0     1 -   14    0   34   -.MIINNNISALNANR.Q
     No match to: 713.4074, 1127.5787
```

Search Parameters

```
Type of search         : Sequence Query
Enzyme                 : Trypsin
Fixed modifications    : Carbamidomethyl (C)
Variable modifications : Oxidation (M)
Mass values            : Monoisotopic
Protein Mass           : Unrestricted
Peptide Mass Tolerance : ± 50 ppm
Fragment Mass Tolerance: ± 0.5 Da
Max Missed Cleavages   : 1
Instrument type        : MALDI-TOF-TOF
Query1 (713.4074,1+)   : Locus:16.1.1.170614.52782
Query2 (1127.5787,1+)  : Locus:16.1.1.170614.52781
Query3 (1557.8585,1+)  : Locus:16.1.1.170614.52780
```

Mascot: http://www.matrixscience.com/

Mascot Search Results

User :
Email :
Search title : F:\ROD_inmunoproteoma\MALDI\ppw_2016_562_563_ROD\ppw_I9_145397049925.txt
MS data file : F:\ROD_inmunoproteoma\MALDI\ppw_2016_562_563_ROD\ppw_I9_145397049925.txt
Database : Brachyspiraceae Brachyspiraceae_20160127-151122 (40573 sequences; 13470793 residues)
Timestamp : 23 Feb 2016 at 16:06:06 GMT
Warning : A Peptide summary report will usually give a much clearer picture of MS/MS search results.
Top Score : 217 for P80160, FLAB2_BRAHO Flagellar filament core protein flaB2 OS=Brachyspira hyodysenteriae GN=flaB2 PE=1 SV=1

Probability Based Mowse Score

Protein score is -10*Log(P), where P is the probability that the observed match is a random event.
Protein scores greater than 59 are significant (p<0.05).
Protein scores are derived from ions scores as a non-probabilistic basis for ranking protein hits.

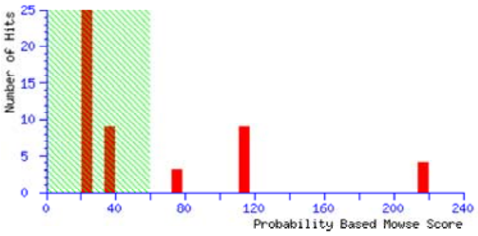

Protein Summary Report

Format As | Protein Summary (deprecated) | Help

Significance threshold p< 0.05 | Max. number of hits AUTO

Standard scoring ◉ MudPIT scoring ○ | Ions score or expect cut-off 0 | Show sub-sets 0

Show pop-ups ◉ Suppress pop-ups ○ | Sort unassigned Decreasing Score | Require bold red ☐

Re-Search All | Search Unmatched

Index

	Accession	Mass	Score	Description
1.	P80160	31113	217	FLAB2_BRAHO Flagellar filament core protein flaB2 OS=Brachyspira hyodysenteriae GN=flaB2 PE=1 SV=1
2.	A0A0H0X544	31129	217	A0A0H0X544_BRAHO Flagellin OS=Brachyspira hyodysenteriae GN=SU43_01870 PE=3 SV=1
3.	A0A0G4K9G4	31184	217	A0A0G4K9G4_9SPIR Flagellin OS=Brachyspira suanatina GN=flaB2 PE=3 SV=1
4.	A0A0H0ULZ7	31057	217	A0A0H0ULZ7_BRAHO Flagellin OS=Brachyspira hyodysenteriae GN=SR30_04500 PE=3 SV=1
5.	Q7M132	2814	119	Q7M132_BRAHO Flagellar core protein, 34K (Fragment) OS=Brachyspira hyodysenteriae PE=1 SV=1
6.	G0EMH3	27613	116	G0EMH3_BRAIP Putative flagellar filament outer layer protein FlaA OS=Brachyspira intermedia (strain ATCC 51140
7.	C0QWY8	27609	116	C0QWY8_BRAHW Putative flagellar filament outer layer protein FlaA OS=Brachyspira hyodysenteriae (strain ATCC 4
8.	A0A0G4K491	31941	116	A0A0G4K491_9SPIR Flagellar filament protein FlaA OS=Brachyspira suanatina GN=BRSU_0390 PE=4 SV=1
9.	A0A0H0UUF0	31982	116	A0A0H0UUF0_BRAHO Flagellar filament protein FlaA OS=Brachyspira hyodysenteriae GN=SR30_02310 PE=4 SV=1
10.	D5U8S7	32080	116	D5U8S7_BRAM5 Flagellar filament outer layer protein FlaA OS=Brachyspira murdochii (strain ATCC 51284 / DSM 125
11.	D5UB01	31218	109	D5UB01_BRAM5 Flagellin OS=Brachyspira murdochii (strain ATCC 51284 / DSM 12563 / 56-150) GN=Bmur_1792 PE=3 SV=
12.	L0X2I6	31181	109	L0X2I6_9SPIR Flagellin OS=Brachyspira hampsonii 30446 GN=A966_05778 PE=3 SV=1
13.	L8XTT2	31194	109	L8XTT2_9SPIR Flagellin OS=Brachyspira hampsonii 30599 GN=H263_08389 PE=3 SV=1
14.	J9UYC2	31304	74	J9UYC2_BRAPL Flagellin OS=Brachyspira pilosicoli B2904 GN=B2904_orf2581 PE=3 SV=1
15.	K0JHQ4	31288	74	K0JHQ4_BRAPL Flagellin OS=Brachyspira pilosicoli WesB GN=flaB2 PE=3 SV=1
16.	Q5XPI2	31258	74	Q5XPI2_BRAPL Flagellin (Fragment) OS=Brachyspira pilosicoli PE=3 SV=1
17.	R5H4X2	79390	38	R5H4X2_9SPIR LL-diaminopimelate aminotransferase OS=Brachyspira sp. CAG:484 GN=BN676_01353 PE=4 SV=1

Results List

1. P80160 **Mass:** 31113 **Score:** 217 **Expect:** 8.1e-18 **Queries matched:** 2
FLAB2_BRAHO Flagellar filament core protein flaB2 OS=Brachyspira hyodysenteriae GN=flaB2 PE=1 SV=1

Observed	Mr(expt)	Mr(calc)	ppm	Start	End	Miss	Ions	Peptide
1571.7750	1570.7677	1570.8249	-36.40	1 -	14	0	97	-.MIINNNISAINAQR.T
1694.7552	1693.7479	1693.8127	-38.25	20 -	35	1	91	R.NVDLSKDMAALSSGMR.I

No match to: 1184.4883, 2161.9756, 2175.9890

2. A0A0H0X544 **Mass:** 31129 **Score:** 217 **Expect:** 8.1e-18 **Queries matched:** 2
A0A0H0X544_BRAHO Flagellin OS=Brachyspira hyodysenteriae GN=SU43_01870 PE=3 SV=1

Observed	Mr(expt)	Mr(calc)	ppm	Start	End	Miss	Ions	Peptide
1571.7750	1570.7677	1570.8249	-36.40	1 -	14	0	97	-.MIINNNISAINAQR.T
1694.7552	1693.7479	1693.8127	-38.25	20 -	35	1	91	R.NVDLSKDMAALSSGMR.I

No match to: 1184.4883, 2161.9756, 2175.9890

3. A0A0G4K9G4 **Mass:** 31184 **Score:** 217 **Expect:** 8.1e-18 **Queries matched:** 2
A0A0G4K9G4_9SPIR Flagellin OS=Brachyspira suanatina GN=flaB2 PE=3 SV=1

Observed	Mr(expt)	Mr(calc)	ppm	Start	End	Miss	Ions	Peptide
1571.7750	1570.7677	1570.8249	-36.40	1 -	14	0	97	-.MIINNNISAINAQR.T
1694.7552	1693.7479	1693.8127	-38.25	20 -	35	1	91	R.NVDLSKDMAALSSGMR.I

No match to: 1184.4883, 2161.9756, 2175.9890

4. A0A0H0ULZ7 **Mass:** 31057 **Score:** 217 **Expect:** 8.1e-18 **Queries matched:** 2
A0A0H0ULZ7_BRAHO Flagellin OS=Brachyspira hyodysenteriae GN=SR30_04500 PE=3 SV=1

Observed	Mr(expt)	Mr(calc)	ppm	Start	End	Miss	Ions	Peptide
1571.7750	1570.7677	1570.8249	-36.40	1 -	14	0	97	-.MIINNNISAINAQR.T
1694.7552	1693.7479	1693.8127	-38.25	20 -	35	1	91	R.NVDLSKDMAALSSGMR.I

No match to: 1184.4883, 2161.9756, 2175.9890

5. Q7M132 **Mass:** 2814 **Score:** 119 **Expect:** 5.1e-08 **Queries matched:** 1
Q7M132_BRAHO Flagellar core protein, 34K (Fragment) OS=Brachyspira hyodysenteriae PE=1 SV=1

Observed	Mr(expt)	Mr(calc)	ppm	Start	End	Miss	Ions	Peptide
1571.7750	1570.7677	1570.8249	-36.40	1 -	14	0	97	-.MIINNNISAINAQR.T

No match to: 1184.4883, 1694.7552, 2161.9756, 2175.9890

6. G0EMH3 **Mass:** 27613 **Score:** 116 **Expect:** 1e-07 **Queries matched:** 1
G0EMH3_BRAIP Putative flagellar filament outer layer protein FlaA OS=Brachyspira intermedia (strain ATCC 51140 / PWS/A) GN=flaA PE=4 S

Observed	Mr(expt)	Mr(calc)	ppm	Start	End	Miss	Ions	Peptide
2161.9756	2160.9683	2161.0514	-38.43	134 -	152	0	102	R.NMSTAVPSFIPQEEPYVPR.A

No match to: 1184.4883, 1571.7750, 1694.7552, 2175.9890

7. C0QWY8 **Mass:** 27609 **Score:** 116 **Expect:** 1e-07 **Queries matched:** 1
C0QWY8_BRAHW Putative flagellar filament outer layer protein FlaA OS=Brachyspira hyodysenteriae (strain ATCC 49526 / WA1) GN=flaA PE=4

Observed	Mr(expt)	Mr(calc)	ppm	Start	End	Miss	Ions	Peptide
2161.9756	2160.9683	2161.0514	-38.43	134 -	152	0	102	R.NMSTAVPSFIPQEEPYVPR.A

No match to: 1184.4883, 1571.7750, 1694.7552, 2175.9890

8. A0A0G4K491 **Mass:** 31941 **Score:** 116 **Expect:** 1e-07 **Queries matched:** 1
A0A0G4K491_9SPIR Flagellar filament protein FlaA OS=Brachyspira suanatina GN=BRSU_0390 PE=4 SV=1

Observed	Mr(expt)	Mr(calc)	ppm	Start	End	Miss	Ions	Peptide
2161.9756	2160.9683	2161.0514	-38.43	170 -	188	0	102	R.NMSTAVPSFIPQEEPYVPR.A

No match to: 1184.4883, 1571.7750, 1694.7552, 2175.9890

9. A0A0H0UUF0 **Mass:** 31982 **Score:** 116 **Expect:** 1e-07 **Queries matched:** 1
A0A0H0UUF0_BRAHO Flagellar filament protein FlaA OS=Brachyspira hyodysenteriae GN=SR30_02310 PE=4 SV=1

Observed	Mr(expt)	Mr(calc)	ppm	Start	End	Miss	Ions	Peptide
2161.9756	2160.9683	2161.0514	-38.43	170 -	188	0	102	R.NMSTAVPSFIPQEEPYVPR.A

No match to: 1184.4883, 1571.7750, 1694.7552, 2175.9890

10. D5U8S7 **Mass:** 32080 **Score:** 116 **Expect:** 1e-07 **Queries matched:** 1
D5U8S7_BRAM5 Flagellar filament outer layer protein FlaA OS=Brachyspira murdochii (strain ATCC 51284 / DSM 12563 / 56-150) GN=Bmur_10(

Observed	Mr(expt)	Mr(calc)	ppm	Start	End	Miss	Ions	Peptide
2161.9756	2160.9683	2161.0514	-38.43	170 -	188	0	102	R.NMSTAVPSFIPQEEPYVPR.A

No match to: 1184.4883, 1571.7750, 1694.7552, 2175.9890

11. D5UB01 **Mass:** 31218 **Score:** 109 **Expect:** 5.1e-07 **Queries matched:** 1
D5UB01_BRAM5 Flagellin OS=Brachyspira murdochii (strain ATCC 51284 / DSM 12563 / 56-150) GN=Bmur_1792 PE=3 SV=1

Observed	Mr(expt)	Mr(calc)	ppm	Start	End	Miss	Ions	Peptide
1571.7750	1570.7677	1570.8249	-36.40	1 -	14	0	97	-.MIINNNISAINAQR.T

No match to: 1184.4883, 1694.7552, 2161.9756, 2175.9890

12. L0X2I6 **Mass:** 31181 **Score:** 109 **Expect:** 5.1e-07 **Queries matched:** 1
L0X2I6_9SPIR Flagellin OS=Brachyspira hampsonii 30446 GN=A966_05778 PE=3 SV=1

Observed	Mr(expt)	Mr(calc)	ppm	Start	End	Miss	Ions	Peptide
1571.7750	1570.7677	1570.8249	-36.40	1 -	14	0	97	-.MIINNNISAINAQR.T

No match to: 1184.4883, 1694.7552, 2161.9756, 2175.9890

13. L8XTT2 **Mass:** 31194 **Score:** 109 **Expect:** 5.1e-07 **Queries matched:** 1
L8XTT2_9SPIR Flagellin OS=Brachyspira hampsonii 30599 GN=H263_08389 PE=3 SV=1

Observed	Mr(expt)	Mr(calc)	ppm	Start	End	Miss	Ions	Peptide
1571.7750	1570.7677	1570.8249	-36.40	1 -	14	0	97	-.MIINNNISAINAQR.T

No match to: 1184.4883, 1694.7552, 2161.9756, 2175.9890

14. J9UYC2 **Mass:** 31304 **Score:** 74 **Expect:** 0.0016 **Queries matched:** 1
J9UYC2_BRAPL Flagellin OS=Brachyspira pilosicoli B2904 GN=B2904_orf2581 PE=3 SV=1

Observed	Mr(expt)	Mr(calc)	ppm	Start	End	Miss	Ions	Peptide
1694.7552	1693.7479	1693.8127	-38.25	20 -	35	1	61	R.NVDLSKDMASLASGMR.I

No match to: 1184.4883, 1571.7750, 2161.9756, 2175.9890

15. K0JHQ4 **Mass:** 31288 **Score:** 74 **Expect:** 0.0016 **Queries matched:** 1
K0JHQ4_BRAPL Flagellin OS=Brachyspira pilosicoli WesB GN=flaB2 PE=3 SV=1

Observed	Mr(expt)	Mr(calc)	ppm	Start	End	Miss	Ions	Peptide
1694.7552	1693.7479	1693.8127	-38.25	20 -	35	1	61	R.NVDLSKDMASLASGMR.I

No match to: 1184.4883, 1571.7750, 2161.9756, 2175.9890

16. Q5XPI2 **Mass:** 31258 **Score:** 74 **Expect:** 0.0016 **Queries matched:** 1
Q5XPI2_BRAPL Flagellin (Fragment) OS=Brachyspira pilosicoli PE=3 SV=1

Observed	Mr(expt)	Mr(calc)	ppm	Start	End	Miss	Ions	Peptide
1694.7552	1693.7479	1693.8127	-38.25	20 -	35	1	61	R.NVDLSKDMASLASGMR.I

No match to: 1184.4883, 1571.7750, 2161.9756, 2175.9890

17. R5H4X2 **Mass:** 79390 **Score:** 38 **Expect:** 7.2 **Queries matched:** 3
R5H4X2_9SPIR LL-diaminopimelate aminotransferase OS=Brachyspira sp. CAG:484 GN=BN676_01353 PE=4 SV=1

Observed	Mr(expt)	Mr(calc)	ppm	Start	End	Miss	Ions	Peptide
1571.7750	1570.7677	1570.7449	14.5	472 -	484	0	---	R.DFAEYMQTLALNR.K
1694.7552	1693.7479	1693.7269	12.4	125 -	139	0	---	K.GEGAFFYAAADWMMK.R
2175.9890	2174.9817	2175.0379	-25.81	249 -	267	0	---	K.HNILIIHDMDNSEVTHSGK.K + Oxidation (M)

No match to: 1184.4883, 2161.9756

Search Parameters

```
Type of search         : Sequence Query
Enzyme                 : Trypsin
Fixed modifications    : Carbamidomethyl (C)
Variable modifications : Oxidation (M)
Mass values            : Monoisotopic
Protein Mass           : Unrestricted
Peptide Mass Tolerance : ± 50 ppm
Fragment Mass Tolerance: ± 0.5 Da
Max Missed Cleavages   : 1
Instrument type        : MALDI-TOF-TOF
Query1 (1184.4883,1+)  : Locus:26.1.1.211173.56644
Query2 (1571.7750,1+)  : Locus:26.1.1.211173.56640
Query3 (1694.7552,1+)  : Locus:26.1.1.211173.56643
Query4 (2161.9756,1+)  : Locus:26.1.1.211173.56641
Query5 (2175.9890,1+)  : Locus:26.1.1.211173.56642
```

Mascot: http://www.matrixscience.com/

(MATRIX SCIENCE) Mascot Search Results

```
User          :
Email         :
Search title  : F:\ROD_inmunoproteoma\MALDI\ppw_2016_562_563_ROD\ppw_I14_145397050330.txt
MS data file  : F:\ROD_inmunoproteoma\MALDI\ppw_2016_562_563_ROD\ppw_I14_145397050330.txt
Database      : Brachyspiraceae Brachyspiraceae_20160127-151122 (40573 sequences; 13470793 residues)
Timestamp     : 23 Feb 2016 at 16:06:19 GMT
Warning       : A Peptide summary report will usually give a much clearer picture of MS/MS search results.
Top Score     : 480 for P80160, FLAB2_BRAHO Flagellar filament core protein flaB2 OS=Brachyspira hyodysenteriae GN=flaB2 PE=1 SV=1
```

Probability Based Mowse Score

Protein score is -10*Log(P), where P is the probability that the observed match is a random event.
Protein scores greater than 59 are significant (p<0.05).
Protein scores are derived from ions scores as a non-probabilistic basis for ranking protein hits.

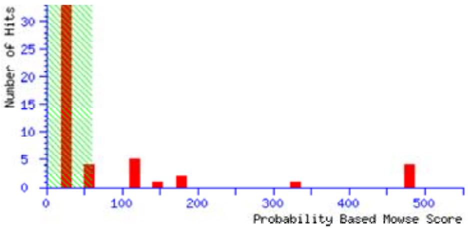

Protein Summary Report

Format As | Protein Summary (deprecated) | Help

Significance threshold p< 0.05 | Max. number of hits AUTO

Standard scoring ◉ MudPIT scoring ○ | Ions score or expect cut-off 0 | Show sub-sets 0

Show pop-ups ◉ Suppress pop-ups ○ | Sort unassigned Decreasing Score | Require bold red ☐

Re-Search All | Search Unmatched

Index

	Accession	Mass	Score	Description
1.	P80160	31113	480	FLAB2_BRAHO Flagellar filament core protein flaB2 OS=Brachyspira hyodysenteriae GN=flaB2 PE=1 SV=1
2.	A0A0H0X544	31129	480	A0A0H0X544_BRAHO Flagellin OS=Brachyspira hyodysenteriae GN=SU43_01870 PE=3 SV=1
3.	A0A0G4K9G4	31184	480	A0A0G4K9G4_9SPIR Flagellin OS=Brachyspira suanatina GN=flaB2 PE=3 SV=1
4.	A0A0H0ULZ7	31057	480	A0A0H0ULZ7_BRAHO Flagellin OS=Brachyspira hyodysenteriae GN=SR30_04500 PE=3 SV=1
5.	D5UB01	31218	315	D5UB01_BRAM5 Flagellin OS=Brachyspira murdochii (strain ATCC 51284 / DSM 12563 / 56-150) GN=Bmur_1792 PE=3 SV=
6.	C0R1I3	28159	184	C0R1I3_BRAHW Flagellin OS=Brachyspira hyodysenteriae (strain ATCC 49526 / WA1) GN=flaB PE=3 SV=1
7.	G0ENU8	28168	184	G0ENU8_BRAIP Flagellin OS=Brachyspira intermedia (strain ATCC 51140 / PWS/A) GN=flaB PE=3 SV=1
8.	Q7M132	2814	137	Q7M132_BRAHO Flagellar core protein, 34K (Fragment) OS=Brachyspira hyodysenteriae PE=1 SV=1
9.	L0X2I6	31181	127	L0X2I6_9SPIR Flagellin OS=Brachyspira hampsonii 30446 GN=A966_05778 PE=3 SV=1
10.	L8XTT2	31194	127	L8XTT2_9SPIR Flagellin OS=Brachyspira hampsonii 30599 GN=H263_08389 PE=3 SV=1
11.	J9UYC2	31304	112	J9UYC2_BRAPL Flagellin OS=Brachyspira pilosicoli B2904 GN=B2904_orf2581 PE=3 SV=1
12.	K0JHQ4	31288	112	K0JHQ4_BRAPL Flagellin OS=Brachyspira pilosicoli WesB GN=flaB2 PE=3 SV=1
13.	Q5XPI2	31258	112	Q5XPI2_BRAPL Flagellin (Fragment) OS=Brachyspira pilosicoli PE=3 SV=1
14.	A0A0H0WHU9	58190	44	A0A0H0WHU9_BRAHO GMP synthase [glutamine-hydrolyzing] OS=Brachyspira hyodysenteriae GN=guaA PE=3 SV=1

Results List

1. P80160 **Mass:** 31113 **Score:** 480 **Expect:** 4.1e-44 **Queries matched:** 3
FLAB2_BRAHO Flagellar filament core protein flaB2 OS=Brachyspira hyodysenteriae GN=flaB2 PE=1 SV=1

Observed	Mr(expt)	Mr(calc)	ppm	Start		End	Miss	Ions	Peptide
1571.8070	1570.7997	1570.8249	-16.03	1	-	14	0	115	-.MIINNNISAINAQR.T
1694.7684	1693.7611	1693.8127	-30.46	20	-	35	1	145	R.NVDLSKDMAALSSGMR.I
2918.4138	2917.4065	2917.4902	-28.67	256	-	282	1	169	K.FAKDQILSQANLAMLAQANTMNQGALR.L

No match to: 1585.7938, 1997.9269

2. A0A0H0X544 **Mass:** 31129 **Score:** 480 **Expect:** 4.1e-44 **Queries matched:** 3
A0A0H0X544_BRAHO Flagellin OS=Brachyspira hyodysenteriae GN=SU43_01870 PE=3 SV=1

Observed	Mr(expt)	Mr(calc)	ppm	Start		End	Miss	Ions	Peptide
1571.8070	1570.7997	1570.8249	-16.03	1	-	14	0	115	-.MIINNNISAINAQR.T
1694.7684	1693.7611	1693.8127	-30.46	20	-	35	1	145	R.NVDLSKDMAALSSGMR.I
2918.4138	2917.4065	2917.4902	-28.67	256	-	282	1	169	K.FAKDQILSQANLAMLAQANTMNQGALR.L

No match to: 1585.7938, 1997.9269

3. A0A0G4K9G4 **Mass:** 31184 **Score:** 480 **Expect:** 4.1e-44 **Queries matched:** 3
A0A0G4K9G4_9SPIR Flagellin OS=Brachyspira suanatina GN=flaB2 PE=3 SV=1

Observed	Mr(expt)	Mr(calc)	ppm	Start		End	Miss	Ions	Peptide
1571.8070	1570.7997	1570.8249	-16.03	1	-	14	0	115	-.MIINNNISAINAQR.T
1694.7684	1693.7611	1693.8127	-30.46	20	-	35	1	145	R.NVDLSKDMAALSSGMR.I
2918.4138	2917.4065	2917.4902	-28.67	256	-	282	1	169	K.FAKDQILSQANLAMLAQANTMNQGALR.L

No match to: 1585.7938, 1997.9269

4. A0A0H0ULZ7 **Mass:** 31057 **Score:** 480 **Expect:** 4.1e-44 **Queries matched:** 3

A0A0H0ULZ7_BRAHO Flagellin OS=Brachyspira hyodysenteriae GN=SR30_04500 PE=3 SV=1

Observed	Mr(expt)	Mr(calc)	ppm	Start		End	Miss	Ions	Peptide
1571.8070	1570.7997	1570.8249	-16.03	1	-	14	0	115	-.MIINNNISAINAQR.T
1694.7684	1693.7611	1693.8127	-30.46	20	-	35	1	145	R.NVDLSKDMAALSSGMR.I
2918.4138	2917.4065	2917.4902	-28.67	256	-	282	1	169	K.FAKDQILSQANLAMLAQANTMNQGALR.L

No match to: 1585.7938, 1997.9269

5. D5UB01 **Mass:** 31218 **Score:** 315 **Expect:** 1.3e-27 **Queries matched:** 2
D5UB01_BRAM5 Flagellin OS=Brachyspira murdochii (strain ATCC 51284 / DSM 12563 / 56-150) GN=Bmur_1792 PE=3 SV=1

Observed	Mr(expt)	Mr(calc)	ppm	Start		End	Miss	Ions	Peptide
1571.8070	1570.7997	1570.8249	-16.03	1	-	14	0	115	-.MIINNNISAINAQR.T
2918.4138	2917.4065	2917.4902	-28.67	256	-	282	1	169	K.FAKDQILSQANLAMLAQANTMNQGALR.L

No match to: 1585.7938, 1694.7684, 1997.9269

6. C0R1I3 **Mass:** 28159 **Score:** 184 **Expect:** 1.6e-14 **Queries matched:** 1
C0R1I3_BRAHW Flagellin OS=Brachyspira hyodysenteriae (strain ATCC 49526 / WA1) GN=flaB PE=3 SV=1

Observed	Mr(expt)	Mr(calc)	ppm	Start		End	Miss	Ions	Peptide
2918.4138	2917.4065	2917.4902	-28.67	230	-	256	1	169	K.FAKDQILSQANLAMLAQANTMNQGALR.L

No match to: 1571.8070, 1585.7938, 1694.7684, 1997.9269

7. G0ENU8 **Mass:** 28168 **Score:** 184 **Expect:** 1.6e-14 **Queries matched:** 1
G0ENU8_BRAIP Flagellin OS=Brachyspira intermedia (strain ATCC 51140 / PWS/A) GN=flaB PE=3 SV=1

Observed	Mr(expt)	Mr(calc)	ppm	Start		End	Miss	Ions	Peptide
2918.4138	2917.4065	2917.4902	-28.67	230	-	256	1	169	K.FAKDQILSQANLAMLAQANTMNQGALR.L

No match to: 1571.8070, 1585.7938, 1694.7684, 1997.9269

8. Q7M132 **Mass:** 2814 **Score:** 137 **Expect:** 8.1e-10 **Queries matched:** 1
Q7M132_BRAHO Flagellar core protein, 34K (Fragment) OS=Brachyspira hyodysenteriae PE=1 SV=1

Observed	Mr(expt)	Mr(calc)	ppm	Start		End	Miss	Ions	Peptide
1571.8070	1570.7997	1570.8249	-16.03	1	-	14	0	115	-.MIINNNISAINAQR.T

No match to: 1585.7938, 1694.7684, 1997.9269, 2918.4138

9. L0X2I6 **Mass:** 31181 **Score:** 127 **Expect:** 8.1e-09 **Queries matched:** 1
L0X2I6_9SPIR Flagellin OS=Brachyspira hampsonii 30446 GN=A966_05778 PE=3 SV=1

Observed	Mr(expt)	Mr(calc)	ppm	Start		End	Miss	Ions	Peptide
1571.8070	1570.7997	1570.8249	-16.03	1	-	14	0	115	-.MIINNNISAINAQR.T

No match to: 1585.7938, 1694.7684, 1997.9269, 2918.4138

10. L8XTT2 **Mass:** 31194 **Score:** 127 **Expect:** 8.1e-09 **Queries matched:** 1
L8XTT2_9SPIR Flagellin OS=Brachyspira hampsonii 30599 GN=H263_08389 PE=3 SV=1

Observed	Mr(expt)	Mr(calc)	ppm	Start		End	Miss	Ions	Peptide
1571.8070	1570.7997	1570.8249	-16.03	1	-	14	0	115	-.MIINNNISAINAQR.T

No match to: 1585.7938, 1694.7684, 1997.9269, 2918.4138

11. J9UYC2 **Mass:** 31304 **Score:** 112 **Expect:** 2.6e-07 **Queries matched:** 1
J9UYC2_BRAPL Flagellin OS=Brachyspira pilosicoli B2904 GN=B2904_orf2581 PE=3 SV=1

Observed	Mr(expt)	Mr(calc)	ppm	Start		End	Miss	Ions	Peptide
1694.7684	1693.7611	1693.8127	-30.46	20	-	35	1	99	R.NVDLSKDMASLASGMR.I

No match to: 1571.8070, 1585.7938, 1997.9269, 2918.4138

12. K0JHQ4 **Mass:** 31288 **Score:** 112 **Expect:** 2.6e-07 **Queries matched:** 1
K0JHQ4_BRAPL Flagellin OS=Brachyspira pilosicoli WesB GN=flaB2 PE=3 SV=1

Observed	Mr(expt)	Mr(calc)	ppm	Start		End	Miss	Ions	Peptide
1694.7684	1693.7611	1693.8127	-30.46	20	-	35	1	99	R.NVDLSKDMASLASGMR.I

No match to: 1571.8070, 1585.7938, 1997.9269, 2918.4138

13. Q5XPI2 **Mass:** 31258 **Score:** 112 **Expect:** 2.6e-07 **Queries matched:** 1
Q5XPI2_BRAPL Flagellin (Fragment) OS=Brachyspira pilosicoli PE=3 SV=1

Observed	Mr(expt)	Mr(calc)	ppm	Start		End	Miss	Ions	Peptide
1694.7684	1693.7611	1693.8127	-30.46	20	-	35	1	99	R.NVDLSKDMASLASGMR.I

No match to: 1571.8070, 1585.7938, 1997.9269, 2918.4138

14. A0A0H0WHU9 **Mass:** 58190 **Score:** 44 **Expect:** 1.7 **Queries matched:** 3
A0A0H0WHU9_BRAHO GMP synthase [glutamine-hydrolyzing] OS=Brachyspira hyodysenteriae GN=guaA PE=3 SV=1

Observed	Mr(expt)	Mr(calc)	ppm	Start		End	Miss	Ions	Peptide
1571.8070	1570.7997	1570.8454	-29.07	412	-	425	0	---	K.ILQEADDIVVSEIK.K
1997.9269	1996.9196	1996.9459	-13.15	445	-	462	1	---	K.SVGVMGDGRTYEQVCAIR.A
2918.4138	2917.4065	2917.4792	-24.91	72	-	97	0	---	K.QLFELGVPILGICYGMQIIVYSMGGK.V + 2 Oxidation (M)

No match to: 1585.7938, 1694.7684

Search Parameters

Type of search : Sequence Query
Enzyme : Trypsin
Fixed modifications : Carbamidomethyl (C)
Variable modifications : Oxidation (M)
Mass values : Monoisotopic
Protein Mass : Unrestricted
Peptide Mass Tolerance : ± 50 ppm
Fragment Mass Tolerance: ± 0.5 Da
Max Missed Cleavages : 1
Instrument type : MALDI-TOF-TOF
Query1 (1571.8070,1+) : Locus:31.1.1.211178.56665
Query2 (1585.7938,1+) : Locus:31.1.1.211178.56669
Query3 (1694.7684,1+) : Locus:31.1.1.211178.56666
Query4 (1997.9269,1+) : Locus:31.1.1.211178.56668
Query5 (2918.4138,1+) : Locus:31.1.1.211178.56667

Mascot: http://www.matrixscience.com/

Mascot Search Results

User :
Email :
Search title : F:\ROD_inmunoproteoma\MALDI\160223_2016_561_562_ROD\ppw_G6_145631468213.txt
MS data file : F:\ROD_inmunoproteoma\MALDI\160223_2016_561_562_ROD\ppw_G6_145631468213.txt
Database : Brachyspiraceae Brachyspiraceae_20160127-151122 (40573 sequences; 13470793 residues)
Timestamp : 24 Feb 2016 at 12:02:55 GMT
Warning : A Peptide summary report will usually give a much clearer picture of MS/MS search results.
Top Score : 293 for J9UYC2, J9UYC2_BRAPL Flagellin OS=Brachyspira pilosicoli B2904 GN=B2904_orf2581 PE=3 SV=1

Probability Based Mowse Score

Protein score is -10*Log(P), where P is the probability that the observed match is a random event.
Protein scores greater than 59 are significant (p<0.05).
Protein scores are derived from ions scores as a non-probabilistic basis for ranking protein hits.

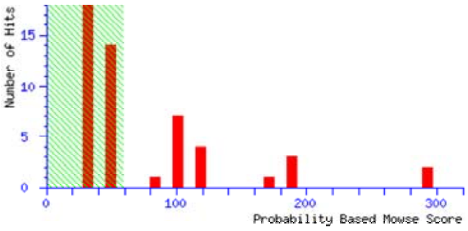

Protein Summary Report

Format As | Protein Summary (deprecated) | Help

Significance threshold p< 0.05 Max. number of hits AUTO

Standard scoring ◉ MudPIT scoring ○ Ions score or expect cut-off 0 Show sub-sets 0

Show pop-ups ◉ Suppress pop-ups ○ Sort unassigned Decreasing Score Require bold red ☐

Re-Search All | Search Unmatched

Index

	Accession	Mass	Score	Description
1.	J9UYC2	31304	293	J9UYC2_BRAPL Flagellin OS=Brachyspira pilosicoli B2904 GN=B2904_orf2581 PE=3 SV=1
2.	K0JHQ4	31288	293	K0JHQ4_BRAPL Flagellin OS=Brachyspira pilosicoli WesB GN=flaB2 PE=3 SV=1
3.	J9U2W9	34204	191	J9U2W9_BRAPL Uncharacterized protein OS=Brachyspira pilosicoli B2904 GN=B2904_orf2565 PE=4 SV=1
4.	K0JII2	34163	191	K0JII2_BRAPL Uncharacterized protein OS=Brachyspira pilosicoli WesB GN=WESB_2576 PE=4 SV=1
5.	D8IDB9	34810	191	D8IDB9_BRAP9 Uncharacterized protein OS=Brachyspira pilosicoli (strain ATCC BAA-1826 / 95/1000) GN=BP951000_11
6.	Q5XPI2	31258	164	Q5XPI2_BRAPL Flagellin (Fragment) OS=Brachyspira pilosicoli PE=3 SV=1
7.	P80160	31113	127	FLAB2_BRAHO Flagellar filament core protein flaB2 OS=Brachyspira hyodysenteriae GN=flaB2 PE=1 SV=1
8.	A0A0H0X544	31129	127	A0A0H0X544_BRAHO Flagellin OS=Brachyspira hyodysenteriae GN=SU43_01870 PE=3 SV=1
9.	A0A0G4K9G4	31184	127	A0A0G4K9G4_9SPIR Flagellin OS=Brachyspira suanatina GN=flaB2 PE=3 SV=1
10.	A0A0H0ULZ7	31057	127	A0A0H0ULZ7_BRAHO Flagellin OS=Brachyspira hyodysenteriae GN=SR30_04500 PE=3 SV=1
11.	A0A0H0USH8	30059	93	A0A0H0USH8_BRAHO Flagellin OS=Brachyspira hyodysenteriae GN=SR30_01525 PE=3 SV=1
12.	A0A0G4K947	30002	93	A0A0G4K947_9SPIR Flagellin OS=Brachyspira suanatina GN=flaB2 PE=3 SV=1
13.	G0EIL9	30029	93	G0EIL9_BRAIP Flagellin OS=Brachyspira intermedia (strain ATCC 51140 / PWS/A) GN=flaB3 PE=3 SV=1
14.	D5U8W2	29993	93	D5U8W2_BRAM5 Flagellin OS=Brachyspira murdochii (strain ATCC 51284 / DSM 12563 / 56-150) GN=Bmur_1039 PE=3 SV=
15.	L0X7D1	30068	93	L0X7D1_9SPIR Flagellin OS=Brachyspira hampsonii 30446 GN=A966_03171 PE=3 SV=1
16.	L8XR86	30027	93	L8XR86_9SPIR Flagellin OS=Brachyspira hampsonii 30599 GN=H263_04258 PE=3 SV=1
17.	Q9F0F6	30445	93	Q9F0F6_BRAHO Flagellin OS=Brachyspira hyodysenteriae GN=flaB3 PE=3 SV=1
18.	P80161	2744	85	FLAB3_BRAHO Flagellar filament core protein flaB3 (Fragment) OS=Brachyspira hyodysenteriae GN=flaB3 PE=1 SV=1
19.	Q26501	31952	53	Q26501_BRAHO Flagellin OS=Brachyspira hyodysenteriae GN=flaB1 PE=3 SV=1

Results List

1. J9UYC2 **Mass:** 31304 **Score:** 293 **Expect:** 2e-25 **Queries matched:** 2
J9UYC2_BRAPL Flagellin OS=Brachyspira pilosicoli B2904 GN=B2904_orf2581 PE=3 SV=1

Observed	Mr(expt)	Mr(calc)	ppm	Start	End	Miss	Ions	Peptide
1557.8604	1556.8531	1556.8093	28.2	1 -	14	0	113	-.MIINNNISAVNAQR.T
1694.8590	1693.8517	1693.8127	23.0	20 -	35	1	151	R.NVDLSKDMASLASGMR.I

No match to: 998.5469, 1127.5756, 1574.8104

2. K0JHQ4 **Mass:** 31288 **Score:** 293 **Expect:** 2e-25 **Queries matched:** 2
K0JHQ4_BRAPL Flagellin OS=Brachyspira pilosicoli WesB GN=flaB2 PE=3 SV=1

Observed	Mr(expt)	Mr(calc)	ppm	Start	End	Miss	Ions	Peptide
1557.8604	1556.8531	1556.8093	28.2	1 -	14	0	113	-.MIINNNISAVNAQR.T
1694.8590	1693.8517	1693.8127	23.0	20 -	35	1	151	R.NVDLSKDMASLASGMR.I

No match to: 998.5469, 1127.5756, 1574.8104

3. J9U2W9 **Mass:** 34204 **Score:** 191 **Expect:** 3.2e-15 **Queries matched:** 3
J9U2W9_BRAPL Uncharacterized protein OS=Brachyspira pilosicoli B2904 GN=B2904_orf2565 PE=4 SV=1

Observed	Mr(expt)	Mr(calc)	ppm	Start	End	Miss	Ions	Peptide
998.5469	997.5396	997.5167	22.9	249 -	255	0	19	R.MRPIYYR.F
1127.5756	1126.5683	1126.5407	24.5	194 -	203	0	86	K.YGGFDNSLVR.T
1574.8104	1573.8031	1573.7525	32.2	236 -	248	0	40	K.NINFVASYVDNYR.M

No match to: 1557.8604, 1694.8590

4. K0JII2 **Mass:** 34163 **Score:** 191 **Expect:** 3.2e-15 **Queries matched:** 3
K0JII2_BRAPL Uncharacterized protein OS=Brachyspira pilosicoli WesB GN=WESB_2576 PE=4 SV=1

Observed	Mr(expt)	Mr(calc)	ppm	Start	End	Miss	Ions	Peptide
998.5469	997.5396	997.5167	22.9	249 -	255	0	19	R.MRPIYYR.F
1127.5756	1126.5683	1126.5407	24.5	194 -	203	0	86	K.YGGFDNSLVR.T
1574.8104	1573.8031	1573.7525	32.2	236 -	248	0	40	K.NINFVASYVDNYR.M

No match to: 1557.8604, 1694.8590

5. D8IDB9 **Mass:** 34810 **Score:** 191 **Expect:** 3.2e-15 **Queries matched:** 3
D8IDB9_BRAP9 Uncharacterized protein OS=Brachyspira pilosicoli (strain ATCC BAA-1826 / 95/1000) GN=BP951000_1152 PE=4 SV=1

Observed	Mr(expt)	Mr(calc)	ppm	Start	End	Miss	Ions	Peptide
998.5469	997.5396	997.5167	22.9	254 -	260	0	19	R.MRPIYYR.F
1127.5756	1126.5683	1126.5407	24.5	199 -	208	0	86	K.YGGFDNSLVR.T
1574.8104	1573.8031	1573.7525	32.2	241 -	253	0	40	K.NINFVASYVDNYR.M

No match to: 1557.8604, 1694.8590

6. Q5XPI2 **Mass:** 31258 **Score:** 164 **Expect:** 1.6e-12 **Queries matched:** 1
Q5XPI2_BRAPL Flagellin (Fragment) OS=Brachyspira pilosicoli PE=3 SV=1

Observed	Mr(expt)	Mr(calc)	ppm	Start	End	Miss	Ions	Peptide
1694.8590	1693.8517	1693.8127	23.0	20 -	35	1	151	R.NVDLSKDMASLASGMR.I

No match to: 998.5469, 1127.5756, 1557.8604, 1574.8104

7. P80160 **Mass:** 31113 **Score:** 127 **Expect:** 8.1e-09 **Queries matched:** 1
FLAB2_BRAHO Flagellar filament core protein flaB2 OS=Brachyspira hyodysenteriae GN=flaB2 PE=1 SV=1

Observed	Mr(expt)	Mr(calc)	ppm	Start	End	Miss	Ions	Peptide
1694.8590	1693.8517	1693.8127	23.0	20 -	35	1	114	R.NVDLSKDMAALSSGMR.I

No match to: 998.5469, 1127.5756, 1557.8604, 1574.8104

8. A0A0H0X544 **Mass:** 31129 **Score:** 127 **Expect:** 8.1e-09 **Queries matched:** 1
A0A0H0X544_BRAHO Flagellin OS=Brachyspira hyodysenteriae GN=SU43_01870 PE=3 SV=1

Observed	Mr(expt)	Mr(calc)	ppm	Start	End	Miss	Ions	Peptide
1694.8590	1693.8517	1693.8127	23.0	20 -	35	1	114	R.NVDLSKDMAALSSGMR.I

No match to: 998.5469, 1127.5756, 1557.8604, 1574.8104

9. A0A0G4K9G4 **Mass:** 31184 **Score:** 127 **Expect:** 8.1e-09 **Queries matched:** 1
A0A0G4K9G4_9SPIR Flagellin OS=Brachyspira suanatina GN=flaB2 PE=3 SV=1

Observed	Mr(expt)	Mr(calc)	ppm	Start	End	Miss	Ions	Peptide
1694.8590	1693.8517	1693.8127	23.0	20 -	35	1	114	R.NVDLSKDMAALSSGMR.I

No match to: 998.5469, 1127.5756, 1557.8604, 1574.8104

10. A0A0H0ULZ7 **Mass:** 31057 **Score:** 127 **Expect:** 8.1e-09 **Queries matched:** 1
A0A0H0ULZ7_BRAHO Flagellin OS=Brachyspira hyodysenteriae GN=SR30_04500 PE=3 SV=1

Observed	Mr(expt)	Mr(calc)	ppm	Start	End	Miss	Ions	Peptide
1694.8590	1693.8517	1693.8127	23.0	20 -	35	1	114	R.NVDLSKDMAALSSGMR.I

No match to: 998.5469, 1127.5756, 1557.8604, 1574.8104

11. A0A0H0USH8 **Mass:** 30059 **Score:** 93 **Expect:** 2e-05 **Queries matched:** 2
A0A0H0USH8_BRAHO Flagellin OS=Brachyspira hyodysenteriae GN=SR30_01525 PE=3 SV=1

Observed	Mr(expt)	Mr(calc)	ppm	Start	End	Miss	Ions	Peptide
1557.8604	1556.8531	1556.8093	28.2	1 -	14	0	63	-.MIINNNISALNANR.Q
1694.8590	1693.8517	1693.8127	23.0	207 -	221	1	1	R.AELGAVQNRMESMVK.S + 2 Oxidation (M)

No match to: 998.5469, 1127.5756, 1574.8104

12. A0A0G4K947 **Mass:** 30002 **Score:** 93 **Expect:** 2e-05 **Queries matched:** 2
A0A0G4K947_9SPIR Flagellin OS=Brachyspira suanatina GN=flaB2 PE=3 SV=1

Observed	Mr(expt)	Mr(calc)	ppm	Start	End	Miss	Ions	Peptide
1557.8604	1556.8531	1556.8093	28.2	1 -	14	0	63	-.MIINNNISALNANR.Q
1694.8590	1693.8517	1693.8127	23.0	207 -	221	1	1	R.AELGAVQNRMESMVK.S + 2 Oxidation (M)

No match to: 998.5469, 1127.5756, 1574.8104

13. G0EIL9 **Mass:** 30029 **Score:** 93 **Expect:** 2e-05 **Queries matched:** 2
G0EIL9_BRAIP Flagellin OS=Brachyspira intermedia (strain ATCC 51140 / PWS/A) GN=flaB3 PE=3 SV=1

Observed	Mr(expt)	Mr(calc)	ppm	Start	End	Miss	Ions	Peptide
1557.8604	1556.8531	1556.8093	28.2	1 -	14	0	63	-.MIINNNISALNANR.Q
1694.8590	1693.8517	1693.8127	23.0	207 -	221	1	1	R.AELGAVQNRMESMVK.S + 2 Oxidation (M)

No match to: 998.5469, 1127.5756, 1574.8104

14. D5U8W2 **Mass:** 29993 **Score:** 93 **Expect:** 2e-05 **Queries matched:** 2
D5U8W2_BRAM5 Flagellin OS=Brachyspira murdochii (strain ATCC 51284 / DSM 12563 / 56-150) GN=Bmur_1039 PE=3 SV=1

Observed	Mr(expt)	Mr(calc)	ppm	Start	End	Miss	Ions	Peptide
1557.8604	1556.8531	1556.8093	28.2	1 -	14	0	63	-.MIINNNISALNANR.Q
1694.8590	1693.8517	1693.8127	23.0	207 -	221	1	1	R.AELGAVQNRMESMVK.S + 2 Oxidation (M)

No match to: 998.5469, 1127.5756, 1574.8104

15. L0X7D1 **Mass:** 30068 **Score:** 93 **Expect:** 2e-05 **Queries matched:** 2
L0X7D1_9SPIR Flagellin OS=Brachyspira hampsonii 30446 GN=A966_03171 PE=3 SV=1

Observed	Mr(expt)	Mr(calc)	ppm	Start	End	Miss	Ions	Peptide
1557.8604	1556.8531	1556.8093	28.2	1 -	14	0	63	-.MIINNNISALNANR.Q
1694.8590	1693.8517	1693.8127	23.0	207 -	221	1	1	R.AELGAVQNRMESMVK.S + 2 Oxidation (M)

No match to: 998.5469, 1127.5756, 1574.8104

16. L8XR86 **Mass:** 30027 **Score:** 93 **Expect:** 2e-05 **Queries matched:** 2
L8XR86_9SPIR Flagellin OS=Brachyspira hampsonii 30599 GN=H263_04258 PE=3 SV=1

Observed	Mr(expt)	Mr(calc)	ppm	Start	End	Miss	Ions	Peptide
1557.8604	1556.8531	1556.8093	28.2	1 -	14	0	63	-.MIINNNISALNANR.Q
1694.8590	1693.8517	1693.8127	23.0	207 -	221	1	1	R.AELGAVQNRMESMVK.S + 2 Oxidation (M)

No match to: 998.5469, 1127.5756, 1574.8104

17. Q9F0F6 **Mass:** 30445 **Score:** 93 **Expect:** 2e-05 **Queries matched:** 2
Q9F0F6_BRAHO Flagellin OS=Brachyspira hyodysenteriae GN=flaB3 PE=3 SV=1

Observed	Mr(expt)	Mr(calc)	ppm	Start	End	Miss	Ions	Peptide
1557.8604	1556.8531	1556.8093	28.2	1 -	14	0	63	-.MIINNNISALNANR.Q
1694.8590	1693.8517	1693.8127	23.0	207 -	221	1	1	R.AELGAVQNRMESMVK.S + 2 Oxidation (M)

No match to: 998.5469, 1127.5756, 1574.8104

18. P80161 **Mass:** 2744 **Score:** 85 **Expect:** 0.00012 **Queries matched:** 1
FLAB3_BRAHO Flagellar filament core protein flaB3 (Fragment) OS=Brachyspira hyodysenteriae GN=flaB3 PE=1 SV=1

Observed	Mr(expt)	Mr(calc)	ppm	Start	End	Miss	Ions	Peptide
1557.8604	1556.8531	1556.8093	28.2	1 -	14	0	63	-.MIINNNISALNANR.Q

No match to: 998.5469, 1127.5756, 1574.8104, 1694.8590

19. Q26501 **Mass:** 31952 **Score:** 53 **Expect:** 0.2 **Queries matched:** 2
Q26501_BRAHO Flagellin OS=Brachyspira hyodysenteriae GN=flaB1 PE=3 SV=1

Observed	Mr(expt)	Mr(calc)	ppm	Start	End	Miss	Ions	Peptide
1557.8604	1556.8531	1556.8093	28.2	1 -	14	0	18	-.MVINNNISAINAQR.T
1574.8104	1573.8031	1573.7584	28.4	36 -	51	0	7	R.INQAGDDASGLAVSEK.M

No match to: 998.5469, 1127.5756, 1694.8590

Search Parameters

Type of search : Sequence Query
Enzyme : Trypsin
Fixed modifications : Carbamidomethyl (C)
Variable modifications : Oxidation (M)
Mass values : Monoisotopic
Protein Mass : Unrestricted
Peptide Mass Tolerance : ± 50 ppm
Fragment Mass Tolerance: ± 0.5 Da
Max Missed Cleavages : 1
Instrument type : MALDI-TOF-TOF
Query1 (998.5469,1+) : Locus:14.1.1.213513.57304
Query2 (1127.5756,1+) : Locus:14.1.1.213513.57303
Query3 (1557.8604,1+) : Locus:14.1.1.213513.57301
Query4 (1574.8104,1+) : Locus:14.1.1.213513.57305
Query5 (1694.8590,1+) : Locus:14.1.1.213513.57302

Mascot: http://www.matrixscience.com/

Mascot Search Results

```
User          :
Email         :
Search title  : F:\ROD_inmunoproteoma\MALDI\ppw_2014_442_ROD\ppw_G8_139601421307.txt
MS data file  : F:\ROD_inmunoproteoma\MALDI\ppw_2014_442_ROD\ppw_G8_139601421307.txt
Database      : Brachyspiraceae Brachyspiraceae_20160127-151122 (40573 sequences; 13470793 residues)
Timestamp     : 22 Feb 2016 at 15:14:06 GMT
Warning       : A Peptide summary report will usually give a much clearer picture of MS/MS search results.
Top Score     : 192 for R5LA18, R5LA18_9SPIR Flagellin OS=Brachyspira sp. CAG:700 GN=BN758_01499 PE=3 SV=1
```

Probability Based Mowse Score

Protein score is -10*Log(P), where P is the probability that the observed match is a random event.
Protein scores greater than 59 are significant (p<0.05).
Protein scores are derived from ions scores as a non-probabilistic basis for ranking protein hits.

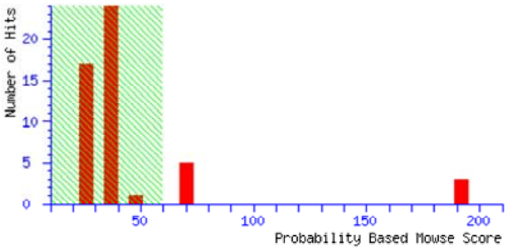

Protein Summary Report

Format As | Protein Summary (deprecated) | Help

Significance threshold p< 0.05 | Max. number of hits AUTO

Standard scoring ◉ MudPIT scoring ○ | Ions score or expect cut-off 0 | Show sub-sets 0

Show pop-ups ◉ Suppress pop-ups ○ | Sort unassigned Decreasing Score | Require bold red ☐

Re-Search All | Search Unmatched

Index

	Accession	Mass	Score	Description
1.	R5LA18	30135	192	R5LA18_9SPIR Flagellin OS=Brachyspira sp. CAG:700 GN=BN758_01499 PE=3 SV=1
2.	J9UXQ6	30134	192	J9UXQ6_BRAPL Flagellin OS=Brachyspira pilosicoli B2904 GN=B2904_orf2664 PE=3 SV=1
3.	K0JLS4	30164	192	K0JLS4_BRAPL Flagellin OS=Brachyspira pilosicoli WesB GN=flaB3 PE=3 SV=1
4.	D8IDG1	26584	75	D8IDG1_BRAP9 Flagellin OS=Brachyspira pilosicoli (strain ATCC BAA-1826 / 95/1000) GN=flaB3 PE=3 SV=1
5.	A0A0G4K947	30002	75	A0A0G4K947_9SPIR Flagellin OS=Brachyspira suanatina GN=flaB2 PE=3 SV=1
6.	G0EIL9	30029	75	G0EIL9_BRAIP Flagellin OS=Brachyspira intermedia (strain ATCC 51140 / PWS/A) GN=flaB3 PE=3 SV=1
7.	L0X7D1	30068	75	L0X7D1_9SPIR Flagellin OS=Brachyspira hampsonii 30446 GN=A966_03171 PE=3 SV=1
8.	L8XR86	30027	75	L8XR86_9SPIR Flagellin OS=Brachyspira hampsonii 30599 GN=H263_04258 PE=3 SV=1
9.	A0A0G4K9F5	11224	42	A0A0G4K9F5_9SPIR Uncharacterized protein OS=Brachyspira suanatina GN=BRSU_2001 PE=4 SV=1

Results List

1. R5LA18 **Mass:** 30135 **Score:** 192 **Expect:** 2.6e-15 **Queries matched:** 2
R5LA18_9SPIR Flagellin OS=Brachyspira sp. CAG:700 GN=BN758_01499 PE=3 SV=1

Observed	Mr(expt)	Mr(calc)	ppm	Start		End	Miss	Ions	Peptide
1358.7311	1357.7238	1357.7024	15.8	195	-	206	0	60	R.IDEGIQMVVAQR.A
1543.8197	1542.8124	1542.7936	12.2	1	-	14	0	98	-.MIINNNVSALNANR.Q

No match to: 713.4055

2. J9UXQ6 **Mass:** 30134 **Score:** 192 **Expect:** 2.6e-15 **Queries matched:** 2
J9UXQ6_BRAPL Flagellin OS=Brachyspira pilosicoli B2904 GN=B2904_orf2664 PE=3 SV=1

Observed	Mr(expt)	Mr(calc)	ppm	Start		End	Miss	Ions	Peptide
1358.7311	1357.7238	1357.7024	15.8	195	-	206	0	60	R.IDEGIQMVVAQR.A
1543.8197	1542.8124	1542.7936	12.2	1	-	14	0	98	-.MIINNNVSALNANR.Q

No match to: 713.4055

3. K0JLS4 **Mass:** 30164 **Score:** 192 **Expect:** 2.6e-15 **Queries matched:** 2
K0JLS4_BRAPL Flagellin OS=Brachyspira pilosicoli WesB GN=flaB3 PE=3 SV=1

Observed	Mr(expt)	Mr(calc)	ppm	Start		End	Miss	Ions	Peptide
1358.7311	1357.7238	1357.7024	15.8	195	-	206	0	60	R.IDEGIQMVVAQR.A
1543.8197	1542.8124	1542.7936	12.2	1	-	14	0	98	-.MIINNNVSALNANR.Q

No match to: 713.4055

4. D8IDG1 **Mass:** 26584 **Score:** 75 **Expect:** 0.0012 **Queries matched:** 1
D8IDG1_BRAP9 Flagellin OS=Brachyspira pilosicoli (strain ATCC BAA-1826 / 95/1000) GN=flaB3 PE=3 SV=1

Observed	Mr(expt)	Mr(calc)	ppm	Start	End	Miss	Ions	Peptide
1358.7311	1357.7238	1357.7024	15.8	162 -	173	0	60	R.IDEGIQMVVAQR.A

No match to: 713.4055, 1543.8197

5. A0A0G4K947 **Mass:** 30002 **Score:** 75 **Expect:** 0.0014 **Queries matched:** 1
A0A0G4K947_9SPIR Flagellin OS=Brachyspira suanatina GN=flaB2 PE=3 SV=1

Observed	Mr(expt)	Mr(calc)	ppm	Start	End	Miss	Ions	Peptide
1358.7311	1357.7238	1357.7024	15.8	195 -	206	0	60	R.IDEGIQMVVAQR.A

No match to: 713.4055, 1543.8197

6. G0EIL9 **Mass:** 30029 **Score:** 75 **Expect:** 0.0014 **Queries matched:** 1
G0EIL9_BRAIP Flagellin OS=Brachyspira intermedia (strain ATCC 51140 / PWS/A) GN=flaB3 PE=3 SV=1

Observed	Mr(expt)	Mr(calc)	ppm	Start	End	Miss	Ions	Peptide
1358.7311	1357.7238	1357.7024	15.8	195 -	206	0	60	R.IDEGIQMVVAQR.A

No match to: 713.4055, 1543.8197

7. L0X7D1 **Mass:** 30068 **Score:** 75 **Expect:** 0.0014 **Queries matched:** 1
L0X7D1_9SPIR Flagellin OS=Brachyspira hampsonii 30446 GN=A966_03171 PE=3 SV=1

Observed	Mr(expt)	Mr(calc)	ppm	Start	End	Miss	Ions	Peptide
1358.7311	1357.7238	1357.7024	15.8	195 -	206	0	60	R.IDEGIQMVVAQR.A

No match to: 713.4055, 1543.8197

8. L8XR86 **Mass:** 30027 **Score:** 75 **Expect:** 0.0014 **Queries matched:** 1
L8XR86_9SPIR Flagellin OS=Brachyspira hampsonii 30599 GN=H263_04258 PE=3 SV=1

Observed	Mr(expt)	Mr(calc)	ppm	Start	End	Miss	Ions	Peptide
1358.7311	1357.7238	1357.7024	15.8	195 -	206	0	60	R.IDEGIQMVVAQR.A

No match to: 713.4055, 1543.8197

9. A0A0G4K9F5 **Mass:** 11224 **Score:** 42 **Expect:** 2.4 **Queries matched:** 2
A0A0G4K9F5_9SPIR Uncharacterized protein OS=Brachyspira suanatina GN=BRSU_2001 PE=4 SV=1

Observed	Mr(expt)	Mr(calc)	ppm	Start	End	Miss	Ions	Peptide
1358.7311	1357.7238	1357.7275	-2.70	7 -	17	1	---	K.EKPIMEEVQKK.Y
1543.8197	1542.8124	1542.7889	15.2	51 -	64	1	---	R.LGKEEGIEQGEINK.A

No match to: 713.4055

Search Parameters

Type of search : Sequence Query
Enzyme : Trypsin
Fixed modifications : Carbamidomethyl (C)
Variable modifications : Oxidation (M)
Mass values : Monoisotopic
Protein Mass : Unrestricted
Peptide Mass Tolerance : ± 50 ppm
Fragment Mass Tolerance: ± 0.5 Da
Max Missed Cleavages : 1
Instrument type : MALDI-TOF-TOF
Query1 (713.4055,1+) : Locus:8.1.1.170606.52758
Query2 (1358.7311,1+) : Locus:8.1.1.170606.52757
Query3 (1543.8197,1+) : Locus:8.1.1.170606.52756

Mascot: http://www.matrixscience.com/

MATRIX SCIENCE Mascot Search Results

```
User          :
Email         :
Search title  : F:\ROD_inmunoproteoma\MALDI\ppw_2014_442_ROD\ppw_G21_139601421720.txt
MS data file  : F:\ROD_inmunoproteoma\MALDI\ppw_2014_442_ROD\ppw_G21_139601421720.txt
Database      : Brachyspiraceae Brachyspiraceae_20160127-151122 (40573 sequences; 13470793 residues)
Timestamp     : 22 Feb 2016 at 15:14:39 GMT
Warning       : A Peptide summary report will usually give a much clearer picture of MS/MS search results.
Top Score     : 193 for R5LA18, R5LA18_9SPIR Flagellin OS=Brachyspira sp. CAG:700 GN=BN758_01499 PE=3 SV=1
```

Probability Based Mowse Score

Protein score is -10*Log(P), where P is the probability that the observed match is a random event.
Protein scores greater than 59 are significant (p<0.05).
Protein scores are derived from ions scores as a non-probabilistic basis for ranking protein hits.

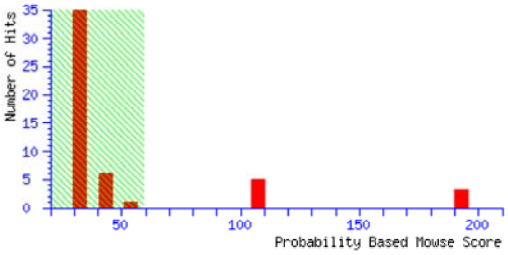

Protein Summary Report

Format As | Protein Summary (deprecated) | Help

Significance threshold p< 0.05 | Max. number of hits AUTO

Standard scoring ◉ MudPIT scoring ○ | Ions score or expect cut-off 0 | Show sub-sets 0

Show pop-ups ◉ Suppress pop-ups ○ | Sort unassigned Decreasing Score | Require bold red ☐

Re-Search All | Search Unmatched

Index

	Accession	Mass	Score	Description
1.	R5LA18	30135	193	R5LA18_9SPIR Flagellin OS=Brachyspira sp. CAG:700 GN=BN758_01499 PE=3 SV=1
2.	J9UXQ6	30134	193	J9UXQ6_BRAPL Flagellin OS=Brachyspira pilosicoli B2904 GN=B2904_orf2664 PE=3 SV=1
3.	K0JLS4	30164	193	K0JLS4_BRAPL Flagellin OS=Brachyspira pilosicoli WesB GN=flaB3 PE=3 SV=1
4.	D8IDG1	26584	108	D8IDG1_BRAP9 Flagellin OS=Brachyspira pilosicoli (strain ATCC BAA-1826 / 95/1000) GN=flaB3 PE=3 SV=1
5.	A0A0G4K947	30002	107	A0A0G4K947_9SPIR Flagellin OS=Brachyspira suanatina GN=flaB2 PE=3 SV=1
6.	G0EIL9	30029	107	G0EIL9_BRAIP Flagellin OS=Brachyspira intermedia (strain ATCC 51140 / PWS/A) GN=flaB3 PE=3 SV=1
7.	L0X7D1	30068	107	L0X7D1_9SPIR Flagellin OS=Brachyspira hampsonii 30446 GN=A966_03171 PE=3 SV=1
8.	L8XR86	30027	107	L8XR86_9SPIR Flagellin OS=Brachyspira hampsonii 30599 GN=H263_04258 PE=3 SV=1
9.	L8XUS2	41134	54	L8XUS2_9SPIR Alanine racemase OS=Brachyspira hampsonii 30599 GN=H263_06432 PE=3 SV=1

Results List

1. R5LA18 **Mass:** 30135 **Score:** 193 **Expect:** 2e-15 **Queries matched:** 3
R5LA18_9SPIR Flagellin OS=Brachyspira sp. CAG:700 GN=BN758_01499 PE=3 SV=1

Observed	Mr(expt)	Mr(calc)	ppm	Start	End	Miss	Ions	Peptide
1307.6901	1306.6828	1306.6742	6.62	54 -	64	1	28	R.SQYRGLQQATR.N
1358.7183	1357.7110	1357.7024	6.37	195 -	206	0	46	R.IDEGIQMVVAQR.A
1543.8168	1542.8095	1542.7936	10.3	1 -	14	0	61	-.MIINNNVSALNANR.Q

2. J9UXQ6 **Mass:** 30134 **Score:** 193 **Expect:** 2e-15 **Queries matched:** 3
J9UXQ6_BRAPL Flagellin OS=Brachyspira pilosicoli B2904 GN=B2904_orf2664 PE=3 SV=1

Observed	Mr(expt)	Mr(calc)	ppm	Start	End	Miss	Ions	Peptide
1307.6901	1306.6828	1306.6742	6.62	54 -	64	1	28	R.SQYRGLQQATR.N
1358.7183	1357.7110	1357.7024	6.37	195 -	206	0	46	R.IDEGIQMVVAQR.A
1543.8168	1542.8095	1542.7936	10.3	1 -	14	0	61	-.MIINNNVSALNANR.Q

3. K0JLS4 **Mass:** 30164 **Score:** 193 **Expect:** 2e-15 **Queries matched:** 3
K0JLS4_BRAPL Flagellin OS=Brachyspira pilosicoli WesB GN=flaB3 PE=3 SV=1

Observed	Mr(expt)	Mr(calc)	ppm	Start	End	Miss	Ions	Peptide
1307.6901	1306.6828	1306.6742	6.62	54 -	64	1	28	R.SQYRGLQQATR.N
1358.7183	1357.7110	1357.7024	6.37	195 -	206	0	46	R.IDEGIQMVVAQR.A
1543.8168	1542.8095	1542.7936	10.3	1 -	14	0	61	-.MIINNNVSALNANR.Q

4. D8IDG1 **Mass:** 26584 **Score:** 108 **Expect:** 6.4e-07 **Queries matched:** 2
D8IDG1_BRAP9 Flagellin OS=Brachyspira pilosicoli (strain ATCC BAA-1826 / 95/1000) GN=flaB3 PE=3 SV=1

Observed	Mr(expt)	Mr(calc)	ppm	Start	End	Miss	Ions	Peptide
1307.6901	1306.6828	1306.6742	6.62	21 -	31	1	28	R.SQYRGLQQATR.N
1358.7183	1357.7110	1357.7024	6.37	162 -	173	0	46	R.IDEGIQMVVAQR.A

No match to: 1543.8168

5. A0A0G4K947 **Mass:** 30002 **Score:** 107 **Expect:** 8.1e-07 **Queries matched:** 2
A0A0G4K947_9SPIR Flagellin OS=Brachyspira suanatina GN=flaB2 PE=3 SV=1

Observed	Mr(expt)	Mr(calc)	ppm	Start	End	Miss	Ions	Peptide
1307.6901	1306.6828	1306.6742	6.62	54 -	64	1	28	R.SQYRGLQQATR.N
1358.7183	1357.7110	1357.7024	6.37	195 -	206	0	46	R.IDEGIQMVVAQR.A

No match to: 1543.8168

6. G0EIL9 **Mass:** 30029 **Score:** 107 **Expect:** 8.1e-07 **Queries matched:** 2
G0EIL9_BRAIP Flagellin OS=Brachyspira intermedia (strain ATCC 51140 / PWS/A) GN=flaB3 PE=3 SV=1

Observed	Mr(expt)	Mr(calc)	ppm	Start	End	Miss	Ions	Peptide
1307.6901	1306.6828	1306.6742	6.62	54 -	64	1	28	R.SQYRGLQQATR.N
1358.7183	1357.7110	1357.7024	6.37	195 -	206	0	46	R.IDEGIQMVVAQR.A

No match to: 1543.8168

7. L0X7D1 **Mass:** 30068 **Score:** 107 **Expect:** 8.1e-07 **Queries matched:** 2
L0X7D1_9SPIR Flagellin OS=Brachyspira hampsonii 30446 GN=A966_03171 PE=3 SV=1

Observed	Mr(expt)	Mr(calc)	ppm	Start	End	Miss	Ions	Peptide
1307.6901	1306.6828	1306.6742	6.62	54 -	64	1	28	R.SQYRGLQQATR.N
1358.7183	1357.7110	1357.7024	6.37	195 -	206	0	46	R.IDEGIQMVVAQR.A

No match to: 1543.8168

8. L8XR86 **Mass:** 30027 **Score:** 107 **Expect:** 8.1e-07 **Queries matched:** 2
L8XR86_9SPIR Flagellin OS=Brachyspira hampsonii 30599 GN=H263_04258 PE=3 SV=1

Observed	Mr(expt)	Mr(calc)	ppm	Start	End	Miss	Ions	Peptide
1307.6901	1306.6828	1306.6742	6.62	54 -	64	1	28	R.SQYRGLQQATR.N
1358.7183	1357.7110	1357.7024	6.37	195 -	206	0	46	R.IDEGIQMVVAQR.A

No match to: 1543.8168

9. L8XUS2 **Mass:** 41134 **Score:** 54 **Expect:** 0.17 **Queries matched:** 3
L8XUS2_9SPIR Alanine racemase OS=Brachyspira hampsonii 30599 GN=H263_06432 PE=3 SV=1

Observed	Mr(expt)	Mr(calc)	ppm	Start	End	Miss	Ions	Peptide
1307.6901	1306.6828	1306.6405	32.4	322 -	333	0	---	K.TGDEVLIFGNDK.K
1358.7183	1357.7110	1357.6990	8.86	36 -	48	0	---	K.ANAYGHGLVEISK.A
1543.8168	1542.8095	1542.8552	-29.58	17 -	29	1	---	K.NMQIIRSIINDVK.L

Search Parameters

Type of search : Sequence Query
Enzyme : Trypsin
Fixed modifications : Carbamidomethyl (C)
Variable modifications : Oxidation (M)
Mass values : Monoisotopic
Protein Mass : Unrestricted
Peptide Mass Tolerance : ± 50 ppm
Fragment Mass Tolerance: ± 0.5 Da
Max Missed Cleavages : 1
Instrument type : MALDI-TOF-TOF
Query1 (1307.6901,1+) : Locus:21.1.1.170619.52795
Query2 (1358.7183,1+) : Locus:21.1.1.170619.52797
Query3 (1543.8168,1+) : Locus:21.1.1.170619.52796

Mascot: http://www.matrixscience.com/

Mascot Search Results

User	:
Email	:
Search title	: F:\ROD_inmunoproteoma\MALDI\ppw_2016_561_562_563_565\ppw_H11_145467559123.txt
MS data file	: F:\ROD_inmunoproteoma\MALDI\ppw_2016_561_562_563_565\ppw_H11_145467559123.txt
Database	: Brachyspiraceae Brachyspiraceae_20160127-151122 (40573 sequences; 13470793 residues)
Timestamp	: 23 Feb 2016 at 16:37:10 GMT
Warning	: A Peptide summary report will usually give a much clearer picture of MS/MS search results.
Top Score	: 396 for A0A0H0USH8, A0A0H0USH8_BRAHO Flagellin OS=Brachyspira hyodysenteriae GN=SR30_01525 PE=3 SV=1

Probability Based Mowse Score

Protein score is -10*Log(P), where P is the probability that the observed match is a random event.
Protein scores greater than 59 are significant (p<0.05).
Protein scores are derived from ions scores as a non-probabilistic basis for ranking protein hits.

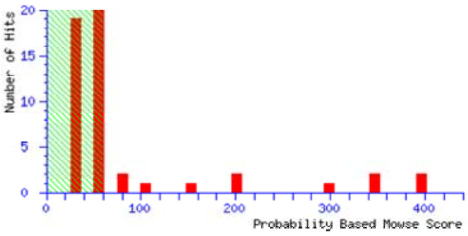

Protein Summary Report

Format As | Protein Summary (deprecated) | Help

Significance threshold p< 0.05 | Max. number of hits AUTO

Standard scoring ◉ MudPIT scoring ○ | Ions score or expect cut-off 0 | Show sub-sets 0

Show pop-ups ◉ Suppress pop-ups ○ | Sort unassigned Decreasing Score | Require bold red ☐

Re-Search All | Search Unmatched

Index

	Accession	Mass	Score	Description
1.	A0A0H0USH8	30059	396	A0A0H0USH8_BRAHO Flagellin OS=Brachyspira hyodysenteriae GN=SR30_01525 PE=3 SV=1
2.	Q9F0F6	30445	396	Q9F0F6_BRAHO Flagellin OS=Brachyspira hyodysenteriae GN=flaB3 PE=3 SV=1
3.	A0A0G4K947	30002	353	A0A0G4K947_9SPIR Flagellin OS=Brachyspira suanatina GN=flaB2 PE=3 SV=1
4.	G0EIL9	30029	353	G0EIL9_BRAIP Flagellin OS=Brachyspira intermedia (strain ATCC 51140 / PWS/A) GN=flaB3 PE=3 SV=1
5.	C0R1D6	27693	291	C0R1D6_BRAHW Flagellin OS=Brachyspira hyodysenteriae (strain ATCC 49526 / WA1) GN=flaB3 PE=3 SV=1
6.	L0X7D1	30068	200	L0X7D1_9SPIR Flagellin OS=Brachyspira hampsonii 30446 GN=A966_03171 PE=3 SV=1
7.	L8XR86	30027	200	L8XR86_9SPIR Flagellin OS=Brachyspira hampsonii 30599 GN=H263_04258 PE=3 SV=1
8.	D5U8W2	29993	145	D5U8W2_BRAM5 Flagellin OS=Brachyspira murdochii (strain ATCC 51284 / DSM 12563 / 56-150) GN=Bmur_1039 PE=3 SV=
9.	P80161	2744	106	FLAB3_BRAHO Flagellar filament core protein flaB3 (Fragment) OS=Brachyspira hyodysenteriae GN=flaB3 PE=1 SV=1
10.	L8XSM5	47036	76	L8XSM5_9SPIR Enolase OS=Brachyspira hampsonii 30599 GN=eno PE=3 SV=1
11.	L0X4B5	46993	76	L0X4B5_9SPIR Enolase OS=Brachyspira hampsonii 30446 GN=eno PE=3 SV=1
12.	J9UYC2	31304	65	J9UYC2_BRAPL Flagellin OS=Brachyspira pilosicoli B2904 GN=B2904_orf2581 PE=3 SV=1
13.	K0JHQ4	31288	65	K0JHQ4_BRAPL Flagellin OS=Brachyspira pilosicoli WesB GN=flaB2 PE=3 SV=1
14.	A0A0H0W3D6	47082	61	A0A0H0W3D6_BRAHO Enolase OS=Brachyspira hyodysenteriae GN=eno PE=3 SV=1
15.	A0A0G4KAH6	47151	61	A0A0G4KAH6_9SPIR Enolase OS=Brachyspira suanatina GN=eno PE=3 SV=1
16.	C0QV52	47082	61	C0QV52_BRAHW Enolase OS=Brachyspira hyodysenteriae (strain ATCC 49526 / WA1) GN=eno PE=3 SV=1
17.	A0A0H0XEZ9	47112	61	A0A0H0XEZ9_BRAHO Enolase OS=Brachyspira hyodysenteriae GN=eno PE=3 SV=1
18.	G0EQW4	47138	61	G0EQW4_BRAIP Enolase OS=Brachyspira intermedia (strain ATCC 51140 / PWS/A) GN=eno PE=3 SV=1
19.	J9URW4	47046	61	J9URW4_BRAPL Enolase OS=Brachyspira pilosicoli B2904 GN=eno PE=3 SV=1
20.	D8IET2	47046	61	D8IET2_BRAP9 Enolase OS=Brachyspira pilosicoli (strain ATCC BAA-1826 / 95/1000) GN=eno PE=3 SV=1
21.	K0JI69	47046	61	K0JI69_BRAPL Enolase OS=Brachyspira pilosicoli WesB GN=eno PE=3 SV=1
22.	D5U9W9	47170	61	D5U9W9_BRAM5 Enolase OS=Brachyspira murdochii (strain ATCC 51284 / DSM 12563 / 56-150) GN=eno PE=3 SV=1
23.	D8IDG1	26584	49	D8IDG1_BRAP9 Flagellin OS=Brachyspira pilosicoli (strain ATCC BAA-1826 / 95/1000) GN=flaB3 PE=3 SV=1

Results List

1. A0A0H0USH8 **Mass:** 30059 **Score:** 396 **Expect:** 1e-35 **Queries matched:** 4
A0A0H0USH8_BRAHO Flagellin OS=Brachyspira hyodysenteriae GN=SR30_01525 PE=3 SV=1

Observed	Mr(expt)	Mr(calc)	ppm	Start		End	Miss	Ions	Peptide
1063.5970	1062.5897	1062.5492	38.2	26	-	35	0	33	K.TIAQLSSGMR.I
1374.7489	1373.7416	1373.6973	32.3	195	-	206	0	80	R.IDEGIQMVVSQR.A
1557.8683	1556.8610	1556.8093	33.3	1	-	14	0	84	-.MIINNNISALNANR.Q
2208.0667	2207.0594	2206.9888	32.0	140	-	159	0	129	R.FAADGQTPMTFHIGANMDQR.V

No match to: 1320.7006

2. Q9F0F6 **Mass:** 30445 **Score:** 396 **Expect:** 1e-35 **Queries matched:** 4
Q9F0F6_BRAHO Flagellin OS=Brachyspira hyodysenteriae GN=flaB3 PE=3 SV=1

Observed	Mr(expt)	Mr(calc)	ppm	Start		End	Miss	Ions	Peptide
1063.5970	1062.5897	1062.5492	38.2	26	-	35	0	33	K.TIAQLSSGMR.I
1374.7489	1373.7416	1373.6973	32.3	195	-	206	0	80	R.IDEGIQMVVSQR.A
1557.8683	1556.8610	1556.8093	33.3	1	-	14	0	84	-.MIINNNISALNANR.Q
2208.0667	2207.0594	2206.9888	32.0	140	-	159	0	129	R.FAADGQTPMTFHIGANMDQR.V

No match to: 1320.7006

3. A0A0G4K947 **Mass:** 30002 **Score:** 353 **Expect:** 2e-31 **Queries matched:** 4
A0A0G4K947_9SPIR Flagellin OS=Brachyspira suanatina GN=flaB2 PE=3 SV=1

Observed	Mr(expt)	Mr(calc)	ppm	Start	End	Miss	Ions	Peptide
1063.5970	1062.5897	1062.5492	38.2	26 -	35	0	33	K.TIAQLSSGMR.I
1374.7489	1373.7416	1373.6973	32.3	195 -	206	0	37	R.IDEGIQMVVAQR.A + Oxidation (M)
1557.8683	1556.8610	1556.8093	33.3	1 -	14	0	84	-.MIINNNISALNANR.Q
2208.0667	2207.0594	2206.9888	32.0	140 -	159	0	129	R.FAADGQTPMTFHIGANMDQR.V

No match to: 1320.7006

4. G0EIL9 **Mass:** 30029 **Score:** 353 **Expect:** 2e-31 **Queries matched:** 4
G0EIL9_BRAIP Flagellin OS=Brachyspira intermedia (strain ATCC 51140 / PWS/A) GN=flaB3 PE=3 SV=1

Observed	Mr(expt)	Mr(calc)	ppm	Start	End	Miss	Ions	Peptide
1063.5970	1062.5897	1062.5492	38.2	26 -	35	0	33	K.TIAQLSSGMR.I
1374.7489	1373.7416	1373.6973	32.3	195 -	206	0	37	R.IDEGIQMVVAQR.A + Oxidation (M)
1557.8683	1556.8610	1556.8093	33.3	1 -	14	0	84	-.MIINNNISALNANR.Q
2208.0667	2207.0594	2206.9888	32.0	140 -	159	0	129	R.FAADGQTPMTFHIGANMDQR.V

No match to: 1320.7006

5. C0R1D6 **Mass:** 27693 **Score:** 291 **Expect:** 3.2e-25 **Queries matched:** 3
C0R1D6_BRAHW Flagellin OS=Brachyspira hyodysenteriae (strain ATCC 49526 / WA1) GN=flaB3 PE=3 SV=1

Observed	Mr(expt)	Mr(calc)	ppm	Start	End	Miss	Ions	Peptide
1063.5970	1062.5897	1062.5492	38.2	4 -	13	0	33	K.TIAQLSSGMR.I
1374.7489	1373.7416	1373.6973	32.3	173 -	184	0	80	R.IDEGIQMVVSQR.A
2208.0667	2207.0594	2206.9888	32.0	118 -	137	0	129	R.FAADGQTPMTFHIGANMDQR.V

No match to: 1320.7006, 1557.8683

6. L0X7D1 **Mass:** 30068 **Score:** 200 **Expect:** 4.1e-16 **Queries matched:** 3
L0X7D1_9SPIR Flagellin OS=Brachyspira hampsonii 30446 GN=A966_03171 PE=3 SV=1

Observed	Mr(expt)	Mr(calc)	ppm	Start	End	Miss	Ions	Peptide
1063.5970	1062.5897	1062.5492	38.2	26 -	35	0	33	K.TIAQLSSGMR.I
1374.7489	1373.7416	1373.6973	32.3	195 -	206	0	37	R.IDEGIQMVVAQR.A + Oxidation (M)
1557.8683	1556.8610	1556.8093	33.3	1 -	14	0	84	-.MIINNNISALNANR.Q

No match to: 1320.7006, 2208.0667

7. L8XR86 **Mass:** 30027 **Score:** 200 **Expect:** 4.1e-16 **Queries matched:** 3
L8XR86_9SPIR Flagellin OS=Brachyspira hampsonii 30599 GN=H263_04258 PE=3 SV=1

Observed	Mr(expt)	Mr(calc)	ppm	Start	End	Miss	Ions	Peptide
1063.5970	1062.5897	1062.5492	38.2	26 -	35	0	33	K.TIAQLSSGMR.I
1374.7489	1373.7416	1373.6973	32.3	195 -	206	0	37	R.IDEGIQMVVAQR.A + Oxidation (M)
1557.8683	1556.8610	1556.8093	33.3	1 -	14	0	84	-.MIINNNISALNANR.Q

No match to: 1320.7006, 2208.0667

8. D5U8W2 **Mass:** 29993 **Score:** 145 **Expect:** 1.3e-10 **Queries matched:** 2
D5U8W2_BRAM5 Flagellin OS=Brachyspira murdochii (strain ATCC 51284 / DSM 12563 / 56-150) GN=Bmur_1039 PE=3 SV=1

Observed	Mr(expt)	Mr(calc)	ppm	Start	End	Miss	Ions	Peptide
1063.5970	1062.5897	1062.5492	38.2	26 -	35	0	33	K.TIAQLSSGMR.I
1557.8683	1556.8610	1556.8093	33.3	1 -	14	0	84	-.MIINNNISALNANR.Q

No match to: 1320.7006, 1374.7489, 2208.0667

9. P80161 **Mass:** 2744 **Score:** 106 **Expect:** 1e-06 **Queries matched:** 1
FLAB3_BRAHO Flagellar filament core protein flaB3 (Fragment) OS=Brachyspira hyodysenteriae GN=flaB3 PE=1 SV=1

Observed	Mr(expt)	Mr(calc)	ppm	Start	End	Miss	Ions	Peptide
1557.8683	1556.8610	1556.8093	33.3	1 -	14	0	84	-.MIINNNISALNANR.Q

No match to: 1063.5970, 1320.7006, 1374.7489, 2208.0667

10. L8XSM5 **Mass:** 47036 **Score:** 76 **Expect:** 0.001 **Queries matched:** 2
L8XSM5_9SPIR Enolase OS=Brachyspira hampsonii 30599 GN=eno PE=3 SV=1

Observed	Mr(expt)	Mr(calc)	ppm	Start	End	Miss	Ions	Peptide
1320.7006	1319.6933	1319.6543	29.6	121 -	132	0	50	K.AAAEELGMPLYR.Y
2208.0667	2207.0594	2207.0059	24.3	282 -	300	0	---	K.YPIISIEDGVAEDDWDGWK.I

No match to: 1063.5970, 1374.7489, 1557.8683

11. L0X4B5 **Mass:** 46993 **Score:** 76 **Expect:** 0.001 **Queries matched:** 2
L0X4B5_9SPIR Enolase OS=Brachyspira hampsonii 30446 GN=eno PE=3 SV=1

Observed	Mr(expt)	Mr(calc)	ppm	Start	End	Miss	Ions	Peptide
1320.7006	1319.6933	1319.6543	29.6	121 -	132	0	50	K.AAAEELGMPLYR.Y
2208.0667	2207.0594	2207.0059	24.3	282 -	300	0	---	K.YPIISIEDGVAEDDWDGWK.I

No match to: 1063.5970, 1374.7489, 1557.8683

12. J9UYC2 **Mass:** 31304 **Score:** 65 **Expect:** 0.014 **Queries matched:** 1
J9UYC2_BRAPL Flagellin OS=Brachyspira pilosicoli B2904 GN=B2904_orf2581 PE=3 SV=1

Observed	Mr(expt)	Mr(calc)	ppm	Start	End	Miss	Ions	Peptide
1557.8683	1556.8610	1556.8093	33.2	1 -	14	0	52	-.MIINNNISAVNAQR.T

No match to: 1063.5970, 1320.7006, 1374.7489, 2208.0667

13. K0JHQ4 **Mass:** 31288 **Score:** 65 **Expect:** 0.014 **Queries matched:** 1
K0JHQ4_BRAPL Flagellin OS=Brachyspira pilosicoli WesB GN=flaB2 PE=3 SV=1

Observed	Mr(expt)	Mr(calc)	ppm	Start	End	Miss	Ions	Peptide
1557.8683	1556.8610	1556.8093	33.2	1 -	14	0	52	-.MIINNNISAVNAQR.T

No match to: 1063.5970, 1320.7006, 1374.7489, 2208.0667

14. A0A0H0W3D6 **Mass:** 47082 **Score:** 61 **Expect:** 0.035 **Queries matched:** 1
A0A0H0W3D6_BRAHO Enolase OS=Brachyspira hyodysenteriae GN=eno PE=3 SV=1

Observed	Mr(expt)	Mr(calc)	ppm	Start	End	Miss	Ions	Peptide
1320.7006	1319.6933	1319.6543	29.6	121 -	132	0	50	K.AAAEELGMPLYR.Y

No match to: 1063.5970, 1374.7489, 1557.8683, 2208.0667

15. A0A0G4KAH6 **Mass:** 47151 **Score:** 61 **Expect:** 0.035 **Queries matched:** 1
A0A0G4KAH6_9SPIR Enolase OS=Brachyspira suanatina GN=eno PE=3 SV=1

Observed	Mr(expt)	Mr(calc)	ppm	Start	End	Miss	Ions	Peptide
1320.7006	1319.6933	1319.6543	29.6	121 -	132	0	50	K.AAAEELGMPLYR.Y

No match to: 1063.5970, 1374.7489, 1557.8683, 2208.0667

16. C0QV52 **Mass:** 47082 **Score:** 61 **Expect:** 0.035 **Queries matched:** 1
C0QV52_BRAHW Enolase OS=Brachyspira hyodysenteriae (strain ATCC 49526 / WA1) GN=eno PE=3 SV=1

Observed	Mr(expt)	Mr(calc)	ppm	Start	End	Miss	Ions	Peptide
1320.7006	1319.6933	1319.6543	29.6	121 -	132	0	50	K.AAAEELGMPLYR.Y

No match to: 1063.5970, 1374.7489, 1557.8683, 2208.0667

17. A0A0H0XEZ9 **Mass:** 47112 **Score:** 61 **Expect:** 0.035 **Queries matched:** 1
A0A0H0XEZ9_BRAHO Enolase OS=Brachyspira hyodysenteriae GN=eno PE=3 SV=1

Observed	Mr(expt)	Mr(calc)	ppm	Start	End	Miss	Ions	Peptide
1320.7006	1319.6933	1319.6543	29.6	121 -	132	0	50	K.AAAEELGMPLYR.Y

No match to: 1063.5970, 1374.7489, 1557.8683, 2208.0667

18. G0EQW4 **Mass:** 47138 **Score:** 61 **Expect:** 0.035 **Queries matched:** 1
G0EQW4_BRAIP Enolase OS=Brachyspira intermedia (strain ATCC 51140 / PWS/A) GN=eno PE=3 SV=1

Observed	Mr(expt)	Mr(calc)	ppm	Start	End	Miss	Ions	Peptide
1320.7006	1319.6933	1319.6543	29.6	121 -	132	0	50	K.AAAEELGMPLYR.Y

No match to: 1063.5970, 1374.7489, 1557.8683, 2208.0667

19. J9URW4 **Mass:** 47046 **Score:** 61 **Expect:** 0.035 **Queries matched:** 1
J9URW4_BRAPL Enolase OS=Brachyspira pilosicoli B2904 GN=eno PE=3 SV=1

Observed	Mr(expt)	Mr(calc)	ppm	Start	End	Miss	Ions	Peptide
1320.7006	1319.6933	1319.6543	29.6	121 -	132	0	50	K.AAAEELGMPLYR.Y

No match to: 1063.5970, 1374.7489, 1557.8683, 2208.0667

20. D8IET2 **Mass:** 47046 **Score:** 61 **Expect:** 0.035 **Queries matched:** 1
D8IET2_BRAP9 Enolase OS=Brachyspira pilosicoli (strain ATCC BAA-1826 / 95/1000) GN=eno PE=3 SV=1

Observed	Mr(expt)	Mr(calc)	ppm	Start	End	Miss	Ions	Peptide
1320.7006	1319.6933	1319.6543	29.6	121 -	132	0	50	K.AAAEELGMPLYR.Y

No match to: 1063.5970, 1374.7489, 1557.8683, 2208.0667

21. K0JI69 **Mass:** 47046 **Score:** 61 **Expect:** 0.035 **Queries matched:** 1
K0JI69_BRAPL Enolase OS=Brachyspira pilosicoli WesB GN=eno PE=3 SV=1

Observed	Mr(expt)	Mr(calc)	ppm	Start	End	Miss	Ions	Peptide
1320.7006	1319.6933	1319.6543	29.6	121 -	132	0	50	K.AAAEELGMPLYR.Y

No match to: 1063.5970, 1374.7489, 1557.8683, 2208.0667

22. D5U9W9 **Mass:** 47170 **Score:** 61 **Expect:** 0.035 **Queries matched:** 1
D5U9W9_BRAM5 Enolase OS=Brachyspira murdochii (strain ATCC 51284 / DSM 12563 / 56-150) GN=eno PE=3 SV=1

Observed	Mr(expt)	Mr(calc)	ppm	Start	End	Miss	Ions	Peptide
1320.7006	1319.6933	1319.6543	29.6	121 -	132	0	50	K.AAAEELGMPLYR.Y

No match to: 1063.5970, 1374.7489, 1557.8683, 2208.0667

23. D8IDG1 **Mass:** 26584 **Score:** 49 **Expect:** 0.48 **Queries matched:** 1
D8IDG1_BRAP9 Flagellin OS=Brachyspira pilosicoli (strain ATCC BAA-1826 / 95/1000) GN=flaB3 PE=3 SV=1

Observed	Mr(expt)	Mr(calc)	ppm	Start	End	Miss	Ions	Peptide
1374.7489	1373.7416	1373.6973	32.3	162 -	173	0	37	R.IDEGIQMVVAQR.A + Oxidation (M)

No match to: 1063.5970, 1320.7006, 1557.8683, 2208.0667

Search Parameters

Type of search : Sequence Query
Enzyme : Trypsin
Fixed modifications : Carbamidomethyl (C)
Variable modifications : Oxidation (M)
Mass values : Monoisotopic
Protein Mass : Unrestricted
Peptide Mass Tolerance : ± 50 ppm
Fragment Mass Tolerance: ± 0.5 Da
Max Missed Cleavages : 1
Instrument type : MALDI-TOF-TOF
Query1 (1063.5970,1+) : Locus:11.1.1.212745.57052
Query2 (1320.7006,1+) : Locus:11.1.1.212745.57053
Query3 (1374.7489,1+) : Locus:11.1.1.212745.57051
Query4 (1557.8683,1+) : Locus:11.1.1.212745.57050
Query5 (2208.0667,1+) : Locus:11.1.1.212745.57049

Mascot: http://www.matrixscience.com/

MATRIX SCIENCE Mascot Search Results

```
User          :
Email         :
Search title  : F:\ROD_inmunoproteoma\MALDI\160223_2016_561_562_ROD\ppw_G11_145631468418.txt
MS data file  : F:\ROD_inmunoproteoma\MALDI\160223_2016_561_562_ROD\ppw_G11_145631468418.txt
Database      : Brachyspiraceae Brachyspiraceae_20160127-151122 (40573 sequences; 13470793 residues)
Timestamp     : 24 Feb 2016 at 12:03:08 GMT
Warning       : A Peptide summary report will usually give a much clearer picture of MS/MS search results.
Top Score     : 323 for D8IDG1, D8IDG1_BRAP9 Flagellin OS=Brachyspira pilosicoli (strain ATCC BAA-1826 / 95/1000) GN=flaB3 PE=3 SV=1
```

Probability Based Mowse Score

Protein score is -10*Log(P), where P is the probability that the observed match is a random event.
Protein scores greater than 59 are significant (p<0.05).
Protein scores are derived from ions scores as a non-probabilistic basis for ranking protein hits.

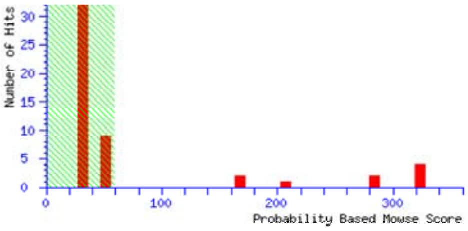

Protein Summary Report

Format As [Protein Summary (deprecated)] Help

Significance threshold p< [0.05] Max. number of hits [AUTO]

Standard scoring ◉ MudPIT scoring ○ Ions score or expect cut-off [0] Show sub-sets [0]

Show pop-ups ◉ Suppress pop-ups ○ Sort unassigned [Decreasing Score] Require bold red ☐

[Re-Search All] [Search Unmatched]

Index

	Accession	Mass	Score	Description
1.	D8IDG1	26584	323	D8IDG1_BRAP9 Flagellin OS=Brachyspira pilosicoli (strain ATCC BAA-1826 / 95/1000) GN=flaB3 PE=3 SV=1
2.	L0X7D1	30068	321	L0X7D1_9SPIR Flagellin OS=Brachyspira hampsonii 30446 GN=A966_03171 PE=3 SV=1
3.	L8XR86	30027	321	L8XR86_9SPIR Flagellin OS=Brachyspira hampsonii 30599 GN=H263_04258 PE=3 SV=1
4.	R5LA18	30135	320	R5LA18_9SPIR Flagellin OS=Brachyspira sp. CAG:700 GN=BN758_01499 PE=3 SV=1
5.	J9UXQ6	30134	291	J9UXQ6_BRAPL Flagellin OS=Brachyspira pilosicoli B2904 GN=B2904_orf2664 PE=3 SV=1
6.	K0JLS4	30164	291	K0JLS4_BRAPL Flagellin OS=Brachyspira pilosicoli WesB GN=flaB3 PE=3 SV=1
7.	D5U8W2	29993	206	D5U8W2_BRAM5 Flagellin OS=Brachyspira murdochii (strain ATCC 51284 / DSM 12563 / 56-150) GN=Bmur_1039 PE=3 SV=
8.	A0A0G4K947	30002	163	A0A0G4K947_9SPIR Flagellin OS=Brachyspira suanatina GN=flaB2 PE=3 SV=1
9.	G0EIL9	30029	163	G0EIL9_BRAIP Flagellin OS=Brachyspira intermedia (strain ATCC 51140 / PWS/A) GN=flaB3 PE=3 SV=1
10.	T1WF92	20075	53	T1WF92_9SPIR 60 kDa chaperonin (Fragment) OS=Brachyspira sp. CH-013 GN=cpn60 PE=4 SV=1

Results List

1. D8IDG1 **Mass:** 26584 **Score:** 323 **Expect:** 2e-28 **Queries matched:** 3
D8IDG1_BRAP9 Flagellin OS=Brachyspira pilosicoli (strain ATCC BAA-1826 / 95/1000) GN=flaB3 PE=3 SV=1

Observed	Mr(expt)	Mr(calc)	ppm	Start	End	Miss	Ions	Peptide
1307.7197	1306.7124	1306.6742	29.3	21 -	31	1	39	R.SQYRGLQQATR.N
1358.7521	1357.7448	1357.7024	31.3	162 -	173	0	96	R.IDEGIQMVVAQR.A
2222.0876	2221.0803	2221.0045	34.2	107 -	126	0	138	R.FAADAQTPMTFHIGANMDQR.V

No match to: 1372.7646, 1543.8564

2. L0X7D1 **Mass:** 30068 **Score:** 321 **Expect:** 3.2e-28 **Queries matched:** 3
L0X7D1_9SPIR Flagellin OS=Brachyspira hampsonii 30446 GN=A966_03171 PE=3 SV=1

Observed	Mr(expt)	Mr(calc)	ppm	Start	End	Miss	Ions	Peptide
1307.7197	1306.7124	1306.6742	29.3	54 -	64	1	39	R.SQYRGLQQATR.N
1358.7521	1357.7448	1357.7024	31.3	195 -	206	0	96	R.IDEGIQMVVAQR.A
2222.0876	2221.0803	2221.0045	34.2	140 -	159	0	138	R.FAADAQTPMTFHIGANMDQR.V

No match to: 1372.7646, 1543.8564

3. L8XR86 **Mass:** 30027 **Score:** 321 **Expect:** 3.2e-28 **Queries matched:** 3
L8XR86_9SPIR Flagellin OS=Brachyspira hampsonii 30599 GN=H263_04258 PE=3 SV=1

Observed	Mr(expt)	Mr(calc)	ppm	Start	End	Miss	Ions	Peptide
1307.7197	1306.7124	1306.6742	29.3	54 -	64	1	39	R.SQYRGLQQATR.N
1358.7521	1357.7448	1357.7024	31.3	195 -	206	0	96	R.IDEGIQMVVAQR.A
2222.0876	2221.0803	2221.0045	34.2	140 -	159	0	138	R.FAADAQTPMTFHIGANMDQR.V

No match to: 1372.7646, 1543.8564

4. R5LA18 **Mass:** 30135 **Score:** 320 **Expect:** 4.1e-28 **Queries matched:** 4
R5LA18_9SPIR Flagellin OS=Brachyspira sp. CAG:700 GN=BN758_01499 PE=3 SV=1

Observed	Mr(expt)	Mr(calc)	ppm	Start	End	Miss	Ions	Peptide
1307.7197	1306.7124	1306.6742	29.3	54 -	64	1	39	R.SQYRGLQQATR.N
1358.7521	1357.7448	1357.7024	31.3	195 -	206	0	96	R.IDEGIQMVVAQR.A

1543.8564	1542.8491	1542.7936	36.0	1 -	14	0	109	-.MIINNNVSALNANR.Q
2222.0876	2221.0803	2221.0222	26.2	140 -	159	0	5	R.FASDGQTPITFHIGANMDQR.V + Oxidation (M)

No match to: 1372.7646

5. [J9UXQ6] **Mass:** 30134 **Score:** 291 **Expect:** 3.2e-25 **Queries matched:** 3
J9UXQ6_BRAPL Flagellin OS=Brachyspira pilosicoli B2904 GN=B2904_orf2664 PE=3 SV=1

Observed	Mr(expt)	Mr(calc)	ppm	Start	End	Miss	Ions	Peptide
1307.7197	1306.7124	1306.6742	29.3	54 -	64	1	39	R.SQYRGLQQATR.N
1358.7521	1357.7448	1357.7024	31.3	195 -	206	0	96	R.IDEGIQMVVAQR.A
1543.8564	1542.8491	1542.7936	36.0	1 -	14	0	109	-.MIINNNVSALNANR.Q

No match to: 1372.7646, 2222.0876

6. [K0JLS4] **Mass:** 30164 **Score:** 291 **Expect:** 3.2e-25 **Queries matched:** 3
K0JLS4_BRAPL Flagellin OS=Brachyspira pilosicoli WesB GN=flaB3 PE=3 SV=1

Observed	Mr(expt)	Mr(calc)	ppm	Start	End	Miss	Ions	Peptide
1307.7197	1306.7124	1306.6742	29.3	54 -	64	1	39	R.SQYRGLQQATR.N
1358.7521	1357.7448	1357.7024	31.3	195 -	206	0	96	R.IDEGIQMVVAQR.A
1543.8564	1542.8491	1542.7936	36.0	1 -	14	0	109	-.MIINNNVSALNANR.Q

No match to: 1372.7646, 2222.0876

7. [D5U8W2] **Mass:** 29993 **Score:** 206 **Expect:** 1e-16 **Queries matched:** 2
D5U8W2_BRAM5 Flagellin OS=Brachyspira murdochii (strain ATCC 51284 / DSM 12563 / 56-150) GN=Bmur_1039 PE=3 SV=1

Observed	Mr(expt)	Mr(calc)	ppm	Start	End	Miss	Ions	Peptide
1307.7197	1306.7124	1306.6742	29.3	54 -	64	1	39	R.SQYRGLQQATR.N
2222.0876	2221.0803	2221.0045	34.2	140 -	159	0	138	R.FAADAQTPMTFHIGANMDQR.V

No match to: 1358.7521, 1372.7646, 1543.8564

8. [A0A0G4K947] **Mass:** 30002 **Score:** 163 **Expect:** 2e-12 **Queries matched:** 2
A0A0G4K947_9SPIR Flagellin OS=Brachyspira suanatina GN=flaB2 PE=3 SV=1

Observed	Mr(expt)	Mr(calc)	ppm	Start	End	Miss	Ions	Peptide
1307.7197	1306.7124	1306.6742	29.3	54 -	64	1	39	R.SQYRGLQQATR.N
1358.7521	1357.7448	1357.7024	31.3	195 -	206	0	96	R.IDEGIQMVVAQR.A

No match to: 1372.7646, 1543.8564, 2222.0876

9. [G0EIL9] **Mass:** 30029 **Score:** 163 **Expect:** 2e-12 **Queries matched:** 2
G0EIL9_BRAIP Flagellin OS=Brachyspira intermedia (strain ATCC 51140 / PWS/A) GN=flaB3 PE=3 SV=1

Observed	Mr(expt)	Mr(calc)	ppm	Start	End	Miss	Ions	Peptide
1307.7197	1306.7124	1306.6742	29.3	54 -	64	1	39	R.SQYRGLQQATR.N
1358.7521	1357.7448	1357.7024	31.3	195 -	206	0	96	R.IDEGIQMVVAQR.A

No match to: 1372.7646, 1543.8564, 2222.0876

10. [T1WF92] **Mass:** 20075 **Score:** 53 **Expect:** 0.22 **Queries matched:** 3
T1WF92_9SPIR 60 kDa chaperonin (Fragment) OS=Brachyspira sp. CH-013 GN=cpn60 PE=4 SV=1

Observed	Mr(expt)	Mr(calc)	ppm	Start	End	Miss	Ions	Peptide
1358.7521	1357.7448	1357.7453	-0.34	78 -	90	1	---	K.VGKEGVITVEEAK.S
1372.7646	1371.7573	1371.7544	2.14	15 -	27	1	---	K.NVTSGANPMLIKK.G
1543.8564	1542.8491	1542.8691	-12.92	136 -	148	1	---	K.ISNMKELLPILEK.I + Oxidation (M)

No match to: 1307.7197, 2222.0876

Search Parameters

Type of search : Sequence Query
Enzyme : Trypsin
Fixed modifications : Carbamidomethyl (C)
Variable modifications : Oxidation (M)
Mass values : Monoisotopic
Protein Mass : Unrestricted
Peptide Mass Tolerance : ± 50 ppm
Fragment Mass Tolerance: ± 0.5 Da
Max Missed Cleavages : 1
Instrument type : MALDI-TOF-TOF
Query1 (1307.7197,1+) : Locus:19.1.1.213518.57329
Query2 (1358.7521,1+) : Locus:19.1.1.213518.57328
Query3 (1372.7646,1+) : Locus:19.1.1.213518.57330
Query4 (1543.8564,1+) : Locus:19.1.1.213518.57326
Query5 (2222.0876,1+) : Locus:19.1.1.213518.57327

Mascot: http://www.matrixscience.com/

Mascot Search Results

```
User          :
Email         :
Search title  : F:\ROD_inmunoproteoma\MALDI\ppw_2016_562_563_ROD\ppw_I8_145397049824.txt
MS data file  : F:\ROD_inmunoproteoma\MALDI\ppw_2016_562_563_ROD\ppw_I8_145397049824.txt
Database      : Brachyspiraceae Brachyspiraceae_20160127-151122 (40573 sequences; 13470793 residues)
Timestamp     : 23 Feb 2016 at 16:06:04 GMT
Warning       : A Peptide summary report will usually give a much clearer picture of MS/MS search results.
Top Score     : 668 for A0A0H0USH8, A0A0H0USH8_BRAHO Flagellin OS=Brachyspira hyodysenteriae GN=SR30_01525 PE=3 SV=1
```

Probability Based Mowse Score

Protein score is -10*Log(P), where P is the probability that the observed match is a random event.
Protein scores greater than 59 are significant (p<0.05).
Protein scores are derived from ions scores as a non-probabilistic basis for ranking protein hits.

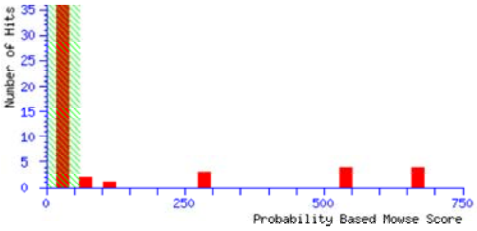

Protein Summary Report

Format As | Protein Summary (deprecated) | Help

Significance threshold p< 0.05 | Max. number of hits AUTO

Standard scoring ◉ MudPIT scoring ○ | Ions score or expect cut-off 0 | Show sub-sets 0

Show pop-ups ◉ Suppress pop-ups ○ | Sort unassigned Decreasing Score | Require bold red ☐

Re-Search All | Search Unmatched

Index

	Accession	Mass	Score	Description
1.	A0A0H0USH8	30059	668	A0A0H0USH8_BRAHO Flagellin OS=Brachyspira hyodysenteriae GN=SR30_01525 PE=3 SV=1
2.	A0A0G4K947	30002	668	A0A0G4K947_9SPIR Flagellin OS=Brachyspira suanatina GN=flaB2 PE=3 SV=1
3.	G0EIL9	30029	668	G0EIL9_BRAIP Flagellin OS=Brachyspira intermedia (strain ATCC 51140 / PWS/A) GN=flaB3 PE=3 SV=1
4.	Q9F0F6	30445	668	Q9F0F6_BRAHO Flagellin OS=Brachyspira hyodysenteriae GN=flaB3 PE=3 SV=1
5.	C0R1D6	27693	539	C0R1D6_BRAHW Flagellin OS=Brachyspira hyodysenteriae (strain ATCC 49526 / WA1) GN=flaB3 PE=3 SV=1
6.	D5U8W2	29993	524	D5U8W2_BRAM5 Flagellin OS=Brachyspira murdochii (strain ATCC 51284 / DSM 12563 / 56-150) GN=Bmur_1039 PE=3 SV=
7.	L0X7D1	30068	524	L0X7D1_9SPIR Flagellin OS=Brachyspira hampsonii 30446 GN=A966_03171 PE=3 SV=1
8.	L8XR86	30027	524	L8XR86_9SPIR Flagellin OS=Brachyspira hampsonii 30599 GN=H263_04258 PE=3 SV=1
9.	J9UXQ6	30134	297	J9UXQ6_BRAPL Flagellin OS=Brachyspira pilosicoli B2904 GN=B2904_orf2664 PE=3 SV=1
10.	K0JLS4	30164	297	K0JLS4_BRAPL Flagellin OS=Brachyspira pilosicoli WesB GN=flaB3 PE=3 SV=1
11.	D8IDG1	26584	296	D8IDG1_BRAP9 Flagellin OS=Brachyspira pilosicoli (strain ATCC BAA-1826 / 95/1000) GN=flaB3 PE=3 SV=1
12.	P80161	2744	126	FLAB3_BRAHO Flagellar filament core protein flaB3 (Fragment) OS=Brachyspira hyodysenteriae GN=flaB3 PE=1 SV=1
13.	J9UYC2	31304	82	J9UYC2_BRAPL Flagellin OS=Brachyspira pilosicoli B2904 GN=B2904_orf2581 PE=3 SV=1
14.	K0JHQ4	31288	82	K0JHQ4_BRAPL Flagellin OS=Brachyspira pilosicoli WesB GN=flaB2 PE=3 SV=1
15.	L8XVF9	33845	40	L8XVF9_9SPIR N-acetylneuraminate lyase OS=Brachyspira hampsonii 30599 GN=H263_04848 PE=3 SV=1

Results List

1. A0A0H0USH8 **Mass:** 30059 **Score:** 668 **Expect:** 6.4e-63 **Queries matched:** 5
A0A0H0USH8_BRAHO Flagellin OS=Brachyspira hyodysenteriae GN=SR30_01525 PE=3 SV=1

Observed	Mr(expt)	Mr(calc)	ppm	Start - End	Miss	Ions	Peptide
1063.5226	1062.5153	1062.5492	-31.85	26 - 35	0	82	K.TIAQLSSGMR.I
1557.7694	1556.7621	1556.8093	-30.27	1 - 14	0	104	-.MIINNNISALNANR.Q
1834.8276	1833.8203	1833.8891	-37.48	36 - 53	1	157	R.INTAGDDASGLAVSEKMR.S
2207.9109	2206.9036	2206.9888	-38.60	140 - 159	0	117	R.FAADGQTPMTFHIGANMDQR.V
2843.2712	2842.2639	2842.3555	-32.23	65 - 89	0	108	R.NAQNGISFIQTTEGYLNETTNIMQR.M

2. A0A0G4K947 **Mass:** 30002 **Score:** 668 **Expect:** 6.4e-63 **Queries matched:** 5
A0A0G4K947_9SPIR Flagellin OS=Brachyspira suanatina GN=flaB2 PE=3 SV=1

Observed	Mr(expt)	Mr(calc)	ppm	Start - End	Miss	Ions	Peptide
1063.5226	1062.5153	1062.5492	-31.85	26 - 35	0	82	K.TIAQLSSGMR.I
1557.7694	1556.7621	1556.8093	-30.27	1 - 14	0	104	-.MIINNNISALNANR.Q
1834.8276	1833.8203	1833.8891	-37.48	36 - 53	1	157	R.INTAGDDASGLAVSEKMR.S
2207.9109	2206.9036	2206.9888	-38.60	140 - 159	0	117	R.FAADGQTPMTFHIGANMDQR.V
2843.2712	2842.2639	2842.3555	-32.23	65 - 89	0	108	R.NAQNGISFIQTTEGYLNETTNIMQR.M

3. G0EIL9 **Mass:** 30029 **Score:** 668 **Expect:** 6.4e-63 **Queries matched:** 5
G0EIL9_BRAIP Flagellin OS=Brachyspira intermedia (strain ATCC 51140 / PWS/A) GN=flaB3 PE=3 SV=1

Observed	Mr(expt)	Mr(calc)	ppm	Start - End	Miss	Ions	Peptide
1063.5226	1062.5153	1062.5492	-31.85	26 - 35	0	82	K.TIAQLSSGMR.I
1557.7694	1556.7621	1556.8093	-30.27	1 - 14	0	104	-.MIINNNISALNANR.Q
1834.8276	1833.8203	1833.8891	-37.48	36 - 53	1	157	R.INTAGDDASGLAVSEKMR.S
2207.9109	2206.9036	2206.9888	-38.60	140 - 159	0	117	R.FAADGQTPMTFHIGANMDQR.V

2843.2712	2842.2639	2842.3555	-32.23	65 -	89	0	108	R.NAQNGISFIQTTEGYLNETTNIMQR.M

4. Q9F0F6 **Mass:** 30445 **Score:** 668 **Expect:** 6.4e-63 **Queries matched:** 5
Q9F0F6_BRAHO Flagellin OS=Brachyspira hyodysenteriae GN=flaB3 PE=3 SV=1

Observed	Mr(expt)	Mr(calc)	ppm	Start	End	Miss	Ions	Peptide
1063.5226	1062.5153	1062.5492	-31.85	26 -	35	0	82	K.TIAQLSSGMR.I
1557.7694	1556.7621	1556.8093	-30.27	1 -	14	0	104	-.MIINNNISALNANR.Q
1834.8276	1833.8203	1833.8891	-37.48	36 -	53	1	157	R.INTAGDDASGLAVSEKMR.S
2207.9109	2206.9036	2206.9888	-38.60	140 -	159	0	117	R.FAADGQTPMTFHIGANMDQR.V
2843.2712	2842.2639	2842.3555	-32.23	65 -	89	0	108	R.NAQNGISFIQTTEGYLNETTNIMQR.M

5. C0R1D6 **Mass:** 27693 **Score:** 539 **Expect:** 5.1e-50 **Queries matched:** 4
C0R1D6_BRAHW Flagellin OS=Brachyspira hyodysenteriae (strain ATCC 49526 / WA1) GN=flaB3 PE=3 SV=1

Observed	Mr(expt)	Mr(calc)	ppm	Start	End	Miss	Ions	Peptide
1063.5226	1062.5153	1062.5492	-31.85	4 -	13	0	82	K.TIAQLSSGMR.I
1834.8276	1833.8203	1833.8891	-37.48	14 -	31	1	157	R.INTAGDDASGLAVSEKMR.S
2207.9109	2206.9036	2206.9888	-38.60	118 -	137	0	117	R.FAADGQTPMTFHIGANMDQR.V
2843.2712	2842.2639	2842.3555	-32.23	43 -	67	0	108	R.NAQNGISFIQTTEGYLNETTNIMQR.M

No match to: 1557.7694

6. D5U8W2 **Mass:** 29993 **Score:** 524 **Expect:** 1.6e-48 **Queries matched:** 4
D5U8W2_BRAM5 Flagellin OS=Brachyspira murdochii (strain ATCC 51284 / DSM 12563 / 56-150) GN=Bmur_1039 PE=3 SV=1

Observed	Mr(expt)	Mr(calc)	ppm	Start	End	Miss	Ions	Peptide
1063.5226	1062.5153	1062.5492	-31.85	26 -	35	0	82	K.TIAQLSSGMR.I
1557.7694	1556.7621	1556.8093	-30.27	1 -	14	0	104	-.MIINNNISALNANR.Q
1834.8276	1833.8203	1833.8891	-37.48	36 -	53	1	157	R.INTAGDDASGLAVSEKMR.S
2843.2712	2842.2639	2842.3555	-32.23	65 -	89	0	108	R.NAQNGISFIQTTEGYLNETTNIMQR.M

No match to: 2207.9109

7. L0X7D1 **Mass:** 30068 **Score:** 524 **Expect:** 1.6e-48 **Queries matched:** 4
L0X7D1_9SPIR Flagellin OS=Brachyspira hampsonii 30446 GN=A966_03171 PE=3 SV=1

Observed	Mr(expt)	Mr(calc)	ppm	Start	End	Miss	Ions	Peptide
1063.5226	1062.5153	1062.5492	-31.85	26 -	35	0	82	K.TIAQLSSGMR.I
1557.7694	1556.7621	1556.8093	-30.27	1 -	14	0	104	-.MIINNNISALNANR.Q
1834.8276	1833.8203	1833.8891	-37.48	36 -	53	1	157	R.INTAGDDASGLAVSEKMR.S
2843.2712	2842.2639	2842.3555	-32.23	65 -	89	0	108	R.NAQNGISFIQTTEGYLNETTNIMQR.M

No match to: 2207.9109

8. L8XR86 **Mass:** 30027 **Score:** 524 **Expect:** 1.6e-48 **Queries matched:** 4
L8XR86_9SPIR Flagellin OS=Brachyspira hampsonii 30599 GN=H263_04258 PE=3 SV=1

Observed	Mr(expt)	Mr(calc)	ppm	Start	End	Miss	Ions	Peptide
1063.5226	1062.5153	1062.5492	-31.85	26 -	35	0	82	K.TIAQLSSGMR.I
1557.7694	1556.7621	1556.8093	-30.27	1 -	14	0	104	-.MIINNNISALNANR.Q
1834.8276	1833.8203	1833.8891	-37.48	36 -	53	1	157	R.INTAGDDASGLAVSEKMR.S
2843.2712	2842.2639	2842.3555	-32.23	65 -	89	0	108	R.NAQNGISFIQTTEGYLNETTNIMQR.M

No match to: 2207.9109

9. J9UXQ6 **Mass:** 30134 **Score:** 297 **Expect:** 8.1e-26 **Queries matched:** 2
J9UXQ6_BRAPL Flagellin OS=Brachyspira pilosicoli B2904 GN=B2904_orf2664 PE=3 SV=1

Observed	Mr(expt)	Mr(calc)	ppm	Start	End	Miss	Ions	Peptide
1834.8276	1833.8203	1833.8891	-37.48	36 -	53	1	157	R.INTAGDDASGLAVSEKMR.S
2843.2712	2842.2639	2842.3555	-32.23	65 -	89	0	108	R.NAQNGISFIQTTEGYLNETTNIMQR.M

No match to: 1063.5226, 1557.7694, 2207.9109

10. K0JLS4 **Mass:** 30164 **Score:** 297 **Expect:** 8.1e-26 **Queries matched:** 2
K0JLS4_BRAPL Flagellin OS=Brachyspira pilosicoli WesB GN=flaB3 PE=3 SV=1

Observed	Mr(expt)	Mr(calc)	ppm	Start	End	Miss	Ions	Peptide
1834.8276	1833.8203	1833.8891	-37.48	36 -	53	1	157	R.INTAGDDASGLAVSEKMR.S
2843.2712	2842.2639	2842.3555	-32.23	65 -	89	0	108	R.NAQNGISFIQTTEGYLNETTNIMQR.M

No match to: 1063.5226, 1557.7694, 2207.9109

11. D8IDG1 **Mass:** 26584 **Score:** 296 **Expect:** 1e-25 **Queries matched:** 2
D8IDG1_BRAP9 Flagellin OS=Brachyspira pilosicoli (strain ATCC BAA-1826 / 95/1000) GN=flaB3 PE=3 SV=1

Observed	Mr(expt)	Mr(calc)	ppm	Start	End	Miss	Ions	Peptide
1834.8276	1833.8203	1833.8891	-37.48	3 -	20	1	157	R.INTAGDDASGLAVSEKMR.S
2843.2712	2842.2639	2842.3555	-32.23	32 -	56	0	108	R.NAQNGISFIQTTEGYLNETTNIMQR.M

No match to: 1063.5226, 1557.7694, 2207.9109

12. P80161 **Mass:** 2744 **Score:** 126 **Expect:** 1e-08 **Queries matched:** 1
FLAB3_BRAHO Flagellar filament core protein flaB3 (Fragment) OS=Brachyspira hyodysenteriae GN=flaB3 PE=1 SV=1

Observed	Mr(expt)	Mr(calc)	ppm	Start	End	Miss	Ions	Peptide
1557.7694	1556.7621	1556.8093	-30.27	1 -	14	0	104	-.MIINNNISALNANR.Q

No match to: 1063.5226, 1834.8276, 2207.9109, 2843.2712

13. J9UYC2 **Mass:** 31304 **Score:** 82 **Expect:** 0.00027 **Queries matched:** 1
J9UYC2_BRAPL Flagellin OS=Brachyspira pilosicoli B2904 GN=B2904_orf2581 PE=3 SV=1

Observed	Mr(expt)	Mr(calc)	ppm	Start	End	Miss	Ions	Peptide
1557.7694	1556.7621	1556.8093	-30.28	1 -	14	0	69	-.MIINNNISAVNAQR.T

No match to: 1063.5226, 1834.8276, 2207.9109, 2843.2712

14. K0JHQ4 **Mass:** 31288 **Score:** 82 **Expect:** 0.00027 **Queries matched:** 1
K0JHQ4_BRAPL Flagellin OS=Brachyspira pilosicoli WesB GN=flaB2 PE=3 SV=1

Observed	Mr(expt)	Mr(calc)	ppm	Start	End	Miss	Ions	Peptide
1557.7694	1556.7621	1556.8093	-30.28	1 -	14	0	69	-.MIINNNISAVNAQR.T

No match to: 1063.5226, 1834.8276, 2207.9109, 2843.2712

```
15.   L8XVF9    Mass: 33845    Score: 40     Expect: 4.3  Queries matched: 2
      L8XVF9_9SPIR N-acetylneuraminate lyase OS=Brachyspira hampsonii 30599 GN=H263_04848 PE=3 SV=1
       Observed   Mr(expt)   Mr(calc)    ppm  Start   End Miss Ions  Peptide
      1834.8276  1833.8203  1833.8964  -41.50   289 - 304   1  ---   K.IENCAKMIDDAINSIK.-
      2207.9109  2206.9036  2207.0131  -49.61   181 - 201   0   10   K.DVGGDDSIIFNGPDEQFVAGR.L
      No match to: 1063.5226, 1557.7694, 2843.2712
```

Search Parameters

```
Type of search         : Sequence Query
Enzyme                 : Trypsin
Fixed modifications    : Carbamidomethyl (C)
Variable modifications : Oxidation (M)
Mass values            : Monoisotopic
Protein Mass           : Unrestricted
Peptide Mass Tolerance : ± 50 ppm
Fragment Mass Tolerance: ± 0.5 Da
Max Missed Cleavages   : 1
Instrument type        : MALDI-TOF-TOF
Query1 (1063.5226,1+)  : Locus:25.1.1.211172.56636
Query2 (1557.7694,1+)  : Locus:25.1.1.211172.56635
Query3 (1834.8276,1+)  : Locus:25.1.1.211172.56637
Query4 (2207.9109,1+)  : Locus:25.1.1.211172.56638
Query5 (2843.2712,1+)  : Locus:25.1.1.211172.56639
```

Mascot: http://www.matrixscience.com/

Mascot Search Results

User :
Email :
Search title : **F:\ROD_inmunoproteoma\MALDI\ppw_2016_562_563_ROD\ppw_I13_145397050229.txt**
MS data file : **F:\ROD_inmunoproteoma\MALDI\ppw_2016_562_563_ROD\ppw_I13_145397050229.txt**
Database : **Brachyspiraceae Brachyspiraceae_20160127-151122 (40573 sequences; 13470793 residues)**
Timestamp : **23 Feb 2016 at 16:06:16 GMT**
Warning : **A Peptide summary report will usually give a much clearer picture of MS/MS search results.**
Top Score : **757 for A0A0H0USH8, A0A0H0USH8_BRAHO Flagellin OS=Brachyspira hyodysenteriae GN=SR30_01525 PE=3 SV=1**

Probability Based Mowse Score

Protein score is -10*Log(P), where P is the probability that the observed match is a random event.
Protein scores greater than 59 are significant (p<0.05).
Protein scores are derived from ions scores as a non-probabilistic basis for ranking protein hits.

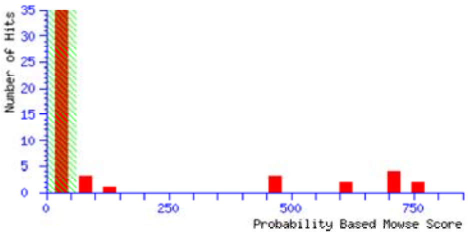

Protein Summary Report

Format As | Protein Summary (deprecated) | Help

Significance threshold p< 0.05 | Max. number of hits AUTO

Standard scoring ◉ MudPIT scoring ○ | Ions score or expect cut-off 0 | Show sub-sets 0

Show pop-ups ◉ Suppress pop-ups ○ | Sort unassigned Decreasing Score | Require bold red ☐

Re-Search All | Search Unmatched

Index

	Accession	Mass	Score	Description
1.	A0A0H0USH8	30059	757	A0A0H0USH8_BRAHO Flagellin OS=Brachyspira hyodysenteriae GN=SR30_01525 PE=3 SV=1
2.	Q9F0F6	30445	757	Q9F0F6_BRAHO Flagellin OS=Brachyspira hyodysenteriae GN=flaB3 PE=3 SV=1
3.	A0A0G4K947	30002	696	A0A0G4K947_9SPIR Flagellin OS=Brachyspira suanatina GN=flaB2 PE=3 SV=1
4.	G0EIL9	30029	696	G0EIL9_BRAIP Flagellin OS=Brachyspira intermedia (strain ATCC 51140 / PWS/A) GN=flaB3 PE=3 SV=1
5.	L0X7D1	30068	696	L0X7D1_9SPIR Flagellin OS=Brachyspira hampsonii 30446 GN=A966_03171 PE=3 SV=1
6.	L8XR86	30027	696	L8XR86_9SPIR Flagellin OS=Brachyspira hampsonii 30599 GN=H263_04258 PE=3 SV=1
7.	C0R1D6	27693	628	C0R1D6_BRAHW Flagellin OS=Brachyspira hyodysenteriae (strain ATCC 49526 / WA1) GN=flaB3 PE=3 SV=1
8.	D5U8W2	29993	626	D5U8W2_BRAM5 Flagellin OS=Brachyspira murdochii (strain ATCC 51284 / DSM 12563 / 56-150) GN=Bmur_1039 PE=3 SV=
9.	J9UXQ6	30134	457	J9UXQ6_BRAPL Flagellin OS=Brachyspira pilosicoli B2904 GN=B2904_orf2664 PE=3 SV=1
10.	K0JLS4	30164	457	K0JLS4_BRAPL Flagellin OS=Brachyspira pilosicoli WesB GN=flaB3 PE=3 SV=1
11.	D8IDG1	26584	457	D8IDG1_BRAP9 Flagellin OS=Brachyspira pilosicoli (strain ATCC BAA-1826 / 95/1000) GN=flaB3 PE=3 SV=1
12.	P80161	2744	126	FLAB3_BRAHO Flagellar filament core protein flaB3 (Fragment) OS=Brachyspira hyodysenteriae GN=flaB3 PE=1 SV=1
13.	J9UYC2	31304	82	J9UYC2_BRAPL Flagellin OS=Brachyspira pilosicoli B2904 GN=B2904_orf2581 PE=3 SV=1
14.	K0JHQ4	31288	82	K0JHQ4_BRAPL Flagellin OS=Brachyspira pilosicoli WesB GN=flaB2 PE=3 SV=1
15.	R5LA18	30135	57	R5LA18_9SPIR Flagellin OS=Brachyspira sp. CAG:700 GN=BN758_01499 PE=3 SV=1

Results List

1. A0A0H0USH8 **Mass:** 30059 **Score:** 757 **Expect:** 8.1e-72 **Queries matched:** 5
A0A0H0USH8_BRAHO Flagellin OS=Brachyspira hyodysenteriae GN=SR30_01525 PE=3 SV=1

Observed	Mr(expt)	Mr(calc)	ppm	Start		End	Miss	Ions	Peptide
1063.5244	1062.5171	1062.5492	-30.15	26	-	35	0	88	K.TIAQLSSGMR.I
1374.6538	1373.6465	1373.6973	-36.96	195	-	206	0	106	R.IDEGIQMVVSQR.A
1557.7740	1556.7667	1556.8093	-27.32	1	-	14	0	104	-.MIINNNISALNANR.Q
1834.8325	1833.8252	1833.8891	-34.81	36	-	53	1	166	R.INTAGDDASGLAVSEKMR.S
2843.2639	2842.2566	2842.3555	-34.80	65	-	89	0	195	R.NAQNGISFIQTTEGYLNETTNIMQR.M

2. Q9F0F6 **Mass:** 30445 **Score:** 757 **Expect:** 8.1e-72 **Queries matched:** 5
Q9F0F6_BRAHO Flagellin OS=Brachyspira hyodysenteriae GN=flaB3 PE=3 SV=1

Observed	Mr(expt)	Mr(calc)	ppm	Start		End	Miss	Ions	Peptide
1063.5244	1062.5171	1062.5492	-30.15	26	-	35	0	88	K.TIAQLSSGMR.I
1374.6538	1373.6465	1373.6973	-36.96	195	-	206	0	106	R.IDEGIQMVVSQR.A
1557.7740	1556.7667	1556.8093	-27.32	1	-	14	0	104	-.MIINNNISALNANR.Q
1834.8325	1833.8252	1833.8891	-34.81	36	-	53	1	166	R.INTAGDDASGLAVSEKMR.S
2843.2639	2842.2566	2842.3555	-34.80	65	-	89	0	195	R.NAQNGISFIQTTEGYLNETTNIMQR.M

3. A0A0G4K947 **Mass:** 30002 **Score:** 696 **Expect:** 1e-65 **Queries matched:** 5
A0A0G4K947_9SPIR Flagellin OS=Brachyspira suanatina GN=flaB2 PE=3 SV=1

Observed	Mr(expt)	Mr(calc)	ppm	Start		End	Miss	Ions	Peptide
1063.5244	1062.5171	1062.5492	-30.15	26	-	35	0	88	K.TIAQLSSGMR.I
1374.6538	1373.6465	1373.6973	-36.96	195	-	206	0	45	R.IDEGIQMVVAQR.A + Oxidation (M)
1557.7740	1556.7667	1556.8093	-27.32	1	-	14	0	104	-.MIINNNISALNANR.Q
1834.8325	1833.8252	1833.8891	-34.81	36	-	53	1	166	R.INTAGDDASGLAVSEKMR.S

2843.2639	2842.2566	2842.3555	-34.80	65 -	89	0	195	R.NAQNGISFIQTTEGYLNETTNIMQR.M

4. G0EIL9 **Mass:** 30029 **Score:** 696 **Expect:** 1e-65 **Queries matched:** 5
G0EIL9_BRAIP Flagellin OS=Brachyspira intermedia (strain ATCC 51140 / PWS/A) GN=flaB3 PE=3 SV=1

Observed	Mr(expt)	Mr(calc)	ppm	Start	End	Miss	Ions	Peptide
1063.5244	1062.5171	1062.5492	-30.15	26 -	35	0	88	K.TIAQLSSGMR.I
1374.6538	1373.6465	1373.6973	-36.96	195 -	206	0	45	R.IDEGIQMVVAQR.A + Oxidation (M)
1557.7740	1556.7667	1556.8093	-27.32	1 -	14	0	104	-.MIINNNISALNANR.Q
1834.8325	1833.8252	1833.8891	-34.81	36 -	53	1	166	R.INTAGDDASGLAVSEKMR.S
2843.2639	2842.2566	2842.3555	-34.80	65 -	89	0	195	R.NAQNGISFIQTTEGYLNETTNIMQR.M

5. L0X7D1 **Mass:** 30068 **Score:** 696 **Expect:** 1e-65 **Queries matched:** 5
L0X7D1_9SPIR Flagellin OS=Brachyspira hampsonii 30446 GN=A966_03171 PE=3 SV=1

Observed	Mr(expt)	Mr(calc)	ppm	Start	End	Miss	Ions	Peptide
1063.5244	1062.5171	1062.5492	-30.15	26 -	35	0	88	K.TIAQLSSGMR.I
1374.6538	1373.6465	1373.6973	-36.96	195 -	206	0	45	R.IDEGIQMVVAQR.A + Oxidation (M)
1557.7740	1556.7667	1556.8093	-27.32	1 -	14	0	104	-.MIINNNISALNANR.Q
1834.8325	1833.8252	1833.8891	-34.81	36 -	53	1	166	R.INTAGDDASGLAVSEKMR.S
2843.2639	2842.2566	2842.3555	-34.80	65 -	89	0	195	R.NAQNGISFIQTTEGYLNETTNIMQR.M

6. L8XR86 **Mass:** 30027 **Score:** 696 **Expect:** 1e-65 **Queries matched:** 5
L8XR86_9SPIR Flagellin OS=Brachyspira hampsonii 30599 GN=H263_04258 PE=3 SV=1

Observed	Mr(expt)	Mr(calc)	ppm	Start	End	Miss	Ions	Peptide
1063.5244	1062.5171	1062.5492	-30.15	26 -	35	0	88	K.TIAQLSSGMR.I
1374.6538	1373.6465	1373.6973	-36.96	195 -	206	0	45	R.IDEGIQMVVAQR.A + Oxidation (M)
1557.7740	1556.7667	1556.8093	-27.32	1 -	14	0	104	-.MIINNNISALNANR.Q
1834.8325	1833.8252	1833.8891	-34.81	36 -	53	1	166	R.INTAGDDASGLAVSEKMR.S
2843.2639	2842.2566	2842.3555	-34.80	65 -	89	0	195	R.NAQNGISFIQTTEGYLNETTNIMQR.M

7. C0R1D6 **Mass:** 27693 **Score:** 628 **Expect:** 6.4e-59 **Queries matched:** 4
C0R1D6_BRAHW Flagellin OS=Brachyspira hyodysenteriae (strain ATCC 49526 / WA1) GN=flaB3 PE=3 SV=1

Observed	Mr(expt)	Mr(calc)	ppm	Start	End	Miss	Ions	Peptide
1063.5244	1062.5171	1062.5492	-30.15	4 -	13	0	88	K.TIAQLSSGMR.I
1374.6538	1373.6465	1373.6973	-36.96	173 -	184	0	106	R.IDEGIQMVVSQR.A
1834.8325	1833.8252	1833.8891	-34.81	14 -	31	1	166	R.INTAGDDASGLAVSEKMR.S
2843.2639	2842.2566	2842.3555	-34.80	43 -	67	0	195	R.NAQNGISFIQTTEGYLNETTNIMQR.M

No match to: 1557.7740

8. D5U8W2 **Mass:** 29993 **Score:** 626 **Expect:** 1e-58 **Queries matched:** 4
D5U8W2_BRAM5 Flagellin OS=Brachyspira murdochii (strain ATCC 51284 / DSM 12563 / 56-150) GN=Bmur_1039 PE=3 SV=1

Observed	Mr(expt)	Mr(calc)	ppm	Start	End	Miss	Ions	Peptide
1063.5244	1062.5171	1062.5492	-30.15	26 -	35	0	88	K.TIAQLSSGMR.I
1557.7740	1556.7667	1556.8093	-27.32	1 -	14	0	104	-.MIINNNISALNANR.Q
1834.8325	1833.8252	1833.8891	-34.81	36 -	53	1	166	R.INTAGDDASGLAVSEKMR.S
2843.2639	2842.2566	2842.3555	-34.80	65 -	89	0	195	R.NAQNGISFIQTTEGYLNETTNIMQR.M

No match to: 1374.6538

9. J9UXQ6 **Mass:** 30134 **Score:** 457 **Expect:** 8.1e-42 **Queries matched:** 3
J9UXQ6_BRAPL Flagellin OS=Brachyspira pilosicoli B2904 GN=B2904_orf2664 PE=3 SV=1

Observed	Mr(expt)	Mr(calc)	ppm	Start	End	Miss	Ions	Peptide
1374.6538	1373.6465	1373.6973	-36.96	195 -	206	0	45	R.IDEGIQMVVAQR.A + Oxidation (M)
1834.8325	1833.8252	1833.8891	-34.81	36 -	53	1	166	R.INTAGDDASGLAVSEKMR.S
2843.2639	2842.2566	2842.3555	-34.80	65 -	89	0	195	R.NAQNGISFIQTTEGYLNETTNIMQR.M

No match to: 1063.5244, 1557.7740

10. K0JLS4 **Mass:** 30164 **Score:** 457 **Expect:** 8.1e-42 **Queries matched:** 3
K0JLS4_BRAPL Flagellin OS=Brachyspira pilosicoli WesB GN=flaB3 PE=3 SV=1

Observed	Mr(expt)	Mr(calc)	ppm	Start	End	Miss	Ions	Peptide
1374.6538	1373.6465	1373.6973	-36.96	195 -	206	0	45	R.IDEGIQMVVAQR.A + Oxidation (M)
1834.8325	1833.8252	1833.8891	-34.81	36 -	53	1	166	R.INTAGDDASGLAVSEKMR.S
2843.2639	2842.2566	2842.3555	-34.80	65 -	89	0	195	R.NAQNGISFIQTTEGYLNETTNIMQR.M

No match to: 1063.5244, 1557.7740

11. D8IDG1 **Mass:** 26584 **Score:** 457 **Expect:** 8.1e-42 **Queries matched:** 3
D8IDG1_BRAP9 Flagellin OS=Brachyspira pilosicoli (strain ATCC BAA-1826 / 95/1000) GN=flaB3 PE=3 SV=1

Observed	Mr(expt)	Mr(calc)	ppm	Start	End	Miss	Ions	Peptide
1374.6538	1373.6465	1373.6973	-36.96	162 -	173	0	45	R.IDEGIQMVVAQR.A + Oxidation (M)
1834.8325	1833.8252	1833.8891	-34.81	3 -	20	1	166	R.INTAGDDASGLAVSEKMR.S
2843.2639	2842.2566	2842.3555	-34.80	32 -	56	0	195	R.NAQNGISFIQTTEGYLNETTNIMQR.M

No match to: 1063.5244, 1557.7740

12. P80161 **Mass:** 2744 **Score:** 126 **Expect:** 1e-08 **Queries matched:** 1
FLAB3_BRAHO Flagellar filament core protein flaB3 (Fragment) OS=Brachyspira hyodysenteriae GN=flaB3 PE=1 SV=1

Observed	Mr(expt)	Mr(calc)	ppm	Start	End	Miss	Ions	Peptide
1557.7740	1556.7667	1556.8093	-27.32	1 -	14	0	104	-.MIINNNISALNANR.Q

No match to: 1063.5244, 1374.6538, 1834.8325, 2843.2639

13. J9UYC2 **Mass:** 31304 **Score:** 82 **Expect:** 0.00029 **Queries matched:** 1
J9UYC2_BRAPL Flagellin OS=Brachyspira pilosicoli B2904 GN=B2904_orf2581 PE=3 SV=1

Observed	Mr(expt)	Mr(calc)	ppm	Start	End	Miss	Ions	Peptide
1557.7740	1556.7667	1556.8093	-27.33	1 -	14	0	69	-.MIINNNISAVNAQR.T

No match to: 1063.5244, 1374.6538, 1834.8325, 2843.2639

14. K0JHQ4 **Mass:** 31288 **Score:** 82 **Expect:** 0.00029 **Queries matched:** 1
K0JHQ4_BRAPL Flagellin OS=Brachyspira pilosicoli WesB GN=flaB2 PE=3 SV=1

Observed	Mr(expt)	Mr(calc)	ppm	Start	End	Miss	Ions	Peptide

```
1557.7740  1556.7667  1556.8093  -27.33     1 -  14   0   69   -.MIINNNISAVNAQR.T
```
No match to: 1063.5244, 1374.6538, 1834.8325, 2843.2639

15. R5LA18 **Mass:** 30135 **Score:** 57 **Expect:** 0.085 **Queries matched:** 1

R5LA18_9SPIR Flagellin OS=Brachyspira sp. CAG:700 GN=BN758_01499 PE=3 SV=1

Observed	Mr(expt)	Mr(calc)	ppm	Start	End	Miss	Ions	Peptide
1374.6538	1373.6465	1373.6973	-36.96	195 -	206	0	45	R.IDEGIQMVVAQR.A + Oxidation (M)

No match to: 1063.5244, 1557.7740, 1834.8325, 2843.2639

Search Parameters

```
Type of search         : Sequence Query
Enzyme                 : Trypsin
Fixed modifications    : Carbamidomethyl (C)
Variable modifications : Oxidation (M)
Mass values            : Monoisotopic
Protein Mass           : Unrestricted
Peptide Mass Tolerance : ± 50 ppm
Fragment Mass Tolerance: ± 0.5 Da
Max Missed Cleavages   : 1
Instrument type        : MALDI-TOF-TOF
Query1 (1063.5244,1+)  : Locus:30.1.1.211177.56663
Query2 (1374.6538,1+)  : Locus:30.1.1.211177.56664
Query3 (1557.7740,1+)  : Locus:30.1.1.211177.56660
Query4 (1834.8325,1+)  : Locus:30.1.1.211177.56662
Query5 (2843.2639,1+)  : Locus:30.1.1.211177.56661
```

Mascot: http://www.matrixscience.com/

MATRIX SCIENCE Mascot Search Results

User :
Email :
Search title : **F:\ROD_inmunoproteoma\MALDI\ppw_2016_562_563_ROD\ppw_I20_145397050836.txt**
MS data file : **F:\ROD_inmunoproteoma\MALDI\ppw_2016_562_563_ROD\ppw_I20_145397050836.txt**
Database : **Brachyspiraceae Brachyspiraceae_20160127-151122 (40573 sequences; 13470793 residues)**
Timestamp : **23 Feb 2016 at 16:06:34 GMT**
Warning : **A Peptide summary report will usually give a much clearer picture of MS/MS search results.**
Top Score : **341 for A0A0H0USH8, A0A0H0USH8_BRAHO Flagellin OS=Brachyspira hyodysenteriae GN=SR30_01525 PE=3 SV=1**

Probability Based Mowse Score

Protein score is -10*Log(P), where P is the probability that the observed match is a random event.
Protein scores greater than 59 are significant (p<0.05).
Protein scores are derived from ions scores as a non-probabilistic basis for ranking protein hits.

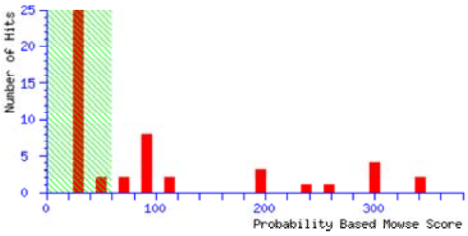

Protein Summary Report

Format As | Protein Summary (deprecated) | Help

Significance threshold p< 0.05 | Max. number of hits AUTO

Standard scoring ◉ MudPIT scoring ○ | Ions score or expect cut-off 0 | Show sub-sets 0

Show pop-ups ◉ Suppress pop-ups ○ | Sort unassigned Decreasing Score | Require bold red ☐

Re-Search All | Search Unmatched

Index

	Accession	Mass	Score	Description
1.	A0A0H0USH8	30059	341	A0A0H0USH8_BRAHO Flagellin OS=Brachyspira hyodysenteriae GN=SR30_01525 PE=3 SV=1
2.	Q9F0F6	30445	341	Q9F0F6_BRAHO Flagellin OS=Brachyspira hyodysenteriae GN=flaB3 PE=3 SV=1
3.	A0A0G4K947	30002	302	A0A0G4K947_9SPIR Flagellin OS=Brachyspira suanatina GN=flaB2 PE=3 SV=1
4.	G0EIL9	30029	302	G0EIL9_BRAIP Flagellin OS=Brachyspira intermedia (strain ATCC 51140 / PWS/A) GN=flaB3 PE=3 SV=1
5.	L0X7D1	30068	302	L0X7D1_9SPIR Flagellin OS=Brachyspira hampsonii 30446 GN=A966_03171 PE=3 SV=1
6.	L8XR86	30027	302	L8XR86_9SPIR Flagellin OS=Brachyspira hampsonii 30599 GN=H263_04258 PE=3 SV=1
7.	D5U8W2	29993	252	D5U8W2_BRAM5 Flagellin OS=Brachyspira murdochii (strain ATCC 51284 / DSM 12563 / 56-150) GN=Bmur_1039 PE=3 SV=
8.	C0R1D6	27693	238	C0R1D6_BRAHW Flagellin OS=Brachyspira hyodysenteriae (strain ATCC 49526 / WA1) GN=flaB3 PE=3 SV=1
9.	J9UXQ6	30134	198	J9UXQ6_BRAPL Flagellin OS=Brachyspira pilosicoli B2904 GN=B2904_orf2664 PE=3 SV=1
10.	K0JLS4	30164	198	K0JLS4_BRAPL Flagellin OS=Brachyspira pilosicoli WesB GN=flaB3 PE=3 SV=1
11.	D8IDG1	26584	198	D8IDG1_BRAP9 Flagellin OS=Brachyspira pilosicoli (strain ATCC BAA-1826 / 95/1000) GN=flaB3 PE=3 SV=1
12.	P80161	2744	104	FLAB3_BRAHO Flagellar filament core protein flaB3 (Fragment) OS=Brachyspira hyodysenteriae GN=flaB3 PE=1 SV=1
13.	Q7M132	2814	103	Q7M132_BRAHO Flagellar core protein, 34K (Fragment) OS=Brachyspira hyodysenteriae PE=1 SV=1
14.	R5LA18	30135	100	R5LA18_9SPIR Flagellin OS=Brachyspira sp. CAG:700 GN=BN758_01499 PE=3 SV=1
15.	P80160	31113	93	FLAB2_BRAHO Flagellar filament core protein flaB2 OS=Brachyspira hyodysenteriae GN=flaB2 PE=1 SV=1
16.	A0A0H0X544	31129	93	A0A0H0X544_BRAHO Flagellin OS=Brachyspira hyodysenteriae GN=SU43_01870 PE=3 SV=1
17.	A0A0G4K9G4	31184	93	A0A0G4K9G4_9SPIR Flagellin OS=Brachyspira suanatina GN=flaB2 PE=3 SV=1
18.	A0A0H0ULZ7	31057	93	A0A0H0ULZ7_BRAHO Flagellin OS=Brachyspira hyodysenteriae GN=SR30_04500 PE=3 SV=1
19.	D5UB01	31218	93	D5UB01_BRAM5 Flagellin OS=Brachyspira murdochii (strain ATCC 51284 / DSM 12563 / 56-150) GN=Bmur_1792 PE=3 SV=
20.	L0X2I6	31181	93	L0X2I6_9SPIR Flagellin OS=Brachyspira hampsonii 30446 GN=A966_05778 PE=3 SV=1
21.	L8XTT2	31194	93	L8XTT2_9SPIR Flagellin OS=Brachyspira hampsonii 30599 GN=H263_08389 PE=3 SV=1
22.	J9UYC2	31304	64	J9UYC2_BRAPL Flagellin OS=Brachyspira pilosicoli B2904 GN=B2904_orf2581 PE=3 SV=1
23.	K0JHQ4	31288	64	K0JHQ4_BRAPL Flagellin OS=Brachyspira pilosicoli WesB GN=flaB2 PE=3 SV=1
24.	G0ENJ8	83561	43	G0ENJ8_BRAIP Aconitate hydratase OS=Brachyspira intermedia (strain ATCC 51140 / PWS/A) GN=Bint_1258 PE=4 SV=1

Results List

1. A0A0H0USH8 **Mass:** 30059 **Score:** 341 **Expect:** 3.2e-30 **Queries matched:** 4
A0A0H0USH8_BRAHO Flagellin OS=Brachyspira hyodysenteriae GN=SR30_01525 PE=3 SV=1

Observed	Mr(expt)	Mr(calc)	ppm	Start	End	Miss	Ions	Peptide
1307.6259	1306.6186	1306.6742	-42.52	54 -	64	1	44	R.SQYRGLQQATR.N
1374.6479	1373.6406	1373.6973	-41.26	195 -	206	0	67	R.IDEGIQMVVSQR.A
1557.7516	1556.7443	1556.8093	-41.70	1 -	14	0	82	-.MIINNNISALNANR.Q
1834.8167	1833.8094	1833.8891	-43.42	36 -	53	1	78	R.INTAGDDASGLAVSEKMR.S

No match to: 1571.7649

2. Q9F0F6 **Mass:** 30445 **Score:** 341 **Expect:** 3.2e-30 **Queries matched:** 4
Q9F0F6_BRAHO Flagellin OS=Brachyspira hyodysenteriae GN=flaB3 PE=3 SV=1

Observed	Mr(expt)	Mr(calc)	ppm	Start	End	Miss	Ions	Peptide
1307.6259	1306.6186	1306.6742	-42.52	54 -	64	1	44	R.SQYRGLQQATR.N
1374.6479	1373.6406	1373.6973	-41.26	195 -	206	0	67	R.IDEGIQMVVSQR.A
1557.7516	1556.7443	1556.8093	-41.70	1 -	14	0	82	-.MIINNNISALNANR.Q
1834.8167	1833.8094	1833.8891	-43.42	36 -	53	1	78	R.INTAGDDASGLAVSEKMR.S

No match to: 1571.7649

3. A0A0G4K947 **Mass:** 30002 **Score:** 302 **Expect:** 2.6e-26 **Queries matched:** 4
A0A0G4K947_9SPIR Flagellin OS=Brachyspira suanatina GN=flaB2 PE=3 SV=1

Observed	Mr(expt)	Mr(calc)	ppm	Start	End	Miss	Ions	Peptide
1307.6259	1306.6186	1306.6742	-42.52	54	64	1	44	R.SQYRGLQQATR.N
1374.6479	1373.6406	1373.6973	-41.26	195	206	0	28	R.IDEGIQMVVAQR.A + Oxidation (M)
1557.7516	1556.7443	1556.8093	-41.70	1	14	0	82	-.MIINNNISALNANR.Q
1834.8167	1833.8094	1833.8891	-43.42	36	53	1	78	R.INTAGDDASGLAVSEKMR.S

No match to: 1571.7649

4. G0EIL9 **Mass:** 30029 **Score:** 302 **Expect:** 2.6e-26 **Queries matched:** 4
G0EIL9_BRAIP Flagellin OS=Brachyspira intermedia (strain ATCC 51140 / PWS/A) GN=flaB3 PE=3 SV=1

Observed	Mr(expt)	Mr(calc)	ppm	Start	End	Miss	Ions	Peptide
1307.6259	1306.6186	1306.6742	-42.52	54	64	1	44	R.SQYRGLQQATR.N
1374.6479	1373.6406	1373.6973	-41.26	195	206	0	28	R.IDEGIQMVVAQR.A + Oxidation (M)
1557.7516	1556.7443	1556.8093	-41.70	1	14	0	82	-.MIINNNISALNANR.Q
1834.8167	1833.8094	1833.8891	-43.42	36	53	1	78	R.INTAGDDASGLAVSEKMR.S

No match to: 1571.7649

5. L0X7D1 **Mass:** 30068 **Score:** 302 **Expect:** 2.6e-26 **Queries matched:** 4
L0X7D1_9SPIR Flagellin OS=Brachyspira hampsonii 30446 GN=A966_03171 PE=3 SV=1

Observed	Mr(expt)	Mr(calc)	ppm	Start	End	Miss	Ions	Peptide
1307.6259	1306.6186	1306.6742	-42.52	54	64	1	44	R.SQYRGLQQATR.N
1374.6479	1373.6406	1373.6973	-41.26	195	206	0	28	R.IDEGIQMVVAQR.A + Oxidation (M)
1557.7516	1556.7443	1556.8093	-41.70	1	14	0	82	-.MIINNNISALNANR.Q
1834.8167	1833.8094	1833.8891	-43.42	36	53	1	78	R.INTAGDDASGLAVSEKMR.S

No match to: 1571.7649

6. L8XR86 **Mass:** 30027 **Score:** 302 **Expect:** 2.6e-26 **Queries matched:** 4
L8XR86_9SPIR Flagellin OS=Brachyspira hampsonii 30599 GN=H263_04258 PE=3 SV=1

Observed	Mr(expt)	Mr(calc)	ppm	Start	End	Miss	Ions	Peptide
1307.6259	1306.6186	1306.6742	-42.52	54	64	1	44	R.SQYRGLQQATR.N
1374.6479	1373.6406	1373.6973	-41.26	195	206	0	28	R.IDEGIQMVVAQR.A + Oxidation (M)
1557.7516	1556.7443	1556.8093	-41.70	1	14	0	82	-.MIINNNISALNANR.Q
1834.8167	1833.8094	1833.8891	-43.42	36	53	1	78	R.INTAGDDASGLAVSEKMR.S

No match to: 1571.7649

7. D5U8W2 **Mass:** 29993 **Score:** 252 **Expect:** 2.6e-21 **Queries matched:** 3
D5U8W2_BRAM5 Flagellin OS=Brachyspira murdochii (strain ATCC 51284 / DSM 12563 / 56-150) GN=Bmur_1039 PE=3 SV=1

Observed	Mr(expt)	Mr(calc)	ppm	Start	End	Miss	Ions	Peptide
1307.6259	1306.6186	1306.6742	-42.52	54	64	1	44	R.SQYRGLQQATR.N
1557.7516	1556.7443	1556.8093	-41.70	1	14	0	82	-.MIINNNISALNANR.Q
1834.8167	1833.8094	1833.8891	-43.42	36	53	1	78	R.INTAGDDASGLAVSEKMR.S

No match to: 1374.6479, 1571.7649

8. C0R1D6 **Mass:** 27693 **Score:** 238 **Expect:** 6.4e-20 **Queries matched:** 3
C0R1D6_BRAHW Flagellin OS=Brachyspira hyodysenteriae (strain ATCC 49526 / WA1) GN=flaB3 PE=3 SV=1

Observed	Mr(expt)	Mr(calc)	ppm	Start	End	Miss	Ions	Peptide
1307.6259	1306.6186	1306.6742	-42.52	32	42	1	44	R.SQYRGLQQATR.N
1374.6479	1373.6406	1373.6973	-41.26	173	184	0	67	R.IDEGIQMVVSQR.A
1834.8167	1833.8094	1833.8891	-43.42	14	31	1	78	R.INTAGDDASGLAVSEKMR.S

No match to: 1557.7516, 1571.7649

9. J9UXQ6 **Mass:** 30134 **Score:** 198 **Expect:** 6.4e-16 **Queries matched:** 3
J9UXQ6_BRAPL Flagellin OS=Brachyspira pilosicoli B2904 GN=B2904_orf2664 PE=3 SV=1

Observed	Mr(expt)	Mr(calc)	ppm	Start	End	Miss	Ions	Peptide
1307.6259	1306.6186	1306.6742	-42.52	54	64	1	44	R.SQYRGLQQATR.N
1374.6479	1373.6406	1373.6973	-41.26	195	206	0	28	R.IDEGIQMVVAQR.A + Oxidation (M)
1834.8167	1833.8094	1833.8891	-43.42	36	53	1	78	R.INTAGDDASGLAVSEKMR.S

No match to: 1557.7516, 1571.7649

10. K0JLS4 **Mass:** 30164 **Score:** 198 **Expect:** 6.4e-16 **Queries matched:** 3
K0JLS4_BRAPL Flagellin OS=Brachyspira pilosicoli WesB GN=flaB3 PE=3 SV=1

Observed	Mr(expt)	Mr(calc)	ppm	Start	End	Miss	Ions	Peptide
1307.6259	1306.6186	1306.6742	-42.52	54	64	1	44	R.SQYRGLQQATR.N
1374.6479	1373.6406	1373.6973	-41.26	195	206	0	28	R.IDEGIQMVVAQR.A + Oxidation (M)
1834.8167	1833.8094	1833.8891	-43.42	36	53	1	78	R.INTAGDDASGLAVSEKMR.S

No match to: 1557.7516, 1571.7649

11. D8IDG1 **Mass:** 26584 **Score:** 198 **Expect:** 6.4e-16 **Queries matched:** 3
D8IDG1_BRAP9 Flagellin OS=Brachyspira pilosicoli (strain ATCC BAA-1826 / 95/1000) GN=flaB3 PE=3 SV=1

Observed	Mr(expt)	Mr(calc)	ppm	Start	End	Miss	Ions	Peptide
1307.6259	1306.6186	1306.6742	-42.52	21	31	1	44	R.SQYRGLQQATR.N
1374.6479	1373.6406	1373.6973	-41.26	162	173	0	28	R.IDEGIQMVVAQR.A + Oxidation (M)
1834.8167	1833.8094	1833.8891	-43.42	3	20	1	78	R.INTAGDDASGLAVSEKMR.S

No match to: 1557.7516, 1571.7649

12. P80161 **Mass:** 2744 **Score:** 104 **Expect:** 1.6e-06 **Queries matched:** 1
FLAB3_BRAHO Flagellar filament core protein flaB3 (Fragment) OS=Brachyspira hyodysenteriae GN=flaB3 PE=1 SV=1

Observed	Mr(expt)	Mr(calc)	ppm	Start	End	Miss	Ions	Peptide
1557.7516	1556.7443	1556.8093	-41.70	1	14	0	82	-.MIINNNISALNANR.Q

No match to: 1307.6259, 1374.6479, 1571.7649, 1834.8167

13. Q7M132 **Mass:** 2814 **Score:** 103 **Expect:** 2e-06 **Queries matched:** 1
Q7M132_BRAHO Flagellar core protein, 34K (Fragment) OS=Brachyspira hyodysenteriae PE=1 SV=1

Observed	Mr(expt)	Mr(calc)	ppm	Start	End	Miss	Ions	Peptide
1571.7649	1570.7576	1570.8249	-42.83	1	14	0	80	-.MIINNNISAINAQR.T

No match to: 1307.6259, 1374.6479, 1557.7516, 1834.8167

14. R5LA18 **Mass:** 30135 **Score:** 100 **Expect:** 4.3e-06 **Queries matched:** 2
R5LA18_9SPIR Flagellin OS=Brachyspira sp. CAG:700 GN=BN758_01499 PE=3 SV=1

Observed	Mr(expt)	Mr(calc)	ppm	Start - End	Miss	Ions	Peptide
1307.6259	1306.6186	1306.6742	-42.52	54 - 64	1	44	R.SQYRGLQQATR.N
1374.6479	1373.6406	1373.6973	-41.26	195 - 206	0	28	R.IDEGIQMVVAQR.A + Oxidation (M)

No match to: 1557.7516, 1571.7649, 1834.8167

15. P80160 **Mass:** 31113 **Score:** 93 **Expect:** 2.1e-05 **Queries matched:** 1
FLAB2_BRAHO Flagellar filament core protein flaB2 OS=Brachyspira hyodysenteriae GN=flaB2 PE=1 SV=1

Observed	Mr(expt)	Mr(calc)	ppm	Start - End	Miss	Ions	Peptide
1571.7649	1570.7576	1570.8249	-42.83	1 - 14	0	80	-.MIINNNISAINAQR.T

No match to: 1307.6259, 1374.6479, 1557.7516, 1834.8167

16. A0A0H0X544 **Mass:** 31129 **Score:** 93 **Expect:** 2.1e-05 **Queries matched:** 1
A0A0H0X544_BRAHO Flagellin OS=Brachyspira hyodysenteriae GN=SU43_01870 PE=3 SV=1

Observed	Mr(expt)	Mr(calc)	ppm	Start - End	Miss	Ions	Peptide
1571.7649	1570.7576	1570.8249	-42.83	1 - 14	0	80	-.MIINNNISAINAQR.T

No match to: 1307.6259, 1374.6479, 1557.7516, 1834.8167

17. A0A0G4K9G4 **Mass:** 31184 **Score:** 93 **Expect:** 2.1e-05 **Queries matched:** 1
A0A0G4K9G4_9SPIR Flagellin OS=Brachyspira suanatina GN=flaB2 PE=3 SV=1

Observed	Mr(expt)	Mr(calc)	ppm	Start - End	Miss	Ions	Peptide
1571.7649	1570.7576	1570.8249	-42.83	1 - 14	0	80	-.MIINNNISAINAQR.T

No match to: 1307.6259, 1374.6479, 1557.7516, 1834.8167

18. A0A0H0ULZ7 **Mass:** 31057 **Score:** 93 **Expect:** 2.1e-05 **Queries matched:** 1
A0A0H0ULZ7_BRAHO Flagellin OS=Brachyspira hyodysenteriae GN=SR30_04500 PE=3 SV=1

Observed	Mr(expt)	Mr(calc)	ppm	Start - End	Miss	Ions	Peptide
1571.7649	1570.7576	1570.8249	-42.83	1 - 14	0	80	-.MIINNNISAINAQR.T

No match to: 1307.6259, 1374.6479, 1557.7516, 1834.8167

19. D5UB01 **Mass:** 31218 **Score:** 93 **Expect:** 2.1e-05 **Queries matched:** 1
D5UB01_BRAM5 Flagellin OS=Brachyspira murdochii (strain ATCC 51284 / DSM 12563 / 56-150) GN=Bmur_1792 PE=3 SV=1

Observed	Mr(expt)	Mr(calc)	ppm	Start - End	Miss	Ions	Peptide
1571.7649	1570.7576	1570.8249	-42.83	1 - 14	0	80	-.MIINNNISAINAQR.T

No match to: 1307.6259, 1374.6479, 1557.7516, 1834.8167

20. L0X2I6 **Mass:** 31181 **Score:** 93 **Expect:** 2.1e-05 **Queries matched:** 1
L0X2I6_9SPIR Flagellin OS=Brachyspira hampsonii 30446 GN=A966_05778 PE=3 SV=1

Observed	Mr(expt)	Mr(calc)	ppm	Start - End	Miss	Ions	Peptide
1571.7649	1570.7576	1570.8249	-42.83	1 - 14	0	80	-.MIINNNISAINAQR.T

No match to: 1307.6259, 1374.6479, 1557.7516, 1834.8167

21. L8XTT2 **Mass:** 31194 **Score:** 93 **Expect:** 2.1e-05 **Queries matched:** 1
L8XTT2_9SPIR Flagellin OS=Brachyspira hampsonii 30599 GN=H263_08389 PE=3 SV=1

Observed	Mr(expt)	Mr(calc)	ppm	Start - End	Miss	Ions	Peptide
1571.7649	1570.7576	1570.8249	-42.83	1 - 14	0	80	-.MIINNNISAINAQR.T

No match to: 1307.6259, 1374.6479, 1557.7516, 1834.8167

22. J9UYC2 **Mass:** 31304 **Score:** 64 **Expect:** 0.015 **Queries matched:** 1
J9UYC2_BRAPL Flagellin OS=Brachyspira pilosicoli B2904 GN=B2904_orf2581 PE=3 SV=1

Observed	Mr(expt)	Mr(calc)	ppm	Start - End	Miss	Ions	Peptide
1557.7516	1556.7443	1556.8093	-41.71	1 - 14	0	52	-.MIINNNISAVNAQR.T

No match to: 1307.6259, 1374.6479, 1571.7649, 1834.8167

23. K0JHQ4 **Mass:** 31288 **Score:** 64 **Expect:** 0.015 **Queries matched:** 1
K0JHQ4_BRAPL Flagellin OS=Brachyspira pilosicoli WesB GN=flaB2 PE=3 SV=1

Observed	Mr(expt)	Mr(calc)	ppm	Start - End	Miss	Ions	Peptide
1557.7516	1556.7443	1556.8093	-41.71	1 - 14	0	52	-.MIINNNISAVNAQR.T

No match to: 1307.6259, 1374.6479, 1571.7649, 1834.8167

24. G0ENJ8 **Mass:** 83561 **Score:** 43 **Expect:** 2.2 **Queries matched:** 3
G0ENJ8_BRAIP Aconitate hydratase OS=Brachyspira intermedia (strain ATCC 51140 / PWS/A) GN=Bint_1258 PE=4 SV=1

Observed	Mr(expt)	Mr(calc)	ppm	Start - End	Miss	Ions	Peptide
1307.6259	1306.6186	1306.5677	39.0	297 - 307	0	---	K.ADEEVYNNPEK.Y
1374.6479	1373.6406	1373.7038	-45.99	487 - 498	1	8	K.DTLINEEGKEVK.L
1557.7516	1556.7443	1556.7769	-20.94	645 - 657	1	---	R.EHAAMEPRFLNVK.A + Oxidation (M)

No match to: 1571.7649, 1834.8167

Search Parameters

```
Type of search         : Sequence Query
Enzyme                 : Trypsin
Fixed modifications    : Carbamidomethyl (C)
Variable modifications : Oxidation (M)
Mass values            : Monoisotopic
Protein Mass           : Unrestricted
Peptide Mass Tolerance : ± 50 ppm
Fragment Mass Tolerance: ± 0.5 Da
Max Missed Cleavages   : 1
Instrument type        : MALDI-TOF-TOF
Query1 (1307.6259,1+)  : Locus:37.1.1.211184.56695
Query2 (1374.6479,1+)  : Locus:37.1.1.211184.56698
Query3 (1557.7516,1+)  : Locus:37.1.1.211184.56696
Query4 (1571.7649,1+)  : Locus:37.1.1.211184.56697
```

Query5 (1834.8167,1+) : Locus:37.1.1.211184.56699

**Mascot:** http://www.matrixscience.com/

Mascot Search Results

```
User          :
Email         :
Search title  : F:\ROD_inmunoproteoma\MALDI\ppw_2016_561_562_563_565\ppw_H10_145467559122.txt
MS data file  : F:\ROD_inmunoproteoma\MALDI\ppw_2016_561_562_563_565\ppw_H10_145467559122.txt
Database      : Brachyspiraceae Brachyspiraceae_20160127-151122 (40573 sequences; 13470793 residues)
Timestamp     : 23 Feb 2016 at 16:37:07 GMT
Warning       : A Peptide summary report will usually give a much clearer picture of MS/MS search results.
Top Score     : 234 for C0QWY9, FLAAL_BRAHW Putative flagellar filament outer layer-like protein OS=Brachyspira hyodysenteriae (strain ATC
```

Probability Based Mowse Score

Protein score is -10*Log(P), where P is the probability that the observed match is a random event.
Protein scores greater than 59 are significant (p<0.05).
Protein scores are derived from ions scores as a non-probabilistic basis for ranking protein hits.

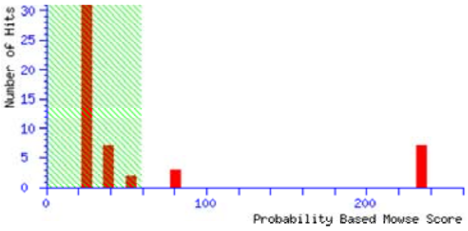

Protein Summary Report

Format As | Protein Summary (deprecated) | Help

Significance threshold p< 0.05 | Max. number of hits AUTO

Standard scoring ◉ MudPIT scoring ○ | Ions score or expect cut-off 0 | Show sub-sets 0

Show pop-ups ◉ Suppress pop-ups ○ | Sort unassigned Decreasing Score | Require bold red ☐

Re-Search All | Search Unmatched

Index

	Accession	Mass	Score	Description
1.	C0QWY9	24747	234	FLAAL_BRAHW Putative flagellar filament outer layer-like protein OS=Brachyspira hyodysenteriae (strain ATCC 49
2.	G0EMH2	24694	234	G0EMH2_BRAIP Flagellar filament outer layer protein FlaA OS=Brachyspira intermedia (strain ATCC 51140 / PWS/A)
3.	A0A0H0XM61	26786	233	A0A0H0XM61_BRAHO Flagellar filament protein FlaA OS=Brachyspira hyodysenteriae GN=SR30_02315 PE=4 SV=1
4.	A0A0G4K408	26732	233	A0A0G4K408_9SPIR Putative flagellar filament outer layer-like protein OS=Brachyspira suanatina GN=flaAL PE=4 S
5.	L0X1P6	26748	233	L0X1P6_9SPIR Flagellar filament outer layer protein FlaA OS=Brachyspira hampsonii 30446 GN=A966_07050 PE=4 SV=
6.	D5U8S6	26845	233	D5U8S6_BRAM5 Flagellar filament outer layer protein FlaA OS=Brachyspira murdochii (strain ATCC 51284 / DSM 125
7.	L8XPJ3	27049	233	L8XPJ3_9SPIR Flagellar filament outer layer protein FlaA OS=Brachyspira hampsonii 30599 GN=H263_14877 PE=4 SV=
8.	D8ICA7	23998	78	D8ICA7_BRAP9 Flagellar filament outer layer protein, FlaA-2 OS=Brachyspira pilosicoli (strain ATCC BAA-1826 /
9.	J9U0T5	26037	78	J9U0T5_BRAPL Flagellar filament outer layer protein FlaA-0 OS=Brachyspira pilosicoli B2904 GN=B2904_orf2169 PE
10.	K0JKQ0	26037	78	K0JKQ0_BRAPL Flagellar filament outer layer protein FlaA-2 OS=Brachyspira pilosicoli WesB GN=flaA-2 PE=4 SV=1
11.	R5HH97	9276	50	R5HH97_9SPIR Uncharacterized protein OS=Brachyspira sp. CAG:484 GN=BN676_00712 PE=4 SV=1

Results List

1. C0QWY9 **Mass:** 24747 **Score:** 234 **Expect:** 1.6e-19 **Queries matched:** 3
FLAAL_BRAHW Putative flagellar filament outer layer-like protein OS=Brachyspira hyodysenteriae (strain ATCC 49526 / WA1) GN=flaAL PE=1

Observed	Mr(expt)	Mr(calc)	ppm	Start	End	Miss	Ions	Peptide
1021.6055	1020.5982	1020.5604	37.1	50 -	58	0	48	R.DYGVVSIIR.R
1177.7051	1176.6978	1176.6615	30.9	50 -	59	1	72	R.DYGVVSIIRR.E
1731.9810	1730.9737	1730.9144	34.2	87 -	101	0	64	R.TGYPWFSVTPPRPVK.I

No match to: 1028.5901, 1743.9816

2. G0EMH2 **Mass:** 24694 **Score:** 234 **Expect:** 1.6e-19 **Queries matched:** 3
G0EMH2_BRAIP Flagellar filament outer layer protein FlaA OS=Brachyspira intermedia (strain ATCC 51140 / PWS/A) GN=Bint_2337 PE=4 SV=1

Observed	Mr(expt)	Mr(calc)	ppm	Start	End	Miss	Ions	Peptide
1021.6055	1020.5982	1020.5604	37.1	50 -	58	0	48	R.DYGVVSIIR.R
1177.7051	1176.6978	1176.6615	30.9	50 -	59	1	72	R.DYGVVSIIRR.E
1731.9810	1730.9737	1730.9144	34.2	87 -	101	0	64	R.TGYPWFSVTPPRPVK.I

No match to: 1028.5901, 1743.9816

3. A0A0H0XM61 **Mass:** 26786 **Score:** 233 **Expect:** 2e-19 **Queries matched:** 3
A0A0H0XM61_BRAHO Flagellar filament protein FlaA OS=Brachyspira hyodysenteriae GN=SR30_02315 PE=4 SV=1

Observed	Mr(expt)	Mr(calc)	ppm	Start	End	Miss	Ions	Peptide
1021.6055	1020.5982	1020.5604	37.1	68 -	76	0	48	R.DYGVVSIIR.R
1177.7051	1176.6978	1176.6615	30.9	68 -	77	1	72	R.DYGVVSIIRR.E
1731.9810	1730.9737	1730.9144	34.2	105 -	119	0	64	R.TGYPWFSVTPPRPVK.I

No match to: 1028.5901, 1743.9816

4. A0A0G4K408 **Mass:** 26732 **Score:** 233 **Expect:** 2e-19 **Queries matched:** 3
A0A0G4K408_9SPIR Putative flagellar filament outer layer-like protein OS=Brachyspira suanatina GN=flaAL PE=4 SV=1

Observed	Mr(expt)	Mr(calc)	ppm	Start	End	Miss	Ions	Peptide
1021.6055	1020.5982	1020.5604	37.1	68 -	76	0	48	R.DYGVVSIIR.R

Observed	Mr(expt)	Mr(calc)	ppm	Start	End	Miss	Ions	Peptide
1177.7051	1176.6978	1176.6615	30.9	68 -	77	1	72	R.DYGVVSIIRR.E
1731.9810	1730.9737	1730.9144	34.2	105 -	119	0	64	R.TGYPWFSVTPPRPVK.I

No match to: 1028.5901, 1743.9816

5. L0X1P6 **Mass:** 26748 **Score:** 233 **Expect:** 2e-19 **Queries matched:** 3
L0X1P6_9SPIR Flagellar filament outer layer protein FlaA OS=Brachyspira hampsonii 30446 GN=A966_07050 PE=4 SV=1

Observed	Mr(expt)	Mr(calc)	ppm	Start	End	Miss	Ions	Peptide
1021.6055	1020.5982	1020.5604	37.1	68 -	76	0	48	R.DYGVVSIIR.R
1177.7051	1176.6978	1176.6615	30.9	68 -	77	1	72	R.DYGVVSIIRR.E
1731.9810	1730.9737	1730.9144	34.2	105 -	119	0	64	R.TGYPWFSVTPPRPVK.I

No match to: 1028.5901, 1743.9816

6. D5U8S6 **Mass:** 26845 **Score:** 233 **Expect:** 2e-19 **Queries matched:** 3
D5U8S6_BRAM5 Flagellar filament outer layer protein FlaA OS=Brachyspira murdochii (strain ATCC 51284 / DSM 12563 / 56-150) GN=Bmur_10[illegible]

Observed	Mr(expt)	Mr(calc)	ppm	Start	End	Miss	Ions	Peptide
1021.6055	1020.5982	1020.5604	37.1	69 -	77	0	48	R.DYGVVSIIR.R
1177.7051	1176.6978	1176.6615	30.9	69 -	78	1	72	R.DYGVVSIIRR.E
1731.9810	1730.9737	1730.9144	34.2	106 -	120	0	64	R.TGYPWFSVTPPRPVK.I

No match to: 1028.5901, 1743.9816

7. L8XPJ3 **Mass:** 27049 **Score:** 233 **Expect:** 2e-19 **Queries matched:** 3
L8XPJ3_9SPIR Flagellar filament outer layer protein FlaA OS=Brachyspira hampsonii 30599 GN=H263_14877 PE=4 SV=1

Observed	Mr(expt)	Mr(calc)	ppm	Start	End	Miss	Ions	Peptide
1021.6055	1020.5982	1020.5604	37.1	70 -	78	0	48	R.DYGVVSIIR.R
1177.7051	1176.6978	1176.6615	30.9	70 -	79	1	72	R.DYGVVSIIRR.E
1731.9810	1730.9737	1730.9144	34.2	107 -	121	0	64	R.TGYPWFSVTPPRPVK.I

No match to: 1028.5901, 1743.9816

8. D8ICA7 **Mass:** 23998 **Score:** 78 **Expect:** 0.00063 **Queries matched:** 1
D8ICA7_BRAP9 Flagellar filament outer layer protein, FlaA-2 OS=Brachyspira pilosicoli (strain ATCC BAA-1826 / 95/1000) GN=flaA-2 PE=4

Observed	Mr(expt)	Mr(calc)	ppm	Start	End	Miss	Ions	Peptide
1731.9810	1730.9737	1730.9144	34.2	80 -	94	0	64	K.TGYPWFSVTPPRPVK.I

No match to: 1021.6055, 1028.5901, 1177.7051, 1743.9816

9. J9U0T5 **Mass:** 26037 **Score:** 78 **Expect:** 0.00069 **Queries matched:** 1
J9U0T5_BRAPL Flagellar filament outer layer protein FlaA-0 OS=Brachyspira pilosicoli B2904 GN=B2904_orf2169 PE=4 SV=1

Observed	Mr(expt)	Mr(calc)	ppm	Start	End	Miss	Ions	Peptide
1731.9810	1730.9737	1730.9144	34.2	98 -	112	0	64	K.TGYPWFSVTPPRPVK.I

No match to: 1021.6055, 1028.5901, 1177.7051, 1743.9816

10. K0JKQ0 **Mass:** 26037 **Score:** 78 **Expect:** 0.00069 **Queries matched:** 1
K0JKQ0_BRAPL Flagellar filament outer layer protein FlaA-2 OS=Brachyspira pilosicoli WesB GN=flaA-2 PE=4 SV=1

Observed	Mr(expt)	Mr(calc)	ppm	Start	End	Miss	Ions	Peptide
1731.9810	1730.9737	1730.9144	34.2	98 -	112	0	64	K.TGYPWFSVTPPRPVK.I

No match to: 1021.6055, 1028.5901, 1177.7051, 1743.9816

11. R5HH97 **Mass:** 9276 **Score:** 50 **Expect:** 0.42 **Queries matched:** 2
R5HH97_9SPIR Uncharacterized protein OS=Brachyspira sp. CAG:484 GN=BN676_00712 PE=4 SV=1

Observed	Mr(expt)	Mr(calc)	ppm	Start	End	Miss	Ions	Peptide
1021.6055	1020.5982	1020.5968	1.42	52 -	60	0	13	R.ATTLFSILR.A
1028.5901	1027.5828	1027.5774	5.26	10 -	18	1	---	K.LGDGIRLER.L

No match to: 1177.7051, 1731.9810, 1743.9816

Search Parameters

Type of search : Sequence Query
Enzyme : Trypsin
Fixed modifications : Carbamidomethyl (C)
Variable modifications : Oxidation (M)
Mass values : Monoisotopic
Protein Mass : Unrestricted
Peptide Mass Tolerance : ± 50 ppm
Fragment Mass Tolerance: ± 0.5 Da
Max Missed Cleavages : 1
Instrument type : MALDI-TOF-TOF
Query1 (1021.6055,1+) : Locus:10.1.1.212744.57046
Query2 (1028.5901,1+) : Locus:10.1.1.212744.57047
Query3 (1177.7051,1+) : Locus:10.1.1.212744.57045
Query4 (1731.9810,1+) : Locus:10.1.1.212744.57044
Query5 (1743.9816,1+) : Locus:10.1.1.212744.57048

Mascot: http://www.matrixscience.com/

Mascot Search Results

**User**	:
**Email**	:
**Search title**	: F:\ROD_inmunoproteoma\MALDI\ppw_2016_562_563_ROD\ppw_I7_145397049723.txt
**MS data file**	: F:\ROD_inmunoproteoma\MALDI\ppw_2016_562_563_ROD\ppw_I7_145397049723.txt
**Database**	: Brachyspiraceae Brachyspiraceae_20160127-151122 (40573 sequences; 13470793 residues)
**Timestamp**	: 23 Feb 2016 at 16:06:01 GMT
**Warning**	: A Peptide summary report will usually give a much clearer picture of MS/MS search results.
**Top Score**	: 158 for C0QWY9, FLAAL_BRAHW Putative flagellar filament outer layer-like protein OS=Brachyspira hyodysenteriae (strain ATC

Probability Based Mowse Score

Protein score is -10*Log(P), where P is the probability that the observed match is a random event.
Protein scores greater than 59 are significant (p<0.05).
Protein scores are derived from ions scores as a non-probabilistic basis for ranking protein hits.

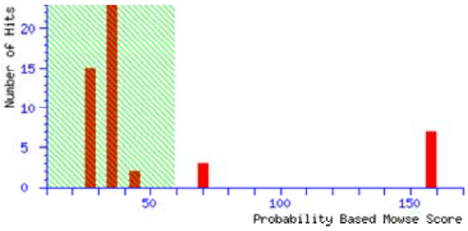

Protein Summary Report

Format As | Protein Summary (deprecated) | Help

Significance threshold p< 0.05 | Max. number of hits AUTO

Standard scoring (•) MudPIT scoring ( ) | Ions score or expect cut-off 0 | Show sub-sets 0

Show pop-ups (•) Suppress pop-ups ( ) | Sort unassigned Decreasing Score | Require bold red [ ]

Re-Search All | Search Unmatched

Index

	Accession	Mass	Score	Description
1.	C0QWY9	24747	158	FLAAL_BRAHW Putative flagellar filament outer layer-like protein OS=Brachyspira hyodysenteriae (strain ATCC 49
2.	G0EMH2	24694	158	G0EMH2_BRAIP Flagellar filament outer layer protein FlaA OS=Brachyspira intermedia (strain ATCC 51140 / PWS/A)
3.	A0A0H0XM61	26786	157	A0A0H0XM61_BRAHO Flagellar filament protein FlaA OS=Brachyspira hyodysenteriae GN=SR30_02315 PE=4 SV=1
4.	A0A0G4K408	26732	157	A0A0G4K408_9SPIR Putative flagellar filament outer layer-like protein OS=Brachyspira suanatina GN=flaAL PE=4 S
5.	L0X1P6	26748	157	L0X1P6_9SPIR Flagellar filament outer layer protein FlaA OS=Brachyspira hampsonii 30446 GN=A966_07050 PE=4 SV=
6.	D5U8S6	26845	157	D5U8S6_BRAM5 Flagellar filament outer layer protein FlaA OS=Brachyspira murdochii (strain ATCC 51284 / DSM 125
7.	L8XPJ3	27049	157	L8XPJ3_9SPIR Flagellar filament outer layer protein FlaA OS=Brachyspira hampsonii 30599 GN=H263_14877 PE=4 SV=
8.	D8ICA7	23998	67	D8ICA7_BRAP9 Flagellar filament outer layer protein, FlaA-2 OS=Brachyspira pilosicoli (strain ATCC BAA-1826 /
9.	J9U0T5	26037	66	J9U0T5_BRAPL Flagellar filament outer layer protein FlaA-0 OS=Brachyspira pilosicoli B2904 GN=B2904_orf2169 PE
10.	K0JKQ0	26037	66	K0JKQ0_BRAPL Flagellar filament outer layer protein FlaA-2 OS=Brachyspira pilosicoli WesB GN=flaA-2 PE=4 SV=1
11.	J9USC0	13333	40	J9USC0_BRAPL 50S ribosomal protein L7/L12 OS=Brachyspira pilosicoli B2904 GN=rplL PE=3 SV=1

Results List

1. C0QWY9 **Mass:** 24747 **Score:** 158 **Expect:** 6.4e-12 **Queries matched:** 2
FLAAL_BRAHW Putative flagellar filament outer layer-like protein OS=Brachyspira hyodysenteriae (strain ATCC 49526 / WA1) GN=flaAL PE=1

Observed	Mr(expt)	Mr(calc)	ppm	Start		End	Miss	Ions	Peptide
1177.6119	1176.6046	1176.6615	-48.34	50	-	59	1	75	R.DYGVVSIIRR.E
1731.8381	1730.8308	1730.9144	-48.31	87	-	101	0	53	R.TGYPWFSVTPPRPVK.I

No match to: 1239.5286, 1954.8615, 2896.3196

2. G0EMH2 **Mass:** 24694 **Score:** 158 **Expect:** 6.4e-12 **Queries matched:** 2
G0EMH2_BRAIP Flagellar filament outer layer protein FlaA OS=Brachyspira intermedia (strain ATCC 51140 / PWS/A) GN=Bint_2337 PE=4 SV=1

Observed	Mr(expt)	Mr(calc)	ppm	Start		End	Miss	Ions	Peptide
1177.6119	1176.6046	1176.6615	-48.34	50	-	59	1	75	R.DYGVVSIIRR.E
1731.8381	1730.8308	1730.9144	-48.31	87	-	101	0	53	R.TGYPWFSVTPPRPVK.I

No match to: 1239.5286, 1954.8615, 2896.3196

3. A0A0H0XM61 **Mass:** 26786 **Score:** 157 **Expect:** 8.1e-12 **Queries matched:** 2
A0A0H0XM61_BRAHO Flagellar filament protein FlaA OS=Brachyspira hyodysenteriae GN=SR30_02315 PE=4 SV=1

Observed	Mr(expt)	Mr(calc)	ppm	Start		End	Miss	Ions	Peptide
1177.6119	1176.6046	1176.6615	-48.34	68	-	77	1	75	R.DYGVVSIIRR.E
1731.8381	1730.8308	1730.9144	-48.31	105	-	119	0	53	R.TGYPWFSVTPPRPVK.I

No match to: 1239.5286, 1954.8615, 2896.3196

4. A0A0G4K408 **Mass:** 26732 **Score:** 157 **Expect:** 8.1e-12 **Queries matched:** 2
A0A0G4K408_9SPIR Putative flagellar filament outer layer-like protein OS=Brachyspira suanatina GN=flaAL PE=4 SV=1

Observed	Mr(expt)	Mr(calc)	ppm	Start		End	Miss	Ions	Peptide
1177.6119	1176.6046	1176.6615	-48.34	68	-	77	1	75	R.DYGVVSIIRR.E
1731.8381	1730.8308	1730.9144	-48.31	105	-	119	0	53	R.TGYPWFSVTPPRPVK.I

No match to: 1239.5286, 1954.8615, 2896.3196

5. L0X1P6 **Mass:** 26748 **Score:** 157 **Expect:** 8.1e-12 **Queries matched:** 2
L0X1P6_9SPIR Flagellar filament outer layer protein FlaA OS=Brachyspira hampsonii 30446 GN=A966_07050 PE=4 SV=1

Observed	Mr(expt)	Mr(calc)	ppm	Start		End	Miss	Ions	Peptide
1177.6119	1176.6046	1176.6615	-48.34	68	-	77	1	75	R.DYGVVSIIRR.E
1731.8381	1730.8308	1730.9144	-48.31	105	-	119	0	53	R.TGYPWFSVTPPRPVK.I

No match to: 1239.5286, 1954.8615, 2896.3196

6. D5U8S6 **Mass:** 26845 **Score:** 157 **Expect:** 8.1e-12 **Queries matched:** 2
D5U8S6_BRAM5 Flagellar filament outer layer protein FlaA OS=Brachyspira murdochii (strain ATCC 51284 / DSM 12563 / 56-150) GN=Bmur_10[illegible]

Observed	Mr(expt)	Mr(calc)	ppm	Start		End	Miss	Ions	Peptide
1177.6119	1176.6046	1176.6615	-48.34	69	-	78	1	75	R.DYGVVSIIRR.E
1731.8381	1730.8308	1730.9144	-48.31	106	-	120	0	53	R.TGYPWFSVTPPRPVK.I

No match to: 1239.5286, 1954.8615, 2896.3196

7. L8XPJ3 **Mass:** 27049 **Score:** 157 **Expect:** 8.1e-12 **Queries matched:** 2
L8XPJ3_9SPIR Flagellar filament outer layer protein FlaA OS=Brachyspira hampsonii 30599 GN=H263_14877 PE=4 SV=1

Observed	Mr(expt)	Mr(calc)	ppm	Start		End	Miss	Ions	Peptide
1177.6119	1176.6046	1176.6615	-48.34	70	-	79	1	75	R.DYGVVSIIRR.E
1731.8381	1730.8308	1730.9144	-48.31	107	-	121	0	53	R.TGYPWFSVTPPRPVK.I

No match to: 1239.5286, 1954.8615, 2896.3196

8. D8ICA7 **Mass:** 23998 **Score:** 67 **Expect:** 0.0089 **Queries matched:** 1
D8ICA7_BRAP9 Flagellar filament outer layer protein, FlaA-2 OS=Brachyspira pilosicoli (strain ATCC BAA-1826 / 95/1000) GN=flaA-2 PE=4

Observed	Mr(expt)	Mr(calc)	ppm	Start		End	Miss	Ions	Peptide
1731.8381	1730.8308	1730.9144	-48.31	80	-	94	0	53	K.TGYPWFSVTPPRPVK.I

No match to: 1177.6119, 1239.5286, 1954.8615, 2896.3196

9. J9U0T5 **Mass:** 26037 **Score:** 66 **Expect:** 0.0095 **Queries matched:** 1
J9U0T5_BRAPL Flagellar filament outer layer protein FlaA-0 OS=Brachyspira pilosicoli B2904 GN=B2904_orf2169 PE=4 SV=1

Observed	Mr(expt)	Mr(calc)	ppm	Start		End	Miss	Ions	Peptide
1731.8381	1730.8308	1730.9144	-48.31	98	-	112	0	53	K.TGYPWFSVTPPRPVK.I

No match to: 1177.6119, 1239.5286, 1954.8615, 2896.3196

10. K0JKQ0 **Mass:** 26037 **Score:** 66 **Expect:** 0.0095 **Queries matched:** 1
K0JKQ0_BRAPL Flagellar filament outer layer protein FlaA-2 OS=Brachyspira pilosicoli WesB GN=flaA-2 PE=4 SV=1

Observed	Mr(expt)	Mr(calc)	ppm	Start		End	Miss	Ions	Peptide
1731.8381	1730.8308	1730.9144	-48.31	98	-	112	0	53	K.TGYPWFSVTPPRPVK.I

No match to: 1177.6119, 1239.5286, 1954.8615, 2896.3196

11. J9USC0 **Mass:** 13333 **Score:** 40 **Expect:** 4.1 **Queries matched:** 2
J9USC0_BRAPL 50S ribosomal protein L7/L12 OS=Brachyspira pilosicoli B2904 GN=rplL PE=3 SV=1

Observed	Mr(expt)	Mr(calc)	ppm	Start		End	Miss	Ions	Peptide
1731.8381	1730.8308	1730.9124	-47.11	2	-	16	1	---	M.ALSKEEILQAIEEMK.V
2896.3196	2895.3123	2895.4436	-45.34	29	-	59	1	---	K.EKFNVTAAMPVAAVAAAPAAGGAAPAADEEK.S

No match to: 1177.6119, 1239.5286, 1954.8615

Search Parameters

Type of search : Sequence Query
Enzyme : Trypsin
Fixed modifications : Carbamidomethyl (C)
Variable modifications : Oxidation (M)
Mass values : Monoisotopic
Protein Mass : Unrestricted
Peptide Mass Tolerance : ± 50 ppm
Fragment Mass Tolerance: ± 0.5 Da
Max Missed Cleavages : 1
Instrument type : MALDI-TOF-TOF
Query1 (1177.6119,1+) : Locus:24.1.1.211171.56630
Query2 (1239.5286,1+) : Locus:24.1.1.211171.56631
Query3 (1731.8381,1+) : Locus:24.1.1.211171.56634
Query4 (1954.8615,1+) : Locus:24.1.1.211171.56632
Query5 (2896.3196,1+) : Locus:24.1.1.211171.56633

Mascot: http://www.matrixscience.com/

Mascot Search Results

User :
Email :
Search title : F:\ROD_inmunoproteoma\MALDI\160223_2016_561_562_ROD\ppw_G5_145631468112.txt
MS data file : F:\ROD_inmunoproteoma\MALDI\160223_2016_561_562_ROD\ppw_G5_145631468112.txt
Database : Brachyspiraceae Brachyspiraceae_20160127-151122 (40573 sequences; 13470793 residues)
Timestamp : 24 Feb 2016 at 12:02:53 GMT
Warning : A Peptide summary report will usually give a much clearer picture of MS/MS search results.
Top Score : 412 for D8ICA7, D8ICA7_BRAP9 Flagellar filament outer layer protein, FlaA-2 OS=Brachyspira pilosicoli (strain ATCC BAA-182

Probability Based Mowse Score

Protein score is -10*Log(P), where P is the probability that the observed match is a random event.
Protein scores greater than 59 are significant (p<0.05).
Protein scores are derived from ions scores as a non-probabilistic basis for ranking protein hits.

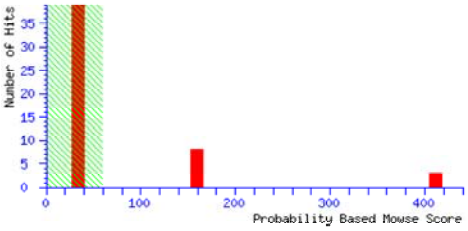

Protein Summary Report

Format As | Protein Summary (deprecated) | Help

Significance threshold p< 0.05 | Max. number of hits AUTO

Standard scoring ◉ MudPIT scoring ○ | Ions score or expect cut-off 0 | Show sub-sets 0

Show pop-ups ◉ Suppress pop-ups ○ | Sort unassigned Decreasing Score | Require bold red ☐

Re-Search All | Search Unmatched

Index

	Accession	Mass	Score	Description
1.	D8ICA7	23998	412	D8ICA7_BRAP9 Flagellar filament outer layer protein, FlaA-2 OS=Brachyspira pilosicoli (strain ATCC BAA-1826 /
2.	J9U0T5	26037	411	J9U0T5_BRAPL Flagellar filament outer layer protein FlaA-0 OS=Brachyspira pilosicoli B2904 GN=B2904_orf2169 PE
3.	K0JKQ0	26037	411	K0JKQ0_BRAPL Flagellar filament outer layer protein FlaA-2 OS=Brachyspira pilosicoli WesB GN=flaA-2 PE=4 SV=1
4.	R5LT25	26061	163	R5LT25_9SPIR Flagellar filament outer layer protein FlaA OS=Brachyspira sp. CAG:700 GN=BN758_01590 PE=4 SV=1
5.	C0QWY9	24747	156	FLAAL_BRAHW Putative flagellar filament outer layer-like protein OS=Brachyspira hyodysenteriae (strain ATCC 49
6.	G0EMH2	24694	156	G0EMH2_BRAIP Flagellar filament outer layer protein FlaA OS=Brachyspira intermedia (strain ATCC 51140 / PWS/A)
7.	A0A0H0XM61	26786	156	A0A0H0XM61_BRAHO Flagellar filament protein FlaA OS=Brachyspira hyodysenteriae GN=SR30_02315 PE=4 SV=1
8.	A0A0G4K408	26732	156	A0A0G4K408_9SPIR Putative flagellar filament outer layer-like protein OS=Brachyspira suanatina GN=flaAL PE=4 S
9.	L0X1P6	26748	156	L0X1P6_9SPIR Flagellar filament outer layer protein FlaA OS=Brachyspira hampsonii 30446 GN=A966_07050 PE=4 SV=
10.	D5U8S6	26845	156	D5U8S6_BRAM5 Flagellar filament outer layer protein FlaA OS=Brachyspira murdochii (strain ATCC 51284 / DSM 125
11.	L8XPJ3	27049	156	L8XPJ3_9SPIR Flagellar filament outer layer protein FlaA OS=Brachyspira hampsonii 30599 GN=H263_14877 PE=4 SV=
12.	J9TZH7	36672	42	J9TZH7_BRAPL Phosphate acyltransferase OS=Brachyspira pilosicoli B2904 GN=plsX PE=3 SV=1

Results List

1. D8ICA7 **Mass:** 23998 **Score:** 412 **Expect:** 2.6e-37 **Queries matched:** 4
D8ICA7_BRAP9 Flagellar filament outer layer protein, FlaA-2 OS=Brachyspira pilosicoli (strain ATCC BAA-1826 / 95/1000) GN=flaA-2 PE=4

Observed	Mr(expt)	Mr(calc)	ppm	Start		End	Miss	Ions	Peptide
1016.5771	1015.5698	1015.5451	24.4	101	-	109	0	85	K.EISVWVAGR.N
1191.7017	1190.6944	1190.6771	14.5	43	-	52	1	72	R.DYGVISIIRR.E
1731.9473	1730.9400	1730.9144	14.8	80	-	94	0	41	K.TGYPWFSVTPPRPVK.I
2016.9648	2015.9575	2015.9265	15.4	176	-	191	1	140	R.DSYGKYYIYFDNLEAR.T

No match to: 1225.5924

2. J9U0T5 **Mass:** 26037 **Score:** 411 **Expect:** 3.2e-37 **Queries matched:** 4
J9U0T5_BRAPL Flagellar filament outer layer protein FlaA-0 OS=Brachyspira pilosicoli B2904 GN=B2904_orf2169 PE=4 SV=1

Observed	Mr(expt)	Mr(calc)	ppm	Start		End	Miss	Ions	Peptide
1016.5771	1015.5698	1015.5451	24.4	119	-	127	0	85	K.EISVWVAGR.N
1191.7017	1190.6944	1190.6771	14.5	61	-	70	1	72	R.DYGVISIIRR.E
1731.9473	1730.9400	1730.9144	14.8	98	-	112	0	41	K.TGYPWFSVTPPRPVK.I
2016.9648	2015.9575	2015.9265	15.4	194	-	209	1	140	R.DSYGKYYIYFDNLEAR.T

No match to: 1225.5924

3. K0JKQ0 **Mass:** 26037 **Score:** 411 **Expect:** 3.2e-37 **Queries matched:** 4
K0JKQ0_BRAPL Flagellar filament outer layer protein FlaA-2 OS=Brachyspira pilosicoli WesB GN=flaA-2 PE=4 SV=1

Observed	Mr(expt)	Mr(calc)	ppm	Start		End	Miss	Ions	Peptide
1016.5771	1015.5698	1015.5451	24.4	119	-	127	0	85	K.EISVWVAGR.N
1191.7017	1190.6944	1190.6771	14.5	61	-	70	1	72	R.DYGVISIIRR.E
1731.9473	1730.9400	1730.9144	14.8	98	-	112	0	41	K.TGYPWFSVTPPRPVK.I
2016.9648	2015.9575	2015.9265	15.4	194	-	209	1	140	R.DSYGKYYIYFDNLEAR.T

No match to: 1225.5924

4. R5LT25 **Mass:** 26061 **Score:** 163 **Expect:** 2e-12 **Queries matched:** 2
R5LT25_9SPIR Flagellar filament outer layer protein FlaA OS=Brachyspira sp. CAG:700 GN=BN758_01590 PE=4 SV=1

Observed	Mr(expt)	Mr(calc)	ppm	Start	End	Miss	Ions	Peptide
1016.5771	1015.5698	1015.5451	24.4	120 -	128	0	85	K.EISVWVAGR.N
1191.7017	1190.6944	1190.6771	14.5	62 -	71	1	49	R.DYGLVSIIRR.E

No match to: 1225.5924, 1731.9473, 2016.9648

5. C0QWY9 **Mass:** 24747 **Score:** 156 **Expect:** 1e-11 **Queries matched:** 2
FLAAL_BRAHW Putative flagellar filament outer layer-like protein OS=Brachyspira hyodysenteriae (strain ATCC 49526 / WA1) GN=flaAL PE=1

Observed	Mr(expt)	Mr(calc)	ppm	Start	End	Miss	Ions	Peptide
1016.5771	1015.5698	1015.5451	24.4	108 -	116	0	85	K.ELSVWVAGR.N
1731.9473	1730.9400	1730.9144	14.8	87 -	101	0	41	R.TGYPWFSVTPPRPVK.I

No match to: 1191.7017, 1225.5924, 2016.9648

6. G0EMH2 **Mass:** 24694 **Score:** 156 **Expect:** 1e-11 **Queries matched:** 2
G0EMH2_BRAIP Flagellar filament outer layer protein FlaA OS=Brachyspira intermedia (strain ATCC 51140 / PWS/A) GN=Bint_2337 PE=4 SV=1

Observed	Mr(expt)	Mr(calc)	ppm	Start	End	Miss	Ions	Peptide
1016.5771	1015.5698	1015.5451	24.4	108 -	116	0	85	K.ELSVWVAGR.N
1731.9473	1730.9400	1730.9144	14.8	87 -	101	0	41	R.TGYPWFSVTPPRPVK.I

No match to: 1191.7017, 1225.5924, 2016.9648

7. A0A0H0XM61 **Mass:** 26786 **Score:** 156 **Expect:** 1e-11 **Queries matched:** 2
A0A0H0XM61_BRAHO Flagellar filament protein FlaA OS=Brachyspira hyodysenteriae GN=SR30_02315 PE=4 SV=1

Observed	Mr(expt)	Mr(calc)	ppm	Start	End	Miss	Ions	Peptide
1016.5771	1015.5698	1015.5451	24.4	126 -	134	0	85	K.ELSVWVAGR.N
1731.9473	1730.9400	1730.9144	14.8	105 -	119	0	41	R.TGYPWFSVTPPRPVK.I

No match to: 1191.7017, 1225.5924, 2016.9648

8. A0A0G4K408 **Mass:** 26732 **Score:** 156 **Expect:** 1e-11 **Queries matched:** 2
A0A0G4K408_9SPIR Putative flagellar filament outer layer-like protein OS=Brachyspira suanatina GN=flaAL PE=4 SV=1

Observed	Mr(expt)	Mr(calc)	ppm	Start	End	Miss	Ions	Peptide
1016.5771	1015.5698	1015.5451	24.4	126 -	134	0	85	K.ELSVWVAGR.N
1731.9473	1730.9400	1730.9144	14.8	105 -	119	0	41	R.TGYPWFSVTPPRPVK.I

No match to: 1191.7017, 1225.5924, 2016.9648

9. L0X1P6 **Mass:** 26748 **Score:** 156 **Expect:** 1e-11 **Queries matched:** 2
L0X1P6_9SPIR Flagellar filament outer layer protein FlaA OS=Brachyspira hampsonii 30446 GN=A966_07050 PE=4 SV=1

Observed	Mr(expt)	Mr(calc)	ppm	Start	End	Miss	Ions	Peptide
1016.5771	1015.5698	1015.5451	24.4	126 -	134	0	85	K.ELSVWVAGR.N
1731.9473	1730.9400	1730.9144	14.8	105 -	119	0	41	R.TGYPWFSVTPPRPVK.I

No match to: 1191.7017, 1225.5924, 2016.9648

10. D5U8S6 **Mass:** 26845 **Score:** 156 **Expect:** 1e-11 **Queries matched:** 2
D5U8S6_BRAM5 Flagellar filament outer layer protein FlaA OS=Brachyspira murdochii (strain ATCC 51284 / DSM 12563 / 56-150) GN=Bmur_10

Observed	Mr(expt)	Mr(calc)	ppm	Start	End	Miss	Ions	Peptide
1016.5771	1015.5698	1015.5451	24.4	127 -	135	0	85	K.EISVWVAGR.N
1731.9473	1730.9400	1730.9144	14.8	106 -	120	0	41	R.TGYPWFSVTPPRPVK.I

No match to: 1191.7017, 1225.5924, 2016.9648

11. L8XPJ3 **Mass:** 27049 **Score:** 156 **Expect:** 1e-11 **Queries matched:** 2
L8XPJ3_9SPIR Flagellar filament outer layer protein FlaA OS=Brachyspira hampsonii 30599 GN=H263_14877 PE=4 SV=1

Observed	Mr(expt)	Mr(calc)	ppm	Start	End	Miss	Ions	Peptide
1016.5771	1015.5698	1015.5451	24.4	128 -	136	0	85	K.ELSVWVAGR.N
1731.9473	1730.9400	1730.9144	14.8	107 -	121	0	41	R.TGYPWFSVTPPRPVK.I

No match to: 1191.7017, 1225.5924, 2016.9648

12. J9TZH7 **Mass:** 36672 **Score:** 42 **Expect:** 2.4 **Queries matched:** 1
J9TZH7_BRAPL Phosphate acyltransferase OS=Brachyspira pilosicoli B2904 GN=plsX PE=3 SV=1

Observed	Mr(expt)	Mr(calc)	ppm	Start	End	Miss	Ions	Peptide
1016.5771	1015.5698	1015.5662	3.58	328 -	336	1	31	K.ELLDSGVKR.R

No match to: 1191.7017, 1225.5924, 1731.9473, 2016.9648

Search Parameters

Type of search : Sequence Query
Enzyme : Trypsin
Fixed modifications : Carbamidomethyl (C)
Variable modifications : Oxidation (M)
Mass values : Monoisotopic
Protein Mass : Unrestricted
Peptide Mass Tolerance : ± 50 ppm
Fragment Mass Tolerance: ± 0.5 Da
Max Missed Cleavages : 1
Instrument type : MALDI-TOF-TOF
Query1 (1016.5771,1+) : Locus:13.1.1.213512.57299
Query2 (1191.7017,1+) : Locus:13.1.1.213512.57296
Query3 (1225.5924,1+) : Locus:13.1.1.213512.57297
Query4 (1731.9473,1+) : Locus:13.1.1.213512.57300
Query5 (2016.9648,1+) : Locus:13.1.1.213512.57298

Mascot: http://www.matrixscience.com/
