# Supplementary material for: Brachyspira hyodysenteriae and B. pilosicoli Proteins Recognized by Sera of Challenged Pigs
Source: Front Microbiol. 2017 May 4;8:723. doi: 10.3389/fmicb.2017.00723 (PMC5415613; doi:10.3389/fmicb.2017.00723)

Supplementary figure S1: Test of the OFFGEL IEF fractionation reproducibility by Coomassie staining of a random selection of 7 of the 24 fractions collected. One milligram of total protein from cell lysates of each *Brachyspira* strain were fractionated for these experiments.

Supplementary figure S2: General view of the immunoreactive proteins in each of the IEF fractions of the protein extracts obtained from *Brachyspira pilosicoli* strains OLA9 (A) , ATCC 51139(B) and *Brachyspira hyodysenteriae* strain V1 (C) .
The images correspond to Western blots prepared with different control and challenged sera (codes at right in each row of gels). Twenty four consecutive IEF protein fractions covering a pI range from 3 to 10, were analyzed in the corresponding lanes of three SDS-PAGE gels. The "Inp" lane corresponds to the crude extract before IEF separation. Fractions presenting intense immunoreactive bands in these preliminary experiments were submitted to a detailed immunoproteomics analysis using all the individual sera available.

Supplementary figure S3: Western-Blot images of selected fractions incubated with three sera from control pigs for: A*) B. pilosicoli* strains OLA9 and ATCC 51139 and B. hyodysenteriae V1 in SDS-PAGE gels of 12% acrylamide, B*)* IEF fractions #8 and #9 from *B. pilosicoli* were also separated on 7.5% acrylamide gels to better resolve the bands in the high-mass range of the gels.

Supplementary figure S4: Western-Blot images of selected fractions for *B. pilosicoli* strains OLA9 and ATCC 51139 and *B. hyodysenteriae* strain V1 incubated with the sera from challenged pigs (code for each individual serum is indicated in the heading of the images).

Supplementary figure S5: Identification of immunoreactive proteins in the selected IEF fractions from the three *Brachyspira* strains studied. A-C*) B. pilosicoli* strain OLA9, D-F) *B. pilosicoli* strain ATCC 51139, and G-H*) B. hyodysenteriae* strain V1. Each image corresponds to a different IEF fraction and shows the Western blot densitometry profiles (left and right) and the protein band profile of the corresponding silver-stained gel lane (middle). Immunoreactivity traces for the 5 sera from challenged pigs (left) and the 3 sera for control pigs (right) are shown with different colors. Bands identified as immunoreactive were sliced from the SDS gel lane and submitted to MS analyses for identification. Code numbers for the bands analyzed from each lane are indicated in red. All fractions were separated in 12% acrylamide gels. In the case of IEF fractions #8 and #9 from *B. pilosicoli*, a separation on 7.5% acrylamide gels was also performed to better resolve the bands in the high-mass range of the gels (images at right in Figures 5B and 5E.

Supplementary Figure S1

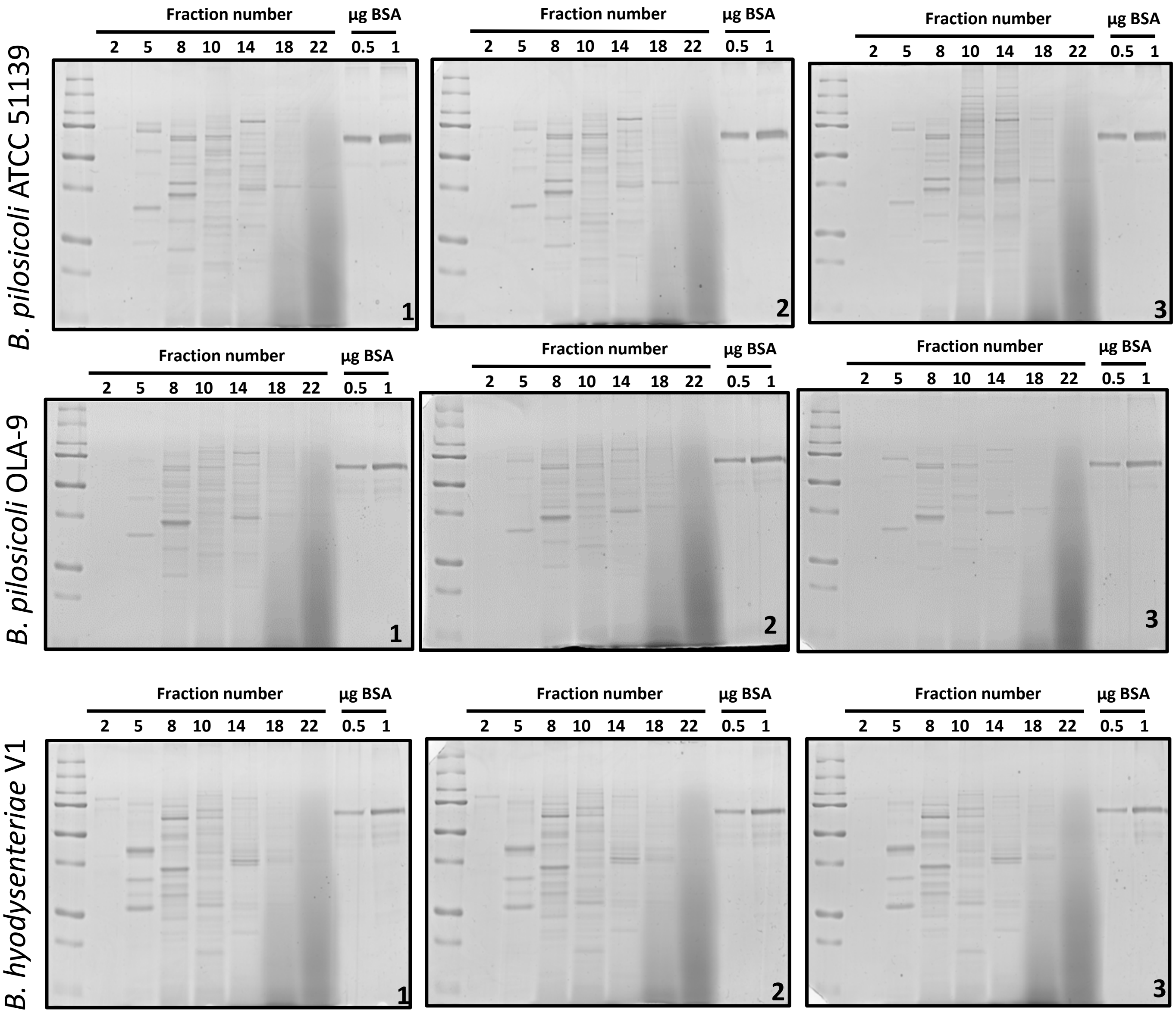

Supplementary Figure S2

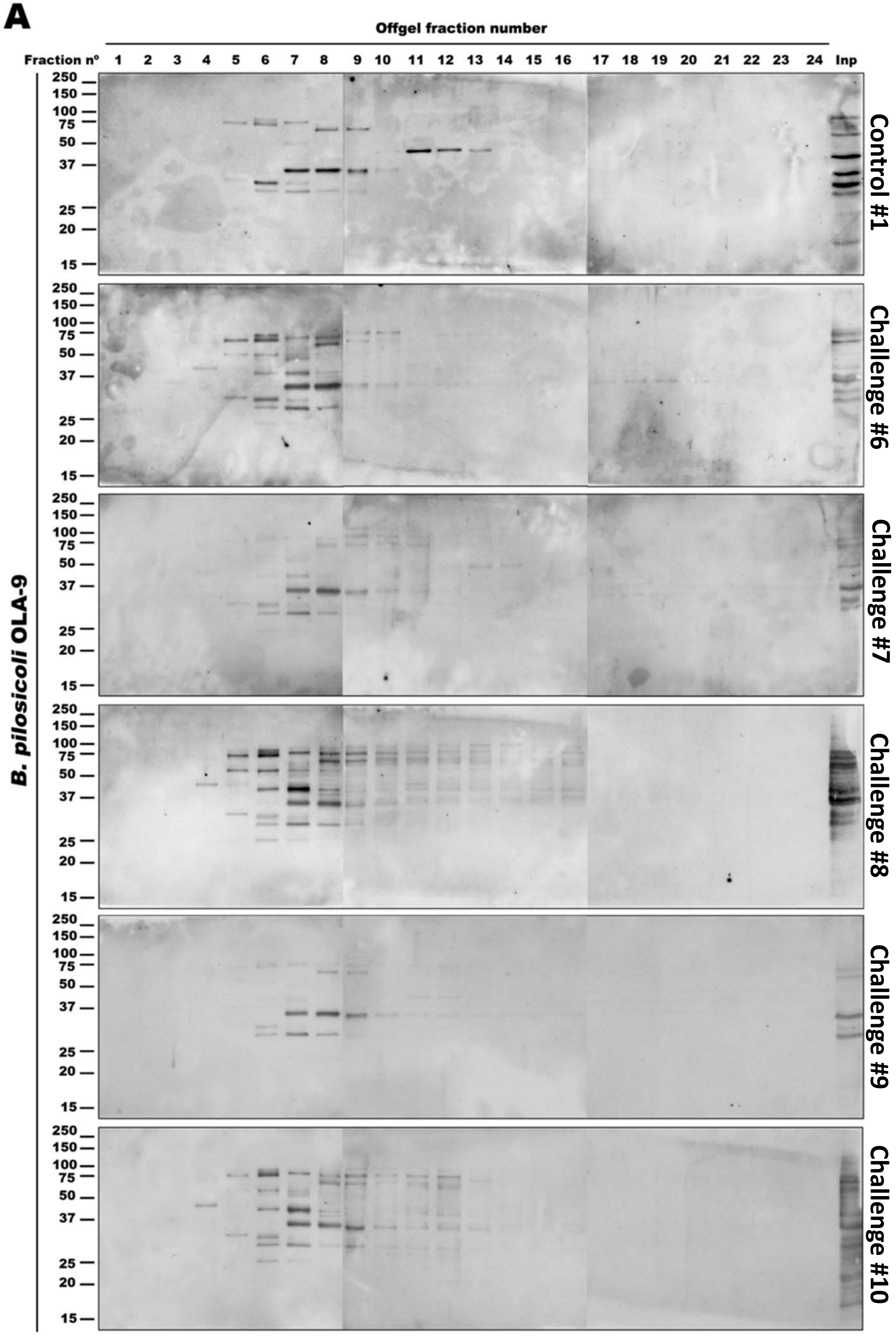

Supplementary Figure S2

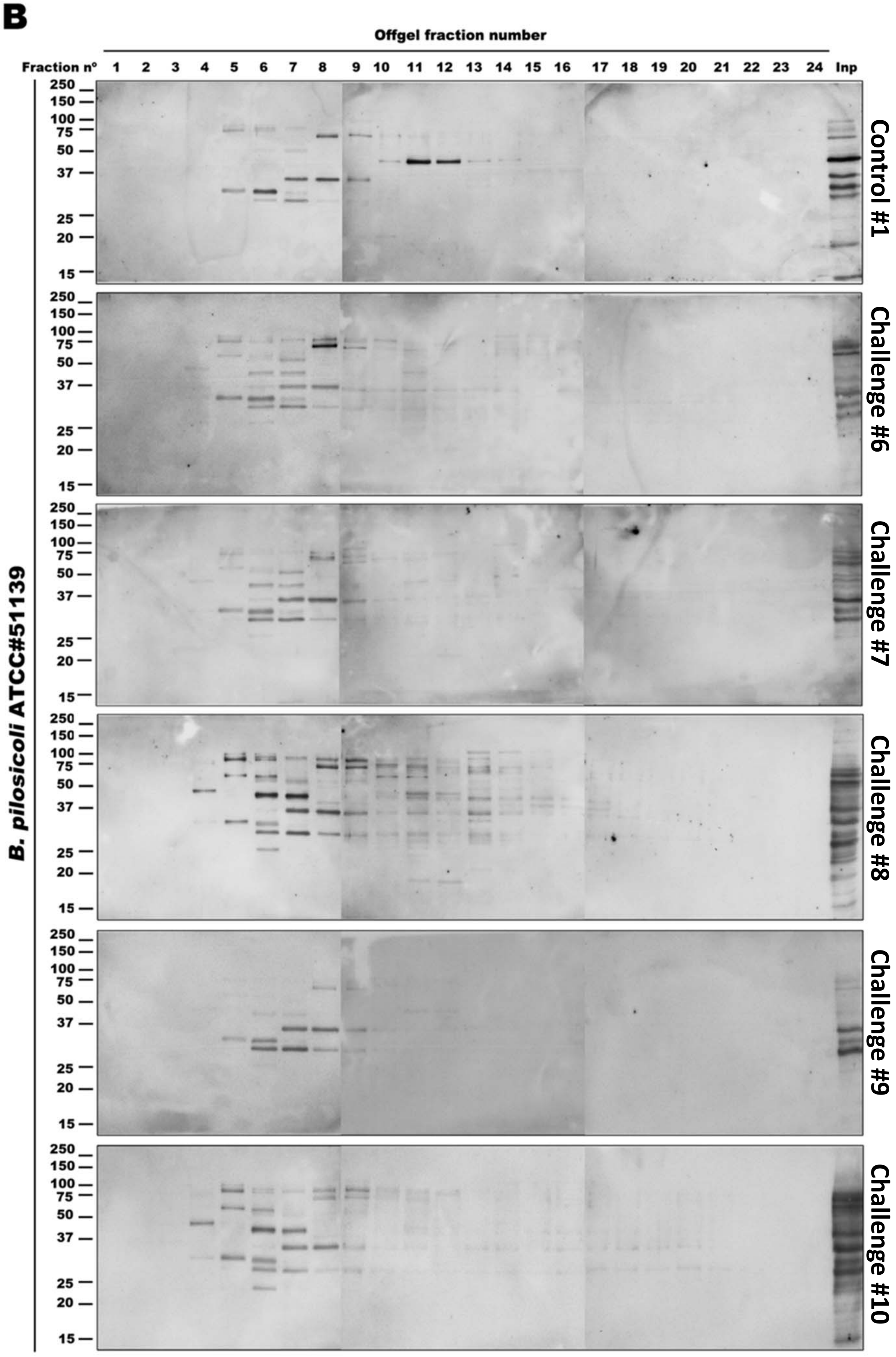

Supplementary Figure S2

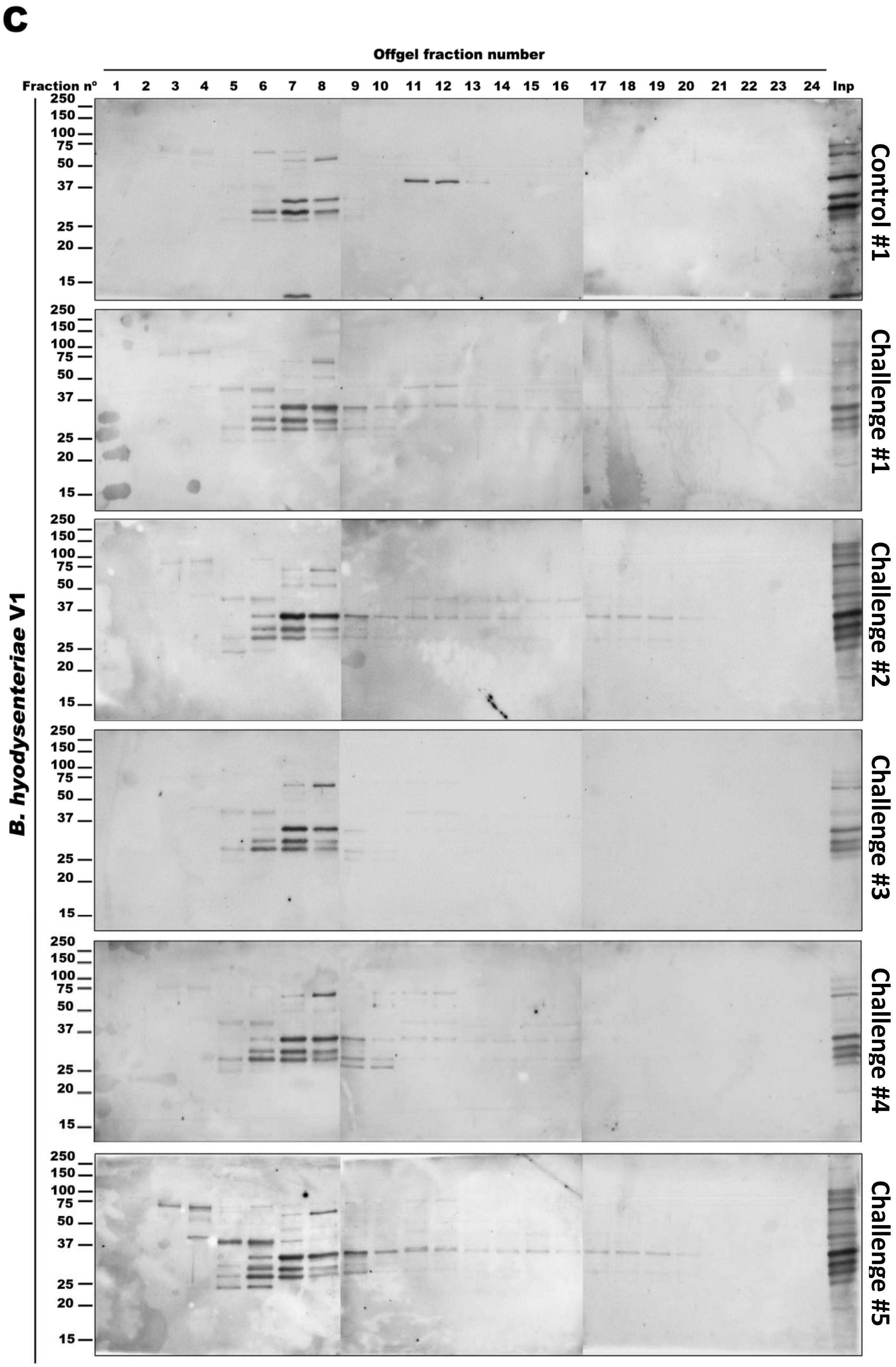

Supplementary Figure S3

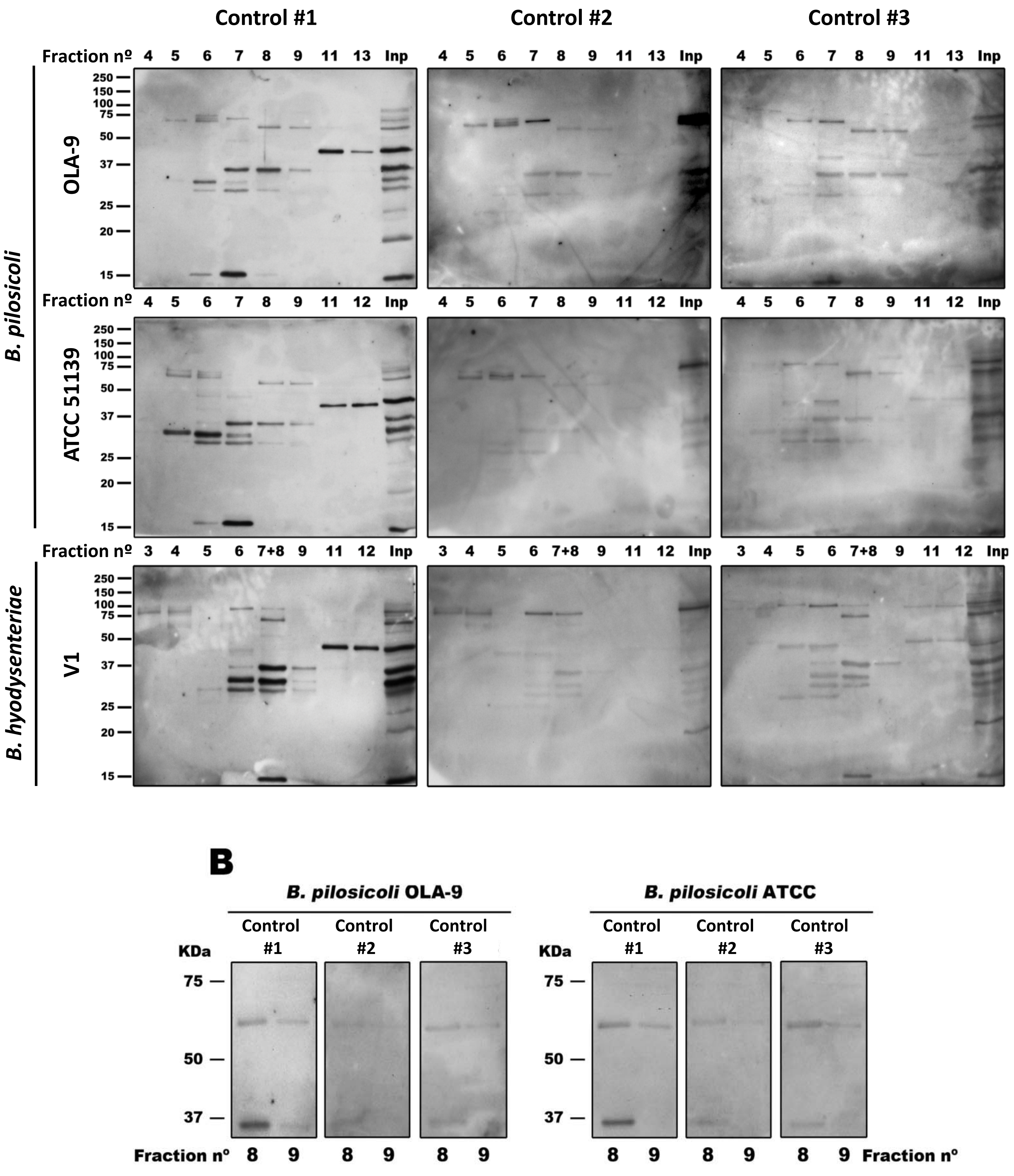

Supplementary Figure S4

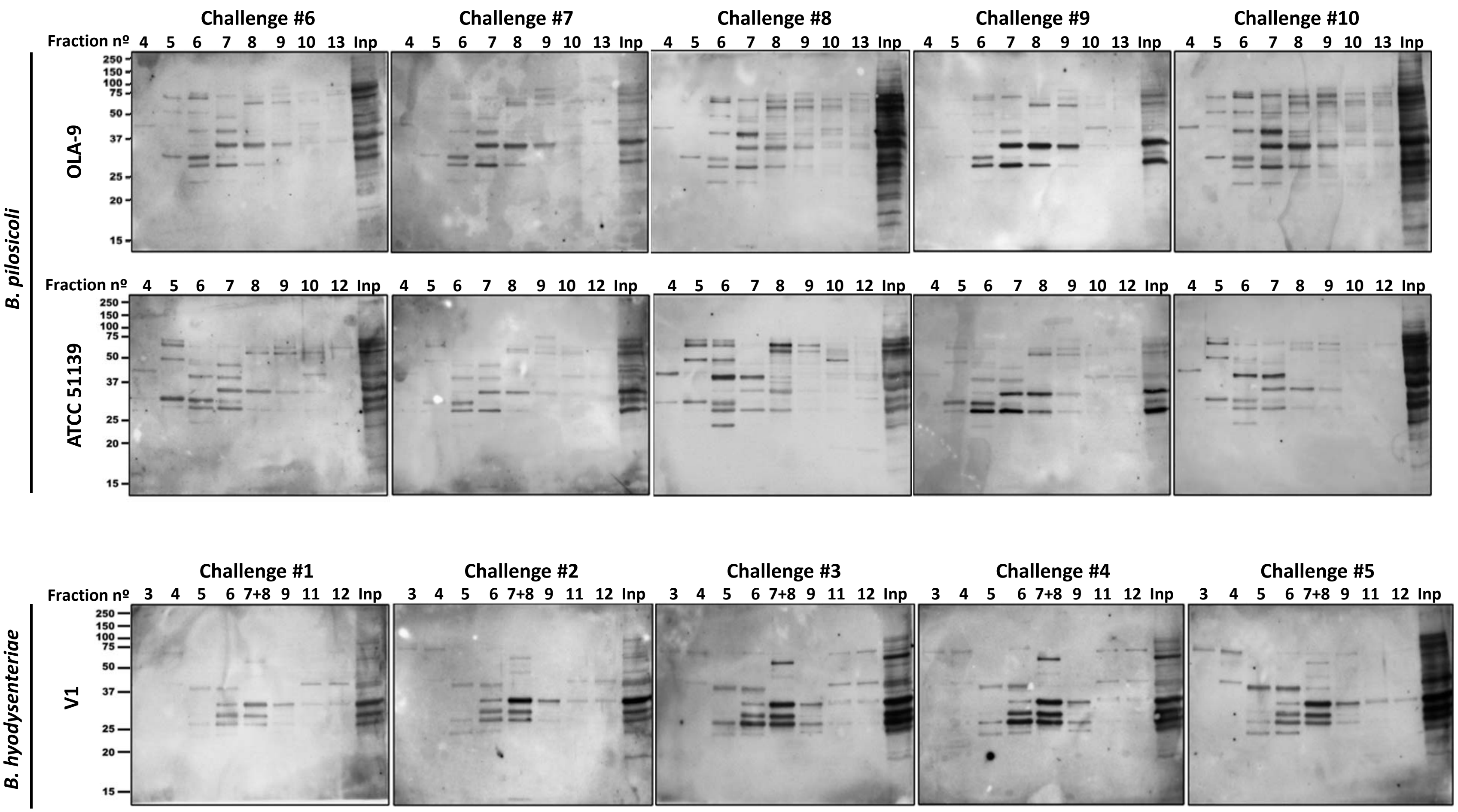

Supplementary Figure S5A

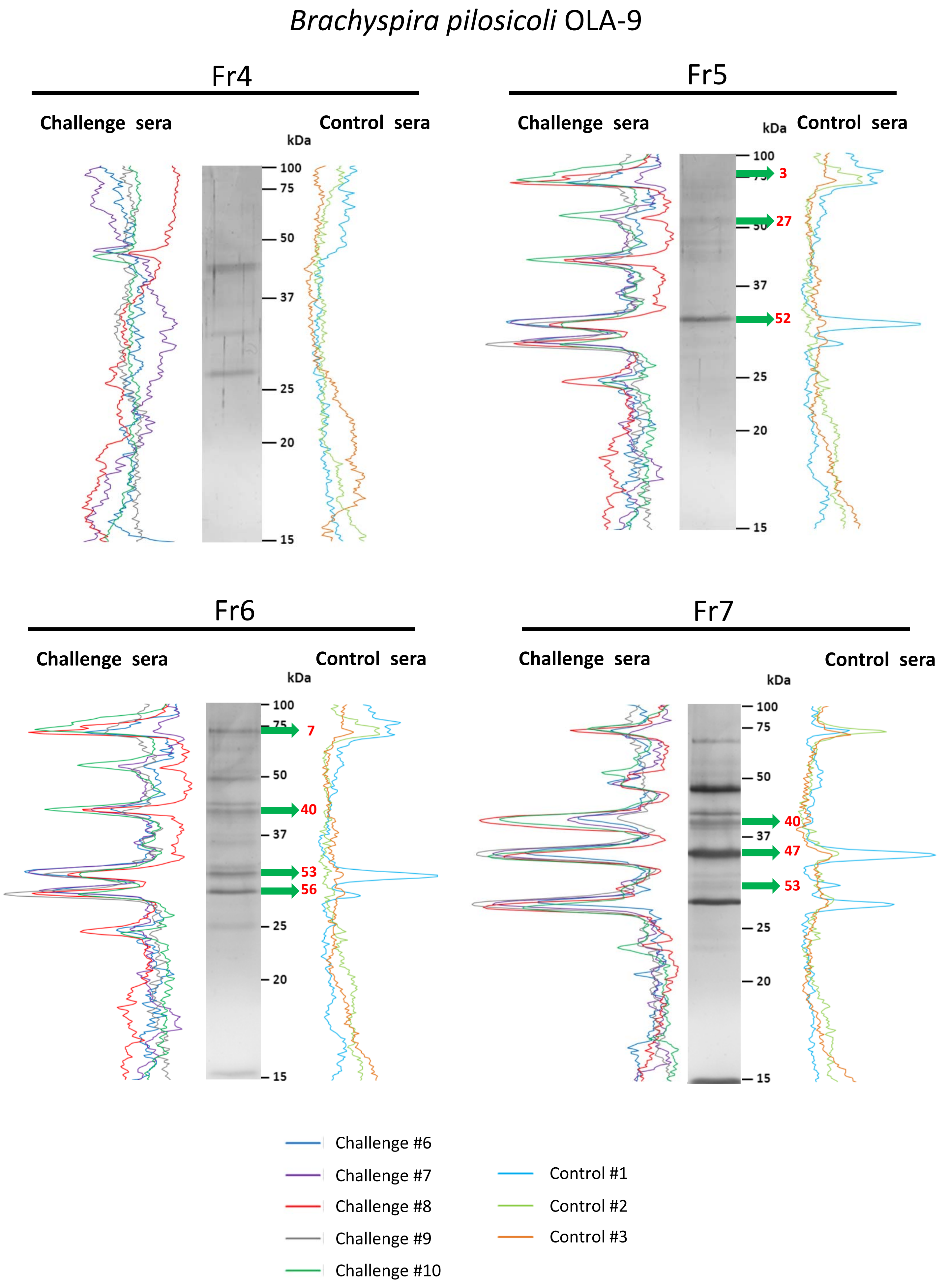

Supplementary Figure S5B

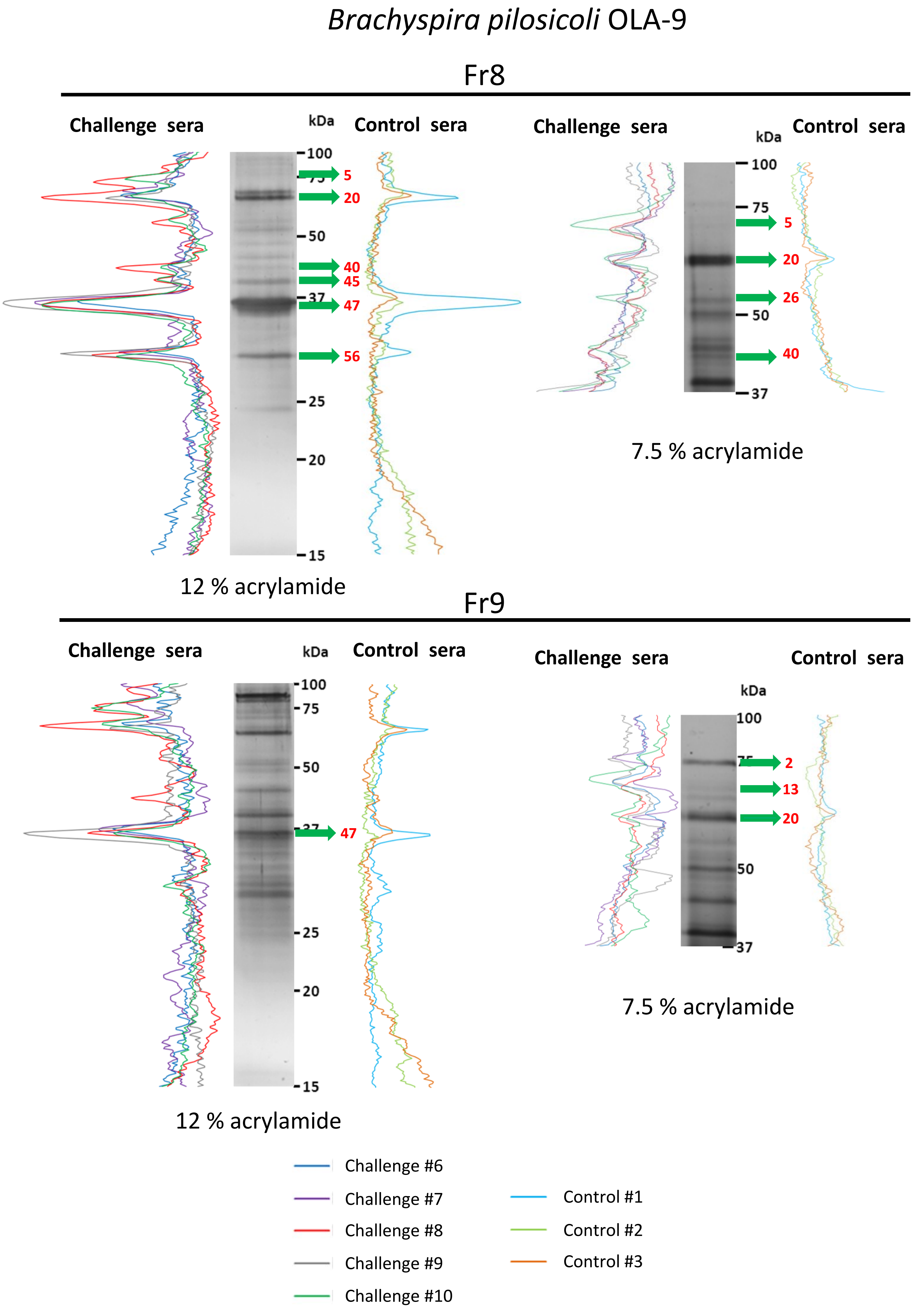

Supplementary Figure S5C

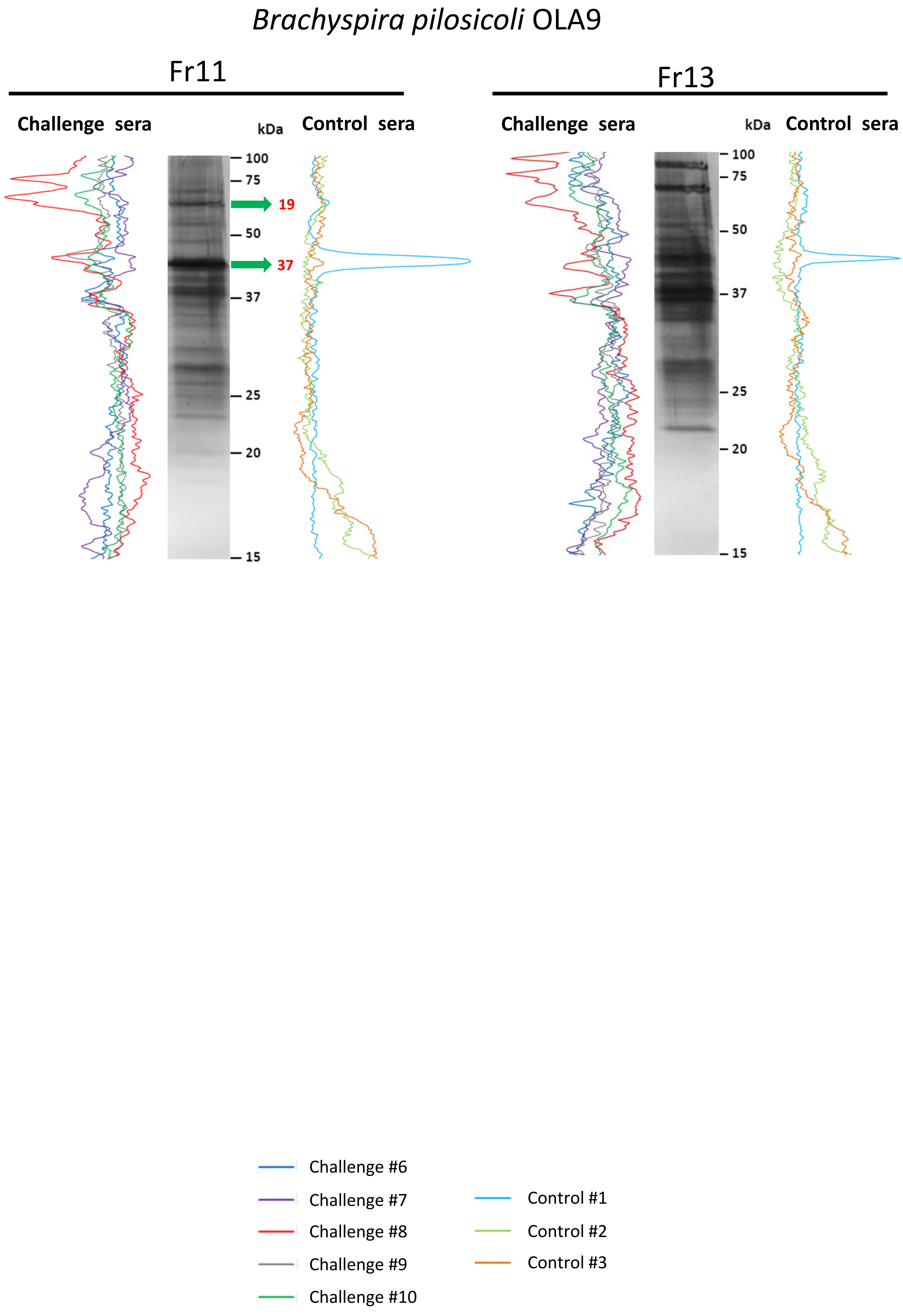

Supplementary Figure S5D

Brachyspira pilosicoli ATCC 51139

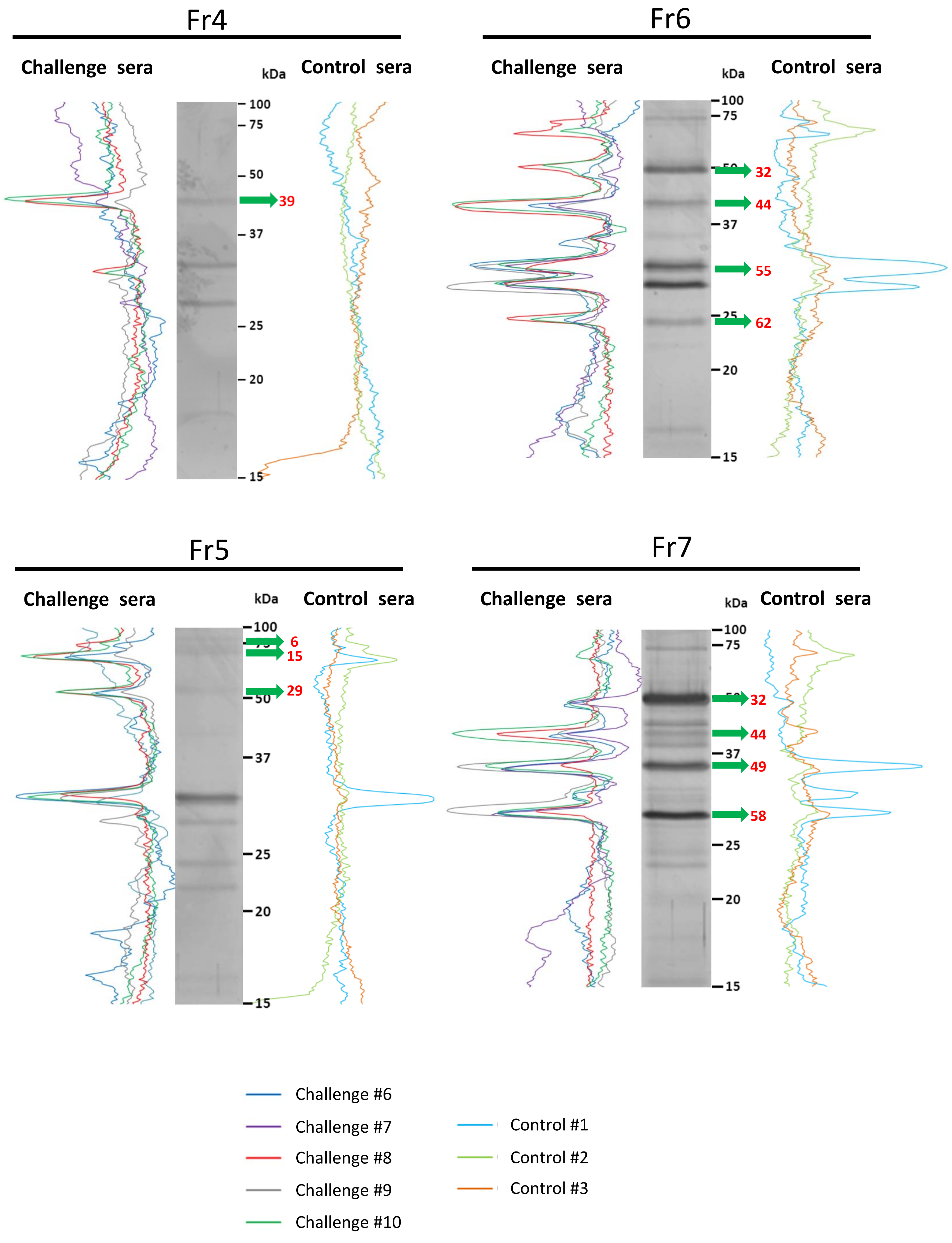

Supplementary Figure S5E

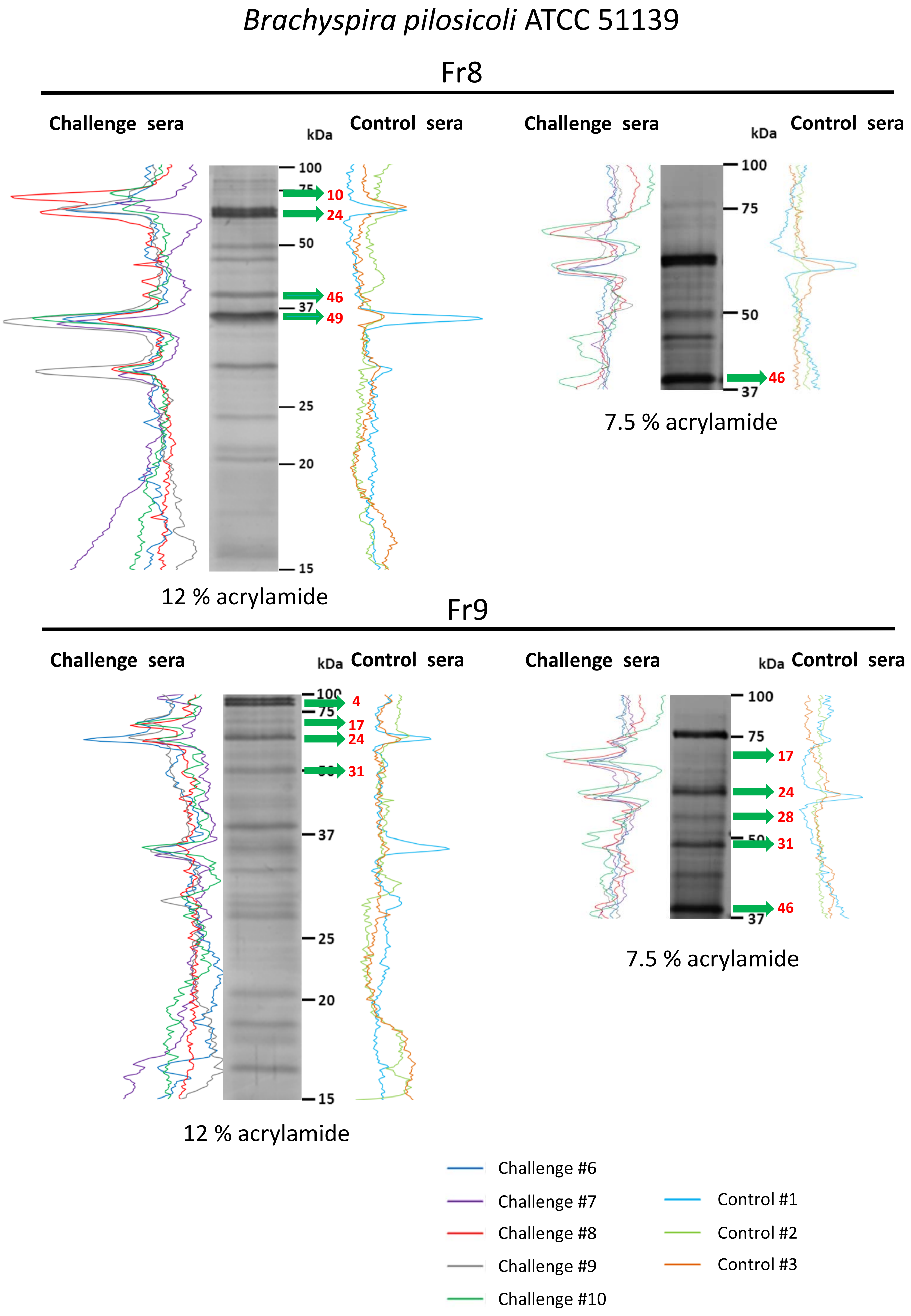

Supplementary Figure S5F

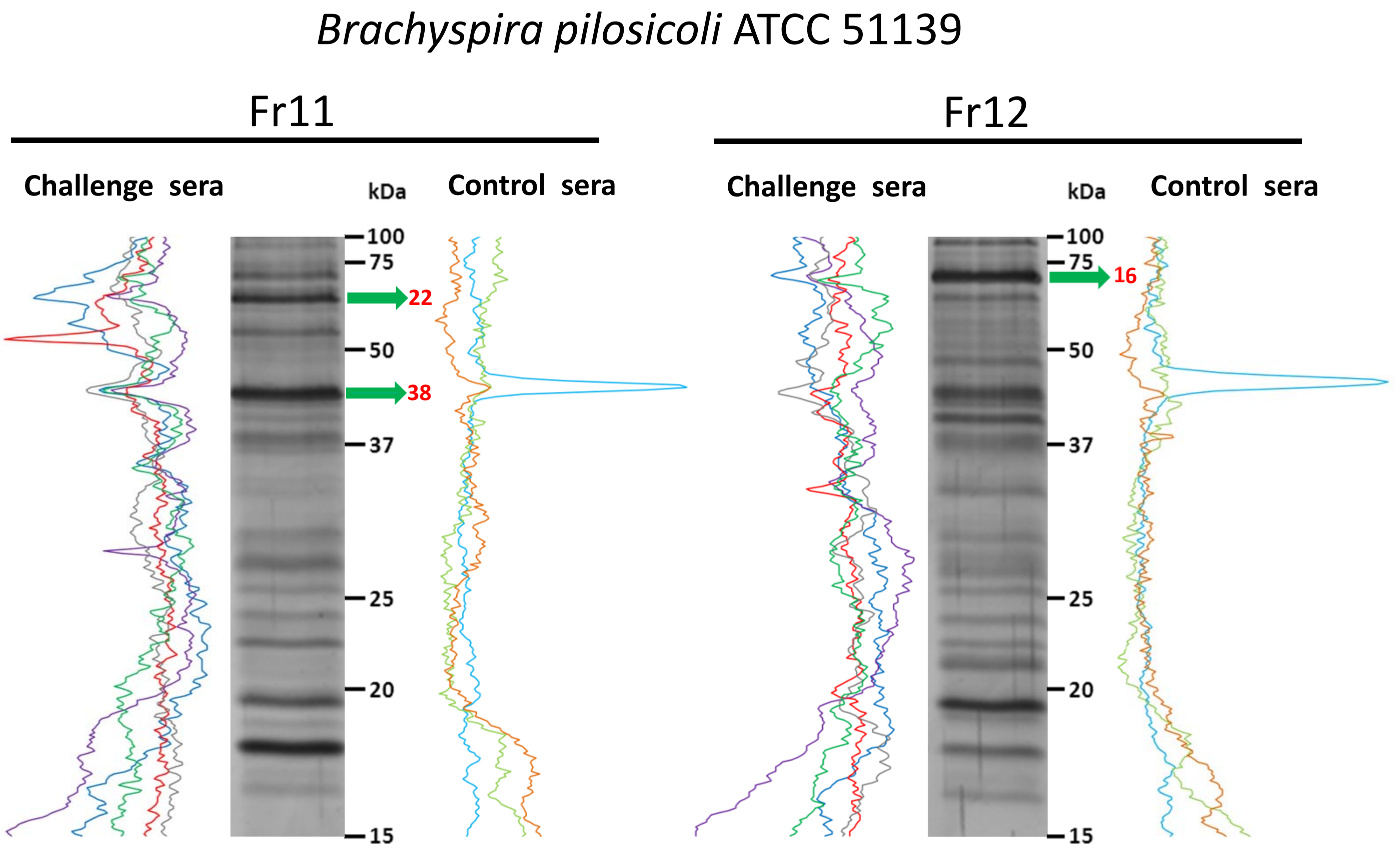

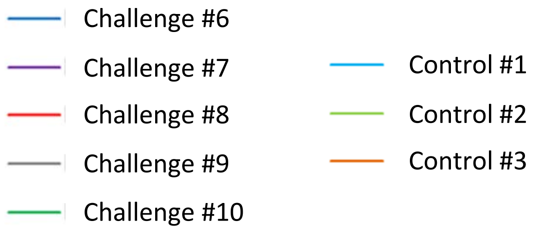

Supplementary Figure S5G

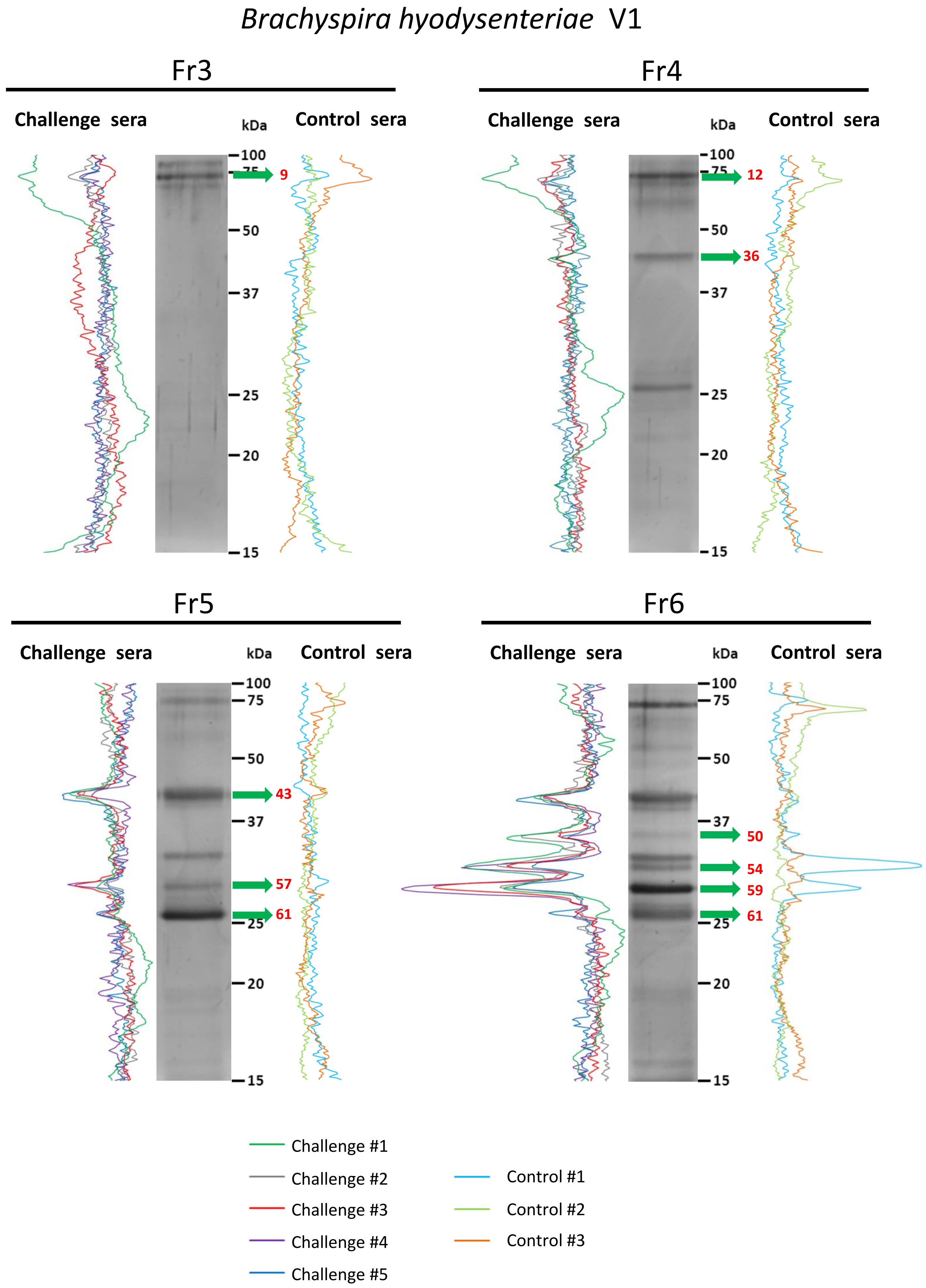

Supplementary Figure S5H

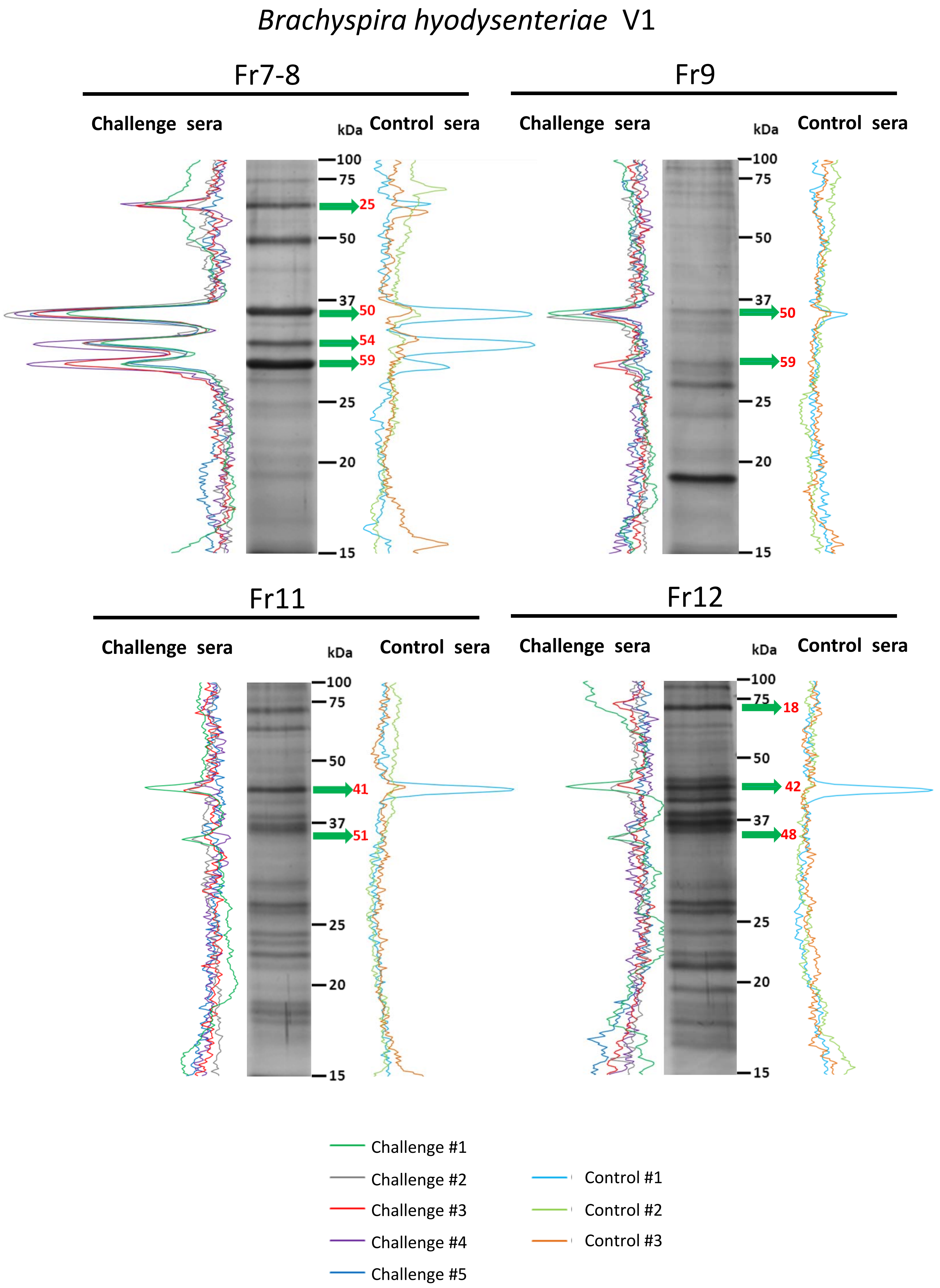

Supplement: Supplementary file 2 [file Presentation1.PDF]
